# Self-Assembled Liposomes Enhance Electron Transfer for Efficient Photocatalytic CO_2_ Reduction

**DOI:** 10.1021/jacs.2c01725

**Published:** 2022-05-20

**Authors:** Santiago Rodríguez-Jiménez, Hongwei Song, Erwin Lam, Demelza Wright, Andrea Pannwitz, Shannon A. Bonke, Jeremy J. Baumberg, Sylvestre Bonnet, Leif Hammarström, Erwin Reisner

**Affiliations:** † Yusuf Hamied Department of Chemistry, University of Cambridge, Lensfield Road, Cambridge CB2 1EW, U.K.; ‡ Department of Chemistry − Angstrom Laboratory, Uppsala University, Box 523, 751 20 Uppsala, Sweden; § Nanophotonics Centre, Department of Physics, Cavendish Laboratory, University of Cambridge, Cambridge CB3 0HE, U.K.; ∥ Leiden Institute of Chemistry, Leiden University, Einsteinweg 55, 2333 CC Leiden, The Netherlands

## Abstract

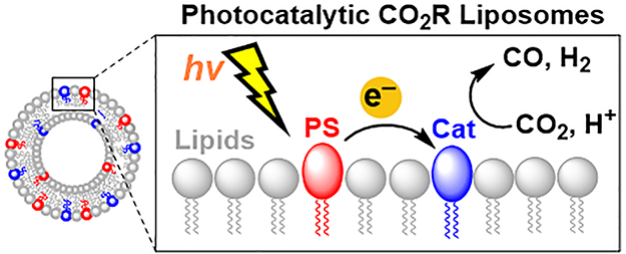

Light-driven conversion of CO_2_ to chemicals provides a sustainable alternative to fossil fuels, but homogeneous systems are typically limited by cross reactivity between different redox half reactions and inefficient charge separation. Herein, we present the bioinspired development of amphiphilic photosensitizer and catalyst pairs that self-assemble in lipid membranes to overcome some of these limitations and enable photocatalytic CO_2_ reduction in liposomes using precious metal-free catalysts. Using sodium ascorbate as a sacrificial electron source, a membrane-anchored alkylated cobalt porphyrin demonstrates higher catalytic CO production (1456 vs 312 turnovers) and selectivity (77 vs 11%) compared to its water-soluble nonalkylated counterpart. Time-resolved and steady-state spectroscopy revealed that self-assembly facilitates this performance enhancement by enabling a charge-separation state lifetime increase of up to two orders of magnitude in the dye while allowing for a ninefold faster electron transfer to the catalyst. Spectroelectrochemistry and density functional theory calculations of the alkylated Co porphyrin catalyst support a four-electron-charging mechanism that activates the catalyst prior to catalysis, together with key catalytic intermediates. Our molecular liposome system therefore benefits from membrane immobilization and provides a versatile and efficient platform for photocatalysis.

## Introduction

The sunlight-driven reduction of CO_2_ to value-added products is a promising and sustainable path to mitigate anthropogenic CO_2_ emissions and produce renewable platform chemicals. The use of lipid membranes such as liposomes as artificial photosynthetic scaffolds is an elegant and bioinspired approach to design photosynthetic systems.^1^ These synthetic liposomes can self-assemble into biomimetics of thylakoid membranes while allowing tunability of their supramolecular and photocatalytic components. Crucially, they facilitate charge separation^2−4^ and can spatially separate (compartmentalize) redox half reactions,^5,6^ thereby avoiding cross reactivity (such as back reactions and charge recombination)^7−10^ that severely limits homogeneous photocatalysis.^1,11^

Liposomes have been explored as scaffolds for different photochemical processes, including charge separation dynamics across lipid membranes^5,7,9,12,13^ and molecule-based photocatalytic systems for water oxidation and reduction.^14−17^ More recently, full water splitting was reported using liposomes embedded with photocatalytic metal organic frameworks.^6^ However, CO_2_ photoreduction liposome systems remain scarce,^18,19^ and understanding has been limiting, hence preventing further development. The previously reported examples utilized a membrane-bound ruthenium tris-bipyridine dye and Lehn-type rhenium bipyridine catalyst, which generated moderate amounts of CO under visible light irradiation (CO turnover number [TON_CO_] = 190 after 15 h^18^ and 15 after 3 h^19^). In comparison, the library of homogeneous CO_2_ photocatalytic systems is extensive, and earth-abundant catalysts based on terpyridine and porphyrin ligand families display high catalytic activity and product selectivity under aqueous conditions.^20−25^

**Figure 1:**
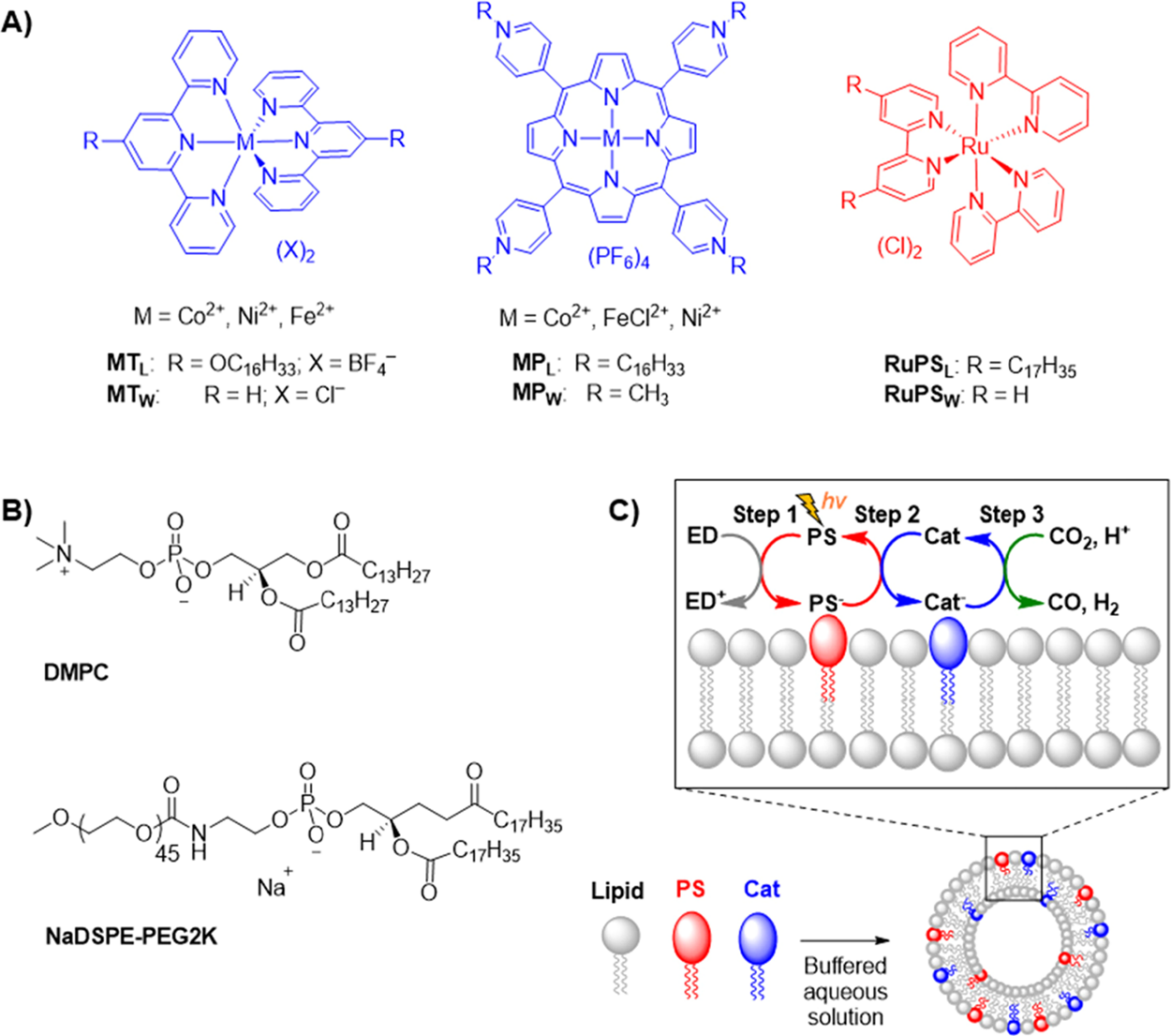
Structures of molecular components and phospholipids, and schematic representation of molecularly decorated liposomes. (A) Lipophilic (subscript L) and water-soluble (subscript W) bis-terpyridine- and porphyrin-based molecular catalysts (blue, left and center) and ruthenium tris-bipyridine photosensitizer (red, right). (B) Phospholipids 1,2-dimyristoyl-sn-glycero-3-phosphocholine (DMPC) and 1,2-distearoyl-sn-glycero-3-phosphoethanolamine-N-[methoxy(polyethylene glycol)-2000] (NaDSPE-PEG2K). (C) Scheme of a molecularly functionalized liposome system, with the inset highlighting a simplified representation of different electron transfer steps occurring during photocatalytic CO_2_ reduction at the water–membrane interfaces. Cat = catalyst, PS = photosensitizer, and ED = electron donor, i.e., sodium ascorbate (NaHAsc).

Herein, we exploit the tunability of molecular catalysts to synthesize alkylated CO_2_ reduction catalysts to self-assemble with alkylated photosensitizers in liposome membranes. These new catalysts are based on state-of-the-art homogeneous catalysts,^20,21^ with modified ligand scaffolds. The beneficial effects of self-assembly and flexibility of the approach,^1^ which enable facile variation of active sites in the liposomes, are demonstrated by a series of new alkylated precious metal-free catalysts based on terpyridine and porphyrin ligands (Figure 1A). Photocatalysis results comparing the performance between alkylated catalysts and water-soluble catalyst analogues are provided, and time-resolved/steady-state emission (photoluminescence) and transient absorption spectroscopies are utilized to determine the beneficial effects of self-assembly on charge separation. These techniques provide unprecedented insights into the photoinduced charge-transfer dynamics at the water–membrane interfaces. Key interactions between the sacrificial electron donor sodium ascorbate (NaHAsc), membrane-bound [Ru(bipyridine)_3_]^2+^-type photosensitizer, and catalyst molecules are examined to explain the superior photocatalytic activity of liposomes compared to their homogeneous analogues. Furthermore, the most active catalyst, 5,10,15,20-(tetra-N-hexadecyl-4-pyridinium)porphyrin cobalt(II) (**CoP_L_**), is comprehensively studied on transparent electrodes using in situ UV–vis–NIR and resonance Raman spectroelectrochemistry to understand its catalytic behavior, an approach that still remains scarce.^26−32^ In combination with density functional theory (DFT), these methods reveal important reaction intermediates during CO_2_ reduction and an unusual precatalytic four-electron charging mechanism that precedes its catalytic activity.

## Results and Discussion

### Synthesis and Assembly of Photocatalytic Liposomes

The tunability of molecular catalysts allows the periphery of the catalyst to be functionalized for self-assembly while maintaining a functional catalytically active site. The 3d transition metal complexes of Fe, Co, and Ni have emerged as active CO_2_ reduction catalysts with terpyridine^20,23,33^ and porphyrin^21,24^ ligands (hereinafter denoted as T and P, respectively), with no reports yet implementing them in self-assembled photocatalytic CO_2_ reduction liposome systems. To increase their lipophilicity and facilitate assembly at the water–membrane interface in the liposomes, hexadecyl chains were introduced into the ligands to prepare a systematic series (denoted as **MT_L_** and **MP_L_**, where L = lipophilic, W = water-soluble, and M = Co, Ni, Fe; Figure 1).^1^ Full synthetic and characterization details are provided in the Supplementary Methods section (see Figures S1–S4).

**Figure 2:**
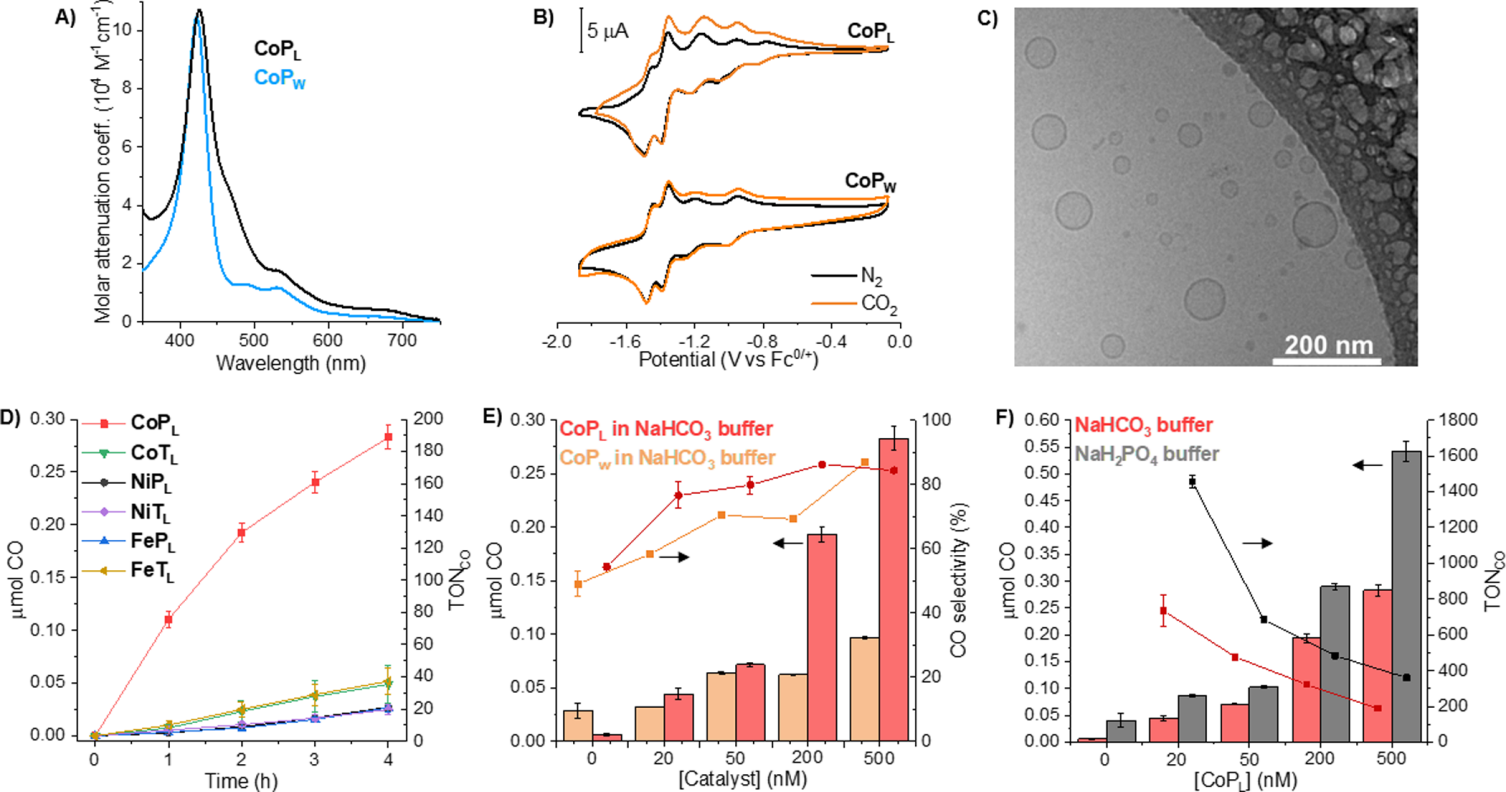
Comparison of UV–vis spectra and cyclic voltammetry (CV) of **CoP_L_** and **CoP_W_** catalysts and photocatalytic CO_2_ reduction results. (A) UV–vis spectra of porphyrin-based (blue) water-soluble **CoP_w_** and (black) lipophilic **CoP_L_** catalysts in acetone. (B) CV of **CoP_L_** and **CoP_W_** in (black) N_2_- and (orange) CO_2_-saturated 0.2 M TBAPF_6_ DMF solutions. (C) Cryo-TEM of unilamellar liposomes containing [DMPC] = 100 μM, [NaDSPE-PEG2K] = 1 μM, [**RuPS_L_**] = 10 μM, and [**NiT_L_**] = 2 μM. (D) Photocatalytic activity as a function of time of liposomes containing all alkylated catalysts at the same concentration (500 nM) in CO_2_-saturated 0.1 M NaHCO_3_. (E) Photocatalytic activity of (red) **CoP_L_** in liposomes and (orange) **CoP_W_** in homogeneous conditions as a function of catalyst concentration (0–500 nM) in CO_2_-saturated 0.1 M NaHCO_3_ after 4-h experiments. (F) Photocatalytic activity of liposomes containing **CoP_L_** as a function of catalyst concentration (0–500 nM) in CO_2_-saturated 0.1 M (red) NaHCO_3_ and (gray) NaH_2_PO_4_ buffer after 4-h experiments. Arrows in Figure 2E,F indicate the dataset’s *y* axis. CV experimental conditions: working electrode: glassy carbon (φ = 3 mm), counter electrode: Pt mesh, reference electrode: Ag/AgNO_3_ (10 mM) in 0.2 M TBAPF_6_ acetonitrile. Fc^+/0^ couple = +0.07 V vs Ag/AgNO_3_. Scan rate: 100 mV s^–1^. Photocatalytic experimental conditions: (liposomes, plots D-F) [DMPC] = 100 μM, [NaDSPE-PEG2K] = 1 μM, [**RuPS_L_**] = 10 μM, [Catalyst] = 500 nM in plot D or 20–500 nM in plots E and F. (Homogeneous, plot E) [**RuPS_W_**] = 10 μM, [Catalyst] = 20–500 nM. CO_2_-saturated 0.1 M NaHAsc and 0.1 M NaHCO_3_ (pH = 6.7) or NaH_2_PO_4_ (pH = 6.3) at 25 °C.

The UV–vis spectra and cyclic voltammograms (CVs) are comparable for the alkylated and water-soluble analogues in all cases, including the photosensitizers (Figures 2A,B and S5–S15), which indicates that the catalytically active site remains largely unchanged. Focusing on the most active catalysts **CoP_L_** and **CoP_W_** (see below), analogous absorption features are observed by UV–vis spectroscopy in acetone (Figure 2A, Soret bands: ε_426nm_ = 1.07 × 10^5^ and ε_423nm_ 1.05 × 10^5^ M^–1^ cm^–1^, respectively), as well as analogous electrochemical response. The CV of **CoP_L_** in N_2_- and CO_2_-saturated dimethylformamide (DMF) shows five reversible redox processes centered at −0.82, −0.99, −1.20, −1.38, and −1.49 V vs Fc^0/+^ (Figure 2B).^21,34^ The first two processes correspond to the same metal-centered single-electron reduction process (Co^II/I^), possibly due to different electroactive environments created by the (de)coordination of DMF molecules and the different arrangement of the long alkyl tails in solution.^34^ The other three redox waves are assigned based on the literature to a one-electron porphyrin-centered single-electron reduction (**P_W_**^0/–^) and two pyridinium-centered two-electron reductions (Table S1).^21,34^ Integration of the square wave voltammetry (SWV) scans of **CoP_L_** and **CoP_W_** and comparison of the relative ratios between the charge passed during chronoamperometry measurements in DMF confirm that both molecules can store up to six electrons (Figure S16 and Tables S2 and S3).

The liposomes are fabricated by extrusion using two different phospholipids (Figure 1B) to increase the liposomes’ stability and the affinity between the membrane and the metal complexes, which are mixed with the two phospholipids before extrusion (see the Supplementary Methods section).^14−16,35^ The first lipid is 1,2-dimyristoyl-sn-glycero-3-phosphocholine (DMPC), which is a zwitterionic lipid at neutral pH, has a transition-phase temperature of 24 °C, and is used to form the bulk of the membrane bilayers. The second lipid, 1,2-distearoyl-sn-glycero-3-phosphoethanolamine-N-[methoxy(polyethylene glycol)-2000] (NaDSPE-PEG2K), is an anionic and bulky lipid used as a dopant (<1% mol). The use of NaDSPE-PEG2K has a twofold benefit as electrostatic attraction improves immobilization of the positively charged molecular components, whereas its long methoxy polyethylene groups help diminish liposome aggregation.^1^ Dynamic light scattering measurements showed that extruded liposomes have average diameters of 149 ± 11 nm in 0.1 M NaHCO_3_ and 127 ± 9 nm in 0.1 M NaH_2_PO_4_ and 0.1 M NaHAsc (Tables S4–S6). These sizes are consistent with Cryo-TEM (Figure 2C). Dynamic light scattering also showed that liposome size, with and without dye and catalyst molecules, is not affected after 4 h of visible light irradiation (<10% size variation), which highlights the photostability of the lipids under our experimental conditions.

Furthermore, initial screening of molecule-containing liposomes fabricated with DMPC and two different lipids, i.e., 1,2-dilauroyl-sn-glycero-3-phosphocholine and 1,2-dipalmitoyl-sn-glycero-3-phosphocholine, showed that all three doped liposomes were in the fluid liquid crystal phase at room temperature, possibly due to the presence of 10% **RuPS_L_** (Figure S17). Importantly, DMPC-based liposomes exhibited better catalytic activity and electron transfer kinetics than the other two; hence, we selected DMPC as the main liposome building block thereafter (see Supplementary Note 1).

### Photocatalytic CO_2_ Reduction in Liposomes

The photocatalytic activity of liposomes was assessed in CO_2_-saturated aqueous NaHCO_3_ buffer solution (25 °C) containing sodium ascorbate (NaHAsc) as a sacrificial electron donor (pH ≈ 6.7) under visible light irradiation from a solar light simulator (AM 1.5G, 100 mW cm^–2^, λ > 400 nm UV filter, IR water filter) (Figures S18–25). The photosensitizer is a single electron donor; therefore, photocatalytic tests employ an excess of photosensitizer to drive the 2e^–^ reduction of CO_2_ to CO. During catalyst screening, a 20:1 photosensitizer to catalyst ratio was used to minimize electron transfer limitations and allow the nature of the catalysts to limit system performance.

CO evolved as the major photocatalytic CO_2_ reduction product from all six alkylated catalysts (Figures 1A and 2D) and was analyzed by gas chromatography (GC), with moderate-to-high CO selectivity (62% for **NiP_L_** and 74–87% for all others; Tables 1, S7, and S8). H_2_ was a byproduct, and no other products were detected after 4 h of photocatalysis (such as methane using GC or formate using nuclear magnetic resonance (NMR) and ion chromatography). In contrast, analogous homogeneous systems containing water-soluble photosensitizer **RuPS_W_** and catalysts (**MP_W_** or **MT_W_**) produce lower amounts of CO and, in most cases, higher amounts of H_2_ under the same experimental conditions (Figure 2E, Tables 1, S8, and S9). This is exemplified by comparing **CoP_L_** in liposomes with its homogeneous analogue **CoP_W_**, reported previously,^21^ as **CoP_L_** shows more catalytic turnovers (TON_CO_ = 189 ± 8 vs 65 ± 1) and a higher CO formation rate (89 ± 18 vs 24 ± 1 nmol_CO_ h^–1^) under the same experimental conditions. This difference in performance can be ascribed to diffusion limitations for the homogeneous system, such as slower electron transfer kinetics between **RuPS_W_** and catalysts (see below).^3,4,18^ This can be probed indirectly by varying the catalyst concentration, with **CoP_L_**-containing liposomes being more active and CO selective at all concentrations (20–500 nM) with a directly proportional relationship between CO formation and **CoP_L_** concentration (Figure 2F). At 20 nM catalyst concentration, **CoP_L_** reaches a TON_CO_ of 735 ± 91 and CO selectivity of 78%, compared to a TON_CO_ of 529 ± 3 and CO selectivity of 58% for **CoP_W_**.

**Table 1:** Summary of Exclusion Control and Buffer-Dependent Experiments^a^

entry	PS^b^	catalyst (nM)	buffer	CO/nmol (TON_CO_)	H_2_/nmol (TON_H_2__)	PTON_CO_^c^	CO Sel. /%^d^
1	**RuPS_L_**	**CoP_L_** (500)	NaHCO_3_	283 (189)	55 (36)	19	84
2	**RuPS_L_**	**CoP_L_** (20)	NaHCO_3_	44 (735)	14 (225)	3	78
3	**RuPS_L_**	**CoP_L_** (500)	NaH_2_PO_4_	541 (361)	120 (80)	36	82
4	**RuPS_L_**	**CoP_L_** (20)	NaH_2_PO_4_	87 (1456)	26 (434)	6	77
5	**RuPS_W_**	**CoP_W_** (500)	NaHCO_3_	97 (65)	15 (10)	6	87
6	**RuPS_W_**	**CoP_W_**(20)	NaHCO_3_	32 (529)	23 (379)	2	58
7	**RuPS_W_**	**CoP_W_** (500)	NaH_2_PO_4_	199 (133)	97 (65)	13	73
8	**RuPS_W_**	**CoP_W_** (20)	NaH_2_PO_4_	19 (312)	146 (2425)	1	11
9^e^	**RuPS_L_**	**CoP_L_** (500)	NaHCO_3_	n.d. (−)	n.d. (−)		
10	–	**CoP_L_** (500)	NaHCO_3_	n.d. (−)	n.d. (−)		
11^f^	**RuPS_L_**	–	NaHCO_3_	6 (−)	5 (−)	<1	54
12^g^	**RuPS_L_**	**CoP_L_** (500)	NaHCO_3_	n.d. (−)	n.d. (−)		
13^f^	**RuPS_W_**	–	NaHCO_3_	28 (−)	31 (−)	2	48

a Results confirm the origin of CO and compare the buffer-dependent catalytic activity of **CoP_L_** in liposomes and **CoP_W_** in homogeneous conditions.

b In all cases, [PS] = 10 μM. [DMPC] = 100 μM and [NaDSPE-PEG2K] = 1 μM used with **RuPS_L_**; [NaHAsc] = 0.1 M in CO_2_-saturated aqueous 0.1 M NaHCO_3_ (pH ≈ 6.7) or 0.1 M NaH_2_PO_4_ (pH ≈ 6.3) buffer solution, λ > 400 nm, AM 1.5G, 100 mW cm^–2^, 25 °C.

c PTON_CO_ is the TON_CO_ based on PS and is calculated to be 2 × mol CO /mol PS.

d CO selectivity (%) = *n*_CO_/(*n*_CO_ + *n*_H_2__) × 100. “n.d.” stands for not detected.

e Experiments carried out in the dark.

f In experiments without a catalyst, the CO and H_2_ evolved likely come from **RuPS_L_** or **RuPS_W_** and unidentified photodegraded byproducts.^37,38^

g NaHAsc was absent.

Exchanging the CO_2_-saturated 0.1 M buffer from NaHCO_3_ to NaH_2_PO_4_ (pH ≈ 6.7 vs 6.3) provides a higher buffering capacity and minimizes proton gradients near the two-dimensional water–membrane interface.^36^ This change increases the rate of CO production for **CoP_L_** at varying catalyst concentrations while also maintaining high CO selectivity (Figure 2F). This leads to a TON_CO_ of 1456 ± 36 and CO selectivity of 77% for **CoP_L_** at 20 nM, compared to 312 ± 22 and 11% for **CoP_W_**. These results exceed previously reported Re(bipyridine)-based liposome systems,^18,19^ and match top performing homogeneous photocatalytic CO_2_ reduction systems in aqueous conditions (Table S10).

Exclusion control experiments for the **CoP_L_** system confirm that no gaseous products evolve in the absence of **RuPS_L_**, NaHAsc, or light irradiation (Table 1 for details). Photocatalysis with isotopically labeled ^13^CO_2_ shows the formation of ^13^CO as the only photocatalytic CO_2_ reduction product, which confirms that CO is produced from CO_2_ (Figure S26). The rate of CO formation in all cases decays over time, which can be attributed to the photodegradation of **RuPS_L_** during light irradiation. This hypothesis is confirmed by electronic absorption spectroscopy showing that after light irradiation in liposomes, containing both **RuPS_L_** and an alkylated catalyst, the 450 nm band belonging to **RuPS_L_** decreases in intensity irreversibly over time. This is in contrast to liposomes containing only **CoP_L_**, where the Soret band intensity does not diminish (Figure S27), consistent with previous reports.^15,37−39^ Additionally, while 0.1 M NaHAsc was chosen as the optimal concentration to obtain a high CO evolution rate and CO selectivity, variation of NaHAsc concentration (50–400 mM), as well as visible light intensity (20–100%), shows that CO and H_2_ formation is affected by both variables (Tables S11 and S12), confirming that formation of reduced **RuPS_L_^–^** species is limiting the overall reaction of the studied liposomes.

### Photoinduced Charge Transfer in Liposomes

**Figure 3:**
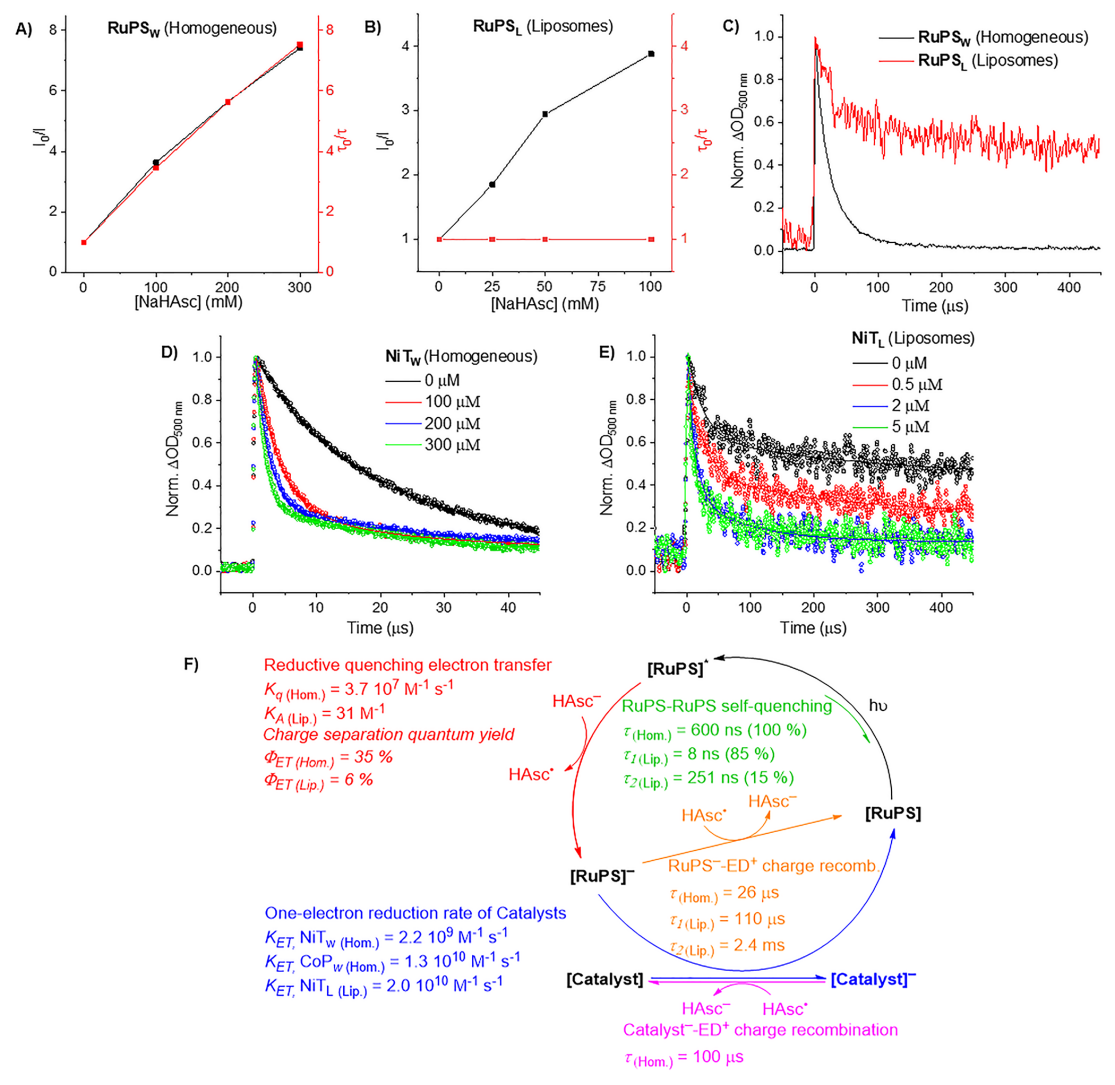
Photoinduced charge transfer in liposomes. (A and B) Stern–Volmer plots in the homogeneous environment and liposomes, respectively, from steady-state emission intensity (*I*_0_/*I*) and lifetime (τ_0_/τ) data as a function of NaHAsc concentration, where *I*_0_ and τ_0_ are the values in the absence of NaHAsc. (C) Normalized transient absorption kinetic traces collected for **RuPS**^–^ at 500 nm for 500 μs after laser excitation. (D) Normalized kinetic traces for **RuPS_W_^–^** at 500 nm (original ΔOD ≈ 0.025) obtained in the presence of [**NiT_W_**] = 0–300 μM. (E) Normalized kinetic traces for **RuPS_L_^–^** at 500 nm (original ΔOD ≈ 0.003) obtained in the presence of [**NiT_L_**] = 0–5 μM. (F) Summary of photoinduced charge-transfer dynamics of photocatalytic liposome and homogeneous systems (see also Table S14). (**Green**) Lifetime of excited photosensitizer molecules, and in brackets, the percent contribution for the short-lifetime component, in homogeneous (Hom.) and liposomes (Lip.) before self-quenching occurs, without the presence of HAsc^–^. Experimental conditions: (homogeneous) [**RuPS_W_**] = 30 μM; and (liposomes) [DMPC] = 100 μM, [NaDSPE-PEG2K] = 1 μM, [**RuPS_L_**] = 10 μM in Ar-saturated 0.1 M NaHCO_3_. (**Red**) Reductive quenching rate and adsorption rate constants (*k*_q_ and *K*_A_, respectively), and charge separation quantum yields (φ_ET_) for homogeneous and liposome systems (see Figure S31 and Supplementary Note 3). Experimental conditions: (homogeneous) [**RuPS_W_**] = 30 μM, [NaHAsc] = 0–0.3 M; and (liposomes) [DMPC] = 100 μM, [NaDSPE-PEG2K] = 1 μM, [**RuPS_L_**] = 10 μM, [NaHAsc] = 0–0.1 M in Ar-saturated 0.1 M NaHCO_3_. (**Orange**) Charge recombination timescale between reduced PS and oxidized quenchers in homogeneous and liposomes. Experimental conditions: (homogeneous) [**RuPS_W_**] = 30 μM; and (liposomes) [DMPC] = 100 μM, [NaDSPE-PEG2K] = 1 μM, [**RuPS_L_**] = 10 μM in Ar-saturated 0.1 M NaHAsc and 0.1 M NaHCO_3_. (**Blue**) One-electron transfer rate constants between the reduced PS and a catalyst. Experimental conditions: (homogeneous) [**RuPS_W_**] = 30 μM, [**NiT_W_**] = 0–300 μM or [**CoP_W_**] = 0–25 μM; and (liposomes) [DMPC] = 100 μM, [NaDSPE-PEG2K] = 1 μM, [**RuPS_L_**] = 10 μM, [**NiT_L_**] = 0–5 μM in Ar-saturated 0.1 M NaHAsc and 0.1 M NaHCO_3_. (**Pink**) Charge recombination timescale between the reduced catalyst (**NiT_W_**) and oxidized quencher in homogeneous conditions. Experimental conditions: [**RuPS_W_**] = 30 μM, [**NiT_W_**] = 100 μM in Ar-saturated 0.1 M NaHAsc and 0.1 M NaHCO_3_.

To determine the effects of membrane self-assembly on electron transfer steps, time-resolved and steady-state emission quenching studies (Stern–Volmer analysis) were carried out with water-soluble and lipophilic photosensitizers (i.e., **RuPS_W_** or **RuPS_L_**) (Figure S28). In both cases, [Ru^II^(bpy)_3_]^2+^ is photoexcited and reductively quenched by NaHAsc to form [Ru^II^(bpy)_2_(bpy^·^^–^)]^+^, with the photoluminescence intensity of photoexcited Ru(II) being dependent on the quenching rate.^40^ Examining homogeneous **RuPS_W_**, the quenching occurs by diffusional encounter with NaHAsc, as observed with indistinguishable steady-state and time-resolved Stern–Volmer plots (i.e., *I*_0_/*I* and τ_0_/τ as a function of [NaHAsc] in Figure 3A; bimolecular quenching rate constant *k*_q_ = 3.7 × 10^7^ M^–1^ s^–1^). In contrast, while the emission intensity is strongly decreased by increasing the concentration of NaHAsc, it does not have an obvious effect on the emission lifetime of **RuPS_L_** in liposomes (Figure 3B). This can be attributed to a high local concentration of HAsc^–^, which is electrostatically attracted to the charge-dense liposome membranes loaded with cationic **RuPS_L_** (coulombic association-driven static quenching with an association constant *K*_A_ of 31 M^–1^). This is further supported by comparing the quenching quantum efficiencies (; *I* = emission intensity) in liposomes of 100 mM anionic HAsc^–^ (φ = 0.74) with a 100 mM concentration of the cationic quencher methyl viologen (φ = 0.16; Figure S29). By contrast in homogeneous solution, methyl viologen shows a rate constant *k*_q_ ≈ 1.0 × 10^9^ M^–1^ s^–1^ with excited [Ru^II^(bpy)_3_]^2+^, which is twenty-seven-fold larger than that of NaHAsc.

Immobilizing complexes in liposomes increases their local concentration, which may increase the rate of self-quenching processes of **RuPS_L_**.^1^ This was examined by monitoring the phosphorescence decay rate at 600 and 650 nm in the absence of NaHAsc, which showed no difference for homogeneous **RuPS_W_**. In contrast, the decay for membrane-bound **RuPS_L_** was faster as the **RuPS_L_** concentration increased (DMPC/**RuPS_L_** molar ratios of 10:1, 20:1, and 40:1 were studied; Figure S30). Data fitting of the emission trace at 650 nm indicated a short-lifetime component attributed to self-quenching by a neighboring ground-state **RuPS_L_** molecule (see Supplementary Note 2 and Table S14). The contribution of this self-quenching component to the overall rate is smaller at higher concentrations of DMPC. This indicates that diluting **RuPS_L_** in the liposomes hinders self-quenching events, presumably by spatially separating them. This emphasizes the importance of balancing higher photosensitizer concentrations to maximize light absorption against self-quenching processes. Photocatalysis results showed that higher concentrations of DMPC (more liposomes), with constant total concentrations of **RuPS_L_** and **CoP_L_**, had higher catalytic activity consistent with the above findings (Figure S24 and Table S13).

Transient absorption spectroscopy (TAS) uses laser pulse excitation and measures the absorption of photogenerated species. This allows the lifetimes of the photoexcited [Ru^III^(bpy)_2_(bpy^·^^–^)]^2+*^ and reductively quenched [Ru^II^(bpy)_2_(bpy^·^^–^)]^+^ (**RuPS^–^**) to be compared in homogeneous solution and within liposomes.^41^ Reductive quenching of the photoexcited state by NaHAsc forms the formal **RuPS^–^**, which absorbs at 500 nm. **RuPS^–^** has a reduction potential of ≈−1.2 V vs SHE in 0.1 M NaHCO_3_,^22^ which provides enough driving force to reduce the catalysts. The conversion of **RuPS** to **RuPS^–^** (i.e., charge separation quantum yield or φ_ET_) is higher in homogeneous conditions than in liposomes (35 vs 6%) and may be ascribed to the charged liposome membranes. While liposomes favor static quenching (see above), they also hinder diffusion of oxidized ascorbate species and thereby lower their solvent-cage escape yield (Figure S31 and Supplementary Note 3). In contrast, the decay of **RuPS^–^** is far slower in liposomes than in homogeneous solution, with a substantial absorbance value remaining even 500 μs after the excitation pulse (Figure 3C). While the homogeneous **RuPS_W_^–^** decay is approximately single exponential (26 μs time constant), **RuPS_L_^–^** is strongly biphasic (Figure S32), with one phase similar to that in homogeneous solution (110 μs time constant, 23% contribution) and one much slower, which represents the majority of **RuPS_L_^–^** (2.4 ms time constant, 77% contribution). A tentative assignment is that the fast phase is the rapid recombination of immobilized **RuPS_L_^–^** and oxidized ascorbate molecules remaining near the reaction site at the same liposome, possibly at the interior liposome interface, while the slower recombination is between **RuPS_L_^–^** species and oxidized ascorbate molecules that have escaped into the bulk solution.^3^ Thereby, despite liposomes showing lower φ_ET_, the incorporation of charged dyes into the liposome membrane slows recombination processes and favors long-lived charge separated states, highlighting liposomes as more efficient systems for photoinduced charge separation.

Key to catalytic turnover is the electron transfer kinetics between the reduced photosensitizer **RuPS^–^** and catalyst, which can be probed using TAS to monitor the absorption decay of **RuPS^–^** in the presence of the catalyst. **NiT_W_** and **CoP_W_** were analyzed as homogeneous model catalysts because of the lack of visible absorption of **NiT_W_**, which complements that of **RuPS**, and the high catalytic performance of **CoP_W_**. The presence of either leads to a more rapid decay of **RuPS_W_^–^** species (Figures 3D and S33 and Supplementary Note 4) and is accompanied by the formation of new absorption bands at 450 and 470 nm for **NiT_W_^–^** and **CoP_W_^–^**, respectively, as well as the bleaching of the Soret band at ≈430 nm for **CoP_W_^–^** (Figures S34 and S35). In liposomes, all six alkylated catalysts accelerate the decay of the **RuPS_L_^–^** species, which is a solid indicator of electron transfer occurring from **RuPS_L_^–^** to the catalysts in close proximity (Figures 3E, S36, and S37). Exemplifying the beneficial forward electron transfer kinetics of membrane-bound species over homogeneous systems, the bimolecular electron transfer rate constant *k*_ET_ of membrane-bound **NiT_L_** is nine times faster than that of the homogeneous system **NiT_W_** (2.0 × 10^10^ vs 2.2 × 10^9^ M^–1^ s^–1^; Figure S38). This enables a comparable electron transfer yield for 5 μM **NiT_L_** in liposomes and 100 μM **NiT_W_** in homogeneous solution (ca. 80% in both cases; see Table S14 and Supplementary Note 5 for details). Notably, the *k*_ET_ of **CoP_W_** (1.3 × 10^10^ M^–1^ s^–1^) is six times faster than that of **NiT_W_** and highlights the larger driving force to reduce **CoP_W_** compared to **NiT_W_**.

Taken together, these findings (summarized in Figure 3F) show that self-assembly of the membrane-bound species strongly affects reductive quenching and self-quenching dynamics. They can also increase charge separation and recombination lifetimes. Crucially, despite the lower φ_ET_ of liposomes, the relatively high surface concentration of membrane-bound species in liposomes diminishes diffusion limitations that hinder homogeneous systems. This is due to shorter electron transfer distances between photosensitizers and catalysts, which greatly assists catalysis.^1,15^

### Mechanistic Studies of **CoP_L_**-Mediated CO_2_ Reduction

The high catalytic activity of **CoP_L_** prompted an investigation into its catalytic mechanism. The hydrophobic nature of its alkyl tails enables it to be immobilized via physisorption onto conductive supports such as transparent fluorine-doped tin oxide (FTO) or glassy carbon electrodes (GCE) after dropcasting. This allowed a mechanistic study coupling its electrochemical response to spectroelectrochemical (SEC) UV–vis and Raman spectroscopies. The results were rationalized by DFT calculations to examine the molecular changes that **CoP_L_** undergoes during CO_2_ reduction.

**Figure 4:**
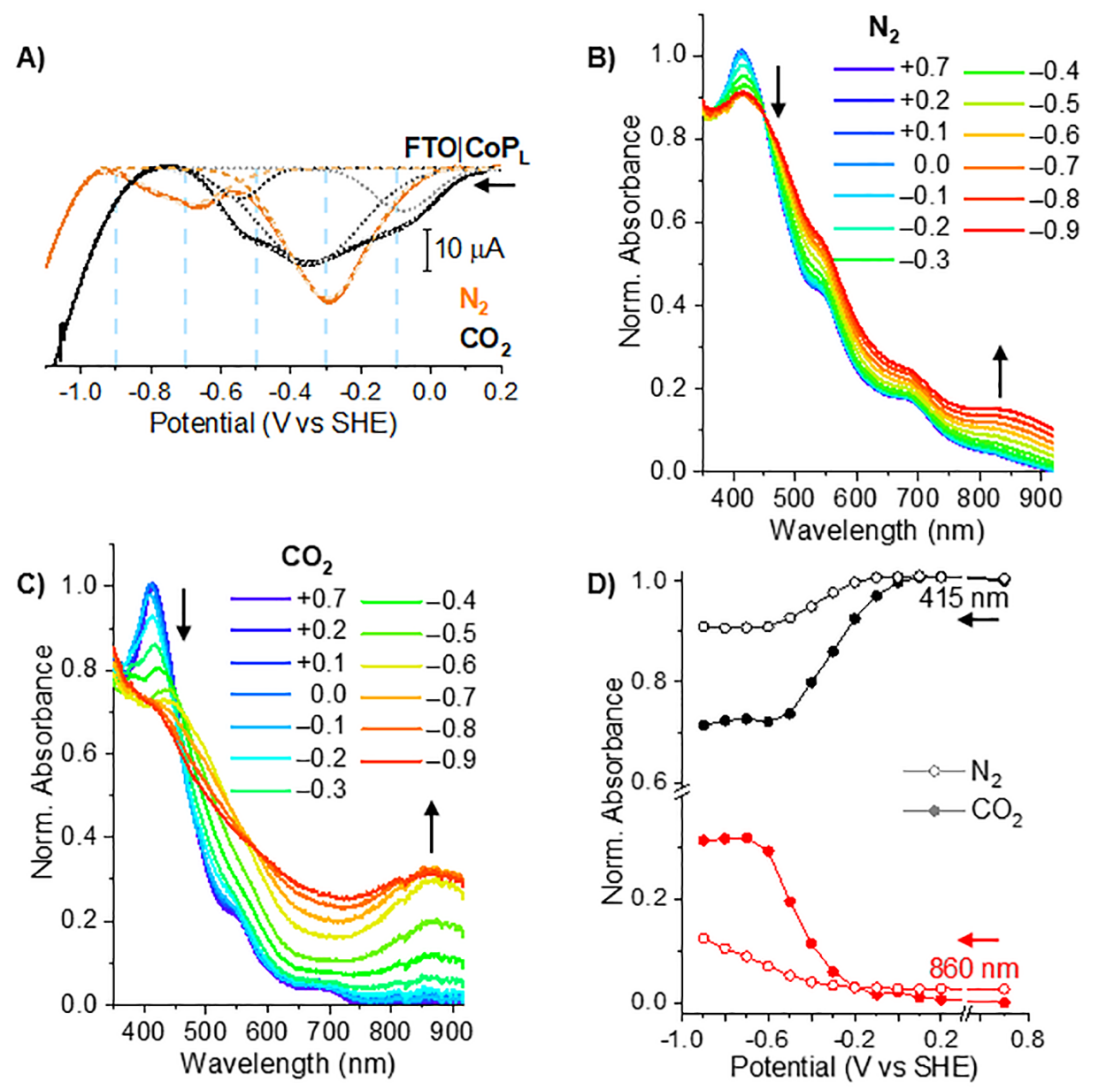
Square wave voltammetry (SWV) scans, chronoamperometry, and UV–vis–NIR spectroelectrochemistry (SEC) of **CoP_L_** immobilized on FTO. (A) SWV scans of **CoP_L_** dropcast on fluorine-doped tin oxide (FTO) in N_2_- and CO_2_-saturated 0.1 M NaHCO_3_. Deconvoluted SWV waves are shown as dashed lines. (B and C) In situ UV–vis–NIR SEC of **CoP_L_** dropcast on FTO in N_2_- and CO_2_-saturated 0.1 M NaHCO_3_, respectively. (D) Potential-dependent change in the normalized absorbance of the 415 nm (Soret band of **CoP_L_**) and 860 nm bands under CO_2_ compared to under N_2_ (filled vs open circles). N.B.: The small differences between plots B and C in the shape of the initial spectra at +0.7 V vs SHE are attributed to the different degrees of aggregation of **CoP_L_** molecules on the electrode surfaces after dropcasting.^44^

The SWV of FTO|**CoP_L_** in CO_2_-saturated 0.1 M NaHCO_3_ displays two reduction waves appearing at −0.1 and −0.35 V vs SHE (Figure 4A), which are assigned to a first metal-centered one-electron process and then a ligand-centered three-electron process (Table S3). FTO|**CoP_L_** and GCE|**CoP_L_** presented a catalytic CO_2_ reduction wave with an onset potential (*E*_onset_) at −0.9 V, and CO is detected by GC after chronoamperometry at −0.9 V (0.07 and 0.16 μmol CO cm^–2^, respectively), (Figure S39 and Table S15). In contrast, the equivalent blank chronoamperometry experiments using bare FTO and GCE evolved 0.01 and 0 μmol CO cm^–2^, respectively. For comparison, the immobilized catalyst shows similar redox processes and catalytic onset to **CoP_W_**.^21,24^ Chronoamperometry measurements at −0.9 V of the other five alkylated catalysts (**MP_L_** and **MT_L_**) on GCE reveal that they are less active and CO selective than **CoP_L_** (Figure S39C), indicating that **CoP_L_** has the lowest overpotential (η ≃ 0.37 V) to reduce CO_2_ of all six alkylated catalysts, which supports the trend observed in photocatalysis.

UV–vis–NIR SEC of FTO|**CoP_L_** compared changes in N_2_- and CO_2_-saturated 0.1 M NaHCO_3_ (pH 8.0 and 6.7, respectively) with chronoamperometric potential steps from +0.7 to −0.9 V vs SHE. The pH difference between N_2_- and CO_2_-saturated 0.1 M NaHCO_3_ solutions is caused by the hydration of CO_2_ to form carbonic acid.^36^ At +0.7 V, the complex is in the Co(II) state and features a Soret band at 415 nm and Q-band absorption at 535 and 670 nm (Figure 4B,C). The Soret band decreases in intensity as Co(II) is reduced to Co(I),^34^ starting at 0.0 V in N_2_ and −0.1 V in CO_2_, with complete reduction at −0.7 V in both N_2_ and CO_2_ (Figure 4D). This is consistent with our SWV and TAS, and the Soret band bleaching may correspond to formation of cobalt hydride species (Co–H) under N_2_, or binding of CO_2_ under CO_2_ saturation.^42^ Concurrent with Co^II/I^ reduction, the absorption bands at 510 and 575 nm become more intense (Figure S40), indicating reduction of the porphyrin ligand.^34^ Additionally, a new absorption at 860 nm grows in intensity from −0.3 to −0.7 V and is assigned to the reduction of two hexadecyl-N-pyridinium rings, in agreement with our SWV results and the literature.^21,34,43^ There are negligible absorption changes at 860 nm from −0.7 to −0.9 V, indicating that the remaining two hexadecyl-N-pyridinium rings are not reduced, even under catalytic conditions. Therefore, this analysis indicates a four-electron reduction that activates the cobalt porphyrin prior to catalysis in water (see below), in contrast to the six-electron reduction of homogeneous **CoP_L_** previously observed in DMF, which is not catalytically active (Figures 2B and S13 and Supplementary Note 6 with associated Figure S41 for further discussion).

**Figure 5:**
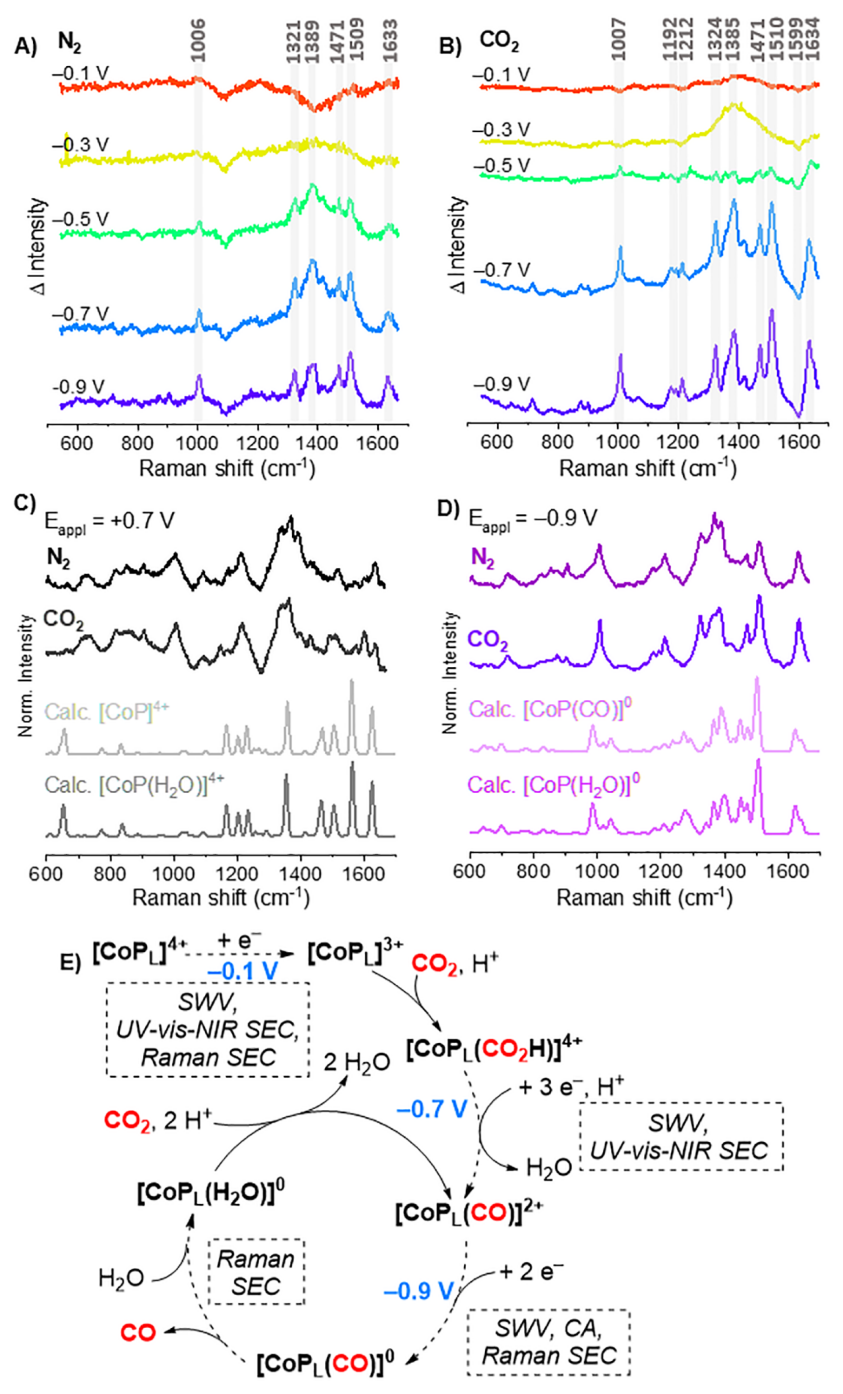
Resonance Raman spectroelectrochemistry (SEC), DFT calculations, and proposed catalytic cycle for **CoP_L_** on FTO. Potential-dependent in situ Raman SEC: (A) difference spectra for N_2_- and (B) CO_2_-saturated conditions. Gray translucent bars highlight prominent bands. (C and D) (upper) Experimental and (lower) DFT-calculated spectra at +0.7 and −0.9 V, respectively. All experimental spectra have a polynomial background removed. (E) Proposed catalytic mechanism for **CoP_L_** immobilized on FTO supported by experimental and computational results. SWV = square wave voltammetry, CA = chronoamperometry. The dashed box and arrows highlight the experimental techniques utilized to identify different intermediates at the steady state. N.B.: The small differences between plots A and B in the shape of the initial spectra at −0.1 V vs SHE are attributed to the different degrees of aggregation of **CoP_L_** molecules on the electrode surfaces after dropcasting.^44^

Raman SEC on FTO|**CoP_L_** was performed analogously to UV–vis SEC and interpreted as difference spectra obtained by subtracting the oxidized species spectrum (+0.7 V) from each spectrum (Figure 5A,B). Thereby, reduction of the porphyrin ring is observed below −0.3 V under N_2_ and CO_2_ consistent with SWV and UV–vis–NIR SEC results (Figures S42 and S43), specifically changes to peaks at 1007 and 1599 cm^–1^, ascribed to stretching and bending modes of pyrrole rings (C_α_–C_β_, C_β_–C_β_, and C_α_–N) and methine bridge (C_α_–C_*m*_) within the porphyrin core ligand,^45^ and between 1300 and 1500 cm^–1^ arising from CH_2_ twisting and CH_3_ bending modes from the alkyl tails (Table S16).^46^ Further reduction of the **CoP_L_** films from −0.5 to −0.7 V in N_2_ and CO_2_ induces the concomitant appearance of new and more intense bands (especially in the case of CO_2_) at 1192, 1212, and 1634 cm^–1^ that are ascribed to bending and stretching modes of C–C, C–N^+^, and N^+^–CH_2_ in the alkylated pyridinium rings.^45,47^ This corresponds to the increase in absorption at 860 nm attributed to reduced pyridinium rings. Importantly, applying −0.9 V induces no further spectral changes, highlighting that no more than two pyridinium rings of the **CoP_L_** molecules are reduced after −0.7 V, which is consistent with our SEC results and the literature.^47^ Furthermore, monitoring potential-dependent Raman intensities at 1599 and 1007 cm^–1^ for an oxidized and a reduced species, respectively, reproduces the trend observed in Figure 5B (Figure S44 and Table S17).

Having identified at least two species with distinct charge by Raman SEC, DFT calculations were carried out to obtain simulated Raman spectra for possible reaction intermediates. DFT calculations were performed using **CoP_W_** as a simplified structural model (**CoP** hereinafter) with various charges (+5 to −2) with and without several coadsorbed ligands (CO, CO_2_, COOH, H_2_O, and H). Results confirm that the cobalt oxidation state largely influences the Raman spectrum, whereas the axial ligands (other than CO_2_) result in minimal changes (Supplementary Note 7 and Figures S45–S48). Crucially, the DFT-calculated Raman spectra for the unreduced (**[CoP]^4+^** and **[CoP(H_2_O)]^4+^**) and four-electron reduced (**[CoP(CO)]^0^** and **[CoP(H_2_O)]^0^**) complexes reproduce the experimental spectra obtained under N_2_ and CO_2_ at +0.7 and −0.9 V, respectively (Figure 5C,D). This indicates that **CoP_L_** molecules can store up to four electrons, in agreement with SWV and UV–vis–NIR SEC. We hence propose a catalytic cycle for **CoP_L_** immobilized on FTO (Figure 5E). This catalytic mechanism proceeds via binding of CO_2_ and protonation by the singly reduced [**CoP_L_**]^3+^ (*E*_appl_ = −0.1 V vs SHE). Three further electron transfer steps, protonation, and dehydration of the CO_2_H adduct then form [**CoP_L_**(CO)]^2+^ (*E*_appl_ = −0.7 V). Subsequently, two-electron transfer steps (*E*_appl_ = −0.9 V) lead to the formation of the six-electron-reduced [**CoP_L_**(CO)]^0^ adduct. Desorption of CO and coordination of H_2_O form [**CoP_L_**(H_2_O)]^0^, which can react with CO_2_ and two protons to reform [**CoP_L_**(CO)]^2+^ and close the cycle.

## Conclusions

We report a systematic series of amphiphilic earth-abundant CO_2_ reduction catalysts, which were designed to readily self-assemble into lipid membranes and form, together with an amphiphilic ruthenium dye, photocatalytic liposomes. The most active liposome system containing the 5,10,15,20-(tetra-N-hexadecyl-4-pyridinium)porphyrin cobalt(II) catalyst **CoP_L_** is more active than its water-soluble analogue, achieving a high TON_CO_ (1456 after 4 h) with high CO selectivity (77%). The **CoP_L_** system thereby exceeds previously reported benchmarks in CO_2_ photoreduction in liposome and homogeneous systems, highlighting the beneficial effect on the activity and product selectivity when immobilizing molecular catalysts onto two-dimensional lipid bilayer surfaces.

Time-resolved and steady-state spectroscopies provided unprecedented insights into the origin of the higher activity of liposome-bound molecular systems. Results revealed that self-assembled dyes have a 4–100 times longer charge separation state lifetime, and display a ninefold faster electron transfer to self-assembled catalysts compared to homogeneous analogues. Two-dimensional charged membranes diminish diffusion limitations between ascorbate and self-assembled photosensitizers due to electrostatic attraction, and despite lowering φ_ET_, they increase the reduced photosensitizer lifetime. Membrane immobilization also leads to a higher relative surface concentration of membrane-bound species. This shortens the electron transfer distance between photosensitizers and catalysts, thereby resulting in enhanced catalytic activity. Furthermore, the superior catalytic activity of **CoP_L_** was examined to show that it undergoes a four-electron activation mechanism before catalytic turnover with key intermediates being determined by DFT calculations. The proposed multielectron activation mechanism further highlights the advantage of self-assembled systems as the electron transfer efficiency between dye–catalyst pairs is much higher than for diffusional systems. This effect is fundamental to the high activity of these photocatalytic liposome systems.

Hence, beyond providing new insights into the photoinduced charge-transfer dynamics of membrane-bound species and the catalytic mechanism of **CoP_L_**, this work illustrates the power of combining time-resolved and in situ spectroscopic techniques to understand molecule-based systems. This work shows the potential of liposome-bound molecular systems for efficient photocatalysis, which can move beyond CO_2_ reduction in future development.

## Experimental Section

### Materials

All synthetic procedures involving air- or moisture-sensitive materials were carried out under an inert N_2_ atmosphere by using Schlenk techniques. Solvents were purchased dried (e.g., DMF) or dried using standard purification procedures under an inert atmosphere. Reagents for synthesis were purchased from commercial suppliers in the highest purity available and used without further purification. CO_2_ and N_2_ gas bottles (2% methane internal standard) were purchased from BOC. NaHCO_3_ (99%), [Co(H_2_O)_6_](BF_4_)_2_ (>99%), (+)-sodium L-ascorbate (>99%), iodomethane (99%), sodium acetate (99%), tetrabutylammonium hexafluorophosphate (TBAPF_6_, >99%), 4,4′-dimethyl-2,2′-dipyridyl (98%), and n-butyllithium solution (2.5 M hexane) were purchased from Merck. [Ni(H_2_O)_6_](BF_4_)_2_ (>99%) was purchased from Fisher Scientific. 4’-Hydroxy-2,2′:6′,2″-terpyridine (98%) was purchased from HETCAT. Anhydrous FeCl_2_ (99%), 2,2′:6′,2″-terpyridine (97%), and 1-bromohexadecane (97%) were purchased from AK Scientific. [RuCl_2_(bpy)_2_] (with a minimum of 19% Ruthenium content) was purchased from Alfa Aesar. Sodium hexafluorophosphate (98.5%), Ni(acetate)_2_·4H_2_O (97%), and Co(acetate)_2_·4H_2_O (97%) were purchased from Acros Organics. 5,10,15,20-(Tetra-4-pyridyl)porphyrin (**P**, 98%), iron(III) 5,10,15,20-(tetra-*N*-methyl-4-pyridyl)porphyrin pentachloride (95%), and nickel(III) 5,10,15,20-(tetra-*N*-methyl-4-pyridyl)porphyrin pentachloride (95%) were purchased from Porphychem.

Lipid (dry powder) DMPC and polycarbonate extrusion filters (pore size = 0.2 μm; diameter = 19 mm) were purchased from Merck. Lipid (dry powder) NaDSPE-PEG2K, DLPC, DPPC and the extruder set, containing two needles with a holder and heating block, were purchased from Avanti.

### Physical Characterization

^1^H and ^13^C NMR spectra were collected with a Bruker 400 MHz NMR spectrometer at room temperature. Chemical shifts for ^1^H NMR spectra are referenced relative to residual protons in the deuterated solvent (Eurisotop). Elemental analyses were carried out by the Microanalysis Service of the Yusuf Hamied Department of Chemistry, University of Cambridge, using a Perkin-Elmer 240 Elemental Analyzer. High-resolution mass spectra were recorded using a Synapt G2-Si high-definition mass spectrometer. UV–vis spectra were collected using a Cary 60 UV–vis spectrometer. Attenuated total reflectance fourier-transform infrared spectra were recorded on a Nicolet iS50 spectrometer. Dynamic light scattering experiments were performed with a Zetasizer Nano ZS.

### Preparation of Liposomes and Synthesis of Catalysts and Photosensitizer

Full details of the followed methodology can be found in the Experimental Section in the Supporting Information.

### Characterization of Liposomes

Liposome samples were characterized via dynamic light scattering and by cryogenic transmission electron microscopy, which were used to confirm liposome size and analyze the fluidity of liposome samples containing molecular species.

#### Dynamic Light Scattering

The size distribution of the hydrodynamic diameter (*Z*_ave_) and the polydispersity index were measured at 25 °C by dynamic light scattering with a Zetasizer Nano-S from Malvern operating at 632.8 nm with a scattering angle of 173°.

#### Cryogenic Transmission Electron Microscopy (Cryo-TEM)

Samples were analyzed by Cryo-TEM as described elsewhere.^48^ In brief, samples were equilibrated at 25 °C and high relative humidity within a climate chamber. A small drop of each sample was deposited on a carbon-sputtered copper grid covered with a perforated polymer film. Excess liquid was thereafter removed by blotting with a filter paper, leaving a thin film of the solution on the grid. The sample was vitrified in liquid ethane and transferred to a microscope, continuously kept below −160 °C and protected against atmospheric conditions. Analyses were performed with a Zeiss Libra 120 transmission electron microscope (Carl Zeiss AG, Oberkochen, Germany) operating at 80 kV and in zero-loss bright-field mode. Digital images were recorded under low-dose conditions with a BioVision Pro-SM Slow Scan CCD camera (Proscan elektronische Systeme GmbH, Scheuring, Germany).

### Photocatalysis

Before photocatalytic testing, the liposome or homogeneous reaction solution (3 mL) was purged for 20 min with CO_2_, or N_2_ for control experiments, containing in both cases 2% methane as the internal standard for GC. After purging, the vials were kept in a water bath at 25 °C and irradiated for 4 h using a Newport Oriel Xenon 150 W solar light simulator (100 mW cm^–2^, AM1.5G) containing infrared water and ultraviolet (λ > 400 nm) filters. Each different photocatalytic experiment was performed in triplicate, unless otherwise stated. In the case of light intensity experiments, additional neutral density filters were used to achieve different light intensities (90, 50 and 20%).

### Gaseous Product Analysis

The amount of produced CO and H_2_ was analyzed by headspace gas analysis using a Shimadzu Tracera GC-2010 Plus with a barrier discharge ionization detector. The GC-2010 Plus was equipped with a ShinCarbon micro ST column (0.53 mm diameter) kept at 40 °C using helium carrier gas. Aliquots of 50 or 100 μL of the headspace gas were removed from the sealed photocatalytic vials using a gastight syringe (Hamilton) for GC analysis at hourly time intervals. Data are presented as mean ± standard error of the mean and were calculated from a number of repeats of independent experiments. No formate was detected using ^1^H NMR and ion chromatography. Photocatalytically generated methane was not detected, and this was confirmed by carrying out experiments with CO_2_ gas without any internal standard CH_4_. Then, after photocatalysis, the headspace gas was analyzed using GC.

### Isotopic Labeling Experiment

Photocatalysis experiments in 0.1 M NaH_2_PO_4_ and 0.1 M NaHAsc aqueous solution with ^13^CO_2_ as the headspace gas were performed. After 3 h of simulated light irradiation, the vial headspace was transferred to an evacuated gas infrared cell (SpecAc, 10-cm path length, equipped with KBr windows) and a high-resolution transmission spectrum was collected with a Thermo Scientific Nicolet iS50 FT-IR spectrometer.

### Quantum Yield Measurements

One-milliliter solutions containing DMPC (100 μM) liposomes made of **RuPS_L_** (10 μM) and **CoP_L_** (500 nM) were irradiated with monochromatic light (λ = 450 nm), using two different light intensities (*I*_1_ = 5.55 and *I*_2_ = 11.73 mW cm^–2^), produced with a solar simulator (LOT LSN 254) equipped with a monochromator (LOT MSH 300). Duplicate experiments were performed for each light intensity, and the averaged values of the produced μmol of CO were utilized to determine Φ_CO_ using eq 1:
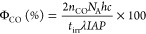
1where *n*_CO_ is the moles of photogenerated CO gas, *N*_A_ is the Avogadro constant in mol^–1^, *h* is the Planck constant in J s, *c* is the speed of light in m s^–1^, *t*_irr_ is the irradiation time in s, λ is the monochromatic light wavelength in m, *I* is the light intensity in J s^–1^ m^–2^, and *A* is the irradiation cross-section in m^2^. *P* is the probability of absorbing a photon by the photosensitizer, i.e., 1–10^–(Abs@454nm)^, where due to the high scattering of the DMPC liposomes the absorbance used was calculated employing the bulk concentration of **RuPS_L_** (10 μM) and its molar attenuation coefficient (1.35 × 10^4^ M^–1^ cm^–1^) in methanol.

### Steady-State Emission and Absorption Spectroscopy

Absorption spectra were recorded in 1.0 cm quartz cuvettes on a Cary 50 Bio spectrometer. Steady-state emission spectra were recorded in 1.0 cm quartz cuvettes on a Fluorolog 3 fluorimeter (Horiba) with double grating monochromators and a P928 PMT detector, and before measurements, all solutions were degassed with Ar.

### Determination of the **RuPS** Quenching Constants

For dynamic (diffusional) quenching, the Stern–Volmer equation (eq 2) was applied:^49^
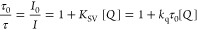
2where *I*_0_ and *I* are emission intensities in the absence and presence of the quencher, while τ_0_ and τ are emission lifetimes in the absence and presence of the quencher, respectively. *K*_SV_ is the Stern–Volmer constant, and *k*_q_ is the second-order rate constant for the quenching reaction.

For static quenching, where the emission intensity from the associated complex can be neglected, the following relation (eq 3) was used:^49^
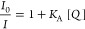
3where *K*_A_ is the association constant between the dye and quencher, and *I*_0_ and *I* have the same meaning as in the previous equation. In the case of purely static quenching, the observed lifetime of the unquenched dyes is not affected (τ_0_ = τ).

### Nanosecond Transient Absorption and Emission Measurements

For nanosecond transient absorption and emission measurements, optical excitation was performed by using the third harmonic output of a frequency-doubled Q-switched Nd:YAG laser combined with an OPO to generate 460 nm excitation pulses. For time-resolved spectra and kinetic traces on nano-to-microsecond time scales, a Quanta-Ray Pro series/OPO combination (Spectra-Physics) was used to give 460 nm, 8 mJ pulse^–1^ (in some cases, 20, 30, and 50 mJ pulse^–1^). The laser was coupled to a LP 920 detection system (Edinburgh Instruments) equipped with a pulsed XBO 450 W xenon Arc Lamp (Osram), which can provide the white light for probing. An iStar CCD camera (Andor Technology) and a LP920-K photomultiplier (PMT) detector connected to a Tektronix TDS 3052 500 MHz 5 GS/s oscilloscope were used for transient signal detection. Transient absorption and emission data were acquired using LP 900 software and processed using Origin 2018 software. For kinetic traces on milli-second time scales and above, a Quantel, Brilliant B laser with Opotek OPO was used to provide 460 nm, 15 mJ pulses. The probe light was single wavelength and provided using an un-pulsed 150 W Xe lamp in a flash photolysis spectrometer (Applied Photophysics LKS.60). Two monochromators were used to minimize sample excitation by probe light: the first monochromator was set to the desired detection wavelength before reaching the sample, and the second monochromator was placed after samples. The absorption difference of samples at specified wavelength can be monitored with a PMT Hamamatsu R928 detector and digitized using an Agilent Technologies Infinium digital oscilloscope (600 MHZ). Transient absorption data were acquired within the Applied Photophysics LKS software package. All transient absorption and emission measurements were carried out at room temperature, and a 1.0 cm path length quartz cell cuvette was used for the measurements, and before measurements, all solutions were degassed with Ar.

### Fabrication of GCE|Catalyst

Before immobilizing the alkylated catalysts, the GCE surface (diameter = 3.0 mm; area = 0.09 cm^2^) was cleaned by polishing using 0.015 μm alumina, rinsed with Milli-Q water, followed by sonication in Milli-Q water and acetone for 10 min each solvent, and dried with a N_2_ stream. The alkylated catalysts were immobilized onto the GC electrodes via dropcasting a known concentration of the catalysts in methanol (**MT_L_**) or acetone (**MP_L_**), followed by air drying. The concentration of catalysts on the GCE, calculated based on the dropcast volume and concentration of the initial solution, was 1.15 nmol cm^–2^.

### Fabrication of FTO|**CoP_L_**

Before immobilizing **CoP_L_**, the FTO electrodes were sonicated in acetone and isopropanol for 10 min each and then dried in air overnight. **CoP_L_** was immobilized onto FTO by dropcasting 0.4 mL of a 0.2 mM 1:1 acetone/hexane solution of the catalyst and drying in air.

### Electrochemistry

CV, SWV, and chronoamperometry measurements were conducted using an Ivium CompactStat potentiostat.

CV and SWV were used to characterize the catalysts in N_2_- or CO_2_-saturated 0.2 M TBAPF_6_ DMF homogeneous solutions at room temperature. A custom-made two compartment H-cell with frit separating the compartments with a three-electrode configuration was employed with airtight compartments. The glassy carbon and Pt mesh were used as working and counter electrodes, respectively, and Ag/AgNO_3_ (10 mM) was used as the reference electrode. All experiments in DMF are referenced against the ferrocene redox couple [*E*(Fc^0/+^) = +0.07 V vs Ag/AgNO_3_ (10 mM)].

Chronoamperometry measurements of GCE|catalysts and SWV and chronoamperometry measurements of FTO|**CoP_L_** were performed in a custom-made three-neck one-compartment cell. A three-electrode configuration was employed, using the GCE|catalyst or FTO|**CoP_L_** as the working electrode, Pt mesh as the counter electrode, and Ag/AgCl (KCl_sat_) as the reference electrode (BASi RE-6). The potentials were converted from Ag/AgCl (KCl_sat_) to the standard hydrogen electrode (SHE) by adding +0.199 V. All experiments carried out in aqueous conditions were reported against SHE. The electrolyte solution was 0.1 M NaHCO_3_ (aq.) (15 mL) and was purged with N_2_ or CO_2_ for 30 min to remove atmospheric O_2_. The pH of the N_2_- and CO_2_-saturated 0.1 M NaHCO_3_ was 8.0 and 6.7, respectively. All chronoamperometry experiments were performed for 4 h, and the applied potential was −1.1 V vs Ag/AgCl (KCl_sat_), i.e., −0.9 V vs SHE, without *iR* correction. All measurements were performed at room temperature as triplicate for each catalyst, and data are presented as mean ± standard error of the mean. The mean values and standard errors of the mean were calculated from the number of repeats of independent experiments.

### In Situ UV–vis–NIR Spectroelectrochemistry

Measurements were conducted in a single-compartment airtight electrochemical cell using N_2_- or CO_2_-saturated 0.1 M NaHCO_3_, and a three-electrode configuration was employed. FTO|**CoP_L_** was used as the working electrode, Pt mesh as the counter electrode, and Ag/AgCl (KCl_sat_) as the reference electrode (BASi RE-6). For stepwise chronoamperometry (+0.7 V to −0.9 V vs SHE), the working electrode was kept at each potential for 1 min and the UV–vis–NIR spectra were recorded on an Agilent Cary 60 spectrophotometer using Cary WinUV scanning software. Applied potentials were +0.7, +0.2, +0.1, 0.0, −0.1, −0.2, −0.3, −0.4, −0.5, −0.6, −0.7, −0.8, and −0.9 V vs SHE. Using different electrodes, as the final step, after the stepwise reduction of the film, the potential was switched back to +0.7 V to reoxidize the film. Normalized absorbance values were calculated using eq 4:
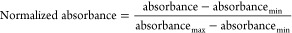
4

### In Situ Resonance Raman Spectroelectrochemistry

Raman spectra were obtained using a Renishaw inVia spectrometer. Excitation at 785 nm and collection were via a 20× 0.45 NA objective. Typical laser power was 0.4 mW with 60 s exposure time. SEC experiments were performed using an Autolab PGSTAT204 in a custom-built 3D printed cell using FTO|**CoP_L_** as the working electrode, leakless Ag/AgCl as the reference electrode (Green Leaf Scientific), and Pt mesh as the counter electrode. During chronoamperometry, 1 min was allowed at each applied potential step (i.e., +0.7, −0.1, −0.3, −0.5, −0.7, and −0.9 V vs SHE), before spectra were recorded to allow the cell to equilibrate. Spectral analysis was performed with a custom python script. Approximately 10 spectra were recorded per potential on different sample areas, with averaged spectra used for further analysis. Spectra were background-subtracted using a 4^th^ order polynomial estimation method. Difference spectra were calculated from the difference of each spectrum with the first, recorded at +0.7 V vs SHE, using both raw and background-subtracted spectra to ensure that no processing artifacts are introduced by background subtraction. Relative intensity versus potential was calculated as follows. First, characteristic modes for the oxidized and reduced species were selected and confirmed via comparison to DFT calculations. Next, the mode area at each potential, (*V*), is obtained by integrating spectral intensity. Relative intensity is then calculated using eq 5:
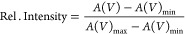
5

### Computational Details

DFT calculations were performed with Gaussian09 (revision D1).^50^ Geometry optimization, vibrational analysis, and Raman activities were calculated with a 6–31 + G*^51,52^ basis set for C, H, O, and N and the Stuttgart/Dresden effective core potential (SDD)^53,54^ for Co, Ni, Fe, and Ru. All the calculations were performed using the uB3LYP^55^ functional including Grimmes D3 dispersion correction.^56^ Single-point energy calculations were performed with a 6-311++G(3df,3pd)^57,58^ basis set for C, H, O, and N and the SDD for Co, Ni, Fe, and Ru. Free energies were calculated from single-point energy calculations and free energy corrections obtained from geometry optimization and vibrational frequency calculation, and a correction to a 1 M standard was applied (1.9 kcal mol^–1^). Solvent effects for the geometry optimization and single-point calculations were modeled with a PCM solvation model with the dielectric constant of H_2_O (78.4).^59^ Various spin states of the intermediates were calculated, and the most stable one was chosen. Electron transfer energies were referenced by the calculated [Ru(bipy)_3_]^2+^/[Ru(bipy)_3_]^1+^ redox cycle, and proton transfer energies were calculated from the free energy of a free proton in H_2_O (−272.2 kcal mol^–1^).^60,61^

Theoretical Raman spectra were simulated based on the calculated Raman activities for a corresponding frequency according to eq 6:
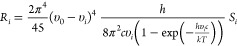
6where *υ_i_* is the individually *i* calculated frequency, *υ*_0_ is the frequency of the probing light (12738.85 cm^–1^), *h* is the Plank constant (6.626·10^–34^ J s), *c* is the speed of light (3.00·10^–8^ m s^–1^), *k* is the Boltzmann constant (1.38·10^–23^ J K^–1^), *T* is the temperature (298.15 K), and *S_i_* is the DFT-calculated Raman activity for each individually *i* calculated frequency. A correction factor of 0.96 for the calculated frequencies was applied. For the simulated spectra, a gaussian broadening with a variance of 40 cm^–1^ was applied to each frequency and all the individual gaussian curves were summed up to obtain the final simulated Raman spectra.

## Supporting Information Available

Additional experimental details for the synthesis of the described ligands and metal complexes (including NMR, MS, UV–vis, and IR data), preparation of liposomes, electrochemistry data of all catalysts in organic media and on electrodes, cryogenic transmission electron microscopy of liposomes, photocatalysis results, photoinduced charge-transfer dynamic investigations, UV–vis–NIR and resonance Raman spectroelectrochemistry results for **CoP_L_** on FTO, and DFT-calculated Raman spectra, and computed structures (PDF)
XYZ atomic coordinates (ZIP)

## References

[ref1] Pannwitz A.; Klein D. M.; Rodríguez-Jiménez S.; Casadevall C.; Song H.; Reisner E.; Hammarström L.; Bonnet S. Roadmap towards solar fuel synthesis at the water interface of liposome membranes. Chem. Soc. Rev. 2021, 50, 4833–4855. 10.1039/D0CS00737D. 33659967

[ref2] Takayanagi T.; Nagamura T.; Matsuo T. Photoinduced Electron Transfer between Amphipathic Ruthenium(II) Complex and N-Butylphenothiazine in Various Microenvironments. Ber. Bunsen. Phys. Chem. 1980, 84, 1125–1129. 10.1002/bbpc.19800841109.

[ref3] Infelta P. P.; Graetzel M.; Fendler J. H. Aspects of artificial photosynthesis. Photosensitized electron transfer and charge separation in cationic surfactant vesicles. J. Am. Chem. Soc. 1980, 102, 1479–1483. 10.1021/ja00525a001.

[ref4] Hammarström L.; Norrby T.; Stenhagen G.; Mårtensson J.; Åkermark B.; Almgren M. Two-Dimensional Emission Quenching and Charge Separation Using a Ru(II)-Photosensitizer Assembled with Membrane-Bound Acceptors. J. Phys. Chem. B. 1997, 101, 7494–7504. 10.1021/jp9710805.

[ref5] Stikane A.; Hwang E. T.; Ainsworth E. V.; Piper S. E. H.; Critchley K.; Butt J. N.; Reisner E.; Jeuken L. J. C. Towards compartmentalized photocatalysis: multihaem proteins as transmembrane molecular electron conduits. Faraday Discuss. 2019, 215, 26–38. 10.1039/C8FD00163D. 30969289

[ref6] Hu H.; Wang Z.; Cao L.; Zeng L.; Zhang C.; Lin W.; Wang C. Metal–organic frameworks embedded in a liposome facilitate overall photocatalytic water splitting. Nat. Chem. 2021, 13, 358–366. 10.1038/s41557-020-00635-5. 33589788

[ref7] Grimaldi J. J.; Boileau S.; Lehn J.-M. Light-driven, carrier-mediated electron transfer across artificial membranes. Nature 1977, 265, 229–230. 10.1038/265229a0. 834265

[ref8] Steinberg-Yfrach G.; Rigaud J.-L.; Durantini E. N.; Moore A. L.; Gust D.; Moore T. A. Light-driven production of ATP catalysed by F0F1-ATP synthase in an artificial photosynthetic membrane. Nature 1998, 392, 479–482. 10.1038/33116. 9548252

[ref9] Limburg B.; Bouwman E.; Bonnet S. Catalytic photoinduced electron transport across a lipid bilayer mediated by a membrane-soluble electron relay. Chem. Commun. 2015, 51, 17128–17131. 10.1039/C5CC07745A. 26456173

[ref10] Schenning A.; Lutje Spelberg J.; Driessen M.; Hauser M.; Feiters M.; Nolte R. Enzyme Mimic Displaying Oscillatory Behavior. Oscillating Reduction of Manganese(III) Porphyrin in a Membrane-Bound Cytochrome P-450 Model System. J. Am. Chem. Soc. 1995, 117, 12655–12656. 10.1021/ja00155a600.

[ref11] Calvin M. Simulating photosynthetic quantum conversion. Acc. Chem. Res. 1978, 11, 369–374. 10.1021/ar50130a001.

[ref12] Steinberg-Yfrach G.; Liddell P. A.; Hung S.-C.; Moore A. L.; Gust D.; Moore T. A. Conversion of light energy to proton potential in liposomes by artificial photosynthetic reaction centres. Nature 1997, 385, 239–241. 10.1038/385239a0.

[ref13] Bhosale S.; Sisson A. L.; Talukdar P.; Fürstenberg A.; Banerji N.; Vauthey E.; Bollot G.; Mareda J.; Röger C.; Würthner F.; Sakai N.; Matile S. Photoproduction of proton gradients with pi-stacked fluorophore scaffolds in lipid bilayers. Science 2006, 313, 84–86. 10.1126/science.1126524. 16825567

[ref14] Hansen M.; Li F.; Sun L.; König B. Photocatalytic Water Oxidation at Soft Interfaces. Chem. Sci. 2014, 5, 2683–2687. 10.1039/C4SC01018C.

[ref15] Limburg B.; Wermink J.; van Nielen S. S.; Kortlever R.; Koper M. T. M.; Bouwman E.; Bonnet S. Kinetics of Photocatalytic Water Oxidation at Liposomes: Membrane Anchoring Stabilizes the Photosensitizer. ACS Catal. 2016, 6, 5968–5977. 10.1021/acscatal.6b00151.

[ref16] Troppmann S.; König B. Functionalized Membranes for Photocatalytic Hydrogen Production. Chem. – Eur. J. 2014, 20, 14570–14574. 10.1002/chem.201404480. 25283542

[ref17] Troppmann S.; Brandes E.; Motschmann H.; Li F.; Wang M.; Sun L.; König B. Enhanced Photocatalytic Hydrogen Production by Adsorption of an [FeFe]-Hydrogenase Subunit Mimic on Self-Assembled Membranes. Eur. J. Inorg. Chem. 2016, 2016, 554–560. 10.1002/ejic.201501377.

[ref18] Ikuta N.; Takizawa S.-Y.; Murata S. Photochemical reduction of CO_2_ with ascorbate in aqueous solution using vesicles acting as photocatalysts. Photochem. Photobiol. Sci. 2014, 13, 691–702. 10.1039/C3PP50429H. 24549095

[ref19] Klein D. M.; Rodríguez-Jiménez S.; Hoefnagel M. E.; Pannwitz A.; Prabhakaran A.; Siegler M. A.; Keyes T. E.; Reisner E.; Brouwer A. M.; Bonnet S. Shorter Alkyl Chains Enhance Molecular Diffusion and Electron Transfer Kinetics Between Photosensitisers and Catalysts in CO_2_-Reducing Photocatalytic Liposomes. Chem. – Eur. J. 2021, 27, 17203 10.1002/chem.202102989. 34726811 PMC9299206

[ref20] Kuehnel M. F.; Orchard K. L.; Dalle K. E.; Reisner E. Selective photocatalytic CO_2_ reduction in water through anchoring of a molecular Ni catalyst on CdS nanocrystals. J. Am. Chem. Soc. 2017, 139, 7217–7223. 10.1021/jacs.7b00369. 28467076

[ref21] Zhang X.; Cibian M.; Call A.; Yamauchi K.; Sakai K. Photochemical CO_2_ Reduction Driven by Water-Soluble Copper(I) Photosensitizer with the Catalysis Accelerated by Multi-Electron Chargeable Cobalt Porphyrin. ACS Catal. 2019, 9, 11263–11273. 10.1021/acscatal.9b04023.

[ref22] Call A.; Cibian M.; Yamamoto K.; Nakazono T.; Yamauchi K.; Sakai K. Highly Efficient and Selective Photocatalytic CO_2_ Reduction to CO in Water by a Cobalt Porphyrin Molecular Catalyst. ACS Catal. 2019, 9, 4867–4874. 10.1021/acscatal.8b04975.

[ref23] Wang Q.; Warnan J.; Rodríguez-Jiménez S.; Leung J. J.; Kalathil S.; Andrei V.; Domen K.; Reisner E. Molecularly engineered photocatalyst sheet for scalable solar formate production from carbon dioxide and water. Nat. Energy 2020, 5, 703–710. 10.1038/s41560-020-0678-6.

[ref24] Zhang X.; Yamauchi K.; Sakai K. Earth-Abundant Photocatalytic CO_2_ Reduction by Multielectron Chargeable Cobalt Porphyrin Catalysts: High CO/H_2_ Selectivity in Water Based on Phase Mismatch in Frontier MO Association. ACS Catal. 2021, 10436–10449. 10.1021/acscatal.1c02475.

[ref25] Arcudi F.; Đorđević L.; Nagasing B.; Stupp S. I.; Weiss E. A. Quantum Dot-Sensitized Photoreduction of CO_2_ in Water with Turnover Number > 80,000. J. Am. Chem. Soc. 2021, 143, 18131–18138. 10.1021/jacs.1c06961. 34664969

[ref26] Mondal B.; Rana A.; Sen P.; Dey A. Intermediates Involved in the 2e^–^/2H^+^ Reduction of CO_2_ to CO by Iron(0) Porphyrin. J. Am. Chem. Soc. 2015, 137, 11214–11217. 10.1021/jacs.5b05992. 26313628

[ref27] Reuillard B.; Ly K. H.; Rosser T. E.; Kuehnel M. F.; Zebger I.; Reisner E. Tuning Product Selectivity for Aqueous CO_2_ Reduction with a Mn(bipyridine)-pyrene Catalyst Immobilized on a Carbon Nanotube Electrode. J. Am. Chem. Soc. 2017, 139, 14425–14435. 10.1021/jacs.7b06269. 28885841 PMC5649446

[ref28] Leung J. J.; Warnan J.; Ly K. H.; Heidary N.; Nam D. H.; Kuehnel M. F.; Reisner E. Solar-driven reduction of aqueous CO_2_ with a cobalt bis(terpyridine)-based photocathode. Nat. Catal. 2019, 2, 354–365. 10.1038/s41929-019-0254-2.

[ref29] Guo Z.; Chen G.; Cometto C.; Ma B.; Zhao H.; Groizard T.; Chen L.; Fan H.; Man W.-L.; Yiu S.-M.; Lau K.-C.; Lau T.-C.; Robert M. Selectivity control of CO versus HCOO^–^ production in the visible-light-driven catalytic reduction of CO_2_ with two cooperative metal sites. Nat. Catal. 2019, 2, 801–808. 10.1038/s41929-019-0331-6.

[ref30] Fernández S.; Franco F.; Casadevall C.; Martin-Diaconescu V.; Luis J. M.; Lloret-Fillol J. A Unified Electro- and Photocatalytic CO_2_ to CO Reduction Mechanism with Aminopyridine Cobalt Complexes. J. Am. Chem. Soc. 2020, 142, 120–133. 10.1021/jacs.9b06633. 31820956

[ref31] Lu X.; Ahsaine H. A.; Dereli B.; Garcia-Esparza A. T.; Reinhard M.; Shinagawa T.; Li D.; Adil K.; Tchalala M. R.; Kroll T.; Eddaoudi M.; Sokaras D.; Cavallo L.; Takanabe K. Operando Elucidation on the Working State of Immobilized Fluorinated Iron Porphyrin for Selective Aqueous Electroreduction of CO_2_ to CO. ACS Catal. 2021, 11, 6499–6509. 10.1021/acscatal.1c01157.

[ref32] Amanullah S.; Saha P.; Dey A. Activating the Fe(I) State of Iron Porphyrinoid with Second-Sphere Proton Transfer Residues for Selective Reduction of CO_2_ to HCOOH via Fe(III/II)–COOH Intermediate(s). J. Am. Chem. Soc. 2021, 143, 13579–13592. 10.1021/jacs.1c04392. 34410125

[ref33] Elgrishi N.; Chambers M. B.; Artero V.; Fontecave M. Terpyridine complexes of first row transition metals and electrochemical reduction of CO_2_ to CO. Phys. Chem. Chem. Phys. 2014, 16, 13635–13644. 10.1039/C4CP00451E. 24651983

[ref34] Araullo-McAdams C.; Kadish K. M. Electrochemistry, spectroscopy, and reactivity of (meso-tetrakis(1-methylpyridinium-4-yl)porphinato)cobalt(III,II,I) in nonaqueous media. Inorg. Chem. 1990, 29, 2749–2757. 10.1021/ic00340a009.

[ref35] Hansen M.; Troppmann S.; König B. Artificial Photosynthesis at Dynamic Self-Assembled Interfaces in Water. Chem. – Eur. J. 2016, 22, 58–72. 10.1002/chem.201503712. 26552728

[ref36] Edwardes Moore E.; Cobb S. J.; Coito A. M.; Oliveira A. R.; Pereira I. A. C.; Reisner E. Understanding the local chemical environment of bioelectrocatalysis. Proc. Natl. Acad. Sci. U. S. A. 2022, 119, e2114097119 10.1073/pnas.2114097119. 35058361 PMC8795565

[ref37] Hawecker J.; Lehn J.-M.; Ziessel R. Photochemical reduction of carbon dioxide to formate mediated by ruthenium bipyridine complexes as homogeneous catalysts. J. Chem. Soc., Chem. Commun. 1985, 56–58. 10.1039/c39850000056.

[ref38] Grant J. L.; Goswami K.; Spreer L. O.; Otvos J. W.; Calvin M. Photochemical reduction of carbon dioxide to carbon monoxide in water using a nickel(II) tetra-azamacrocycle complex as catalyst. J. Chem. Soc., Dalton Trans. 1987, 2105–2109. 10.1039/dt9870002105.

[ref39] Nakada A.; Koike K.; Nakashima T.; Morimoto T.; Ishitani O. Photocatalytic CO_2_ Reduction to Formic Acid Using a Ru(II)–Re(I) Supramolecular Complex in an Aqueous Solution. Inorg. Chem. 2015, 54, 1800–1807. 10.1021/ic502707t. 25654586

[ref40] Arias-Rotondo D. M.; McCusker J. K. The photophysics of photoredox catalysis: a roadmap for catalyst design. Chem. Soc. Rev. 2016, 45, 5803–5820. 10.1039/C6CS00526H. 27711624

[ref41] Lomoth R.; Häupl T.; Johansson O.; Hammarström L. Redox-Switchable Direction of Photoinduced Electron Transfer in an Ru(bpy)_3_^2+^–Viologen Dyad. Chem. – Eur. J. 2002, 8, 102–110. 10.1002/1521-3765(20020104)8:1<102::AID-CHEM102>3.0.CO;2-S. 11822443

[ref42] Hu X.-M.; Rønne M. H.; Pedersen S. U.; Skrydstrup T.; Daasbjerg K. Enhanced Catalytic Activity of Cobalt Porphyrin in CO_2_ Electroreduction upon Immobilization on Carbon Materials. Angew. Chem., Int. Ed. 2017, 56, 6468–6472. 10.1002/anie.201701104. 28466962

[ref43] Ogawa M.; Ajayakumar G.; Masaoka S.; Kraatz H.-B.; Sakai K. Platinum(II)-Based Hydrogen-Evolving Catalysts Linked to Multipendant Viologen Acceptors: Experimental and DFT Indications for Bimolecular Pathways. Chem. – Eur. J. 2011, 17, 1148–1162. 10.1002/chem.201002470. 21243681

[ref44] Götz R.; Ly H. K.; Wrzolek P.; Schwalbe M.; Weidinger I. M. Surface enhanced resonance Raman spectroscopy of iron Hangman complexes on electrodes during electrocatalytic oxygen reduction: advantages and problems of common drycast methods. Dalton Trans. 2017, 46, 13220–13228. 10.1039/C7DT01174A. 28682383

[ref45] Terekhov S. N.; Kruglik S. G.; Malinovskii V. L.; Galievsky V. A.; Chirvony V. S.; Turpin P.-Y. Resonance Raman characterization of cationic Co(II) and Co(III) tetrakis(N-methyl-4-pyridinyl)porphyrins in aqueous and non-aqueous media. J. Raman. Spectrosc. 2003, 34, 868–881. 10.1002/jrs.1068.

[ref46] Orendorff C. J.; Ducey M. W. Jr.; Pemberton J. E. Quantitative Correlation of Raman Spectral Indicators in Determining Conformational Order in Alkyl Chains. J. Phys. Chem. A 2002, 106, 6991–6998. 10.1021/jp014311n. 31307137

[ref47] Liu B.; Blaszczyk A.; Mayor M.; Wandlowski T. Redox-Switching in a Viologen-type Adlayer: An Electrochemical Shell-Isolated Nanoparticle Enhanced Raman Spectroscopy Study on Au(111)-(1×1) Single Crystal Electrodes. ACS Nano 2011, 5, 5662–5672. 10.1021/nn201307g. 21634391

[ref48] Almgren M.; Edwards K.; Karlsson G. Cryo transmission electron microscopy of liposomes and related structures. Colloids Surf. A Physicochem. Eng. Asp. 2000, 174, 3–21. 10.1016/S0927-7757(00)00516-1.

[ref49] Lakowicz J. R. Principles of fluorescence spectroscopy; Springer: Boston, MA, 2006.

[ref50] Frisch M. J., Trucks G. W., Schlegel H. B., Scuseria G. E., Robb M. A., Cheeseman J. R., Scalmani G., Barone V., Mennucci B., Petersson G. A., Nakatsuji H., Caricato M., Li X., Hratchian H. P., Izmaylov A. F., Bloino J., Zheng G., Sonnenberg J. L., Hada M., Ehara M., Toyota K., Fukuda R., Hasegawa J., Ishida M., Nakajima T., Honda Y., Kitao O., Nakai H., Vreven T., Montgomery J. J. A., Peralta J. E., Ogliaro F., Bearpark M., Heyd J. J., Brothers E., Kudin K. N., Staroverov V. N., Kobayashi R., Normand J., Raghavachari K., Rendell A., Burant J. C., Iyengar S. S., Tomasi J., Cossi M., Rega N., Millam J. M., Klene M., Knox J. E., Cross J. B., Bakken V., Adamo C., Jaramillo J., Gomperts R., Stratmann R. E., Yazyev O., Austin A. J., Cammi R., Pomelli C., Ochterski J. W., Martin R. L., Morokuma K., Zakrzewski V. G., Voth G. A., Salvador P., Dannenberg J. J., Dapprich S., Daniels A. D., Farkas Ö., Fore J. B., Cioslowski J.; Fox D. J. Gaussian 09, Revision D.01; Gaussian, Inc.: Wallingford, CT, 2009.

[ref51] Rassolov V. A.; Ratner M. A.; Pople J. A.; Redfern P. C.; Curtiss L. A. 6-31G* basis set for third-row atoms. J. Comput. Chem. 2001, 22, 976–984. 10.1002/jcc.1058.

[ref52] Francl M. M.; Pietro W. J.; Hehre W. J.; Binkley J. S.; Gordon M. S.; DeFrees D. J.; Pople J. A. Self-consistent molecular orbital methods. XXIII. A polarization-type basis set for second-row elements. J. Chem. Phys. 1982, 77, 3654–3665. 10.1063/1.444267.

[ref53] Dolg M.; Wedig U.; Stoll H.; Preuss H. Energy-adjusted ab initio pseudopotentials for the first row transition elements. J. Chem. Phys. 1987, 86, 866–872. 10.1063/1.452288.

[ref54] Andrae D.; Häußermann U.; Dolg M.; Stoll H.; Preuß H. Energy-adjusted ab initio pseudopotentials for the second and third row transition elements. Theor. Chim. Acta 1990, 77, 123–141. 10.1007/BF01114537.

[ref55] Becke A. D. Density-functional thermochemistry. III. The role of exact exchange. J. Chem. Phys. 1993, 98, 5648–5652. 10.1063/1.464913.

[ref56] Grimme S.; Antony J.; Ehrlich S.; Krieg H. A consistent and accurate ab initio parametrization of density functional dispersion correction (DFT-D) for the 94 elements H-Pu. J. Chem. Phys. 2010, 132, 154104 10.1063/1.3382344. 20423165

[ref57] Krishnan R.; Binkley J. S.; Seeger R.; Pople J. A. Self-consistent molecular orbital methods. XX. A basis set for correlated wave functions. J. Chem. Phys. 1980, 72, 650–654. 10.1063/1.438955.

[ref58] McLean A. D.; Chandler G. S. Contracted Gaussian basis sets for molecular calculations. I. Second row atoms, Z=11–18. J. Chem. Phys. 1980, 72, 5639–5648. 10.1063/1.438980.

[ref59] Miertuš S.; Scrocco E.; Tomasi J. Electrostatic interaction of a solute with a continuum. A direct utilization of AB initio molecular potentials for the prevision of solvent effects. Chem. Phys. 1981, 55, 117–129. 10.1016/0301-0104(81)85090-2.

[ref60] Kelly C. P.; Cramer C. J.; Truhlar D. G. Aqueous Solvation Free Energies of Ions and Ion–Water Clusters Based on an Accurate Value for the Absolute Aqueous Solvation Free Energy of the Proton. J. Phys. Chem. B. 2006, 110, 16066–16081. 10.1021/jp063552y. 16898764

[ref61] Casasnovas R.; Ortega-Castro J.; Frau J.; Donoso J.; Muñoz F. Theoretical pKa calculations with continuum model solvents, alternative protocols to thermodynamic cycles. Int. J. Quantum Chem. 2014, 114, 1350–1363. 10.1002/qua.24699.

