# Supplementary material for: Self-Assembled Liposomes Enhance Electron Transfer for Efficient Photocatalytic CO2 Reduction
Source: J Am Chem Soc. 2022 May 20;144(21):9399–412. doi: 10.1021/jacs.2c01725 (PMC9164230; doi:10.1021/jacs.2c01725)
Supplement: Supplementary file 1 — ja2c01725_si_001.pdf [file ja2c01725_si_001.pdf]

Supporting information

Self-Assembled Liposomes Enhance Electron Transfer for Efficient Photocatalytic CO_2 Reduction

Santiago Rodríguez-Jiménez,[1#] Hongwei Song,[2#] Erwin Lam,[1] Demelza Wright,[3] Andrea Pannwitz,[4,5] Shannon A. Bonke,[1] Jeremy J. Baumberg,[3] Sylvestre Bonnet,[4] Leif Hammarström,[2] Erwin Reisner[1]**

[1] Yusuf Hamied Department of Chemistry, University of Cambridge, Lensfield Road, Cambridge CB2 1EW, UK.

[2] Department of Chemistry – Angstrom Laboratory, Uppsala University, Box 523, 751 20 Uppsala, Sweden.

[3] Nanophotonics Centre, Department of Physics, Cavendish Laboratory, University of Cambridge, Cambridge CB3 0HE, UK.

[4] Leiden Institute of Chemistry, Leiden University, Einsteinweg 55, 2333 CC, Leiden, The Netherlands.

[5] Institute of Inorganic Chemistry I, Ulm University, Albert-Einstein-Allee 11, 89081 Ulm, Germany.

[#]Equal contribution.

<u>Corresponding author email address:</u>

Erwin Reisner: reisner@ch.cam.ac.uk

Leif Hammarström: leif.hammarstrom@kemi.uu.se

Table of Contents

Synthetic methods 3
Synthesis of terpyridine-based ligand and catalysts 3
Synthesis of porphyrin-based ligand and catalysts 5
Synthesis of alkylated bipyridine ligand and ruthenium photosensitizer 8
Sample preparation 10
Supplementary notes 11
Supplementary tables 16
Summary of electrochemical data of all catalysts in organic media 16
Summary of dynamic light scattering results 19
Tabulated photocatalysis results 21
Tabulated information from photoinduced charge-transfer dynamics investigations 27
Electrochemistry results of electrode-immobilized catalysts in aqueous conditions 28
Summary of experimental and calculated resonance Raman data 29
Tabulated Gibbs free energy of all calculated species 31
Supplementary Schemes 37
Preparation of liposomes 37
Synthesis of ligands, catalysts and photosensitizer 37
Summary of atom labeling for Raman assignments of the cobalt porphyrin 39
Supplementary Figures 40
NMR spectroscopy of prepared ligands and $RuPS_L$ 40
Absorption spectroscopy of molecular species 44
Electrochemistry measurements of molecular catalysts in DMF 47
Cryogenic transmission electron microscopy 53
Summary of photocatalytic performance of alkylated catalysts in liposomes 54
Isotopic labeling control experiment 58
UV-vis spectra of before and after light irradiation of molecule containing DMPC liposomes 59
Reductive quenching studies 60
Self-quenching studies 62
Charge separation quantum yields in homogeneous and liposome systems 63
Monitoring the generation of $RuPS^{-}$ in homogeneous and charge separation lifetimes in liposomes 64
Kinetic studies between $RuPS^{-}$ and catalysts in homogeneous and liposomes 65
Square wave voltammetry and chronoamperometry 71
UV-vis-NIR spectroelectrochemistry 72
Resonance Raman spectroelectrochemistry 73
DFT calculated Raman spectra 75
Calculated structures of proposed reaction intermediates using model catalyst CoP 79
References 83

Synthetic methods

Synthesis of terpyridine-based ligand and catalysts

Synthesis of T_L. This synthesis protocol was adapted from ref.[1] To a stirring dry dimethylformamide solution (12 mL) of 4'-hydroxy-2,2':6',2''-terpyridine (**T_{OH}**, 329 mg, 1.32 mmol) under N_2 atmosphere, 1-bromohexadecane (0.45 mL, 1.47 mmol) was added. After 5 minutes of stirring, K_2CO_3 (372 mg, 2.69 mmol) was added under N_2 to the brown solution and the resulting suspension was heated at 80 °C for 8 h under a N_2 atmosphere. Then after cooling the suspension to room temperature, $CHCl_3$ (20 mL) was added and was washed with water (10 mL) and the organic phase was dried over $MgSO_4$ and taken to dryness. The resulting yellowish solid was recrystallized in acetonitrile and upon cooling a fluffy solid formed, which was filtered and washed with water and dried in vacuo to give a fluffy off-white crystalline solid (490 mg, 77 %). **EA results:** Calcd. for $C_{31}H_{43}N_3O$ (M = 473.34 g·mol^{-1}): C 78.60, H 9.15, N 8.87 %. Found: C 78.28, H 9.12, N 8.83 %. **^{1}H NMR ($CDCl_3$, 400 MHz):** δ (ppm) = 8.70-8.68 (ddd, 2H_a), 8.63-8.61 (dd, 2H_d), 8.01 (s, 2H_e), 7.87-7.82 (td, 2H_c), 7.34-7.31 (ddd, 2H_b), 4.24-4.21 (t, 2H_f), 1.89-1.82 (q, 2H_g), 1.53-1.46 (q, 2 H_h), 1.39-1.34 (m, 2H_i), 1.32-1.22 (m, 22 H_j), 0.89-0.86 (t, 3 H_k). **^{13}C NMR ($CDCl_3$, 100 MHz):** δ (ppm) = 167.54, 157.18, 156.40, 149.16, 136.89, 123.88, 121.49, 107.58, 68.39, 32.07, 29.84, 29.74, 29.72, 29.50, 29.47, 29.20, 26.11, 22.83, 14.25. **ESI-MS (+, methanol):** m/z calcd. for $C_{31}H_{44}N_3O^+$ (i.e. MH^+) 474.3484, found 474.3483. **UV-vis ($CHCl_3$):** λ_{max} (nm) = 280. **ATR-FTIR:** ν (cm^{-1}) = 2943, 2915, 2871, 2847, 1581, 1563, 1469, 1443, 1402, 1359, 1200, 1026, 796, 720.

Synthesis of CoT_L. This synthesis protocol was adapted from ref.[2] 4'-Hexadecyloxy-2,2':6',2''-terpyridine (**T_L**, 100 mg, 212 μmol) and $[Co^{II}(H_2O)_6](BF_4)_2$ (35 mg, 103 μmol) were stirred for two hours in 1:1 methanol:$CHCl_3$ (5 mL) at room temperature and under N_2 atmosphere to give a dark red solution. Subsequently, the solution was evaporated to dryness, and the solid was suspended in hexane (2 mL), sonicated for 15 min, then it was filtered off and washed with hexane and dried under vacuum to yield a deep red powder (80 mg, 75 %). **EA results:** Calcd. for $C_{62}H_{86}N_6O_2B_2F_8Co$ (M = 1179.95 g·mol^{-1}): C 63.11, H 7.35, N 7.12 %.

Found: C 62.82, H 7.52, N 7.14 %. **ESI-MS (+, methanol):** m/z calcd. for $C_{62}H_{86}N_6O_2Co^{2+}$ (i.e. M^{2+}) 502.8067, found 502.8051; $C_{62}H_{86}N_6O_2BF_4Co^+$ (i.e. $[M^{2+} + BF_4^-]^+$) 1092.6173, found 1092.6053. **UV-vis (methanol):** λ_{max} (nm) (ε, $M^{-1}\cdot cm^{-1}$) = 308 (20.3 x 10^3), 360 (2.0 x 10^3), 450 (7.3 x 10^2), 500 (5.4 x 10^2). **ATR-FTIR:** ν (cm^{-1}) = 3093, 2922, 2849, 1616, 1557, 1470, 1441, 1366, 1223, 1030, 793.

Synthesis of NiT_L. This catalyst was synthesized analogously to **CoT_L**,[2] but replacing $[Co^{II}(H_2O)_6](BF_4)_2$ with $[Ni^{II}(H_2O)_6](BF_4)_2$. Pale brown-pink crystalline powder (101 mg, 41 %). **EA results:** Calcd. for $C_{62}H_{86}N_6O_2B_2F_8Ni$ (*M* = 1183.74 $g\cdot mol^{-1}$): C 62.91, H 7.66, N 7.10 %. Found: C 62.98, H 7.38, N 7.17 %. **ESI-MS (+, methanol):** m/z calcd. for $C_{62}H_{86}N_6O_2BF_4Ni^+$ (i.e. $[M^{2+} - BF_4^-]^+$) 1091.6195, found 1091.6246. **UV-vis (methanol):** λ_{max} (nm) (ε, $M^{-1}\cdot cm^{-1}$) = 300 (20.3 x 10^3), 312 (19.2 x 10^3), 325 (15.6 x 10^3). **ATR-FTIR:** ν (cm^{-1}) = 3096, 2920, 2849, 1604, 1562, 1470, 1438, 1368, 1224, 1033, 793.

Synthesis of FeT_L. This catalyst was synthesized analogously to **CoT_L**,[2] but replacing $[Co^{II}(H_2O)_6](BF_4)_2$ with $[Fe^{II}(H_2O)_6](BF_4)_2$. Dark fuchsia powder (16 mg, 24 %). **EA results:** Calcd. for $C_{62}H_{86}N_6O_2B_2F_8Fe\cdot 0.5H_2O$ (*M* = 1185.87 $g\cdot mol^{-1}$): C 62.80, H 7.39, N 7.09 %. Found: C 62.54, H 7.14, N 7.13 %. **ESI-MS (+, methanol):** m/z calcd. for $C_{62}H_{86}N_6O_2Fe^{2+}$ (i.e. M^{2+}) 501.3075, found 501.3091. **UV-vis (methanol):** λ_{max} (nm) (ε, $M^{-1}\cdot cm^{-1}$) = 316 (22.5 x 10^3), 363 (2.9 x 10^3), 514 (5.0 x 10^3), 556 (7.0 x 10^3). **ATR-FTIR:** ν (cm^{-1}) = 3092, 2920, 2848, 1615, 1469, 1426, 1393, 1363, 1216, 1057, 789.

Synthesis of CoT_W. This synthesis protocol was adapted from ref.[3] This cobalt catalyst was synthesized in a similar manner to **NiT_W** but replacing anhydrous $NiCl_2$ with anhydrous $CoCl_2$. Deep red crystalline powder (15.0 mg, 10 %). **EA results:** Calcd. for $C_{30}H_{22}N_6Cl_2Co\cdot 3.55H_2O$ (*M* = 660.34 $g\cdot mol^{-1}$): C 54.57, H 4.44, N 12.73 %. Found: C 54.14, H 4.09, N 12.33 %. **ESI-MS (+, methanol):** m/z calcd. for $C_{30}H_{22}N_6ClCo^+$ (i.e. $[M^{2+} + Cl^-]^+$) 560.0969, found 560.0926. **UV-vis (methanol):** λ_{max} (nm) (ε, $M^{-1}\cdot cm^{-1}$) = 318 (20.0 x 10^3), 444 (8.3 x 10^2), 505 (6.7 x 10^2), 551 (2.3 x 10^2). **ATR-FTIR:** ν (cm^{-1}) = 3058, 1597, 1560, 1470, 1448, 1400, 1246, 1160, 1015, 769.

Synthesis of NiT_W. The synthesis protocol was adapted from ref.[3] 2,2':6',2''-terpyridine (**T**, 124 mg, 530 µmol) and $NiCl_2$ (33 mg, 250 µmol) were dissolved in methanol:$CHCl_3$ (1:1, 10 mL) producing a pale brown-salmon solution that stirred for 2 h at room temperature. Subsequently, the volume was reduced to one third and left overnight in the freezer forming a precipitate that was filtered, washed with cold methanol, and dried under vacuum to yield a pale salmon powder (80 mg, 69 %). **EA results:** Calcd. for $C_{30}H_{22}N_6NiCl_2 \cdot 4H_2O$ (M = 668.22 g·mol^{-1}): C 53.92, H 4.53, N 12.58 %. Found: C 53.65, H 4.16, N 12.35 %. **ESI-MS (+, methanol):** m/z calcd. for $C_{30}H_{22}N_6ClNi^+$ (i.e. $[M^{2+}+Cl^-]^+$) 559.0948, found 559.0955. **UV-vis (methanol):** λ_{max} (nm) (ε, $M^{-1} \cdot cm^{-1}$) = 310 (15.3 x 10^3), 321 (23.5 x 10^3), 336 (23.9 x 10^3). **ATR-FTIR:** ν (cm^{-1}) = 3017, 1600, 1574, 1472, 1448, 1320, 1251, 1220, 1168, 1015, 778.

Synthesis of FeT_W. This iron catalyst was synthesized in a similar manner to **NiT_W** but replacing anhydrous $NiCl_2$ with $FeCl_2$.[3] Deep fuchsia powder (52 mg, 38 %). **EA results**: Calcd. for $C_{30}H_{22}N_6FeCl_2 \cdot 2H_2O \cdot 0.5CH_3OH$ (M = 645.35 g·mol^{-1}): C 56.77, H 4.37, N 13.02 %. Found: C 56.67, H 4.05, N 12.70 %. **ESI-MS (+, methanol):** m/z calcd. for $C_{30}H_{22}N_6Fe^{2+}$ (i.e. M^{2+}) 261.0622, found 261.0633. **UV-vis (methanol):** λ_{max} (nm) (ε, $M^{-1} \cdot cm^{-1}$) = 320 (54.3 x 10^3), 363 (4.4 x 10^3), 484 (5.6 x 10^3), 551 (12.2 x 10^3), 607 (2.3 x 10^3). **ATR-FTIR:** ν (cm^{-1}) = 3034, 1603, 1447, 1397, 1284, 1245, 1160, 1031, 776.

Synthesis of porphyrin-based ligand and catalysts

Synthesis of P_L. To a stirring dry dimethylformamide solution (15 mL) of 5,10,15,20-(tetra-4-pyridyl)porphyrin (**P**, 300 mg, 485 µmol) under N_2 atmosphere, 1-bromohexadecane (1.5 mL, 4.9 mmol) was added, and the solution was refluxed at 130 °C for 12 h. Then after cooling to room temperature the solution was taken to dryness and the residue was taken up in a $CHCl_3$:methanol (85:15, 10 mL) solution, followed by the addition of acetone (200 mL) resulting in the formation of a dark precipitate that was filtered off, washed with acetone (2 x 25 mL) and air dried for 12 h. The isolated solid was recrystallized in ethanol (200 mL) using 21 mL of ethanol per each 92 mg of solid, and the solution was kept in the freezer for 12 h, and the resulting dark precipitate was filtered and washed with ethanol (2 x 20 mL) and diethyl

ether (2 x 20 mL) and dried in vacuo to give a dark brown solid (609 mg, 66 %). **EA results:** Calcd. for $C_{104}H_{158}N_8Br_4\cdot3H_2O$ (*M* = 1894.11 g·mol^{-1}): C 65.95, H 8.73, N 5.92 %. Found: C 65.65, H 8.64, N 6.05 %. **^{1}H NMR (DMSO-d$_6$, 400 MHz):** δ (ppm) = 9.59-9.57 (d, 8H$_d$), 9.23 (s, 8H$_b$), 9.03-9.01 (d, 8H$_c$), 4.98-4.94 (t, 8H$_e$), 2.32-2.24 (q, 8H$_f$), 1.64-1.57 (q, 8 H$_g$), 1.53-1.47 (q, 8H$_h$), 1.44-1.18 (m, 88 H$_i$), 0.86-0.82 (t, 12 H$_j$), –3.09 (s, 2 H$_a$). **^{13}C NMR (DMSO-d$_6$, 100 MHz):** δ (ppm) = 156.46, 154.30, 153.39, 143.49, 132.56, 126.45, 122.89, 115.92, 114.59, 114.37, 110.68, 60.93, 31.33, 31.12, 29.17, 29.16, 29.12, 29.05, 29.02, 28.77, 28.75, 25.83, 22.13, 14.00. **ESI-MS (+, DMSO):** m/z calcd. For $C_{104}H_{158}N_8Br_3^+$ (i.e. [M^{4+} + 3 x Br^-]$^+$) 1755.9977, found 1756.0613; $C_{104}H_{158}N_8Br_2^{2+}$ (i.e. [M^{4+} + 2 x Br^-]$^{2+}$) 838.5394, found 838.5403. **UV-vis (methanol):** λ_{max} (nm) = 426, 516, 550, 592, 650. **ATR-FTIR:** ν (cm^{-1}) = 3399 (broad), 3029, 2918, 2849, 1635, 1560, 1508, 1452, 1169, 969, 816, 720.

Synthesis of CoP$_L$. 5,10,15,20-(tetra-N-hexadecyl-4-pyridyl)porphyrin tetrabromide (**P$_L$**, 57 mg, 30 µmol) and [Co^{II}(OAc)$_2$]·4H$_2$O (69 mg, 277 µmol) heated in dry dimethylformamide (5 mL) at 130 °C for 3 h. The solution was cooled to room temperature and poured into diethyl ether (100 mL) forming a brown precipitate that was filtered off, washed with diethyl ether (2 x 25 mL) and dried in vacuo for 2 hours. The solid was dissolved in an acetone:methanol (90:10, 10 mL) solution and an aqueous saturated $NaPF_6$ solution (4 mL) was added under stirring causing the precipitation of a dark brown solid that was filtered and washed with water (5 x 10 mL) and diethyl ether (2 x 10 mL). Subsequently, this solid was re-dissolved in acetone (3 mL) and precipitated again with water (10 mL), filtered, washed with water (2 x 10 mL) and dried at room temperature in vacuo for 12 h to yield a dark brown solid (37 mg, 57 %). **EA results:** Calcd. for $C_{104}H_{156}N_8P_4F_{24}Co\cdot3H_2O$ (*M* = 2211.27 g·mo^{-1}): C 56.49, H 7.38, N 5.07 %. Found: C 56.18, H 7.32, N 5.26 %. **ESI-MS (+, methanol):** m/z calcd. for $C_{104}H_{156}N_8P_3F_{18}Co^+$ (i.e. [M^{4+} + 3 x PF_6^-]) 2011.0710, found 2011.0836; $C_{104}H_{156}N_8Co^{4+}$ (i.e. M^{4+}) 394.2949, found 394.2888. **UV-vis (acetone):** λ_{max} (nm) (ε, M^{-1}·cm^{-1}) = 426 (10.7 x 10^4), 455 (6.8 x 10^4), 530 (2.0 x 10^4), 676 (0.6 x 10^4). **ATR-FTIR:** ν (cm^{-1}) = 3663, 3134, 2922, 2852, 1637, 1553, 1458, 1356, 1170, 1003, 829, 717, 556.

Synthesis of NiP$_L$. This catalyst was synthesized analogously to **CoP$_L$** but replacing $[Co^{II}(OAc)_2]\cdot 4H_2O$ with $[Ni^{II}(OAc)_2]\cdot 4H_2O$. Red brown powder (97 mg, 71 %). **EA results:** Calcd. for $C_{104}H_{156}N_8P_4F_{24}Ni$ (M = 2157.00 g·mol^{-1}): C 57.91, H 7.29, N 5.19 %. Found: C 57.95, H 7.15, N 5.08 %. **ESI-MS (+, acetone):** m/z calcd. for $C_{104}H_{156}N_8P_3F_{18}Ni^+$ (i.e. $[M^{4+} + 3 \times PF_6^-]^+$) 2010.0732, found 2010.0857; $C_{104}H_{156}N_8P_2F_{12}Ni^{2+}$ (i.e. $[M^{4+} + 2 \times PF_6^-]^{2+}$) 933.0556, found 933.0320; $C_{104}H_{156}N_8Ni^{4+}$ (i.e. M^{4+}) 394.0455, found 394.0375. **UV-vis (acetone):** λ_{max} (nm) (ε, M^{-1}·cm^{-1}) = 420 (10.8 x 10^4), 531 (9.9 x 10^3), 562 (4.8 x 10^3). **ATR-FTIR:** ν (cm^{-1}) = 3666, 3135, 2923, 2852, 1638, 1560, 1513, 1458, 1356, 1170, 1005, 830, 716, 555.

Synthesis of FeP$_L$. This catalyst was synthesized analogously to **CoP$_L$** but replacing $[Co^{II}(OAc)_2]\cdot 4H_2O$ with $FeCl_2$ and sodium acetate (1 eq. Fe : 2 eq. acetate). Greenish brown powder (88 mg, 64 %). **EA results:** Calcd. for $C_{104}H_{156}N_8P_4F_{24}FeCl\cdot 3H_2O$ (M = 2128.05 g·mol^{-1}): C 55.67, H 7.28, N 4.99%. Found: C 55.49, H 6.90, N 5.04%. **ESI-MS (+, acetone):** m/z calcd. for $C_{104}H_{156}N_8P_2F_{12}FeCl^{2+}$ (i.e. $[M + 2 \times PF6^-]^{2+}$) 949.5399, found 949.5533. **UV-vis (acetone):** λ_{max} (nm) (ε, M^{-1}·cm^{-1}) = 418 (6.7 x 10^4), 458 (3.6 x 10^4), 571 (9.2 x 10^3), 622 (3.6 x 10^3). **ATR-FTIR:** ν (cm^{-1}) = 3657, 3136, 2922, 2852, 1637, 1527, 1458, 1377, 1169, 1085, 1022, 830, 720, 556.

Synthesis of CoP$_W$. The synthesis was carried out following reported protocol from ref [4] and the physicochemical characterisation of **CoP$_W$** matches that reported in the same ref. Dark brown powder (36 mg, 50 %). **EA results:** Calcd. for $C_{44}H_{36}N_8P_4F_{24}Co\cdot 2.5H_2O$ (M = 1360.65 g·mol^{-1}): C 38.84, H 3.04, N 8.24 %. Found: C 38.39, H 2.57, N 8.09 %. **ESI-MS (+,acetone):** m/z calcd. for $C_{44}H_{36}N_8P_3F_{18}Co^+$ (i.e. $[M^{4+} + 3 \times PF_6^-]^+$) 1170.1320, found 1170.1243; $C_{44}H_{36}N_8P_2F_{12}Co^{2+}$ (i.e. $[M^{4+} + 2 \times PF_6^-]^{2+}$) 512.5834, found 512.5787. **UV-vis (acetone):** λ_{max} (nm) (ε, M^{-1}·cm^{-1}) = 423 (10.5 x 10^4), 490 (1.3 x 10^4), 531 (1.2 x 10^4), 664 (2.0 x 10^3); **UV-vis (Milli-Q water):** λ_{max} (nm) (ε, M^{-1}·cm^{-1}) = 437 (10.3 x 10^4), 550 (9.5 x 10^3), 590 (3.3 x 10^3); **ATR-FTIR:** ν (cm^{-1}) = 3655, 3138, 3063, 1641, 1556, 1516, 1465, 1355, 1278, 1190, 1088, 1002, 826, 716, 555.

Synthesis of NiP$_W$. This catalyst was prepared by anion exchange of the commercial chloride-containing analogue. The method followed is analogous to that described for **CoP$_W$**. Reddish brown powder (20 mg, 76 %). **EA results:** Calcd. for $C_{44}H_{36}N_8P_4F_{24}Ni·2.4H_2O$ (*M* = 1358.63 g·mol^{-1}): C 38.90, H 3.03, N 8.25 %. Found: C 39.03, H 2.72, N 7.94 %. **ESI-MS (+, acetone):** m/z calcd. for $C_{44}H_{36}N_8P_3F_{18}Ni^+$ (i.e. [M^{4+} + 3 x PF_6^-]) 1169.1342, found 1169.1393. **UV-vis (acetone):** $λ_{max}$ (nm) (ε, $M^{-1}·cm^{-1}$) = 423 (10.0 x 10^4), 516 (8.9 x 10^3), 551 (4.2 x 10^3), 590 (4.0 x 10^3). **ATR-FTIR:** ν (cm^{-1}) = 3668, 3135, 1641, 1574, 1513, 1460, 1403, 1278, 1186, 974, 829, 733, 555.

Synthesis of FeP$_W$. This catalyst was prepared by anion exchange of the commercial chloride-containing analogue. The method followed is analogous to that described for **CoP$_W$**. Greenish brown powder (15 mg, 80 %). **EA results**: Calcd. for $C_{44}H_{36}N_8P_4F_{24}FeCl·6.7H_2O·3NaPF_6$ (*M* = 1972.54 g·mol^{-1}): C 26.79, H 2.52, N 5.68%. Found: C 27.13, H 2.45, N 5.48 %. **ESI-MS (+, acetone):** m/z calcd. for $C_{44}H_{36}N_8P_2F_{12}FeCl^{2+}$ [M+2 x PF_6^- + Cl^-]$^{2+}$ 528.5687, found 528.5686. **UV-vis (acetone):** $λ_{max}$ (nm) (ε, $M^{-1}·cm^{-1}$) = 415 (13.5 x 10^4), 458 (5.0 x 10^4), 570 (1.5 x 10^4), 625 (5.5 x 10^3). **ATR-FTIR: ν (cm^{-1})** = 3653, 3137, 1642, 1529, 1463, 1276, 1191, 1089, 1005, 828, 719, 555.

Synthesis of alkylated bipyridine ligand and ruthenium photosensitizer

Synthesis of Bpy$_L$. The synthesis was carried out following reported protocol from ref [5] and the physicochemical characterisation matches that reported in the same ref. To diisopropylamine (4.10 mL, 2.94 g, 29.00 mmol) in dry THF at 0 °C was added n-BuLi in hexane (2.5 M, 11.60 mL, 29.00 mmol). This mixture was stirred at 0 °C for 1 h. At 0 °C 4,4'-dimethyl-2,2'-bipyridine (2.00 g, 10.86 mmol) in dry THF was added via a syringe and stirring at 0 °C was continued for 3 h before adding hexadecyl bromide (8.80 g, 29.0 mmol) in dry THF at 0 °C. The mixture was stirred at room temperature for 3 days. The reaction mixture was poured onto water with ice (200 mL). The aqueous phase was extracted with diethyl ether (1 x 500 mL). The organic solvent was removed in vacuo and the solid was recrystallized from pentane (100 mL). Filtration and drying at air yielded 4,4'-diheptadecyl-2,2'-bipyridine (**Bpy$_L$**)

as white solid (2.20 g, 32 %). **EA results:** Calcd. for $C_{44}H_{76}N_2$ (M = 633.10 g·mol^{-1}): C 83.47, H 12.10, N 4.42 %, found: C 83.43, H 12.07, N 4.39 %. **^{1}H NMR ($CDCl_3$, 400 MHz):** δ (ppm) = 8.56-8.55 (d, J = 5.0 Hz, 2 H_a), 8.23 (d, J = 1.7 Hz, 2 H_c), 7.13-7.12 (dd, J = 5.0, 1.7 Hz, 2 H_b), 2.71-2.67 (t, 4 H_d), 1.72-1.65 (q, J = 7.4 Hz, 4H_e), 1.38-1.22 (m, 56 H_6), 0.89-0.86 (t, J = 6.7 Hz, 6H_g). **^{13}C NMR ($CDCl_3$, 100 MHz):** δ (ppm) = 156.19, 152.92, 148.96, 123.89, 121.32, 35.55, 31.92, 30.46, 29.69, 29.65, 29.53, 29.43, 29.36, 29.33, 22.69, 14.10. **ESI-MS (+, methanol):** m/z calcd. for $C_{44}H_{77}N_2^+$ (i.e. MH^+) 633.6087, found: 633.6067. **UV-vis ($CHCl_3$):** λ_{max} (nm) = 284. **ATR-FTIR: ν (cm^{-1})** = 3062, 2955, 2917, 2847, 1598, 1547, 1469, 1419, 1384, 1112, 993, 896, 845, 827, 719, 669, 592, 508.

Synthesis of RuPS$_L$. The synthesis was carried out following reported protocol from ref [5] and the physicochemical characterisation matches that reported in the same ref. A mixture of [Ru(Cl)$_2$(bpy)$_2$] (754 mg, 1.56 mmol) and 4,4'-diheptadecyl-2,2'-bipyridine (978 mg, 1.54 mmol) in a 1:1:1 mixture of ethanol:water:chloroform (60 mL) was degassed via N_2 bubbling for 15 min and then heated at 110 °C for 2 days. After cooling to room temperature the solvent mixture was removed in vacuo. The reaction residue was subjected to column chromatography (stationary phase: SiO_2; 1st eluent: acetone, 2nd eluent: 8:4:1 acetone:water:brine, 3rd eluent: 100:10:1 acetone:water:aqueous sat. $KNO_{3[aq]}$) to isolate the red-orange fraction. The solvent was removed in vacuo and the red compound was extracted with chloroform. The combined organic layers were dried over $MgSO_4$ and taken to dryness. The red solid was taken up in methanol and subjected to ion exchange column with Amberlite (50 g, presoaked with brine and washed 10 times with water and 3 times with methanol). The solvent was removed, and the red solid was taken up in a mixture of chloroform and 1:1 water:brine. The two phases were separated, and the aqueous phase was extracted with chloroform. The combined organic layers were dried with $MgSO_4$ and the solvent was evaporated in vacuo. Trituration of the solid in acetone (100 mL) followed by removal of 50 mL of acetone at the rotavap, cooling to room temperature and filtration and washing with acetone (50 mL) yielded the desired compound as ([Ru(bpy)$_2$(Bpy$_L$)](Cl)$_2$·7H_2O) (1.10 g,

60 %). **EA results:** Calcd. for $C_{64}H_{92}N_6RuCl_2·7H_2O$ (M = 1243.56 g·mol^{-1}): C 61.81, H 8.59, N 6.76 %. Found: C 61.47, H 8.27, N 6.43 %. **^{1}H NMR (CD_3OD, 400 MHz):** δ (ppm) = 8.71-8.69 (d, J = 8.2 Hz, 4$H_{d'}$), 8.62 (d, J = 1.9 Hz, 2H_d), 8.14-8.10 (t, 4$H_{c'}$), 7.82-7.81 (m, 4$H_{a'}$), 7.64-7.62 (d, J = 5.8 Hz, 2H_a), 7.51-7.46 (dtd, J = 7.2, 5.7, 1.3 Hz, 4$H_{b'}$), 7.35-7.34 (dd, J = 5.9, 1.8 Hz, 2H_b), 2.87-2.83 (t, J = 7.9 Hz, 4H_e), 1.77-1.70 (q, J = 7.3 Hz, 4H_f), 1.40-1.25 (m, 56H_g), 0.91-0.88 (t, J = 6.6 Hz, 6H_h). **^{13}C NMR (CD_3OD, 100 MHz):** δ (ppm) = δ 158.63, 158.60, 158.19, 156.56, 152.65, 152.52, 151.87, 139.01, 128.95, 128.84, 125.67, 125.49, 36.26, 33.09, 31.35, 30.80, 30.77, 30.74, 30.63, 30.49, 30.44, 30.42, 23.75, 14.47. **ESI-MS (+, methanol)** m/z calcd. for $C_{64}H_{92}N_6ClRu^+$ (i.e. [M^{2+} + Cl^-]$^+$): 1081.6115, found: 1081.5840. **UV-vis (methanol):** λ_{max} (nm) (ε, M^{-1}·cm^{-1}) = 454 (13.5 x 10^3). **ATR-FTIR:** ν (cm^{-1}) = 3381 (broad), 3068, 2955, 2920, 2851, 1615, 1463, 1422, 1313, 1244, 1160, 1123, 1026, 772, 730.

Sample preparation

Preparation of liposomes. Method A was utilized to prepare DMPC/DLPC/DPPC-based liposomes (see main text). Method B was employed to prepare DMPC-based liposomes with different DMPC:**RuPS$_L$** ratios to investigate their effect in self-quenching of membrane-bound **RuPS$_L$** and photocatalytic CO_2 reduction (see supplementary sections *photophysical dynamics investigations* and *photocatalysis experiments*, respectively).

Method A: Aliquots of lipid DMPC (0.25 mL of a 10 mM chloroform solution), NaDSPE-PEG2K (0.25 mL of a 0.1 mM chloroform solution), **RuPS$_L$** (0.25 mL of a 1 mM methanol solution) and catalyst [ranging from 0.02 to 0.25 mL of 50 μM in methanol solution (**MT$_L$**) or 50 μM acetone solution (**MP$_L$**)] were added to a reaction tube (using a scale and the solvents' density for higher accuracy) and taken to dryness using a rotavapor (45 °C, 300 mm Hg) to yield a lipid film. The film was dried in vacuo for 30 min and hydrated with aqueous 0.1M $NaHCO_3$ solution (0.5 mL). The lipid film was repeatedly freeze-thaw-sonication three times (i.e., it was frozen using liquid N_2, thawed using a water bath at 45 °C (DLPC and DMPC) and 55°C (DPPC) and

followed by sonication for 10 sec). This process resulted in a homogenously dispersed solution that was extruded through a 0.2 µm polycarbonate filters (19 mm) at 45 °C (DLPC and DMPC) and 55 °C (DPPC). A photocatalysis sample contains an aliquot of the liposome extruded solution (60 µL) diluted in an aqueous solution containing 0.1 M $NaHCO_3$ and 0.1 M sodium ascorbate (2.94 mL) to achieve a final concentration of lipid:NaDSPE-PEG2K:**RuPS$_L$** of 100 µM:1 µM:10 µM, with a variable catalyst concentration of 500 nM, 200 nM, 50 nM and 20 nM (see DLS results in Tables S4-S6). Subsequently, this solution was purged with either CO_2:CH_4 or N_2:CH_4 (98:2) for 20 minutes before starting a light irradiation experiment.

Method B: This protocol differs from *Method A* in that only lipid DMPC was utilized and the volume of lipids DMPC and NaDSPE-PEG2K (10 mM and 0.1mM chloroform solution respectively) was doubled or quadrupled, and while the concentration of **RuPS$_L$** is maintain the same as in *Method A* to have the same optical density (i.e. final concentration of **RuPS$_L$** = 10 µM), to obtain a final concentration (after extrusion and dilution) of lipid:NaDSPE-PEG2K:**RuPS$_L$** equal to 200µM:2µM:10µM or 400µM:4µM:10µM. The final concentration of catalysts was also 500, 200, 50 and 20 nM.

Homogeneous samples. Homogeneous control samples were prepared analogously to liposome samples from Method A, using the same concentrations for the molecular species but without any lipids. Alkylated catalysts and **RuPS$_L$**, water-soluble catalysts **MT$_W$** (M = Co, Fe, Ni) and **MP$_W$** (M = Co, FeCl, Ni) and photosensitizer $[Ru(bpy)_3](Cl)_2$ (**RuPS$_W$**) were utilized instead. Furthermore, due to the lack of lipids, there was no need to conduct frozen-thawed-sonicated cycles or extrude the solution.

Supplementary notes

Supplementary Note 1 regarding liposome screening. Cryo-TEM results (Figure S17) reveal that liposomes made of 100 µM DMPC, DLPC or DPPC containing NaDSPE-PEG2K, **RuPS$_L$** and **NiT$_L$**, are all smoothly rounded. Pure DMPC and DPPC liposomes should give

facetted liposomes below their transition phase temperature (Tm).[6] We attribute the fact that DPPC and DLPC liposomes are smooth at room temperature in the present study to the fact that they are heavily doped (i.e. contain 1% NaDSPE-PEG2K and 10% **RuPS$_L$** and catalyst), which probably lowers their Tm compared to that of pure DMPC or DPPC liposomes.

These results can be further supported by electron transfer (ET) kinetic measurements carried out at room temperature with liposomes made of DLPC, DMPC and DPPC containing (A) 1µM NaDSPE-PEG2K and 10 µM **RuPS$_L$** or (B) 1µM NaDSPE-PEG2K and 10 µM **RuPS$_L$** and 2 µM **NiT$_L$** (see Figure S36). Results show a similar rate of bleaching of **RuPS$_L^-$** for all three liposomes when both dye and catalyst molecules are present, with DMPC and DLPC liposomes exhibiting a slightly faster decay of **RuPS$_L^-$** than DPPC liposomes. Furthermore, trends in photocatalysis results are coherent with expectations based on the aforementioned ET kinetics results (see Figure S25 and Table S13). Experiments carried out using DMPC, DLPC and DPPC liposomes with 100 µM lipids, 1 µM NaDSPE-PEG2K, 10 µM **RuPS$_L$** and 0.5 µM **CoP$_L$** showed that DMPC and DLPC performed within experimental error approximately the same, with DMPC yielding slightly better results, compared to DPPC liposomes.

Overall, these screening results highlight that all three liposomes were in the liquid crystal (fluid) phase at room temperature, and that DMPC was the best building block to construct our self-assembled system to study CO_2 reduction and light-driven ET kinetics.

Supplementary Note 2 regarding Figure S30C and S30D. To elucidate the non-exponential decay of **RuPS$_L$** in DMPC liposomes, different surface concentrations of dyes were used (Figure S30C), and the lifetime increased with dilution of the dye. Excitation-pulse-energy-dependent measurements were further employed to examine the emission decays at 650 nm in liposomes with a mole ratio 10:1 between DMPC and **RuPS$_L$** (Figure S30D). The emission decays show laser-power independent dynamics by using pump laser power at 20-50

mJ/pulse, which is different from the behaviour expected for triplet-triplet annihilation processes.[7] Therefore, we can ascribe the short-lifetime component in the emission decays to the self-quenching of $\mathbf{RuPS_L}$ in the surface of DMPC liposomes, i.e. quenching by ground state $\mathbf{RuPS_L}$ molecules. In support of this assignment, we also observe a faster decay on the blue edge of the emission band (600 nm) than near the band maximum (650 nm; Figure S30B). This is consistent with heterogeneity of dye interactions at higher surface concentrations, where high-energy sites may be quenched by energy transfer to dyes at low-energy sites. The corresponding effect is absent at the same concentration of dye in the homogeneous solution (Figure S30A). The rate constants and fitting parameters are summarized in Table S14.

Supplementary Note 3 regarding Figure S31. Time-resolved absorption spectroscopy can be also used to obtain light-driven charge separation yields between photosensitizer and quencher molecules. The charge separation quantum yield between photosensitizer and quencher in homogenous environment and liposomes (φ_{ET}) in Figure S31 was estimated as φ_{ET} = [Ru(I)]/ [Ru*] using the difference in molar attenuation coefficients for different Ru species, i.e. $\Delta\varepsilon_{Ru*-Ru(II)}$ = 1.1 10^4 M^{-1} s^{-1} at 450 nm and $\Delta\varepsilon_{Ru(I)-Ru(II)}$ = 1.3 10^4 M^{-1} s^{-1} at 510 nm. In homogenous conditions, for a solution with [$\mathbf{RuPS_W}$] = 20 µM and 0.1 M ascorbate in 0.1 M $NaHCO_3$ buffer and concentration values of $[Ru^*]_{t=0}$ = 2.4 µM and $[Ru(I)]_{max}$ = 0.84 µM, the φ_{ET} is 35 %. In liposomes, for a solution with [DMPC] = 100 µM, [NaDSPE-PEG2K] = 1 µM, [$\mathbf{RuPS_L}$] = 10 µM and 0.1 M ascorbate in 0.1 M $NaHCO_3$ buffer, and values of $[Ru^*]_{t=0}$ = 1.27 µM and $[Ru(I)]_{max}$ = 0.07 µM, the φ_{ET} is 6 %. This lower quantum yield value in liposomes could be explained by the coulombic association (or ion-pair formation) between the cationic photosensitizer and the anionic ascorbate in the ground state, resulting in undesirable charge recombination and lower charge separation quantum yield.

Supplementary Note 4 regarding Figure 3D. The rate of electron transfer reaction $\mathbf{RuPS_W^-}$ + $\mathbf{NiT_W}$ → $\mathbf{RuPS_W}$ + $\mathbf{NiT_W^-}$ can be described as $v = k_{et}$ [$\mathbf{RuPS_W^-}$] [$\mathbf{NiT_W}$], when [$\mathbf{NiT_W}$] >>

[$\mathbf{RuPS_W^-}$]. The reaction has a pseudo-first-order behavior and thus $v = k_{obs}$ [$\mathbf{RuPS_W^-}$], i.e. $k_{obs} = k_{et}$ [$\mathbf{NiT_W}$] and hence k_{et} can be extracted from the k_{obs} vs [$\mathbf{NiT_W}$] plot. The kinetic traces of 100, 200, 300 µM $\mathbf{NiT_W}$ (Figure 3D) can be fitted well by using biexponential decays (see Table S14 for all time constants and corresponding amplitudes). The longer component is ascribed to the decay of reduced catalyst $\mathbf{NiT_W^-}$, and is therefore not considered into the k_{et} calculation. In addition, without catalyst the reduced photosensitizer $\mathbf{RuPS_W^-}$ can recombine with oxidized ascorbate, hence such recombination reaction must be considered as it is in competition with an ET from $\mathbf{RuPS_W^-}$ to $\mathbf{NiT_W}$. Therefore, $k_{obs} = 1/\tau_1 - 1/\tau_0$, where τ_1 is the short-lifetime components with different catalyst concentrations, and τ_0 is the recombination reaction lifetime without catalyst.

Supplementary Note 5 regarding Table S14. The ET rate constants in Table S14 suggest that at 100 µM $\mathbf{NiT_L}$ the fast kinetic component lifetime (3.5 µs) is ca. five times shorter than the lifetime in the absence of catalyst (18.5 µs), suggesting ~80% efficiency of electron transfer to the catalyst. Similarly, for the liposomes at 5 µM $\mathbf{NiT_W}$ the fast component is reduced ca. four-fold, from 28 µs to 7.4 µs. Also the slow component without catalyst clearly reacts and leads to a loss in long-lived amplitude, which is attributable to electron transfer to catalyst. This efficiency estimate is more uncertain than in homogeneous solution, due to the heterogeneous system giving heterogeneous kinetics. We estimate that the yield is in the range 57-82%, based on a lower limit of the amplitude of the fast phase, and an upper limit of the combined amplitudes of the first and second phases.

Supplementary Note regarding Figure S39B. The slightly different position of the reduction waves in GCE|$\mathbf{CoP_L}$ (i.e. –0.30 and –0.50 V vs SHE in CO_2) vs FTO|$\mathbf{CoP_L}$ (i.e. –0.10 and –0.35 V in CO_2) highlights the effect that surface choice and film self-assembly can have on the electrochemical behaviour.[8]

Supplementary Note 6 regarding Figure S41. Re-oxidation of the reduced FTO|**CoP$_L$** films, by re-applying +0.7 V after the stepwise reduction, shows that under N_2 the absorption of the re-oxidized film remains similar to the as-prepared film (Figure S41A).[9] Under CO_2 the re-oxidized film shows a hypochromic shift of the Soret band and two new absorptions at 618 and 802 nm (Figure S41B), which can be attributed to the possible non-reversible reduction of the porphyrin ligand core to chlorin or bacteriochlorin[10] or the formation of the proposed catalytic intermediate $[CoP_L(CO)]^{2+}$ species (see Figure 5). An analogous results was also observed with Resonance Raman SEC under CO_2 conditions (Figure S43).

Supplementary Note 7 regarding DFT matching. We have calculated the spectra for several species using a simplified DFT model that omits lipophilic alkyl chains of the catalyst species (Figures S49-55). This simplified model was chosen to significantly decrease the computational cost associated with screening several oxidation states and adducts.

Comparing DFT results to experimental data at +0.7 V vs SHE (where no adduct or H_2O-adduct are expected) and at –0.9 V vs SHE (where H_2O- and CO-adducts are expected), we found that the pyridinium modes between 1150-1250 cm^{-1} and 1635 cm^{-1} are reproduced. Modes in the lower wavenumber region < 900 cm^{-1} are broad in the experimental spectra, presumably due to the disordered film structure.[8] The DFT correctly predicts a strong response at ≈1360 cm^{-1} corresponding to the porphyrin core, while additional broad modes are also observed in the 1300-1350 cm^{-1} region that are not found in the DFT or in previous work.[11] This spectral region hosts various alkyl chain modes such as the CH_2 twisting mode and CH_3 bend[12] and therefore it is likely that these modes, not included in the DFT model, are assigned here.

Having benchmarked the DFT model, we compared the calculated spectra and Gibbs free energies for several different oxidation states to assist identifying the reduced species (Figures

S45 and S46 for summary of calculated Raman spectra, and Tables 17-18 for summary of Gibbs free energies).

Supplementary tables

Summary of electrochemical data of all catalysts in organic media

Table S1. Summary of electrochemical data for all catalysts in N_2- and CO_2-saturated 0.2 M $TBAPF_6$ DMF solution given in V vs $Fc^{0/+}$ as half-wave potential with the peak separation in brackets or for irreversible waves (irr) indicating if they are oxidation (ox) and reduction (red) processes. In those cases where a peak is very similar in CO_2/N_2 only one value is provided, unless otherwise stated within brackets.

Catalyst	CV		SWV		Reference
**CoT_L**	N_2	–0.20 (0.08), –1.19 (0.07), –2.04 (0.08)	N_2	–0.20, –1.19, –2.05	[13]
	CO_2	–0.20 (0.08), –1.20 (0.06), –2.14 (red, irr)	CO_2		
**CoT_W**	N_2	–0.04 (0.08), –1.06 (0.08), –1.83 (0.07)	N_2	0.04, –1.05, –1.84	[3]
	CO_2	0.03 (0.09), –1.08 (0.07), –1.88 (red, irr)	CO_2		
**NiT_L**	N_2	–1.73 (0.10), –1.95 (0.14), –2.12 (0.09), –2.25 (0.07)	N_2	–1.75, –1.96, –2.13, –2.26	This work
	CO_2	–1.79 (red, irr), –1.96 (red, irr), –2.25 (red, irr)	CO_2	–1.75, –1.97, –2.20	
**NiT_W**	N_2	–1.58 (0.12), –1.90 (0.11)	N_2	–1.63, –1.93	[3, 14]
	CO_2	–1.68 (red, irr), –1.95 (red, irr), –2.21 (red, irr)	CO_2	–1.63, –1.88, –2.16	
**FeT_L**	N_2	–0.17 (ox, irr), –1.75 (0.07), –1.88 (0.07)	N_2	–1.75, –1.89	This work
	CO_2	–0.16 (ox, irr), –1.20 (ox, irr), –1.75 (0.09), –1.90 (0.09), –2.01 (red, irr)	CO_2		
**FeT_W**	N_2	–1.65 (0.06), –1.78 (0.06)	N_2	–1.65, –1.79	[3]
	CO_2		CO_2		
**CoP_L**	N_2	–0.82 (0.06), –0.99 (0.07), –1.20 (0.08), –1.38 (0.06), –1.49 (0.06)	N_2	–0.81, –0.99, –1.20, –1.38, –1.48	This work
	CO_2		CO_2		
	CO_2 + 5 % H_2O	–0.82 (0.07), –1.00 (0.09), –1.18 (0.13), –1.29 (0.13), –1.49 (red, irr)	CO_2 + 5 % H_2O	–0.82, –1.0, –1.19, –1.34, –1.46	
**CoP_W**	N_2	–0.98 (0.06), –1.23 (0.07), –1.37 (0.06), –1.46 (0.06)	N_2	–0.92, –1.23, –1.38, –1.46	[4, 15]
	CO_2		CO_2		
**NiP_L**	N_2	–0.91 (in CO_2, 0.13), –0.96 (in N_2, 0.16), –1.11 (0.06), –1.32 (0.06), –1.47 (0.07), –1.96 (in N_2, 0.11), –1.82 (in CO_2, 0.18)	N_2	–1.00, –1.11, –1.32, –1.47, –1.86 (in CO_2), –1.97 (in N_2)	This work
	CO_2		CO_2		
**NiP_W**	N_2	–0.98 (0.06), –1.29 (0.06), –1.42 (0.06), –1.83 (in N_2, 0.18), –1.85 (in CO_2, 0.18)	N_2	–0.98, –1.29, –1.42, –1.86 (in N_2),–1.75 (in CO_2)	[16]
	CO_2		CO_2		
**FeP_L**	N_2	–0.62 (in CO_2, 0.10), –0.63 (in N_2, 0.13), –1.13 (in CO_2, 0.09), –1.14 (in N_2, 0.14), –1.38 (in CO_2, 0.06), –1.40 (in N_2, 0.08), –1.51 (in CO_2, 0.13), –1.54 (in N_2, 0.14), –1.83 (0.10)	N_2	–0.59, –1.19, –1.53, –1.84	This work
	CO_2		CO_2	–0.61, –1.16, –1.39, –1.48, –1.51, –1.83	
**FeP_W**	N_2	–0.63 (0.08), –1.18 (0.06), –1.38 (0.06), –1.50 (0.06), –1.97 (0.07)	N_2	–0.62, –1.19, –1.38, –1.47 (CO_2), –1.51 (N_2), –1.86 (CO_2), –1.97 (N_2)	This work
	CO_2	–0.63 (0.08), –1.17 (0.07), –1.38 (0.06), –1.46 (0.07), –1.84 (0.09)	CO_2		

Table S2. Summary of passed charges and the relative ratio between charges during chronoamperometry at different E_{appl} for **CoP$_L$** and **CoP$_W$** in DMF and GCE|**CoP$_L$** and FTO|**CoP$_L$** in 0.1 M $NaHCO_3$.

	Experimental conditions	E_{appl} (V vs $Fc^{0/+}$)	Coulombs (C) [a]	Relative ratio between Coulomb values [b]	Theoretical ratio[c]	Accumulative number of e^- [d]
**CoP$_W$**	N_2 saturated 0.2 M $TBAPF_6$ DMF	–0.90<br>–1.15<br>–1.30<br>–1.50	$2.64\ 10^{-5}$<br>$6.06\ 10^{-5}$<br>$1.03\ 10^{-4}$<br>$1.52\ 10^{-4}$	0.17<br>0.39<br>0.67<br>1.00	0.17<br>0.33<br>0.67<br>1.00	1<br>2<br>4<br>6
	CO_2 saturated 0.2 M $TBAPF_6$ DMF	–0.90<br>–1.15<br>–1.30<br>–1.50	$2.88\ 10^{-5}$<br>$6.25\ 10^{-5}$<br>$9.51\ 10^{-5}$<br>$1.53\ 10^{-4}$	0.19<br>0.41<br>0.62<br>1.00		
**CoP$_L$**	N_2 saturated 0.2 M $TBAPF_6$ DMF	–0.90<br>–1.15<br>–1.30<br>–1.50	$1.58\ 10^{-5}$<br>$3.09\ 10^{-5}$<br>$6.20\ 10^{-5}$<br>$9.52\ 10^{-5}$	0.17<br>0.33<br>0.65<br>1.00	0.17<br>0.33<br>0.67<br>1.00	1<br>2<br>4<br>6
	CO_2 saturated 0.2 M $TBAPF_6$ DMF	–0.90<br>–1.15<br>–1.30<br>–1.50	$1.56\ 10^{-5}$<br>$3.49\ 10^{-5}$<br>$5.69\ 10^{-5}$<br>$1.01\ 10^{-4}$	0.15<br>0.35<br>0.56<br>1.00		

[a] obtained from chronoamperometry measurement at E_{appl} after 60 seconds. [b] Calculated from dividing the coulomb values obtained at more anodic E_{appl} by the coulomb value obtained at E_{appl} = –1.50 V vs $Fc^{0/+}$. [c] Fractions corresponding to one (0.17), two (0.33), four (0.67) and six (1.00) e^-. [d] Calculated based on the relative experimental ratios and assuming a total number of six e^-.

Table S3. Summary of integrated areas and the relative ratio between areas obtained from the fitting reduction SWV peaks shown in Figure S16 for **CoP_L** and **CoP_W** in DMF, and for GCE|**CoP_L** and FTO|**CoP_L** in 0.1 M $NaHCO_3$ shown in Figure 5A.

	Experimental conditions	E_{red} (V vs $Fc^{0/+}$)	Integrated area (10^{-7})[a]	Area (%)	Relative ratio between % areas [b]	Number of e^{-} [c]
**CoP_W**	CO_2 saturated 0.2 M $TBAPF_6$ DMF	–0.98<br>–1.23<br>–1.37<br>–1.46	3.21<br>4.25<br>9.89<br>9.72	0.12<br>0.15<br>0.37<br>0.36	0.33<br>0.44<br>1.02<br>1.00	0.71<br>0.94<br>2.19<br>2.15
**CoP_L**	CO_2 saturated 0.2 M $TBAPF_6$ DMF	–0.80 & –0.99<br>–1.20<br>–1.37<br>–1.48	3.44 & 5.82<br>10.61<br>17.54<br>29.98	0.13 (= 0.05 + 0.08)<br>0.16<br>0.26<br>0.45	0.29<br>0.36<br>0.58<br>1.00	0.82<br>0.95<br>1.56<br>2.67
GCE|**CoP_L**	N_2 saturated 0.1 M $NaHCO_3$	–0.38<br>–0.50	16.90<br>6.28	0.73<br>0.27	2.70<br>1.00	2.92<br>1.08
	CO_2 saturated 0.1 M $NaHCO_3$	–0.32<br>–0.51	6.51<br>13.68	0.32<br>0.68	0.47<br>1.00	1.29<br>2.71
FTO|**CoP_L**	N_2 saturated 0.1 M $NaHCO_3$	–0.29<br>–0.69	96.03<br>23.36	0.80<br>0.20	4.11<br>1.00	3.22<br>0.78
	CO_2 saturated 0.1 M $NaHCO_3$	–0.11<br>–0.36 & –0.54	39.47<br>64.36 & 16.93	0.33<br>0.67 (=0.53 + 0.14)	0.49<br>1.00	1.31<br>2.69

[a] obtained using *Multi Peak Fit* function in *OriginPrO 2017*. [b] Calculated from dividing the three first % areas by the % area obtained at peaks with E_{red} = –1.46 or –1.48 V vs $Fc^{0/+}$ in **CoP_W** or **CoP_L**, respectively. [c] calculated from multiplying the % areas per 6 e^{-} for **CoP_W** and **CoP_L** in DMF, or per 4 e^{-} for GCE|**CoP_L** and FTO|**CoP_L**.

Summary of dynamic light scattering results

Table S4. Summary of DLS results obtained for different DMPC liposomes containing [DMPC] = 100 µM, [NaDSPE-PEG2K] = 1 µM, [**RuPS$_L$**] = 10 µM and [alkylated catalyst] = 20-500 nM. All experiments were conducted in 0.1 M $NaHCO_3$ solutions at 25 °C.

Lipid	Catalyst	Catalyst concentration (nM)	Buffer	Average size (nm)	Polydispersity index
DMPC	**CoP$_L$**	500	$NaHCO_3$	150 ± 6	0.09 ± 0.02
		200		144 ± 6	0.08 ± 0.02
		50		153 ± 6	0.08 ± 0.02
		20		149 ± 6	0.10 ± 0.04
		500 (No **RuPS$_L$**)		162 ± 7	0.10 ± 0.02
		0		142 ± 5	0.10 ± 0.04
		500	NaH_2PO_4	155 ± 4	0.06 ± 0.02
		200		156 ± 3	0.05 ± 0.03
		50		154 ± 5	0.09 ± 0.01
		20		153 ± 5	0.08 ± 0.02
		500 (No **RuPS$_L$**)		155 ± 3	0.12 ± 0.03
		0		198 ± 7	0.15 ± 0.04
	**CoT$_L$**	500	$NaHCO_3$	151 ± 9	0.14 ± 0.01
		200		149 ± 4	0.07 ± 0.02
		50		146 ± 6	0.07 ± 0.01
		20		155 ± 7	0.10 ± 0.02
	**NiP$_L$**	500		151 ± 6	0.07 ± 0.02
		200		149 ± 5	0.09 ± 0.03
		50		149 ± 7	0.08 ± 0.02
		20		151 ± 7	0.08 ± 0.01
	**NiT$_L$**	500		139 ± 2	0.09 ± 0.01
		200		145 ± 3	0.07 ± 0.01
		50		139 ± 2	0.10 ± 0.01
		20		133 ± 5	0.09 ± 0.02
	**FeP$_L$**	500		136 ± 1	0.07 ± 0.01
		200		137 ± 1	0.09 ± 0.01
		50		141 ± 2	0.09 ± 0.01
		20		146 ± 2	0.04 ± 0.01
	**FeT$_L$**	500		146 ± 4	0.08 ± 0.03
		200		151 ± 6	0.06 ± 0.03
		50		146 ± 5	0.06 ± 0.01
		20		144 ± 5	0.06 ± 0.02

Table S5. Summary of DLS results obtained for liposomes solutions containing different DMPC lipid-concentrations and different lipid types (DLPC, DMPC, DPPC). [lipid] = 100-400 µM, [NaDSPE-PEG2K] = 1 µM, [**RuPS$_L$**] = 10 µM and [**CoP$_L$** or **NiT$_L$**] = 500 nM. All measurements were conducted in 0.1 M $NaHCO_3$ solutions at 25 °C.

**Lipid**	**Lipid concentration (mM)**	**Catalyst**	**Catalyst concentration (nM)**	**Average size (nm)**	**DPI**
DMPC	100	**CoP$_L$**	500	150 ± 6	0.09 ± 0.02
	200			153 ± 5	0.11 ± 0.02
	400			141 ± 4	0.07 ± 0.02
DLPC	100	**CoP$_L$**	500	114 ± 2	0.11 ± 0.01
DMPC				150 ± 6	0.09 ± 0.02
DPPC				194 ± 6	0.12 ± 0.02
DLPC	100	**NiT$_L$**	500	130 ± 4	0.11 ± 0.01
DMPC				143 ± 3	0.09 ± 0.01
DPPC				173 ± 5	0.14 ± 0.01

Table S6. Summary of DLS results obtained as a function of irradiation time for liposomes solutions containing [DMPC] = 100 µM and [NaDSPE-PEG2K] = 1 µM, or [DMPC] = 100 µM and [NaDSPE-PEG2K] = 1 µM [**RuPS$_L$**] = 10 µM and [**CoP$_L$**] = 500 nM. All measurements were conducted in 0.1 M $NaHCO_3$ and 0.1 M sodium ascorbate solutions at 25 °C. NB: the difference in average liposome sizes between Tables S4 and S6 is due to isotonic vs hypotonic effects.[17]

**Time (h)**	**Irradiation**	**Content**	**Average size (nm)**	**DPI**
0	Dark	Only lipids	128 ± 2	0.129 ± 0.02
		All components	119 ± 1	0.135 ± 0.02
	Light	Only lipids	121 ± 1	0.144 ± 0.01
		All components	122 ± 1	0.118 ± 0.02
1	Dark	Only lipids	134 ± 2	0.117 ± 0.02
		All components	118 ± 1	0.239 ± 0.01
	Light	Only lipids	135 ± 1	0.110 ± 0.01
		All components	122 ± 1	0.118 ± 0.02
2	Dark	Only lipids	162 ± 5	0.257 ± 0.01
		All components	126 ± 1	0.142 ± 0.01
	Light	Only lipids	126 ± 1	0.142 ± 0.02
		All components	127 ± 1	0.172 ± 0.01
3	Dark	Only lipids	132 ± 1	0.125 ± 0.02
		All components	120 ± 1	0.184 ± 0.01
	Light	Only lipids	128 ± 1	0.128 ± 0.01
		All components	120 ± 1	0.208 ± 0.01
4	Dark	Only lipids	129 ± 1	0.131 ± 0.03
		All components	125 ± 1	0.140 ± 0.01
	Light	Only lipids	129 ± 2	0.152 ± 0.01
		All components	125 ± 2	0.170 ± 0.01

Tabulated photocatalysis results

Table S7. Summary of all photocatalytic experiment results obtained from all alkylated catalysts in DMPC liposomes. All experiments were carried out in CO_2-saturated 0.1 M $NaHCO_3$ and 0.1 M sodium ascorbate solutions for 4 hours under visible light and at 25 °C, and in triplicates. In addition to CO and H_2 no other products such as formate or CH_4 were detected. [a]CO selectivity (%) = n_{CO} / (n_{CO} + n_{H2}) x 100.

Catalyst	Catalyst concentration (nm)	Gaseous products and rate of formation after 4-hour experiments								
		CO (nmol)	H_2 (nmol)	TON_{CO}	TON_{H2}	TOF_{CO} (h^{-1})	TOF_{H2} (h^{-1})	$PTON_{CO}$	$PTON_{H2}$	CO selectivity (%)[a]
CoP_L	500	282.9 ± 11.2	54.6 ± 8.9	188.6 ± 7.5	36.4 ± 5.9	47.1 ± 11.7	9.1 ± 2.1	18.9 ± 0.4	3.6 ± 0.3	84 ± 1
	200	193.5 ± 7.2	30.9 ± 3.1	322.5 ± 12.1	51.4 ± 5.1	80.6 ± 41.5	12.9 ± 7.1	12.9 ± 0.2	2.1 ± 0.1	86 ± 1
	50	71.5 ± 1.3	18.1 ± 2.4	476.4 ± 8.6	120.5 ± 16.2	119.1 ± 19.9	30.1 ± 2.1	4.8 ± 0.1	1.2 ± 0.1	82 ± 3
	20	44.1 ± 5.5	13.5 ± 1.4	735.1 ± 91.3	224.7 ± 24.1	183.8 ± 6.2	56.2 ± 11.9	2.9 ± 0.2	0.9 ± 0.1	78 ± 4
CoT_L	500	48.9 ± 18.0	9.8 ± 2.0	32.6 ± 12.0	6.5 ± 1.3	8.1 ± 1.6	1.6 ± 0.4	3.3 ± 0.6	0.6 ± 0.1	87 ± 2
	200	95.2 ± 10.0	12.7 ± 3.3	158.6 ± 16.6	21.1 ± 5.5	39.7 ± 4.0	5.3 ± 1.4	6.3 ± 0.3	0.8 ± 0.1	92 ± 3
	50	57.7 ± 3.8	15.2 ± 0.5	384.4 ± 25.6	101.2 ± 19.4	96.1 ± 15.9	25.3 ± 4.6	3.8 ± 0.1	1.0 ± 0.1	81 ± 2
	20	35.4 ± 4.8	8.2 ± 3.8	589.6 ± 79.7	137.3 ± 64.0	147.4 ± 28.7	34.3 ± 6.4	2.4 ± 0.2	0.5 ± 0.1	82 ± 2
NiP_L	500	27.3 ± 3.9	14.6 ± 3.0	18.2 ± 2.6	9.7 ± 2.0	4.6 ± 1.1	2.4 ± 0.4	1.8 ± 0.1	1.0 ± 0.1	62 ± 6
	200	14.3 ± 2.7	5.6 ± 2.6	23.9 ± 4.5	9.4 ± 4.4	6.0 ± 1.5	2.3 ± 0.5	1.0 ± 0.1	0.4 ± 0.1	70 ± 2
	50	10.9 ± 0.6	6.8 ± 2.1	73.0 ± 3.8	45.7 ± 13.7	18.2 ± 4.7	11.4 ± 2.5	0.7 ± 0.1	0.5 ± 0.1	56 ± 6
	20	11.1 ± 3.6	4.3 ± 1.9	184.5 ± 60.5	71.8 ± 31.8	46.1 ± 14.1	18.0 ± 5.0	0.7 ± 0.1	0.3 ± 0.1	71 ± 2
NiT_L	500	24.8 ± 5.0	12.8 ± 4.5	16.5 ± 3.3	8.5 ± 3.0	4.1 ± 0.3	2.1 ± 0.6	1.7 ± 0.2	0.8 ± 0.1	74 ± 7
	200	27.6 ± 5.5	11.5 ± 2.1	46.0 ± 9.2	19.2 ± 3.4	11.5 ± 0.7	4.8 ± 1.6	1.8 ± 0.2	0.8 ± 0.1	77 ± 8
	50	26.2 ± 0.8	7.7 ± 1.3	174.3 ± 5.6	51.6 ± 8.4	43.6 ± 6.3	12.9 ± 3.1	1.7 ± 0.1	0.5 ± 0.1	79 ± 3
	20	28.5 ± 2.7	8.2 ± 3.0	474.8 ± 44.3	137.0 ± 50.3	118.7 ± 16.3	34.2 ± 11.1	1.9 ± 0.1	0.5 ± 0.1	82 ± 6
FeP_L	500	26.7 ± 3.8	8.4 ± 1.1	17.8 ± 2.5	5.6 ± 0.7	4.5 ± 1.0	1.4 ± 0.5	1.8 ± 0.1	0.6 ± 0.1	82 ± 5
	200	25.7 ± 2.5	8.4 ± 0.8	42.9 ± 4.1	14.0 ± 1.4	10.7 ± 2.6	3.5 ± 1.0	1.7 ± 0.1	0.6 ± 0.1	78 ± 2
	50	27.5 ± 3.4	23.8 ± 4.9	183.5 ± 22.4	158.6 ± 32.4	45.9 ± 10.1	39.6 ± 5.8	1.8 ± 0.1	1.6 ± 0.2	50 ± 3
	20	26.9 ± 3.2	25.0 ± 3.5	448.3 ± 53.5	416.6 ± 58.5	112.1 ± 24.6	104.1 ± 10.5	1.8 ± 0.1	1.7 ± 0.1	47 ± 5
FeT_L	500	51.9 ± 12.5	11.1 ± 2.3	34.6 ± 8.3	7.4 ± 7.4	8.7 ± 0.9	1.8 ± 0.4	3.5 ± 0.4	0.7 ± 0.1	87 ± 3
	200	38.4 ± 4.3	9.8 ± 2.6	64.0 ± 7.1	16.4 ± 4.4	16.0 ± 2.4	4.1 ± 0.7	2.6 ± 0.1	0.7 ± 0.1	81 ± 2
	50	38.1 ± 2.2	8.7 ± 1.6	254.2 ± 14.8	58.6 ± 10.4	63.6 ± 14.4	14.7 ± 3.6	2.5 ± 0.1	0.6 ± 0.1	83 ± 3
	20	26.6 ± 5.5	11.1 ± 0.7	442.8 ± 91.3	185.2 ± 11.5	110.7 ± 34.9	46.3 ± 11.7	1.8 ± 0.2	0.7 ± 0.1	66 ± 4

Table S8. Summary of photocatalytic control experiment results obtained from (orange shade) DMPC liposomes and (blue shade) homogeneous analogous systems. All experiments were conducted in CO_2- or N_2-saturated 0.1 M $NaHCO_3$ and 0.1 M sodium ascorbate solutions for 4 hours under visible light and at 25 °C, and in triplicates. [Photosensitizer] = 10 μM and [Catalyst] = 500 nM. Symbols "✓" and "-" stand for "included" and "not included" respectively, and "n. d." stands for "not detected". Colour code: alkylated catalysts (red shaded), water-soluble catalysts (blue shaded).

Type of catalyst	Components				Gaseous products after 4-hour experiments				
	Catalyst	Photosensitizer	Sodium ascorbate	CO_2[a]	CO (nmol)	H_2 (nmol)	TON_{CO}	TON_{H2}	CO selectivity (%)[b]
CoP_L	✓	✓	✓	✓	282.9 ± 11.2	54.6 ± 8.9	188.6 ± 7.5	36.4 ± 5.9	84 ± 1
	✓	✓	✓	✓	n.d. [c]	n.d.	-	-	-
	✓	-	✓	✓	n.d.	n.d.	-	-	-
	-	✓	✓	✓	6.1 ± 0.8	5.1 ± 0.1	-	-	54 ± 1
	✓	✓	-	✓	n.d.	n.d.	-	-	-
	✓	✓	✓	-	2.0[d]	2.9	1.3	3.8	27
CoP_W	✓	✓	✓	✓	96.8 ± 1.4	14.5 ± 0.7	64.5 ± 0.9	9.7 ± 0.5	87 ± 1
	-	✓	✓	✓	28.4 ± 7.2	30.5 ± 12.2	-	-	48 ± 2
CoT_L	✓	✓	✓	✓	48.9 ± 18.0	9.8 ± 2.0	32.6 ± 12.0	6.5 ± 1.3	87 ± 2
CoT_W	✓	✓	✓	✓	42.8 ± 9.4	87.3 ± 39.1	28.6 ± 6.3	58.2 ± 26.1	33 ± 11
NiP_L	✓	✓	✓	✓	27.3 ± 3.0	14.6 ± 3.0	18.2 ± 2.6	9.7 ± 2.0	62 ± 6
NiP_W	✓	✓	✓	✓	76.9 ± 4.2	41.9 ± 6.9	51.3 ± 3	28.0 ± 4.6	65 ± 6
NiT_L	✓	✓	✓	✓	24.8 ± 5.0	12.8 ± 4.5	16.5 ± 3.3	8.5 ± 3.0	74 ± 7
NiT_W	✓	✓	✓	✓	11.4 ± 2.1	4.4 ± 0.2	7.6 ± 1.4	3.0 ± 0.2	72 ± 5
FeP_L	✓	✓	✓	✓	26.7 ± 3.8	8.4 ± 1.1	17.8 ± 2.5	5.6 ± 0.7	82 ± 5
FeP_W	✓	✓	✓	✓	33.4 ± 8.0	47.4 ± 22.7	22.3 ± 5.3	31.6 ± 15.2	41 ± 16
FeT_L	✓	✓	✓	✓	51.9 ± 12.5	11.1 ± 2.3	34.6 ± 8.3	7.4 ± 7.4	87 ± 3
FeT_W	✓	✓	✓	✓	27.6 ± 3.1	87.6 ± 14.2	18.4 ± 2.1	58.4 ± 9.4	24 ± 2

[a] Symbol "✓" stands for CO_2-saturated and "-" for N_2-saturated 0.1M $NaHCO_3$ and 0.1 M sodium ascorbate solutions. [b] CO selectivity (%) = n_{CO} / (n_{CO} + n_{H2}) x 100. [c] Experiments were carried out in the dark. [d] Detected CO is likely produced by the reduction of CO_2 coming from $NaHCO_3$.

Table S9. Summary of photocatalytic gaseous products obtained by using different CO_2-saturated buffered solutions [0.1 M $NaHCO_3$ (pH = 6.7) vs 0.1 M NaH_2PO_4 (pH = 6.3)] with DMPC liposomes containing 20:1 **$RuPS_L$:CoP_L** or homogeneous **$RuPS_W$**:**CoP_W**. Liposomes were composed of [DMPC] = 100 µM, [NaDSPE-PEG2K] = 1 µM, [**$RuPS_L$**] = 10 µM and [**CoP_L**] = 20-500 nM, and homogeneous solution contained [**$RuPS_W$**] = 10 µM and [**CoP_W**] = 20-500 nM. All experiments were conducted for four hours under visible light (λ > 400 nm, AM 1.5G, 100 mW cm^{-2}), at 25 °C, and in triplicates (liposomes) or in duplicates (homogeneous).

Catalyst	Catalyst concentration (nm)	Buffer	CO (nmol)	H_2 (nmol)	TON_{CO}	TON_{H2}	CO selectivity (%)[a]
CoP_L	500	$NaHCO_3$	282.9 ± 11.2	54.6±8.9	188.6 ± 7.5	36.4±5.9	84 ± 1
	200		193.5 ± 7.2	30.9±3.1	322.5 ± 12.1	51.4±5.1	86 ± 1
	50		71.5 ± 1.3	18.1±2.4	476.4 ± 8.6	120.5±16.2	82 ± 3
	20		44.1 ± 5.5	13.5±1.4	735.1 ± 91.3	224.7±24.1	78 ± 4
	0		6.1 ± 0.8	5.1 ± 0.1	-	-	54 ± 1
	500	NaH_2PO_4	541.3 ± 18.7	119.7 ± 4.7	360.8 ± 12.5	79.8 ± 3.1	82 ± 1
	200		290.1 ± 5.5	78.4 ± 0.2	483.5 ± 9.1	130.6 ± 0.3	79 ± 1
	50		102.8 ± 1.6	39.3 ± 5.9	685.5 ± 10.9	261.9 ± 39.1	72 ± 4
	20		87.3 ± 2.1	26.0 ± 0.1	1455.5 ± 35.5	433.6 ± 0.5	77 ± 1
	0		40.8 ± 12.2	105.6 ± 93.2	-	-	28 ± 18
CoP_W	500	$NaHCO_3$	96.8 ± 1.4	14.5 ± 0.7	64.5 ± 0.9	9.7 ± 1.0	87 ± 1
	200		62.1 ± 0.3	27.7 ± 1.4	103.5 ± 0.5	46.1 ± 2.3	69 ± 1
	50		64.0 ± 1.0	26.9 ± 1.4	426.7 ± 6.7	179.3 ± 9.2	70 ± 1
	20		31.7 ± 0.2	22.7 ± 0.2	528.8 ± 2.9	379.1 ± 3.2	58 ± 1
	0		28.4 ± 7.2	29.1 ± 12.8	-	-	49 ± 4
	500	NaH_2PO_4	198.8 ± 140.1	97.0 ± 82.9	132.5 ± 93.8	64.7 ± 55.3	73 ± 10
	200		81.6 ± 7.0	114.6 ± 26.5	135.9 ± 11.7	191.0 ± 44.1	42 ± 5
	50		18.1 ± 2.7	175.6 ± 14.8	120.8 ± 17.9	1170.4 ± 98.9	9 ± 1
	20		18.7 ± 1.3	145.5 ± 7.9	312.1 ± 22.4	2425.2 ± 131.2	11 ± 2
	0		6.7 ± 0.6	294.0 ± 31.2	-	-	2 ± 1

[a] CO selectivity (%) = n_{CO} / (n_{CO} + n_{H2}) x 100.

Table S10. Summary of reported molecule- and enzyme-based photocatalytic systems for CO_2 reduction under aqueous conditions.

Catalyst (µM)	Photosensitizer (µM)	Sacrificial donor (M)	CO (µmol)	TON (h)	QY (%)	Solvent	CO Sel. (%)	Reference
Co(*p*TPPS) (0.25)	$CuInS_2$-$SC_2H_4NH_3^+$ (2.5)	NaHAsc (0.025)<br>NaHAsc (0.005) + TCEP (5)	36<br>42	72,484 (96)<br>84,101 (96)	3.39<br>0.96	H_2O	>99	[18]
CODH enzyme (<0.1 nmol)	CdS	MES (0.35)	2	22,500	0.01	MES aq. Buffer	n. r.	[19]
Co(*o*TMpyP) (5)	Cu complex (500)	NaHAsc (0.1)	200	4000 (12)	5.7	$NaHCO_3$ aq. buffer	90	[20]
Co(*p*TMpyP), i.e. CoP_W, (5)	Cu complex (500)	NaHAsc (0.1)	135	2680 (4)	1.6	$NaHCO_3$ aq. buffer	77	[4]
Co(*p*TCPP) (2.5)	$[Ru(bpy)_3]Cl_2$, i.e. $RuPS_W$ (500)	NaHAsc (0.1)	63	2500 (4)	0.24	$NaHCO_3$ aq. Buffer	74	[21]
CODH enzyme (117)	RuP/TiO_2	MES (0.2)	6	2100	n. r.	MES aq. Buffer	n. r.	[22]
CoP_L (0.5)<br>CoP_L (0.02)	$RuPS_L$ (10)	NaHAsc (0.1)	0.28<br>0.09	189 (4)<br>1456 (4)	0.16<br>n. r.	$NaHCO_3$ aq. Buffer<br>NaH_2PO_4 aq. Buffer	84<br>77	This work
Co_2-cryptate (1)	MPA-CdS QD	TEOA (0.3)	34	1380 (120)	n. r.	$NaHCO_3$ aq. buffer	95	[23]
Co(*p*TPPS) (10)	$[Ru(bpy)_3]Cl_2$ (500)	NaHAsc (0.1)	92	926 (4)	0.81	$NaHCO_3$ aq. buffer	82	[24]
Co(TPP-S_3N_1)	ZnSe	NaHAsc ()	18.6	619 (16.7)	5.1	H_2O	>40	[25]
Fe(*p*TMAP) (1)	MPA-$CuInS_2$/ZnS QDs (30)	TEOA	0.22	450 (30)	0.025	KCl aq.	99	[26]
$[Co(tpyP)_2]^{2+}$ (17 nmol cm^{-2})	$SrTiO_3$:La,Rh|Au|RuO_2-BiVO4:Mo	H_2O	6.53 cm^{-2} (formate)	399 (6)	2.6	$KHCO_3$ aq. Buffer	97	[27]
NiCycP (10)	BF_4-ZnSe QDs (0.5)	NaHAsc (0.1)	6	283 (20)	3.4	H_2O	34	[28]
Re(dtb)(CO_3)Cl (40)	Ru(dtb)$(bpy)_2]$ (40)	NaHAsc (0.1)	15	190 (15)	n. r.	Tris-HCl aq.	98	[29]
$Ru(dmb)_2$-(BL)-$Re(CO_3Cl]^{2+}$ (50)		$Bi(CO_2)H$ (0.01)	13	130 (6)	13.5	H_2O	81	[30]
$[Ni(tpyS)_2]^{2+}$ (100)	BF_4-CdS (1)	TEOA (0.1)	4	20 (22)	0.28	H_2O	>90	[14]
$[Ni(cyclam)]^{2+}$ (100)	$[Ru(bpy)_3]^{2+}$ (5000)	NaHAsc (0.5)	3.6	3.6	0.14	H_2O–supercritical CO_2	87	[31]
$[Ni(cyclam)]^{2+}$ (2000)	$[Ru(bpy)_3]^{2+}$ (500)	NaHAsc (1)	n. r.	0.1	n. r.	H_2O	13	[32]

Table S11. Summary of photocatalytic gaseous products and CO selectivity obtained by modifying the concentration of the sacrificial electron donor sodium ascorbate (NaHAsc) with DMPC liposomes containing [DMPC] = 100 µM, [NaDSPE-PEG2K] = 1 µM, [**$RuPS_L$**] = 10 µM and [**CoP_L**] = 500 nM. All experiments were performed in CO_2-saturated 0.1M $NaHCO_3$ solution for four hours (λ > 400 nm, AM 1.5G, 100 mW cm^{-2}) and in duplicates, with the exception of 100 mM sodium ascorbate solution which were performed as triplicates.

[NaHAsc] (mM)	CO (nmol)	H_2 (nmol)	TON_{CO}	TON_{H2}	CO selectivity (%)[a]
0	n.d. [b]	n.d.	-	-	-
50	171.2 ± 32.8	27.2 ± 3.8	114.1 ± 21.9	18.1 ± 2.5	86 ± 1
100	282.9 ± 11.2	51.0 ± 12.7	188.6 ± 7.5	36.4 ± 5.9	84 ± 1
200	327.5 ± 37.6	58.9 ± 6.3	218.4 ± 25.1	39.3 ± 4.2	85 ± 1
400	390.0 ± 18.8	82.0 ± 4.6	260.0 ± 12.5	54.6 ± 3.1	83 ± 1

[a] CO selectivity (%) = n_{CO} / (n_{CO} + n_{H2}) x 100. [b] "n.d." stands for not detected.

Table S12. Summary of photocatalytic gaseous products obtained after two hours by using different light intensities with DMPC liposomes containing [DMPC] = 100 µM, [NaDSPE-PEG2K] = 1 µM, [**$RuPS_L$**] = 10 µM and [**CoP_L**] = 500 nM. All experiments were conducted in CO_2-saturated 0.1M $NaHCO_3$ and 0.1 M sodium ascorbate for two hours under visible light irradiation (λ > 400 nm, AM 1.5G, 100 mW cm^{-2} for light intensity = 100 %) at 25 °C, and in triplicates.

Light intensity (%)	CO (nmol)	H_2 (nmol)	TON_{CO}	TON_{H2}	CO selectivity (%)[a]
100	192.8±9.0	34.8±5.0	128.6±6.0	23.2±3.3	85 ± 1
90	190.2±5.3	35.2±4.2	126.8±3.5	23.5±2.8	84 ± 1
50	82.2±9.9	18.1±3.0	54.8±6.6	12.1±2.0	82 ± 1
20	19.6±1.9	4.9±0.7	13.1±1.2	3.3±0.4	80 ± 2

[a] CO selectivity (%) = n_{CO} / (n_{CO} + n_{H2}) x 100.

Table S13. Summary of photocatalytic gaseous products obtained by using different lipids (DMPC, DLPC, DPPC at 100 µM) and concentrations of DMPC in liposomes, i.e. [DMPC] = 100-400 µM, [NaDSPE-PEG2K] = 1-4 µM, [$\mathbf{RuPS_L}$] = 10 µM and [$\mathbf{CoP_L}$] = 500 nM. All experiments were performed in CO_2-saturated 0.1 M $NaHCO_3$ and 0.1 M sodium ascorbate for four hours under visible light irradiation (λ > 400 nm, AM 1.5G, 100 mW cm^{-2}) in triplicates.

[Lipid] (mM)	Lipid	Tm (°C)[a]	CO (nmol)	H_2 (nmol)	TON_{CO}	TON_{H2}	CO selectivity (%)[b]
100	DLPC	–2	216.8 ± 40.3	49.5 ± 15.0	144.5 ± 26.8	33.0 ± 10.0	81 ± 1
100	DPPC	41	119.0 ± 32.3	14.5 ± 1.6	79.3 ± 21.5	9.7 ± 1.0	89 ± 1
100	DMPC	24	282.9 ± 11.2	51.0 ± 12.7	188.6 ± 7.5	36.4 ± 5.9	84 ± 1
200			389.3± 29.2	95.1 ± 15.5	259.5 ± 19.5	63.4 ± 10.4	81 ± 1
400			347.7 ± 28.6	81.8 ± 17.9	231.8 ± 19.0	12.0	81 ± 1

[a] Provided phase transition temperature values correspond only to liposomes made of exclusively the specified lipid. [b] CO selectivity (%) = n_{CO} / (n_{CO} + n_{H2}) x 100.

Tabulated information from photoinduced charge-transfer dynamics investigations

Table S14. (First row) Bimolecular quenching reaction rate constant (K_q) for **RuPS$_W$** and NaHAsc, and association constant (K_A) for **RuPS$_L$** in DMPC liposomes and NaHAsc. (Second row) Time constants obtained from fitting of emission decay at 650 nm of photoexcited **RuPS$_W$** and **RuPS$_L$** (in various mole ratios with DMPC) in the absence of NaHAsc. (Third row) Time constants and contribution (in %) at 500 nm of photocatalytic system with **NiT$_W$** and **NiT$_L$** in various concentrations. (Fourth row) Electron transfer rates in homogeneous and liposomes. N.B. for further details see Supplementary Note 5 in section above.

**Reductive quenching electron transfer**	
**K_q (M^{-1} s^{-1})**	**K_A (M^{-1})**
3.7 10^7	31

**Charge separation quantum yield**	
Homogeneous Φ_{ET}	35 %
Liposome Φ_{ET}	6 %

**PS-PS self-quenching (in the absence of NaHAsc)**		
**RuPS$_W$**	**τ_1 (ns) / (a_1 %)**	
	600 (100)	
**DMPC/RuPS$_L$ (mole ratios)**	**τ_1 (ns) / (a_1 %)[a]**	**τ_2 (ns) / (a_2 %)[b]**
10:1	8 (85)	251 (15)
20:1	12 (36)	298 (64)
40:1	12 (22)	377 (78)

**Time constants and contributions obtained from kinetic traces**						
**NiT$_W$**			**NiT$_L$**			
**Concentration**	**τ_1 (µs) / (a_1 %)[c]**	**τ_2 (µs) / (a_2 %)**	**Concentration**	**τ_1 (µs) / (a_1 %)**	**τ_2 (µs) / (a_2 %)**	**Offset / (a_3 %)**
0 µM	18.5 (100)	-	0 µM	28 (37)	330 (20)	(43)
100 µM	3.5 (77)	19.0 (23)	0.5 µM	22.8 (53)	195 (20)	(27)
200 µM	2.0 (78)	25 (22)	2 µM	12.2 (67)	100 (20)	(13)
300 µM	1.5 (77)	28 (23)	5 µM	7.4 (57)	80 (25)	(18)

**One-electron reduction rate constants of Liposome-immobilized and water-soluble catalysts**	
**Catalyst**	**K_{ET} (M^{-1} s^{-1})**
**NiT$_L$**	2.00 10^{10}
**NiT$_W$**	2.16 10^9
**CoP$_W$**	1.31 10^{10}
**CoT$_W$**	2.67 10^9
**NiP$_W$**	2.00 10^{10}
**FeT$_W$**	2.30 10^9
**FeP$_W$**	3.00 10^{10}

a_1 % and a_2 % stand for amplitude of data fitting in %. a_3 stands for the offset amplitude of very long-live components. [a] percent contribution of the short lifetime, obtained from a biexponential decay function, and associated with self-quenching processes. [b] percent contribution of the long lifetime, obtained from a biexponential decay function, and associated with normal lifetime of photoexcited Ru(II). [c] percent contribution of short-lifetime components related to electron transfer rates with different catalyst concentrations.

Electrochemistry results of electrode-immobilized catalysts in aqueous conditions

Table S15. Summary of results obtained from four hours of chronoamperometry of GCE with different alkylated molecular catalysts and FTO electrodes with **CoP$_L$** at an applied potential of −0.9 V vs SHE. Measurements were carried out in CO_2-saturated 0.1 M $NaHCO_3$ solutions (pH = 6.7). All experiments were performed in triplicates and the average metrics are presented. Observed faradaic yield values are consistent with reported values for analogous homogeneous catalysts[13].

Catalysts	Gaseous products (µmol cm^{-2})		CO Selectivity (%)[a]	Charge passed (C cm^{-2})	Faradaic efficiency (FE, %)		
	CO	H_2			FE_{CO}	FE_{H2}	FE_{CO+H2}
GCE|**CoP$_L$**	0.16 ± 0.04	0.06 ± 0.02	74 ± 4	0.07 ± 0.02	50.8 ± 13.4	17.0 ± 1.1	67.8 ± 14.5
FTO|**CoP$_L$**	0.07 ± 0.03	0.54 ± 0.57	11 ± 5	0.23 ± 0.12	2.5 ± 1.4	42.7 ± 27.5	45.2 ± 28.2
GCE|**CoT$_L$**	0.04 ± 0.01	0.14 ± 0.11	25 ± 9	0.09 ± 0.02	7.4 ± 0.6	26.7 ± 16.6	34.1 ± 16.9
GCE|**NiP$_L$**	0.02 ± 0.01	0.22 ± 0.11	12 ± 9	0.08 ± 0.03	3.6 ± 2.9	50.5 ± 13.3	54.1 ± 10.8
GCE|**NiT$_L$**	0.02 ± 0.01	0.99 ± 0.34	2 ± 1	0.35 ± 0.15	0.9 ± 0.3	55.6 ± 6.1	56.5 ± 6.2
GCE|**FeP$_L$**	0.03 ± 0.02	0.08 ± 0.04	30 ± 9	0.09 ± 0.08	8.0 ± 3.0	19.4 ± 6.1	27.4 ± 6.6
GCE|**FeT$_L$**	0.01 ± 0.01	0.13 ± 0.08	12 ± 10	0.11 ± 0.08	3.6 ± 2.8	25.4 ± 9.3	29.0 ± 9.8
Bare GCE [b]	n. d.[c]	0.58 ± 0.52	-	0.16 ± 0.12	-	65.0 ± 10.2	65.0 ± 10.2
Bare FTO	0.01 ± 0.01	0.77 ± 0.14	1 ± 1	0.25 ± 0.04	0.6 ± 0.3	60.8 ± 16.5	61.4 ± 16.7

[a] CO selectivity (%) = n_{CO} / (n_{CO} + n_{H2}) x 100. [b] CO was not detected in the headspace gas when no alkylated catalyst was drop-casted on the GCE electrode. [c] "n.d." stands for not detected.

Summary of experimental and calculated resonance Raman data

Table S16. Summary of experimental resonance Raman peaks. For summary of atom labeling see below Scheme S5.

This work						Terekhov[11]		Assignment[c]
FTO|**CoP**$_L$ in CO_2-saturated 0.1 M $NaHCO_3$						**Co^{II}P$_W$** [a]	**Co^{III}P$_W$** [b]	
**+0.7 V**	**–0.1 V**	**–0.3 V**	**–0.5**	**–0.7**	**–0.9**			
1635	1635	1636	1638	1634	1635	1643	1644	v(pyr), A_{1g}
1599	1599	1599	1599				1597	v($C_\beta C_\beta$ + $C_\alpha C_m$), B_{1g}
1575	1573	1574	1575			1573	1579	v($C_\beta C_\beta$ + $C_\alpha C_m$), A_{1g}
1508			1506	1510	1510		1516	v($C_\beta C_\beta$),B_{1g}
			1471	1470	1472	1467	1475	v_s($C_\alpha C_\beta$), A_{1g}
				1382	1383		1372	v_s(C_αN), A_{1g}
1363	1362	1360	1362	1369	1366	1367		v_s(C_αN), A_{1g}
1342	1337	1336	1332	1323	1323			τ(CH_2) / δ(CH_3)[11]
		1240	1240			1253	1257	v(C_m-pyr), A_{1g}
1217	1218	1218	1218	1213	1211	1220	1221	δ(pyr), A_{1g}
				1194	1195	1192	1192	δ(pyr), v(N^+-CH_3/alkyl)
1092	1091	1092	1092	1090	1087	1098	1101	$δ_s$(C_βH), A_{1g}
						1057	1057	pyr δ(CH), A_{1g}
1007	1007	1007	1007	1007	1007	1008(sh)	1008	v_s($C_\alpha C_m$), A_{1g}
990(sh)	990(sh)	990(sh)	990(sh)			998		v_s($C_\alpha C_m$), A_{1g}
908	906	906	907	901	900	906	907	$δ_s$(por), A_{1g}
667	662	662	664	667	667		665	δ(pyr) + δ(C-N^+-CH_3/alkyl), A_{1g}

[a] measured in water at pH 7. [b] measured in water at pH 8. [c] Assignments based on literature.[11] Por = porphyrin ring, pyr = N-pyridinium ring, sh = shoulder, v = stretching, δ = bending, τ = twisting, s = symmetric (subscript).

Table S17. Selection of experimental frequencies (cm^{-1}) of FTO|**CoP_L** at +0.7 and –0.9 V vs SHE in CO_2-saturated 0.1 M $NaHCO_3$ and calculated frequencies for **$[CoP]^{4+}$**, **$[CoP(H_2O)]^{4+}$**, **$[CoP(H_2O)]^0$** and **$[CoP(CO)]^0$** and their proposed assigned vibrational modes. Experimental and calculated peaks are shown in Figures S44-46. The proposed assignments are based on calculated vibrational modes for each calculated structure for the selected Raman peaks. For summary of atom labeling see Scheme S5. N.B. for further details see Supplementary Note 7 in section above.

Experimental		Calculated			Proposed assignment	
FTO|**CoP_L**						
**+0.7 V**	**–0.9 V**	**$[CoP]^{4+}$**	**$[CoP(H_2O)]^{4+}$**	**$[CoP(H_2O)]^0$**	**$[CoP(CO)]^0$**	
-	1007	-	-	981, 984, 988, 992	981, 984, 989, 991	δ_s(pyr)
				998, 1000, 1001, 1003, 1008, 1016, 1018, 1022	997, 1000, 1001, 1004, 1008, 1018, 1019, 1024	δ(pyr), δ(Por)
				1034, 1035,	1036, 1037,	δ_{as}(pyr), r(N^+-CH_3)
				1038, 1040, 1042, 1044,	1039, 1040, 1043, 1045,	r(N^+-CH_3), δ_s(C_βH)
				1056, 1058	1058, 1058	δ_s(C_βH)
1599	-	1622, 1623, 1623, 1625	1623, 1623, 1623, 1626	-	-	v_s(pyr)

Por = porphyrin ring, pyr = N-pyridinium ring, v = stretching, δ = bending, r = rocking, s = symmetric (subscript), as = asymmetric (subscript).

Tabulated Gibbs free energy of all calculated species

Table S18. Gibbs free energy of the calculated cobalt porphyrin complexes **CoP**, organized by the different co-absorbed ligands and at distinct oxidation states, with respect to [**CoP**]$^{+4}$. Electron transfer energies were referenced by the $[Ru(bpy)_3]^{2+}/[Ru(bpy)_3]^{1+}$ redox cycle and proton transfer energies were calculated from the free energy of a free proton in H_2O (–272.2 kcal mol^{-1}).[33-35] N.B. for further details see Supplementary Note 7 in section above.

**Compound**	**ΔG [Kcal mol^{-1}]**
[**CoP**]$^{5+}$	62.7
[**CoP**]$^{4+}$	0.0
[**CoP**]$^{3+}$	–14.6
[**CoP**]$^{2+}$	–26.3
[**CoP**]$^{1+}$	–37.1
[**CoP**]^{0}	–39.8
[**CoP**]$^{1-}$	–38.8
[**CoP**]$^{2-}$	–30.8

[**CoP**(CO_2)]$^{3+}$	1.2
[**CoP**(CO_2)]$^{2+}$	–6.7
[**CoP**(CO_2)]$^{1+}$	–22.9
[**CoP**(CO_2)]^{0}	–26.6
[**CoP**(CO_2)]$^{1-}$	–28.4
[**CoP**(CO_2)]$^{2-}$	–23.9

[**CoP**(H_2O)]$^{5+}$	56.8
[**CoP**(H_2O)]$^{4+}$	–3.2
[**CoP**(H_2O)]$^{3+}$	–12.5
[**CoP**(H_2O)]$^{2+}$	–31.8
[**CoP**(H_2O)]$^{1+}$	–37.8
[**CoP**(H_2O)]^{0}	–42.3
[**CoP**(H_2O)]$^{1-}$	–45.4

[**CoP**(COOH)]$^{4+}$	-0.3
[**CoP**(COOH)]$^{3+}$	–14.1
[**CoP**(COOH)]$^{2+}$	–37.1
[**CoP**(COOH)]$^{1+}$	–44.9
[**CoP**(COOH)]^{0}	–49.7
[**CoP**(COOH)]$^{1-}$	–43.5

[**CoP**(CO)]$^{4+}$	–43.6
[**CoP**(CO)]$^{2+}$	–69.7
[**CoP**(CO)]$^{1+}$	–75.3
[**CoP**(CO)]^{0}	–79.7
[**CoP**(CO)]$^{1-}$	–80.4

[**CoP**(H)]$^{4+}$	13.2
[**CoP**(H)]$^{3+}$	–20.1
[**CoP**(H)]$^{2+}$	–38.3
[**CoP**(H)]$^{1+}$	–45.5
[**CoP**(H)]$^{0+}$	–48.6
[**CoP**(H)]$^{1-}$	–50.8

Table S19. Gibbs free energy of the calculated cobalt porphyrin complexes **CoP**, at different oxidation state and containing different co-absorbed ligands, with respect to [$\mathbf{CoP_L}$]$^{+4}$. Electron transfer energies were referenced by the [Ru(bpy)$_3$]$^{2+}$/[Ru(bpy)$_3$]$^{1+}$ redox cycle and proton transfer energies were calculated from the free energy of a free proton in H_2O (–272.2 kcal mol^{-1}).[33-35]

Compound	ΔG [Kcal mol^{-1}]
[**CoP**]$^{5+}$	62.7
[**CoP**(H_2O)]$^{5+}$	56.8
[**CoP**]$^{4+}$	0.0
[**CoP**(H_2O)]$^{4+}$	–3.2
[**CoP**]$^{3+}$	–14.6
[**CoP**(H_2O)]$^{3+}$	–12.5
[**CoP**(CO_2)]$^{3+}$	1.2
[**CoP**(COOH)]$^{4+}$	–0.3
[**CoP**(H)]$^{4+}$	13.2
[**CoP**]$^{2+}$	–26.3
[**CoP**(H_2O)]$^{2+}$	–31.8
[**CoP**(CO_2)]$^{2+}$	–6.7
[**CoP**(COOH)]$^{3+}$	–14.1
[**CoP**(CO)]$^{4+}$	–43.6
[**CoP**(H)]$^{3+}$	–20.1
[**CoP**]$^{1+}$	–37.1
[**CoP**(H_2O)]$^{1+}$	–37.8
[**CoP**(CO_2)]$^{1+}$	–22.9
[**CoP**(COOH)]$^{2+}$	–37.1
[**CoP**(H)]$^{2+}$	–38.3
[**CoP**]^{0}	–39.8
[**CoP**(H_2O)]^{0}	–42.3
[**CoP**(CO_2)]^{0}	–26.6
[**CoP**(COOH)]$^{1+}$	–44.9
[**CoP**(CO)]$^{2+}$	–69.7
[**CoP**(H)]$^{1+}$	–45.5
[**CoP**]$^{1-}$	–38.8
[**CoP**(H_2O)]$^{1-}$	–45.4
[**CoP**(CO_2)]$^{1-}$	–28.4
[**CoP**(COOH)]^{0}	–49.7
[**CoP**(CO)]$^{1+}$	–75.3
[**CoP**(H)]^{0}	–48.6
[**CoP**]$^{2-}$	–30.8
[**CoP**(CO_2)]$^{2-}$	–23.9
[**CoP**(COOH)]$^{1-}$	–43.5
[**CoP**(CO)]^{0}	–79.7
[**CoP**(H)]$^{1-}$	–50.8
[**CoP**(CO)]$^{1-}$	–80.4

Table S20. Electronic energies and Gibbs free energy corrections of small molecules, $[Ru(bpy)_3]^{2+/1+}$ and cobalt porphyrin complexes (based on computed **CoP** structures). Different spin states were calculated with M = 2S + 1 with S being the number of unpaired electrons. Energies are given in Hartree atomic units.

Compound	M	6-31G*			6-311++G(3df,3pd)	
		G Corr. [a.u.]	G [a.u.]	G [a.u.] (1M)	Eel [a.u.]	G [a.u.] (1M)
$[Ru(bpy)_3]^{+1}$	2	0.419876	–1580.764845	–1580.761833	–1581.642759	–1581.219871
$[Ru(bpy)_3]^{+2}$	1	0.42648	–1580.648686	–1580.645674	–1581.531323	–1581.101831
CO_2	1	–0.009236	–188.599743	–188.5967311	–188.6627292	–188.6689533
CO	1	–0.014111	–113.32173	–113.3187181	–113.3571505	–113.3682496
H_2O	1	0.002667	–76.427248	–76.42423609	–76.4711115	–76.46543259
[**CoP**]$^{+5}$	1	0.639596	–2280.92664	–2280.923628	–2282.253796	–2281.611188
[**CoP**]$^{+5}$	3	0.63777	–2280.928838	–2280.925826	–2282.254029	–2281.613247
[**CoP**]$^{+5}$	5	0.628689	–2280.870717	–2280.867705	–2282.186365	–2281.554664
[**CoP**]$^{+4}$	2	0.639532	–2281.14158	–2281.138568	–2282.473743	–2281.831199
[**CoP**]$^{+4}$	4	0.634925	–2281.086733	–2281.083721	–2282.412466	–2281.77453
[**CoP**]$^{+3}$	1	0.640622	–2281.230188	–2281.227176	–2282.56636	–2281.922726
[**CoP**]$^{+3}$	3	0.638331	–2281.277746	–2281.274734	–2282.613901	–2281.972558
[**CoP**]$^{+3}$	5	0.635541	–2281.229048	–2281.226036	–2282.561084	–2281.922531
[**CoP**]$^{+2}$	2	0.637201	–2281.406607	–2281.403595	–2282.749417	–2282.109204
[**CoP**]$^{+2}$	4	0.635189	–2281.405818	–2281.402806	–2282.745271	–2282.107071
[**CoP**]$^{+2}$	6	0.632136	–2281.361726	–2281.358714	–2282.695844	–2282.060696
[**CoP**]$^{+2}$	8	0.628564	–2281.360361	–2281.357349	–2282.690377	–2282.058801
[**CoP**]$^{+1}$	1	0.633737	–2281.533837	–2281.530825	–2282.880132	–2282.243383
[**CoP**]$^{+1}$	3	0.632564	–2281.534883	–2281.531871	–2282.880022	–2282.244446
[**CoP**]$^{+1}$	5	0.631462	–2281.524856	–2281.521844	–2282.866913	–2282.232439
[**CoP**]^{0}	2	0.630162	–2281.646589	–2281.643577	–2282.997533	–2282.36436
[**CoP**]^{0}	4	0.629637	–2281.6469	–2281.643888	–2282.997363	–2282.364714
[**CoP**]^{0}	6	0.626205	–2281.649252	–2281.64624	–2282.995991	–2282.366774
[**CoP**]^{0}	8	0.619691	–2281.603781	–2281.600769	–2282.946054	–2282.323351
[**CoP**]$^{-1}$	1	0.629284	–2281.753873	–2281.750861	–2283.115455	–2282.483159
[**CoP**]$^{-1}$	3	0.6246	–2281.744998	–2281.741986	–2283.10158	–2282.473968

[**CoP**]$^{-1}$	5	0.624134	–2281.745541	–2281.742529	–2283.101667	–2282.474521
[**CoP**]$^{-2}$	2	0.62087	–2281.792174	–2281.789162	–2283.158744	–2282.534862
[**CoP**]$^{-2}$	4	0.621241	–2281.823866	–2281.820854	–2283.191842	–2282.567589
[**CoP**]$^{-2}$_2[a]	4	0.623083	–2281.841033	–2281.838021	–2283.204081	–2282.577987
[**CoP**]$^{-2}$_3[a]	4	0.621719	–2281.847494	–2281.844482	–2283.213291	–2282.58856
[**CoP**]$^{-2}$	6	0.613778	–2281.791201	–2281.788189	–2283.154059	–2282.537269
[**CoP**]$^{-2}$_2[a]	6	0.611804	–2281.776743	–2281.773731	–2283.134612	–2282.519796
[**CoP**]$^{-2}$	8	0.612877	–2281.781341	–2281.778329	–2283.140792	–2282.524903
[**CoP**]$^{-2}$_2[a]	8	0.613056	–2281.786812	–2281.7838	–2283.14984	–2282.533772
[**CoP**(CO_2)]$^{+3}$	1	0.645536	–2469.841898	–2469.838886	–2471.264788	–2470.61624
[**CoP**(CO_2)]$^{+3}$_2[a]	1	0.646858	–2469.840583	–2469.837571	–2471.264831	–2470.614961
[**CoP**(CO_2)]$^{+2}$	2	0.643547	–2469.966783	–2469.963771	–2471.393515	–2470.746956
[**CoP**(CO_2)]$^{+2}$	4	0.641631	–2469.914513	–2469.911501	–2471.337056	–2470.692413
[**CoP**(CO_2)]$^{+1}$	1	0.643738	–2470.100563	–2470.097551	–2471.537546	–2470.890797
[**CoP**(CO_2)]$^{+1}$	3	0.642296	–2470.087598	–2470.084586	–2471.520579	–2470.875271
[**CoP**(CO_2)]^{0}	2	0.639432	–2470.2161	–2470.213088	–2471.657141	–2471.014697
[**CoP**(CO_2)]^{0}	4	0.63721	–2470.208676	–2470.205664	–2471.64484	–2471.004618
[**CoP**(CO_2)]^{0}	6	0.633406	–2470.201519	–2470.198507	–2471.628213	–2470.991795
[**CoP**(CO_2)]$^{-1}$	1	0.636746	–2470.328067	–2470.325055	–2471.774966	–2471.135208
[**CoP**(CO_2)]$^{-1}$	3	0.636748	–2470.327685	–2470.324673	–2471.775309	–2471.135549
[**CoP**(CO_2)]$^{-1}$	5	0.63267	–2470.283968	–2470.280956	–2471.729099	–2471.093417
[**CoP**(CO2)]$^{-2}$	2	0.628668	–2470.411252	–2470.40824	–2471.854687	–2471.223007
[**CoP**(CO_2)]$^{-2}$_2[a]	2	0.636852	–2470.425713	–2470.422701	–2471.886344	–2471.24648
[**CoP**(CO_2)]$^{-2}$	4	0.627911	–2470.412578	–2470.409566	–2471.855202	–2471.224279
[**CoP**(CO_2)]$^{-2}$_2[a]	4	0.630105	–2470.396288	–2470.393276	–2471.852618	–2471.219501
[**CoP**(CO_2)]$^{-2}$	6	0.625235	–2470.354505	–2470.351493	–2471.807457	–2471.17921
[**CoP**(H_2O)]$^{+5}$	1	0.666432	–2357.34183	–2357.338818	–2358.741449	–2358.072005
[**CoP**(H_2O)]$^{+5}$	3	0.663862	–2357.35696	–2357.353948	–2358.755023	–2358.088149
[**CoP**(H_2O)]$^{+4}$	2	0.659701	–2357.567227	–2357.564215	–2358.964467	–2358.301754
[**CoP**(H_2O)]$^{+4}$	4	0.657861	–2357.507223	–2357.504211	–2358.902343	–2358.241471
[**CoP**(H_2O)]$^{+3}$	1	0.662225	–2357.644018	–2357.641006	–2359.048726	–2358.383489
[**CoP**(H_2O)]$^{+3}$	3	0.659132	–2357.695277	–2357.692265	–2359.096754	–2358.43461

[**CoP**(H_2O)]$^{+3}$	5	0.656354	–2357.646604	–2357.643592	–2359.043866	–2358.3845
[**CoP**(H_2O)]$^{+2}$	2	0.658526	–2357.835681	–2357.832669	–2359.244953	–2358.583415
[**CoP**(H_2O)]$^{+2}$	4	0.655717	–2357.820999	–2357.817987	–2359.225629	–2358.566901
[**CoP**(H_2O)]$^{+2}$	6	0.652758	–2357.784473	–2357.781461	–2359.184191	–2358.528421
[**CoP**(H_2O)]$^{+1}$	1	0.654963	–2357.954881	–2357.951869	–2359.367891	–2358.709916
[**CoP**(H_2O)]$^{+1}$	3	0.653946	–2357.955913	–2357.952901	–2359.367897	–2358.710939
[**CoP**(H_2O)]$^{+1}$	5	0.651049	–2357.947967	–2357.944955	–2359.355105	–2358.701045
[**CoP**(H_2O)]^{0}	2	0.650292	–2358.071796	–2358.068784	–2359.488601	–2358.835297
[**CoP**(H_2O)]^{0}	4	0.649778	–2358.072633	–2358.069621	–2359.488919	–2358.836129
[**CoP**(H_2O)]^{0}_2[a]	4	0.649756	–2358.072166	–2358.069154	–2359.488483	–2358.835715
[**CoP**(H_2O)]^{0}	8	0.642383	–2358.02003	–2358.017018	–2359.428112	–2358.782717
[**CoP**(H_2O)]$^{-1}$	1	0.648963	–2358.181713	–2358.178701	–2359.610198	–2358.958223
[**CoP**(H_2O)]$^{-1}$	3	0.647951	–2358.182717	–2358.179705	–2359.610192	–2358.959229
[**CoP**(COOH)]$^{+4}$	1	0.660773	–2470.280545	–2470.277533	–2471.716236	–2471.052451
[**CoP**(COOH)]$^{+4}$	3	0.658585	–2470.219866	–2470.216854	–2471.649981	–2470.988384
[**CoP**(COOH)]$^{+3}$	2	0.658449	–2470.416878	–2470.413866	–2471.853905	–2471.192444
[**CoP**(COOH)]$^{+3}$	4	0.656098	–2470.362741	–2470.359729	–2471.795654	–2471.136544
[**CoP**(COOH)]$^{+2}$	1	0.656065	–2470.562898	–2470.559886	–2472.006141	–2471.347064
[**CoP**(COOH)]$^{+2}$	3	0.656393	–2470.549653	–2470.546641	–2471.990896	–2471.331492
[**CoP**(COOH)]$^{+2}$	5	0.652261	–2470.509375	–2470.506363	–2471.944745	–2471.289472
[**CoP**(COOH)]$^{+1}$	2	0.654419	–2470.686402	–2470.68339	–2472.135062	–2471.477631
[**CoP**(COOH)]$^{+1}$	4	0.653413	–2470.674846	–2470.671834	–2472.12036	–2471.463935
[**CoP**(COOH)]$^{+1}$	6	0.6473	–2470.663133	–2470.660121	–2472.103481	–2471.453169
[**CoP**(COOH)]^{0}	1	0.65222	–2470.802369	–2470.799357	–2472.257064	–2471.601832
[**CoP**(COOH)]^{0}	3	0.651219	–2470.803751	–2470.800739	–2472.257498	–2471.603267
[**CoP**(COOH)]^{0}	5	0.647441	–2470.776928	–2470.773916	–2472.22882	–2471.578368
[**CoP**(COOH)]$^{-1}$	4	0.644418	–2470.898515	–2470.895503	–2472.358943	–2471.711513
[**CoP**(COOH)]$^{-1}$	6	0.640929	–2470.852063	–2470.849051	–2472.310942	–2471.667001
[**CoP**(CO)]$^{+4}$	2	0.637374	–2394.473629	–2394.470617	–2395.84816	–2395.207774
[**CoP**(CO)]$^{+4}$	4	0.633871	–2394.414836	–2394.411824	–2395.785509	–2395.148626

[**CoP**(CO)]$^{+2}$	2	0.638144	–2394.738828	–2394.735816	–2396.126662	–2395.485506
[**CoP**(CO)]$^{+2}$	4	0.634987	–2394.727108	–2394.724096	–2396.109659	–2395.47166

[**CoP**(CO)]$^{+1}$	1	0.636352	–2394.85805	–2394.855038	–2396.251169	–2395.611805
[**CoP**(CO)]$^{+1}$	3	0.635028	–2394.858741	–2394.855729	–2396.250494	–2395.612454
[**CoP**(CO)]$^{+1}$	5	0.633873	–2394.849687	–2394.846675	–2396.238339	–2395.601455

[**CoP**(CO)]^{0}	2	0.632215	–2394.974691	–2394.971679	–2396.371925	–2395.736698
[**CoP**(CO)]^{0}	4	0.631586	–2394.975461	–2394.972449	–2396.372076	–2395.737478
[**CoP**(CO)]^{0}	6	0.626359	–2394.956901	–2394.953889	–2396.346913	–2395.717542

[**CoP**(CO)]$^{-1}$	1	0.632256	–2395.081784	–2395.078772	–2396.490721	–2395.855453
[**CoP**(CO)]$^{-1}$	3	0.63124	–2395.082887	–2395.079875	–2396.490809	–2395.856557

[**CoP**(H)]$^{+4}$	1	0.650552	–2281.670440	–2281.667428	–2283.015501	–2282.361937
[**CoP**(H)]$^{+4}$	3	0.647395	–2281.638705	–2281.635693	–2282.980652	–2282.330246
[**CoP**(H)]$^{+4}$	5	0.64352	–2281.581998	–2281.578986	–2282.919091	–2282.272559
[**CoP**(H)]$^{+3}$	2	0.645288	–2281.834992	–2281.831980	–2283.181287	–2282.532987
[**CoP**(H)]$^{+3}$	4	0.64458	–2281.783498	–2281.780486	–2283.127742	–2282.48015
[**CoP**(H)]$^{+3}$	6	0.641274	–2281.733393	–2281.730381	–2283.073289	–2282.429003
[**CoP**(H)]$^{+2}$	1	0.645945	–2281.973993	–2281.970981	–2283.329012	–2282.680055
[**CoP**(H)]$^{+2}$	3	0.644731	–2281.960290	–2281.957278	–2283.311964	–2282.66422
[**CoP**(H)]$^{+2}$	5	0.640024	–2281.923331	–2281.920319	–2283.268826	–2282.62579
[**CoP**(H)]$^{+1}$	2	0.640821	–2282.096416	–2282.093404	–2283.453383	–2282.80955
[**CoP**(H)]$^{+1}$	4	0.640796	–2282.086124	–2282.083112	–2283.44105	–2282.797242
[**CoP**(H)]^{0}	1	0.641021	–2282.207833	–2282.204821	–2283.575154	–2282.931121
[**CoP**(H)]^{0}	3	0.638536	–2282.211110	–2282.208098	–2283.574071	–2282.932523
[**CoP**(H)]^{0}	5	0.635801	–2282.182015	–2282.179003	–2283.536532	–2282.89772
[**CoP**(H)]$^{-1}$	2	0.636838	–2282.321057	–2282.318045	–2283.694025	–2283.054175
[**CoP**(H)]$^{-1}$	4	0.632433	–2282.303103	–2282.300091	–2283.664712	–2283.029267
[**CoP**(H)]$^{-1}$	6	0.627017	–2282.245617	–2282.242605	–2283.611417	–2282.981388

[a] different conformer of the same system with the same charge and spin.

Supplementary Schemes

Preparation of liposomes

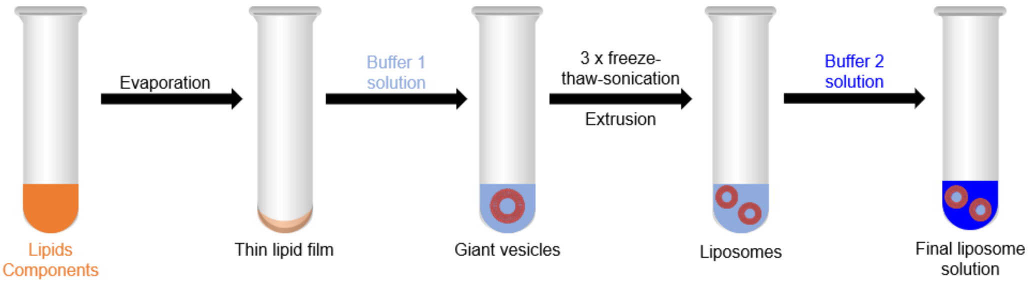

Scheme S1. Scheme of preparation of liposomes.

Synthesis of ligands, catalysts and photosensitizer

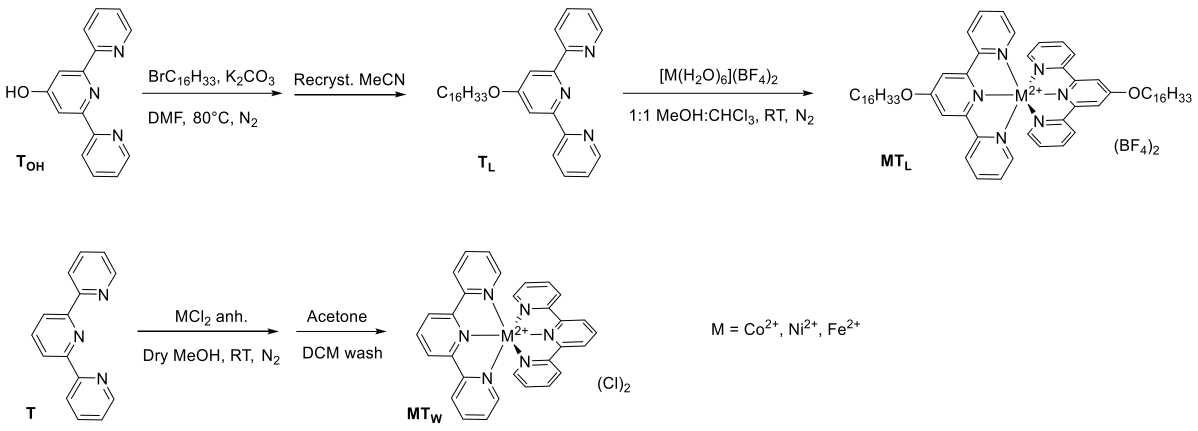

Scheme S2. Synthetic procedure to terpyridine catalysts **MT**$_L$ and **MT**$_W$ (M = Co^{2+}, Ni^{2+} or Fe^{2+}).

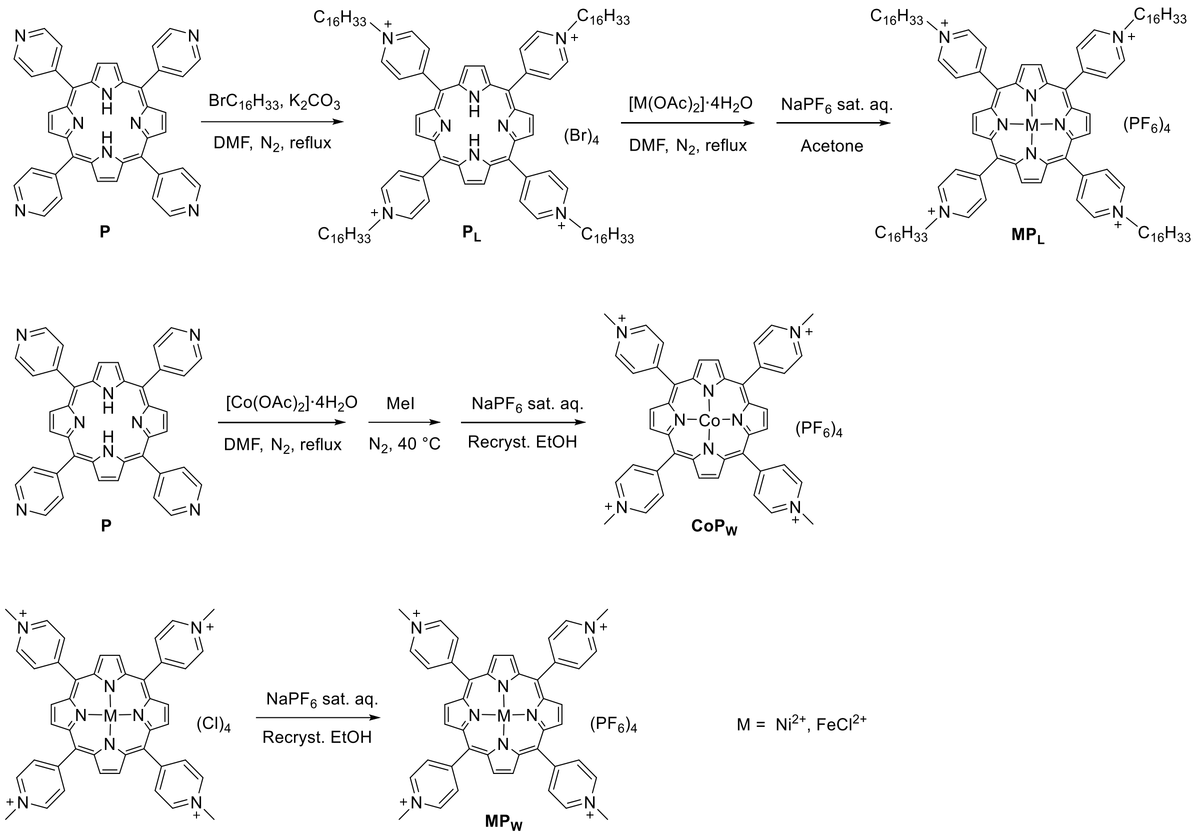

Scheme S3. Synthetic procedure to porphyrin catalysts **MP$_L$** and **MP$_W$** (M = Co^{2+}, Ni^{2+} or $FeCl^{2+}$).

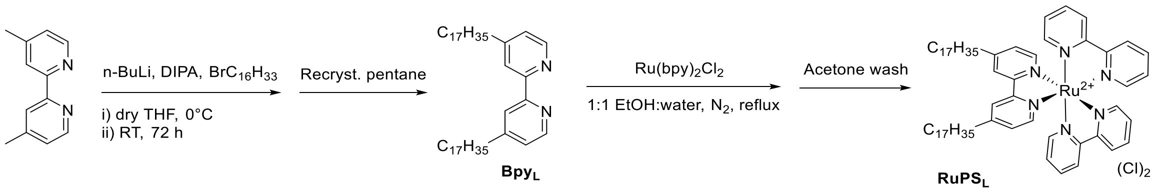

Scheme S4. Synthetic procedure to alkylated ruthenium bipyridine photosensitizer **RuPS$_L$**.

Summary of atom labeling for Raman assignments of the cobalt porphyrin

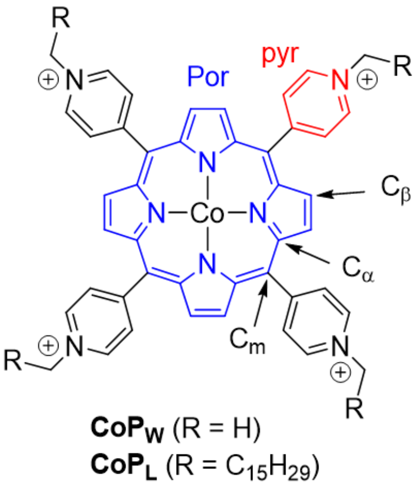

Scheme S5. Simplified structure of studied cobalt porphyrins with summary of atom labeling for Raman assignments. C_α and C_β correspond to pyrrole carbon atoms, while C_m stands for methine carbon atom. (Blue) Por = porphyrin ring, (red) Pyr = N-pyridinium ring.

Supplementary Figures

NMR spectroscopy of prepared ligands and $RuPS_L$

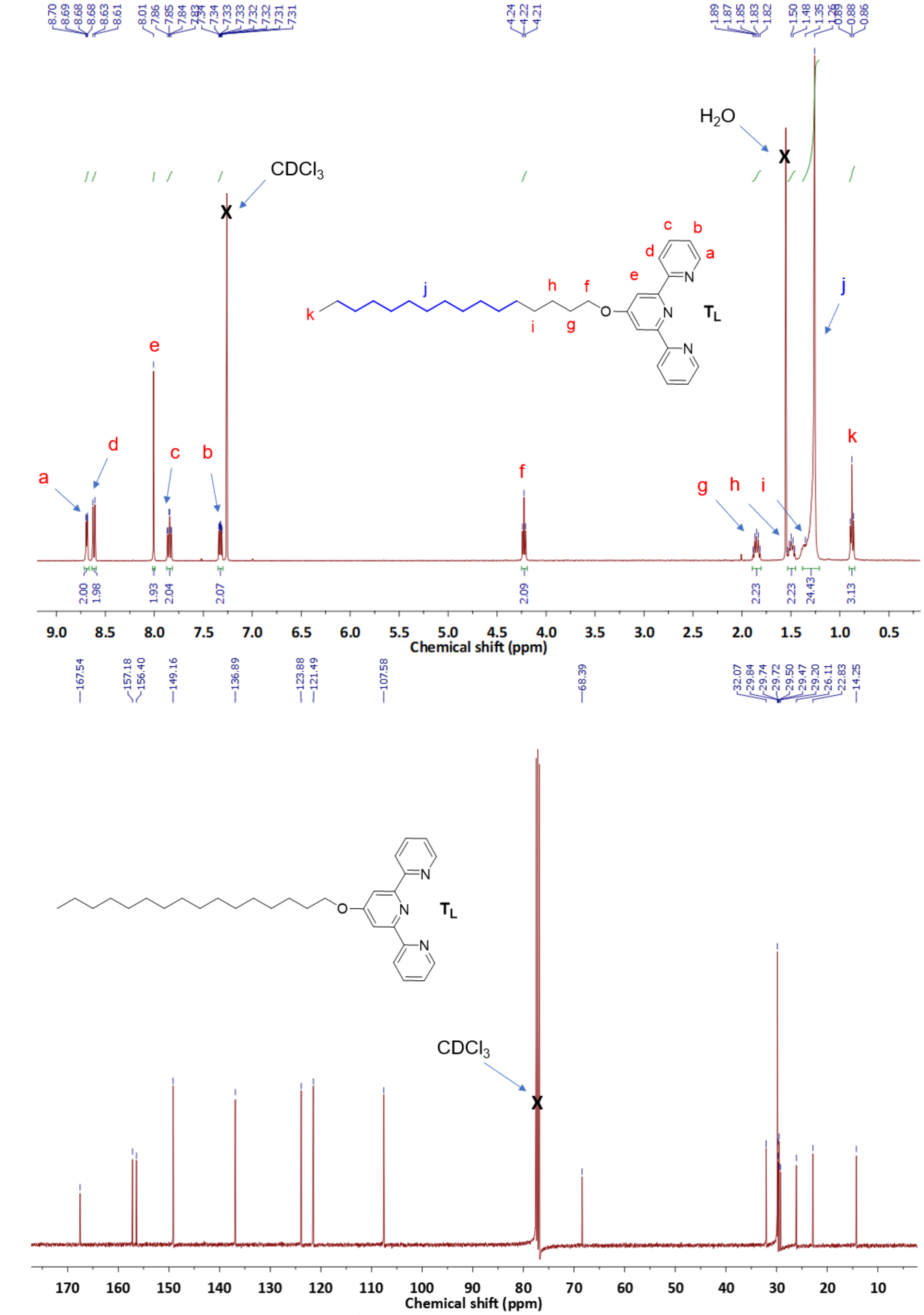

Figure S1. (Top) ^{1}H and (bottom) ^{13}C NMR spectra of alkylated terpyridine ligand $\mathbf{T_L}$ in $CDCl_3$.

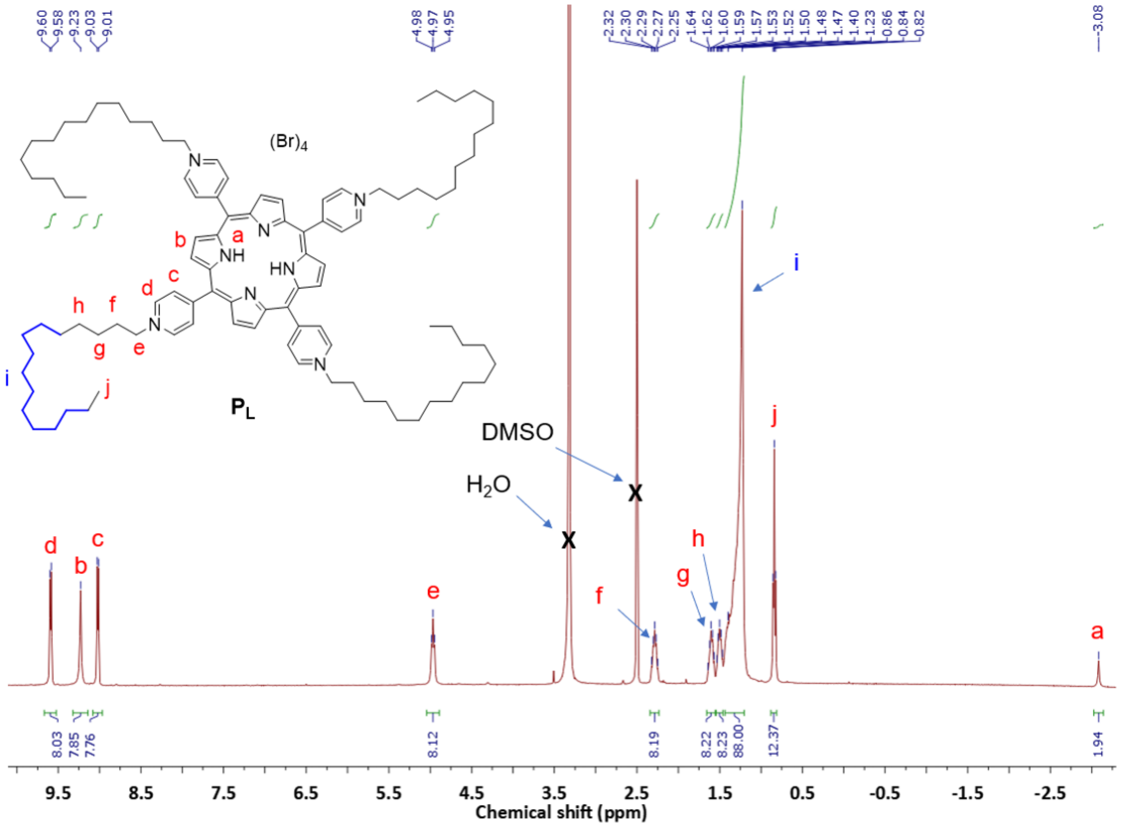

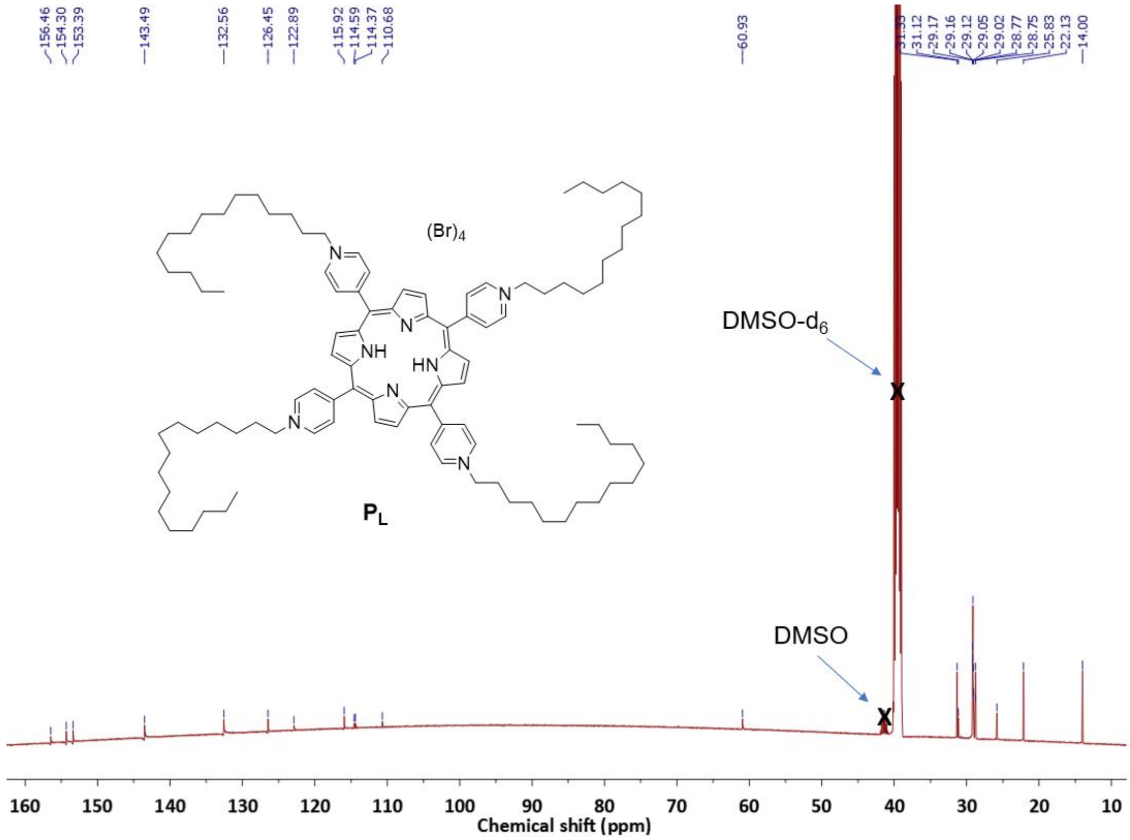

Figure S2. (Top) ^{1}H and (bottom) ^{13}C NMR spectra of alkylated porphyrin ligand $\mathbf{P_L}$ in DMSO-d_6.

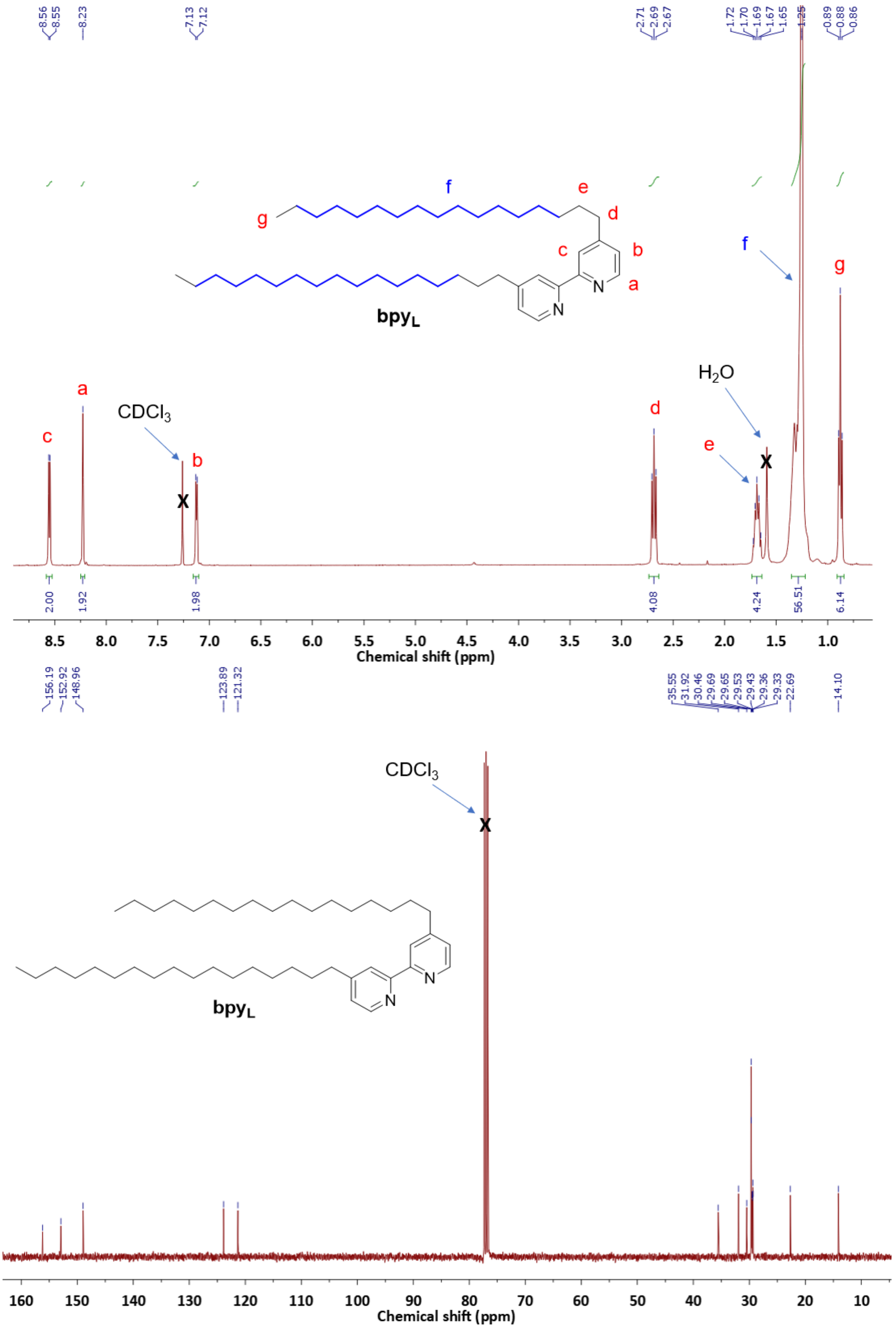

Figure S3. (Top) ^{1}H and (bottom) ^{13}C NMR spectra of alkylated bipyridine ligand **bpy$_L$** in $CDCl_3$.

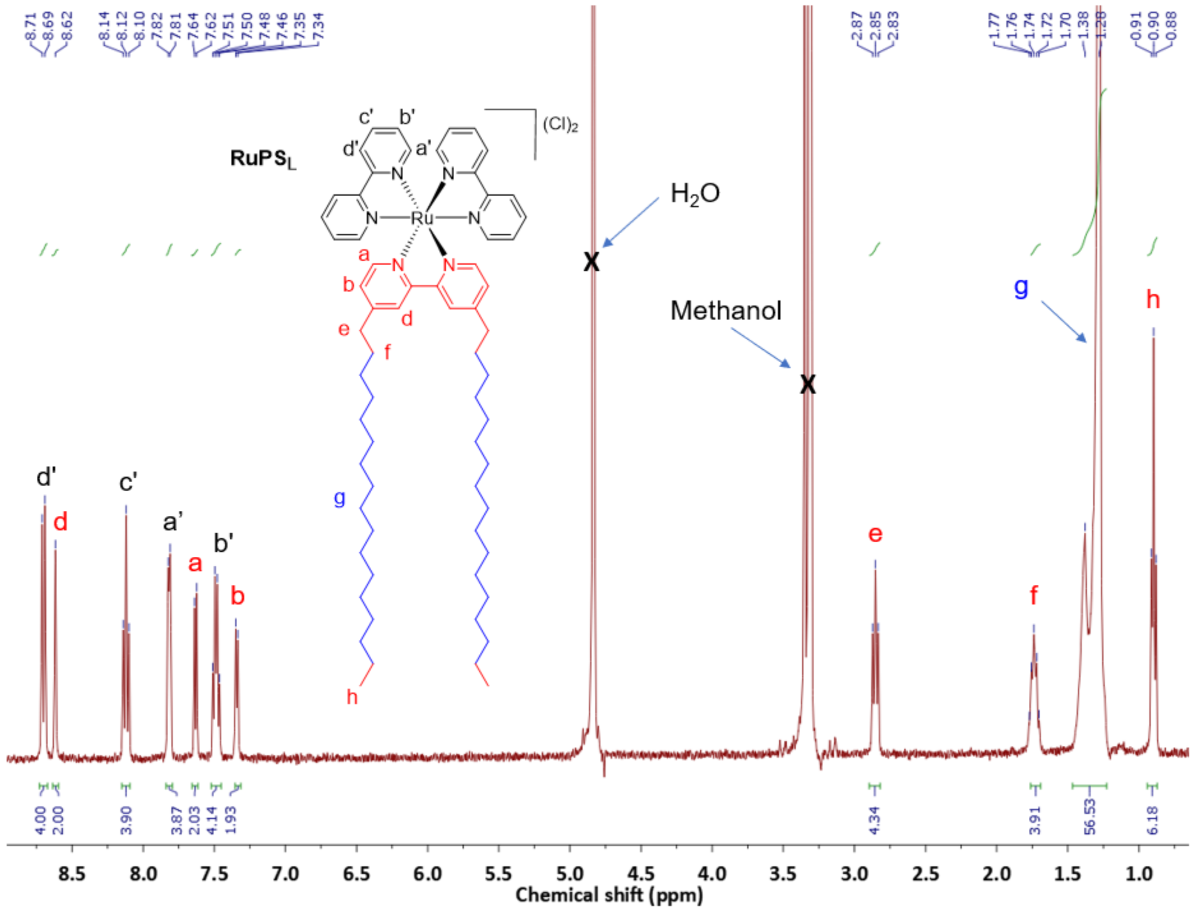

Figure S4. ^{1}H NMR of alkylated ruthenium tris-bipyridine photosensitizer **RuPS$_L$** in CD_3OD.

Absorption spectroscopy of molecular species

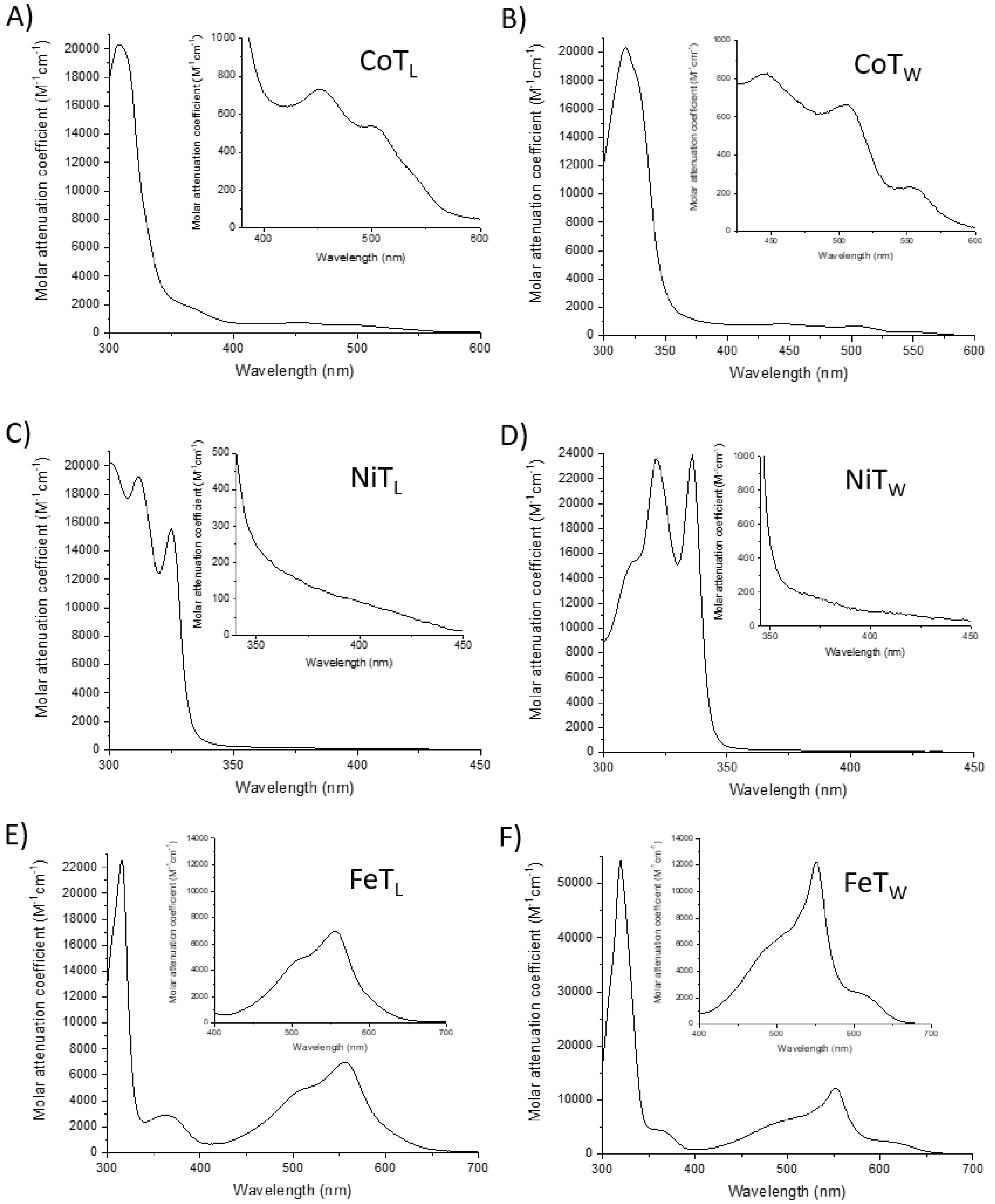

Figure S5. UV-vis absorption spectra in methanol of terpyridine-based catalysts: (A) **CoT$_L$**, (B) **CoT$_W$**, (C) **NiT$_L$**, (D) **NiT$_W$**, (E) **FeT$_L$** and (F) **FeT$_W$**. In all cases, due to the electron-donating effect of the $OC_{16}H_{33}$ tails **MT$_L$** present a small blue shift of the absorption features compared to **MT$_W$**.

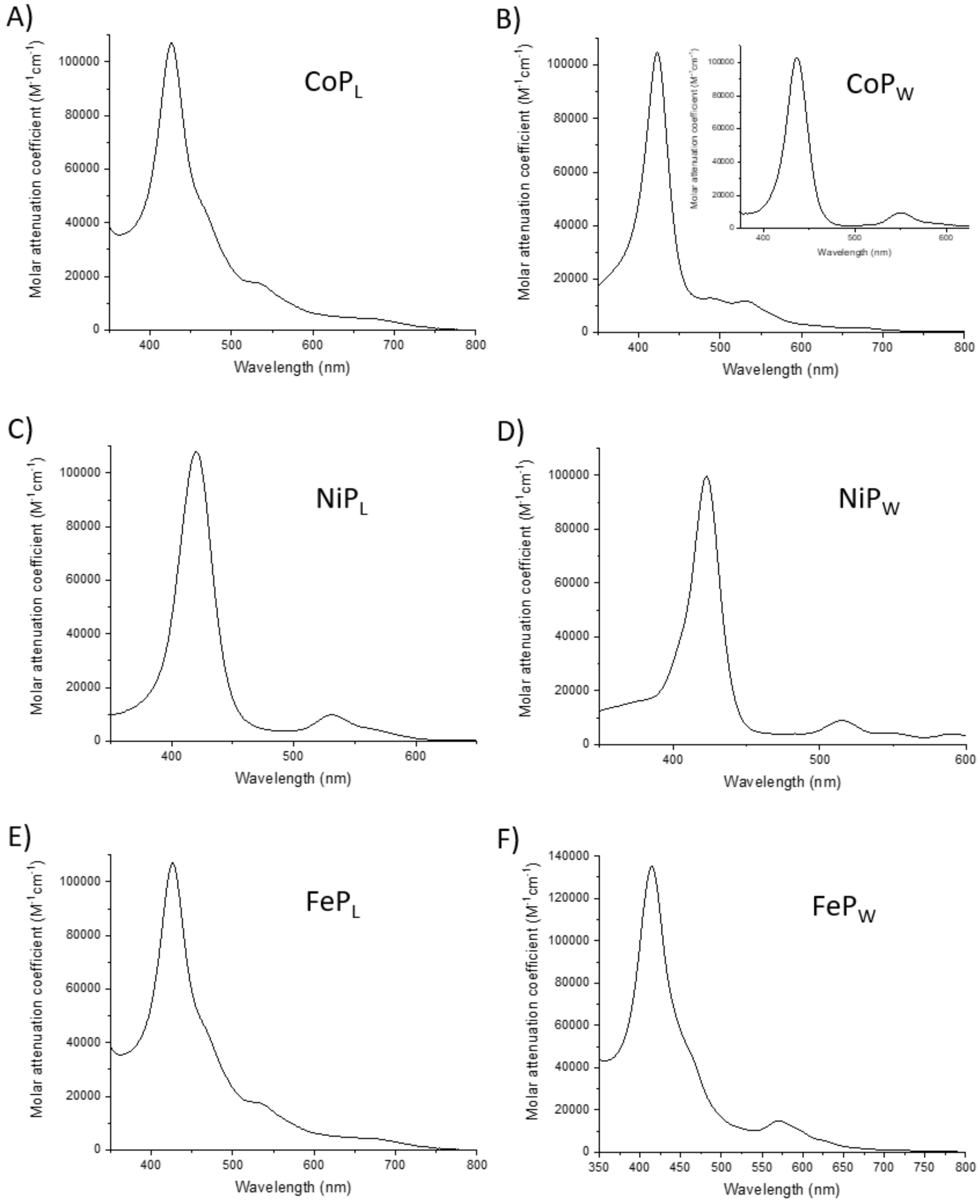

Figure S6. UV-vis absorption spectra in acetone of porphyrin-based catalysts: (A) **CoP$_L$**, (B) **CoP$_W$** (inset, in Milli-Q water), (C) **NiP$_L$**, (D) **NiP$_W$**, (E) **FeP$_L$** and (F) **FeP$_W$**.

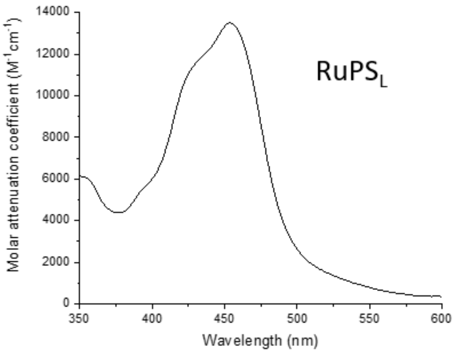

Figure S7. UV-vis absorption spectrum of photosensitizer **RuPS$_L$** in methanol.

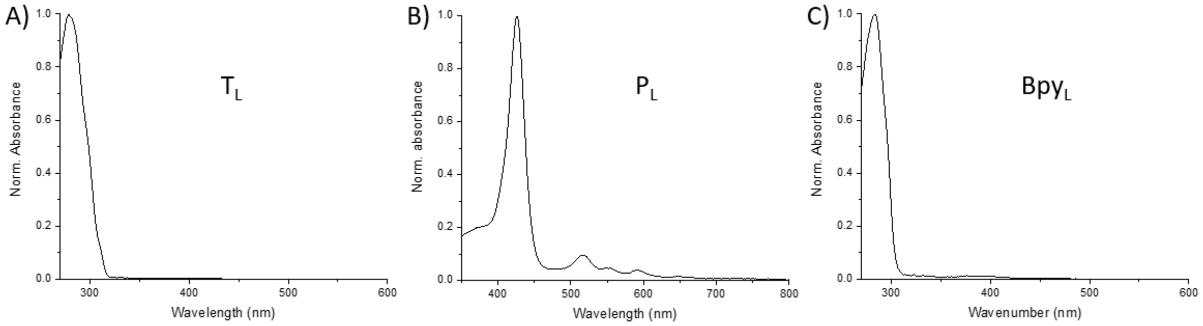

Figure S8. UV-vis absorption spectrum of (A) **T$_L$** in $CHCl_3$, (B) **P$_L$** in methanol and (C) **Bpy$_L$** in $CHCl_3$.

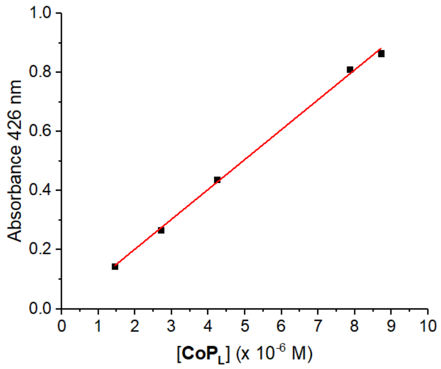

Figure S9. Calibration curve for the Soret band of **CoP$_L$** at 426 nm in acetone. Molar attenuation coefficient (ε_{426nm} = $1.07 \cdot 10^5$ M^{-1} cm^{-1}), i.e. molar extinction coefficient, was calculated for all molecular species from the slope of the linear fit.

Electrochemistry measurements of molecular catalysts in DMF

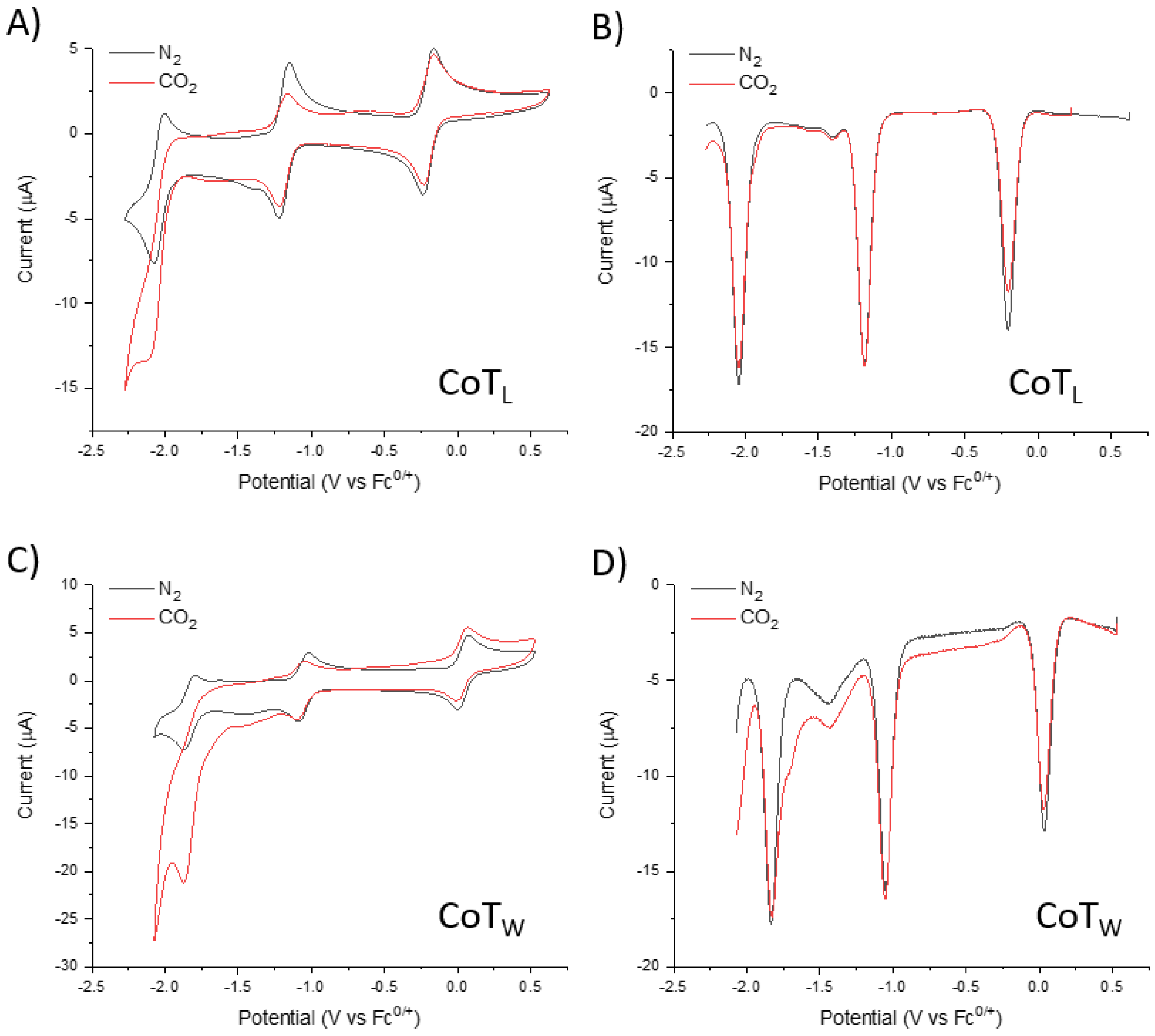

Figure S10. (A and C) CV and (B and D) SWV of (top) **CoT$_L$** and (bottom) **CoT$_W$** in N_2- and CO_2-saturated 0.2 M $TBAPF_6$ DMF solutions. The electrochemical behaviour of both terpyridine-based catalysts match that of previous reports.[3, 13]

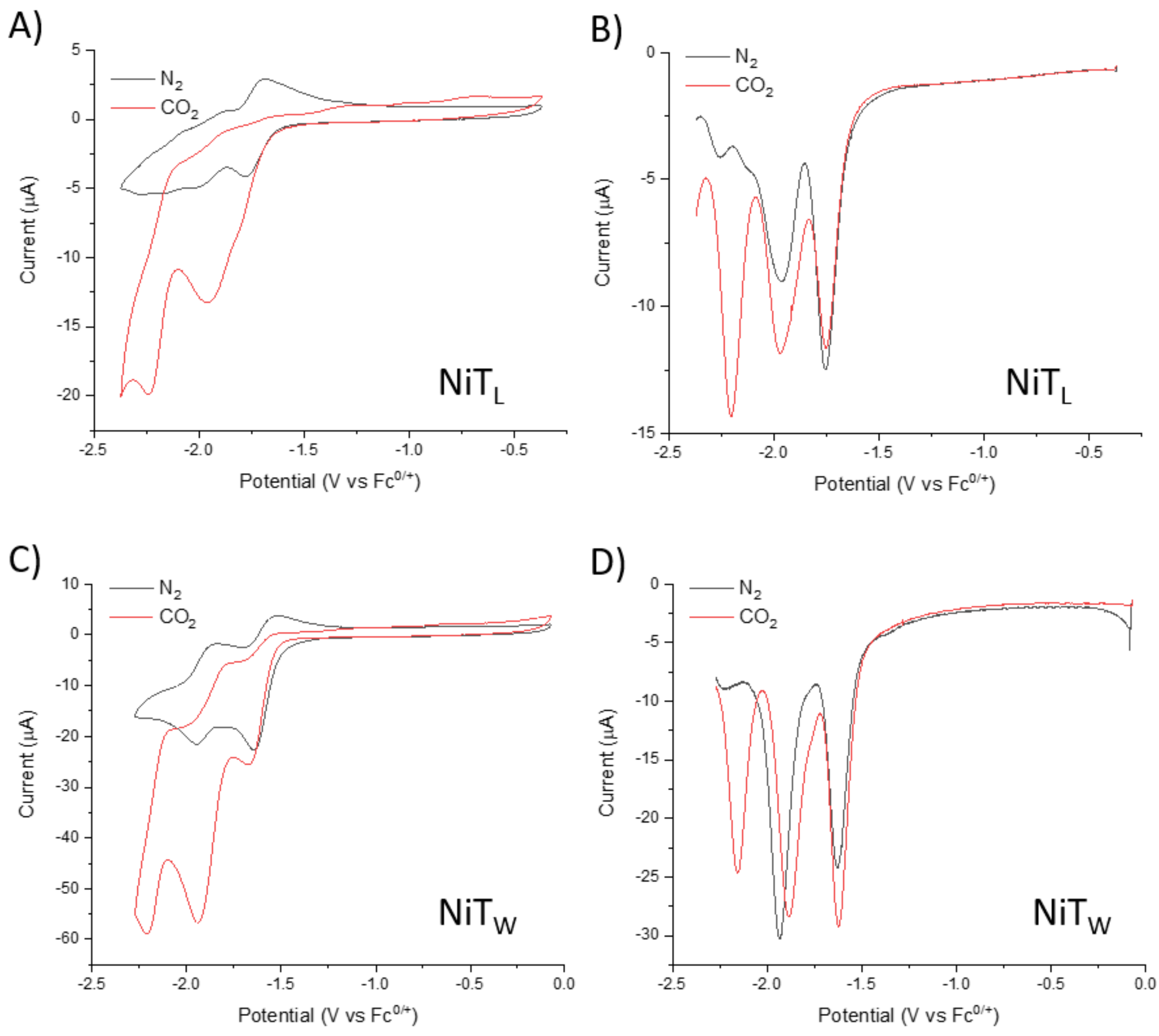

Figure S11. (A and C) CV and (B and D) SWV of (top) **NiT$_L$** and (bottom) **NiT$_W$** in N_2- and CO_2-saturated 0.2 M $TBAPF_6$ DMF solutions. The electrochemical behaviour of both terpyridine-based catalysts match that of previous reports.[3, 13]

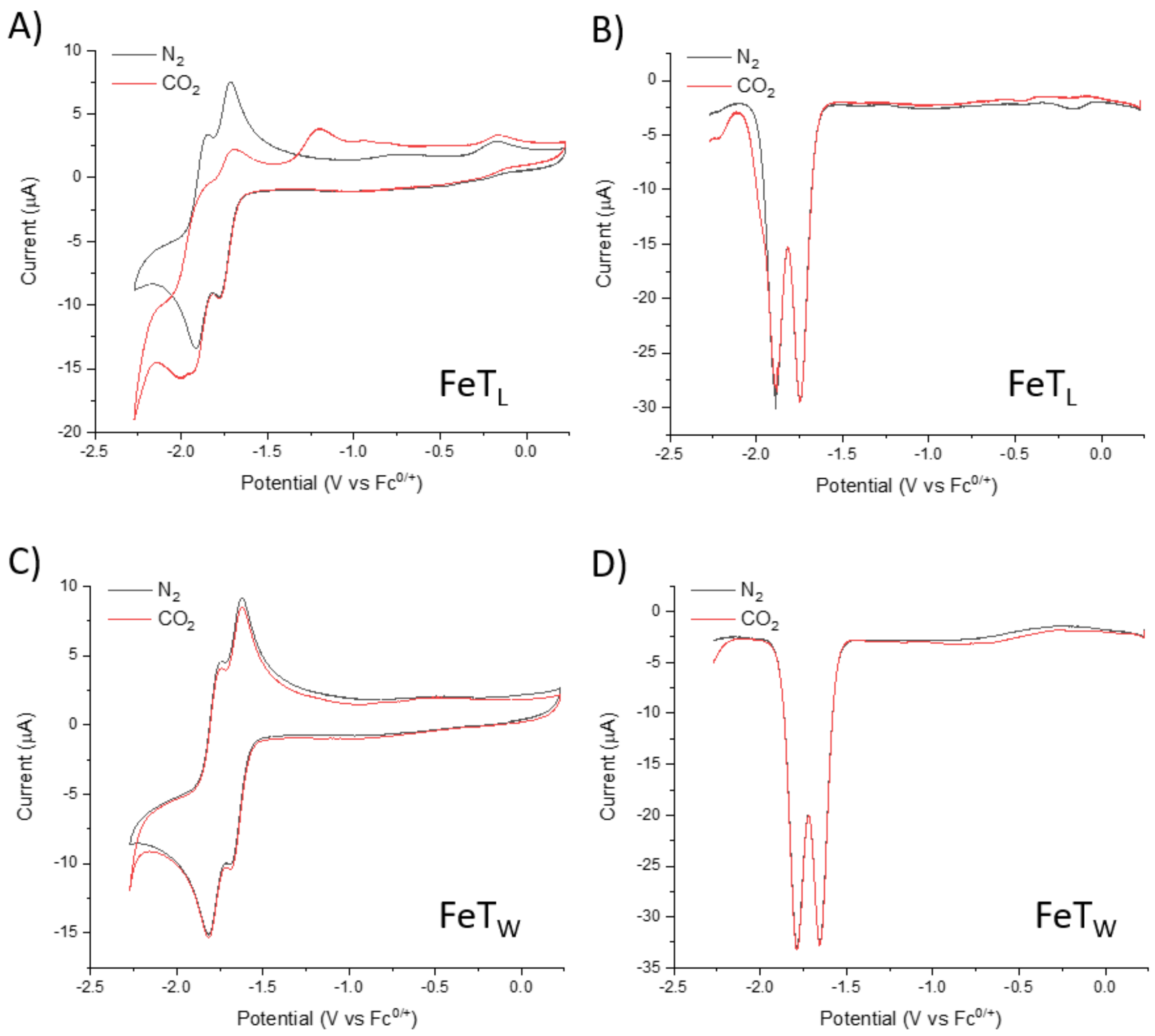

Figure S12. (A and C) CV and (B and D) SWV of (top) **FeT_L** and (bottom) **FeT_W** in N_2- and CO_2-saturated 0.2 M $TBAPF_6$ DMF solutions. The electrochemical behaviour of both terpyridine-based catalysts match that of previous reports.[3, 13]

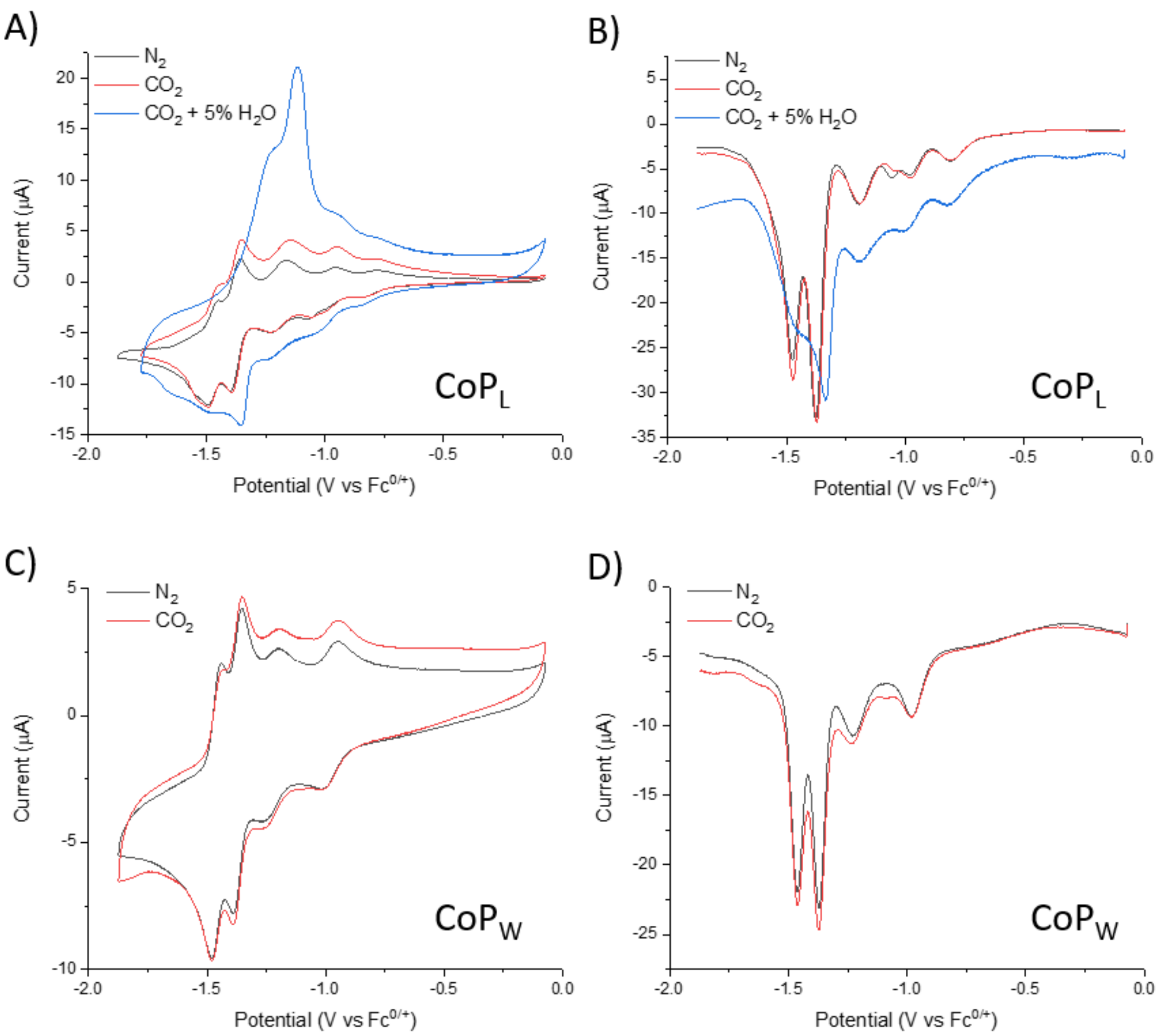

Figure S13. (A and C) CV and (B and D) SWV of (top) **CoP$_L$** and (bottom) **CoP$_W$** in N_2- and CO_2-saturated 0.2 M $TBAPF_6$ DMF solutions. In blue: **CoP$_L$** in CO_2-saturated 95:5 0.2 M $TBAPF_6$ DMF:0.2 M KCl Milli-Q water.

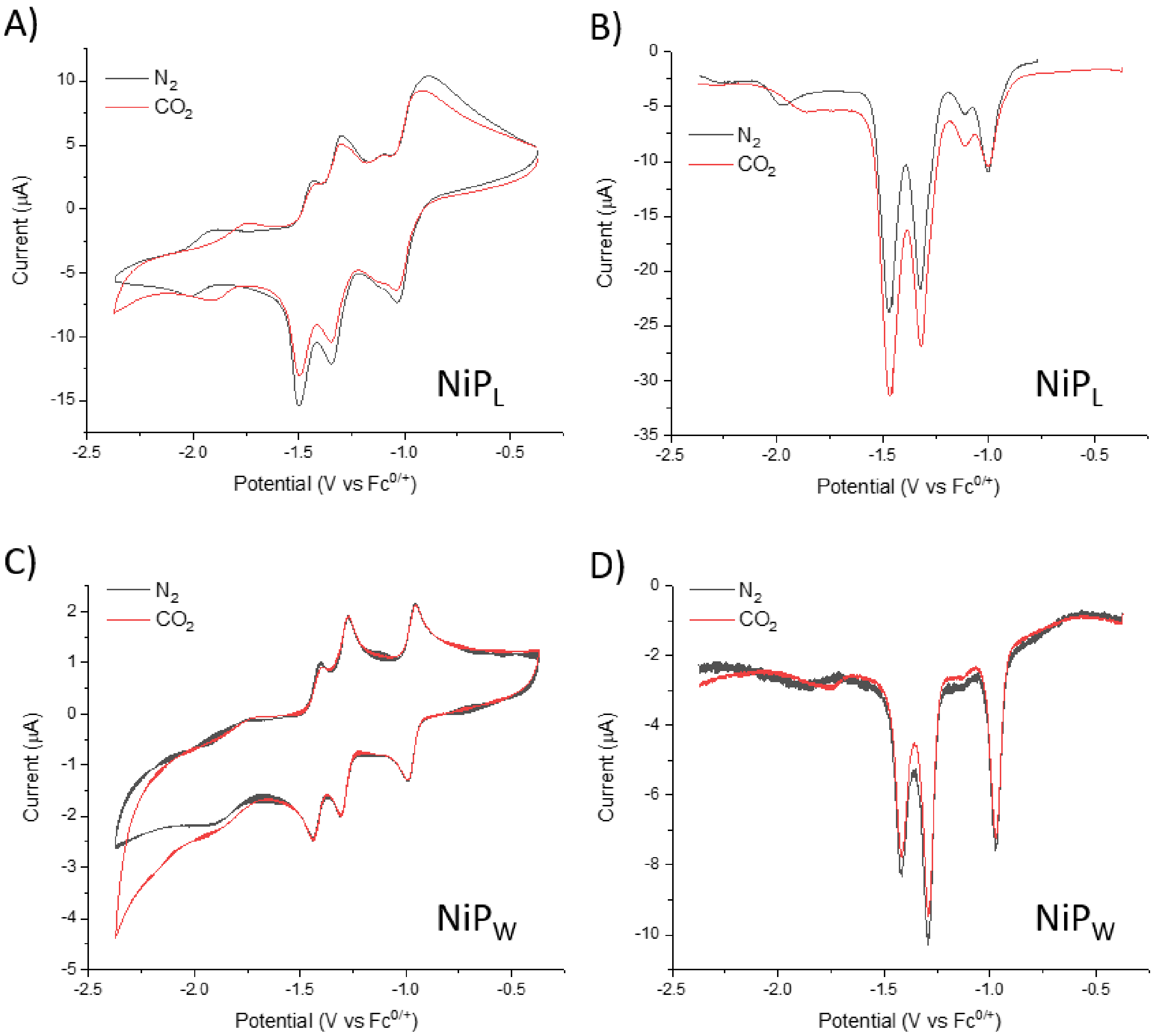

Figure S14. (A and C) CV and (B and D) SWV of (top) **NiP_L** and (bottom) **NiP_W** in N_2- and CO_2-saturated 0.2 M $TBAPF_6$ DMF solutions.

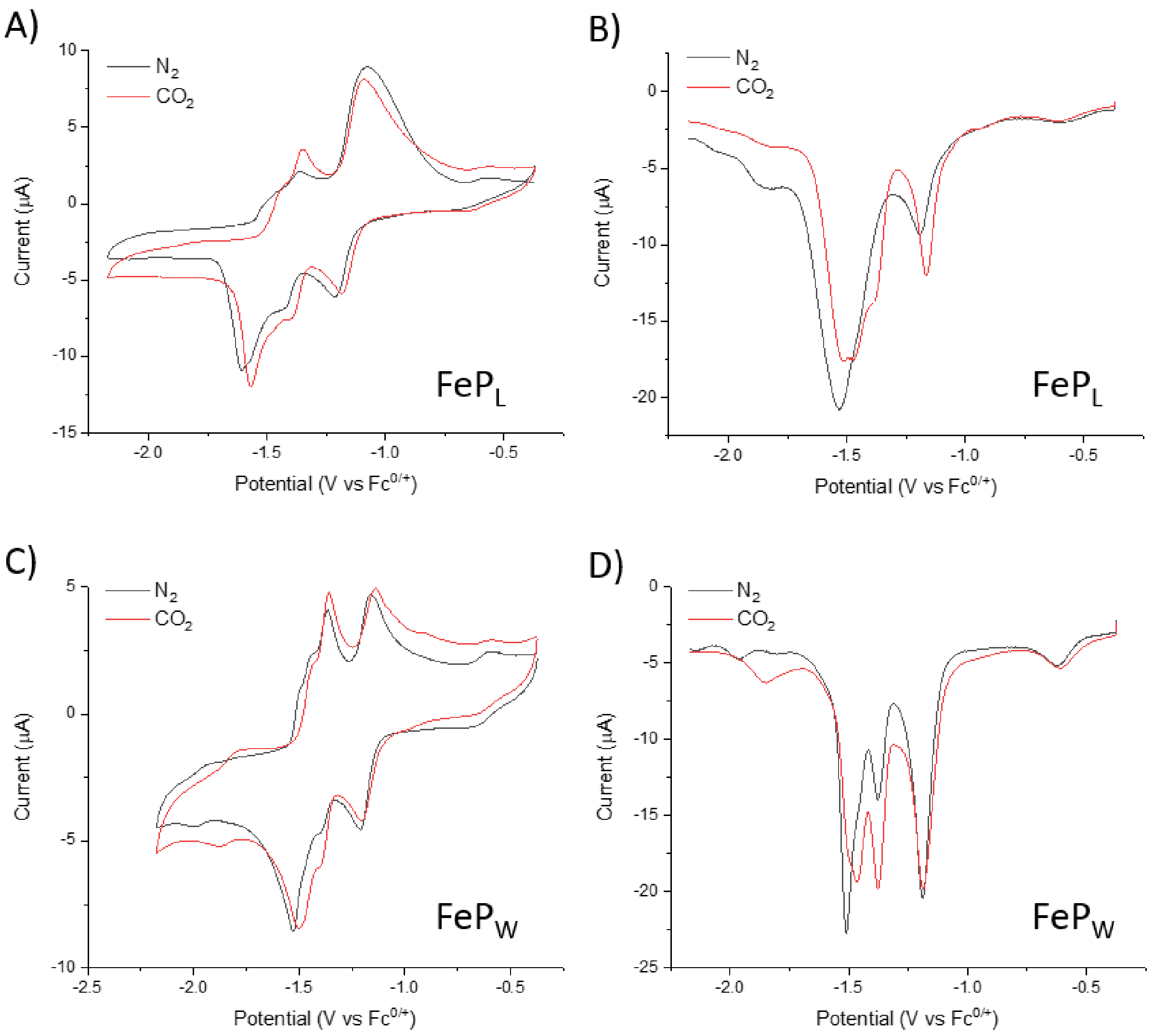

Figure S15. (A and C) CV and (B and D) SWV of (top) **FeP$_L$** and (bottom) **FeP$_W$** in N_2- and CO_2-saturated 0.2 M TBAPF$_6$ DMF solutions.

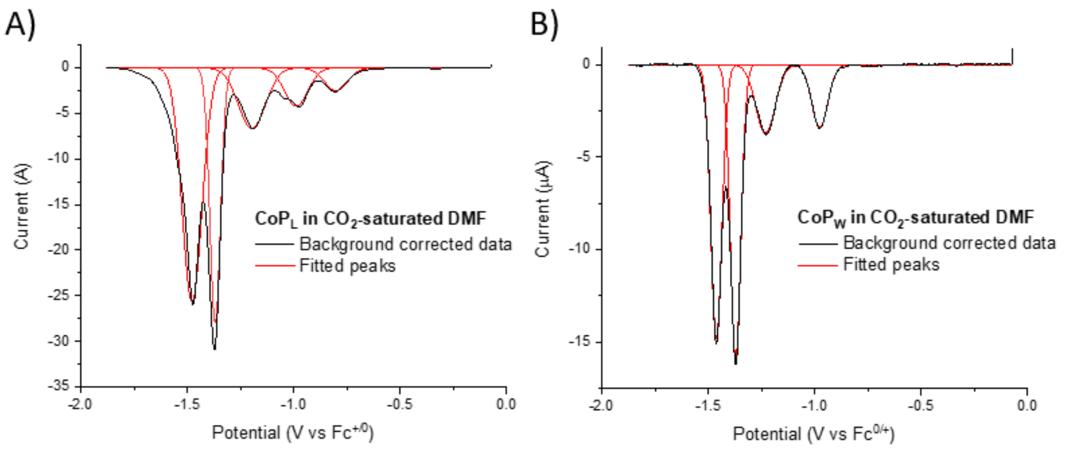

Figure S16. SWV of **CoP_L** and **CoP_W** in CO_2-saturated DMF and (red) fitted peaks. Fitting of peaks was performed using *Multi Peak Fit* function in *OriginPrO 2017*.

Cryogenic transmission electron microscopy

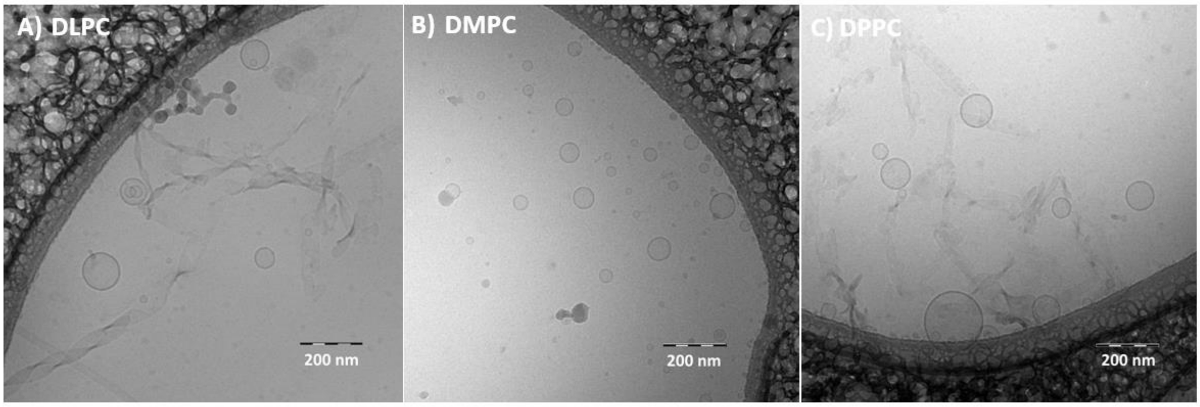

Figure S17. Cryo-TEM picture of unilamellar liposomes containing DLPC, DMPC or DPPC at [lipid] = 100 µM, and [NaDSPE-PEG2K] = 1 µM, [**$RuPS_L$**] = 10 µM and [**NiT_L**] = 2 µM. N.B. for further details see Supplementary Note 1 in section above.

Summary of photocatalytic performance of alkylated catalysts in liposomes

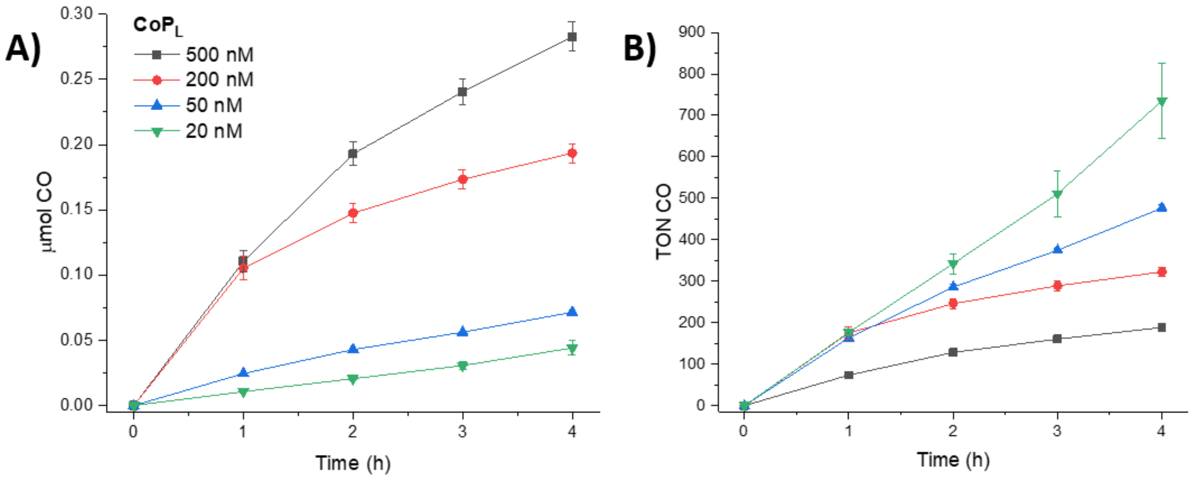

Figure S18. CO formation as a function of time and concentration of **CoP$_L$** in DMPC liposomes containing [DMPC] = 100 µM, [NaDSPE-PEG2K] =1 µM, [**RuPS$_L$**] = 10 µM, [**CoP$_L$**] = 20-500 nM. Conditions: CO_2-saturated 0.1 M $NaHCO_3$ and 0.1 M sodium ascorbate (3 mL, pH = 6.7) at 25 °C under visible light irradiation (λ > 400 nm, AM 1.5G, 100 mW cm^{-2}). Experiments were performed in triplicates. See Tables S7 and S8 for further information.

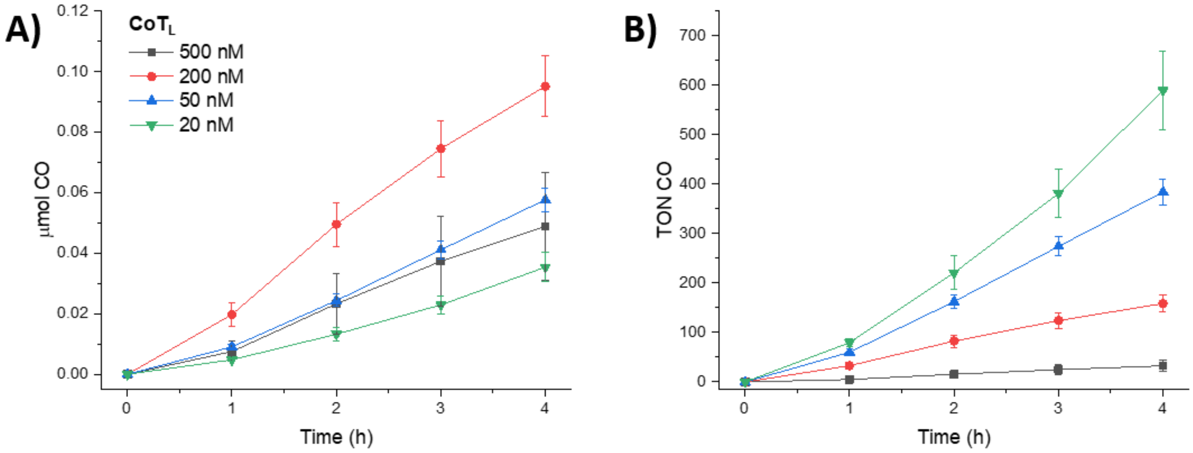

Figure S19. CO formation as a function of time and concentration of **CoT$_L$** in DMPC liposomes containing [DMPC] = 100 µM, [NaDSPE-PEG2K] =1 µM, [**RuPS$_L$**] = 10 µM, [**CoT$_L$**] = 20-500 nM. Conditions: CO_2-saturated 0.1 M $NaHCO_3$ and 0.1 M sodium ascorbate (3 mL, pH = 6.7) at 25 °C under visible light irradiation (λ > 400 nm, AM 1.5G, 100 mW cm^{-2}). Experiments were performed in triplicates. See Tables S7 and S8 for further information.

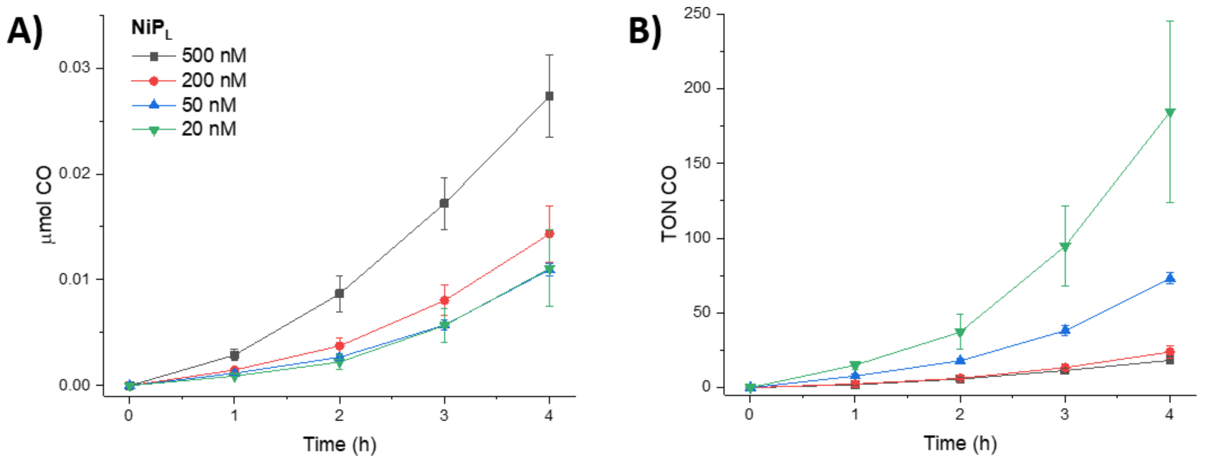

Figure S20. CO formation as a function of time and concentration of **NiP$_L$** in DMPC liposomes containing [DMPC] = 100 µM, [NaDSPE-PEG2K] =1 µM, [**RuPS$_L$**] = 10 µM, [**NiP$_L$**] = 20-500 nM. Conditions: CO_2-saturated 0.1 M $NaHCO_3$ and 0.1 M sodium ascorbate (3 mL, pH = 6.7) at 25 °C under visible light irradiation (λ > 400 nm, AM 1.5G, 100 mW cm^{-2}). Experiments were performed in triplicates. See Tables S7 and S8 for further information.

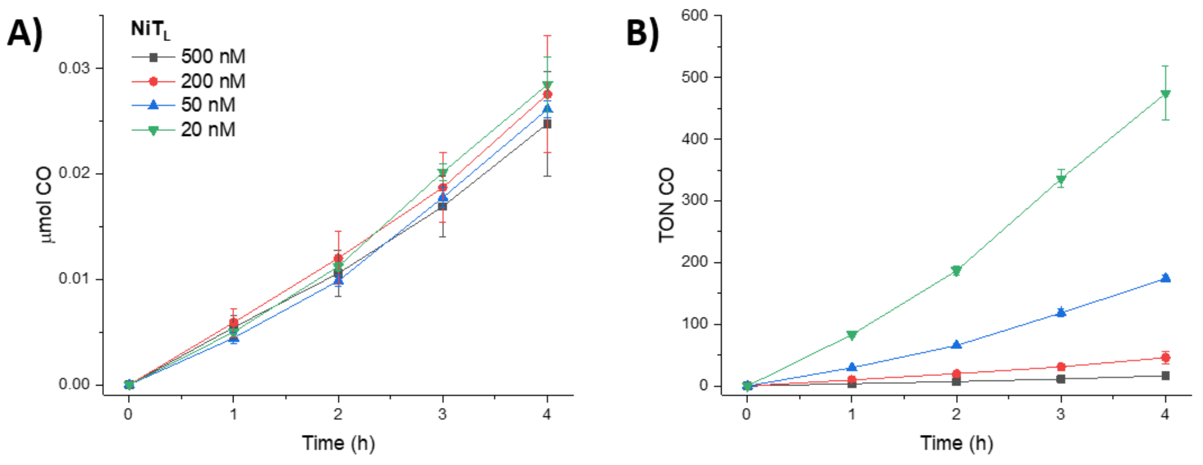

Figure S21. CO formation as a function of time and concentration of **NiT$_L$** in DMPC liposomes containing [DMPC] = 100 µM, [NaDSPE-PEG2K] =1 µM, [**RuPS$_L$**] = 10 µM, [**NiT$_L$**] = 20-500 nM. Conditions: CO_2-saturated 0.1 M $NaHCO_3$ and 0.1 M sodium ascorbate (3 mL, pH = 6.7) at 25 °C under visible light irradiation (λ > 400 nm, AM 1.5G, 100 mW cm^{-2}). Experiments were performed in triplicates. See Tables S7 and S8 for further information.

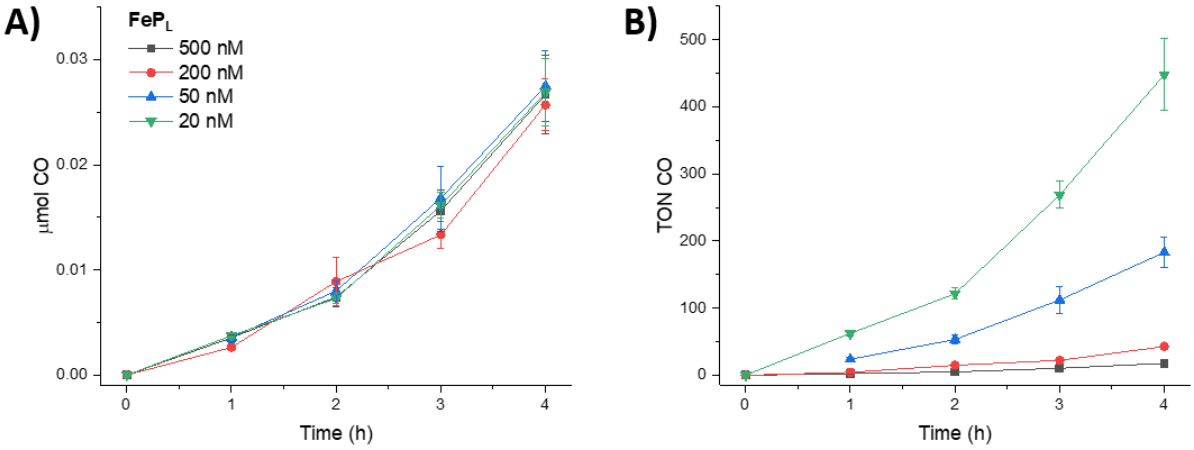

Figure S22. CO formation as a function of time and concentration of **FeP$_L$** in DMPC liposomes containing [DMPC] = 100 µM, [NaDSPE-PEG2K] =1 µM, [**RuPS$_L$**] = 10 µM, [**FeP$_L$**] = 20-500 nM. Conditions: CO_2-saturated 0.1 M $NaHCO_3$ and 0.1 M sodium ascorbate (3 mL, pH = 6.7) at 25 °C under visible light irradiation (λ > 400 nm, AM 1.5G, 100 mW cm^{-2}). Experiments were performed in triplicates. See Tables S7 and S8 for further information.

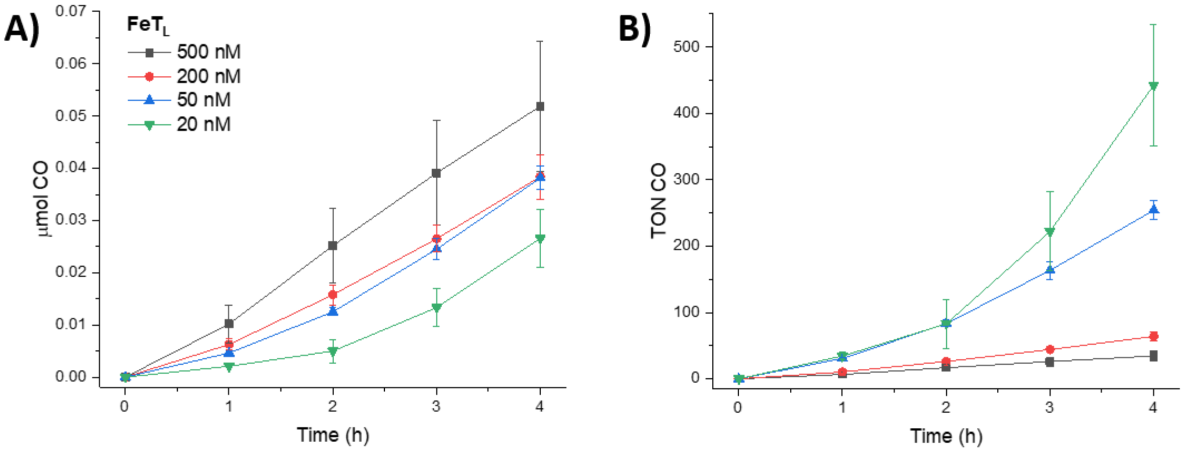

Figure S23. CO formation as a function of time and concentration of **FeT$_L$** in DMPC liposomes containing [DMPC] = 100 µM, [NaDSPE-PEG2K] =1 µM, [**RuPS$_L$**] = 10 µM, [**FeT$_L$**] = 20-500 nM. Conditions: CO_2-saturated 0.1 M $NaHCO_3$ and 0.1 M sodium ascorbate (3 mL, pH = 6.7) at 25 °C under visible light irradiation (λ > 400 nm, AM 1.5G, 100 mW cm^{-2}). Experiments were performed in triplicates. See Tables S7 and S8 for further information.

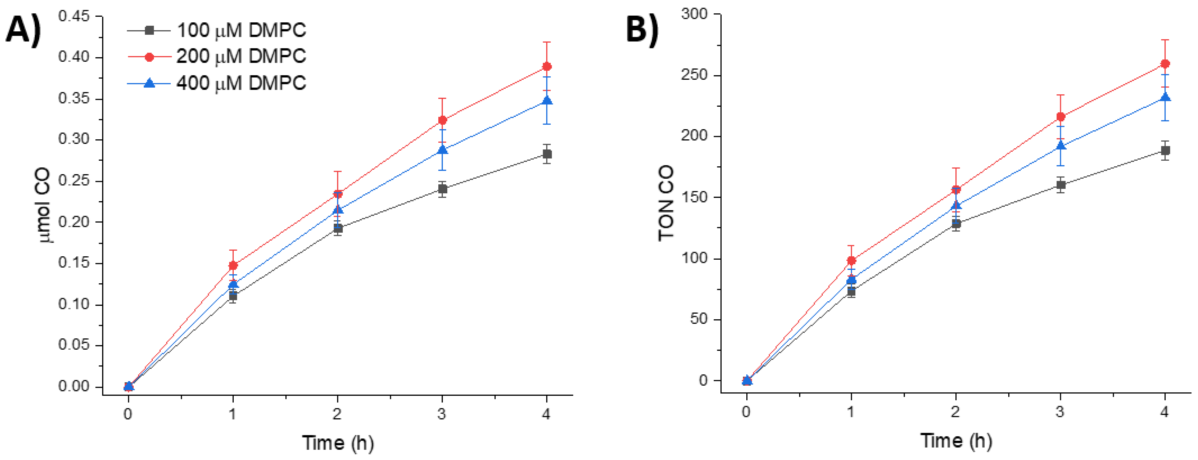

Figure S24. CO formation as a function of time and concentration of DMPC in liposomes containing [DMPC] = 100-400 µM, [NaDSPE-PEG2K] =1-4 µM, [**RuPS$_L$**] = 10 µM, [**CoP$_L$**] = 500 nM. Conditions: CO_2-saturated 0.1 M $NaHCO_3$ and 0.1 M sodium ascorbate (3 mL, pH = 6.7) at 25 °C under visible light irradiation (λ > 400 nm, AM 1.5G, 100 mW cm^{-2}). Experiments were performed in triplicates. See Table S13 for further information.

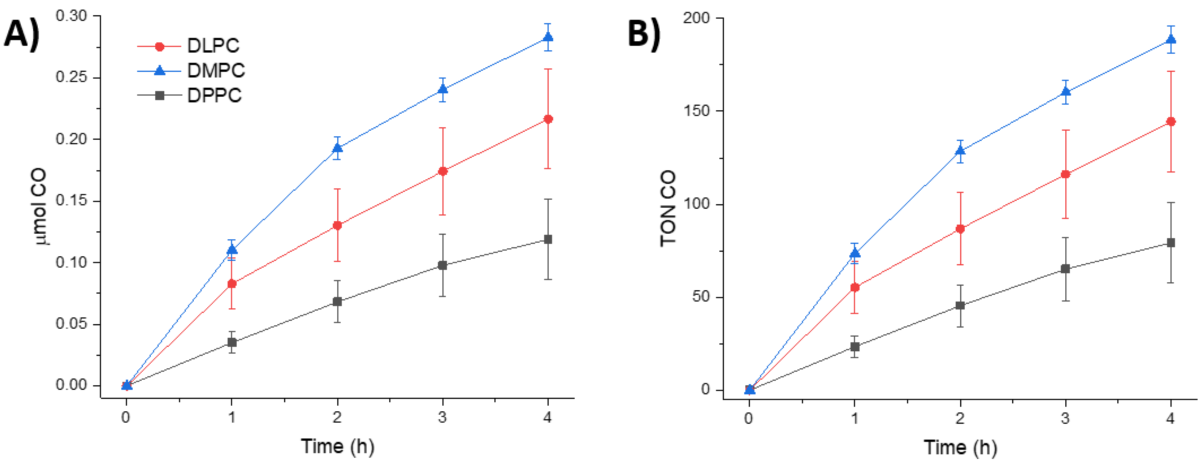

Figure S25. Comparison of CO formation between DLPC, DMPC and DPPC liposomes containing [lipid] = 100 µM, [NaDSPE-PEG2K] =1 µM, [**RuPS$_L$**] = 10 µM, [**CoP$_L$**] = 500 nM. Conditions: four-hour visible light irradiation experiments in triplicates (λ > 400 nm, AM 1.5G, 100 mW cm^{-2}), CO_2-saturated 0.1M $NaHCO_3$ and 0.1 M sodium ascorbate at 25 °C. N.B. for further details see Supplementary Note 1 and Table S13 in sections above.

Isotopic labeling control experiment

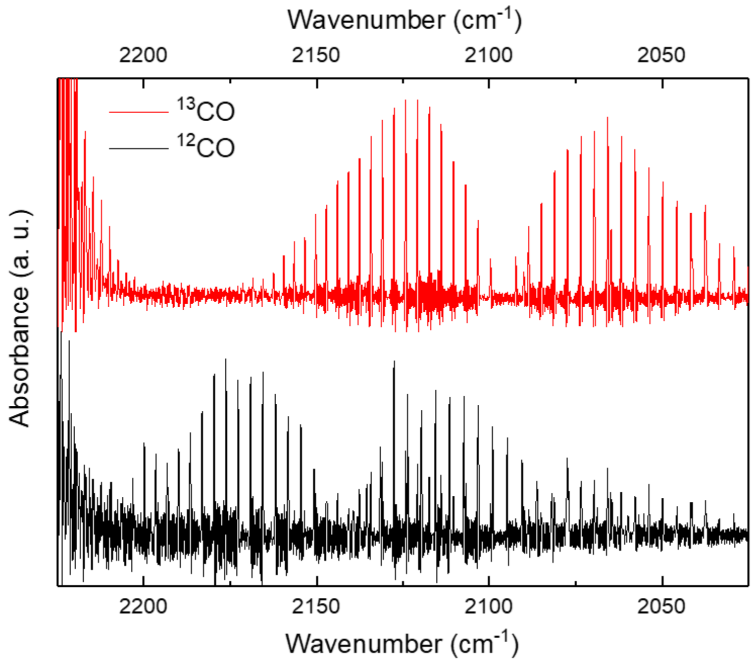

Figure S26. IR absorbance spectra of isotopic labeling experiments of the gaseous products obtained from (red) $^{13}CO_2$ and (black) $^{12}CO_2$ after 4 h of photocatalysis of catalyst **CoP$_L$** in DMPC liposomes. Conditions: [DMPC] = 100 µM, [NaDSPE-PEG2K] = 1 µM, [**RuPS$_L$**] = 10 µM, [**CoP$_L$**] = 500 nM, 0.1 M sodium ascorbate, $^{13}CO_2$-saturated 0.1 M NaH_2PO_4 (red) or $^{12}CO_2$-saturated 0.1 M $NaH^{12}CO_3$ (black).

UV-vis spectra of before and after light irradiation of molecule containing DMPC liposomes

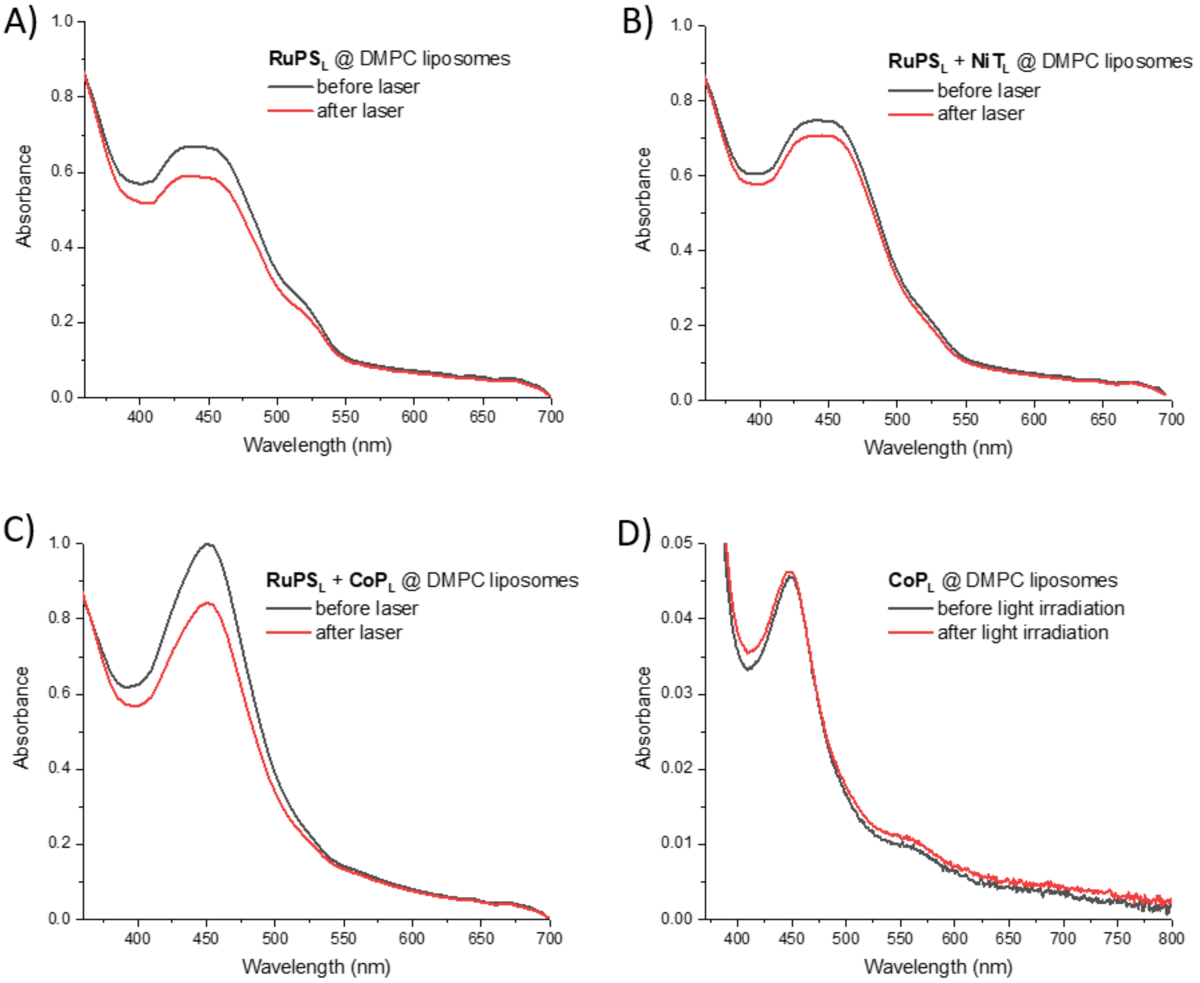

Figure S27. Comparison of UV-vis absorption spectra (black) before and (red) after (A, B and C) laser photolysis or (D) light irradiation of DMPC liposome solutions in the presence of 0.1 M sodium ascorbate in (A, B and C) Ar-saturated or (D) CO_2-saturated 0.1 M $NaHCO_3$. Experimental conditions: (A) [DMPC]=100 µM, [NaDSPE-PEG2K] =1 µM, [**RuPS$_L$**] = 10 µM. (B) [DMPC]=100 µM, [NaDSPE-PEG2K] =1 µM, [**RuPS$_L$**] = 10 µM, [**NiT$_L$**] = 500 nM. (C) [DMPC]=100 µM, [NaDSPE-PEG2K] =1 µM, [**RuPS$_L$**] = 10 µM, [**CoP$_L$**] = 500 nM. (D) [DMPC]=100 µM, [NaDSPE-PEG2K] =1 µM, [**CoP$_L$**] = 500 nM.

Reductive quenching studies

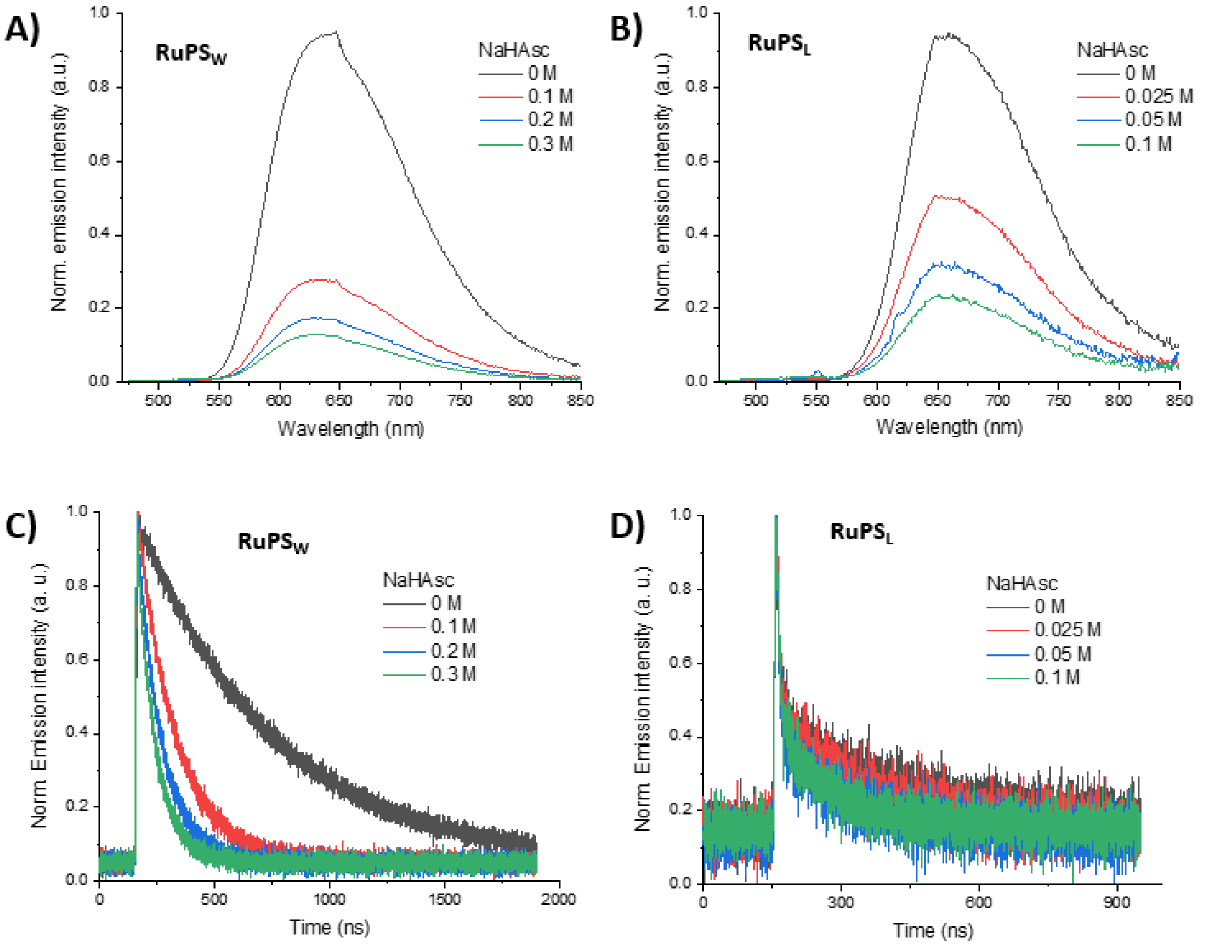

Figure S28. Normalized (A and B) emission intensity and (C and D) time-resolved emission intensity of photosensitizers **RuPS$_W$** and **RuPS$_L$** by sodium ascorbate (NaHAsc), determined by stationary fluorimetry (excitation at 460 nm, detection wavelength at 650 nm) in homogenous environment and liposomes. Experimental homogenous conditions (A and C): [**RuPS$_W$**] = 30 µM in 0.1 M $NaHCO_3$ buffer. Experimental liposome conditions (B and D): [DMPC]=100 µM, [NaDSPE-PEG2K] =1 µM, [**RuPS$_L$**] = 10 µM in 0.1 M $NaHCO_3$ buffer. All solutions were purged with Ar before measurements. NB: The emission intensity decreases successively with addition of increasing concentrations of NaHAsc in both homogeneous and liposomal systems (Figure S26A-B). The observed emission lifetime in homogeneous solution shows a parallel decrease, while in liposomes the lifetime is not significantly affected, as is characteristic for so-called static quenching with pre-aggregated dye-quencher pairs (see main article).

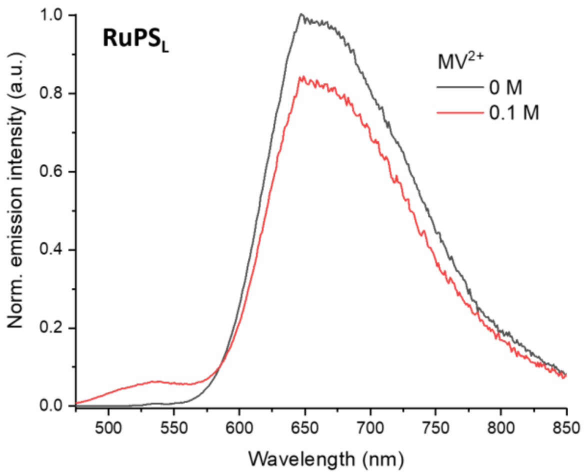

Figure S29. Normalized emission intensity of photosensitizer **RuPS$_L$** by 0.1 M methyl viologen ($MVCl_2$). Conditions liposomes: [DMPC]=100 µM, [NaDSPE-PEG2K] =1 µM, [**RuPS$_L$**] = 10 µM in 0.1 M $NaHCO_3$ buffer. All solutions were purged with Ar before measurements. N.B: The emission intensity decreases modestly with the addition of cationic quencher methyl viologen in the positively-charged liposome system. The use of negatively-charged $HAsc^-$ as a quencher for positively-charged liposomes yields a larger decrease in emission intensity, and involves micro-heterogenous environments, which can enhance the quenching quantum efficiency ($\varphi = 1–I/I_0$) of photosensitizer with anionic $HAsc^-$ ($\varphi = 0.74$) vs cationic quencher methyl viologen ($\varphi = 0.16$).

Self-quenching studies

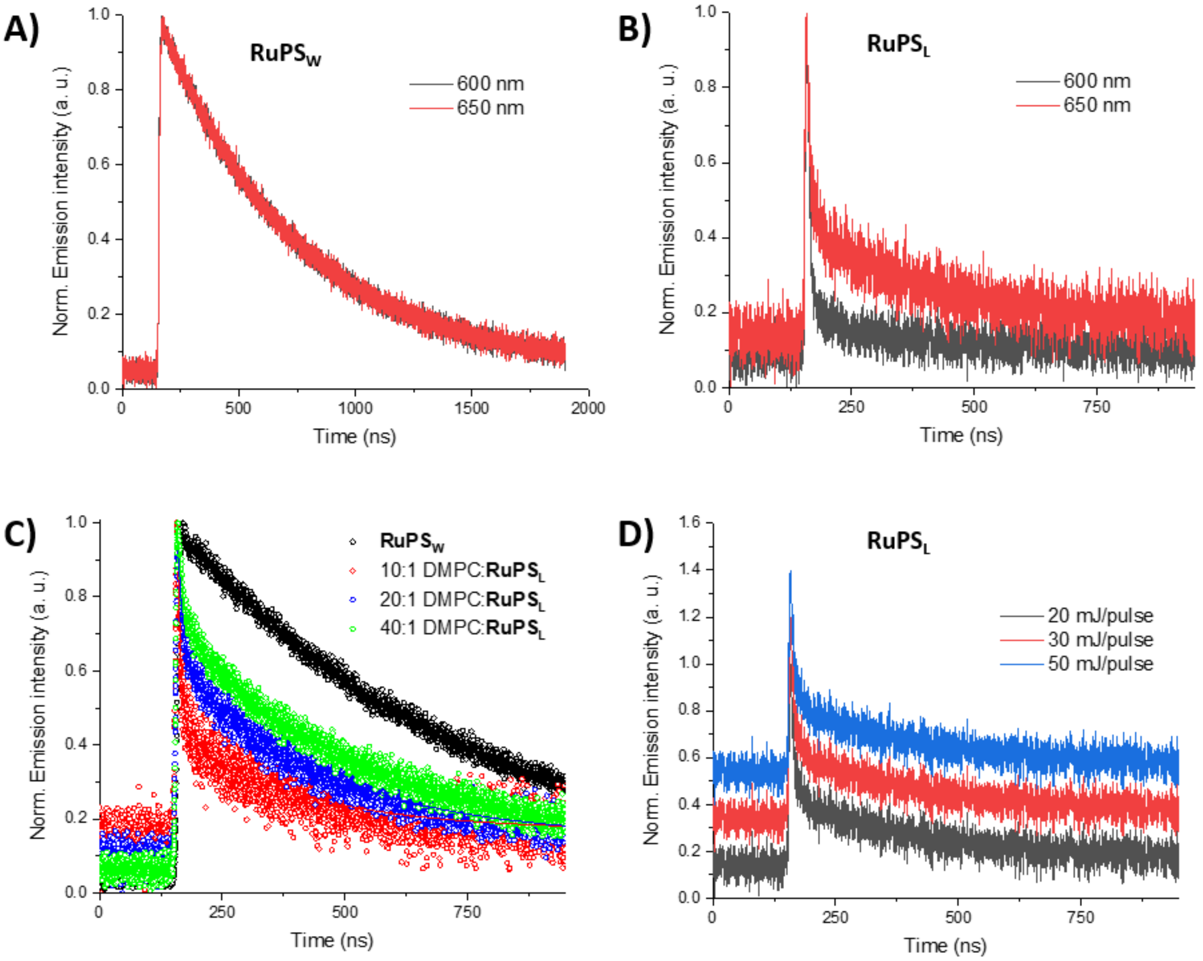

Figure S30. Normalized time-resolved emission intensity of (A) **RuPS$_W$** and (B) **RuPS$_L$** at 600 and 650 nm. Experimental homogenous conditions (A): [**RuPS$_W$**] = 30 µM, in 0.1 M $NaHCO_3$ buffer. Experimental liposome conditions (B): [DMPC]=100 µM, [NaDSPE-PEG2K] =1 µM, [**RuPS$_L$**] = 10 µM, in 0.1 M $NaHCO_3$ buffer. (C) Normalized time-resolved emission intensity at 650 nm of **RuPS$_W$** (black trace) and **RuPS$_L$** in liposomes with different mole ratios between DMPC and **RuPS$_L$**. (D) Normalized time-resolved emission intensity at 650 nm of **RuPS$_L$** in liposomes (10:1 DMPC:**RuPS$_L$** ratio) using different laser excitation energies. Experimental homogeneous conditions (C): [**RuPS$_W$**] = 30 µM, in 0.1 M $NaHCO_3$ buffer. Experimental liposome conditions (C and D): [DMPC]=100 µM, [NaDSPE-PEG2K] =1 µM, [**RuPS$_L$**] = 10, 5, 2.5 µM, Ar-purged 0.1 M $NaHCO_3$ buffer at 20 °C. Excitation and emission wavelengths were fixed at 460 and 650 nm, respectively. N.B. for further details see Supplementary Note 2 in section above.

Charge separation quantum yields in homogeneous and liposome systems

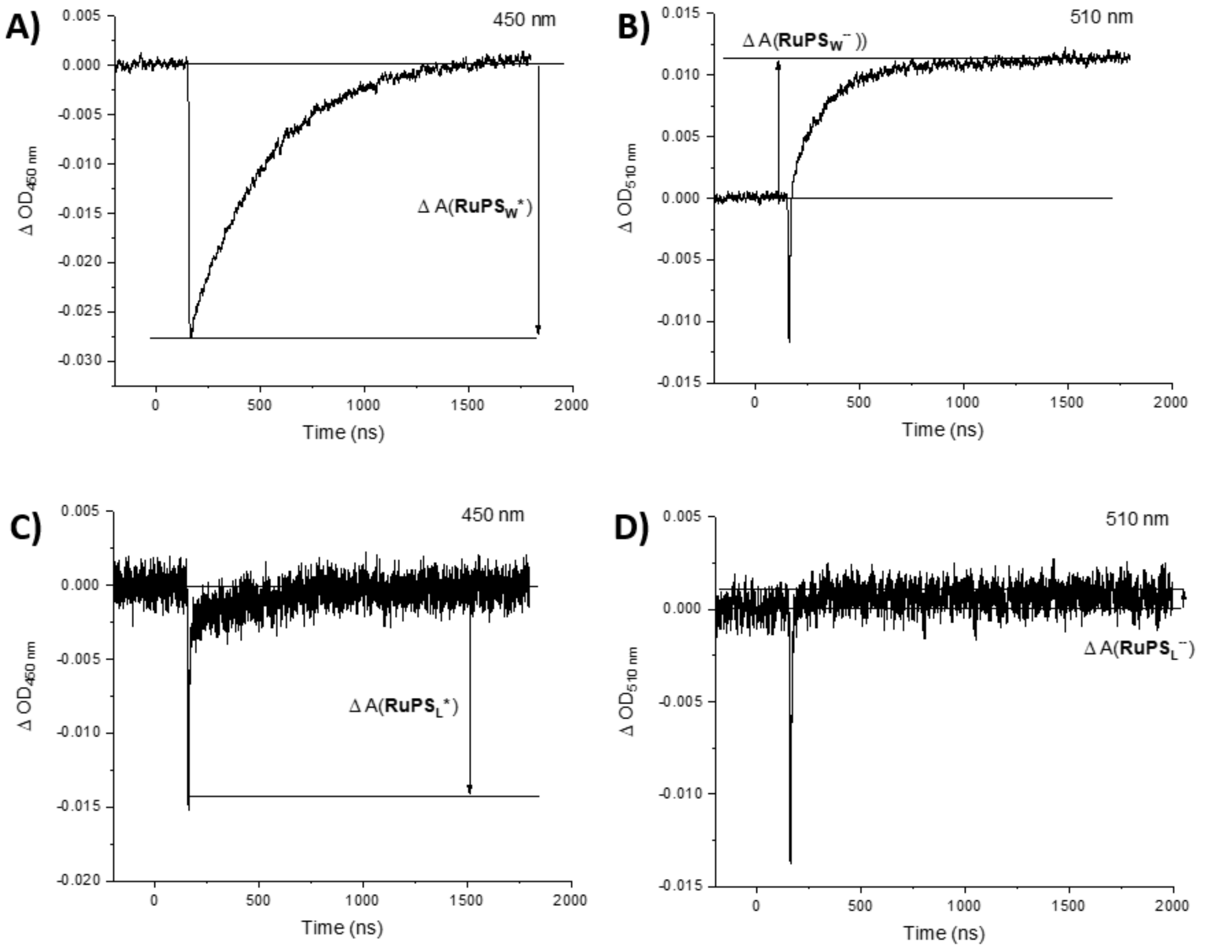

Figure S31. Transient absorption kinetic traces (A and C) at 450nm (ΔA(**$RuPS_W$***) and ΔA(**$RuPS_L$***), respectively) and (B and D) at 510nm (ΔA(**$RuPS_W$**$^-$) and ΔA(**$RuPS_L$**$^-$), respectively), after 460 nm laser excitation of (A and B) homogeneous **$RuPS_W$** and (C and D) liposome **$RuPS_L$** systems. Experimental homogeneous conditions (A and B): [**$RuPS_W$**] = 20 µM and 0.1 M sodium ascorbate in 0.1 M $NaHCO_3$ aq. buffer. Experimental liposome conditions (C and D): [DMPC] = 100 µM, [NaDSPE-PEG2K] = 1 µM, [**$RuPS_L$**] = 10 µM and 0.1 M ascorbate in 0.1 M $NaHCO_3$ aq. buffer. . N.B. for further details see Supplementary Note 3 in section above.

Monitoring the generation of RuPS⁻ in homogeneous and charge separation lifetimes in liposomes

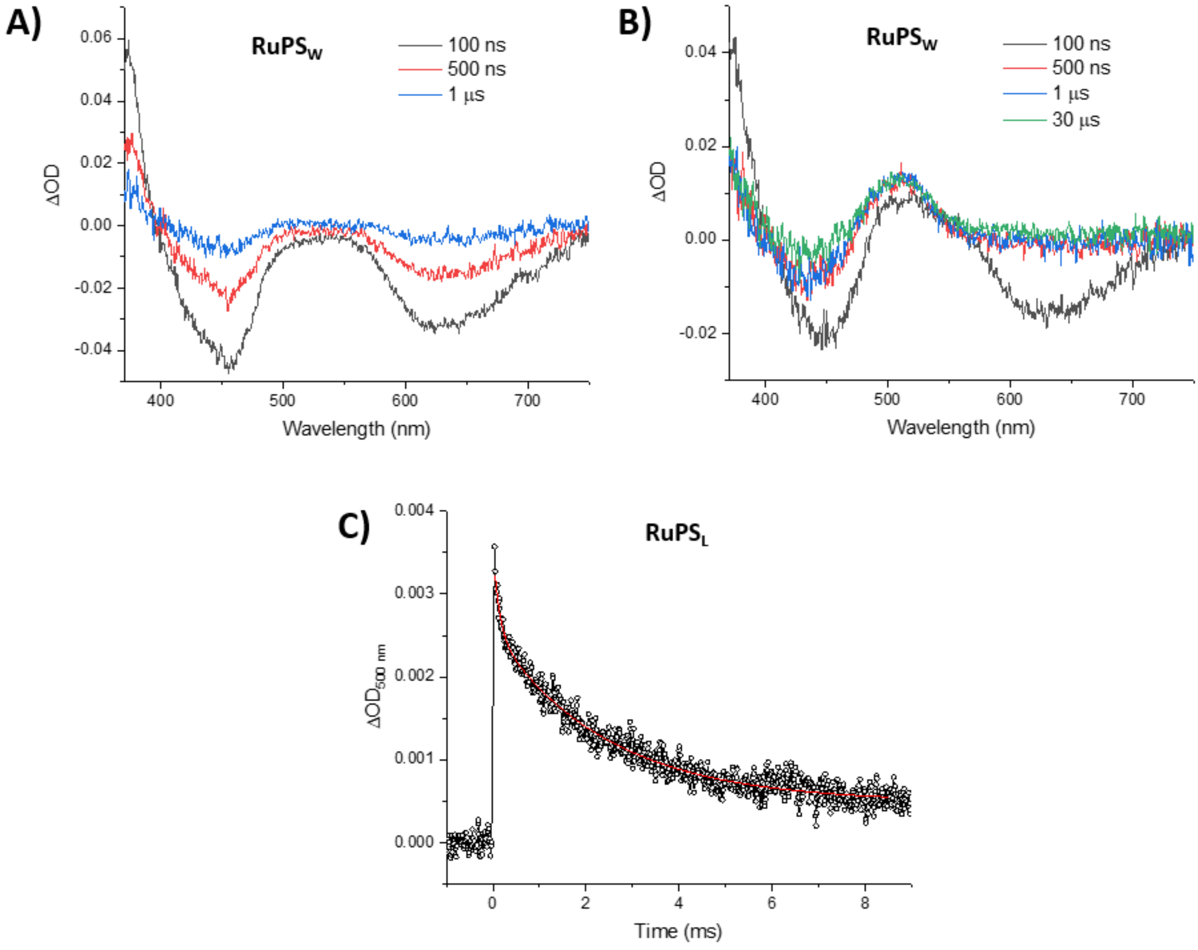

Figure S32. Transient difference absorption spectra of homogeneous **RuPS$_W$** system (A) without and (B) with 0.1 M NaHAsc, following 460 nm laser excitation (1 Hz, 10 mJ/pulse) at different durations in a degassed solution. (C) Transient different absorption kinetic trace collected at 500 nm after laser excitation in 10 ms timescale for **RuPS$_L$** in DMPC liposomes. Experimental homogeneous conditions: (A) 30 µM **RuPS$_W$** in 0.1 M $NaHCO_3$ buffer or (B) 30 µM **RuPS$_W$** and 0.1 M NaHAsc in 0.1 M $NaHCO_3$ buffer. Experimental liposomes conditions (C): [DMPC]=100 µM, [NaDSPE-PEG2K] =1 µM, [**RuPS$_L$**] = 10 µM and 0.1 M NaHAsc in 0.1 M $NaHCO_3$ buffer.

Kinetic studies between RuPS⁻ and catalysts in homogeneous and liposomes

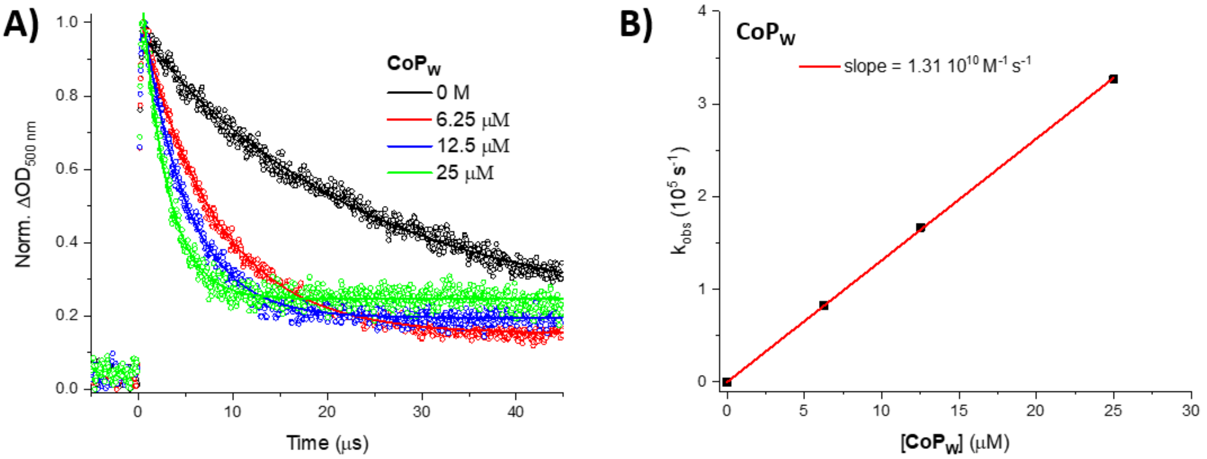

Figure S33. (A) Normalized kinetic traces at 500 nm (original ΔOD ≈ 0.03) obtained at different [**CoP$_W$**] and (B) observed rates vs the concentrations for the calculation of the bimolecular rate constants between **RuPS$_W$** and **CoP$_W$.** Experimental conditions: [**CoP$_W$**] = 0-25 µM, [**RuPS$_W$**] = 30 µM and 0.1 M sodium ascorbate in 0.1 M $NaHCO_3$ aq. buffer.

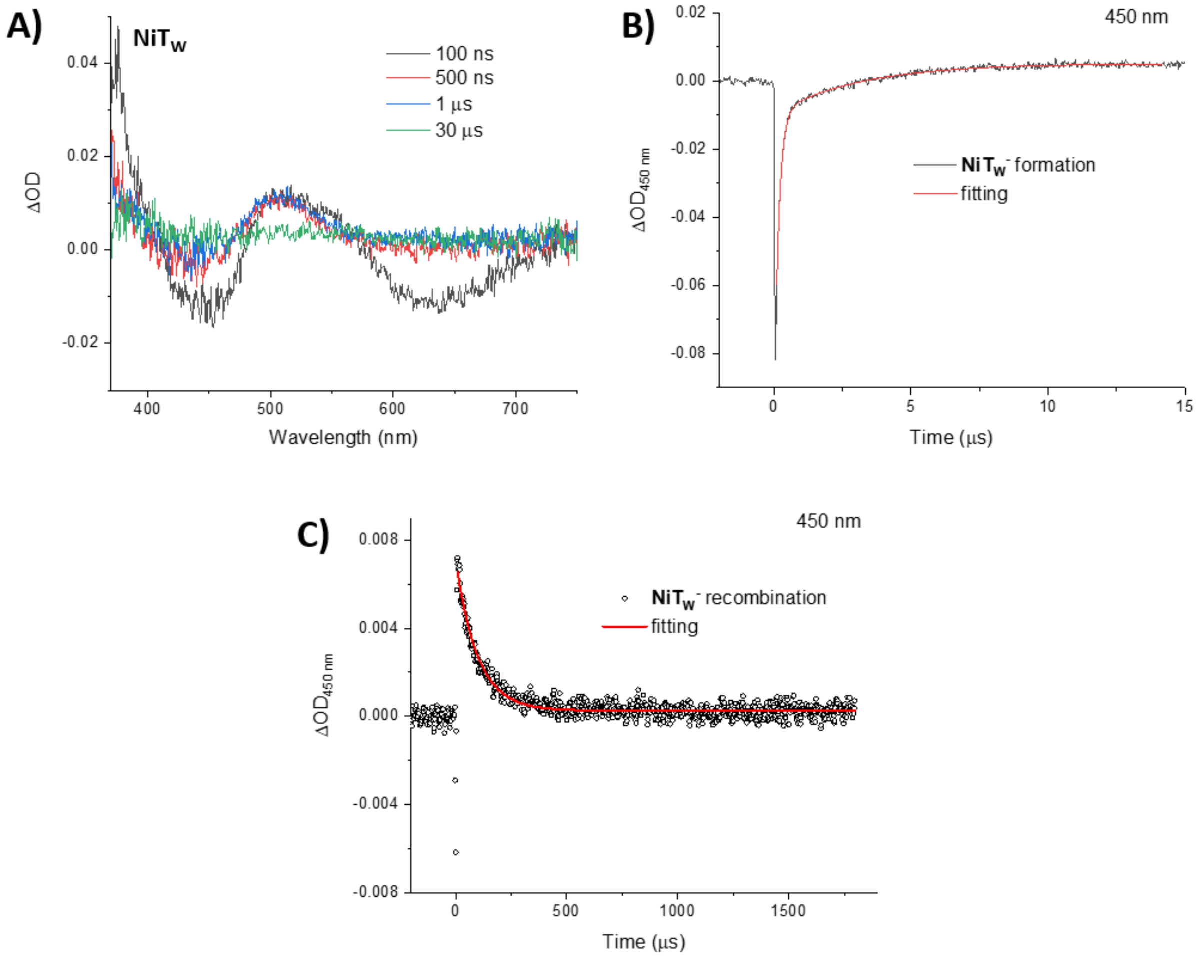

Figure S34. (A) Difference absorption spectra obtained after 460 nm laser excitation (1 Hz, 10 mJ/pulse) of a homogeneous solution containing **RuPS**$_W$ and **NiT**$_W$. (B and C) Transient absorption kinetic traces collected at 450 nm after 460 nm laser excitation (1 Hz, 10 mJ/pulse) at 20 µs and 2000 µs timescale, respectively. Experimental homogeneous conditions: [**RuPS**$_W$] = 30 µM, [**NiT**$_W$] = 100 µM, 0.1 M sodium ascorbate, Ar-saturated 0.1 M $NaHCO_3$.

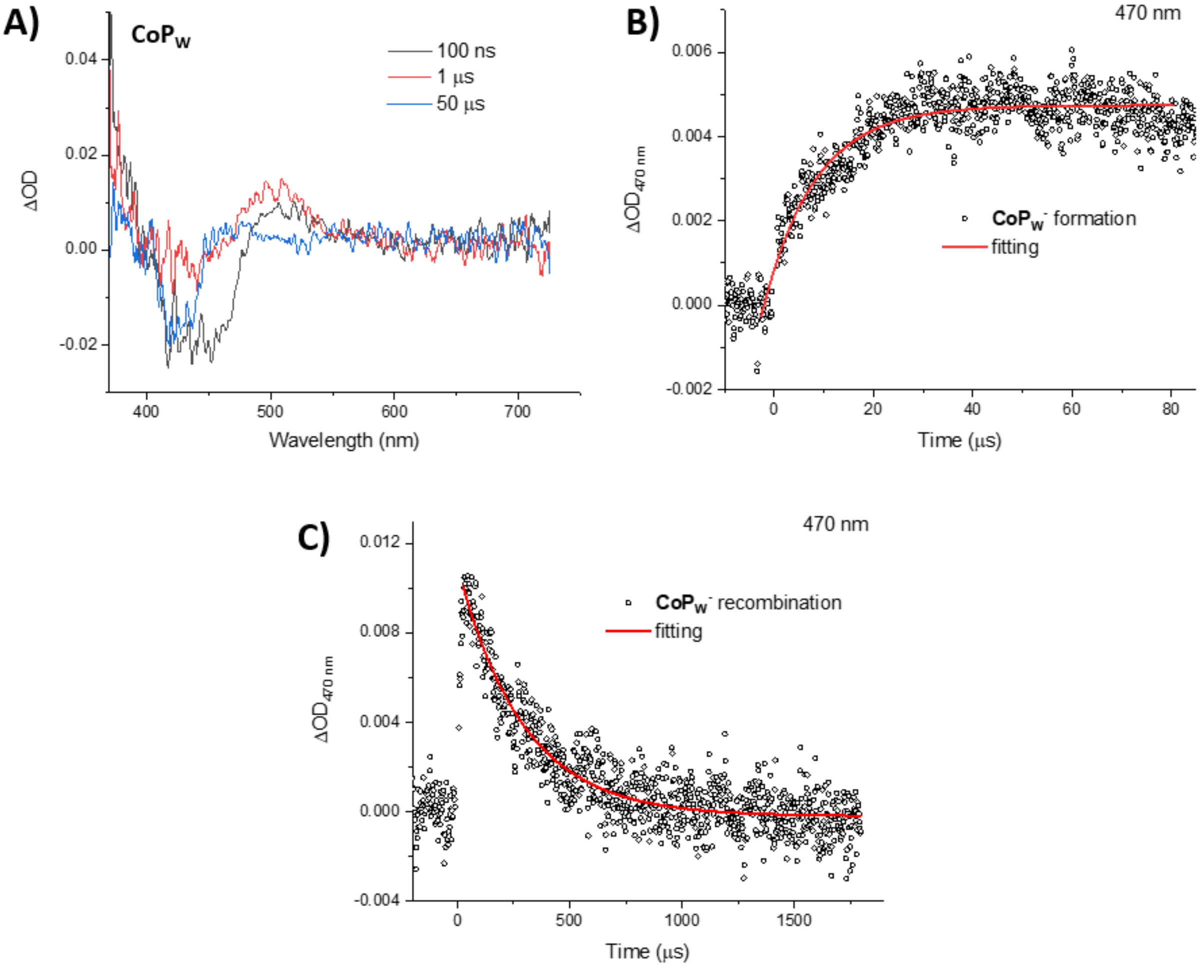

Figure S35. (A) Difference absorption spectra obtained after 460 nm laser excitation (1 Hz, 10 mJ/pulse) of a homogeneous solution containing **RuPS$_W$** and **CoP$_W$**. (B and C) Transient absorption kinetic traces collected at 470 nm after 460 nm laser excitation (1 Hz, 10 mJ/pulse) at 100 μs and 2000 μs timescale, respectively. Experimental homogeneous conditions: [**RuPS$_W$**] = 30 μM, [**CoP$_W$**] = 10 μM, 0.1 M sodium ascorbate, Ar-saturated 0.1 M $NaHCO_3$.

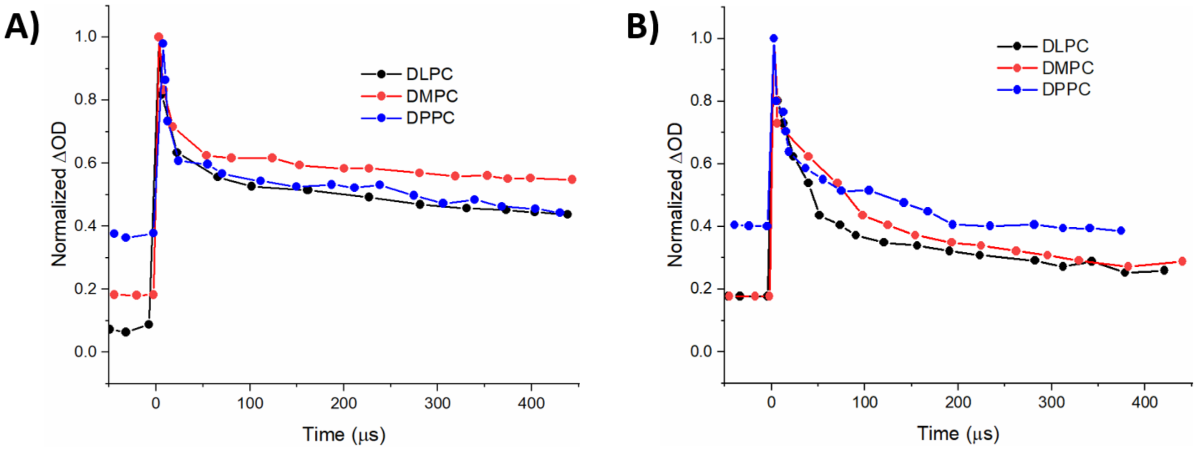

Figure S36. Comparison of normalized kinetic traces at 500 nm obtained from liposomes containing DLPC, DMPC and DPPC. Experimental conditions: (A) [Lipid] = 100 μM, [NaDSPE-PEG2K] = 1 μM, [$RuPS_L$] = 10 μM, 0.1 M sodium ascorbate, Ar-saturated 0.1 M $NaHCO_3$. (B) Lipid] = 100 μM, [NaDSPE-PEG2K] = 1 μM, [$RuPS_L$] = 10 μM, [NiT_L] = 2 μM, 0.1 M sodium ascorbate, Ar-saturated 0.1 M $NaHCO_3$. N.B. for further details see Supplementary Note 1 in section above.

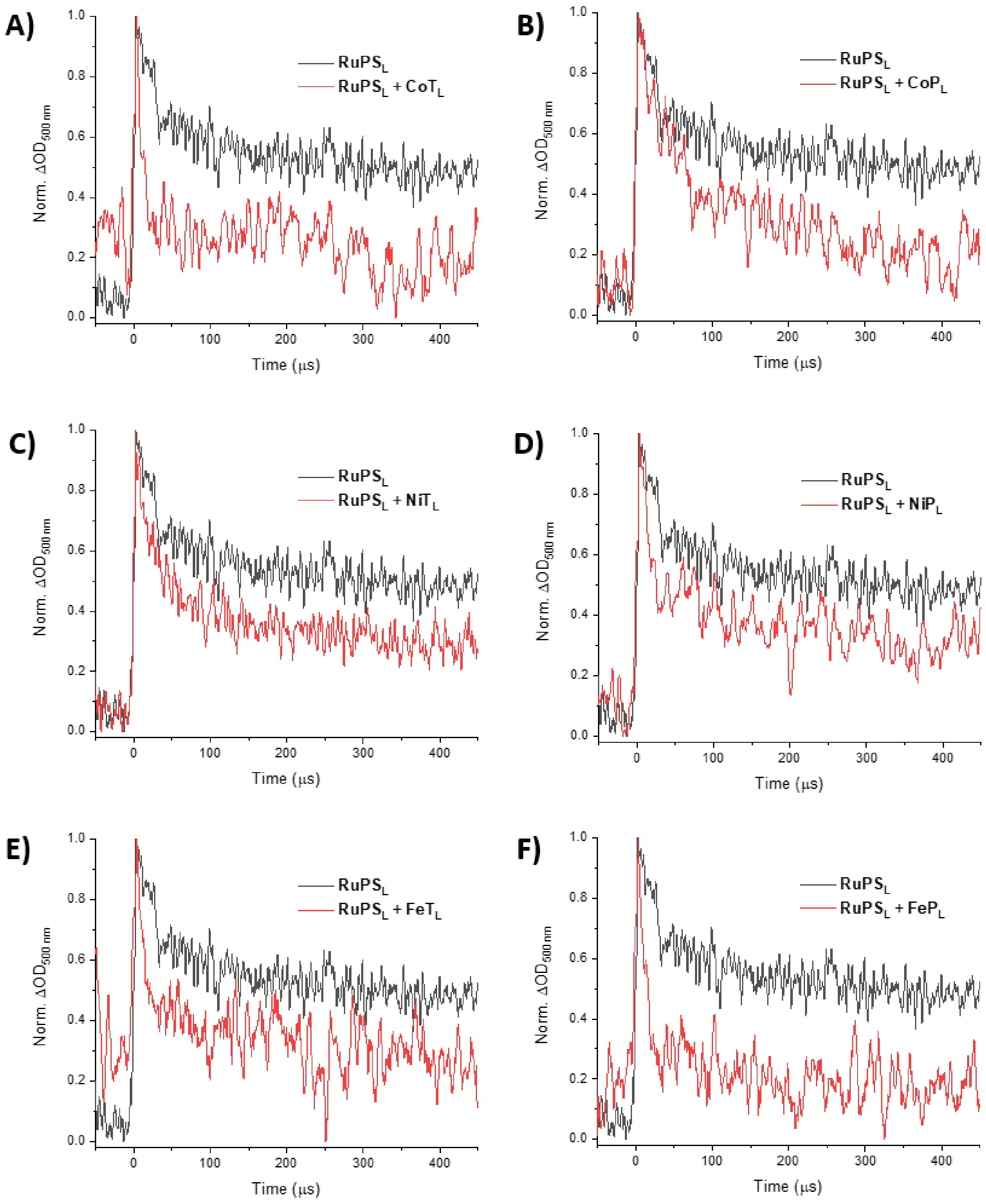

Figure S37. Normalized kinetic traces at 500 nm (original ΔOD ≈ 0.002) of **RuPS**$_L$ without alkylated catalyst (black line) and **RuPS**$_L$ with one of the six different alkylated catalysts (red line) in DMPC liposomes. Experimental conditions: [DMPC]=100 μM, [NaDSPE-PEG2K] =1 μM, [**RuPS**$_L$] = 10 μM, [**CoT**$_L$] = [**NiT**$_L$] = [**FeT**$_L$] = [**CoP**$_L$] = [**NiP**$_L$] = [**FeP**$_L$] = 500 nM and 0.1 M sodium ascorbate in 0.1 M $NaHCO_3$ aq. buffer.

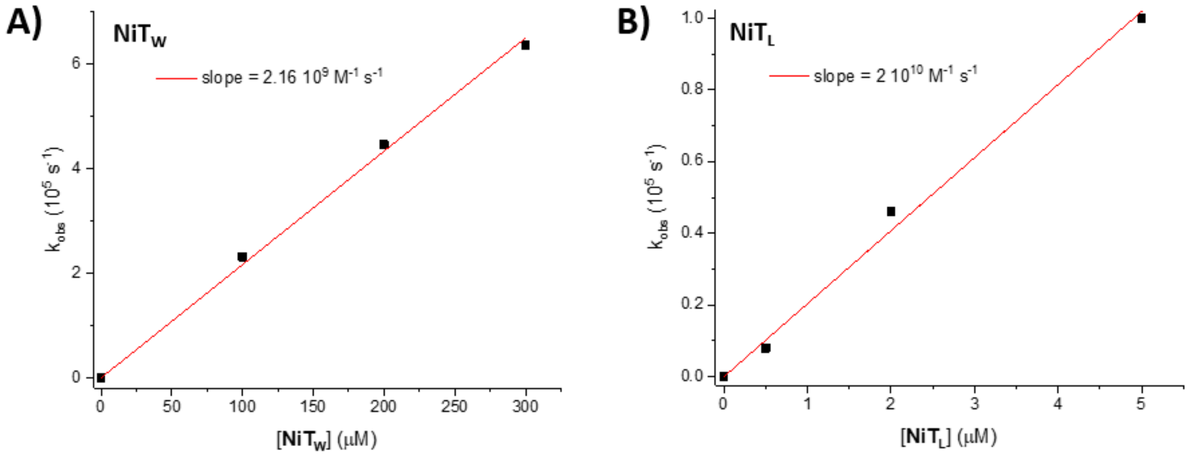

Figure S38. Observed rate constants (A) k_{obs} vs [**NiT$_W$**] or (B) K_{obs} vs [**NiT$_L$**], employed to calculate the bimolecular electron transfer rate constants K_{ET} (i.e. slope). Experimental homogeneous conditions (A): [**RuPS$_W$**] = 30 µM and 0.1 M NaHAsc in Ar-saturated 0.1 M $NaHCO_3$ aq. buffer. Experimental liposome conditions: [DMPC]=100 µM, [NaDSPE-PEG2K] =1 µM, [**RuPS$_L$**] = 10 µM and 0.1 M NaHAsc in Ar-saturated 0.1 M $NaHCO_3$ aq. buffer.

Square wave voltammetry and chronoamperometry

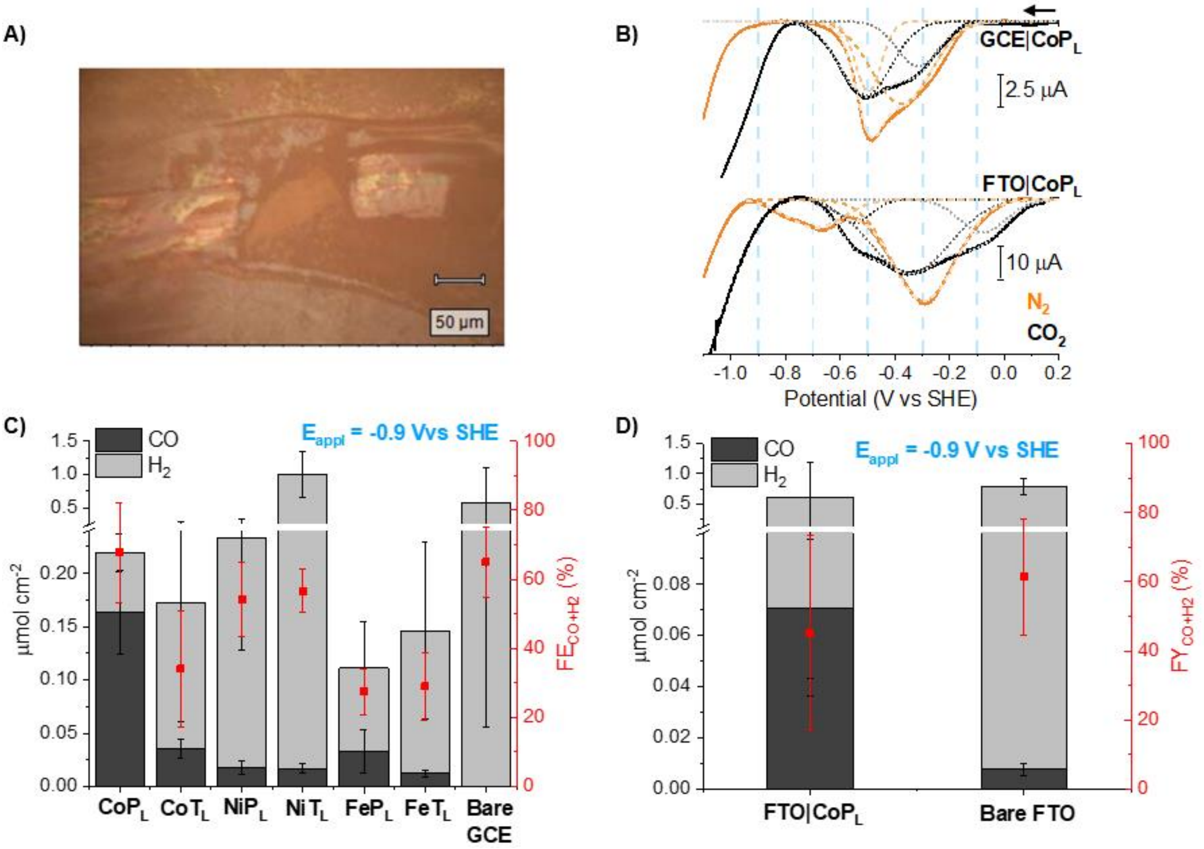

Figure S39. (A) Optical microscope image of a **CoP$_L$** film on FTO. (B) SWV scans comparing **CoP$_L$** dropcasted on glassy carbon electrode (GCE) (top) and fluorine-doped tin oxide (FTO) (bottom) in N_2- and CO_2-saturated 0.1 M $NaHCO_3$. (C) Chronoamperometry results of all alkylated catalysts dropcasted on GCE and bare GCE (no Cat., i.e. without catalysts) at E_{appl} = –0.9 V vs SHE for 4 h in CO_2-saturated 0.1 M $NaHCO_3$. (D) Chronoamperometry results of FTO|**CoP$_L$** and bare FTO (no **CoP$_L$**) conducted at an applied potential (E_{appl}) of –0.9 V vs SHE for four hours in CO_2-saturated 0.1 M $NaHCO_3$ solution (pH = 6.7) at room temperature. N.B. for further details see Supplementary Note in section above.

UV-vis-NIR spectroelectrochemistry

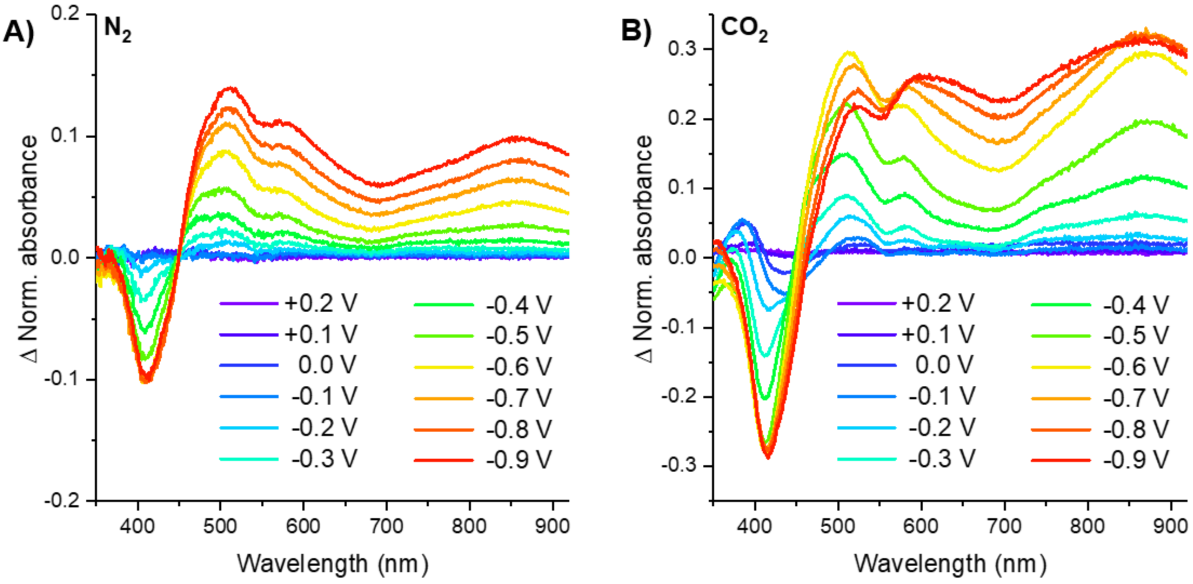

Figure S40. Variation UV-vis-NIR SEC of **CoP$_L$** dropcasted on FTO in (A) N_2- and (B) CO_2-saturated 0.1 M $NaHCO_3$.

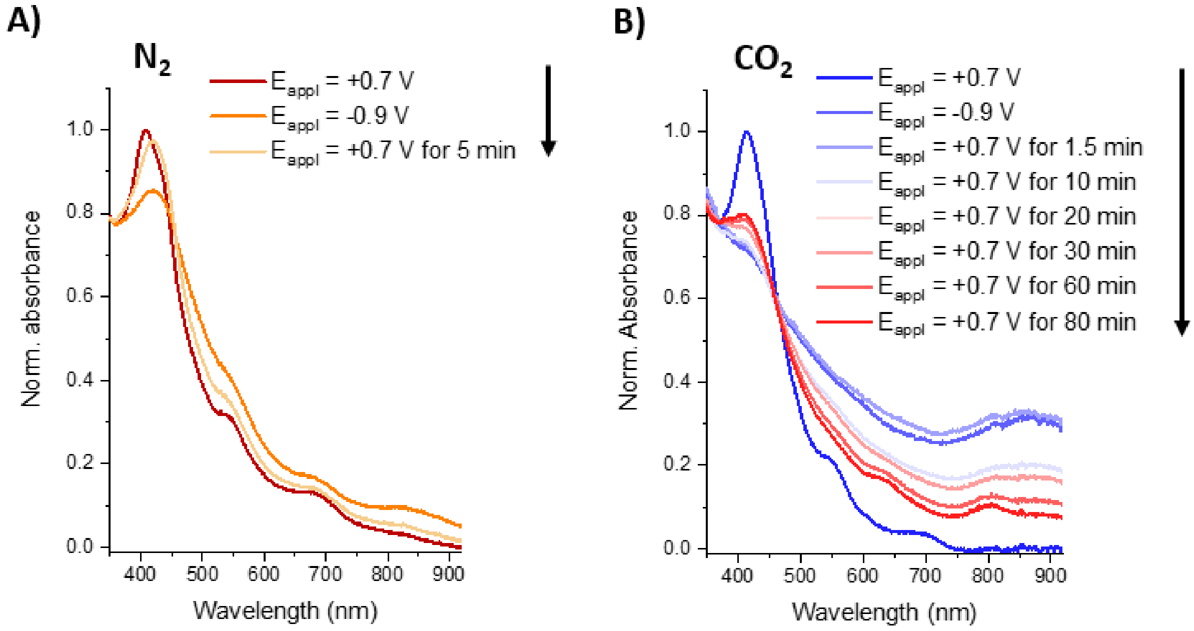

Figure S41. UV-vis-NIR SEC of re-oxidized **CoP$_L$** films on FTO, after E_{appl} = –0.9 V was applied for one minute, in (A) N_2- and (B) CO_2-saturated 0.1 M $NaHCO_3$. Note: further oxidation of reduced **CoP$_L$** film in the presence of N_2 (A) did not lead to further changes in the absorption features of the film. N.B. for further details see Supplementary Note 6 in section above.

Resonance Raman spectroelectrochemistry

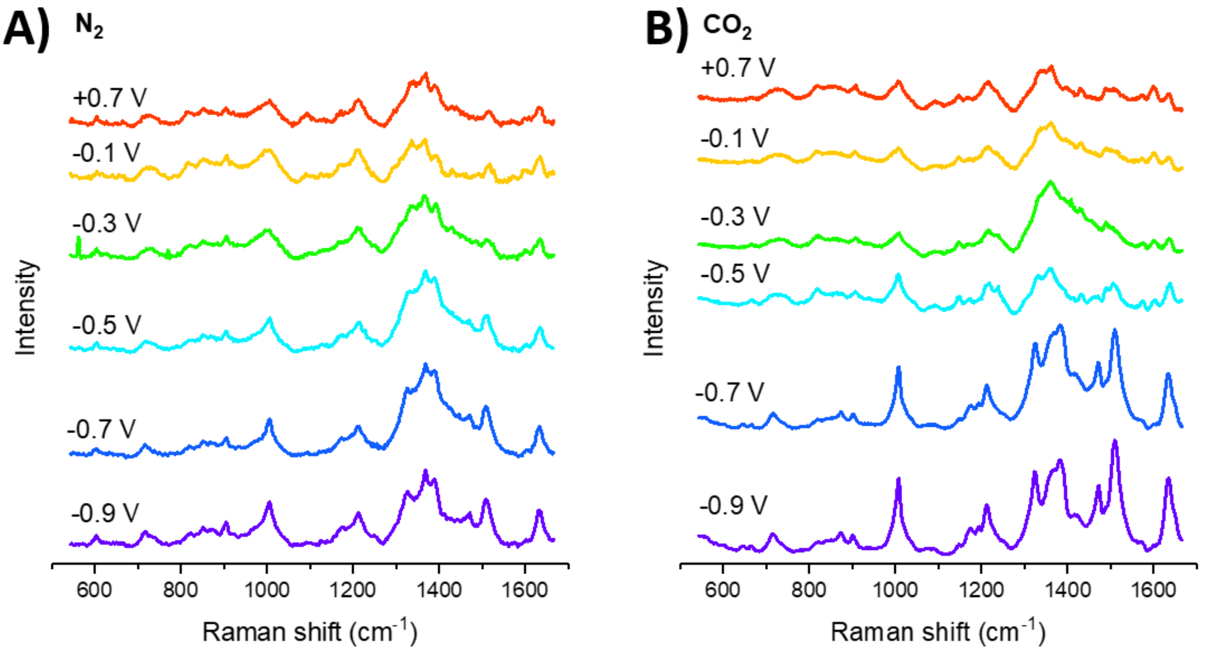

Figure S42. Resonance Raman SEC in (A) N_2- and (B) CO_2-saturated 0.1 M $NaHCO_3$.

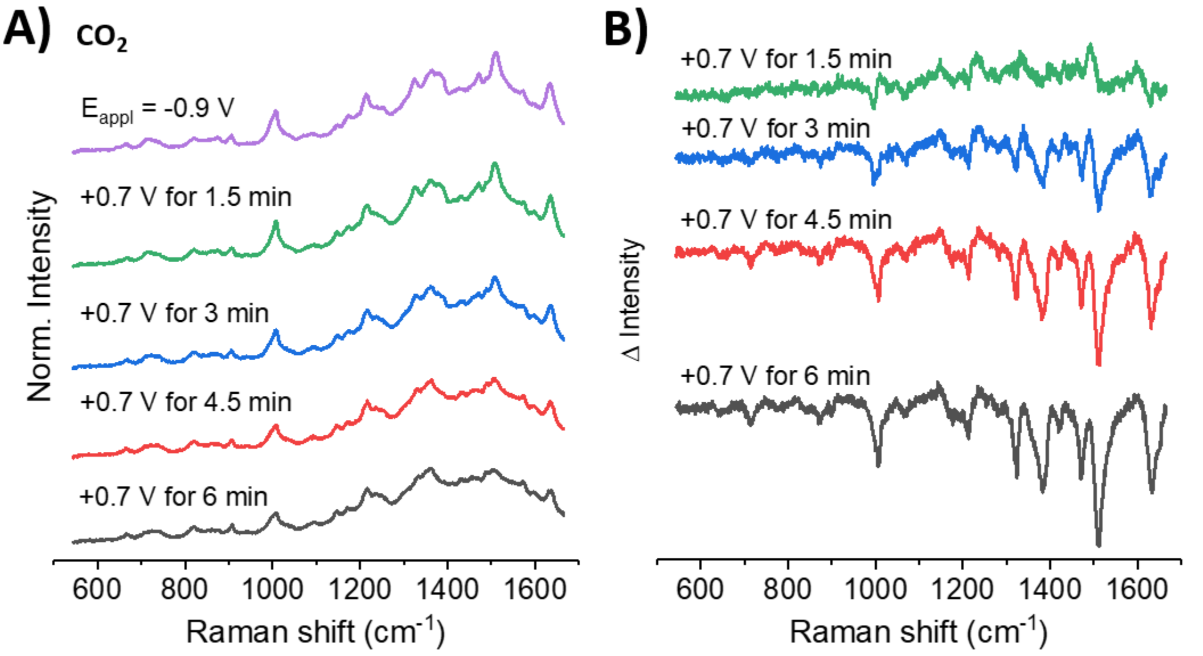

Figure S43. (A) Resonance Raman SEC of re-oxidized **CoP_L** films on FTO, after E_{appl} = –0.9 V vs SHE was applied for one minute, in CO_2-saturated 0.1 M $NaHCO_3$. (B) Difference Resonance Raman SEC of the same spectra shown in plot A. The difference spectra were obtained by subtracting the reduced species spectrum (–0.9 V) from each spectrum. NB. for further details about these results see above Supplementary Note 6.

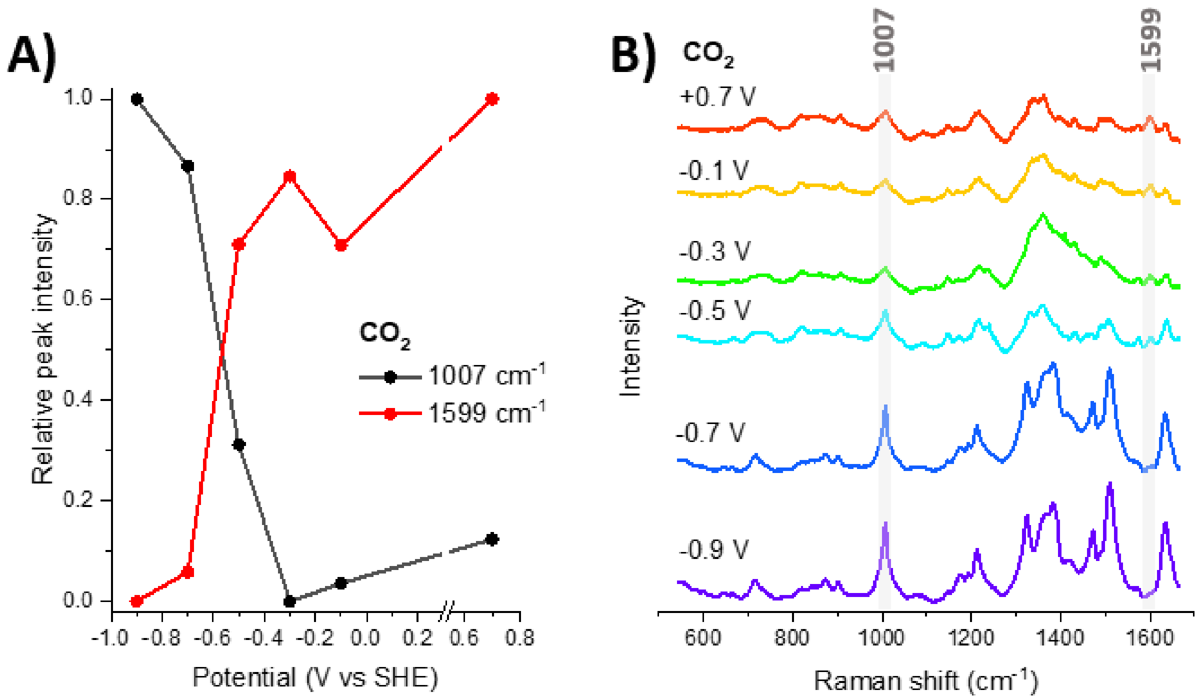

Figure S44. (A) Relative peak intensity vs applied potential for peaks at 1007 and 1599 cm^{-1} and (B) Raman SEC highlighting the two monitored peaks in CO_2-saturated 0.1 M $NaHCO_3$.

DFT calculated Raman spectra

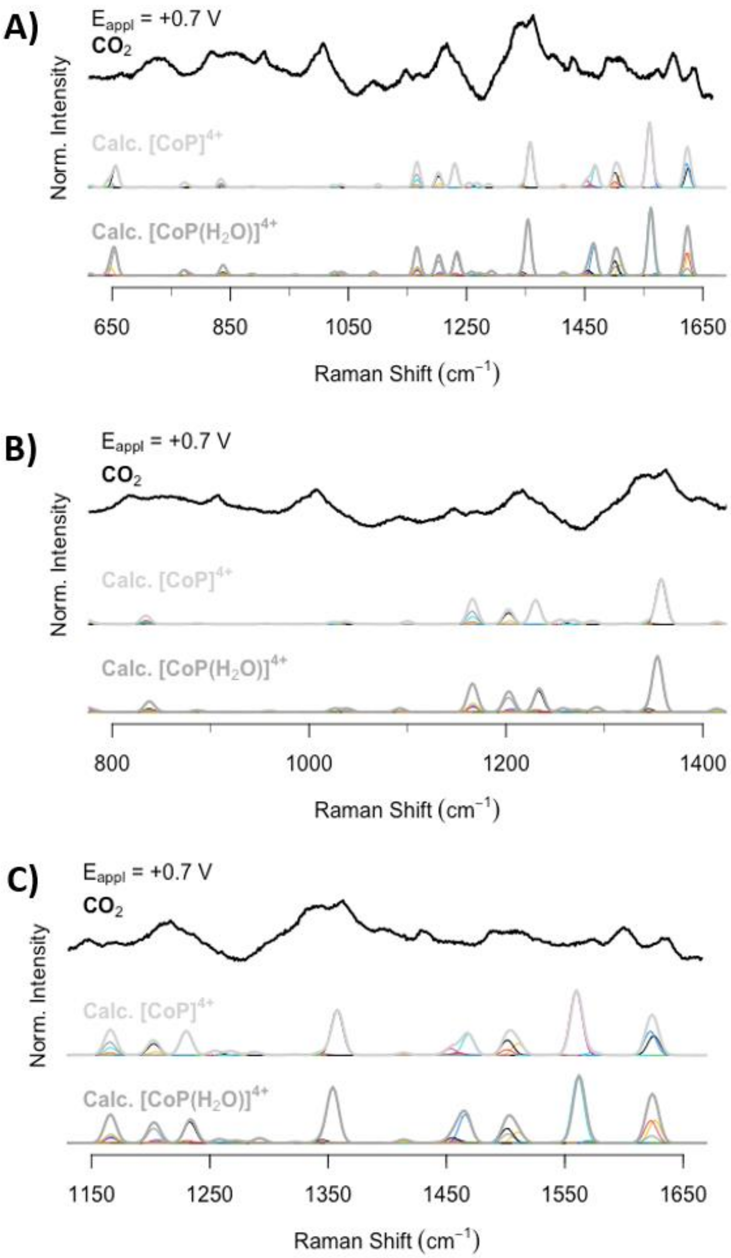

Figure S45. Comparison of (A) experimental spectrum at +0.7 V vs SHE and calculated Raman spectra and (B and C) zoom-in of selected calculated Raman frequencies of **[CoP]$^{4+}$** and **[CoP(H$_2$O)]$^{4+}$**. The experimental Raman spectra corresponds to FTO|**CoP$_L$** at +0.7 V vs SHE in CO_2-saturated 0.1 M $NaHCO_3$. N.B. for further details see Supplementary Note 7 in section above.

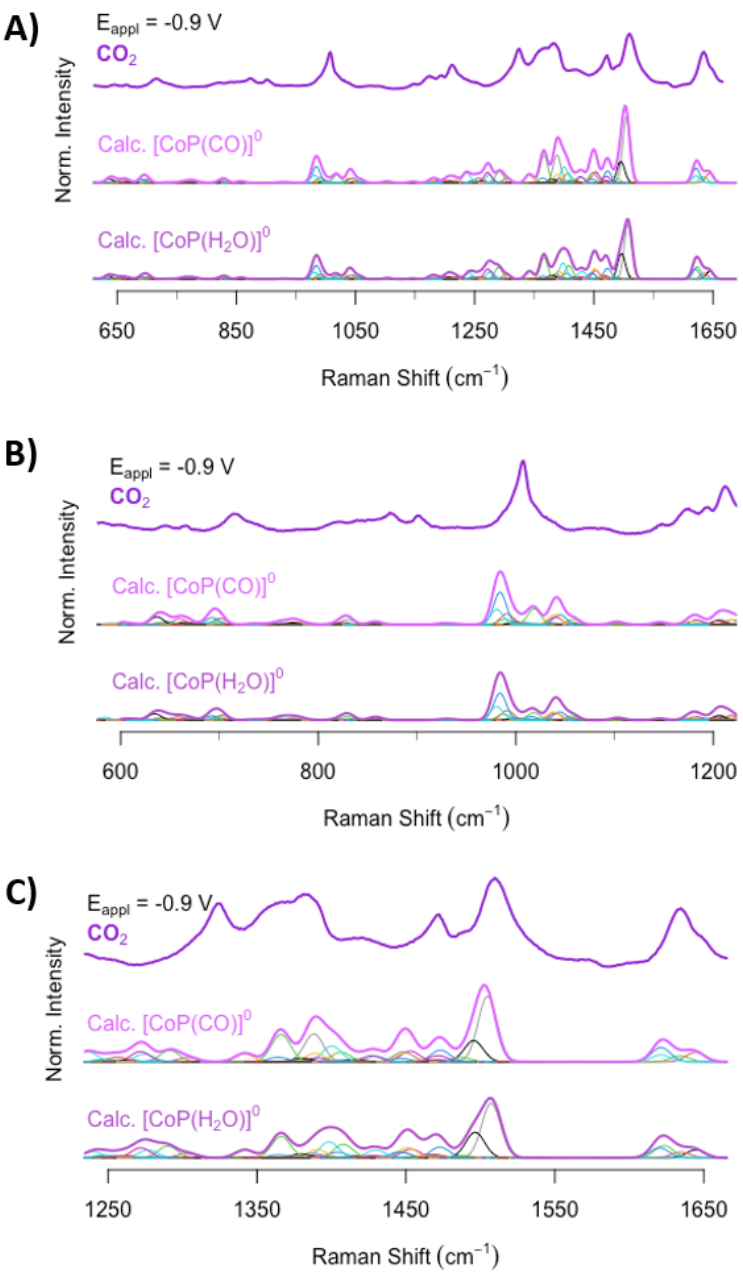

Figure S46. Comparison of (A) experimental spectrum at –0.9 V vs SHE and calculated Raman spectra and (B and C) zoom-in of selected calculated Raman frequencies of **[CoP(H$_2$O)]^0** and **[CoP(CO)]^0**. The experimental Raman spectra corresponds to FTO|**CoP$_L$** at –0.9 V vs SHE in CO_2-saturated 0.1 M $NaHCO_3$. N.B. for further details see Supplementary Note 7 in section above.

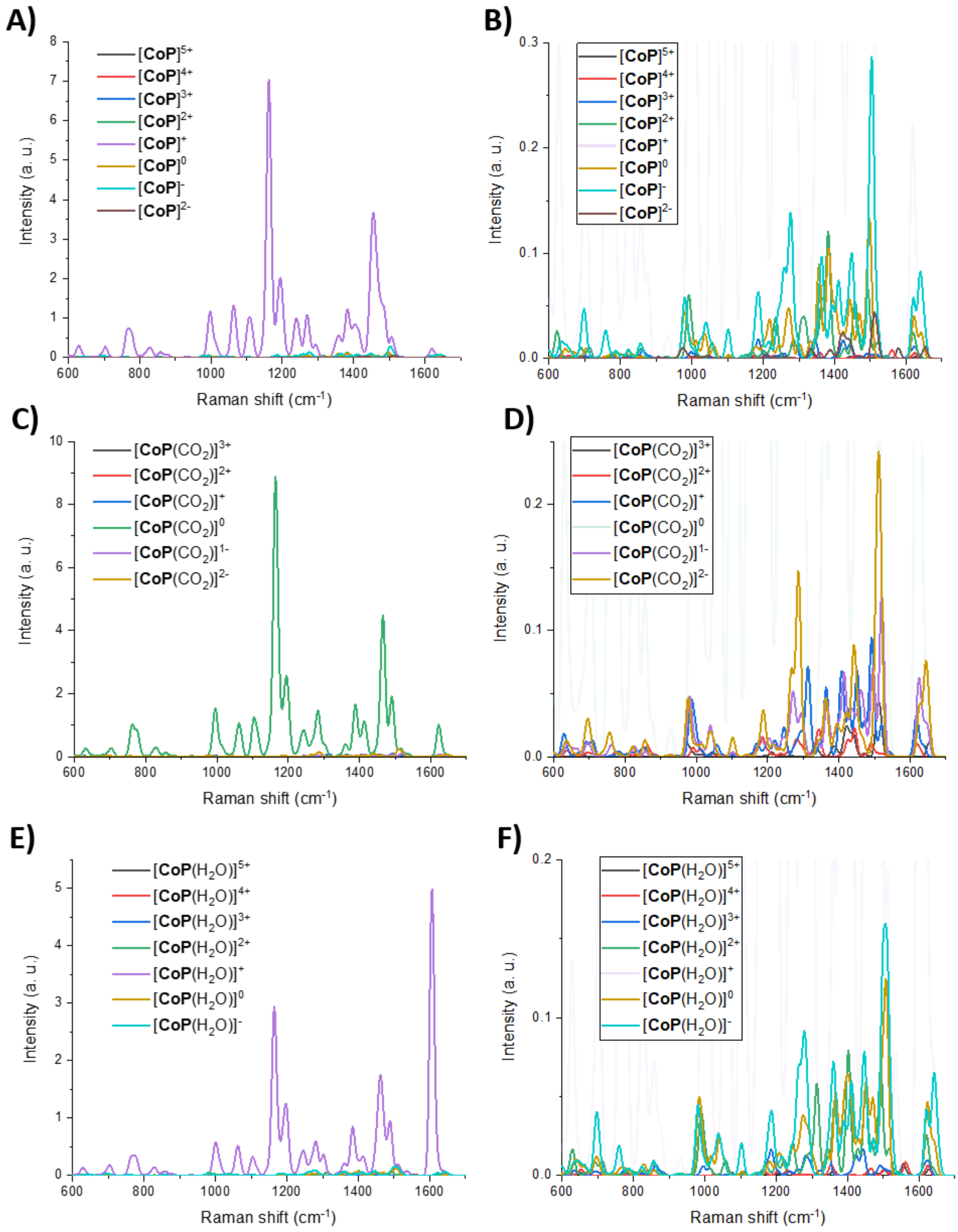

Figure S47. Calculated Raman spectra (left column: full intensity range; right column: zoom-in) for different oxidation states of (A and B) adduct-free [**CoP**]^n, (C and D) [**CoP**(CO_2)]^n and (E and F) [**CoP**(H_2O)]^n. Oxidation state *n* can range from +5 to –2. In all right columns, to aid visualisation the most intense spectrum is shown 75 % more transparent.

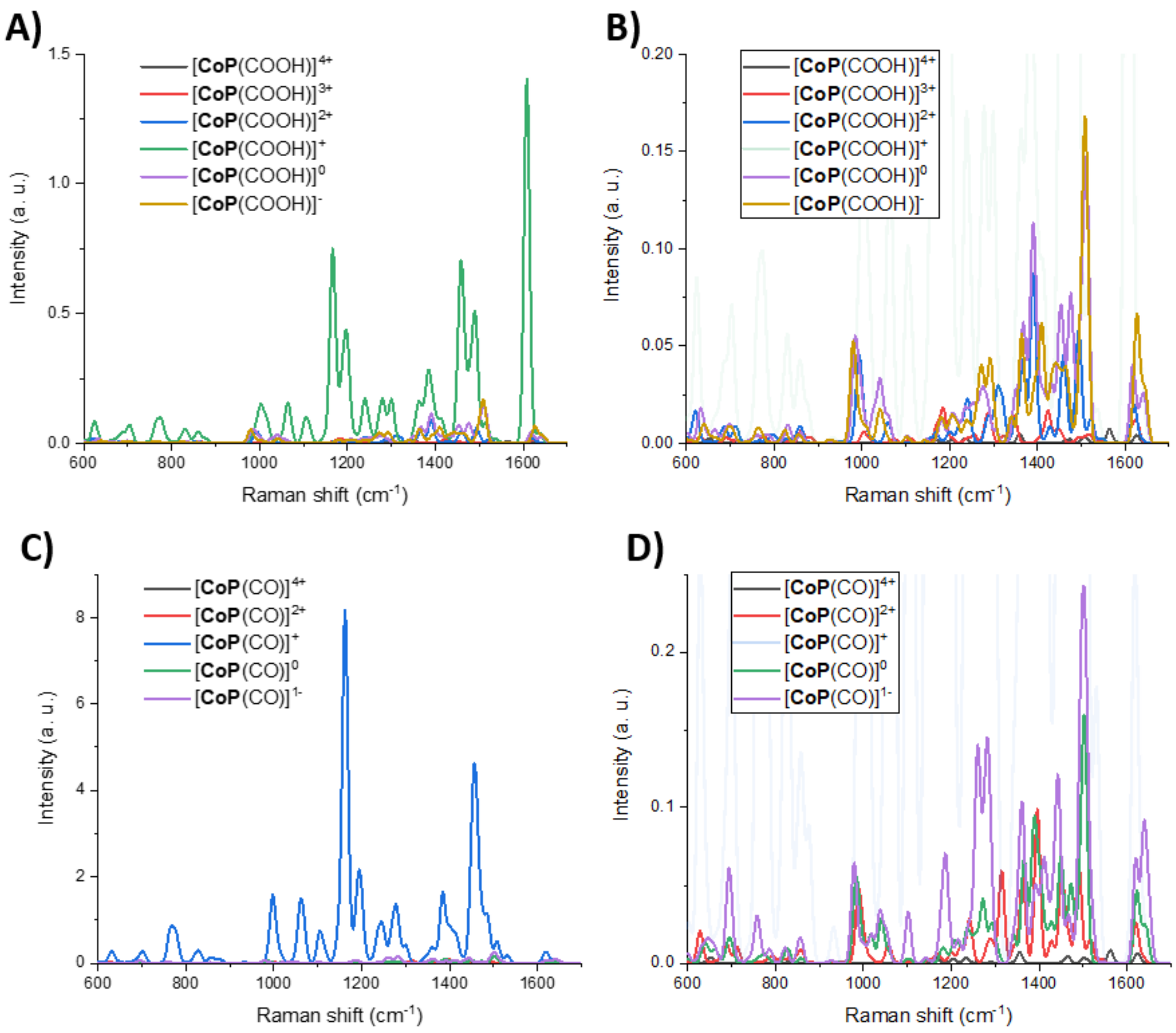

Figure S48. Calculated Raman spectra (left column: full intensity range; right column: zoom-in) for different oxidation states of (A and B) [**CoP**(COOH)]^n and (C and D) [**CoP**(CO)]^n. Oxidation state n can range from +4 to –1. In all right columns, to aid visualisation the most intense spectrum is shown 75 % more transparent.

Calculated structures of proposed reaction intermediates using model catalyst CoP

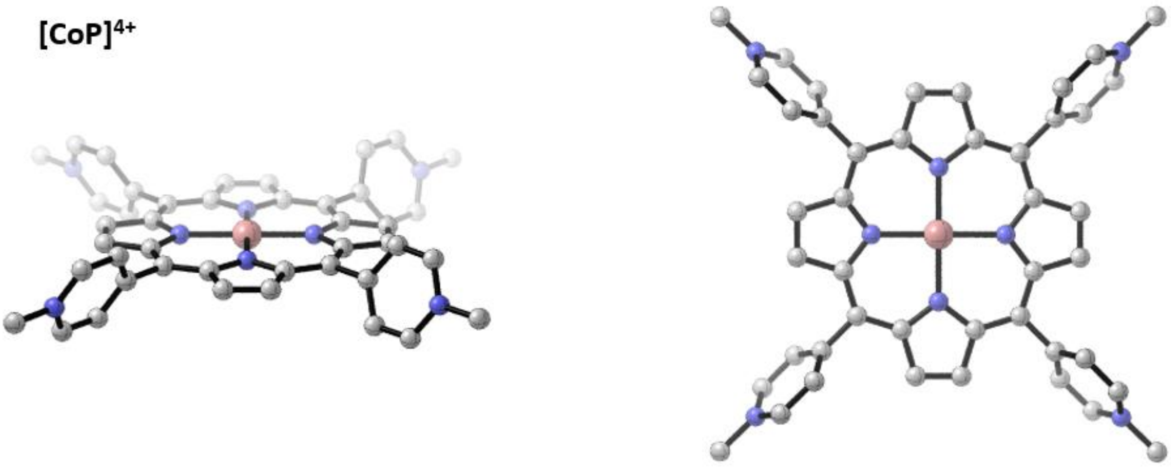

Figure S49. Side and top views of calculated structure of **[CoP]$^{4+}$**.

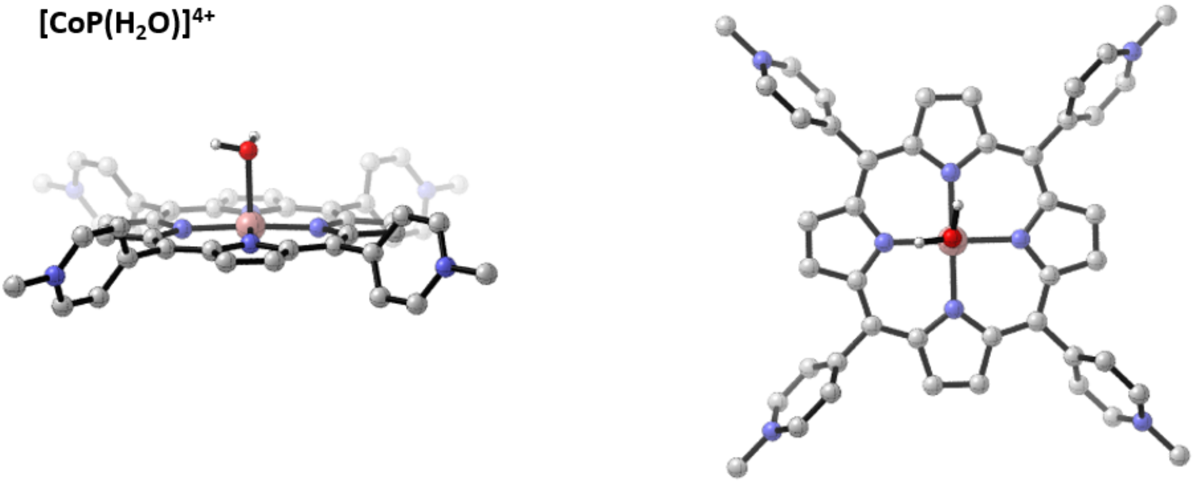

Figure S50. Side and top views of calculated structure of **[CoP(H$_2$O)]$^{4+}$**.

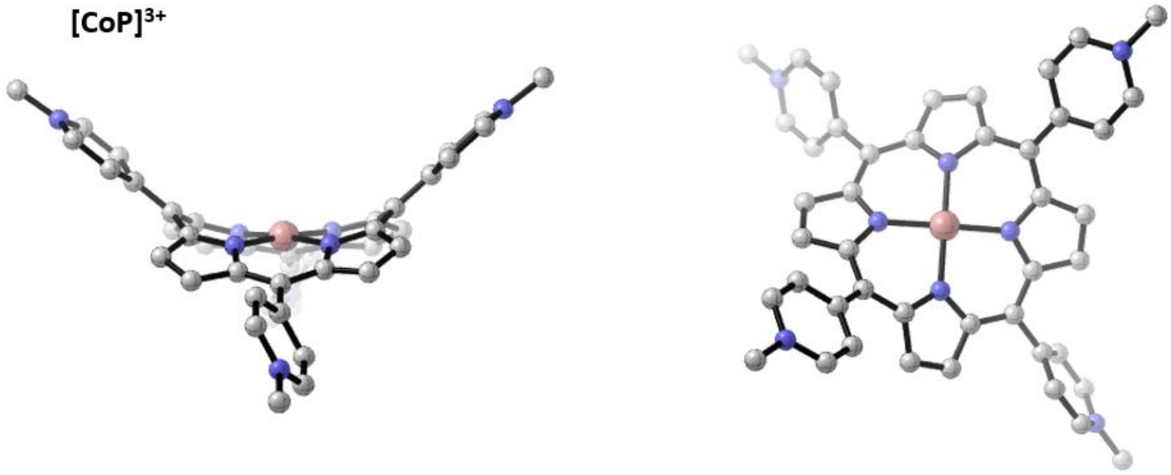

Figure S51. Side and top views of calculated structure of **$[CoP]^{3+}$**.

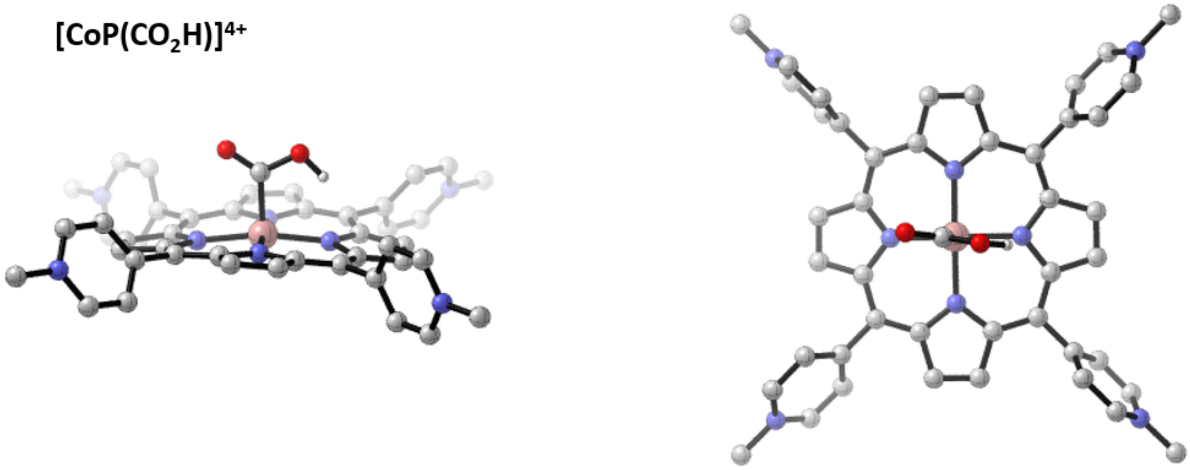

Figure S52. Side and top views of calculated structure of **$[CoP(CO_2H)]^{4+}$**.

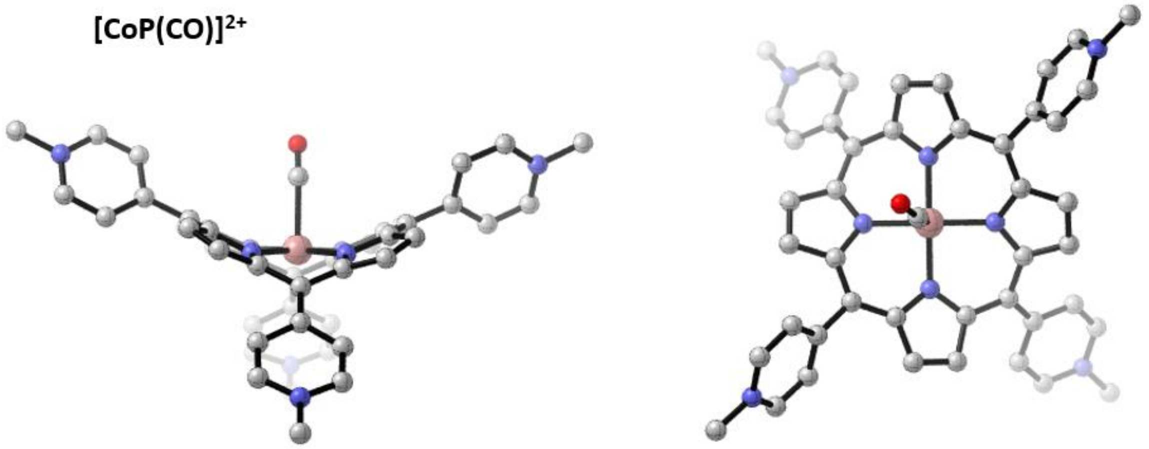

Figure S53. Side and top views of calculated structure of **[CoP(CO)]$^{2+}$**.

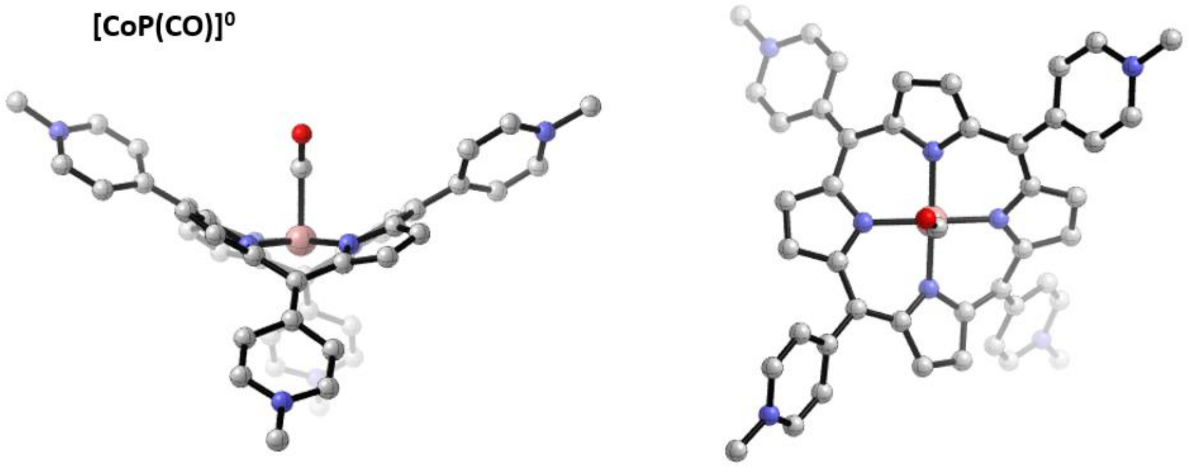

Figure S54. Side and top views of calculated structure of **[CoP(CO)]^{0}**.

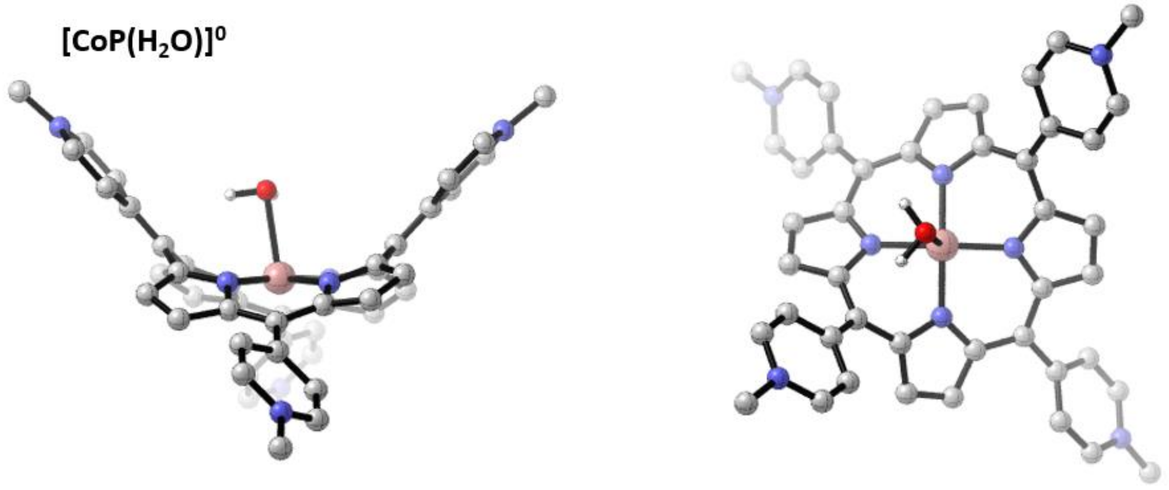

Figure S55. Side and top views of calculated structure of **[CoP(H_2O)]^0**

References

1. Sekiya, R.; Tsutsui, Y.; Choi, W.; Sakurai, T.; Seki, S.; Bando, Y.; Maeda, H., Ion-based assemblies of planar anion complexes and cationic Pt^{II} complexes. *Chem. Comm.* **2014,** *50*, 10615-10618.
2. Nielsen, P.; Toftlund, H.; Bond, A. D.; Boas, J. F.; Pilbrow, J. R.; Hanson, G. R.; Noble, C.; Riley, M. J.; Neville, S. M.; Moubaraki, B.; Murray, K. S., Systematic study of spin crossover and structure in $[Co(terpyRX)_2](Y)_2$ systems (terpyRX = 4'-alkoxy-2,2':6',2''-terpyridine, X = 4, 8, 12, Y = BF_4^-, ClO_4^-, PF_6^-, BPh_4^-). *Inorg. Chem.* **2009,** *48*, 7033-7047.
3. Elgrishi, N.; Chambers, M. B.; Artero, V.; Fontecave, M., Terpyridine complexes of first row transition metals and electrochemical reduction of CO_2 to CO. *Phys. Chem. Chem. Phys.* **2014,** *16*, 13635-13644.
4. Zhang, X.; Cibian, M.; Call, A.; Yamauchi, K.; Sakai, K., Photochemical CO_2 Reduction Driven by Water-Soluble Copper(I) Photosensitizer with the Catalysis Accelerated by Multi-Electron Chargeable Cobalt Porphyrin. *ACS Catal.* **2019,** *9*, 11263-11273.
5. Klein, D. M.; Rodríguez-Jiménez, S.; Hoefnagel, M. E.; Pannwitz, A.; Prabhakaran, A.; Siegler, M. A.; Keyes, T. E.; Reisner, E.; Brouwer, A. M.; Bonnet, S., Shorter Alkyl Chains Enhance Molecular Diffusion and Electron Transfer Kinetics Between Photosensitisers and Catalysts in CO_2-Reducing Photocatalytic Liposomes. *Chem. Eur. J.* **2021,** *27*, 17203.
6. Andersson, M.; Hammarstroem, L.; Edwards, K., Effect of Bilayer Phase Transitions on Vesicle Structure, and its Influence on the Kinetics of Viologen Reduction. *J. Phys. Chem.* **1995,** *99*, 14531-14538.
7. Olesund, A.; Gray, V.; Mårtensson, J.; Albinsson, B., Diphenylanthracene Dimers for Triplet–Triplet Annihilation Photon Upconversion: Mechanistic Insights for Intramolecular Pathways and the Importance of Molecular Geometry. *J. Am. Chem. Soc.* **2021,** *143*, 5745-5754.
8. Götz, R.; Ly, H. K.; Wrzolek, P.; Schwalbe, M.; Weidinger, I. M., Surface enhanced resonance Raman spectroscopy of iron Hangman complexes on electrodes during electrocatalytic oxygen reduction: advantages and problems of common drycast methods. *Dalton Trans.* **2017,** *46*, 13220-13228.
9. Marianov, A. N.; Kochubei, A. S.; Roman, T.; Conquest, O. J.; Stampfl, C.; Jiang, Y., Resolving Deactivation Pathways of Co Porphyrin-Based Electrocatalysts for CO_2 Reduction in Aqueous Medium. *ACS Catal.* **2021,** *11*, 3715-3729.
10. Jiang, J.; Matula, A. J.; Swierk, J. R.; Romano, N.; Wu, Y.; Batista, V. S.; Crabtree, R. H.; Lindsey, J. S.; Wang, H.; Brudvig, G. W., Unusual Stability of a Bacteriochlorin Electrocatalyst under Reductive Conditions. A Case Study on CO_2 Conversion to CO. *ACS Catal.* **2018,** *8*, 10131-10136.
11. Terekhov, S. N.; Kruglik, S. G.; Malinovskii, V. L.; Galievsky, V. A.; Chirvony, V. S.; Turpin, P.-Y., Resonance Raman characterization of cationic Co(II) and Co(III) tetrakis(N-methyl-4-pyridinyl)porphyrins in aqueous and non-aqueous media. *J. Raman. Spectrosc.* **2003,** *34*, 868-881.
12. Orendorff, C. J.; Ducey, M. W.; Pemberton, J. E., Quantitative Correlation of Raman Spectral Indicators in Determining Conformational Order in Alkyl Chains. *J. Phys. Chem. A.* **2002,** *106*, 6991-6998.
13. Elgrishi, N.; Chambers, M. B.; Fontecave, M., Turning it off! Disfavouring hydrogen evolution to enhance selectivity for CO production during homogeneous CO_2 reduction by cobalt–terpyridine complexes. *Chem. Sci.* **2015,** *6*, 2522-2531.

14. Kuehnel, M. F.; Orchard, K. L.; Dalle, K. E.; Reisner, E., Selective photocatalytic CO_2 reduction in water through anchoring of a molecular Ni catalyst on CdS nanocrystals. *J. Am. Chem. Soc.* **2017,** *139*, 7217-7223.
15. Araullo-McAdams, C.; Kadish, K. M., Electrochemistry, spectroscopy, and reactivity of (meso-tetrakis(1-methylpyridinium-4-yl)porphinato)cobalt(III,II,I) in nonaqueous media. *Inorg. Chem.* **1990,** *29*, 2749-2757.
16. Han, Y.; Wu, Y.; Lai, W.; Cao, R., Electrocatalytic Water Oxidation by a Water-Soluble Nickel Porphyrin Complex at Neutral pH with Low Overpotential. *Inorg. Chem.* **2015,** *54*, 5604-5613.
17. Pannwitz, A.; Klein, D. M.; Rodríguez-Jiménez, S.; Casadevall, C.; Song, H.; Reisner, E.; Hammarström, L.; Bonnet, S., Roadmap towards solar fuel synthesis at the water interface of liposome membranes. *Chem. Soc. Rev.* **2021,** *50*, 4833-4855.
18. Arcudi, F.; Đorđević, L.; Nagasing, B.; Stupp, S. I.; Weiss, E. A., Quantum Dot-Sensitized Photoreduction of CO_2 in Water with Turnover Number > 80,000. *J. Am. Chem. Soc.* **2021,** *143*, 18131-18138.
19. Chaudhary, Y. S.; Woolerton, T. W.; Allen, C. S.; Warner, J. H.; Pierce, E.; Ragsdale, S. W.; Armstrong, F. A., Visible light-driven CO_2 reduction by enzyme coupled CdS nanocrystals. *Chem. Comm.* **2012,** *48*, 58-60.
20. Zhang, X.; Yamauchi, K.; Sakai, K., Earth-Abundant Photocatalytic CO_2 Reduction by Multielectron Chargeable Cobalt Porphyrin Catalysts: High CO/H_2 Selectivity in Water Based on Phase Mismatch in Frontier MO Association. *ACS Catal.* **2021**, 10436-10449.
21. Call, A.; Cibian, M.; Yamauchi, K.; Sakai, K., Visible-light-driven reduction of CO2 to CO in fully aqueous media using a water-soluble cobalt porphyrin. *Sustain. Energy Fuels* **2022**.
22. Woolerton, T. W.; Sheard, S.; Reisner, E.; Pierce, E.; Ragsdale, S. W.; Armstrong, F. A., Efficient and clean photoreduction of CO_2 to CO by enzyme-modified TiO_2 nanoparticles using visible light. *J. Am. Chem. Soc.* **2010,** *132*, 2132-2133.
23. Bi, Q.-Q.; Wang, J.-W.; Lv, J.-X.; Wang, J.; Zhang, W.; Lu, T.-B., Selective Photocatalytic CO2 Reduction in Water by Electrostatic Assembly of CdS Nanocrystals with a Dinuclear Cobalt Catalyst. *ACS Catal.* **2018,** *8*, 11815-11821.
24. Call, A.; Cibian, M.; Yamamoto, K.; Nakazono, T.; Yamauchi, K.; Sakai, K., Highly Efficient and Selective Photocatalytic CO_2 Reduction to CO in Water by a Cobalt Porphyrin Molecular Catalyst. *ACS Catal.* **2019,** *9*, 4867-4874.
25. Sahm, C. D.; Ucoski, G. M.; Roy, S.; Reisner, E., Automated and Continuous-Flow Platform to Analyze Semiconductor–Metal Complex Hybrid Systems for Photocatalytic CO_2 Reduction. *ACS Catal.* **2021,** *11*, 11266-11277.
26. Lian, S.; Kodaimati, M. S.; Weiss, E. A., Photocatalytically Active Superstructures of Quantum Dots and Iron Porphyrins for Reduction of CO_2 to CO in Water. *ACS Nano* **2018,** *12*, 568-575.
27. Wang, Q.; Warnan, J.; Rodríguez-Jiménez, S.; Leung, J. J.; Kalathil, S.; Andrei, V.; Domen, K.; Reisner, E., Molecularly engineered photocatalyst sheet for scalable solar formate production from carbon dioxide and water. *Nat. Energy* **2020**.
28. Kuehnel, M. F.; Sahm, C. D.; Neri, G.; Lee, J. R.; Orchard, Katherine L.; Cowan, A. J.; Reisner, E., ZnSe quantum dots modified with a Ni(cyclam) catalyst for efficient visible-light driven CO_2 reduction in water. *Chem. Sci.* **2018,** *9*, 2501-2509.
29. Ikuta, N.; Takizawa, S.-y.; Murata, S., Photochemical reduction of CO_2 with ascorbate in aqueous solution using vesicles acting as photocatalysts. *Photochem. Photobiol. Sci.* **2014,** *13*, 691-702.

30. Nakada, A.; Koike, K.; Maeda, K.; Ishitani, O., Highly efficient visible-light-driven CO_2 reduction to CO using a Ru(ii)–Re(i) supramolecular photocatalyst in an aqueous solution. *Green Chem.* **2016,** *18*, 139-143.
31. Méndez, M. A.; Voyame, P.; Girault, H. H., Interfacial photoreduction of supercritical CO_2 by an aqueous catalyst. *Angew. Chem. Int. Ed.* **2011,** *50*, 7391-4.
32. Grant, J. L.; Goswami, K.; Spreer, L. O.; Otvos, J. W.; Calvin, M., Photochemical reduction of carbon dioxide to carbon monoxide in water using a nickel(II) tetra-azamacrocycle complex as catalyst. *J. Chem. Soc., Dalton Trans.* **1987**, 2105.
33. Miyamoto, K.; Asahi, R., Water Facilitated Electrochemical Reduction of CO2 on Cobalt-Porphyrin Catalysts. *J. Phys. Chem. C.* **2019,** *123*, 9944-9948.
34. Kelly, C. P.; Cramer, C. J.; Truhlar, D. G., Aqueous Solvation Free Energies of Ions and Ion−Water Clusters Based on an Accurate Value for the Absolute Aqueous Solvation Free Energy of the Proton. *J. Phys. Chem. B.* **2006,** *110*, 16066-16081.
35. Casasnovas, R.; Ortega-Castro, J.; Frau, J.; Donoso, J.; Muñoz, F., Theoretical pKa calculations with continuum model solvents, alternative protocols to thermodynamic cycles. *Int. J. Quantum Chem.* **2014,** *114*, 1350-1363.

End of Supporting Information
