# Supplementary material for: Self-Assembled Liposomes Enhance Electron Transfer for Efficient Photocatalytic CO2 Reduction
Source: J Am Chem Soc. 2022 May 20;144(21):9399–412. doi: 10.1021/jacs.2c01725 (PMC9164230; doi:10.1021/jacs.2c01725)
Supplement: Supplementary file 2 — ja2c01725_si_002.zip [file ja2c01725_si_002.zip › Coordinates/CoP/Coord_CoP.docx]

[CoP]^+5^ M = 1

C -1.1995909 -2.7896813 -0.0680157

C -0.8526939 -4.1962353 -0.0361197

C 0.4951951 -4.2612273 0.0770323

C 0.9809791 -2.8949343 0.0773573

N -0.0899529 -2.0077133 -0.0049727

C -2.5356619 -2.3133403 -0.1466937

C 2.3121001 -2.5389643 0.1373363

C 2.7799321 -1.2006263 0.0663053

C 4.1915301 -0.8573213 0.0168783

C 4.2578601 0.4869717 -0.0968497

C 2.8864241 0.9731127 -0.1106897

N 1.9978331 -0.0942153 -0.0117597

C 2.5396931 2.3063017 -0.1554537

C 1.2035911 2.7826257 -0.0748567

C 0.8567251 4.1892707 -0.0451517

C -0.4909079 4.2545257 0.0706633

C -0.9766379 2.8882747 0.0748283

N 0.0941221 2.0008537 -0.0079867

C -2.3075939 2.5324647 0.1385673

C -2.7754679 1.1940207 0.0713603

C -4.1871129 0.8506197 0.0261313

C -4.2536499 -0.4938043 -0.0857037

C -2.8821849 -0.9799563 -0.1023687

N -1.9934849 0.0874927 -0.0069727

Co 0.0021381 -0.0034093 -0.0070647

H -1.5489539 -5.0195393 -0.0885317

H 1.1097471 -5.1463323 0.1464383

H 5.0107211 -1.5593783 0.0562533

H 5.1415471 1.1049217 -0.1524707

H 1.5526191 5.0126537 -0.1009557

H -1.1052499 5.1398277 0.1394023

H -5.0062499 1.5526727 0.0668203

H -5.1376459 -1.1115933 -0.1380307

H -5.5067419 5.0431647 2.4534543

H -3.8274269 3.2045187 2.3281743

C -4.9520939 4.8363007 1.5470943

C -4.0191679 3.8207917 1.4580723

H -7.2051379 6.2396227 0.7865873

H -5.9636079 7.3294487 1.4783663

C -6.2307619 6.7034257 0.6264793

N -5.2125679 5.6337287 0.4838363

C -3.3287949 3.6159987 0.2544953

H -6.2473019 7.3020657 -0.2819177

C -4.5671169 5.4531477 -0.6889537

C -3.6209629 4.4513377 -0.8296487

H -4.8283939 6.1212327 -1.4988867

H -3.1232799 4.3283307 -1.7837227

H -5.9246119 -4.5733143 -2.4469677

H -4.0937789 -2.8826413 -2.3326957

C -5.3505029 -4.4122673 -1.5435307

C -4.3348809 -3.4781063 -1.4607087

H -7.7248269 -5.6028343 -0.6873917

H -6.6225059 -6.7548353 -1.5029047

C -6.7861679 -6.1537973 -0.6082457

N -5.6738859 -5.1805793 -0.4772787

C -3.6303939 -3.3228303 -0.2587917

H -6.8012789 -6.7960093 0.2700663

C -5.0114869 -5.0503923 0.6937663

C -3.9854919 -4.1305853 0.8283593

H -5.3274539 -5.6897893 1.5073963

H -3.4780119 -4.0467483 1.7816993

H 5.3138951 5.6967347 1.5009053

H 3.4759211 4.0367517 1.7763383

C 4.9999021 5.0530367 0.6892103

C 3.9833791 4.1263247 0.8234863

H 6.4158761 7.1277697 -0.1475267

H 7.6196101 5.8078317 -0.0226417

C 6.7585711 6.1859937 -0.5761967

N 5.6609921 5.1921527 -0.4842837

C 3.6326731 3.3176437 -0.2671047

H 7.0197831 6.3298557 -1.6226837

C 5.3459041 4.4225987 -1.5494177

C 4.3360191 3.4786657 -1.4664637

H 5.9153201 4.5852017 -2.4550367

H 4.0996571 2.8833357 -2.3398427

H 5.5180801 -5.0422243 2.4506503

H 3.8368591 -3.2053283 2.3249873

C 4.9610521 -4.8380413 1.5451353

C 4.0270341 -3.8235593 1.4559303

H 7.2146961 -6.2396423 0.7774523

H 5.9785411 -7.3274173 1.4819563

C 6.2399781 -6.7054483 0.6253673

N 5.2197101 -5.6376983 0.4830993

C 3.3337341 -3.6220673 0.2534753

H 6.2519581 -7.3087793 -0.2799947

C 4.5711571 -5.4605673 -0.6885077

C 3.6237781 -4.4599213 -0.8292897

H 4.8311471 -6.1303053 -1.4974907

H 3.1236841 -4.3396173 -1.7824397

[CoP]^+5^ M = 3

C 1.1880833 2.7923235 -0.0691717

C 0.8321693 4.1991055 -0.0402587

C -0.5139557 4.2567875 0.0739923

C -0.9936967 2.8850545 0.0774573

N 0.0792803 2.0038915 -0.0034867

C 2.5238493 2.3262075 -0.1468137

C -2.3253707 2.5262155 0.1397413

C -2.7841127 1.1883355 0.0705303

C -4.1963127 0.8356835 0.0240273

C -4.2550607 -0.5070575 -0.0909707

C -2.8786557 -0.9861525 -0.1080377

N -1.9962867 0.0837695 -0.0100787

C -2.5272627 -2.3196615 -0.1557447

C -1.1914887 -2.7857665 -0.0761117

C -0.8356217 -4.1926385 -0.0492457

C 0.5102473 -4.2505915 0.0676413

C 0.9899363 -2.8788975 0.0749263

N -0.0828577 -1.9975365 -0.0065957

C 2.3214413 -2.5202205 0.1410183

C 2.7802333 -1.1822335 0.0756733

C 4.1924793 -0.8294935 0.0334873

C 4.2514493 0.5133875 -0.0795937

C 2.8750253 0.9924985 -0.0996047

N 1.9925373 -0.0775395 -0.0052317

Co -0.0018427 0.0031595 -0.0062667

H 1.5245303 5.0255655 -0.0948307

H -1.1339417 5.1381635 0.1423443

H -5.0188707 1.5336215 0.0659883

H -5.1347597 -1.1306465 -0.1460427

H -1.5276527 -5.0191465 -0.1071367

H 1.1300343 -5.1321555 0.1353573

H 5.0149783 -1.5274225 0.0768223

H 5.1314603 1.1368175 -0.1312887

H 5.5341283 -5.0181385 2.4521313

H 3.8467153 -3.1867495 2.3285833

C 4.9769583 -4.8141815 1.5466483

C 4.0396763 -3.8026095 1.4584433

H 7.2348433 -6.2087815 0.7784853

H 6.0010213 -7.3007895 1.4805203

C 6.2613963 -6.6768355 0.6250373

N 5.2384303 -5.6116335 0.4836783

C 3.3463873 -3.6005465 0.2560123

H 6.2755523 -7.2787945 -0.2812137

C 4.5899533 -5.4344375 -0.6880637

C 3.6402153 -4.4359655 -0.8278247

H 4.8525613 -6.1020325 -1.4979877

H 3.1409463 -4.3152885 -1.7814117

H 5.9078033 4.5952775 -2.4466837

H 4.0841123 2.8970545 -2.3312487

C 5.3328703 4.4338245 -1.5438247

C 4.3211613 3.4955845 -1.4602037

H 7.6992903 5.6387125 -0.6931877

H 6.5898173 6.7867725 -1.5045187

C 6.7582463 6.1851295 -0.6111307

N 5.6507133 5.2067545 -0.4791287

C 3.6147183 3.3401195 -0.2592927

H 6.7724903 6.8261095 0.2680843

C 4.9865333 5.0769005 0.6909343

C 3.9647303 4.1524855 0.8261393

H 5.2980843 5.7199945 1.5033633

H 3.4562033 4.0686355 1.7789363

H -5.2862757 -5.7252625 1.4969663

H -3.4544937 -4.0585485 1.7734573

C -4.9759587 -5.0784745 0.6862963

C -3.9628637 -4.1480505 0.8210743

H -6.3865517 -7.1561445 -0.1539287

H -7.5941517 -5.8405385 -0.0212507

C -6.7334137 -6.2140175 -0.5784557

N -5.6388347 -5.2169655 -0.4862707

C -3.6164997 -3.3352875 -0.2679557

H -6.9971647 -6.3551945 -1.6246677

C -5.3282857 -4.4435955 -1.5500707

C -4.3215187 -3.4964715 -1.4664447

H -5.8985657 -4.6061885 -2.4551537

H -4.0885667 -2.8985805 -2.3390357

H -5.5452997 5.0159645 2.4493493

H -3.8557797 3.1865715 2.3252803

C -4.9856157 4.8149715 1.5447453

C -4.0471357 3.8045385 1.4562783

H -7.2445397 6.2073715 0.7669523

H -6.0175797 7.2962305 1.4856873

C -6.2705987 6.6777695 0.6240663

N -5.2452027 5.6148655 0.4831453

C -3.3507767 3.6060675 0.2550243

H -6.2786917 7.2859575 -0.2780937

C -4.5934527 5.4414365 -0.6873557

C -3.6424117 4.4442275 -0.8272897

H -4.8547167 6.1108405 -1.4962207

H -3.1406017 4.3265055 -1.7799047

[CoP]^+5^ M = 5

C -1.1859737 -2.8185510 -0.0676177

C -0.8318977 -4.2055860 0.0346563

C 0.5213803 -4.2518400 0.1749133

C 0.9942523 -2.8838020 0.1084013

N -0.0471977 -2.0162270 -0.0195077

C -2.5019177 -2.3453140 -0.1824837

C 2.3697743 -2.5130250 0.1624853

C 2.8117453 -1.1711780 0.0447793

C 4.2176603 -0.7995510 -0.0923137

C 4.2531163 0.5465390 -0.2209747

C 2.8817483 1.0030460 -0.1660957

N 2.0119433 -0.0773400 -0.0155387

C 2.5038163 2.3421600 -0.1886937

C 1.1879753 2.8155220 -0.0720107

C 0.8346183 4.2026150 0.0313193

C -0.5183687 4.2492160 0.1742013

C -0.9920187 2.8813730 0.1074373

N 0.0490953 2.0135460 -0.0219367

C -2.3675877 2.5107370 0.1633933

C -2.8095927 1.1686860 0.0478183

C -4.2156217 0.7967370 -0.0867157

C -4.2513027 -0.5495330 -0.2130547

C -2.8797757 -1.0059050 -0.1604117

N -2.0098487 0.0747120 -0.0128127

Co 0.0009963 -0.0013140 -0.0018107

H -1.5188887 -5.0383800 0.0196353

H 1.1339103 -5.1285760 0.3167233

H 5.0508023 -1.4850690 -0.1110727

H 5.1220123 1.1764080 -0.3401597

H 1.5218773 5.0352170 0.0157233

H -1.1302057 5.1260790 0.3179983

H -5.0489307 1.4820400 -0.1051107

H -5.1205697 -1.1794410 -0.3291677

H -5.9560777 4.6102390 2.2952943

H -4.2519777 2.8052950 2.1384243

C -5.2464427 4.5652040 1.4789333

C -4.2950957 3.5711080 1.3735003

H -7.3577307 6.1080770 0.6203783

H -6.2702837 7.0594660 1.6766893

C -6.3799467 6.5855400 0.7004013

N -5.3300537 5.5501960 0.5514243

C -3.3889037 3.5729330 0.2964823

H -6.2633767 7.3280500 -0.0866937

C -4.4772727 5.5773880 -0.4978627

C -3.5024657 4.6082720 -0.6459237

H -4.6043547 6.3816850 -1.2101087

H -2.8516607 4.6545800 -1.5104267

H -5.6953987 -4.8086150 -2.5540257

H -3.8681427 -3.1137740 -2.4408597

C -5.1980237 -4.5661980 -1.6236887

C -4.1853337 -3.6280560 -1.5418917

H -7.6503767 -5.6563350 -0.8597457

H -6.5028167 -6.8861250 -1.4749257

C -6.7297627 -6.2049860 -0.6546237

N -5.6183647 -5.2302390 -0.5215997

C -3.5904177 -3.3632420 -0.3017277

H -6.8256397 -6.7636490 0.2741723

C -5.0574007 -4.9933100 0.6846303

C -4.0400267 -4.0636450 0.8223823

H -5.4427957 -5.5580350 1.5230343

H -3.6136127 -3.8909490 1.8029273

H 5.4522233 5.5521670 1.5143583

H 3.6255993 3.8773110 1.7994643

C 5.0586573 4.9885350 0.6782873

C 4.0464363 4.0570920 0.8177923

H 6.4239223 7.1282340 -0.0994027

H 7.6088053 5.7870820 -0.1643567

C 6.7188533 6.2197380 -0.6244697

N 5.6128693 5.2344770 -0.5320017

C 3.5913043 3.3611360 -0.3095177

H 6.9092413 6.4438020 -1.6720257

C 5.1895223 4.5761270 -1.6333667

C 4.1777633 3.6335190 -1.5495027

H 5.6767903 4.8205210 -2.5680267

H 3.8575733 3.1232260 -2.4496747

H 5.9635313 -4.6078570 2.2900223

H 4.2595143 -2.8027260 2.1329423

C 5.2515823 -4.5648410 1.4755663

C 4.3002443 -3.5707090 1.3700673

H 7.3600033 -6.1107360 0.6104223

H 6.2785763 -7.0558190 1.6784763

C 6.3825353 -6.5873760 0.6989483

N 5.3321883 -5.5524110 0.5505563

C 3.3911753 -3.5751930 0.2955353

H 6.2608973 -7.3342050 -0.0832827

C 4.4763873 -5.5822690 -0.4961937

C 3.5015123 -4.6131930 -0.6442217

H 4.6011153 -6.3886620 -1.2064757

H 2.8479013 -4.6618480 -1.5064837

[CoP]^+4^ M = 2

C -1.0982092 -2.8449596 0.0041476

C -0.6815342 -4.2233306 -0.0045414

C 0.6776848 -4.2248406 0.0032176

C 1.0974308 -2.8473776 -0.0066644

N 0.0005638 -2.0084606 -0.0016394

C -2.4331392 -2.4309556 -0.0117514

C 2.4333508 -2.4363346 0.0089876

C 2.8433118 -1.1014866 0.0147286

C 4.2273268 -0.6852776 0.0006886

C 4.2289168 0.6700714 -0.0100664

C 2.8458788 1.0894184 -0.0223444

N 2.0068498 -0.0050676 -0.0032504

C 2.4388558 2.4250834 -0.0163054

C 1.1037728 2.8389774 0.0003726

C 0.6871198 4.2173484 -0.0098634

C -0.6720962 4.2189514 -0.0006814

C -1.0918582 2.8415584 -0.0083194

N 0.0049978 2.0025744 -0.0032064

C -2.4277422 2.4306064 0.0090936

C -2.8376262 1.0957724 0.0165186

C -4.2215752 0.6795494 0.0044516

C -4.2230952 -0.6758166 -0.0050234

C -2.8400602 -1.0951446 -0.0185624

N -2.0011112 -0.0006336 -0.0014294

Co 0.0028308 -0.0029036 -0.0023564

H -1.3354992 -5.0823426 -0.0185994

H 1.3297018 -5.0853116 0.0180396

H 5.0848438 -1.3412706 -0.0059884

H 5.0877778 1.3242884 -0.0048784

H 1.3408088 5.0765404 -0.0261334

H -1.3240402 5.0794824 0.0135846

H -5.0791392 1.3354784 -0.0017714

H -5.0821472 -1.3297766 0.0021236

H -5.9672962 4.8088044 1.9449846

H -4.2236042 3.0291684 2.0062086

C -5.2970702 4.6358694 1.1125086

C -4.3253352 3.6550774 1.1280756

H -7.4746452 5.9926694 0.2203186

H -6.3027892 7.1700224 0.8871356

C -6.5109702 6.4803014 0.0679756

N -5.4518772 5.4435414 0.0359316

C -3.4813832 3.4840704 0.0184666

H -6.5137082 7.0176214 -0.8783964

C -4.6536752 5.3049824 -1.0463154

C -3.6656942 4.3369794 -1.0788094

H -4.8329592 5.9781894 -1.8740924

H -3.0486882 4.2471674 -1.9645004

H -5.9822952 -4.7983786 -1.9432134

H -4.2331052 -3.0242456 -2.0066904

C -5.3102492 -4.6277716 -1.1117244

C -4.3354152 -3.6500746 -1.1285654

H -7.4905702 -5.9783986 -0.2170434

H -6.3229592 -7.1591856 -0.8852464

C -6.5281362 -6.4688356 -0.0658504

N -5.4659842 -5.4351636 -0.0350904

C -3.4894042 -3.4817946 -0.0201304

H -6.5312962 -7.0061176 0.8805356

C -4.6657972 -5.2992356 1.0460476

C -3.6747232 -4.3343646 1.0772566

H -4.8460102 -5.9719966 1.8739846

H -3.0562502 -4.2465306 1.9621206

H 4.8392338 5.9813474 1.8616826

H 3.0583628 4.2409844 1.9579286

C 4.6577368 5.3028864 1.0377586

C 3.6758298 4.3327484 1.0727456

H 6.0140788 7.4666414 0.1263556

H 7.2008948 6.2913154 0.7700446

C 6.4958088 6.5015664 -0.0355014

N 5.4557078 5.4453434 -0.0477014

C 3.4935048 3.4775144 -0.0263644

H 7.0144258 6.5029304 -0.9922334

C 5.3062328 4.6359374 -1.1201964

C 4.3367018 3.6490474 -1.1331804

H 5.9721098 4.8053784 -1.9559644

H 4.2381648 3.0223924 -2.0111334

H 5.9745268 -4.8128846 1.9439226

H 4.2309288 -3.0331536 2.0050616

C 5.3036178 -4.6406496 1.1118486

C 4.3319118 -3.6598316 1.1273916

H 7.4808178 -5.9975826 0.2170206

H 6.3105858 -7.1737806 0.8887076

C 6.5169028 -6.4856976 0.0677106

N 5.4575548 -5.4491956 0.0358036

C 3.4869998 -3.4897866 0.0183706

H 6.5177298 -7.0249696 -0.8775514

C 4.6584318 -5.3115596 -1.0458814

C 3.6703628 -4.3436436 -1.0783254

H 4.8370398 -5.9854466 -1.8732504

H 3.0525398 -4.2546346 -1.9635274

[CoP]^+4^ M = 4

C 1.56001 -2.57936 0.02788

C 1.45150 -3.94605 -0.38690

C 0.13138 -4.17532 -0.64712

C -0.57761 -2.95635 -0.33466

N 0.32948 -1.96575 0.01680

C 2.78371 -1.94663 0.37947

C -1.96589 -2.79812 -0.32311

C -2.57967 -1.54713 0.04880

C -3.95462 -1.43153 0.49413

C -4.17819 -0.11583 0.74574

C -2.95248 0.58044 0.43545

N -1.96135 -0.35012 0.06760

C -2.78604 1.94806 0.37095

C -1.56158 2.57973 0.01942

C -1.45233 3.94534 -0.39878

C -0.13175 4.17391 -0.65728

C 0.57667 2.95593 -0.33984

N -0.33119 1.96609 0.01209

C 1.96488 2.79799 -0.32340

C 2.57740 1.54800 0.05388

C 3.95117 1.43317 0.50302

C 4.17446 0.11769 0.75610

C 2.94939 -0.57893 0.44392

N 1.95897 0.35102 0.07321

Co -0.00097 0.00018 0.01218

H 2.27209 -4.63890 -0.50347

H -0.30670 -5.08513 -1.02800

H -4.64360 -2.25329 0.62089

H -5.08768 0.33847 1.11020

H -2.27288 4.63767 -0.51843

H 0.30712 5.08264 -1.03985

H 4.63959 2.25524 0.63079

H 5.08308 -0.33654 1.12283

H 5.61410 4.73747 -2.48413

H 4.14470 2.80652 -1.98266

C 4.77892 4.82041 -1.80024

C 3.95196 3.76082 -1.50748

H 6.49595 6.90492 -1.17727

H 5.55187 7.26406 -2.65375

C 5.50629 7.14595 -1.56999

N 4.57570 6.04441 -1.24391

C 2.85408 3.92326 -0.63006

H 5.14277 8.06626 -1.11603

C 3.54682 6.23357 -0.38236

C 2.68517 5.20502 -0.06401

H 3.45154 7.22101 0.04972

H 1.90205 5.39456 0.65874

H 4.97356 -5.42199 2.61046

H 2.99202 -3.95966 2.22866

C 4.97149 -4.64741 1.85417

C 3.88420 -3.82649 1.62920

H 7.67444 -5.13956 2.37914

H 6.89419 -6.44035 1.42869

C 7.25077 -5.41032 1.41065

N 6.10517 -4.51386 1.12442

C 3.95015 -2.83071 0.64030

H 7.99946 -5.29246 0.62962

C 6.19600 -3.56977 0.16184

C 5.13600 -2.71913 -0.09876

H 7.12564 -3.52265 -0.38947

H 5.23483 -1.98665 -0.88998

H -7.13666 3.53202 -0.35904

H -5.24959 1.99283 -0.88324

C -6.19947 3.57270 0.18134

C -5.14368 2.72468 -0.09235

H -7.40320 6.04311 0.51897

H -8.14143 4.80198 1.57776

C -7.25559 5.41130 1.39630

N -6.10087 4.51472 1.14892

C -3.94952 2.83419 0.63799

H -7.04611 6.02822 2.26788

C -4.96136 4.64875 1.86464

C -3.87452 3.82739 1.62565

H -4.94962 5.41965 2.62369

H -2.97779 3.95923 2.21854

H -5.60630 -4.74551 -2.49162

H -4.14002 -2.81223 -1.98998

C -4.77376 -4.82601 -1.80423

C -3.94863 -3.76504 -1.51125

H -6.49126 -6.91123 -1.18107

H -5.54268 -7.27126 -2.65444

C -5.50022 -7.15154 -1.57073

N -4.57189 -6.04835 -1.24376

C -2.85394 -3.92441 -0.62931

H -5.13675 -8.07076 -1.11450

C -3.54650 -6.23431 -0.37735

C -2.68673 -5.20432 -0.05861

H -3.45243 -7.22037 0.05815

H -1.90639 -5.39120 0.66782

[CoP]^+3^ M = 1

C -2.6366355 1.1885996 -0.8649728

C -3.2495415 2.4708406 -1.0731598

C -2.4112975 3.4026196 -0.5263708

C -1.2571555 2.6929146 -0.0358718

N -1.3828055 1.3569616 -0.2997538

C -3.2309005 -0.0662794 -1.0729618

C -0.0680385 3.2696766 0.5203822

C 1.1726125 2.6952496 0.1506612

C 2.4149885 3.4161686 -0.0368538

C 3.2978035 2.5476676 -0.5992518

C 2.6294445 1.2724976 -0.7079918

N 1.3220975 1.3891966 -0.2710848

C 3.2387335 0.0635336 -1.0617088

C 2.6446535 -1.1916964 -0.8558898

C 3.2587575 -2.4735804 -1.0624168

C 2.4186755 -3.4059334 -0.5194008

C 1.2622535 -2.6969294 -0.0331948

N 1.3887525 -1.3607354 -0.2953988

C 0.0705265 -3.2740274 0.5172382

C -1.1684565 -2.6992314 0.1427402

C -2.4102795 -3.4200204 -0.0491898

C -3.2914665 -2.5510724 -0.6133338

C -2.6227155 -1.2757904 -0.7192408

N -1.3165335 -1.3929504 -0.2788198

C 0.1238125 -4.4000174 1.4442002

C 4.6218485 0.1180946 -1.5958988

C -0.1262255 4.3950046 1.4477472

C -4.6152475 -0.1184394 -1.6041788

C 4.9100795 0.8509006 -2.7613628

C 6.1979835 0.8930466 -3.2549768

N 7.2045085 0.2362256 -2.6287288

C 6.9629775 -0.4698094 -1.5003948

C 5.6891665 -0.5434134 -0.9683578

C -1.0074295 -4.7936814 2.2137862

C -0.9502875 -5.8380724 3.1053582

N 0.2011595 -6.5216944 3.3239192

C 1.3293015 -6.1265584 2.6821932

C 1.3218755 -5.0880854 1.7822272

C -5.6785355 0.5448066 -0.9716888

C -6.9550285 0.4727486 -1.4971728

N -7.2033315 -0.2343914 -2.6232348

C -6.2005755 -0.8916394 -3.2554478

C -4.9099845 -0.8505534 -2.7685698

C -1.3273185 5.0789216 1.7836992

C -1.3400515 6.1171806 2.6838262

N -0.2144765 6.5160006 3.3278152

C 0.9396325 5.8361576 3.1114932

C 1.0020795 4.7921406 2.2198482

C 0.2279285 -7.6851054 4.2310402

C 8.5736255 0.3211626 -3.1884728

C -0.2469315 7.6792126 4.2349782

C -8.5700455 -0.2945674 -3.1918078

Co 0.0026435 -0.0019194 -0.2174838

H -4.1849035 2.6571326 -1.5808598

H -2.5309095 4.4748456 -0.5665368

H 2.5636875 4.4730476 0.1270452

H 4.3118855 2.7602146 -0.9065008

H 4.1961865 -2.6593094 -1.5664888

H 2.5389635 -4.4780474 -0.5599888

H -2.5593805 -4.4770184 0.1136542

H -4.3046525 -2.7634434 -0.9236308

H 4.1262945 1.3783986 -3.2910708

H 6.4629445 1.4361976 -4.1533728

H 7.8120085 -0.9589414 -1.0412318

H 5.5351365 -1.1068724 -0.0567198

H -1.9425075 -4.2574354 2.1520132

H -1.8130655 -6.1488764 3.6806892

H 2.2350915 -6.6681454 2.9235032

H 2.2747515 -4.8041244 1.3663462

H -5.5194705 1.1069166 -0.0600998

H -7.8007915 0.9636736 -1.0338698

H -6.4714175 -1.4335874 -4.1528888

H -4.1296885 -1.3800744 -3.3014318

H -2.2784325 4.7915656 1.3660312

H -2.2481745 6.6555576 2.9235712

H 1.8002195 6.1497556 3.6885742

H 1.9390595 4.2590486 2.1599902

H 0.0736985 -8.5983024 3.6513802

H 1.1940125 -7.7233354 4.7345492

H -0.5638785 -7.5783554 4.9719962

H 8.9333675 1.3464546 -3.0879768

H 9.2252005 -0.3574204 -2.6411508

H 8.5429685 0.0340056 -4.2401778

H -1.2137765 7.7136006 4.7373042

H 0.5443975 7.5755466 4.9768832

H -0.0956125 8.5930596 3.6555702

H -9.2591635 0.2117096 -2.5186118

H -8.5694135 0.2009806 -4.1639108

H -8.8612625 -1.3397604 -3.3010888

[CoP]^+3^ M = 3

C 2.6363429 -1.2144174 -0.7637068

C 3.2496809 -2.5042734 -0.9029398

C 2.4035679 -3.4125904 -0.3229728

C 1.2427259 -2.6863144 0.1219522

N 1.3841529 -1.3672754 -0.1821578

C 3.2075669 0.0328366 -1.0598708

C 0.0320979 -3.2537624 0.6598732

C -1.2047981 -2.7014494 0.1748572

C -2.3983181 -3.4332494 -0.1658568

C -3.2637521 -2.5451124 -0.7474738

C -2.6183181 -1.2631354 -0.7349878

N -1.3502661 -1.3970894 -0.1910038

C -3.2128771 -0.0306664 -1.0527968

C -2.6409251 1.2169216 -0.7589408

C -3.2536361 2.5070926 -0.8987398

C -2.4065291 3.4154596 -0.3203118

C -1.2452871 2.6890886 0.1232712

N -1.3877971 1.3698316 -0.1793208

C -0.0331951 3.2568316 0.6575422

C 1.2022479 2.7044706 0.1689292

C 2.3946399 3.4362376 -0.1759868

C 3.2586379 2.5476586 -0.7590698

C 2.6134659 1.2655966 -0.7432858

N 1.3467319 1.3998946 -0.1962088

C -0.0619241 4.3441126 1.5953772

C -4.5631851 -0.0636704 -1.6685158

C 0.0636239 -4.3409894 1.5977672

C 4.5592519 0.0645046 -1.6729158

C -4.7702851 -0.7214294 -2.8937768

C -6.0255191 -0.7422514 -3.4669528

N -7.0759111 -0.1361074 -2.8623248

C -6.9127441 0.4942936 -1.6767248

C -5.6746721 0.5430756 -1.0631898

C 1.1178999 4.8796436 2.2036682

C 1.0641239 5.9208246 3.0920562

N -0.1187331 6.4793316 3.4729982

C -1.2763891 5.9410096 2.9980492

C -1.2769361 4.8988406 2.1091512

C 5.6694689 -0.5345604 -1.0583688

C 6.9108459 -0.4838424 -1.6653818

N 7.0774129 0.1405086 -2.8533148

C 6.0270909 0.7357596 -3.4693288

C 4.7691549 0.7138786 -2.9023688

C 1.2801799 -4.8955104 2.1081532

C 1.2823939 -5.9383554 2.9962322

N 0.1262009 -6.4775014 3.4738072

C -1.0578071 -5.9188264 3.0967662

C -1.1143091 -4.8769634 2.2093162

C -0.1431861 7.6556486 4.3572812

C -8.4102481 -0.2007044 -3.5032988

C 0.1536419 -7.6549034 4.3565562

C 8.4071669 0.1825756 -3.5053368

Co -0.0016461 0.0013466 -0.1090358

H 4.1941949 -2.7134294 -1.3838838

H 2.5288139 -4.4849944 -0.2872458

H -2.5399781 -4.4985154 -0.0583368

H -4.2523391 -2.7560544 -1.1297988

H -4.1983311 2.7166746 -1.3790708

H -2.5313221 4.4879446 -0.2850568

H 2.5362909 4.5016486 -0.0697988

H 4.2460819 2.7582276 -1.1445878

H -3.9490311 -1.2066954 -3.4065868

H -6.2282541 -1.2284584 -4.4126528

H -7.7941811 0.9445476 -1.2394068

H -5.5837471 1.0449346 -0.1081388

H 2.0910049 4.4549316 2.0060812

H 1.9567509 6.3379436 3.5414782

H -2.1949951 6.3752236 3.3724302

H -2.2384051 4.4922756 1.8326592

H 5.5755919 -1.0296154 -0.1000508

H 7.7918619 -0.9273594 -1.2207308

H 6.2332269 1.2126866 -4.4191668

H 3.9484919 1.1931336 -3.4217678

H 2.2407759 -4.4883584 1.8295482

H 2.2021609 -6.3725504 3.3677722

H -1.9490751 -6.3364294 3.5484172

H -2.0880501 -4.4522404 2.0149462

H -0.0882861 8.5684316 3.7580962

H -1.0674421 7.6511396 4.9356602

H 0.7073499 7.6115936 5.0382112

H -8.7970621 -1.2171044 -3.4116958

H -9.0784901 0.4990056 -3.0047798

H -8.3101181 0.0712856 -4.5543838

H 1.0779519 -7.6489024 4.9348702

H -0.6969481 -7.6138344 5.0375812

H 0.1009239 -8.5670194 3.7561602

H 8.6887719 1.2240746 -3.6656458

H 9.1363709 -0.3024874 -2.8592658

H 8.3483239 -0.3439764 -4.4590018

[CoP]^+3^ M = 5

C 2.5699832 -1.0534502 -0.9604641

C 3.1732512 -2.3344802 -1.2413031

C 2.4102212 -3.2776172 -0.6125771

C 1.3137492 -2.5797212 0.0230369

N 1.3893232 -1.2647552 -0.2411711

C 3.0872112 0.2140968 -1.2019751

C 0.2062942 -3.1722542 0.7720099

C -1.0981778 -2.7159842 0.4258119

C -2.3258058 -3.4566362 0.4031769

C -3.2784698 -2.6393242 -0.1610491

C -2.6374928 -1.3972502 -0.4731841

N -1.3253468 -1.4602282 -0.1193201

C -3.2996958 -0.2062482 -0.9946351

C -2.7653148 1.0788548 -0.7403861

C -3.3047368 2.3633248 -1.0802321

C -2.4696498 3.3162918 -0.5314531

C -1.4097028 2.6140538 0.1067269

N -1.5629918 1.2744668 -0.0559341

C -0.2213468 3.2144718 0.7368699

C 1.0342172 2.7522038 0.2669829

C 2.2429332 3.5117348 0.0573869

C 3.1198432 2.6898238 -0.5991861

C 2.4697882 1.4146318 -0.7428281

N 1.2020862 1.4821848 -0.2322181

C -0.3448278 4.2551808 1.7191809

C -4.5424508 -0.3932692 -1.7083981

C 0.4424092 -4.1707772 1.7745499

C 4.3928872 0.3423488 -1.8934541

C -4.7163698 -1.4856232 -2.6099011

C -5.8767708 -1.6496572 -3.3176261

N -6.9316948 -0.7908652 -3.1680471

C -6.8259908 0.2406768 -2.2826341

C -5.6752578 0.4604858 -1.5693091

C 0.7660012 4.7200728 2.4853289

C 0.6250822 5.7175728 3.4160649

N -0.5851268 6.2848708 3.6742089

C -1.6866158 5.8226978 3.0214679

C -1.5997808 4.8314618 2.0770889

C 5.5437372 -0.3201822 -1.4399971

C 6.7388152 -0.1796752 -2.1216761

N 6.8204982 0.5964948 -3.2257701

C 5.7302312 1.2610998 -3.6807681

C 4.5155852 1.1550198 -3.0344661

C 1.7339372 -4.7167412 2.0551249

C 1.9153732 -5.6728312 3.0194529

N 0.8817662 -6.1286102 3.7823679

C -0.3520958 -5.5777992 3.6111339

C -0.5871828 -4.6203962 2.6597569

C -0.7000768 7.4068248 4.6232889

C -8.1798798 -1.0343702 -3.9082581

C 1.0999522 -7.2132342 4.7539919

C 8.0956862 0.7154968 -3.9712171

Co -0.0733148 0.0123338 -0.1017781

H 4.0590952 -2.5045862 -1.8358371

H 2.5496782 -4.3488252 -0.6291771

H -2.4484428 -4.4903252 0.6916879

H -4.3173988 -2.8761122 -0.3393071

H -4.1868308 2.5548738 -1.6713231

H -2.5639068 4.3904998 -0.6086711

H 2.3805132 4.5581808 0.2876009

H 4.1166042 2.9358808 -0.9363401

H -3.9081778 -2.1854372 -2.7837021

H -6.0118048 -2.4489712 -4.0354751

H -7.7044568 0.8623228 -2.1634941

H -5.6741908 1.2665468 -0.8481291

H 1.7428682 4.2689178 2.3844139

H 1.4613142 6.0844418 3.9972889

H -2.6301418 6.2806048 3.2905029

H -2.5189478 4.4962728 1.6181939

H 5.5204062 -0.9314382 -0.5467381

H 7.6482992 -0.6719362 -1.8034041

H 5.8686172 1.8634988 -4.5694551

H 3.6627132 1.6951598 -3.4266741

H 2.6148022 -4.3812802 1.5275779

H 2.8868152 -6.1053192 3.2236769

H -1.1298708 -5.9267622 4.2783889

H -1.5748528 -4.1835352 2.6306809

H -0.6384198 8.3519968 4.0779749

H -1.6564658 7.3411508 5.1425129

H 0.1109082 7.3463328 5.3488259

H -8.7288338 -1.8601272 -3.4476601

H -8.7910138 -0.1325162 -3.8893871

H -7.9414928 -1.2843902 -4.9437061

H 2.0625682 -7.0679112 5.2463319

H 0.3060602 -7.1902992 5.5003469

H 1.0908312 -8.1764042 4.2371329

H 8.3071582 1.7712768 -4.1420071

H 8.8956642 0.2699218 -3.3830331

H 7.9956322 0.1915018 -4.9234061

[CoP]^+2^ M = 2

C 2.6359250 -1.2160684 -0.8096988

C 3.2284150 -2.5071364 -0.9548338

C 2.3711780 -3.4113264 -0.3667878

C 1.2360310 -2.6769274 0.0986722

N 1.3881160 -1.3574134 -0.1987528

C 3.2058220 0.0303266 -1.1142418

C 0.0269390 -3.2232174 0.6917782

C -1.2076710 -2.6900264 0.1506392

C -2.3865940 -3.4244584 -0.2006418

C -3.2560690 -2.5389704 -0.7948498

C -2.6181220 -1.2597844 -0.7906278

N -1.3516630 -1.3878544 -0.2222588

C -3.2080010 -0.0275494 -1.1109948

C -2.6377330 1.2192036 -0.8083478

C -3.2298060 2.5103756 -0.9546888

C -2.3720890 3.4149026 -0.3678778

C -1.2368740 2.6806736 0.0976352

N -1.3895270 1.3608966 -0.1982698

C -0.0270730 3.2273536 0.6889122

C 1.2067990 2.6938646 0.1464342

C 2.3852790 3.4280666 -0.2069318

C 3.2540680 2.5420956 -0.8014138

C 2.6161420 1.2629026 -0.7952448

N 1.3502790 1.3914366 -0.2256468

C -0.0578520 4.2463986 1.6591222

C -4.5509700 -0.0587544 -1.7375528

C 0.0589880 -4.2418414 1.6623982

C 4.5496190 0.0603276 -1.7392228

C -4.7556640 -0.7369454 -2.9529558

C -6.0062530 -0.7592044 -3.5351088

N -7.0575270 -0.1354654 -2.9492628

C -6.8975680 0.5158546 -1.7741098

C -5.6638270 0.5676276 -1.1531498

C 1.1257010 4.7817206 2.2924272

C 1.0651770 5.7855896 3.2121692

N -0.1240270 6.3307936 3.6226672

C -1.2828610 5.7936206 3.1243692

C -1.2814120 4.7888536 2.2036552

C 5.6617670 -0.5620874 -1.1494108

C 6.8976660 -0.5083084 -1.7659128

N 7.0601840 0.1418396 -2.9412248

C 6.0088340 0.7587636 -3.5342048

C 4.7562140 0.7346386 -2.9564948

C 1.2832600 -4.7840234 2.2056362

C 1.2859520 -5.7888454 3.1262752

N 0.1277890 -6.3262794 3.6258522

C -1.0619590 -5.7810594 3.2169472

C -1.1237100 -4.7771244 2.2973542

C -0.1523230 7.4921806 4.5155752

C -8.3871650 -0.2043834 -3.5981198

C 0.1573640 -7.4879244 4.5183742

C 8.3812410 0.1733096 -3.6100388

Co -0.0006300 0.0017696 -0.1430118

H 4.1685060 -2.7270594 -1.4408208

H 2.4891390 -4.4844344 -0.3122188

H -2.5280060 -4.4875154 -0.0679298

H -4.2446910 -2.7546594 -1.1760058

H -4.1698560 2.7302986 -1.4407198

H -2.4897290 4.4881006 -0.3142708

H 2.5267770 4.4912436 -0.0752458

H 4.2422200 2.7574006 -1.1840238

H -3.9339250 -1.2378264 -3.4495088

H -6.2060000 -1.2607424 -4.4734688

H -7.7792580 0.9793216 -1.3512288

H -5.5755320 1.0856026 -0.2066478

H 2.1015210 4.3721186 2.0703302

H 1.9558710 6.1918176 3.6769872

H -2.2030490 6.2080816 3.5189812

H -2.2406880 4.3885806 1.9052002

H 5.5717900 -1.0757244 -0.2007068

H 7.7791620 -0.9686334 -1.3394788

H 6.2107760 1.2554776 -4.4747308

H 3.9351570 1.2335266 -3.4561938

H 2.2421280 -4.3835284 1.9061842

H 2.2066750 -6.2031624 3.5197932

H -1.9520390 -6.1873524 3.6828832

H -2.0998340 -4.3675244 2.0765912

H -0.1067910 8.4223756 3.9397692

H -1.0729140 7.4763866 5.1017772

H 0.6999540 7.4479496 5.1962472

H -8.7749440 -1.2201444 -3.5030808

H -9.0591660 0.4979156 -3.1082458

H -8.2809090 0.0610026 -4.6503848

H 1.0783380 -7.4717814 5.1039792

H -0.6944940 -7.4444164 5.1996102

H 0.1120040 -8.4179654 3.9423032

H 8.6242990 1.2053096 -3.8650328

H 9.1338410 -0.2254254 -2.9321588

H 8.3338870 -0.4370234 -4.5135378

[CoP]^+2^ M = 4

C 2.6851049 -1.2476903 -0.6985634

C 3.2185569 -2.5605423 -0.8826734

C 2.3424329 -3.4463293 -0.2882174

C 1.2402879 -2.6837073 0.1938096

N 1.4412509 -1.3568563 -0.0897784

C 3.2968099 0.0028847 -1.0222184

C 0.0042649 -3.2159433 0.7294936

C -1.2143711 -2.6627423 0.2052636

C -2.4183381 -3.3791823 -0.1289574

C -3.2449461 -2.4986983 -0.7770534

C -2.5685911 -1.2325093 -0.8066594

N -1.3192251 -1.3728563 -0.2230504

C -3.1324401 0.0006837 -1.1814364

C -2.5523961 1.2481897 -0.8873234

C -3.1343141 2.5464427 -1.0651164

C -2.3052911 3.4440377 -0.4380284

C -1.1850231 2.7019807 0.0701926

N -1.3293001 1.3855077 -0.2453114

C -0.0025041 3.2323377 0.7060896

C 1.2616069 2.6779767 0.2898126

C 2.4939629 3.3754237 0.1105366

C 3.3803379 2.4903287 -0.4663284

C 2.6963379 1.2460277 -0.6260884

N 1.4034519 1.3880367 -0.1707424

C -0.0931591 4.2716847 1.6858226

C -4.4625181 -0.0302143 -1.8318734

C 0.0038079 -4.2814393 1.6817306

C 4.5829069 0.0551957 -1.6800914

C -4.6605431 -0.7549313 -3.0224734

C -5.9012631 -0.7815053 -3.6240324

N -6.9525451 -0.1185583 -3.0814994

C -6.7996671 0.5795647 -1.9321854

C -5.5754311 0.6387797 -1.2944774

C 1.0395389 4.7337117 2.4368746

C 0.9361409 5.7309977 3.3670656

N -0.2578721 6.3258147 3.6573496

C -1.3826631 5.8582417 3.0389466

C -1.3335301 4.8589557 2.1066216

C 5.6733149 -0.8217443 -1.3856844

C 6.8722859 -0.7222053 -2.0382104

N 7.0842159 0.2120657 -3.0164884

C 6.0677009 1.0798477 -3.3306504

C 4.8627949 1.0333437 -2.6858864

C 1.2085119 -4.8660633 2.1984276

C 1.1852809 -5.8922433 3.1019846

N 0.0149429 -6.3954703 3.5935726

C -1.1569851 -5.8084043 3.2083976

C -1.1883461 -4.7805613 2.3071016

C -0.3318921 7.4608847 4.5873066

C -8.2725511 -0.1963633 -3.7477044

C 0.0104239 -7.5616313 4.4872286

C 8.3431199 0.2497597 -3.7692944

Co 0.0497429 0.0098717 -0.1200534

H 4.1231279 -2.8192443 -1.4109474

H 2.4203109 -4.5246293 -0.2721034

H -2.5917531 -4.4338943 0.0267716

H -4.2284771 -2.7041183 -1.1754434

H -4.0474691 2.7711067 -1.5973554

H -2.4176541 4.5183097 -0.4064164

H 2.6692429 4.4220047 0.3143646

H 4.4109709 2.6864517 -0.7246384

H -3.8398231 -1.2883863 -3.4858774

H -6.0940141 -1.3172033 -4.5449054

H -7.6806031 1.0723137 -1.5421484

H -5.4959441 1.1922347 -0.3674564

H 2.0092859 4.2748137 2.3124896

H 1.7933399 6.0849037 3.9264786

H -2.3167241 6.3153437 3.3417496

H -2.2772711 4.5083117 1.7157786

H 5.5958049 -1.5423763 -0.5826084

H 7.7156819 -1.3568823 -1.7956274

H 6.2754029 1.7855297 -4.1254414

H 4.0945679 1.7352577 -2.9867894

H 2.1768209 -4.4914973 1.8995166

H 2.0938419 -6.3460463 3.4783006

H -2.0570611 -6.1941353 3.6714516

H -2.1503541 -4.3301703 2.1099236

H -0.2714961 8.4027097 4.0343306

H -1.2748241 7.4186387 5.1346426

H 0.4954169 7.4006767 5.2953676

H -8.6668581 -1.2077823 -3.6346574

H -8.9484641 0.5201367 -3.2843564

H -8.1498941 0.0434337 -4.8043534

H 0.9537919 -7.6005593 5.0330216

H -0.8107761 -7.4684883 5.1994946

H -0.1128831 -8.4793283 3.9048666

H 8.6289789 1.2881907 -3.9478224

H 9.1252299 -0.2393993 -3.1876814

H 8.2270959 -0.2664153 -4.7273974

[CoP]^+2^ M = 6

C 2.6520314 -1.0719427 -0.8483195

C 3.1367764 -2.3610567 -1.2378415

C 2.3272154 -3.3059507 -0.6306775

C 1.3337434 -2.5949947 0.0889595

N 1.5001284 -1.2526527 -0.0746955

C 3.1912904 0.2079073 -1.1179955

C 0.2048874 -3.1600437 0.8460825

C -1.0830106 -2.7052717 0.4498115

C -2.3261036 -3.4141947 0.4332865

C -3.2439656 -2.6009927 -0.1959675

C -2.5642676 -1.3923487 -0.5469495

N -1.2620806 -1.4699077 -0.1607925

C -3.1866216 -0.2057457 -1.1229885

C -2.6470186 1.0740893 -0.8534785

C -3.1299056 2.3632653 -1.2452495

C -2.3215656 3.3081273 -0.6364885

C -1.3303566 2.5970993 0.0861665

N -1.4969286 1.2547533 -0.0771295

C -0.2031186 3.1622513 0.8456285

C 1.0854794 2.7074013 0.4518655

C 2.3286394 3.4161923 0.4374425

C 3.2474254 2.6029983 -0.1905015

C 2.5681394 1.3944903 -0.5427565

N 1.2654334 1.4720273 -0.1585855

C -0.4205526 4.1385153 1.8617285

C -4.3955066 -0.3876967 -1.8898905

C 0.4202284 -4.1359237 1.8630625

C 4.4014724 0.3900643 -1.8829155

C -4.5602456 -1.5151307 -2.7510705

C -5.6902066 -1.6798127 -3.5054615

N -6.7266526 -0.7869537 -3.4449885

C -6.6317826 0.2805643 -2.6004235

C -5.5102366 0.5014973 -1.8439415

C 0.6138974 4.5767603 2.7525995

C 0.3826074 5.5262323 3.7097005

N -0.8507006 6.0875693 3.8824315

C -1.8870076 5.6484653 3.1065315

C -1.7098406 4.7014253 2.1356595

C 5.5177614 -0.4968587 -1.8330995

C 6.6423054 -0.2726327 -2.5845805

N 6.7416214 0.7999263 -3.4216075

C 5.6993894 1.6854333 -3.4930455

C 4.5663564 1.5172253 -2.7442675

C 1.7089304 -4.6986017 2.1400895

C 1.8840634 -5.6451657 3.1118245

N 0.8461534 -6.0839317 3.8857045

C -0.3868136 -5.5226957 3.7100775

C -0.6160726 -4.5737017 2.7520235

C -1.0585256 7.1720513 4.8530895

C -7.9491146 -1.0330267 -4.2246215

C 1.0514874 -7.1682397 4.8570855

C 7.9287614 1.0049703 -4.2650365

Co 0.0016594 0.0010323 -0.1130905

H 3.9654754 -2.5630977 -1.8990595

H 2.4034544 -4.3818607 -0.7098505

H -2.4822546 -4.4291927 0.7689535

H -4.2845656 -2.8187787 -0.3903245

H -3.9564696 2.5654653 -1.9090635

H -2.3970616 4.3840313 -0.7164755

H 2.4843534 4.4311413 0.7734585

H 4.2883594 2.8206953 -0.3831675

H -3.7656846 -2.2439147 -2.8537485

H -5.8154706 -2.5069157 -4.1930695

H -7.4971866 0.9292903 -2.5496495

H -5.5208136 1.3381563 -1.1587555

H 1.5980744 4.1311033 2.7195375

H 1.1588244 5.8632913 4.3851715

H -2.8537596 6.0927063 3.3086325

H -2.5821836 4.3858173 1.5801195

H 5.5285494 -1.3320847 -1.1461375

H 7.5075374 -0.9214387 -2.5331315

H 5.8239874 2.5109163 -4.1826025

H 3.7711944 2.2451063 -2.8486715

H 2.5825044 -4.3831437 1.5864215

H 2.8504334 -6.0891837 3.3162075

H -1.1644276 -5.8593787 4.3841345

H -1.6001496 -4.1280037 2.7167265

H -1.0032736 8.1400243 4.3470345

H -2.0386686 7.0576343 5.3187995

H -0.2876866 7.1182823 5.6222585

H -8.5575806 -1.7964587 -3.7317735

H -8.5184366 -0.1070707 -4.3040435

H -7.6758116 -1.3724087 -5.2253685

H 2.0328314 -7.0570657 5.3209955

H 0.2821774 -7.1107897 5.6275415

H 0.9917434 -8.1365017 4.3520875

H 8.2687664 2.0382113 -4.1692125

H 8.7222284 0.3338603 -3.9372865

H 7.6813034 0.7946963 -5.3090845

[CoP]^+2^ M = 8

C 2.9086752 -0.6978302 -0.4808338

C 3.7380572 -1.8694372 -0.4828798

C 2.9500972 -2.9290952 -0.0913238

C 1.6511902 -2.4065022 0.1817232

N 1.6328042 -1.0613992 -0.0699478

C 3.3416742 0.6313518 -0.7412568

C 0.5110262 -3.1903352 0.6401442

C -0.8295988 -2.7530262 0.4890552

C -2.0149348 -3.3945672 0.9826592

C -3.0887798 -2.6332152 0.5723012

C -2.5580748 -1.5189062 -0.1398818

N -1.1962328 -1.5768392 -0.1631028

C -3.3521088 -0.4457892 -0.7379228

C -2.8708338 0.8855048 -0.7648158

C -3.4333318 2.0191018 -1.4352928

C -2.6623088 3.1166068 -1.0979748

C -1.6301198 2.6398818 -0.2436788

N -1.7225178 1.2951718 -0.0779678

C -0.5523578 3.4562088 0.3488412

C 0.7959942 3.0908398 0.0430492

C 1.8727672 3.9675478 -0.2733878

C 2.9360262 3.1743408 -0.6579928

C 2.5235722 1.8131348 -0.5066948

N 1.2061042 1.7771388 -0.1273758

C -0.8708178 4.6097648 1.1240392

C -4.6567458 -0.7894752 -1.2409328

C 0.7872242 -4.4792132 1.2409932

C 4.6981162 0.8125788 -1.2359928

C -4.9242008 -2.0671892 -1.8261788

C -6.1586498 -2.3835402 -2.3227588

N -7.2064158 -1.5019102 -2.2560168

C -7.0085698 -0.2850372 -1.6643518

C -5.7849078 0.0869058 -1.1764558

C 0.1186112 5.3546888 1.8481512

C -0.2103448 6.4628208 2.5794772

N -1.5006338 6.9017558 2.6785422

C -2.4887628 6.1854518 2.0631542

C -2.2143198 5.0714118 1.3183572

C 5.6120912 1.7517198 -0.6781288

C 6.8890292 1.8762548 -1.1606348

N 7.3303012 1.1268798 -2.2155448

C 6.4873812 0.2107368 -2.7794148

C 5.2098502 0.0343858 -2.3130108

C 0.0156332 -5.6482552 0.9669792

C 0.3067622 -6.8513292 1.5546042

N 1.3406282 -6.9838742 2.4392162

C 2.1109562 -5.8877422 2.7294472

C 1.8736602 -4.6724122 2.1461752

C -1.8181478 8.1466948 3.3931612

C -8.5421218 -1.9021112 -2.7188878

C 1.5933002 -8.2603332 3.1217672

C 8.7184252 1.2620058 -2.6827238

Co -0.0172238 0.1140918 -0.0545518

H 4.7914502 -1.9070182 -0.7176698

H 3.2513342 -3.9631352 -0.0038798

H -2.0538478 -4.2857252 1.5913752

H -4.1367548 -2.8151702 0.7676452

H -4.2813888 2.0103118 -2.1043858

H -2.7868878 4.1378908 -1.4323608

H 1.8222242 5.0470358 -0.3060798

H 3.8821462 3.5245238 -1.0408938

H -4.1323418 -2.7997112 -1.9224568

H -6.3658578 -3.3319902 -2.8024138

H -7.8784568 0.3556148 -1.5907618

H -5.7063778 1.0468208 -0.6833678

H 1.1504012 5.0318418 1.8637432

H 0.5303242 7.0319708 3.1272872

H -3.4984828 6.5511558 2.2022902

H -3.0497568 4.5437948 0.8788112

H 5.3310702 2.3503618 0.1788212

H 7.6101342 2.5561398 -0.7243788

H 6.8796282 -0.3465072 -3.6206228

H 4.5744252 -0.6844792 -2.8152948

H -0.7860598 -5.6170832 0.2402862

H -0.2507678 -7.7528152 1.3330472

H 2.9030702 -6.0408562 3.4515662

H 2.5110122 -3.8403462 2.4193062

H -1.8102458 8.9885768 2.6950082

H -2.8049678 8.0583338 3.8494282

H -1.0755198 8.3129618 4.1741412

H -9.0746468 -2.4277422 -1.9206208

H -9.1042548 -1.0138212 -3.0092888

H -8.4415458 -2.5595802 -3.5837548

H 1.1506722 -9.0712592 2.5429782

H 2.6696412 -8.4232492 3.2000912

H 1.1514922 -8.2427442 4.1223812

H 8.7944172 0.8726518 -3.6980418

H 8.9995592 2.3162408 -2.6812408

H 9.3882662 0.7013958 -2.0246178

[CoP]^+1^ M = 1

C 1.1912184 -2.7120369 0.2318098

C 2.3844544 -3.4410139 0.0126558

C 3.3012884 -2.5671249 -0.5584362

C 2.6656464 -1.3064609 -0.6844692

N 1.3645454 -1.4173159 -0.1993682

C -0.0865756 -3.2192469 0.7155018

C 3.2866274 -0.0767339 -1.0702842

C 2.7015954 1.1864341 -0.7548132

C 3.2397694 2.4835341 -0.9763582

C 2.3744474 3.3945051 -0.3866702

C 1.2902944 2.6535501 0.1435958

N 1.4718254 1.3216971 -0.1045202

C 0.0704604 3.2037691 0.7355338

C -1.1633036 2.6944741 0.1839938

C -2.3506646 3.4281841 -0.1286592

C -3.2068706 2.5636761 -0.7755032

C -2.5595416 1.2921421 -0.8301152

N -1.2969096 1.4047581 -0.2528352

C -3.1513206 0.0694341 -1.2027202

C -2.6004036 -1.1894769 -0.9029202

C -3.1985926 -2.4757299 -1.0867032

C -2.3819156 -3.3902009 -0.4583162

C -1.2604136 -2.6685169 0.0610908

N -1.3828356 -1.3486469 -0.2404352

Co 0.0411284 -0.0101869 -0.1231612

H 2.5297444 -4.4940579 0.2100328

H 4.3233884 -2.7930979 -0.8285742

H 4.1433014 2.7208311 -1.5179512

H 2.4655344 4.4726411 -0.3758302

H -2.5064226 4.4823071 0.0515768

H -4.1945726 2.7908501 -1.1524712

H -4.1141066 -2.6854069 -1.6217862

H -2.5135866 -4.4624519 -0.4107942

H -7.7080476 -0.9472449 -1.6395112

H -5.5360316 -1.1257929 -0.4464662

C -6.8157126 -0.4543709 -2.0038792

C -5.6011086 -0.5447249 -1.3573802

H -8.7645526 -0.5910219 -3.7570722

H -8.8839516 1.1157911 -3.2356662

C -8.2806806 0.3855621 -3.7792092

N -6.9528526 0.2809691 -3.1354842

C -4.4725246 0.1322791 -1.8605232

H -8.1510406 0.7053121 -4.8121112

C -5.8905636 0.9532691 -3.6431272

C -4.6568756 0.8976941 -3.0282672

H -6.0644976 1.5197921 -4.5486512

H -3.8275226 1.4390911 -3.4661512

H -2.4777186 -6.0872879 3.4922888

H -2.3915556 -4.3175019 1.8280368

C -1.5294126 -5.6933119 3.1451948

C -1.4575256 -4.7177459 2.1986688

H -1.4492526 -7.3051369 5.2062248

H -0.4752966 -8.3106129 4.1027048

C -0.5096966 -7.3636199 4.6526188

N -0.4100946 -6.2253799 3.7389368

C -0.1934556 -4.2003619 1.7137508

H 0.3188034 -7.3327419 5.3631848

C 0.8098824 -5.6988159 3.3902108

C 0.9407734 -4.7289219 2.4441498

H 1.6635134 -6.0934339 3.9291928

H 1.9318974 -4.3328579 2.2711268

H -1.8682216 6.1361031 3.7901378

H -2.0427296 4.3330281 2.1685938

C -0.9863266 5.7360651 3.3032288

C -1.0634236 4.7430141 2.3746358

H 0.2159144 8.3686671 4.0290138

H -0.5919296 7.3946061 5.2841448

C 0.2562994 7.4332361 4.5976768

N 0.2114014 6.2769451 3.7015388

C 0.1099304 4.2100891 1.7144488

H 1.1801384 7.4069841 5.1791098

C 1.3623984 5.7439971 3.1743248

C 1.3442044 4.7507611 2.2432918

H 2.2892264 6.1565541 3.5557408

H 2.2956594 4.3583641 1.9100988

H 7.7308014 1.2186331 -1.8065742

H 5.6016524 1.4247831 -0.6071612

C 6.8777744 0.6030751 -2.0640352

C 5.6782164 0.7091151 -1.4150912

H 8.9420834 0.4816931 -3.6048232

H 8.9449704 -1.2723839 -3.2851712

C 8.3792464 -0.4446769 -3.7252672

N 7.0755344 -0.3096109 -3.0692672

C 4.5753214 -0.1466449 -1.7273592

H 8.2327474 -0.6337589 -4.7910452

C 6.0488144 -1.1673029 -3.3912832

C 4.8439534 -1.1131059 -2.7479682

H 6.2500004 -1.8655789 -4.1945272

H 4.0642124 -1.7966099 -3.0613012

[CoP]^+1^ M = 3

C 1.1824425 -2.7144011 0.2360114

C 2.3773625 -3.4448131 0.0309444

C 3.2966975 -2.5752381 -0.5423946

C 2.6617695 -1.3152341 -0.6828246

N 1.3591665 -1.4220991 -0.2052926

C -0.0985775 -3.2173641 0.7135564

C 3.2875775 -0.0887681 -1.0731526

C 2.7048335 1.1770789 -0.7621696

C 3.2424885 2.4724179 -0.9922626

C 2.3794045 3.3870959 -0.4037606

C 1.2985345 2.6494479 0.1361104

N 1.4778655 1.3163819 -0.1063766

C 0.0823735 3.2028239 0.7347744

C -1.1537665 2.6988529 0.1841524

C -2.3401875 3.4364949 -0.1245916

C -3.1986935 2.5759869 -0.7732146

C -2.5540735 1.3028499 -0.8323806

N -1.2910125 1.4109709 -0.2562946

C -3.1489955 0.0827989 -1.2072056

C -2.6017905 -1.1783071 -0.9102396

C -3.2026135 -2.4624901 -1.0990336

C -2.3894295 -3.3807391 -0.4714426

C -1.2682085 -2.6629221 0.0538384

N -1.3864895 -1.3422941 -0.2445346

Co 0.0420885 -0.0099001 -0.1240606

H 2.5211035 -4.4967131 0.2350144

H 4.3199245 -2.8035521 -0.8062546

H 4.1441465 2.7064539 -1.5383696

H 2.4715395 4.4652299 -0.3970886

H -2.4931915 4.4903379 0.0595184

H -4.1860865 2.8064629 -1.1490436

H -4.1176815 -2.6682151 -1.6364286

H -2.5237605 -4.4527951 -0.4270726

H -7.7096085 -0.9178591 -1.6400236

H -5.5365035 -1.1047581 -0.4493856

C -6.8159345 -0.4278491 -2.0049646

C -5.6008775 -0.5227281 -1.3597476

H -8.7647475 -0.5587751 -3.7599006

H -8.8816165 1.1459729 -3.2309986

C -8.2798485 0.4173779 -3.7783256

N -6.9515065 0.3084739 -3.1360006

C -4.4708805 0.1505239 -1.8640816

H -8.1506835 0.7414319 -4.8099186

C -5.8876815 0.9770749 -3.6446816

C -4.6532895 0.9167649 -3.0312776

H -6.0606525 1.5447639 -4.5496486

H -3.8224335 1.4552749 -3.4698686

H -2.5140035 -6.0749001 3.4799844

H -2.4127795 -4.3089821 1.8125444

C -1.5625045 -5.6836071 3.1386394

C -1.4821035 -4.7101491 2.1905084

H -1.4992735 -7.2898551 5.2047814

H -0.5218405 -8.3008691 4.1093694

C -0.5566985 -7.3525221 4.6568514

N -0.4484305 -6.2168361 3.7408284

C -0.2137915 -4.1966971 1.7130114

H 0.2677745 -7.3222051 5.3720774

C 0.7748705 -5.6937391 3.3992864

C 0.9142485 -4.7261321 2.4519914

H 1.6240275 -6.0889091 3.9448584

H 1.9073235 -4.3323501 2.2854104

H -1.8356865 6.1345609 3.8025784

H -2.0225355 4.3356709 2.1777194

C -0.9570635 5.7328239 3.3111564

C -1.0410985 4.7421759 2.3808194

H 0.2595505 8.3601929 4.0379134

H -0.5507435 7.3874379 5.2923844

C 0.2963875 7.4232259 4.6043794

N 0.2441335 6.2689819 3.7062344

C 0.1282115 4.2065439 1.7150384

H 1.2211225 7.3913509 5.1842004

C 1.3915005 5.7340589 3.1724094

C 1.3665135 4.7433099 2.2391374

H 2.3210825 6.1434579 3.5505684

H 2.3152495 4.3497599 1.8993434

H 7.7322915 1.1990969 -1.8190706

H 5.6026325 1.4176779 -0.6237946

C 6.8792935 0.5812149 -2.0710916

C 5.6795055 0.6938229 -1.4243826

H 8.9427875 0.4463799 -3.6136126

H 8.9502785 -1.3028441 -3.2687026

C 8.3822285 -0.4830711 -3.7207346

N 7.0780575 -0.3415641 -3.0677146

C 4.5761685 -0.1653691 -1.7280286

H 8.2370215 -0.6878761 -4.7838106

C 6.0514125 -1.2032021 -3.3807426

C 4.8463945 -1.1426451 -2.7388236

H 6.2529805 -1.9097951 -4.1766336

H 4.0670055 -1.8291521 -3.0464946

[CoP]^+1^ M = 5

C 1.2122184 -2.6631954 0.3435529

C 2.4397984 -3.3597654 0.1658539

C 3.3113584 -2.4952724 -0.4719121

C 2.6247984 -1.2629734 -0.6647961

N 1.3397414 -1.3901674 -0.1728991

C -0.0453996 -3.1934924 0.8080279

C 3.2116724 -0.0359494 -1.1265531

C 2.6172004 1.2231546 -0.8028881

C 3.1370424 2.5327826 -1.0378771

C 2.3010664 3.4264336 -0.3949571

C 1.2362844 2.6694756 0.1679829

N 1.4148584 1.3411836 -0.1157421

C 0.0454094 3.1933966 0.8081449

C -1.2122046 2.6631266 0.3435849

C -2.4397026 3.3597716 0.1656699

C -3.3112256 2.4952996 -0.4721841

C -2.6247466 1.2629186 -0.6648421

N -1.3397506 1.3900616 -0.1727311

C -3.2116236 0.0359116 -1.1266121

C -2.6171706 -1.2232124 -0.8030011

C -3.1370166 -2.5328274 -1.0380351

C -2.3010246 -3.4265134 -0.3951821

C -1.2362616 -2.6695734 0.1678269

N -1.4148316 -1.3412824 -0.1158541

Co -0.0000026 -0.0000474 -0.0686341

H 2.6274954 -4.3942924 0.4151309

H 4.3360004 -2.7009384 -0.7472931

H 4.0069234 2.7843016 -1.6249341

H 2.3821034 4.5048816 -0.3860741

H -2.6273676 4.3943236 0.4148639

H -4.3358136 2.7010436 -0.7477081

H -4.0068986 -2.7843164 -1.6251061

H -2.3820526 -4.5049614 -0.3863711

H -7.5790956 -1.3082034 -2.1725131

H -5.5206256 -1.5389484 -0.8686891

C -6.7292316 -0.6597384 -2.3462581

C -5.5640556 -0.7811244 -1.6392301

H -8.7503586 -0.4567754 -3.9101621

H -8.7203676 1.3081366 -3.6636161

C -8.1562696 0.4482406 -4.0377921

N -6.8958566 0.3083296 -3.3008811

C -4.4655406 0.1142466 -1.8380381

H -7.9447706 0.5881056 -5.1005971

C -5.8758506 1.2046866 -3.5139221

C -4.7058126 1.1371886 -2.8112241

H -6.0512436 1.9470636 -4.2829981

H -3.9289536 1.8557086 -3.0430441

H -2.3674386 -6.2358334 3.4674079

H -2.3160266 -4.4379024 1.8235479

C -1.4310516 -5.7871154 3.1582599

C -1.3771726 -4.7936834 2.2239809

H -1.3298926 -7.3758384 5.2335439

H -0.3083656 -8.3477804 4.1409599

C -0.3810766 -7.4064034 4.6947779

N -0.3052256 -6.2659494 3.7763549

C -0.1304256 -4.2138044 1.7950409

H 0.4373104 -7.3464244 5.4140149

C 0.8955154 -5.6778914 3.4779969

C 1.0039824 -4.6862264 2.5462419

H 1.7505114 -6.0355004 4.0389919

H 1.9763544 -4.2326394 2.4183979

H -1.7506776 6.0356726 4.0387649

H -1.9764696 4.2327606 2.4182129

C -0.8956416 5.6779666 3.4778949

C -1.0040756 4.6862706 2.5461699

H 0.3091344 8.3477956 4.1408469

H -0.4378686 7.3469176 5.4134919

C 0.3809994 7.4064156 4.6947689

N 0.3051114 6.2659446 3.7763689

C 0.1303874 4.2137336 1.7951219

H 1.3294644 7.3753816 5.2341359

C 1.4309734 5.7870116 3.1584139

C 1.3771304 4.7935496 2.2241679

H 2.3673554 6.2356786 3.4676539

H 2.3159994 4.4376896 1.8238359

H 7.5790204 1.3083576 -2.1727441

H 5.5206694 1.5389536 -0.8687021

C 6.7291604 0.6598666 -2.3464291

C 5.5640654 0.7811496 -1.6392651

H 8.7484874 0.4587146 -3.9136021

H 8.7223884 -1.3053674 -3.6603851

C 8.1563644 -0.4480914 -4.0376791

N 6.8957094 -0.3080984 -3.3011891

C 4.4655734 -0.1142724 -1.8379851

H 7.9449854 -0.5923784 -5.0998951

C 5.8758214 -1.2046414 -3.5139831

C 4.7058634 -1.1372514 -2.8111341

H 6.0511724 -1.9470754 -4.2830141

H 3.9290554 -1.8558614 -3.0428431

[CoP]^0^ M = 2

C 2.6429157 -1.1543150 -0.8583654

C 3.1668267 -2.4495860 -1.1104044

C 2.3375217 -3.3630510 -0.4651164

C 1.2885457 -2.6261150 0.1272126

N 1.4523817 -1.2937680 -0.1375904

C 3.2106527 0.1187840 -1.1786384

C 0.1160917 -3.1507480 0.8307076

C -1.1579273 -2.6777060 0.3105376

C -2.3507363 -3.4168400 0.1030246

C -3.2446983 -2.5761050 -0.5452914

C -2.5963913 -1.3241200 -0.7251264

N -1.3135853 -1.4112310 -0.1949824

C -3.2045273 -0.1131740 -1.1854564

C -2.6375963 1.1611280 -0.8684044

C -3.1607863 2.4557000 -1.1258084

C -2.3327983 3.3711420 -0.4818014

C -1.2852123 2.6360420 0.1153156

N -1.4485883 1.3028540 -0.1455924

C -0.1144303 3.1630030 0.8197286

C 1.1607537 2.6881540 0.3043356

C 2.3541237 3.4266290 0.0973166

C 3.2494097 2.5840340 -0.5465414

C 2.6015057 1.3314140 -0.7240064

N 1.3175807 1.4201230 -0.1969714

C -0.2314703 4.0994280 1.8456406

C -4.4542833 -0.2131000 -1.9053564

C 0.2307437 -4.0831880 1.8603776

C 4.4632617 0.2154300 -1.8939164

C -4.6748793 -1.2375220 -2.8824874

C -5.8406973 -1.3245910 -3.5890984

N -6.8791063 -0.4442300 -3.3786444

C -6.7312093 0.5239300 -2.4155534

C -5.5699273 0.6633350 -1.7076554

C 0.8939967 4.6055980 2.6153196

C 0.7466087 5.5389080 3.5915516

N -0.4824703 6.0553750 3.9421996

C -1.5936653 5.5519250 3.2983806

C -1.5063913 4.6152440 2.3183986

C 5.5781617 -0.6604120 -1.6868784

C 6.7435677 -0.5228250 -2.3877064

N 6.9035047 0.4541070 -3.3406754

C 5.8600127 1.3221740 -3.5720834

C 4.6892517 1.2363210 -2.8730404

C 1.5046327 -4.5970120 2.3381806

C 1.5897147 -5.5297170 3.3221056

N 0.4770797 -6.0307390 3.9653716

C -0.7512833 -5.5159920 3.6096016

C -0.8965203 -4.5866640 2.6292766

C -0.5930893 7.1774590 4.8698496

C -8.1447983 -0.6156960 -4.0953524

C 0.5856517 -7.1492170 4.8975916

C 8.1143487 0.5137170 -4.1620354

Co 0.0018737 0.0045250 -0.1202474

H 4.0386247 -2.6873680 -1.7017814

H 2.4394407 -4.4404580 -0.4430444

H -2.5042483 -4.4554360 0.3627726

H -4.2605133 -2.8105880 -0.8325754

H -4.0313033 2.6917760 -1.7197004

H -2.4346593 4.4486210 -0.4635084

H 2.5070847 4.4660380 0.3541056

H 4.2657767 2.8178140 -0.8323784

H -3.8842463 -1.9420570 -3.1107394

H -6.0005903 -2.0687490 -4.3598514

H -7.5935303 1.1560390 -2.2417864

H -5.5366013 1.4227520 -0.9378254

H 1.8875117 4.2124320 2.4441256

H 1.5898847 5.9116310 4.1623826

H -2.5466703 5.9422690 3.6379736

H -2.4298663 4.2431870 1.8931156

H 5.5411847 -1.4146420 -0.9120294

H 7.6042967 -1.1563140 -2.2103454

H 6.0224077 2.0616190 -4.3466114

H 3.9012557 1.9421400 -3.1063224

H 2.4290407 -4.2265160 1.9135516

H 2.5419697 -5.9185620 3.6655026

H -1.5958693 -5.8866460 4.1798516

H -1.8897293 -4.1945110 2.4539666

H -0.5363253 8.1377140 4.3430856

H -1.5463903 7.1201370 5.4006516

H 0.2164327 7.1268520 5.6017816

H -8.7407133 -1.4130340 -3.6385934

H -8.7077343 0.3179390 -4.0636924

H -7.9419123 -0.8701950 -5.1382254

H 1.5381657 -7.0902850 5.4296256

H -0.2249453 -7.0953550 5.6281146

H 0.5292267 -8.1115290 4.3745466

H 8.3379077 1.5541880 -4.4058914

H 8.9521967 0.0943610 -3.6024054

H 7.9797897 -0.0540840 -5.0890404

[CoP]^0^ M = 4

C 2.6402450 -1.1612202 -0.8494378

C 3.1700020 -2.4550412 -1.0949148

C 2.3424660 -3.3698662 -0.4508108

C 1.2864640 -2.6351632 0.1334962

N 1.4461680 -1.3025412 -0.1342868

C 3.2069940 0.1112028 -1.1693138

C 0.1124140 -3.1630442 0.8292672

C -1.1610260 -2.6774042 0.3194002

C -2.3602590 -3.4082822 0.1179602

C -3.2498950 -2.5628072 -0.5294818

C -2.5949120 -1.3156722 -0.7132878

N -1.3105300 -1.4098472 -0.1864708

C -3.2004610 -0.1049932 -1.1766468

C -2.6346150 1.1686968 -0.8600348

C -3.1636880 2.4617898 -1.1110538

C -2.3375700 3.3786798 -0.4682168

C -1.2829720 2.6458788 0.1210432

N -1.4421540 1.3123758 -0.1427118

C -0.1106590 3.1760978 0.8178442

C 1.1639860 2.6886668 0.3129502

C 2.3638000 3.4188678 0.1121942

C 3.2548910 2.5714538 -0.5305168

C 2.6003600 1.3236548 -0.7119358

N 1.3147510 1.4194898 -0.1885498

C -0.2250350 4.1209778 1.8377402

C -4.4501700 -0.2059722 -1.8973658

C 0.2242800 -4.1040292 1.8529182

C 4.4597690 0.2087598 -1.8850788

C -4.6680640 -1.2300482 -2.8760478

C -5.8324190 -1.3186402 -3.5843848

N -6.8733760 -0.4397102 -3.3748938

C -6.7287620 0.5267188 -2.4084108

C -5.5688240 0.6672758 -1.6990858

C 0.9012380 4.6251688 2.6069052

C 0.7576020 5.5670658 3.5756962

N -0.4686250 6.0940298 3.9190532

C -1.5814310 5.5903578 3.2789032

C -1.4975950 4.6453898 2.3063462

C 5.5775200 -0.6640042 -1.6771668

C 6.7416940 -0.5256002 -2.3793578

N 6.8989800 0.4500458 -3.3351928

C 5.8526880 1.3161338 -3.5666328

C 4.6832940 1.2290588 -2.8659718

C 1.4957430 -4.6265122 2.3267302

C 1.5772520 -5.5676702 3.3031422

N 0.4629140 -6.0690142 3.9424832

C -0.7625510 -5.5436492 3.5938992

C -0.9038990 -4.6055622 2.6210972

C -0.5748220 7.2233038 4.8386752

C -8.1386330 -0.6142612 -4.0906148

C 0.5669570 -7.1948702 4.8665192

C 8.1067340 0.5081078 -4.1603328

Co 0.0019800 0.0049258 -0.1080708

H 4.0439810 -2.6910632 -1.6836058

H 2.4463830 -4.4469792 -0.4293258

H -2.5194100 -4.4463852 0.3759492

H -4.2671980 -2.7917392 -0.8158178

H -4.0363470 2.6960288 -1.7023608

H -2.4414880 4.4558588 -0.4505908

H 2.5223660 4.4577978 0.3671832

H 4.2728210 2.7996318 -0.8151628

H -3.8759480 -1.9332062 -3.1036628

H -5.9898590 -2.0623132 -4.3561138

H -7.5929990 1.1560578 -2.2339538

H -5.5389010 1.4246408 -0.9270488

H 1.8925290 4.2238468 2.4426452

H 1.6019650 5.9385248 4.1457072

H -2.5330200 5.9870678 3.6149862

H -2.4229320 4.2732698 1.8854792

H 5.5437270 -1.4160312 -0.8999878

H 7.6041400 -1.1565632 -2.2011908

H 6.0128220 2.0548338 -4.3423598

H 3.8939060 1.9335288 -3.0988868

H 2.4220690 -4.2558792 1.9067232

H 2.5280440 -5.9629512 3.6431412

H -1.6083050 -5.9130832 4.1631702

H -1.8948640 -4.2051542 2.4526352

H -0.5144250 8.1792128 4.3046122

H -1.5282610 7.1734668 5.3699572

H 0.2347400 7.1747968 5.5706432

H -8.7369330 -1.4072692 -3.6290588

H -8.7001640 0.3205608 -4.0662888

H -7.9358490 -0.8765242 -5.1316298

H 1.5193700 -7.1433102 5.3994752

H -0.2440070 -7.1434432 5.5967432

H 0.5073540 -8.1527662 4.3359292

H 8.3290310 1.5480308 -4.4080438

H 8.9468880 0.0911138 -3.6022398

H 7.9705390 -0.0623562 -5.0856338

[CoP]^0^ M = 6

C 2.8598120 -0.7071815 -0.8046124

C 3.4880320 -1.8747135 -1.3155644

C 2.7766990 -2.9723875 -0.8316834

C 1.7100030 -2.4617745 -0.0577214

N 1.7475290 -1.0967385 -0.0580954

C 3.2597820 0.6609845 -0.9946684

C 0.6464570 -3.2264915 0.6228166

C -0.7266880 -2.9301805 0.2246876

C -1.8265190 -3.8274525 0.1150126

C -2.8973960 -3.1034445 -0.3881034

C -2.4504710 -1.7668065 -0.5931234

N -1.1166620 -1.6887415 -0.2101874

C -3.2531830 -0.6517755 -1.0054664

C -2.8536260 0.7166265 -0.8157024

C -3.4792580 1.8837485 -1.3308614

C -2.7697570 2.9818035 -0.8452444

C -1.7064810 2.4718525 -0.0661724

N -1.7443600 1.1067905 -0.0650424

C -0.6454930 3.2371195 0.6176946

C 0.7290650 2.9403655 0.2251546

C 1.8295900 3.8373225 0.1196446

C 2.9021790 3.1130045 -0.3792154

C 2.4557140 1.7764005 -0.5858014

N 1.1204880 1.6986815 -0.2080054

C -0.9788720 4.2187595 1.5525096

C -4.5408880 -0.9261345 -1.6037634

C 0.9762270 -4.2070835 1.5599086

C 4.5505350 0.9345015 -1.5866364

C -4.7464550 -2.0056035 -2.5253774

C -5.9617520 -2.2448075 -3.1006204

N -7.0641170 -1.4717965 -2.8100294

C -6.9225190 -0.4441455 -1.9038424

C -5.7175000 -0.1542645 -1.3295644

C -0.0138800 4.9450345 2.3643816

C -0.3822870 5.9111985 3.2459576

N -1.6986810 6.2601265 3.4551376

C -2.6621710 5.5508725 2.7686746

C -2.3519800 4.5699925 1.8817336

C 5.7259600 0.1626785 -1.3054504

C 6.9345720 0.4534225 -1.8714994

N 7.0860780 1.4943495 -2.7605214

C 5.9800000 2.2530465 -3.0748934

C 4.7610310 2.0130245 -2.5077074

C 2.3480920 -4.5576495 1.8951576

C 2.6548680 -5.5374565 2.7844446

N 1.6887530 -6.2462355 3.4677196

C 0.3731230 -5.8979655 3.2526336

C 0.0080800 -4.9328595 2.3685186

C -2.0522750 7.4120635 4.2795256

C -8.3801670 -1.8094235 -3.3536864

C 2.0392840 -7.3972225 4.2947226

C 8.3600720 1.7162735 -3.4455654

Co 0.0014860 0.0050535 0.0203576

H 4.3417210 -1.9042655 -1.9787704

H 2.9741990 -4.0202585 -1.0218694

H -1.8095970 -4.8871685 0.3297806

H -3.8951400 -3.4776085 -0.5729434

H -4.3300320 1.9128395 -1.9978134

H -2.9661380 4.0294875 -1.0376094

H 1.8121510 4.8970865 0.3340916

H 3.9007150 3.4869795 -0.5601134

H -3.9104170 -2.6277495 -2.8208194

H -6.1157160 -3.0328515 -3.8276554

H -7.8255250 0.1062275 -1.6688214

H -5.6771900 0.6552135 -0.6116024

H 1.0368500 4.6937255 2.3127206

H 0.3437290 6.4440445 3.8502646

H -3.6883850 5.8184775 2.9948336

H -3.1701040 4.0396935 1.4110666

H 5.6820500 -0.6444145 -0.5849904

H 7.8354030 -0.0996465 -1.6342384

H 6.1368230 3.0375165 -3.8051644

H 3.9273350 2.6368745 -2.8061974

H 3.1680080 -4.0276395 1.4272936

H 3.6802130 -5.8045065 3.0151616

H -0.3552270 -6.4304555 3.8544406

H -1.0425130 -4.6819785 2.3121316

H -2.0649360 8.3364125 3.6894676

H -3.0412230 7.2576405 4.7173656

H -1.3262270 7.5183485 5.0891886

H -8.9171020 -2.4853175 -2.6787534

H -8.9640720 -0.8966315 -3.4877784

H -8.2579280 -2.2945575 -4.3239824

H 3.0263930 -7.2420735 4.7364436

H 1.3099750 -7.5028995 5.1015386

H 2.0546310 -8.3221925 3.7056936

H 8.4849200 2.7819925 -3.6480784

H 9.1786190 1.3817655 -2.8054464

H 8.3936660 1.1631295 -4.3907974

[CoP]^0^ M = 8

C 2.8173126 -0.9321623 -0.6560663

C 3.4169746 -2.1618223 -1.0628113

C 2.6237096 -3.1875213 -0.5758303

C 1.5145686 -2.5819453 0.0791387

N 1.6281196 -1.2193753 0.0048157

C 3.3283576 0.3915057 -0.8862823

C 0.3534796 -3.2834363 0.6222707

C -0.9624684 -2.8447063 0.2254877

C -2.1061194 -3.6558193 -0.0441463

C -3.0931584 -2.8284003 -0.5508673

C -2.5650714 -1.4954803 -0.5662143

N -1.2585544 -1.5328743 -0.1120923

C -3.2782164 -0.3219093 -0.9482153

C -2.7886374 1.0013667 -0.7618643

C -3.4785804 2.2162167 -1.0746753

C -2.6915674 3.2637717 -0.6139403

C -1.5143114 2.6957757 -0.0517183

N -1.5744424 1.3231777 -0.1736093

C -0.3651824 3.3878777 0.5133807

C 0.9669396 2.9348997 0.1478547

C 2.1331306 3.7405727 -0.0319733

C 3.1453896 2.9078637 -0.4692903

C 2.6010516 1.5907457 -0.5665033

N 1.2698326 1.6375657 -0.1937443

C -0.5513664 4.4909267 1.3970227

C -4.6422724 -0.4925633 -1.5153153

C 0.5466946 -4.4143323 1.4712487

C 4.6594776 0.5336287 -1.4347133

C -4.8756694 -1.1839463 -2.7423003

C -6.1312884 -1.3614963 -3.2535633

N -7.2614494 -0.8265193 -2.6155013

C -7.0678004 -0.1892543 -1.3801773

C -5.8168134 -0.0133043 -0.8581903

C 0.5205936 5.1442377 2.1126967

C 0.3126556 6.2238477 2.9187137

N -0.9414784 6.7437117 3.1350877

C -2.0110714 6.0825587 2.5798267

C -1.8524424 4.9978507 1.7699167

C 5.7663206 -0.2870813 -1.0392763

C 7.0151066 -0.1216523 -1.5679883

N 7.2748816 0.8321567 -2.5254823

C 6.2390716 1.6341697 -2.9485183

C 4.9831036 1.5200387 -2.4237393

C 1.8444966 -4.9341213 1.8304827

C 1.9959946 -6.0429603 2.6092577

N 0.9191856 -6.7134293 3.1445897

C -0.3340694 -6.1862163 2.9338267

C -0.5340244 -5.0863293 2.1540657

C -1.1229734 7.9895287 3.8794747

C -8.5844044 -1.3129763 -2.9734223

C 1.0961816 -7.9806513 3.8521767

C 8.5902386 0.9150697 -3.1627753

Co 0.0024556 0.0523417 0.0380287

H 4.3048776 -2.2721333 -1.6689363

H 2.7651816 -4.2492533 -0.7299003

H -2.1542144 -4.7326753 0.0466637

H -4.0797894 -3.1147773 -0.8882633

H -4.4364124 2.2844217 -1.5721133

H -2.8931364 4.3218437 -0.7222653

H 2.1935816 4.8132947 0.0849367

H 4.1646946 3.1977957 -0.6804923

H -4.0334784 -1.5921553 -3.2943953

H -6.3245224 -1.8848243 -4.1828213

H -7.9680024 0.1719157 -0.8963293

H -5.7227804 0.5170357 0.0852007

H 1.5290766 4.7603737 2.0597557

H 1.1228616 6.7109507 3.4486847

H -2.9900834 6.4668577 2.8414057

H -2.7497154 4.5050167 1.4234647

H 5.6391916 -1.0277063 -0.2595673

H 7.8649216 -0.7103813 -1.2443163

H 6.4795656 2.3454047 -3.7293003

H 4.2083896 2.1699607 -2.8118523

H 2.7454686 -4.4382473 1.4967467

H 2.9715886 -6.4414393 2.8616327

H -1.1482284 -6.6870873 3.4445487

H -1.5415344 -4.6991203 2.0922447

H -1.0427394 8.8557747 3.2134757

H -2.1076724 7.9910267 4.3511557

H -0.3598134 8.0642657 4.6572897

H -8.8155544 -2.2763243 -2.4931893

H -9.3391624 -0.5824483 -2.6684403

H -8.6472104 -1.4412083 -4.0579083

H 2.0612236 -7.9811203 4.3634297

H 0.3043586 -8.0968273 4.5952557

H 1.0596476 -8.8249533 3.1545557

H 8.7962056 1.9505677 -3.4406393

H 9.3549186 0.5808657 -2.4591583

H 8.6264066 0.2861927 -4.0591103

[CoP]^–1^ M = 1

C 2.2565182 -1.2208606 -1.0286572

C 2.7268852 -2.5343306 -1.3019922

C 1.9813422 -3.4124326 -0.5197642

C 1.0232712 -2.6405266 0.1792998

N 1.1729112 -1.3227786 -0.1520052

C 2.7767072 0.0374044 -1.4772432

C -0.0559368 -3.1121556 1.0542438

C -1.3917018 -2.6067596 0.7198928

C -2.6317488 -3.2619106 0.7498308

C -3.5759478 -2.3913166 0.1605568

C -2.8939888 -1.2266426 -0.2124222

N -1.5626218 -1.3574806 0.1417788

C -3.4062598 0.0007644 -0.8437602

C -2.8965658 1.2552814 -0.2706392

C -3.5648988 2.4527544 0.0163278

C -2.6266158 3.3245954 0.6136318

C -1.4057618 2.6383714 0.6766188

N -1.5801448 1.3734614 0.1383858

C -0.0848088 3.1106724 1.1164988

C 1.0444892 2.6556224 0.3044198

C 2.1835562 3.3824174 -0.1311302

C 2.9423352 2.5259174 -0.9207782

C 2.2665392 1.2757494 -0.9601412

N 1.1011462 1.3892044 -0.2140582

C 0.0916062 3.9433764 2.2193978

C -4.3105038 -0.0274026 -1.8943462

C 0.1957392 -4.0114686 2.0843468

C 3.8905872 0.0981494 -2.3951272

C -4.8249868 -1.2555916 -2.5006492

C -5.7523958 -1.2524656 -3.4883822

N -6.2629398 -0.0837186 -4.0330972

C -5.7441758 1.1114514 -3.5584902

C -4.8173708 1.1665314 -2.5716672

C 1.3873802 4.4062894 2.7031568

C 1.5141812 5.2643374 3.7471608

N 0.4276242 5.7324074 4.4614208

C -0.8164498 5.2396654 4.1162778

C -1.0007768 4.3853054 3.0781648

C 5.0143892 -0.7939006 -2.3666072

C 6.0407162 -0.6929676 -3.2620942

N 6.0490052 0.2635244 -4.2517412

C 4.9926062 1.1487174 -4.3149212

C 3.9619922 1.0984184 -3.4209422

C 1.5159922 -4.5491056 2.3937958

C 1.7195352 -5.4630816 3.3756978

N 0.6987332 -5.9221396 4.1888698

C -0.5566318 -5.3751466 4.0009628

C -0.8191238 -4.4666496 3.0281258

C 0.5708942 6.8108604 5.4316848

C -7.4494558 -0.1044716 -4.8757382

C 0.9047672 -7.0490156 5.0900248

C 7.0884022 0.2735114 -5.2809062

Co -0.2230198 0.0191174 0.0129548

H 3.5062672 -2.8088116 -1.9973292

H 2.0747312 -4.4901176 -0.4763582

H -2.8112858 -4.2730126 1.0926418

H -4.6354568 -2.5752726 0.0297838

H -4.6140368 2.6534404 -0.1625422

H -2.7977558 4.3493264 0.9192558

H 2.3943842 4.4203254 0.0897368

H 3.8920122 2.7489614 -1.3876492

H -4.4394388 -2.2126516 -2.1725922

H -6.1275738 -2.1702886 -3.9298732

H -6.1117818 2.0040364 -4.0547752

H -4.4232598 2.1383964 -2.3028002

H 2.2974982 4.0423114 2.2448198

H 2.4825742 5.6080754 4.0950098

H -1.6369018 5.5646094 4.7472648

H -1.9995688 4.0049974 2.9090978

H 5.1045832 -1.5293806 -1.5782282

H 6.9100412 -1.3385086 -3.2223312

H 5.0292912 1.8708714 -5.1213602

H 3.1568852 1.8153094 -3.5277812

H 2.3809052 -4.2075416 1.8389928

H 2.7028022 -5.8669766 3.5920578

H -1.3167408 -5.7051406 4.7011478

H -1.8150238 -4.0453366 2.9851028

H 0.4973492 7.7966314 4.9546498

H -0.2124698 6.7277074 6.1893958

H 1.5421642 6.7313904 5.9268918

H -8.3755918 -0.0835336 -4.2846332

H -7.4413418 0.7630034 -5.5416762

H -7.4478218 -1.0099126 -5.4891362

H 1.9213732 -7.0170736 5.4910128

H 0.2015642 -6.9831806 5.9242428

H 0.7570662 -8.0094646 4.5792738

H 7.2827762 1.3017274 -5.5935452

H 8.0079482 -0.1494776 -4.8720132

H 6.7788112 -0.3149996 -6.1519782

[CoP]^–1^ M = 3

C 2.9108500 -0.6677258 -0.7862720

C 3.4328380 -1.8280018 -1.3933930

C 2.7214470 -2.9344668 -0.8784860

C 1.7670320 -2.4257328 0.0026270

N 1.8606640 -1.0503658 0.0536250

C 3.3239230 0.7317802 -0.9614670

C 0.7137820 -3.1744088 0.7294560

C -0.6568960 -2.9113938 0.3086380

C -1.7646740 -3.8047708 0.2417730

C -2.8151870 -3.1107498 -0.3424210

C -2.3513170 -1.7939888 -0.6249650

N -1.0268510 -1.6999688 -0.2182720

C -3.1389540 -0.6994708 -1.1178440

C -2.7562780 0.6735202 -0.9260980

C -3.3427160 1.8382682 -1.4906570

C -2.6805390 2.9391832 -0.9430590

C -1.6844370 2.4315732 -0.0785790

N -1.7143920 1.0680272 -0.0854240

C -0.6931030 3.1800022 0.7222160

C 0.7192320 2.9203092 0.4168540

C 1.8350760 3.7657162 0.5538760

C 2.9491260 3.0667812 0.0546320

C 2.4989350 1.8144712 -0.4012200

N 1.1342370 1.7318872 -0.1712650

C -1.1334320 4.0752952 1.6895210

C -4.3851040 -1.0027608 -1.7813360

C 1.0538250 -4.1097808 1.7018690

C 4.5239580 1.0617482 -1.6082060

C -4.5363830 -2.1356958 -2.6501440

C -5.7104270 -2.4060008 -3.2918790

N -6.8264880 -1.6136788 -3.1241110

C -6.7398920 -0.5360318 -2.2693130

C -5.5754440 -0.2151198 -1.6326040

C -0.2631230 4.7634832 2.6402650

C -0.7301870 5.6605002 3.5439800

N -2.0708010 5.9828402 3.6512150

C -2.9531350 5.3031872 2.8269090

C -2.5428610 4.3848212 1.9176490

C 5.6816410 0.1764272 -1.7202500

C 6.8272990 0.5292692 -2.3534590

N 6.9949970 1.7626722 -2.9690980

C 5.9133130 2.6373302 -2.9212240

C 4.7680980 2.3480042 -2.2560390

C 2.4359870 -4.4345648 2.0276770

C 2.7678230 -5.3685808 2.9542940

N 1.8182930 -6.0570988 3.6867810

C 0.4928970 -5.7425138 3.4657260

C 0.1030000 -4.8230128 2.5456760

C -2.5124910 7.1141052 4.4553840

C -8.1045070 -1.9848268 -3.7308690

C 2.1948630 -7.1763238 4.5429160

C 8.0906440 1.9888102 -3.8980330

Co 0.0811190 0.0141652 0.0155600

H 4.2129250 -1.8715308 -2.1406220

H 2.8722410 -3.9816858 -1.1181300

H -1.7629330 -4.8472978 0.5299570

H -3.8122960 -3.4876658 -0.5280100

H -4.1384950 1.8647242 -2.2229460

H -2.8730070 3.9872812 -1.1392150

H 1.8290700 4.7872022 0.9113320

H 3.9708610 3.4242642 0.0278040

H -3.6861930 -2.7768758 -2.8483240

H -5.8199540 -3.2355418 -3.9799570

H -7.6541000 0.0275702 -2.1269250

H -5.5808270 0.6331172 -0.9598140

H 0.7906120 4.5212962 2.6727490

H -0.0758590 6.1550172 4.2545740

H -4.0014510 5.5403512 2.9742850

H -3.3036120 3.8706892 1.3429060

H 5.6730290 -0.7756808 -1.2059590

H 7.6967880 -0.1201668 -2.3752900

H 6.0506680 3.5727412 -3.4533130

H 3.9830520 3.0940082 -2.2651560

H 3.2349250 -3.9222328 1.5053970

H 3.7990940 -5.6178678 3.1794630

H -0.2200890 -6.2620858 4.0969440

H -0.9525500 -4.5925358 2.4874290

H -2.4913590 8.0530442 3.8861180

H -3.5336850 6.9392562 4.8047630

H -1.8620860 7.2189922 5.3279060

H -8.6715300 -2.6502128 -3.0697790

H -8.6915800 -1.0844418 -3.9227400

H -7.9220880 -2.4940148 -4.6794560

H 3.1785140 -6.9876078 4.9802280

H 1.4688600 -7.2769988 5.3536480

H 2.2317810 -8.1187998 3.9820100

H 8.3184980 3.0575172 -3.9448770

H 8.9829600 1.4623572 -3.5470640

H 7.8545400 1.6366682 -4.9130640

[CoP]^–1^ M = 5

C 2.7625740 -0.6821499 -0.9159434

C 3.3408900 -1.8540009 -1.4745664

C 2.6662150 -2.9472269 -0.9269464

C 1.6696180 -2.4276529 -0.0701474

N 1.7126950 -1.0640269 -0.0810034

C 3.1610890 0.6867841 -1.1084164

C 0.6666120 -3.1651179 0.7258386

C -0.7415590 -2.8916829 0.4137966

C -1.8673140 -3.7242359 0.5492606

C -2.9709100 -3.0153119 0.0409966

C -2.5046560 -1.7703639 -0.4186974

N -1.1404110 -1.7013269 -0.1817824

C -3.3155800 -0.6844999 -0.9926204

C -2.8945860 0.7132521 -0.8166174

C -3.4051500 1.8776311 -1.4252674

C -2.6848130 2.9780911 -0.9099274

C -1.7373610 2.4616501 -0.0257764

N -1.8436320 1.0872651 0.0257366

C -0.6814530 3.2019571 0.7054916

C 0.6891410 2.9256801 0.2947996

C 1.8071070 3.8070371 0.2390256

C 2.8558350 3.1012611 -0.3337984

C 2.3802050 1.7897121 -0.6218884

N 1.0513450 1.7097921 -0.2293284

C -1.0196240 4.1425351 1.6743756

C -4.5077820 -1.0112699 -1.6547354

C 1.0935960 -4.0651249 1.6952736

C 4.4158440 0.9756721 -1.7604494

C -4.7511600 -2.3016789 -2.2971164

C -5.8804780 -2.5834829 -2.9909114

N -6.9256990 -1.6723819 -3.1261854

C -6.7916150 -0.4684479 -2.4476674

C -5.6594050 -0.1220449 -1.7865654

C -0.0682620 4.8473541 2.5242886

C -0.4558370 5.7720211 3.4402706

N -1.7795560 6.0999381 3.6509246

C -2.7303070 5.4192211 2.9130346

C -2.4007210 4.4806511 1.9900886

C 5.5957200 0.1727631 -1.6027644

C 6.7697520 0.4800261 -2.2278004

N 6.8844940 1.5717691 -3.0614764

C 5.7732550 2.3655171 -3.2548974

C 4.5896830 2.1081921 -2.6254974

C 2.4985680 -4.3888789 1.9299806

C 2.8951770 -5.3123189 2.8402616

N 2.0020610 -5.9836989 3.6594626

C 0.6656560 -5.6467319 3.5471646

C 0.2121090 -4.7439509 2.6421536

C -2.1512580 7.2234861 4.5036086

C -8.2236250 -2.1068379 -3.6179614

C 2.4284300 -7.1195189 4.4655986

C 8.1118240 1.8099281 -3.8201854

Co -0.0732380 0.0071321 0.0050756

H 4.1409500 -1.8897459 -2.2017734

H 2.8489770 -3.9975659 -1.1202444

H -1.8747960 -4.7440829 0.9111446

H -3.9965030 -3.3613349 0.0104096

H -4.1837970 1.9282801 -2.1734704

H -2.8260140 4.0263921 -1.1506854

H 1.8144400 4.8491601 0.5283286

H 3.8584430 3.4675441 -0.5106444

H -3.9712790 -3.0531769 -2.2852904

H -6.0175830 -3.5239169 -3.5146234

H -7.6607770 0.1803961 -2.4774244

H -5.6576030 0.8307161 -1.2738914

H 0.9853040 4.6062241 2.4746386

H 0.2575510 6.2853231 4.0761356

H -3.7607490 5.6783961 3.1306326

H -3.2008910 3.9749721 1.4632926

H 5.5853570 -0.6739549 -0.9281124

H 7.6740700 -0.0986529 -2.0821024

H 5.8980890 3.1929741 -3.9428804

H 3.7507720 2.7628661 -2.8277004

H 3.2673250 -3.8818739 1.3596496

H 3.9403110 -5.5602319 2.9923706

H 0.0030340 -6.1346109 4.2546396

H -0.8389630 -4.4901689 2.6708926

H -2.1706890 8.1663741 3.9425816

H -3.1416720 7.0466731 4.9304056

H -1.4326160 7.3150301 5.3219586

H -8.8268180 -2.5867409 -2.8330514

H -8.7762320 -1.2462609 -4.0048094

H -8.0857650 -2.8210459 -4.4350594

H 3.4498910 -6.9553889 4.8192896

H 1.7732140 -7.2175579 5.3352896

H 2.3997960 -8.0580359 3.8960466

H 8.2439280 2.8830451 -3.9736244

H 8.9661160 1.4281971 -3.2574734

H 8.0716730 1.3094241 -4.7944144

[CoP]^–2^ M = 2

C 2.6745426 -1.0827270 -0.8123411

C 3.2016466 -2.3688570 -1.0964911

C 2.3964036 -3.3064500 -0.4391721

C 1.3537926 -2.6008270 0.1855989

N 1.4981186 -1.2489060 -0.0666991

C 3.2346466 0.2063600 -1.1132731

C 0.2028046 -3.1567520 0.8924519

C -1.0900314 -2.6915370 0.4220789

C -2.2936524 -3.4218090 0.2934259

C -3.2085824 -2.6032090 -0.3685021

C -2.5669234 -1.3661980 -0.6301031

N -1.2639484 -1.4368990 -0.1466371

C -3.1878864 -0.1825220 -1.1680591

C -2.6341424 1.1085880 -0.8645491

C -3.1580674 2.3926410 -1.1643821

C -2.3635864 3.3349180 -0.5016131

C -1.3291124 2.6345750 0.1431349

N -1.4688264 1.2804400 -0.1032121

C -0.1902844 3.1973310 0.8628359

C 1.1104756 2.7260440 0.4236879

C 2.3184526 3.4537860 0.3147409

C 3.2438726 2.6310680 -0.3256871

C 2.6054876 1.3931550 -0.5927971

N 1.2937986 1.4682320 -0.1351601

C -0.3659934 4.1803670 1.8506849

C -4.4192794 -0.3290930 -1.9077511

C 0.3611426 -4.1253840 1.8951259

C 4.4802386 0.3489910 -1.8299151

C -4.6497934 -1.4336960 -2.8106921

C -5.7908014 -1.5733920 -3.5385101

N -6.8331304 -0.6371910 -3.4735991

C -6.6960324 0.3853870 -2.5294351

C -5.5524974 0.5605360 -1.8102221

C 0.7140496 4.7387510 2.6542629

C 0.5189846 5.7297970 3.5609729

N -0.7348634 6.2660040 3.8205969

C -1.8155014 5.6953940 3.1577729

C -1.6676724 4.6991910 2.2477869

C 5.6078566 -0.5472080 -1.7178741

C 6.7649156 -0.3738830 -2.4153531

N 6.9550506 0.7027960 -3.2856251

C 5.8781116 1.5842880 -3.4439011

C 4.7217776 1.4443170 -2.7397201

C 1.6561756 -4.6382350 2.3223899

C 1.7881576 -5.6202490 3.2497139

N 0.6963056 -6.1813300 3.9025569

C -0.5536374 -5.6524770 3.6099859

C -0.7335754 -4.6758390 2.6850419

C -0.8799164 7.5159880 4.5496539

C -8.1569704 -1.0046090 -3.9488231

C 0.8291286 -7.4204140 4.6522039

C 7.9996146 0.6485660 -4.2944781

Co 0.0140836 0.0157960 -0.0728081

H 4.0627996 -2.5870520 -1.7114301

H 2.5147026 -4.3836930 -0.4369451

H -2.4451704 -4.4511560 0.5914239

H -4.2335884 -2.8443540 -0.6178581

H -4.0110694 2.6058830 -1.7923751

H -2.4827324 4.4120150 -0.5090041

H 2.4661496 4.4846790 0.6088489

H 4.2733356 2.8700830 -0.5580611

H -3.8677354 -2.1727130 -2.9499891

H -5.9397364 -2.3828680 -4.2448711

H -7.5597074 1.0315520 -2.4161841

H -5.5345864 1.3742590 -1.0951841

H 1.7119726 4.3277020 2.5709149

H 1.3283426 6.1315800 4.1621369

H -2.7891894 6.0796260 3.4437609

H -2.5656574 4.2738340 1.8163719

H 5.5747896 -1.3616170 -1.0041391

H 7.6221846 -1.0278080 -2.2943881

H 6.0373696 2.3886160 -4.1536181

H 3.9407626 2.1802250 -2.8997581

H 2.5612206 -4.2192290 1.8994049

H 2.7567886 -5.9998410 3.5582559

H -1.3736334 -6.0478580 4.2009319

H -1.7308274 -4.2693540 2.5747379

H -0.7888864 8.3915450 3.8909129

H -1.8597564 7.5465160 5.0350659

H -0.1093814 7.5812180 5.3232969

H -8.7004184 -1.6356020 -3.2274361

H -8.7453284 -0.1006930 -4.1313811

H -8.0689634 -1.5538520 -4.8910331

H 1.8002826 -7.4430500 5.1552139

H 0.0452016 -7.4747450 5.4131609

H 0.7501126 -8.3058700 4.0051609

H 8.2568346 1.6632960 -4.6128151

H 8.8945356 0.1833190 -3.8706271

H 7.6940536 0.0717890 -5.1818191

[CoP]^–2^ M = 4

C 2.8124635 -1.5168633 -0.1277989

C 3.3552225 -2.7540103 0.2441401

C 2.3146435 -3.5079233 0.8318681

C 1.1593975 -2.7123923 0.8020591

N 1.4738215 -1.4924463 0.2243971

C 3.4635005 -0.3546213 -0.7539749

C -0.2228085 -3.0573083 1.1672271

C -1.2544255 -2.5249823 0.2679001

C -2.3784735 -3.1680653 -0.2868759

C -3.0337225 -2.2375153 -1.1095769

C -2.3086775 -1.0221403 -1.0237239

N -1.2004785 -1.2265563 -0.2036009

C -2.6728415 0.2449447 -1.5642009

C -2.0464065 1.4508497 -1.1368869

C -2.4797445 2.7824357 -1.3617519

C -1.6433865 3.6216337 -0.6069399

C -0.6979485 2.8042907 0.0416561

N -0.9283995 1.4861367 -0.3036629

C 0.4192725 3.1841327 0.9165621

C 1.7002355 2.5314117 0.6081501

C 2.9976315 3.0657767 0.6106291

C 3.8591275 2.0716107 0.0948021

C 3.0691305 0.9553627 -0.2099559

N 1.7538575 1.2362667 0.1159411

C 0.2693695 4.1113607 1.9397861

C -3.8587345 0.3175247 -2.4576249

C -0.5373545 -3.8476333 2.2656961

C 4.3870605 -0.4831773 -1.7801149

C -3.8533885 -0.2380423 -3.7720449

C -4.9558335 -0.2177273 -4.5817629

N -6.1505585 0.4019677 -4.1755069

C -6.2024605 0.9051837 -2.8636439

C -5.1058715 0.8797487 -2.0469569

C 1.3345125 4.4859187 2.8691261

C 1.1753765 5.4363817 3.8219601

N -0.0227675 6.1079057 4.0167301

C -1.0956045 5.7265097 3.2228411

C -0.9913395 4.7803067 2.2584471

C 4.7843625 -1.7613863 -2.3736279

C 5.7507585 -1.8619553 -3.3174709

N 6.4514605 -0.7611983 -3.7926569

C 5.9982095 0.4876967 -3.3880609

C 5.0374475 0.6467027 -2.4466279

C 0.4500225 -4.3614793 3.2142101

C 0.1214805 -5.1798033 4.2432451

N -1.1850295 -5.5674703 4.5038421

C -2.1750205 -5.0321273 3.6914081

C -1.9011715 -4.2083033 2.6510191

C -0.0836525 7.3115507 4.8319661

C -7.3909565 0.0713137 -4.8588929

C -1.4736205 -6.6686083 5.4099101

C 7.3611545 -0.8901973 -4.9214859

Co 0.2672955 0.0031977 -0.0080229

H 4.3877945 -3.0574383 0.1196921

H 2.3746965 -4.5297643 1.1858221

H -2.6556505 -4.2024533 -0.1210419

H -3.9388765 -2.3847163 -1.6854049

H -3.3252485 3.0702997 -1.9736569

H -1.6871215 4.7025907 -0.5438489

H 3.2700125 4.0738517 0.8980271

H 4.9329965 2.1424717 -0.0297499

H -2.9466085 -0.7022153 -4.1513879

H -4.9666085 -0.6375413 -5.5812259

H -7.1521345 1.3380327 -2.5698689

H -5.1924475 1.3156947 -1.0554659

H 2.2836645 3.9667827 2.8273331

H 1.9716245 5.7034897 4.5095501

H -2.0359505 6.2187857 3.4497931

H -1.8910285 4.5025237 1.7231111

H 4.2828545 -2.6696653 -2.0648399

H 6.0320535 -2.8142283 -3.7552039

H 6.4692495 1.3328397 -3.8793339

H 4.7382405 1.6561317 -2.1947609

H 1.4850105 -4.0575413 3.1209151

H 0.8622665 -5.5529103 4.9434451

H -3.1911825 -5.2947473 3.9681401

H -2.7372415 -3.7891563 2.1049021

H 0.1811315 8.2120957 4.2601511

H -1.0956435 7.4355577 5.2278871

H 0.6072525 7.2187487 5.6747941

H -7.8070005 -0.8919273 -4.5235539

H -8.1330645 0.8533297 -4.6726339

H -7.2124685 0.0156737 -5.9367759

H -0.7570045 -6.6610983 6.2362361

H -2.4787745 -6.5489153 5.8243521

H -1.4167615 -7.6441593 4.9066711

H 8.0939215 -0.0790393 -4.8970669

H 7.8994095 -1.8392963 -4.8521129

H 6.8324995 -0.8562733 -5.8849599

[CoP]^–2^_2 M = 4

C -1.0871835 -2.7762741 0.4025408

C -0.7008515 -3.7376691 1.3480908

C 0.7048065 -3.7125171 1.4144058

C 1.1437135 -2.7387181 0.5036638

N 0.0397035 -2.1031091 -0.0628332

C -2.4299005 -2.5092041 -0.1035062

C 2.5118695 -2.4311241 0.1010468

C 2.7837465 -1.0828931 -0.3874832

C 3.7539275 -0.6869311 -1.3199512

C 3.7295915 0.7193169 -1.3716122

C 2.7467325 1.1488929 -0.4657582

N 2.1058645 0.0387739 0.0831338

C 2.4346955 2.5125399 -0.0532382

C 1.0820885 2.7781989 0.4283298

C 0.6775525 3.7364489 1.3690948

C -0.7292415 3.7119059 1.4069358

C -1.1503375 2.7403719 0.4850958

N -0.0348345 2.1062069 -0.0609342

C -2.5096605 2.4337709 0.0546708

C -2.7704595 1.0865389 -0.4443632

C -3.7191305 0.6917159 -1.3988612

C -3.6936835 -0.7146421 -1.4515222

C -2.7319785 -1.1452081 -0.5241622

N -2.1034375 -0.0357561 0.0398278

C -3.5273625 3.3915369 0.1066628

C 3.3922525 3.5304259 -0.1058792

C 3.5278925 -3.3896291 0.1699738

C -3.3884665 -3.5252111 -0.1717112

C 4.8354215 3.3027739 -0.1808722

C 5.7425455 4.3086209 -0.2083502

N 5.3786995 5.6513099 -0.1810022

C 4.0147665 5.9269759 -0.1675112

C 3.0717125 4.9568139 -0.0940732

C -4.9538705 3.0724649 0.0758788

C -5.9239925 4.0155409 0.1487828

N -5.6474905 5.3791809 0.1794028

C -4.3047045 5.7413879 0.2258828

C -3.2993915 4.8336759 0.1998458

C -3.0721535 -4.9534081 -0.1485882

C -4.0140515 -5.9213571 -0.2529422

N -5.3721385 -5.6403471 -0.3756892

C -5.7365095 -4.2986071 -0.3265872

C -4.8288935 -3.2940671 -0.2689112

C 3.2969805 -4.8311081 0.2494368

C 4.3016765 -5.7385171 0.3085358

N 5.6423165 -5.3735631 0.3758688

C 5.9234865 -4.0141421 0.2710368

C 4.9558685 -3.0719131 0.1662378

C -6.6839405 6.3345699 0.5361478

C 6.3375625 6.6850929 -0.5362442

C 6.6881985 -6.3622881 0.1678788

C -6.3638395 -6.6826701 -0.1642132

Co 0.0014895 0.0015149 -0.0007042

H -1.3682275 -4.3792961 1.9106108

H 1.3366085 -4.3291611 2.0416278

H 4.4005935 -1.3485121 -1.8835592

H 4.3504815 1.3574129 -1.9881222

H 1.3337035 4.3777939 1.9450548

H -1.3731425 4.3255429 2.0247078

H -4.3548485 1.3537929 -1.9742722

H -4.3024645 -1.3521901 -2.0806352

H 5.2136195 2.2885569 -0.1511452

H 6.8116175 4.1209509 -0.2143392

H 3.7556975 6.9797179 -0.2133402

H 2.0342485 5.2666789 -0.0736542

H -5.2642945 2.0355469 0.0408538

H -6.9773925 3.7569669 0.1803178

H -4.1162635 6.8101669 0.2456588

H -2.2845285 5.2111469 0.1857328

H -2.0357385 -5.2647901 -0.1046542

H -3.7570805 -6.9754001 -0.2839962

H -6.8047895 -4.1088171 -0.3426752

H -5.2063395 -2.2795731 -0.2432892

H 2.2832065 -5.2090911 0.2085988

H 4.1126735 -6.8070451 0.3111568

H 6.9768735 -3.7566811 0.3165828

H 5.2668105 -2.0348581 0.1372378

H -6.8268575 6.4083689 1.6246308

H -6.4173455 7.3241539 0.1535718

H -7.6320785 6.0347519 0.0807308

H 6.4239765 6.8176349 -1.6250742

H 6.0318235 7.6372669 -0.0933952

H 7.3227395 6.4228359 -0.1395782

H 6.8687105 -6.5607981 -0.8992042

H 7.6209065 -6.0092541 0.6174408

H 6.4075975 -7.3012121 0.6534578

H -6.5729075 -6.8497801 0.9030708

H -7.2977685 -6.4072811 -0.6622872

H -6.0074745 -7.6212381 -0.5987232

[CoP]^–2^_3 M = 4

C -0.7419434 2.9279472 -0.5196397

C -0.1656204 4.2081132 -0.4690937

C 1.1011016 4.0770052 0.1540753

C 1.2688446 2.7219112 0.4736363

N 0.1423266 2.0352062 0.0660513

C -2.0260154 2.4732362 -1.1063967

C 2.4011696 2.0249692 1.1383223

C 2.8551226 0.7377512 0.5595263

C 4.1374256 0.1643802 0.5138133

C 4.0127436 -1.1001608 -0.1131177

C 2.6600216 -1.2713038 -0.4396827

N 1.9678876 -0.1481428 -0.0317287

C 1.9762336 -2.4109088 -1.1036437

C 0.6806446 -2.8654968 -0.5371597

C 0.0992076 -4.1459038 -0.4950837

C -1.1752484 -4.0138538 0.1101893

C -1.3443634 -2.6606368 0.4317973

N -0.2119314 -1.9735368 0.0391583

C -2.4790634 -1.9706708 1.0985363

C -2.9302624 -0.6770708 0.5330213

C -4.2110074 -0.1004778 0.4847143

C -4.0781524 1.1728032 -0.1244627

C -2.7235914 1.3416292 -0.4417087

N -2.0380014 0.2097802 -0.0487647

C -3.0553694 -2.5076478 2.2429643

C 2.5448726 -2.9977368 -2.2305617

C 2.9794206 2.5546742 2.2855123

C -2.5446504 3.0361182 -2.2655867

C 3.8183276 -2.5702378 -2.8156457

C 4.4275756 -3.2377568 -3.8247387

N 3.8632416 -4.3469008 -4.4372557

C 2.5868616 -4.7036088 -4.0291537

C 1.9385136 -4.0743048 -3.0194987

C -4.0972024 -1.8496268 3.0355033

C -4.7144354 -2.4478548 4.0825283

N -4.3808374 -3.7210008 4.5203673

C -3.3080164 -4.3376788 3.8930613

C -2.6510314 -3.7788688 2.8483173

C -1.8821304 4.0759142 -3.0568717

C -2.4616824 4.6653482 -4.1303457

N -3.7396854 4.3420342 -4.5630247

C -4.3396254 3.2444752 -3.9599847

C -3.8014584 2.6140202 -2.8887487

C 2.5737796 3.8214272 2.8995153

C 3.2211216 4.3682752 3.9565093

N 4.2891006 3.7450692 4.5854423

C 4.6326646 2.4809852 4.1302223

C 4.0236156 1.8937162 3.0720403

C -5.2493564 -4.4529718 5.4288713

C 4.6480236 -5.2183028 -5.2974157

C 5.1390446 4.4623982 5.5226633

C -4.2720104 4.8917692 -5.8009977

Co -0.0348604 0.0307792 0.0043323

H -0.6186174 5.1314162 -0.8098987

H 1.8152946 4.8721912 0.3356603

H 5.0588826 0.6199952 0.8557623

H 4.8124536 -1.8085768 -0.2977697

H 0.5556106 -5.0723398 -0.8198567

H -1.8936144 -4.8080478 0.2793393

H -5.1348024 -0.5571268 0.8186813

H -4.8727694 1.8886092 -0.3035917

H 4.3036136 -1.6788358 -2.4382657

H 5.3792456 -2.9168118 -4.2368187

H 2.1233056 -5.4987388 -4.6051837

H 0.9159956 -4.3662148 -2.8239427

H -4.3661254 -0.8255618 2.8117093

H -5.4792064 -1.9446318 4.6662623

H -3.0081084 -5.2894388 4.3205133

H -1.7858494 -4.3007838 2.4598703

H -0.8735944 4.3746892 -2.8039307

H -1.9513464 5.4228552 -4.7163277

H -5.2734124 2.9221132 -4.4092667

H -4.3320254 1.7603412 -2.4874517

H 1.7109406 4.3472632 2.5112683

H 2.9165776 5.3146832 4.3925993

H 5.3998456 1.9748502 4.7081423

H 4.3055286 0.8767912 2.8320443

H -6.0797664 -4.9440558 4.9021453

H -4.6692504 -5.2179708 5.9525413

H -5.6664614 -3.7671848 6.1726823

H 5.1494546 -6.0163848 -4.7318277

H 3.9976566 -5.6794318 -6.0469877

H 5.4095456 -4.6298628 -5.8169757

H 4.5445376 5.2067712 6.0596773

H 5.5554306 3.7618022 6.2528143

H 5.9697476 4.9763702 5.0187563

H -5.3647354 4.9027862 -5.7592027

H -3.9244844 5.9208152 -5.9239997

H -3.9608334 4.3085782 -6.6795537

[CoP]^–2^ M = 6

C 2.6316346 -1.2191098 -0.8073519

C 3.2479176 -2.4932128 -0.8812189

C 2.3764546 -3.4073088 -0.2744779

C 1.2317686 -2.6934238 0.1308971

N 1.3757746 -1.3587538 -0.2123209

C 3.2118216 0.0269172 -1.1667199

C -0.0004334 -3.2233778 0.7130691

C -1.2324574 -2.6931388 0.1307581

C -2.3771884 -3.4067788 -0.2749059

C -3.2484324 -2.4924388 -0.8816039

C -2.6319664 -1.2184418 -0.8074169

N -1.3762054 -1.3583908 -0.2122389

C -3.2119384 0.0277552 -1.1665359

C -2.6315994 1.2680042 -0.7879119

C -3.2569694 2.5387272 -0.8326169

C -2.3850734 3.4477292 -0.2193569

C -1.2320924 2.7337922 0.1633211

N -1.3718824 1.4043742 -0.2022739

C 0.0004226 3.2581282 0.7493141

C 1.2326656 2.7335202 0.1629701

C 2.3856626 3.4472092 -0.2201079

C 3.2572766 2.5379592 -0.8334099

C 2.6317246 1.2673422 -0.7883149

N 1.3721536 1.4040192 -0.2024269

C 0.0006726 4.2207722 1.7574821

C -4.5570214 0.0425622 -1.8001819

C -0.0006164 -4.2028768 1.7039301

C 4.5569246 0.0413652 -1.8003369

C -4.7515884 0.3979722 -3.1680499

C -5.9898584 0.4295322 -3.7489959

N -7.1523584 0.1494012 -3.0091239

C -6.9860374 -0.2530878 -1.6727479

C -5.7513794 -0.2804818 -1.0858059

C 1.2111016 4.7584152 2.3733021

C 1.1844546 5.7397912 3.3083771

N 0.0011656 6.2847822 3.7865191

C -1.1823644 5.7399922 3.3087501

C -1.2094724 4.7586242 2.3736801

C 5.7512486 -0.2812698 -1.0857079

C 6.9859356 -0.2541988 -1.6725979

N 7.1523296 0.1475312 -3.0091929

C 5.9898666 0.4272362 -3.7492879

C 4.7515616 0.3959922 -3.1683909

C 1.2098026 -4.7515328 2.3099931

C 1.1825896 -5.7496328 3.2271231

N -0.0009994 -6.3026608 3.6950571

C -1.1843994 -5.7494088 3.2269001

C -1.2112514 -4.7513008 2.3097691

C 0.0013896 7.5190122 4.5569141

C -8.3753084 -0.2020018 -3.7126829

C -0.0011894 -7.5500398 4.4441021

C 8.3753046 -0.2043018 -3.7124959

Co -0.0000354 0.0222872 -0.1488359

H 4.2259676 -2.6894548 -1.3017419

H 2.5166736 -4.4766058 -0.1720759

H -2.5175984 -4.4760668 -0.1726699

H -4.2264634 -2.6884508 -1.3022779

H -4.2381834 2.7351162 -1.2459809

H -2.5302034 4.5144502 -0.0987609

H 2.5310006 4.5139242 -0.0996989

H 4.2384266 2.7341172 -1.2470329

H -3.8916364 0.6615092 -3.7788139

H -6.1536084 0.7037512 -4.7850249

H -7.9004244 -0.4989968 -1.1445039

H -5.6859354 -0.5642178 -0.0388519

H 2.1755006 4.3486602 2.1000231

H 2.0900176 6.1321692 3.7600491

H -2.0877194 6.1325242 3.7607061

H -2.1740264 4.3490262 2.1007131

H 5.6857466 -0.5643928 -0.0385919

H 7.9002976 -0.4997918 -1.1441629

H 6.1536706 0.7008782 -4.7854609

H 3.8916436 0.6591792 -3.7793539

H 2.1742746 -4.3376618 2.0429331

H 2.0878856 -6.1509988 3.6713541

H -2.0898534 -6.1506088 3.6709591

H -2.1755944 -4.3372558 2.0425151

H 0.0013916 8.4085412 3.9113291

H -0.8854664 7.5524852 5.1961071

H 0.8884216 7.5523142 5.1958741

H -8.3715784 -1.2481328 -4.0576979

H -9.2343834 -0.0580698 -3.0508249

H -8.4982774 0.4494742 -4.5829959

H 0.8857156 -7.5945108 5.0825411

H -0.8881874 -7.5943148 5.0824271

H -0.0012444 -8.4281948 3.7831791

H 8.4983466 0.4466972 -4.5831559

H 9.2343486 -0.0600438 -3.0506699

H 8.3715506 -1.2506208 -4.0569429

[CoP]^–2^_2 M = 6

C 2.6451115 -1.3609819 0.4734478

C 3.9262295 -1.0196959 0.9862418

C 4.0713675 0.3501331 0.8386788

C 2.8902475 0.8445791 0.2282728

N 1.9716515 -0.1969019 0.0717288

C 2.1708175 -2.6675019 0.2482188

C 2.6806465 2.1993281 -0.1917602

C 1.3431435 2.6708531 -0.3807652

C 0.9633475 3.9498631 -0.8668932

C -0.4003415 4.0859791 -0.6617382

C -0.8651355 2.8652981 -0.1054272

N 0.1967615 1.9631051 0.0104318

C -2.1900095 2.6144961 0.3130058

C -2.6679455 1.3039751 0.5072088

C -3.9594925 0.9574031 0.9908598

C -4.1068675 -0.4087579 0.8146238

C -2.9155915 -0.8960659 0.2174308

N -1.9912845 0.1460101 0.0998108

C -2.7019245 -2.2415399 -0.2251552

C -1.3640435 -2.7061819 -0.4279602

C -0.9850535 -3.9688999 -0.9569172

C 0.3785415 -4.1109899 -0.7614652

C 0.8451855 -2.9078819 -0.1686022

N -0.2180525 -2.0105509 -0.0157392

C -3.8308345 -3.1078939 -0.4248342

C -3.1139125 3.7647901 0.4852758

C 3.8127735 3.0647951 -0.3669362

C 3.0943175 -3.8209249 0.3916078

C -2.8085425 4.8655801 1.3420208

C -3.6437225 5.9389981 1.4845468

N -4.8827815 5.9943031 0.8242948

C -5.1808615 4.9566611 -0.0752242

C -4.3471105 3.8833511 -0.2258892

C -3.8360085 -4.5236479 -0.1210572

C -4.9221465 -5.3184209 -0.3181262

N -6.0972325 -4.8425259 -0.8997132

C -6.1591675 -3.4710559 -1.1608272

C -5.1025985 -2.6444139 -0.9410142

C 4.3503095 -3.9005959 -0.2841962

C 5.1914795 -4.9696759 -0.1447592

N 4.8403365 -6.0799009 0.6403488

C 3.6287575 -6.0133839 1.3481198

C 2.7852695 -4.9453989 1.2163168

C 5.0829715 2.6042681 -0.8884012

C 6.1475215 3.4278961 -1.0809102

N 6.1205115 4.7759961 -0.7171162

C 4.9159175 5.2682271 -0.2146462

C 3.8226215 4.4778581 -0.0450092

C -7.3192695 -5.6268779 -0.8266202

C -5.5616645 7.2729041 0.6882378

C 7.1339245 5.6898371 -1.2182842

C 5.8747815 -7.0020729 1.0799658

Co -0.0092695 -0.0242679 0.0382838

H 4.6411185 -1.7236629 1.3919208

H 4.9256515 0.9511871 1.1209198

H 1.6295815 4.6815801 -1.3019152

H -1.0230255 4.9439161 -0.8804012

H -4.6813775 1.6568621 1.3918358

H -4.9662465 -1.0125529 1.0742748

H -1.6535885 -4.6882339 -1.4090072

H 1.0024715 -4.9581159 -1.0157992

H -1.8759025 4.8615621 1.8991008

H -3.4236975 6.7806331 2.1313878

H -6.1214655 5.0567381 -0.6050202

H -4.6461735 3.0919631 -0.9072192

H -2.9630305 -4.9745529 0.3361948

H -4.9344645 -6.3653109 -0.0350412

H -7.0908345 -3.1238199 -1.5944902

H -5.2139085 -1.6014759 -1.2158452

H 4.6594885 -3.0847069 -0.9309442

H 6.1465475 -5.0428279 -0.6525512

H 3.4047465 -6.8740919 1.9680708

H 1.8421525 -4.9646919 1.7550858

H 5.1899355 1.5663621 -1.1832252

H 7.0770005 3.0845411 -1.5216722

H 4.9291065 6.3129531 0.0780638

H 2.9485835 4.9258321 0.4130828

H -7.8283715 -5.5158929 0.1430238

H -8.0062015 -5.3098269 -1.6168682

H -7.0860275 -6.6845749 -0.9780852

H -5.1343065 7.8865921 -0.1204552

H -6.6212175 7.1051521 0.4738008

H -5.4840275 7.8314531 1.6256598

H 6.9220765 6.0240641 -2.2454272

H 7.1855955 6.5702731 -0.5709612

H 8.1104975 5.1972491 -1.2077042

H 6.4350445 -6.6155459 1.9458218

H 5.4211295 -7.9577599 1.3588698

H 6.5794615 -7.1807529 0.2625308

[CoP]^–2^ M = 8

C -1.1945261 -2.8185674 -0.2178845

C -2.3780061 -3.4325704 -0.7153985

C -3.3630681 -2.4551704 -0.7559045

C -2.8007401 -1.2537034 -0.2468905

N -1.4462041 -1.4670164 0.0273595

C 0.0354639 -3.4813634 0.0309215

C -3.5096331 -0.0181974 -0.0195215

C -2.8070541 1.2189746 0.2151635

C -3.3790691 2.4190316 0.7172735

C -2.3965901 3.3993616 0.6879785

C -1.2056971 2.7887806 0.2040825

N -1.4501161 1.4364306 -0.0433815

C 0.0247489 3.4554916 -0.0318275

C 1.2571139 2.7888206 -0.2527175

C 2.4491079 3.3960956 -0.7394085

C 3.4347229 2.4192296 -0.7482745

C 2.8635389 1.2240976 -0.2322405

N 1.5046059 1.4410826 0.0160805

C 3.5687629 -0.0069974 0.0218075

C 2.8646509 -1.2409454 0.2674115

C 3.4332399 -2.4345194 0.7893445

C 2.4513659 -3.4149684 0.7663475

C 1.2632029 -2.8111474 0.2662095

N 1.5095939 -1.4621964 0.0019955

C 5.0057479 -0.0041234 0.0336435

C 0.0230279 4.9433096 -0.0455765

C -4.9454151 -0.0199484 -0.0306655

C 0.0383209 -4.9691854 0.0434555

C -0.8265411 5.7048886 -0.9039145

C -0.8354181 7.0727016 -0.9048595

N 0.0370839 7.8106936 -0.0867445

C 0.8471839 7.0891246 0.8070325

C 0.8552979 5.7215236 0.8152515

C 5.8157049 -1.0965514 -0.4646715

C 7.1753919 -1.0749734 -0.4573575

N 7.9005059 -0.0220424 0.1039685

C 7.1643309 1.0712626 0.5663035

C 5.8053499 1.0940766 0.5388685

C -0.8162791 -5.7341374 0.8937295

C -0.8208781 -7.1019854 0.8935295

N 0.0610439 -7.8365804 0.0823975

C 0.8765739 -7.1116724 -0.8037465

C 0.8805049 -5.7440414 -0.8106975

C -5.7523891 -1.1110304 0.4767375

C -7.1119621 -1.0933674 0.4701255

N -7.8408291 -0.0475084 -0.0993705

C -7.1081491 1.0440756 -0.5710465

C -5.7494251 1.0717366 -0.5443565

C 9.3187769 0.1206666 -0.1812355

C -0.2530171 9.2090746 0.1855815

C -9.2590231 0.0938016 0.1869465

C -0.2228481 -9.2354254 -0.1940665

Co 0.0297939 -0.0131814 0.0010425

H -2.4655551 -4.4695864 -1.0140675

H -4.3783841 -2.5762584 -1.1094985

H -4.3982881 2.5365356 1.0606725

H -2.4906901 4.4361386 0.9854275

H 2.5413789 4.4286256 -1.0517435

H 4.4554449 2.5349946 -1.0877055

H 4.4505059 -2.5478194 1.1398155

H 2.5433449 -4.4476314 1.0784055

H -1.5009161 5.1866596 -1.5798905

H -1.4761681 7.6617436 -1.5513045

H 1.4782149 7.6908496 1.4514345

H 1.5316229 5.2160536 1.4989785

H 5.3298889 -1.9566754 -0.9113055

H 7.7719499 -1.8742404 -0.8834475

H 7.7519109 1.8768066 0.9939885

H 5.3091909 1.9564476 0.9695575

H -1.4981001 -5.2185044 1.5642165

H -1.4653521 -7.6935994 1.5339015

H 1.5150849 -7.7108714 -1.4431265

H 1.5611519 -5.2358064 -1.4880715

H -5.2639851 -1.9659954 0.9303285

H -7.7057711 -1.8908484 0.9034015

H -7.6984391 1.8440856 -1.0054025

H -5.2564861 1.9320026 -0.9828315

H 9.5028249 0.5810736 -1.1642085

H 9.7855569 0.7448916 0.5864275

H 9.7980189 -0.8624454 -0.1630155

H -1.0330741 9.3303716 0.9538995

H 0.6549529 9.7123136 0.5309015

H -0.5908761 9.6996626 -0.7320145

H -9.4428031 0.5613006 1.1666265

H -9.7285561 0.7109136 -0.5847905

H -9.7358051 -0.8906304 0.1770125

H -0.9964621 -9.3579634 -0.9686885

H 0.6892789 -9.7355534 -0.5328945

H -0.5664921 -9.7282584 0.7201775

[CoP]^–2^_2 M = 8

C 2.7351166 -1.2116205 -0.6481606

C 3.3338956 -2.4850325 -0.8407586

C 2.4187166 -3.4444515 -0.3814126

C 1.2751316 -2.7543455 0.0725894

N 1.4641476 -1.3960615 -0.1034046

C 3.3281596 0.0569835 -0.9279006

C 0.0217736 -3.3213045 0.5989394

C -1.2381524 -2.7687645 0.0754864

C -2.3876154 -3.4692065 -0.3489136

C -3.3098334 -2.5208625 -0.8157686

C -2.7093654 -1.2431055 -0.6599956

N -1.4313794 -1.4145175 -0.1284796

C -3.3103574 0.0174535 -0.9578926

C -2.7201634 1.2905075 -0.6936026

C -3.3194704 2.5603875 -0.9067466

C -2.4083184 3.5270935 -0.4547126

C -1.2665504 2.8445935 0.0151834

N -1.4526174 1.4838875 -0.1445516

C -0.0178814 3.4194725 0.5437564

C 1.2464876 2.8592895 0.0393354

C 2.3989776 3.5531665 -0.3873656

C 3.3253226 2.5974935 -0.8306756

C 2.7243856 1.3222355 -0.6574456

N 1.4419386 1.5019645 -0.1397746

C -0.0344064 4.4762715 1.4536834

C -4.6819994 0.0020285 -1.5374616

C 0.0307696 -4.3663455 1.5224704

C 4.7030146 0.0637485 -1.5000616

C -4.9310244 -0.3239245 -2.9041946

C -6.1924024 -0.3575015 -3.4328676

N -7.3186844 -0.0203725 -2.6610296

C -7.1081844 0.2466315 -1.2964636

C -5.8493204 0.2764215 -0.7625856

C 1.1649216 5.0781045 2.0345044

C 1.1212176 6.1362525 2.8806594

N -0.0700214 6.7129665 3.2974184

C -1.2440094 6.1236225 2.8500824

C -1.2545424 5.0649035 2.0035844

C 5.8642686 -0.2249445 -0.7215816

C 7.1241176 -0.2223705 -1.2543996

N 7.3493346 0.1186165 -2.5998416

C 6.2192586 0.3567755 -3.4024876

C 4.9571406 0.3499095 -2.8749516

C 1.2464126 -4.9491765 2.0882894

C 1.2289306 -5.9984285 2.9463964

N 0.0512266 -6.5818535 3.3918244

C -1.1364854 -6.0083805 2.9607124

C -1.1731444 -4.9598155 2.1023774

C -0.0853914 8.0083815 3.9592894

C -8.6358714 -0.4469545 -3.1058096

C 0.0606456 -7.8701575 4.0675074

C 8.5986766 -0.2739965 -3.2318566

Co 0.0046626 0.0448655 0.0051074

H 4.3167166 -2.6590995 -1.2610026

H 2.5377596 -4.5216235 -0.4016426

H -2.5053374 -4.5466135 -0.3480146

H -4.2952114 -2.7052235 -1.2256386

H -4.3000124 2.7272675 -1.3352046

H -2.5286334 4.6037275 -0.4898946

H 2.5161026 4.6305335 -0.4035846

H 4.3139426 2.7752865 -1.2355586

H -4.0944374 -0.5595445 -3.5572726

H -6.3965524 -0.6039165 -4.4687966

H -8.0027214 0.4598515 -0.7219566

H -5.7444354 0.5258125 0.2902744

H 2.1352926 4.6544025 1.8082874

H 2.0193786 6.5725095 3.3062854

H -2.1574744 6.5506395 3.2517954

H -2.2142054 4.6315735 1.7511634

H 5.7542406 -0.4604265 0.3339164

H 8.0145376 -0.4414585 -0.6757806

H 6.4276056 0.5789935 -4.4430886

H 4.1239106 0.5796335 -3.5344496

H 2.2082306 -4.5194645 1.8378594

H 2.1391176 -6.4216305 3.3594504

H -2.0380244 -6.4384105 3.3855554

H -2.1414334 -4.5372905 1.8652954

H -0.0809524 8.8399685 3.2405024

H -0.9806684 8.0913565 4.5821474

H 0.7931526 8.1009495 4.6042244

H -8.8231674 -1.5116525 -2.8936336

H -9.4040454 0.1472035 -2.6020716

H -8.7292394 -0.2868865 -4.1840896

H 0.9514166 -7.9473175 4.6975284

H -0.8225154 -7.9550745 4.7071684

H 0.0604406 -8.7093265 3.3575674

H 8.7598676 0.3270555 -4.1317076

H 9.4306726 -0.0942735 -2.5444856

H 8.6052206 -1.3380665 -3.5171926
