# Supplementary material for: Self-Assembled Liposomes Enhance Electron Transfer for Efficient Photocatalytic CO2 Reduction
Source: J Am Chem Soc. 2022 May 20;144(21):9399–412. doi: 10.1021/jacs.2c01725 (PMC9164230; doi:10.1021/jacs.2c01725)
Supplement: Supplementary file 2 — ja2c01725_si_002.zip [file ja2c01725_si_002.zip › Coordinates/CoP_CO/Coord_CoP_CO.docx]

[CoP(CO)]^+4^ M = 2

C -1.17091050 2.81740986 -0.11330891

C -0.79172341 4.21130986 -0.12923412

C 0.56400447 4.24858367 -0.15242369

C 1.01982825 2.87799581 -0.12411599

N -0.05237559 2.01393425 -0.10870649

C -2.49394463 2.36757380 -0.07932314

C 2.36636261 2.50328802 -0.14155617

C 2.81531708 1.17948638 -0.14421498

C 4.20950223 0.80064631 -0.14284758

C 4.24748505 -0.55499136 -0.11430450

C 2.87682311 -1.01024796 -0.08157668

N 2.01240114 0.06142313 -0.10589318

C 2.50328240 -2.35727062 -0.07615073

C 1.18038000 -2.80723893 -0.11182154

C 0.80120590 -4.20088532 -0.12948817

C -0.55459624 -4.23837365 -0.15514986

C -1.01080915 -2.86820114 -0.12651493

N 0.06149233 -2.00386460 -0.10857698

C -2.35726522 -2.49349951 -0.14616598

C -2.80613524 -1.16974401 -0.14842971

C -4.19995864 -0.79064288 -0.14991194

C -4.23787319 0.56509713 -0.12106081

C -2.86753125 1.02050695 -0.08506375

N -2.00310311 -0.05145161 -0.10783006

Co 0.00489119 0.00538549 0.02499721

H -1.46947455 5.05165942 -0.12519125

H 1.19421818 5.12436320 -0.19238954

H 5.04967522 1.47852799 -0.15900506

H 5.12358390 -1.18597104 -0.12635223

H 1.47882098 -5.04136910 -0.12501069

H -1.18452786 -5.11425707 -0.19715360

H -5.04021779 -1.46835128 -0.16841853

H -5.11418888 1.19573680 -0.13550326

H -5.82591826 -4.95041969 -2.11382188

H -4.13763452 -3.11847941 -2.15159570

C -5.16087887 -4.76861934 -1.27900905

C -4.22006596 -3.75808263 -1.28133551

H -7.30726412 -6.18596565 -0.31241610

H -6.15190266 -7.28345608 -1.12814087

C -6.32453877 -6.65601363 -0.25288598

N -5.29000873 -5.59523932 -0.21342527

C -3.38025232 -3.57585117 -0.17006496

H -6.25476179 -7.25652589 0.65199602

C -4.49537173 -5.44664037 0.87029875

C -3.53764593 -4.44951854 0.91538622

H -4.65368775 -6.13629495 1.68875850

H -2.92179699 -4.35356634 1.80123440

H -6.10588516 4.59299028 1.90089971

H -4.30101154 2.87375752 1.92834151

C -5.43265331 4.45638072 1.06403651

C -4.42714979 3.51030228 1.06108224

H -7.66028430 5.75025796 0.18728629

H -6.53222991 6.95041117 0.88810692

C -6.71303416 6.27379022 0.05178048

N -5.61901080 5.27505894 0.00069044

C -3.58027125 3.38622285 -0.05287779

H -6.72977813 6.83359619 -0.88131633

C -4.82068582 5.18030168 -1.08627487

C -3.79961473 4.24834708 -1.13676300

H -5.02700008 5.85878883 -1.90337331

H -3.18454844 4.19270867 -2.02656359

H 5.03247469 -5.85739487 -1.89404347

H 3.19599803 -4.17928637 -2.02570213

C 4.82175677 -5.17580461 -1.07971378

C 3.80825905 -4.23980454 -1.13426434

H 6.24880780 -7.27819987 -0.15355561

H 7.40748900 -6.05957579 -0.76757561

C 6.69216339 -6.29714031 0.02132308

N 5.61579098 -5.27815901 0.01321485

C 3.58838124 -3.37724585 -0.04756932

H 7.18883957 -6.28338488 0.98949269

C 5.43190061 -4.45980873 1.07374355

C 4.43006021 -3.50593234 1.06652529

H 6.09714485 -4.59583520 1.91617396

H 4.30483978 -2.87035084 1.93460550

H 5.83933602 4.96252867 -2.09884289

H 4.15091217 3.13092365 -2.14269714

C 5.17255002 4.77969232 -1.26565812

C 4.23158840 3.76930733 -1.27137884

H 7.31706814 6.19559325 -0.29401908

H 6.16277618 7.29494573 -1.10875172

C 6.33430856 6.66559563 -0.23463958

N 5.29962801 5.60483966 -0.19868504

C 3.38942034 3.58565886 -0.16211928

H 6.26355899 7.26415859 0.67145858

C 4.50269522 5.45487212 0.88316512

C 3.54471069 4.45785877 0.92483387

H 4.65938174 6.14332523 1.70295419

H 2.92700974 4.36080749 1.80926992

C -0.02958409 -0.03135419 2.13800252

O -0.11496372 -0.12436803 3.26740161

[CoP(CO)]^+4^ M = 4

C -2.57227044 -1.56207602 -0.18787272

C -3.98474468 -1.44790081 0.09856859

C -4.22073035 -0.14249620 0.40829812

C -2.96058619 0.54239066 0.29962904

N -1.95405002 -0.37325408 -0.04352102

C -1.91845055 -2.81370897 -0.50368869

C -2.78902851 1.91385475 0.33974298

C -1.57985308 2.58395171 0.03833119

C -1.47376838 3.99645936 -0.21837775

C -0.17586875 4.23815755 -0.54521841

C 0.53637981 2.98393148 -0.40126617

N -0.35825832 1.97438092 -0.08824354

C 1.91808241 2.81929715 -0.50854667

C 2.57268325 1.56757624 -0.19594081

C 3.98676967 1.45363110 0.08589587

C 4.22322037 0.14921865 0.39779385

C 2.96226760 -0.53565380 0.29347047

N 1.95473897 0.37983449 -0.04802222

C 2.79117943 -1.90666477 0.33475363

C 1.58174900 -2.57726241 0.03379961

C 1.47637331 -3.98834366 -0.22745064

C 0.17776498 -4.23008487 -0.55257266

C -0.53586051 -2.97756895 -0.40205205

N 0.35918197 -1.96819389 -0.08894946

Co 0.00081120 0.00362408 0.04482543

H -4.69912903 -2.25721293 0.08158622

H -5.16749659 0.31554871 0.65568797

H -2.28372859 4.71009941 -0.18764241

H 0.25131038 5.18069197 -0.85037780

H 4.70122356 2.26279953 0.06496192

H 5.17049878 -0.30878766 0.64331740

H 2.28705670 -4.70132742 -0.20167372

H -0.24820223 -5.17185578 -0.86153469

H 6.34124717 -4.78374634 -0.71356813

H 4.33767019 -3.45037857 -1.36075222

C 5.77980882 -4.20847270 0.01139230

C 4.66579475 -3.46491687 -0.32855514

H 7.37065553 -6.00484219 1.13848335

H 8.31758450 -4.48728120 1.20576215

C 7.45141535 -5.02110652 1.60078175

N 6.22925899 -4.24142080 1.28832288

C 3.98776866 -2.73577123 0.65940197

H 7.53803765 -5.12733836 2.68034924

C 5.59637126 -3.54600443 2.25976700

C 4.47577385 -2.78671916 1.97085740

H 6.00916250 -3.61648888 3.25748813

H 3.98752459 -2.24263915 2.77009073

H -3.53273767 -7.19953059 -0.15898207

H -2.04190671 -5.37305050 0.58553963

C -3.54755273 -6.22861808 -0.63709587

C -2.71640227 -5.20126330 -0.24355139

H -4.88653115 -8.11357077 -1.76703930

H -6.29436739 -7.05316774 -1.46927513

C -5.35541460 -7.16267041 -2.01645548

N -4.44423379 -6.06080523 -1.64003664

C -2.78054797 -3.94262379 -0.87876732

H -5.54124442 -7.11851601 -3.08940791

C -4.54840338 -4.85751402 -2.26287146

C -3.74714685 -3.79878320 -1.89971231

H -5.28080006 -4.78735579 -3.05684296

H -3.85602173 -2.86053168 -2.43012826

H 5.27579822 4.80033723 -3.06357041

H 3.83950878 2.87523781 -2.45047821

C 4.54872478 4.86464534 -2.26397643

C 3.73953269 3.80925107 -1.91086135

H 6.37244717 6.80624583 -2.09176024

H 5.02499027 7.53005790 -3.01984554

C 5.34512016 7.16929765 -2.04024603

N 4.46270050 6.05940065 -1.62104739

C 2.78034276 3.94916156 -0.88198094

H 5.27700525 7.97376405 -1.30985553

C 3.56766866 6.22548218 -0.61750220

C 2.72702810 5.20091839 -0.23420805

H 3.56201434 7.19042236 -0.12765482

H 2.05807271 5.36828424 0.60028357

H -6.33658018 4.79112258 -0.71515821

H -4.33212015 3.45702406 -1.35829498

C -5.77642825 4.21609598 0.01100265

C -4.66207531 3.47206243 -0.32668713

H -7.36645088 6.01505721 1.13600197

H -8.31564147 4.49875639 1.19966955

C -7.44972130 5.03104689 1.59729203

N -6.22784012 4.25000185 1.28722314

C -3.98585632 2.74317138 0.66275668

H -7.53892400 5.13651788 2.67672320

C -5.59681339 3.55480660 2.26003896

C -4.47613311 2.79480566 1.97332658

H -6.01106941 3.62610468 3.25708576

H -3.98943114 2.25094227 2.77364525

C -0.02356972 -0.05173937 2.17625393

O -0.08791645 -0.17958629 3.30315813

[CoP(CO)]^+2^ M = 2

C 2.65034068 -1.22164426 -0.74086342

C 3.24759875 -2.51221726 -0.88262044

C 2.38293877 -3.41877552 -0.30945813

C 1.23811179 -2.68618735 0.14499783

N 1.39800869 -1.36954754 -0.14351333

C 3.21112146 0.03149484 -1.04389219

C 0.02072221 -3.23477974 0.72536391

C -1.22367247 -2.69697793 0.20044003

C -2.40870181 -3.42917710 -0.14467463

C -3.28687446 -2.53843299 -0.71950087

C -2.64796488 -1.25952439 -0.71104735

N -1.37792080 -1.39549921 -0.15448632

C -3.23107362 -0.02036363 -1.02921605

C -2.66502526 1.22978111 -0.72977912

C -3.26364796 2.52242975 -0.86599981

C -2.39963222 3.42533299 -0.28942936

C -1.25287539 2.68874670 0.15991031

N -1.41198597 1.37546668 -0.13567117

C -0.03265715 3.23659360 0.73464335

C 1.20833974 2.69924843 0.20152817

C 2.39154950 3.43533026 -0.14959504

C 3.26407123 2.54842812 -0.73537812

C 2.62393127 1.26779902 -0.72738691

N 1.35838733 1.40163486 -0.16253713

C -0.05944997 4.25484404 1.70579817

C -4.57177705 -0.05217611 -1.66168109

C 0.05297297 -4.25156053 1.69735343

C 4.55397088 0.06418725 -1.67180136

C -4.76575941 -0.72241802 -2.88311329

C -6.01289898 -0.74719722 -3.47238721

N -7.07093041 -0.13399399 -2.88713974

C -6.92107115 0.50927087 -1.70629412

C -5.69092972 0.56332265 -1.07823691

C 1.12652324 4.79128706 2.33574146

C 1.07035513 5.79852159 3.25173095

N -0.11675409 6.34677765 3.66503940

C -1.27776679 5.80566233 3.17524402

C -1.28036688 4.79757222 2.25858266

C 5.67031655 -0.55007568 -1.08150895

C 6.90417846 -0.49497297 -1.70188666

N 7.06065293 0.14887600 -2.88150245

C 6.00512133 0.75858478 -3.47478770

C 4.75437746 0.73280815 -2.89318989

C 1.27702197 -4.79342180 2.24456080

C 1.27948579 -5.79816841 3.16473601

N 0.12125922 -6.33755655 3.66321586

C -1.06828657 -5.79110398 3.25395167

C -1.12965194 -4.78728940 2.33471446

C -0.14172933 7.51294206 4.55121143

C -8.39682929 -0.20627524 -3.54313855

C 0.15076004 -7.50052042 4.55327169

C 8.37934106 0.18037243 -3.55484239

Co -0.00848281 0.00372541 -0.20891593

H 4.19732566 -2.73026234 -1.35047953

H 2.50393326 -4.49165649 -0.25501020

H -2.55055034 -4.49297692 -0.01732664

H -4.28262472 -2.74992425 -1.08443857

H -4.21378196 2.74198728 -1.33227328

H -2.51929412 4.49814030 -0.23089220

H 2.53303186 4.49884687 -0.01972623

H 4.25682472 2.76131255 -1.10754837

H -3.93789757 -1.21502305 -3.37786810

H -6.20505382 -1.24270087 -4.41552830

H -7.80779088 0.96432169 -1.28473416

H -5.61027957 1.07459285 -0.12739542

H 2.10128285 4.37960042 2.11330346

H 1.96328000 6.20522383 3.71199367

H -2.19630177 6.21955031 3.57452174

H -2.24116131 4.39394418 1.97006498

H 5.58491807 -1.05880491 -0.12973466

H 7.78877352 -0.94936242 -1.27551791

H 6.20256436 1.25082344 -4.41860315

H 3.92953940 1.22589366 -3.39243877

H 2.23623805 -4.39109824 1.94899694

H 2.20022044 -6.21061276 3.56035198

H -1.95860819 -6.19644562 3.72042289

H -2.10577506 -4.37704570 2.11546425

H -0.11081730 8.44043785 3.96995634

H -1.05410107 7.49405593 5.15020124

H 0.71992299 7.47903006 5.22057706

H -8.77979954 -1.22455919 -3.45580874

H -9.07505811 0.48971690 -3.05284234

H -8.28667876 0.06560560 -4.59332984

H 1.07130576 -7.48513879 5.13974588

H -0.70150713 -7.45887740 5.23425832

H 0.10632677 -8.43016958 3.97627371

H 8.61782751 1.21130407 -3.81841107

H 9.13562699 -0.21037120 -2.87640243

H 8.33147195 -0.43678519 -4.45369132

C -0.02067563 -0.24984999 -2.36682033

O -0.03885936 -0.85984650 -3.32977093

[CoP(CO)]^+2^ M = 4

C 2.69755272 -1.26051728 -0.65756535

C 3.22652843 -2.57549399 -0.83463209

C 2.33679243 -3.45717238 -0.25153627

C 1.23264764 -2.68991885 0.21872869

N 1.44738987 -1.36539404 -0.06208776

C 3.30357851 -0.00583429 -0.98234818

C -0.00627133 -3.21243785 0.76125014

C -1.23108395 -2.65788872 0.24873401

C -2.44507345 -3.37041559 -0.06103464

C -3.27839068 -2.49111198 -0.70371018

C -2.59654985 -1.22914427 -0.75453556

N -1.34127490 -1.37288403 -0.18628479

C -3.15304276 0.00673714 -1.13752897

C -2.57117600 1.25464810 -0.84410764

C -3.14756169 2.55689869 -1.01622526

C -2.31018124 3.44778432 -0.39025402

C -1.19043029 2.69700777 0.11149026

N -1.34611442 1.38572228 -0.20793521

C -0.00406919 3.21667614 0.75270875

C 1.26299423 2.66106452 0.34369447

C 2.50471781 3.35390018 0.19698822

C 3.39508906 2.47313926 -0.37977030

C 2.70421911 1.23657759 -0.57341643

N 1.40755023 1.38159641 -0.13815754

C -0.09706095 4.24538435 1.74442123

C -4.47799949 -0.02533285 -1.79724694

C -0.00478705 -4.26553353 1.72762275

C 4.58653522 0.05523662 -1.64404464

C -4.66547644 -0.76086714 -2.98304708

C -5.90046575 -0.79132855 -3.59597121

N -6.95603616 -0.12267477 -3.06862710

C -6.81308785 0.58566390 -1.92434645

C -5.59470683 0.64970302 -1.27592958

C 1.03050782 4.68869592 2.51451815

C 0.92629893 5.67937174 3.45175284

N -0.26472966 6.28418332 3.73270782

C -1.38681187 5.83122429 3.09906468

C -1.33659424 4.83899329 2.15927183

C 5.67845894 -0.82571261 -1.36816127

C 6.87379684 -0.71626062 -2.02601331

N 7.08013055 0.23185413 -2.99108508

C 6.06262305 1.10494398 -3.28626457

C 4.86111528 1.04878751 -2.63584798

C 1.19964151 -4.85274947 2.24258552

C 1.17644513 -5.86574226 3.16102326

N 0.00710912 -6.35223186 3.67083137

C -1.16334805 -5.76048390 3.28852319

C -1.19470332 -4.74571423 2.37269553

C -0.33852872 7.41366443 4.66945906

C -8.27034526 -0.20646655 -3.74522739

C -0.00006051 -7.50403055 4.58289520

C 8.33620114 0.28376850 -3.74824375

Co 0.04795894 0.00447642 -0.23118115

H 4.13871152 -2.84091365 -1.34607391

H 2.41359269 -4.53542790 -0.22933100

H -2.62402162 -4.42172909 0.11059265

H -4.27191786 -2.69322427 -1.07873727

H -4.06464537 2.78851827 -1.53872048

H -2.41877901 4.52223818 -0.35272636

H 2.68477069 4.39469481 0.42439495

H 4.43252814 2.66436061 -0.61392289

H -3.84055164 -1.29907347 -3.43337611

H -6.08558200 -1.33489420 -4.51382810

H -7.69737058 1.08198313 -1.54665108

H -5.52356009 1.21014047 -0.35243946

H 1.99665076 4.22011475 2.39957936

H 1.78040052 6.01952203 4.02430380

H -2.32013273 6.29380286 3.39578799

H -2.27971125 4.49983213 1.75741926

H 5.60703821 -1.55651405 -0.57396715

H 7.71836480 -1.35457054 -1.79745785

H 6.26677815 1.82300370 -4.07079704

H 4.09146678 1.75588512 -2.92055215

H 2.16861921 -4.49059047 1.93127049

H 2.08489617 -6.32126190 3.53541307

H -2.06208110 -6.13106725 3.76636820

H -2.15471881 -4.28941109 2.17991189

H -0.26574552 8.35846694 4.12309575

H -1.28682219 7.37528258 5.20774349

H 0.48175362 7.34241536 5.38460871

H -8.66647862 -1.21630564 -3.62454939

H -8.94964835 0.51570348 -3.29580539

H -8.13835306 0.02162040 -4.80330175

H 0.95788074 -7.55850131 5.10112724

H -0.79713713 -7.37929480 5.31757216

H -0.16320900 -8.42710453 4.01916409

H 8.62672444 1.32527675 -3.89936925

H 9.11800376 -0.22547725 -3.18385391

H 8.21278720 -0.20525834 -4.71947155

C 0.12585761 -0.27885097 -2.34997483

O 0.20308027 -0.87186251 -3.32084546

[CoP(CO)]^+1^ M = 1

C 2.61184507 -1.19999230 -0.71132176

C 3.19512743 -2.49396375 -0.88005098

C 2.36831167 -3.39281912 -0.23761369

C 1.25493500 -2.65403563 0.27596546

N 1.39791514 -1.34173056 -0.04162029

C 3.15921675 0.05913452 -1.03301122

C 0.07809297 -3.17653397 0.95520950

C -1.20812008 -2.66588445 0.48611738

C -2.41412997 -3.39163293 0.29584313

C -3.32776690 -2.52298989 -0.28566533

C -2.67685935 -1.27097114 -0.44898359

N -1.37968429 -1.38617301 0.03202973

C -3.28230940 -0.04130765 -0.87138191

C -2.69791051 1.22839478 -0.56356565

C -3.22014849 2.53019104 -0.79590401

C -2.33686506 3.43673150 -0.22102369

C -1.25943918 2.68731673 0.31173582

N -1.46670299 1.35796309 0.08023822

C -0.02938819 3.22266972 0.90533520

C 1.20787973 2.69969614 0.35975277

C 2.39854239 3.43277461 0.02628305

C 3.24359557 2.56198639 -0.62165250

C 2.58485345 1.28966249 -0.65838266

N 1.33575785 1.41607638 -0.06225178

C -0.05847390 4.23144059 1.87771235

C -4.54771591 -0.12249933 -1.56812321

C 0.18926397 -4.13668870 1.97118572

C 4.45976325 0.10961962 -1.73405542

C -4.78421624 -1.11982026 -2.56749637

C -5.95967068 -1.18174791 -3.26175616

N -6.98986863 -0.30318077 -3.01262482

C -6.82576239 0.63806207 -2.02864416

C -5.65498955 0.75259410 -1.33048672

C 1.12230792 4.75785704 2.53461617

C 1.05773400 5.76395985 3.44858746

N -0.13399793 6.32575544 3.84106679

C -1.29249970 5.79764202 3.32126609

C -1.28690045 4.79292382 2.40396452

C 5.60043471 -0.57036287 -1.26972471

C 6.79153653 -0.49347185 -1.96413568

N 6.89082377 0.23617601 -3.10061089

C 5.81319848 0.91248458 -3.57317516

C 4.60383600 0.86757645 -2.91357838

C 1.45349093 -4.66576415 2.44694664

C 1.52530455 -5.62725099 3.40689979

N 0.40710648 -6.13333322 4.02729338

C -0.81032846 -5.59075189 3.69027050

C -0.94096364 -4.63374668 2.73212588

C -0.16406684 7.50195231 4.70948376

C -8.25768193 -0.43927685 -3.73544016

C 0.50224032 -7.26612608 4.94668416

C 8.16259460 0.29097899 -3.85533248

Co -0.03487545 0.01379560 -0.08329393

H 4.10981279 -2.72245481 -1.40885347

H 2.49379267 -4.46498484 -0.17342297

H -2.56712656 -4.43792046 0.52196891

H -4.35669516 -2.74071874 -0.53699204

H -4.12545591 2.77736369 -1.32981859

H -2.41920623 4.51575629 -0.21514720

H 2.55970374 4.48714452 0.20128819

H 4.23158138 2.77801874 -1.00497092

H -3.99912761 -1.82042527 -2.82470312

H -6.13338396 -1.90239077 -4.05157313

H -7.68175791 1.27088466 -1.82904880

H -5.60822608 1.49069092 -0.54085744

H 2.09634418 4.33273837 2.33322714

H 1.94461283 6.16012785 3.92996052

H -2.21410291 6.22542636 3.69902936

H -2.24299536 4.40696153 2.07610930

H 5.56826854 -1.14301804 -0.35182835

H 7.69015809 -0.99652570 -1.63194376

H 5.96025798 1.47172070 -4.48848690

H 3.76203436 1.41366014 -3.32068489

H 2.38767742 -4.28603492 2.05567135

H 2.47291576 -6.03063678 3.74531350

H -1.66105426 -5.96245589 4.24998977

H -1.92786860 -4.22288449 2.56928992

H -0.11639935 8.42519367 4.12116596

H -1.08601249 7.49887933 5.29492846

H 0.68613248 7.47083635 5.39425010

H -8.82708033 -1.29118468 -3.35001154

H -8.84327580 0.47211852 -3.61156993

H -8.05749862 -0.59013428 -4.79888472

H 1.45150854 -7.21999107 5.48503906

H -0.31410671 -7.21652523 5.67043758

H 0.44326188 -8.21729237 4.40563873

H 8.41062763 1.33353720 -4.05825654

H 8.95123145 -0.16423222 -3.25854668

H 8.04316713 -0.25594956 -4.79256832

C -0.13949100 -0.21933446 -2.17959946

O -0.28227868 -0.81420324 -3.14405334

[CoP(CO)]^+1^ M = 3

C 2.62086481 -1.19998275 -0.73876638

C 3.20403744 -2.49396940 -0.90583389

C 2.37269860 -3.39342387 -0.26958680

C 1.25725829 -2.65493715 0.23863661

N 1.40213056 -1.34203376 -0.07823809

C 3.17339380 0.05966863 -1.05232207

C 0.07982611 -3.17638005 0.91630478

C -1.20556680 -2.66519699 0.44829652

C -2.41373626 -3.39046098 0.26599143

C -3.32886456 -2.52336981 -0.31364442

C -2.67762292 -1.27188288 -0.48428900

N -1.37729087 -1.38723390 -0.01028567

C -3.28643422 -0.04229420 -0.89898611

C -2.69672027 1.22668411 -0.60063971

C -3.21966517 2.52808133 -0.83387176

C -2.33543886 3.43532698 -0.26212838

C -1.25683788 2.68643040 0.26996726

N -1.46347665 1.35716796 0.03884152

C -0.02908683 3.22137086 0.86730987

C 1.20995472 2.69837436 0.32868627

C 2.40500766 3.43066878 0.01002323

C 3.25501522 2.56132838 -0.63292327

C 2.59458370 1.29013506 -0.68252187

N 1.33960782 1.41608734 -0.09905095

C -0.06262439 4.22564879 1.84614355

C -4.56633749 -0.12039513 -1.56966424

C 0.19046703 -4.13169721 1.93863892

C 4.48577250 0.11032919 -1.72835036

C -4.82870993 -1.11698784 -2.56356978

C -6.02251500 -1.17994890 -3.22547773

N -7.04649926 -0.30169007 -2.94885125

C -6.85616963 0.63978772 -1.96857153

C -5.66699053 0.75491559 -1.30301251

C 1.11456017 4.74304443 2.51489205

C 1.04608026 5.73890331 3.44037811

N -0.14655537 6.29837722 3.83081957

C -1.30211864 5.78012811 3.29704542

C -1.29269278 4.78554298 2.36806050

C 5.61474303 -0.58014132 -1.24897824

C 6.81945631 -0.50274703 -1.91867134

N 6.94512145 0.23721898 -3.04639652

C 5.88010480 0.92491567 -3.53218217

C 4.65827819 0.88023021 -2.89702238

C 1.45419848 -4.65820554 2.41644481

C 1.52655407 -5.60879911 3.38771151

N 0.40931138 -6.10586725 4.01540516

C -0.80764266 -5.56697528 3.67399788

C -0.93893251 -4.62067983 2.70468269

C -0.18003382 7.45862269 4.72143643

C -8.33720230 -0.44365362 -3.62754883

C 0.50687283 -7.22181101 4.95597821

C 8.23022970 0.29095900 -3.77718797

Co -0.03135681 0.01308641 -0.12698961

H 4.12198158 -2.72246787 -1.42895821

H 2.49767452 -4.46565650 -0.20522722

H -2.56680890 -4.43555972 0.49728674

H -4.35980107 -2.74072965 -0.55659432

H -4.12726244 2.77355763 -1.36491852

H -2.41831874 4.51429683 -0.25614469

H 2.56651098 4.48383243 0.19181248

H 4.24756284 2.77768369 -1.00382911

H -4.05051540 -1.81760784 -2.84109404

H -6.21750890 -1.90150152 -4.00950022

H -7.70735579 1.27091411 -1.74461915

H -5.59878689 1.49299973 -0.51485478

H 2.08904128 4.31789564 2.31615135

H 1.93054389 6.12694821 3.93251478

H -2.22526573 6.20600214 3.67291440

H -2.24776245 4.40617111 2.02997444

H 5.56221051 -1.16051806 -0.33690929

H 7.70891393 -1.01290687 -1.57295323

H 6.04805507 1.49260026 -4.43862343

H 3.82756676 1.43529682 -3.31468720

H 2.38837442 -4.28446290 2.01965163

H 2.47426383 -6.00932961 3.72904640

H -1.65798268 -5.93117387 4.23905603

H -1.92593013 -4.21115281 2.53965558

H -0.11921994 8.39168210 4.15036811

H -1.10919704 7.45026916 5.29501873

H 0.66128392 7.40902736 5.41585288

H -8.90530012 -1.27629382 -3.19985112

H -8.90835456 0.47839687 -3.51394152

H -8.17175480 -0.62872209 -4.69155864

H 1.45277751 -7.15969811 5.49843030

H -0.31367363 -7.16455992 5.67410486

H 0.45686984 -8.18210366 4.43073399

H 8.49017803 1.33380731 -3.96347379

H 9.00486867 -0.17736999 -3.17218325

H 8.12411697 -0.24407272 -4.72297686

C -0.13067816 -0.22009056 -2.21347380

O -0.26947770 -0.81442614 -3.17938373

[CoP(CO)]^+1^ M = 5

C 2.61576052 -1.27254697 -0.74292432

C 3.10838873 -2.59323960 -0.96593231

C 2.23952181 -3.46678007 -0.33344194

C 1.18397256 -2.68560115 0.21300135

N 1.40331165 -1.36507477 -0.07165589

C 3.22038276 -0.01557785 -1.07005095

C -0.02072937 -3.17371746 0.86253891

C -1.27536418 -2.61902829 0.41400352

C -2.52637773 -3.29171665 0.26259242

C -3.38493159 -2.41654922 -0.37312603

C -2.66703795 -1.20231382 -0.59522492

N -1.38547634 -1.35553809 -0.11453453

C -3.22698243 0.03470243 -1.07291976

C -2.61846322 1.28973348 -0.74529863

C -3.11251863 2.61190046 -0.96360454

C -2.24665229 3.48235280 -0.32417237

C -1.19191409 2.69734185 0.22121496

N -1.40846249 1.37961513 -0.07295098

C 0.01206280 3.18299539 0.87427205

C 1.26676758 2.62925136 0.42458322

C 2.51734086 3.30529377 0.27443788

C 3.37444527 2.43440279 -0.36723922

C 2.65546301 1.21984286 -0.59347071

N 1.37544920 1.37000774 -0.11193476

C -0.05911232 4.18996778 1.87694948

C -4.46958665 -0.03298133 -1.80310547

C 0.05017833 -4.18292784 1.86302746

C 4.46472504 0.05250570 -1.79691952

C -4.70976629 -1.06955933 -2.76213152

C -5.86567205 -1.12829305 -3.48813117

N -6.87305650 -0.20867657 -3.31310853

C -6.70808775 0.77258033 -2.37183092

C -5.55595628 0.88553479 -1.64264988

C 1.07828620 4.61852052 2.65078077

C 0.98710112 5.59998318 3.59484842

N -0.19979216 6.21907934 3.88691277

C -1.33114043 5.77985101 3.24947875

C -1.29404648 4.79830014 2.30205276

C 5.55050714 -0.86690484 -1.63248448

C 6.70435392 -0.75602415 -2.35816322

N 6.87862962 0.23321638 -3.29073312

C 5.86953149 1.14776248 -3.47493954

C 4.71049638 1.08995367 -2.75303165

C 1.28545315 -4.78965387 2.28911963

C 1.32292801 -5.77172654 3.23605146

N 0.19154381 -6.21345424 3.87139736

C -0.99592046 -5.59638713 3.57809080

C -1.08755294 -4.61431818 2.63461368

C -0.25434432 7.34962725 4.81856465

C -8.11980980 -0.33901896 -4.07428693

C 0.24650033 -7.34508793 4.80188081

C 8.07562618 0.26817104 -4.13681775

Co -0.00387309 0.00764377 -0.19759776

H 3.98361131 -2.87038906 -1.53306853

H 2.30261086 -4.54637450 -0.31532554

H -2.73422829 -4.31802870 0.52932031

H -4.41975730 -2.59680291 -0.62827073

H -3.98671171 2.89069146 -1.53156090

H -2.30907872 4.56190491 -0.30192637

H 2.72399373 4.33108282 0.54423059

H 4.40846248 2.61656308 -0.62439607

H -3.94183950 -1.80664847 -2.96339931

H -6.03930552 -1.88159081 -4.24689130

H -7.54916421 1.43952275 -2.22713345

H -5.51658113 1.65412019 -0.88291526

H 2.03841596 4.13801930 2.52927788

H 1.84493835 5.92440573 4.17156780

H -2.25839877 6.24971167 3.55480103

H -2.23824208 4.47421500 1.88793573

H 5.50926434 -1.63214847 -0.86935634

H 7.54368214 -1.42561765 -2.21442651

H 6.04469033 1.90084940 -4.23322265

H 3.94693354 1.83177404 -2.95334026

H 2.22975192 -4.46359955 1.87679885

H 2.25060472 -6.23995739 3.54253608

H -1.85403326 -5.92259044 4.15340720

H -2.04835692 -4.13539793 2.51249935

H -0.14913548 8.29562671 4.27786945

H -1.20976461 7.33914531 5.34641614

H 0.55372082 7.25798774 5.54614222

H -8.70572181 -1.18427107 -3.70013349

H -8.70177338 0.57701999 -3.97228883

H -7.88872607 -0.49769096 -5.13042975

H 1.20214871 -7.33523451 5.32924818

H -0.56126403 -7.25432134 5.52988948

H 0.14107146 -8.29033459 4.25999448

H 8.33032969 1.30519070 -4.36249509

H 8.90815002 -0.19362352 -3.60383484

H 7.89836006 -0.27409767 -5.07123876

C -0.01655774 -0.24212232 -2.27688993

O -0.05765955 -0.84036848 -3.24929060

[CoP(CO)]^0^ M = 2

C 2.66967606 -1.16318637 -0.80402634

C 3.19160210 -2.45972968 -1.05543992

C 2.34367437 -3.37327449 -0.43235702

C 1.28898805 -2.63254371 0.15012730

N 1.47221046 -1.30310564 -0.09993383

C 3.22605459 0.11817115 -1.12557990

C 0.10799220 -3.15122896 0.85081844

C -1.17510922 -2.67384069 0.34730210

C -2.37739366 -3.40926675 0.15946532

C -3.27914665 -2.56440021 -0.47250370

C -2.62691757 -1.31502034 -0.66478957

N -1.34051239 -1.41175234 -0.15214966

C -3.23302284 -0.09926538 -1.12686866

C -2.67299596 1.17986859 -0.80891094

C -3.19855416 2.47842980 -1.05490660

C -2.35510330 3.38887885 -0.42503778

C -1.29812204 2.64485486 0.15428632

N -1.47646868 1.31871847 -0.10651240

C -0.11618738 3.16414790 0.85230528

C 1.16600165 2.68578578 0.34783217

C 2.36793257 3.42509489 0.15860374

C 3.26697618 2.58401847 -0.47845276

C 2.61370138 1.33260639 -0.67167965

N 1.32897293 1.42749817 -0.15798256

C -0.23093792 4.09368332 1.88555069

C -4.47847482 -0.20562455 -1.85317060

C 0.22433663 -4.07691747 1.88639840

C 4.47663239 0.22180935 -1.84293552

C -4.68928904 -1.23513144 -2.82776635

C -5.84843964 -1.32807837 -3.54378873

N -6.89153035 -0.44798635 -3.34629386

C -6.75377048 0.52372875 -2.38398605

C -5.59897743 0.66866817 -1.66746337

C 0.89500870 4.58786622 2.66342498

C 0.75140323 5.51813726 3.64299263

N -0.47488219 6.04221790 3.99222781

C -1.58761500 5.54628573 3.34515852

C -1.50381267 4.61301050 2.36172342

C 5.59474667 -0.65339433 -1.64434931

C 6.75647574 -0.51185929 -2.34949769

N 6.91192197 0.46982754 -3.29965987

C 5.86559604 1.33903843 -3.52161415

C 4.69847834 1.24851494 -2.81779025

C 1.49830067 -4.59422216 2.36256501

C 1.58407289 -5.52155373 3.35121875

N 0.47267546 -6.01401653 4.00356507

C -0.75469373 -5.49359403 3.65217393

C -0.90053811 -4.56930540 2.66742573

C -0.58069353 7.16225028 4.92260258

C -8.15199928 -0.62866106 -4.06849794

C 0.58072754 -7.12998011 4.93849566

C 8.11550896 0.53019372 -4.13038772

Co -0.00419269 0.00712868 -0.23441133

H 4.07527999 -2.70096706 -1.62758543

H 2.44383373 -4.45098932 -0.41110275

H -2.53288329 -4.44601171 0.42553642

H -4.30272699 -2.79189921 -0.73758029

H -4.08190055 2.72063417 -1.62711180

H -2.45507790 4.46648040 -0.40020974

H 2.52221109 4.46206338 0.42450002

H 4.28931768 2.81332343 -0.74669396

H -3.89482453 -1.93879107 -3.04538471

H -6.00045639 -2.07593330 -4.31254545

H -7.61936190 1.15392630 -2.21927249

H -5.57483766 1.42971739 -0.89891583

H 1.88608379 4.18746513 2.49560770

H 1.59555721 5.88175582 4.21846521

H -2.53926097 5.93907037 3.68583737

H -2.42924590 4.24659728 1.93608229

H 5.56304082 -1.40979951 -0.87137482

H 7.61875838 -1.14503414 -2.17836087

H 6.02435891 2.08281361 -4.29276816

H 3.90887674 1.95520683 -3.04310474

H 2.42267903 -4.23034619 1.93238754

H 2.53641115 -5.91236534 3.69227806

H -1.59792193 -5.85497040 4.23045217

H -1.89243405 -4.17162500 2.49772870

H -0.51980584 8.12379549 4.39855759

H -1.53424993 7.10779303 5.45338026

H 0.22868336 7.10624399 5.65433158

H -8.75052025 -1.42235540 -3.60827420

H -8.71688981 0.30427663 -4.05011494

H -7.94274206 -0.89325626 -5.10770612

H 1.53540810 -7.07298150 5.46697193

H -0.22694246 -7.07120290 5.67194002

H 0.51904458 -8.09389298 4.41886592

H 8.34010456 1.57124591 -4.37137411

H 8.95718062 0.10620761 -3.57985643

H 7.97303721 -0.03267529 -5.05945623

C -0.00427938 -0.27616702 -2.28816030

O -0.01284219 -0.92613794 -3.23035923

[CoP(CO)]^0^ M = 4

C 2.66704170 -1.16555688 -0.80536146

C 3.18763070 -2.46293976 -1.05574498

C 2.33812939 -3.37509872 -0.43310673

C 1.28363477 -2.63289996 0.14807443

N 1.46858192 -1.30369529 -0.10250746

C 3.22549571 0.11476405 -1.12693357

C 0.10173684 -3.15006099 0.84799617

C -1.18057554 -2.67004296 0.34524592

C -2.38432218 -3.40341249 0.15839508

C -3.28489266 -2.55742528 -0.47361452

C -2.63069941 -1.30918092 -0.66677578

N -1.34414478 -1.40784432 -0.15482364

C -3.23563970 -0.09247462 -1.12785181

C -2.67368231 1.18567978 -0.80942778

C -3.19768712 2.48504550 -1.05446418

C -2.35241733 3.39411611 -0.42516020

C -1.29580875 2.64854142 0.15283353

N -1.47618801 1.32258783 -0.10817121

C -0.11282125 3.16607814 0.85007278

C 1.16850690 2.68537856 0.34575456

C 2.37152158 3.42289090 0.15673230

C 3.26952234 2.58066104 -0.48031666

C 2.61469541 1.33014147 -0.67343069

N 1.32994350 1.42672443 -0.15988967

C -0.22569924 4.09586130 1.88338900

C -4.48162171 -0.19694357 -1.85332775

C 0.21651825 -4.07636266 1.88338586

C 4.47627423 0.21658925 -1.84412394

C -4.69477283 -1.22617662 -2.82778329

C -5.85439822 -1.31714472 -3.54325675

N -6.89583236 -0.43513364 -3.34550141

C -6.75598714 0.53624368 -2.38313672

C -5.60066654 0.67919549 -1.66711778

C 0.90124425 4.58796535 2.66111354

C 0.75951083 5.51839539 3.64082131

N -0.46574361 6.04465697 3.99034459

C -1.57948392 5.55061605 3.34358078

C -1.49752823 4.61726207 2.36004966

C 5.59320011 -0.66016348 -1.64547406

C 6.75506711 -0.52039754 -2.35073677

N 6.91188869 0.46090794 -3.30102808

C 5.86683404 1.33168609 -3.52293621

C 4.69963679 1.24293262 -2.81903910

C 1.48953902 -4.59586604 2.35956350

C 1.57371957 -5.52384983 3.34777449

N 0.46147709 -6.01483788 3.99973936

C -0.76489528 -5.49182067 3.64885677

C -0.90909229 -4.56678698 2.66453128

C -0.56940263 7.16481335 4.92080637

C -8.15719082 -0.61368049 -4.06664057

C 0.56759103 -7.13147418 4.93408634

C 8.11555220 0.51951178 -4.13176273

Co -0.00561360 0.00880442 -0.24012805

H 4.07145901 -2.70548850 -1.62707589

H 2.43678978 -4.45294003 -0.41161926

H -2.54131626 -4.43991242 0.42450763

H -4.30908524 -2.78323574 -0.73776504

H -4.08114525 2.72866510 -1.62587994

H -2.45085547 4.47185174 -0.40001775

H 2.52705624 4.45974688 0.42234785

H 4.29227658 2.80853846 -0.74821395

H -3.90168509 -1.93126067 -3.04579850

H -6.00803872 -2.06482736 -4.31185691

H -7.62046301 1.16786072 -2.21796631

H -5.57503460 1.44007952 -0.89845753

H 1.89158637 4.18582840 2.49314402

H 1.60444818 5.88037903 4.21617627

H -2.53038873 5.94491749 3.68457370

H -2.42375749 4.25238752 1.93483068

H 5.56058561 -1.41632450 -0.87230379

H 7.61644261 -1.15479939 -2.17955867

H 6.02663813 2.07516156 -4.29416325

H 3.91108945 1.95079709 -3.04435229

H 2.41464335 -4.23317793 1.92996942

H 2.52541528 -5.91627412 3.68876865

H -1.60873820 -5.85169621 4.22717373

H -1.90022730 -4.16697241 2.49547912

H -0.50719027 8.12627774 4.39677017

H -1.52283516 7.11185626 5.45196129

H 0.24018447 7.10748794 5.65219972

H -8.75862623 -1.40343345 -3.60341601

H -8.71854403 0.32146939 -4.05155166

H -7.94920439 -0.88282872 -5.10491359

H 1.52223158 -7.07624010 5.46282604

H -0.24018586 -7.07185734 5.66734000

H 0.50454931 -8.09498427 4.41388297

H 8.34164864 1.56023056 -4.37278987

H 8.95662162 0.09433477 -3.58122864

H 7.97227396 -0.04317754 -5.06081639

C -0.00627546 -0.26945631 -2.28870748

O -0.01561991 -0.90961365 -3.23760260

[CoP(CO)]^0^ M = 6

C 2.87163482 -0.73418837 -0.75956635

C 3.44293236 -1.89109098 -1.35792515

C 2.71152527 -2.98964238 -0.90309390

C 1.69753333 -2.48180471 -0.05598143

N 1.78242115 -1.12504134 0.01381335

C 3.28861354 0.63657554 -0.89966601

C 0.64115581 -3.24133253 0.64876083

C -0.74002517 -2.92151771 0.31224994

C -1.86515419 -3.79485373 0.28638792

C -2.94164434 -3.06499326 -0.19530436

C -2.47873998 -1.74585138 -0.46674449

N -1.12938292 -1.68176090 -0.14061289

C -3.27333928 -0.63887189 -0.92041392

C -2.85572867 0.73248258 -0.78001167

C -3.41930965 1.88824955 -1.38589669

C -2.69120037 2.98744573 -0.92563648

C -1.68615353 2.48089700 -0.06843392

N -1.77373364 1.12352194 0.00432241

C -0.63578125 3.24093925 0.64448553

C 0.74780495 2.92187548 0.31841348

C 1.87269203 3.79430192 0.29730779

C 2.95277097 3.06340889 -0.17697584

C 2.49266915 1.74438081 -0.44856134

N 1.14155039 1.68016433 -0.12718397

C -0.99693192 4.23253156 1.56018509

C -4.55869000 -0.91916794 -1.51546292

C 0.99520118 -4.23136016 1.56899170

C 4.57556033 0.91808032 -1.49119049

C -4.78533401 -2.04820315 -2.37159308

C -6.00024530 -2.28861382 -2.94664214

N -7.08293983 -1.46771977 -2.72034196

C -6.92127362 -0.38808594 -1.88047788

C -5.71565932 -0.09662151 -1.30912952

C -0.06562286 4.93533829 2.42914087

C -0.46291520 5.90993173 3.28905581

N -1.78018254 6.29114194 3.41799953

C -2.71750603 5.60520515 2.67447238

C -2.37806375 4.61582470 1.80747330

C 5.73389635 0.09860208 -1.27900260

C 6.94092237 0.39180129 -1.84634175

N 7.10816383 1.48208380 -2.67115762

C 6.01869585 2.28768637 -2.91980329

C 4.80214686 2.04541347 -2.34876565

C 2.37439770 -4.61489173 1.82705927

C 2.70717485 -5.60309910 2.69793472

N 1.76424191 -6.28751450 3.43580197

C 0.44813618 -5.90570237 3.29694607

C 0.05748833 -4.93216379 2.43279993

C -2.15628530 7.45002808 4.22292786

C -8.40150347 -1.80140035 -3.26034744

C 2.13422056 -7.44507199 4.24539144

C 8.37688134 1.70864693 -3.36448054

Co 0.00483777 -0.00403412 -0.15666267

H 4.27390677 -1.91357403 -2.05032419

H 2.87087288 -4.03305122 -1.14810411

H -1.86303105 -4.84721177 0.53406610

H -3.95397529 -3.42378377 -0.32405674

H -4.24373719 1.91015351 -2.08614392

H -2.84738511 4.03051514 -1.17415795

H 1.86933944 4.84693052 0.54387929

H 3.96574928 3.42241945 -0.29993949

H -3.96415672 -2.71023225 -2.61740657

H -6.16891496 -3.11658558 -3.62446674

H -7.81024800 0.20254583 -1.69471933

H -5.65889943 0.75729224 -0.64584098

H 0.97911563 4.65628520 2.44395902

H 0.23792542 6.42270692 3.93869133

H -3.74851207 5.89924116 2.83675943

H -3.17822721 4.10651987 1.28553232

H 5.67758654 -0.75136691 -0.61059448

H 7.82943825 -0.19934200 -1.65976267

H 6.18622198 3.11154653 -3.60281524

H 3.98127407 2.70742157 -2.59564336

H 3.17869644 -4.10676363 1.31037586

H 3.73687393 -5.89730186 2.86812872

H -0.25757144 -6.41695314 3.94251223

H -0.98713263 -4.65240299 2.44012515

H -2.12782691 8.37259387 3.63079453

H -3.16681361 7.31067862 4.61419524

H -1.46735243 7.54800299 5.06514631

H -8.95961458 -2.43242434 -2.55964485

H -8.96309713 -0.88305577 -3.44270740

H -8.28222045 -2.33440716 -4.20564688

H 3.14100437 -7.30428097 4.64574833

H 1.43773877 -7.54276539 5.08139255

H 2.11211282 -8.36835512 3.65409039

H 8.51979992 2.77951908 -3.52225981

H 9.19754336 1.33089963 -2.75148150

H 8.38635977 1.19684054 -4.33325965

C 0.01410532 0.19468766 -2.33224752

O 0.00592824 0.51661826 -3.42335666

[CoP(CO)]^–1^ M = 1

C 2.37063140 -1.18685478 -0.72786056

C 2.87606597 -2.50331526 -0.91041595

C 2.08573037 -3.35920300 -0.14453671

C 1.07305003 -2.57185589 0.45593721

N 1.23520987 -1.27057765 0.07896523

C 2.88100111 0.06879698 -1.20786537

C -0.04771698 -3.01967056 1.29735628

C -1.38241083 -2.57005467 0.87768042

C -2.60649955 -3.26169219 0.87288088

C -3.54834779 -2.43648930 0.21827656

C -2.87949310 -1.26376946 -0.16224620

N -1.56747103 -1.35061760 0.25643250

C -3.37991122 -0.06535490 -0.86215903

C -2.92431014 1.22405360 -0.31291441

C -3.62649569 2.41710816 -0.07906033

C -2.71993597 3.32274084 0.51680559

C -1.48759663 2.65669773 0.63566710

N -1.62801873 1.38271632 0.12534126

C -0.18806377 3.15407055 1.11916675

C 0.99141756 2.70334406 0.37319853

C 2.15022764 3.44162911 -0.00225146

C 2.97240326 2.58171675 -0.71922497

C 2.31408898 1.31890302 -0.77675854

N 1.10139577 1.43594580 -0.11977389

C -0.07318582 3.99907085 2.22149923

C -4.21835313 -0.15014772 -1.96275554

C 0.17107092 -3.85219029 2.38858319

C 4.04414132 0.12021811 -2.06102210

C -4.65885141 -1.40997540 -2.56084480

C -5.51177699 -1.46242105 -3.61239374

N -6.01110903 -0.32511761 -4.22905857

C -5.56495772 0.89839586 -3.75274855

C -4.71318825 1.00918565 -2.70472048

C 1.19188704 4.48443830 2.76351338

C 1.25738899 5.35455335 3.80299325

N 0.13200942 5.81555021 4.46012512

C -1.08761128 5.30098122 4.06150423

C -1.21051601 4.43384042 3.02525144

C 5.17906726 -0.75039746 -1.93969882

C 6.25321139 -0.65983494 -2.77829421

N 6.30338912 0.26425715 -3.79635170

C 5.23921229 1.12974168 -3.94708200

C 4.15968624 1.08950617 -3.11238735

C 1.48535862 -4.33874264 2.79669356

C 1.65749244 -5.19042256 3.83840223

N 0.60610618 -5.62894338 4.62419592

C -0.65024054 -5.12580845 4.33989078

C -0.88143688 -4.28047521 3.30468327

C 0.21370241 6.90773337 5.42152320

C -7.12684732 -0.40557950 -5.16031836

C 0.78774019 -6.70207949 5.59303750

C 7.39975596 0.26614763 -4.76472004

Co -0.23616641 0.03663768 -0.06305129

H 3.71017215 -2.79746344 -1.53029319

H 2.19673586 -4.43115303 -0.03960359

H -2.77549560 -4.26216218 1.25109769

H -4.59830060 -2.64978885 0.05618545

H -4.67572388 2.58977491 -0.28722810

H -2.91871186 4.35068426 0.79424121

H 2.33282753 4.48591859 0.21390177

H 3.95213455 2.80637927 -1.11899314

H -4.27545972 -2.34486930 -2.17167443

H -5.83126420 -2.40300730 -4.04966753

H -5.92561908 1.76526963 -4.29727302

H -4.37222793 1.99971759 -2.43074040

H 2.12795083 4.12730182 2.35501833

H 2.20368458 5.71368967 4.19366101

H -1.94137125 5.61936821 4.65039327

H -2.19453401 4.03653542 2.81397672

H 5.23651747 -1.45778672 -1.12327389

H 7.12862546 -1.28855372 -2.66731958

H 5.31183085 1.82745082 -4.77241142

H 3.35144782 1.78942120 -3.28708342

H 2.37037960 -4.00732020 2.26800470

H 2.63708730 -5.55585934 4.12788075

H -1.43853364 -5.43542261 5.01799221

H -1.88325058 -3.88861814 3.18564049

H 0.14654817 7.88681898 4.92966263

H -0.60204838 6.82106328 6.14394563

H 1.16238098 6.85033212 5.96174299

H -8.09675106 -0.39418214 -4.64392375

H -7.08970508 0.44149535 -5.85104824

H -7.05261495 -1.32846771 -5.74238803

H 1.78389363 -6.63002651 6.03777975

H 0.04550915 -6.60493819 6.38969978

H 0.67949470 -7.69150369 5.12952400

H 7.63276518 1.29401932 -5.05226260

H 8.28537960 -0.18150272 -4.31039298

H 7.12817952 -0.30493941 -5.65968624

C -0.40616517 -0.40241491 -2.03163569

O -0.50685152 -1.12840321 -2.91559276

[CoP(CO)]^–1^ M = 3

C 2.37102816 -1.18685281 -0.72712139

C 2.87690612 -2.50333563 -0.90860105

C 2.08638999 -3.35896106 -0.14276546

C 1.07302117 -2.57165720 0.45664267

N 1.23499143 -1.27039503 0.07891493

C 2.88144696 0.06861068 -1.20751619

C -0.04782313 -3.01973162 1.29761899

C -1.38243816 -2.56987791 0.87832360

C -2.60673218 -3.26131181 0.87403196

C -3.54847304 -2.43631994 0.21912401

C -2.87945136 -1.26386689 -0.16199874

N -1.56743897 -1.35072545 0.25650123

C -3.37975152 -0.06547226 -0.86198274

C -2.92433058 1.22388031 -0.31248480

C -3.62675363 2.41671098 -0.07815310

C -2.72029662 3.32238587 0.51770039

C -1.48770368 2.65660898 0.63605575

N -1.62798503 1.38268884 0.12541200

C -0.18819216 3.15410532 1.11909860

C 0.99132558 2.70298245 0.37351833

C 2.15038138 3.44110685 -0.00124108

C 2.97265891 2.58141785 -0.71842726

C 2.31432359 1.31872175 -0.77655100

N 1.10139649 1.43559034 -0.11995906

C -0.07322187 3.99940462 2.22129354

C -4.21814279 -0.15025009 -1.96262641

C 0.17089364 -3.85262665 2.38872604

C 4.04429028 0.12012744 -2.06085573

C -4.65866833 -1.41008282 -2.56064852

C -5.51142745 -1.46253406 -3.61233355

N -6.01055786 -0.32523173 -4.22916607

C -5.56462018 0.89829177 -3.75269130

C -4.71302198 1.00909275 -2.70451879

C 1.19185906 4.48463146 2.76337807

C 1.25741267 5.35511365 3.80255876

N 0.13203817 5.81663656 4.45931941

C -1.08759635 5.30198523 4.06086953

C -1.21050990 4.43447523 3.02491351

C 5.17915068 -0.75084271 -1.94043060

C 6.25292343 -0.66022605 -2.77945371

N 6.30298275 0.26424805 -3.79714482

C 5.23900734 1.13020075 -3.94695111

C 4.15984776 1.08993381 -3.11183527

C 1.48511201 -4.33911439 2.79700265

C 1.65710746 -5.19124269 3.83839352

N 0.60558751 -5.63025311 4.62369124

C -0.65066072 -5.12684892 4.33954232

C -0.88167192 -4.28107221 3.30463007

C 0.21378712 6.90922134 5.42025906

C -7.12608945 -0.40577835 -5.16069108

C 0.78712962 -6.70368527 5.59223928

C 7.39890481 0.26625539 -4.76602125

Co -0.23613011 0.03639489 -0.06511625

H 3.71135506 -2.79769651 -1.52788744

H 2.19762962 -4.43082740 -0.03726802

H -2.77579258 -4.26171359 1.25238593

H -4.59848067 -2.64950466 0.05720604

H -4.67601462 2.58927164 -0.28625408

H -2.91923333 4.35027556 0.79519790

H 2.33295761 4.48535590 0.21512207

H 3.95263291 2.80608348 -1.11760695

H -4.27548404 -2.34497847 -2.17126818

H -5.83097300 -2.40312147 -4.04955607

H -5.92535812 1.76517367 -4.29714792

H -4.37230239 1.99965617 -2.43033301

H 2.12791318 4.12707269 2.35524743

H 2.20373479 5.71411763 4.19328420

H -1.94135220 5.62059968 4.64964171

H -2.19453763 4.03708110 2.81387870

H 5.23696127 -1.45842566 -1.12421660

H 7.12821715 -1.28922823 -2.66907388

H 5.31151765 1.82829185 -4.77197017

H 3.35178985 1.79021147 -3.28589819

H 2.37027422 -4.00724569 2.26885099

H 2.63668614 -5.55662717 4.12798592

H -1.43903087 -5.43657479 5.01749933

H -1.88340718 -3.88896962 3.18580421

H 0.14669501 7.88809315 4.92797013

H -0.60197954 6.82290629 6.14270581

H 1.16246048 6.85199070 5.96050362

H -8.09608405 -0.39497848 -4.64446410

H -7.08913937 0.44159349 -5.85105622

H -7.05136123 -1.32840059 -5.74311950

H 1.78315437 -6.63162293 6.03726502

H 0.04465737 -6.60692494 6.38871837

H 0.67915842 -7.69294605 5.12833178

H 7.63249788 1.29422689 -5.05274453

H 8.28441784 -0.18241683 -4.31249004

H 7.12653357 -0.30383887 -5.66137703

C -0.40801333 -0.40141248 -2.03097371

O -0.50406936 -1.12382666 -2.91842066
