# Supplementary material for: Self-Assembled Liposomes Enhance Electron Transfer for Efficient Photocatalytic CO2 Reduction
Source: J Am Chem Soc. 2022 May 20;144(21):9399–412. doi: 10.1021/jacs.2c01725 (PMC9164230; doi:10.1021/jacs.2c01725)
Supplement: Supplementary file 2 — ja2c01725_si_002.zip [file ja2c01725_si_002.zip › Coordinates/CoP_CO2/Coord_CoP_CO2.docx]

[CoP(CO_2_)]^+3^ M = 1

C 2.55848681 1.61927388 -0.12051593

C 3.99898940 1.49514574 -0.15177212

C 4.27463559 0.16914984 -0.20694221

C 3.00174133 -0.51777258 -0.22493257

N 1.95944660 0.38085071 -0.17382126

C 1.89015386 2.84456201 -0.09466514

C 2.86871777 -1.90861511 -0.22680496

C 1.64097138 -2.57467756 -0.19151844

C 1.50471174 -4.01406542 -0.19511989

C 0.17563407 -4.27823414 -0.14853758

C -0.50277064 -3.00100197 -0.14884728

N 0.40523973 -1.96632923 -0.17403856

C -1.89130558 -2.85857418 -0.10247652

C -2.56001905 -1.63242395 -0.12413310

C -4.00068963 -1.50658924 -0.16075468

C -4.27497188 -0.18002990 -0.21274685

C -3.00194232 0.50550822 -0.22266034

N -1.96026846 -0.39472261 -0.17250026

C -2.86850577 1.89569366 -0.21845438

C -1.64042978 2.55978694 -0.17909164

C -1.50405049 3.99959731 -0.17972060

C -0.17548798 4.26311618 -0.13331822

C 0.50208765 2.98518435 -0.13494958

N -0.40549315 1.95002570 -0.16117899

C -4.10754472 2.71920746 -0.24151644

C -2.71871420 -4.09250782 -0.03677130

C 4.10989127 -2.72680708 -0.25068612

C 2.71546229 4.08244107 -0.03584480

C -2.73216306 -5.02566812 -1.08808109

C -3.51802183 -6.15678261 -1.00179025

N -4.28664631 -6.38799392 0.09043056

C -4.29570320 -5.51060071 1.11998470

C -3.52455578 -4.36385899 1.07968339

C -4.45184168 3.54437221 0.83993226

C -5.60631983 4.30370543 0.79588601

N -6.42157475 4.26303917 -0.28300661

C -6.11821836 3.47613110 -1.34333136

C -4.97590764 2.70036535 -1.34581259

C 3.50094440 4.37546834 1.08846821

C 4.26312321 5.52914357 1.12266353

N 4.26352079 6.38996983 0.07978070

C 3.51547379 6.13599561 -1.02112844

C 2.73963629 4.99690755 -1.10228998

C 4.99028704 -2.68450321 -1.34585270

C 6.13973595 -3.44829442 -1.34225723

N 6.43941130 -4.24883216 -0.29051631

C 5.61328227 -4.31306338 0.77876807

C 4.45062283 -3.56629140 0.82113334

C -7.63996581 5.10516023 -0.33524222

C -5.12025795 -7.61170527 0.13240996

C 7.68723566 -5.04584814 -0.33106044

C 5.08281802 7.62446583 0.11850244

Co -0.00043884 -0.00819487 -0.04360054

H 4.70600192 2.31141205 -0.14575946

H 5.24853617 -0.29667781 -0.22558302

H 2.31385551 -4.72811495 -0.23445108

H -0.29645279 -5.24883367 -0.11805784

H -4.70952913 -2.32124376 -0.16304049

H -5.24776732 0.28808270 -0.23593136

H -2.31293608 4.71385849 -0.21778800

H 0.29773975 5.23309983 -0.10312992

H -2.13756618 -4.86502212 -1.97897977

H -3.56714251 -6.89770871 -1.78968467

H -4.92530848 -5.75808090 1.96440922

H -3.54920367 -3.68459614 1.92294789

H -3.82603896 3.59163001 1.72260388

H -5.90936269 4.94832028 1.61049319

H -6.81037742 3.50071142 -2.17537257

H -4.75866809 2.08714926 -2.21181240

H 3.51652192 3.71016308 1.94300982

H 4.87712326 5.79492743 1.97293659

H 3.56989164 6.86582371 -1.81896446

H 2.15913683 4.81882973 -1.99922454

H 4.77606316 -2.06078608 -2.20502627

H 6.84252959 -3.45270474 -2.16576583

H 5.91515866 -4.96444575 1.58822564

H 3.81616347 -3.63236405 1.69638629

H -7.46391865 5.93649040 -1.02028119

H -8.47499306 4.49777121 -0.68505512

H -7.85294393 5.48303404 0.66303757

H -4.47259042 -8.48396635 0.03451863

H -5.65019297 -7.64921005 1.08229550

H -5.83626295 -7.57893795 -0.69033202

H 8.53878531 -4.36636071 -0.38829814

H 7.75447520 -5.64780038 0.57316449

H 7.66297320 -5.69569534 -1.20709299

H 5.60900671 7.67280714 1.06991377

H 5.80120161 7.59569108 -0.70213896

H 4.42436340 8.48775865 0.01415862

C 0.00253646 -0.02200738 1.99288744

O 0.64283311 0.92444399 2.44012543

O -0.63502855 -0.97482240 2.43187897

[CoP(CO_2_)]^+3^_2 M = 1

C 2.79785614 -1.15174934 -0.09477418

C 3.52505867 -2.40185592 -0.13171773

C 2.59883608 -3.38994589 -0.17984837

C 1.30493315 -2.74376402 -0.18773259

N 1.44019489 -1.37375562 -0.13954979

C 3.41573555 0.10001483 -0.07047743

C 0.09199191 -3.43549000 -0.18143786

C -1.15810812 -2.81453695 -0.14560135

C -2.41261032 -3.53305830 -0.14038526

C -3.39421238 -2.59895185 -0.10230985

C -2.74089916 -1.30827681 -0.11436932

N -1.37222445 -1.45363913 -0.13728015

C -3.42508538 -0.09135609 -0.08123043

C -2.80841587 1.16143773 -0.10674293

C -3.53539341 2.41105172 -0.14386837

C -2.60896718 3.40002993 -0.18927522

C -1.31481483 2.75479082 -0.19728369

N -1.45081880 1.38500445 -0.15092693

C -0.10036079 3.44583336 -0.18388287

C 1.15050506 2.82437049 -0.14435772

C 2.40665527 3.54117606 -0.14266997

C 3.38702138 2.60581002 -0.10193528

C 2.73216364 1.31651056 -0.10848414

N 1.36322363 1.46377961 -0.13373446

C -0.14091485 4.93162750 -0.19372879

C -4.91089335 -0.13035380 -0.02262314

C 0.13672038 -4.92317827 -0.19584697

C 4.90282067 0.13367008 -0.01320120

C -5.67282323 -0.66321795 -1.07683433

C -7.05025031 -0.68858330 -0.99355267

N -7.68965550 -0.20224394 0.09805437

C -6.98594166 0.31896643 1.12901952

C -5.60466775 0.36546785 1.09169292

C 0.34854012 5.67941214 0.88903237

C 0.30079874 7.06016427 0.86025564

N -0.21756226 7.71394492 -0.20532381

C -0.69778379 7.02230962 -1.26706037

C -0.67137986 5.64218924 -1.28416618

C 5.59405868 -0.35417872 1.10559313

C 6.97648025 -0.32770068 1.13532309

N 7.68200003 0.16828449 0.09365752

C 7.04437592 0.65028114 -1.00063215

C 5.66600560 0.64378290 -1.07707656

C 0.64324671 -5.62673908 -1.29852739

C 0.68612198 -7.00916414 -1.28145144

N 0.24384301 -7.70241736 -0.20775108

C -0.25525477 -7.05266789 0.87154280

C -0.31919010 -5.67400810 0.90079413

C -0.23142958 9.19467228 -0.24163506

C -9.16971652 -0.24717118 0.13946350

C 0.28229710 -9.18406807 -0.19766487

C 9.16357386 0.18624669 0.12441624

Co -0.00536482 0.00552297 -0.01386335

H 4.59930569 -2.51143017 -0.13518444

H 2.77665475 -4.45467889 -0.20206609

H -2.52873248 -4.60636249 -0.16817877

H -4.45939056 -2.77225061 -0.06938124

H -4.60980517 2.52020232 -0.14840310

H -2.78796894 4.46473347 -0.20804311

H 2.52605917 4.61381744 -0.17728453

H 4.45283825 2.77630460 -0.07243215

H -5.19238551 -1.05300122 -1.96585502

H -7.67781031 -1.08372286 -1.78239311

H -7.55718778 0.68256712 1.97290617

H -5.06996417 0.78067850 1.93715340

H 0.75866605 5.18649121 1.76185108

H 0.66090431 7.67143204 1.67736534

H -1.08751524 7.60982314 -2.08858071

H -1.05736408 5.11967013 -2.15085349

H 5.05811231 -0.75300723 1.95811895

H 7.54657429 -0.69074776 1.98019803

H 7.67373298 1.02440009 -1.79820314

H 5.18689851 1.02661738 -1.96986277

H 1.00265838 -5.10173342 -2.17509262

H 1.06408220 -7.58882492 -2.11332232

H -0.58467511 -7.67196710 1.69603590

H -0.71237595 -5.18378102 1.78292244

H 0.56015007 9.53860866 -0.91005484

H -1.20326325 9.53105887 -0.60342579

H -0.06240385 9.57565755 0.76403873

H -9.49452051 -1.28353623 0.03849031

H -9.51150680 0.15624112 1.09076847

H -9.56389498 0.35370276 -0.68164830

H 0.83132465 -9.53123256 -1.07089714

H 0.78592348 -9.51684699 0.71054228

H -0.74042059 -9.56389295 -0.22371925

H 9.50633147 1.21502492 0.00688634

H 9.50462594 -0.21095961 1.07852514

H 9.53997929 -0.43312619 -0.69118910

C -0.01458837 0.01407456 2.02243187

O -1.16085398 -0.00271643 2.46140517

O 1.12793722 0.03628075 2.46983370

[CoP(CO_2_)]^+2^ M = 2

C 2.62897803 -1.21390470 -0.85955747

C 3.25196624 -2.50175274 -1.00723300

C 2.41207533 -3.41548113 -0.42828079

C 1.24950858 -2.69126786 0.02136057

N 1.37970415 -1.37359607 -0.27884107

C 3.19686876 0.03555140 -1.15463344

C 0.04072198 -3.26246091 0.55947692

C -1.19239461 -2.70830639 0.06765936

C -2.38546750 -3.44200157 -0.27958075

C -3.24624492 -2.55080255 -0.86083376

C -2.59322445 -1.26902032 -0.83969422

N -1.32865283 -1.40609364 -0.29391666

C -3.18817637 -0.03581386 -1.15218324

C -2.61742385 1.21332511 -0.86413853

C -3.23959561 2.50112117 -1.01619257

C -2.39954600 3.41585528 -0.44004924

C -1.23781854 2.69182594 0.01265518

N -1.36764377 1.37371924 -0.28360822

C -0.03058351 3.26553119 0.55154755

C 1.20355473 2.71224473 0.06139499

C 2.39762346 3.44402902 -0.28550279

C 3.25713489 2.55160346 -0.86786149

C 2.60225904 1.27067527 -0.84694413

N 1.33909596 1.40966342 -0.30041021

C -0.06542819 4.34340946 1.49269912

C -4.54292273 -0.06847772 -1.75900479

C 0.06822449 -4.34050845 1.50074567

C 4.54812334 0.06713106 -1.76515239

C -4.76057044 -0.72062677 -2.98562168

C -6.02072500 -0.74078405 -3.54788629

N -7.06686548 -0.13896687 -2.93145403

C -6.89385854 0.48649322 -1.74450993

C -5.65039383 0.53415081 -1.14194638

C 1.11156086 4.88470443 2.10606927

C 1.04945094 5.91622442 3.00337947

N -0.13854246 6.46315222 3.39094103

C -1.29311557 5.91939021 2.90978931

C -1.28574381 4.88623813 2.01222248

C 5.65776347 -0.53791045 -1.15347779

C 6.89932106 -0.48799377 -1.75864718

N 7.06948831 0.14140265 -2.94406996

C 6.02069961 0.74279254 -3.55787017

C 4.76294887 0.72195422 -2.99185622

C 1.28292582 -4.89940755 2.01635833

C 1.27886329 -5.93441761 2.91187241

N 0.11884987 -6.46464473 3.39475845

C -1.06320916 -5.90113249 3.01231225

C -1.11380687 -4.86691152 2.11751676

C -0.17128251 7.63054884 4.28470562

C -8.40679361 -0.20241868 -3.56106232

C 0.13857808 -7.63559205 4.28414527

C 8.39897257 0.18228338 -3.59453970

Co 0.00632368 0.00128790 -0.09329064

H 4.19345144 -2.70485108 -1.49701814

H 2.53923984 -4.48820014 -0.39700431

H -2.52707774 -4.50798988 -0.17526961

H -4.23273673 -2.75756313 -1.25095641

H -4.18167465 2.70223636 -1.50549581

H -2.52640995 4.48863422 -0.41009267

H 2.54041935 4.50997716 -0.18200590

H 4.24378615 2.75761205 -1.25825372

H -3.94310997 -1.20291017 -3.50724000

H -6.23133745 -1.22292186 -4.49399837

H -7.77204409 0.93377354 -1.29767164

H -5.55082781 1.03287719 -0.18612648

H 2.08728775 4.46849792 1.90215031

H 1.93837693 6.33609087 3.45777572

H -2.21480837 6.34302310 3.28894281

H -2.24337260 4.47384814 1.73008663

H 5.56064838 -1.03730405 -0.19778087

H 7.77892486 -0.93560016 -1.31518188

H 6.22893205 1.22288865 -4.50570923

H 3.94373090 1.20593320 -3.50913569

H 2.24498054 -4.49882659 1.73232296

H 2.19579121 -6.37094885 3.28799609

H -1.95646628 -6.30992468 3.46835674

H -2.08444292 -4.43696625 1.91786598

H -0.11540259 8.55058927 3.69629547

H -1.09880965 7.61894624 4.85801479

H 0.67491860 7.58282166 4.97120180

H -8.78966625 -1.22077389 -3.47499902

H -9.07298352 0.49050237 -3.05045347

H -8.31710050 0.07922455 -4.61057024

H 1.06799302 -7.63852636 4.85457666

H -0.70477202 -7.57917848 4.97340882

H 0.06802885 -8.55261611 3.69260414

H 8.68617764 1.22344081 -3.74763203

H 9.12576516 -0.31058652 -2.95149766

H 8.33964999 -0.33719604 -4.55214424

C 0.01814629 0.00701363 1.91189336

O -1.11667619 -0.06990392 2.39209619

O 1.15836582 0.08669068 2.38032225

[CoP(CO_2_)]^+2^ M = 4

C 2.71195487 -1.15668304 -0.54893218

C 3.22729140 -2.46195964 -0.85650330

C 2.35560417 -3.38008124 -0.30956471

C 1.30147051 -2.63270726 0.29648878

N 1.49356920 -1.30609011 0.11135088

C 3.27475425 0.11308102 -0.81573547

C 0.09595227 -3.19134894 0.93247051

C -1.15121755 -2.69910083 0.46667870

C -2.37922165 -3.43826377 0.26900639

C -3.24595728 -2.59946411 -0.37662939

C -2.56697756 -1.33614502 -0.52477070

N -1.30010444 -1.42759096 -0.02833074

C -3.15902060 -0.12104771 -0.98252674

C -2.61481934 1.13161167 -0.72766990

C -3.18245240 2.43335740 -0.99419338

C -2.38217645 3.34407404 -0.36669704

C -1.29525088 2.60196724 0.24732080

N -1.41905482 1.29755673 -0.02332865

C -0.15417618 3.15745348 0.97214682

C 1.13310246 2.66406415 0.60782282

C 2.38032129 3.38318220 0.58155702

C 3.31071735 2.54795027 0.01519918

C 2.63564574 1.31757465 -0.29720215

N 1.33004897 1.40794686 0.06178783

C -0.34358162 4.15707241 1.98054769

C -4.44019949 -0.22491934 -1.71525863

C 0.19542404 -4.21723367 1.92941525

C 4.51933829 0.27112772 -1.52919985

C -4.53033090 -1.05050695 -2.85180535

C -5.71360448 -1.13611198 -3.55462231

N -6.80867033 -0.43941406 -3.16094639

C -6.76034324 0.34832513 -2.06212739

C -5.59643693 0.47068598 -1.32667109

C 0.71130264 4.57442850 2.85572636

C 0.52138282 5.53848509 3.80856820

N -0.69237343 6.13092650 3.99835306

C -1.75179644 5.70215326 3.25301826

C -1.61497207 4.74028093 2.28864212

C 5.63601005 -0.60667448 -1.39415079

C 6.79119685 -0.40776821 -2.10514238

N 6.92232725 0.62864194 -2.98259942

C 5.88002247 1.50400211 -3.13680269

C 4.71639648 1.36075108 -2.43114048

C 1.44088344 -4.80361925 2.31318070

C 1.50787926 -5.78005306 3.27258145

N 0.39477577 -6.22292469 3.92224431

C -0.80640507 -5.64234351 3.64356012

C -0.92693017 -4.65823335 2.69784979

C -0.85483617 7.23004756 4.96222294

C -8.06453326 -0.58104849 -3.93238874

C 0.48640308 -7.33461826 4.88364252

C 8.14416609 0.80526088 -3.78185326

Co 0.02862295 -0.01015402 -0.05735273

H 4.11712679 -2.68812697 -1.42333563

H 2.42372839 -4.45806693 -0.36070453

H -2.53470667 -4.48131501 0.50375701

H -4.25267571 -2.82222007 -0.70137392

H -4.07112081 2.63783413 -1.57358306

H -2.48805594 4.41953968 -0.36975795

H 2.52684136 4.41198978 0.87645229

H 4.35549499 2.75858305 -0.16246011

H -3.66986623 -1.61049200 -3.19711474

H -5.82418700 -1.74621018 -4.44200788

H -7.67327803 0.86269407 -1.79221856

H -5.60063446 1.09383738 -0.44146000

H 1.68347310 4.10496249 2.81457206

H 1.31948504 5.86189981 4.46498908

H -2.70719395 6.16150160 3.47422814

H -2.51504509 4.42707671 1.78009058

H 5.62115673 -1.41137429 -0.67170729

H 7.65637012 -1.04827350 -1.98847867

H 6.03026194 2.29967108 -3.85579344

H 3.92129651 2.07586744 -2.60398213

H 2.36902320 -4.48412311 1.86111916

H 2.44429917 -6.24186235 3.55947110

H -1.65176974 -5.98894157 4.22432588

H -1.89701189 -4.19576916 2.58379601

H -0.75708349 8.19056211 4.44886934

H -1.83967016 7.16050177 5.42636888

H -0.08805664 7.14799776 5.73290231

H -8.48569534 -1.57023738 -3.74373249

H -8.76599892 0.18912075 -3.61683551

H -7.84216640 -0.46291867 -4.99329951

H 1.44374002 -7.28308816 5.40307692

H -0.32286610 -7.24897696 5.60897836

H 0.40576542 -8.28652228 4.35216553

H 8.48047111 1.84156983 -3.70846935

H 8.92265423 0.14703302 -3.39723591

H 7.94142147 0.55765790 -4.82735533

C 0.07749865 -0.01268502 -2.10960748

O 0.01314794 1.12487519 -2.58057812

O 0.16009866 -1.15268349 -2.57036002

[CoP(CO_2_)]^+1^ M = 1

C 2.62987427 -1.21909684 -0.87774087

C 3.23908158 -2.50641449 -1.03977199

C 2.39118500 -3.42185299 -0.46049032

C 1.24885871 -2.69452023 0.01439528

N 1.38651458 -1.37743739 -0.26880166

C 3.18993105 0.03337563 -1.17829325

C 0.03887083 -3.25408918 0.59532889

C -1.19743948 -2.71221678 0.05759600

C -2.37162001 -3.44777056 -0.32030703

C -3.23812158 -2.55146955 -0.90033739

C -2.59527223 -1.27098668 -0.86265340

N -1.33655918 -1.40927074 -0.28426937

C -3.18205423 -0.03466853 -1.17573587

C -2.61942099 1.21699600 -0.88079418

C -3.22641197 2.50405769 -1.04846081

C -2.37702640 3.42084637 -0.47359671

C -1.23753122 2.69341672 0.00686313

N -1.37588598 1.37584091 -0.27036672

C -0.02840663 3.25492450 0.59037536

C 1.20821684 2.71355203 0.05616013

C 2.38597175 3.44694653 -0.31649451

C 3.25015962 2.55071615 -0.90000104

C 2.60325394 1.27133039 -0.86798027

N 1.34560882 1.41103601 -0.29058220

C -0.06335762 4.27456480 1.55260910

C -4.52545609 -0.06525237 -1.80430323

C 0.06707104 -4.27342437 1.55795145

C 4.52949862 0.06429361 -1.81004033

C -4.72901889 -0.72601043 -3.02946186

C -5.97943805 -0.74310059 -3.61233188

N -7.03265132 -0.13059445 -3.01800441

C -6.87465518 0.50254798 -1.83246930

C -5.64097221 0.54827472 -1.21098714

C 1.11898220 4.82262862 2.18374593

C 1.05268501 5.82874664 3.09883523

N -0.14082735 6.36857768 3.51106713

C -1.29835612 5.81948948 3.01549086

C -1.29137080 4.81236377 2.09956034

C 5.64691583 -0.55629548 -1.22603742

C 6.87874225 -0.50436820 -1.84925222

N 7.03526585 0.14260080 -3.02783994

C 5.97957371 0.75813638 -3.61611612

C 4.73113338 0.73559032 -3.03086836

C 1.28964657 -4.82022030 2.10831795

C 1.28599531 -5.82857658 3.02321841

N 0.12335050 -6.36948443 3.51497876

C -1.06536987 -5.81984664 3.10033838

C -1.12100706 -4.81286988 2.18592395

C -0.17724249 7.53574580 4.39338432

C -8.36259569 -0.19257239 -3.66711833

C 0.14674291 -7.53872383 4.39486575

C 8.35133051 0.17182530 -3.70421253

Co 0.00543151 0.00027084 -0.06749852

H 4.17739725 -2.71329681 -1.53521620

H 2.51337577 -4.49562699 -0.41938105

H -2.50967775 -4.51456422 -0.21141628

H -4.22269501 -2.75925409 -1.29629896

H -4.16559669 2.70916515 -1.54284285

H -2.49852374 4.49478983 -0.43591976

H 2.52606711 4.51317806 -0.20445096

H 4.23558901 2.75780483 -1.29443726

H -3.90583922 -1.21804810 -3.53240775

H -6.17767161 -1.23101499 -4.55815822

H -7.75798495 0.95669810 -1.40278244

H -5.55268675 1.05435420 -0.25808078

H 2.09652859 4.41740735 1.95958053

H 1.94083087 6.24262230 3.56229005

H -2.22046129 6.22818168 3.41219708

H -2.24745100 4.40231905 1.80314230

H 5.56092572 -1.06812240 -0.27604862

H 7.76299454 -0.96307097 -1.42672057

H 6.17582943 1.25120977 -4.55979975

H 3.90675075 1.23319505 -3.52629543

H 2.24957869 -4.41640609 1.81551623

H 2.20374136 -6.24471324 3.42249366

H -1.95803461 -6.22700029 3.56097809

H -2.09476401 -4.40031975 1.95900664

H -0.16168991 8.46413923 3.81214618

H -1.08622865 7.50682291 4.99761908

H 0.68772314 7.51531244 5.05941711

H -8.76185563 -1.20287805 -3.56141101

H -9.02713270 0.52275204 -3.18595510

H -8.25306753 0.06039862 -4.72204772

H 1.05922290 -7.52450345 4.99436219

H -0.71430021 -7.50679200 5.06549768

H 0.11373260 -8.46570022 3.81206492

H 8.59771675 1.20374050 -3.95691622

H 9.10674242 -0.23238395 -3.03253086

H 8.29759679 -0.43457544 -4.61011479

C 0.01730936 0.00419539 1.91746438

O -1.11586881 -0.04463780 2.41566295

O 1.15650418 0.05490826 2.40343870

[CoP(CO_2_)]^+1^ M = 3

C -2.67609167 1.22201264 -0.77760479

C -3.23372788 2.52722705 -0.97398637

C -2.37574041 3.42923021 -0.38220027

C -1.26160749 2.68124364 0.10550086

N -1.43522014 1.35421997 -0.17256028

C -3.27753221 -0.03535212 -1.09041252

C -0.03297840 3.23426935 0.63365600

C 1.18568191 2.69240969 0.09574676

C 2.37749384 3.42430999 -0.25637447

C 3.20606794 2.54791300 -0.90599861

C 2.53668670 1.27410597 -0.91835599

N 1.29472276 1.40506636 -0.32288280

C 3.10997495 0.04512767 -1.29053422

C 2.54617144 -1.21002337 -0.99946000

C 3.14696175 -2.49939301 -1.20104859

C 2.33801435 -3.41449399 -0.57481638

C 1.21609734 -2.68786704 -0.04319455

N 1.33377580 -1.37005216 -0.34583628

C 0.04840286 -3.23864188 0.60489116

C -1.22122550 -2.69502946 0.19737962

C -2.44818324 -3.40675267 0.01768565

C -3.34171962 -2.52540600 -0.55053692

C -2.66299776 -1.27264463 -0.70111609

N -1.37102561 -1.40600132 -0.24840788

C 0.15939616 -4.27389092 1.58025384

C 4.43834975 0.08859380 -1.94397462

C -0.04007498 4.29498249 1.58437298

C -4.56473979 -0.09913128 -1.74572610

C 4.62862061 0.81048012 -3.13772941

C 5.86869786 0.84856093 -3.73980330

N 6.92755086 0.19916345 -3.19534508

C 6.78236773 -0.49641062 -2.04314525

C 5.55910846 -0.56657022 -1.40519765

C -0.96237433 -4.74801440 2.34352371

C -0.83960986 -5.74194844 3.27326110

N 0.36469165 -6.32467372 3.55403654

C 1.47964391 -5.84449584 2.92366241

C 1.41125130 -4.84812080 1.99088305

C -5.66665739 0.76446112 -1.44572288

C -6.86417687 0.65734978 -2.09724769

N -7.06884221 -0.27425806 -3.08377187

C -6.04069046 -1.13007342 -3.40325162

C -4.83748236 -1.07523670 -2.75764493

C -1.25029250 4.86135165 2.11398187

C -1.23460217 5.88376572 3.02021809

N -0.06660413 6.40348374 3.50534627

C 1.11166800 5.83389360 3.10731961

C 1.15094125 4.81100657 2.20228285

C 0.45886725 -7.45883477 4.48135900

C 8.24690393 0.28988780 -3.86056365

C -0.07188688 7.56728456 4.40006428

C -8.32130936 -0.30655215 -3.84417364

Co -0.03646262 -0.00575869 -0.07305534

H -4.13893517 2.76561426 -1.51100462

H -2.46647768 4.50693906 -0.37301356

H 2.53891996 4.48266741 -0.10994671

H 4.18198961 2.76014023 -1.31946963

H 4.05424755 -2.70450320 -1.75123495

H 2.46183536 -4.48818706 -0.55613938

H -2.61094435 -4.45746796 0.21270586

H -4.36971712 -2.72802454 -0.81480890

H 3.80173989 1.33339739 -3.60217195

H 6.05532502 1.38287032 -4.66274917

H 7.66887839 -0.97808296 -1.65177508

H 5.48432913 -1.11899506 -0.47717208

H -1.93733128 -4.29781885 2.22571440

H -1.68706422 -6.10377327 3.84258527

H 2.42121485 -6.29092874 3.21953392

H 2.34635847 -4.48716833 1.58795058

H -5.59494859 1.48103336 -0.63818134

H -7.71466267 1.28077661 -1.84964896

H -6.24025539 -1.83242552 -4.20314793

H -4.06212635 -1.76775918 -3.06244858

H -2.21474820 4.47233271 1.81999084

H -2.14631959 6.32269352 3.40678452

H 2.00939423 6.23087060 3.56563686

H 2.11716234 4.37472483 1.99301231

H 0.40396019 -8.40245399 3.93026792

H 1.40576366 -7.40744817 5.02139898

H -0.36262514 -7.40841569 5.19722649

H 8.63769418 1.30145200 -3.73641093

H 8.92574739 -0.42921619 -3.40549907

H 8.12558901 0.06135513 -4.91985764

H -1.00888746 7.58894229 4.95810478

H 0.75916380 7.48804808 5.10281856

H 0.02795253 8.48971799 3.82011515

H -8.57559702 -1.34117693 -4.08269117

H -9.12133424 0.12445833 -3.24050069

H -8.22043275 0.26555734 -4.77236163

C 0.08627313 -0.05191048 1.91613790

O 1.24349379 0.07963212 2.33750651

O -1.00962240 -0.20785652 2.47038897

[CoP(CO_2_)]^0^ M = 2

C 2.69308577 -1.18848874 -0.83616945

C 3.25596240 -2.47429013 -1.07834124

C 2.40749077 -3.40727539 -0.49761686

C 1.31439568 -2.68597082 0.04675449

N 1.47027927 -1.35404015 -0.18617226

C 3.26085781 0.08844733 -1.13510579

C 0.10060935 -3.25204099 0.64061128

C -1.13551281 -2.75272625 0.06731614

C -2.30024145 -3.50547575 -0.29793671

C -3.15725266 -2.63551988 -0.93571430

C -2.51815533 -1.35289919 -0.93855980

N -1.27420039 -1.46360724 -0.33045538

C -3.11418672 -0.12621771 -1.29559597

C -2.58173461 1.13704133 -0.98722452

C -3.20159001 2.41737323 -1.18008149

C -2.40623657 3.34244629 -0.54582388

C -1.27714763 2.63238582 -0.01381559

N -1.37306387 1.31612934 -0.31924549

C -0.11966231 3.19846387 0.65430158

C 1.16628274 2.69456860 0.18880872

C 2.35028849 3.44628837 -0.05499769

C 3.26348173 2.58130049 -0.63265691

C 2.63092043 1.30760298 -0.73861204

N 1.34372994 1.40757662 -0.22580825

C -0.24326737 4.19123623 1.63704950

C -4.43072393 -0.18603172 -1.96531567

C 0.14215481 -4.23515556 1.62840859

C 4.54337126 0.17734882 -1.80559220

C -4.60120072 -0.91763594 -3.15782590

C -5.82800276 -0.95843392 -3.78460038

N -6.89913479 -0.30554748 -3.26549781

C -6.77646251 0.39275647 -2.11113691

C -5.56674184 0.46645725 -1.45048385

C 0.88327471 4.73425598 2.36934235

C 0.74151239 5.72146820 3.29538031

N -0.48386103 6.25020786 3.62388926

C -1.59697640 5.70088549 3.03321860

C -1.51388860 4.70814457 2.10562508

C 5.66704039 -0.65817953 -1.50091568

C 6.86117209 -0.52835162 -2.15230011

N 7.04485909 0.40306535 -3.14969821

C 5.98875222 1.22728545 -3.48004006

C 4.78934026 1.14686516 -2.83153690

C 1.38056079 -4.75124065 2.18583230

C 1.40018125 -5.71941334 3.13901831

N 0.24711509 -6.25894758 3.66768798

C -0.95637860 -5.74793531 3.23258680

C -1.03601072 -4.78066325 2.28112060

C -0.59391608 7.40809265 4.51126239

C -8.20525679 -0.39940995 -3.95310258

C 0.29689584 -7.39960653 4.57807657

C 8.28924557 0.45174918 -3.91699690

Co 0.03452788 -0.02379939 -0.04928308

H 4.16148731 -2.68628935 -1.62762337

H 2.51439538 -4.48466224 -0.49691485

H -2.43872720 -4.56719825 -0.14486736

H -4.13404867 -2.86230214 -1.34113208

H -4.11135588 2.61340135 -1.73012072

H -2.55079992 4.41344572 -0.50419604

H 2.48017480 4.50462413 0.12637000

H 4.27647698 2.81567168 -0.92872037

H -3.76458201 -1.44179016 -3.60280602

H -5.99563857 -1.49490488 -4.70993026

H -7.67157503 0.87354120 -1.73836436

H -5.51129158 1.02073038 -0.52242785

H 1.87654734 4.33761762 2.20908238

H 1.58889341 6.13034994 3.83366387

H -2.54928417 6.09664360 3.36724027

H -2.44273745 4.29481065 1.73599599

H 5.60736138 -1.37359723 -0.69078159

H 7.72779964 -1.12741634 -1.89986765

H 6.16936338 1.92637786 -4.28741893

H 3.99505603 1.81612437 -3.13986542

H 2.32913746 -4.35068468 1.85173050

H 2.32712928 -6.10763759 3.54601358

H -1.83833945 -6.15217219 3.71679211

H -2.01792441 -4.39307196 2.04271017

H -0.55269286 8.34302884 3.94150515

H -1.53998758 7.36204741 5.05485222

H 0.22611238 7.39290264 5.23218969

H -8.62795911 -1.39156160 -3.78262068

H -8.87345193 0.36263298 -3.55536326

H -8.05683040 -0.23384729 -5.02060379

H 1.20957904 -7.34953603 5.17651038

H -0.56282205 -7.36734162 5.25142641

H 0.28310134 -8.34840128 4.02835324

H 8.51851672 1.48751311 -4.17695878

H 9.10435699 0.05283308 -3.31059420

H 8.20255506 -0.13912370 -4.83588033

C -0.09495924 -0.01739844 1.91741163

O 0.99756046 0.08283553 2.49944752

O -1.25753411 -0.11695941 2.34439242

[CoP(CO_2_)]^0^ M = 4

C 2.60586439 -1.22570509 -0.90296294

C 3.14843666 -2.52841809 -1.14636148

C 2.32363960 -3.43685144 -0.51182294

C 1.24421417 -2.69303143 0.04936910

N 1.40258009 -1.36629419 -0.22817627

C 3.19148463 0.04041580 -1.21475993

C 0.05447247 -3.23303525 0.67920977

C -1.20243312 -2.70308107 0.21286452

C -2.42304028 -3.41508795 0.00047100

C -3.28853408 -2.54475570 -0.62938732

C -2.59897048 -1.29893308 -0.78209560

N -1.32791111 -1.42697911 -0.26855297

C -3.19016748 -0.06740554 -1.22097124

C -2.59922688 1.19444200 -0.89993369

C -3.13127091 2.49941155 -1.15097992

C -2.31006332 3.40396871 -0.50741798

C -1.24265767 2.65479669 0.06944918

N -1.40342886 1.32833261 -0.20940165

C -0.05607299 3.19279583 0.70623014

C 1.20090551 2.66220519 0.24556537

C 2.42309427 3.37499238 0.04113156

C 3.28798302 2.51039201 -0.59525582

C 2.59754502 1.26655200 -0.76120845

N 1.32650945 1.38943585 -0.24809847

C -0.14422280 4.22836908 1.67512884

C -4.44799855 -0.13571121 -1.92917489

C 0.13899730 -4.25039580 1.66288838

C 4.44761202 0.12324003 -1.92429453

C -4.69199779 -1.13472376 -2.92817716

C -5.86298094 -1.18622678 -3.62937207

N -6.88544722 -0.29422239 -3.39336790

C -6.71733035 0.64723939 -2.40712643

C -5.55055808 0.75093149 -1.70274876

C 0.99090860 4.71574626 2.41663492

C 0.88305877 5.72683391 3.32682443

N -0.31859571 6.32069322 3.61418075

C -1.44550299 5.82583417 3.00937130

C -1.39182827 4.81294072 2.09643098

C 5.56010977 -0.75324594 -1.70461463

C 6.73075071 -0.62141423 -2.39779865

N 6.89958358 0.34888030 -3.35540371

C 5.85894621 1.21626586 -3.60068600

C 4.68303812 1.13659609 -2.91023005

C 1.38739966 -4.81875729 2.11042252

C 1.43997117 -5.80101781 3.05454542

N 0.31065217 -6.28471989 3.66823268

C -0.89400246 -5.70930780 3.34858498

C -1.00091230 -4.72931232 2.40666083

C -0.39198060 7.48481792 4.50222747

C -8.15625785 -0.42403923 -4.10958600

C 0.38539291 -7.41490090 4.59593086

C 8.11991564 0.40793883 -4.16279048

Co 0.00024493 -0.01970433 -0.03410022

H 4.02270683 -2.76176359 -1.73498161

H 2.41630843 -4.51484690 -0.51006388

H -2.59917340 -4.45934389 0.21883413

H -4.30556235 -2.74997990 -0.93254753

H -3.99635623 2.73766626 -1.75100445

H -2.39641697 4.48225322 -0.51033344

H 2.60021253 4.41731662 0.26628470

H 4.30493905 2.71925560 -0.89572360

H -3.91357344 -1.84469339 -3.18100807

H -6.03831683 -1.90812801 -4.41785117

H -7.56881155 1.28761971 -2.21209175

H -5.50284846 1.48994925 -0.91392404

H 1.96302861 4.25971415 2.29377755

H 1.73813646 6.09817543 3.87883867

H -2.38183930 6.27968961 3.31126495

H -2.33020132 4.44637190 1.70428263

H 5.51951045 -1.49994190 -0.92259140

H 7.58990117 -1.25341056 -2.20786819

H 6.02772341 1.94996531 -4.37930219

H 3.89825350 1.84182631 -3.15635891

H 2.32649019 -4.45669238 1.71531964

H 2.37697849 -6.23699756 3.38030887

H -1.75119912 -6.06781735 3.90609155

H -1.97508544 -4.28425656 2.26062604

H -0.30322856 8.41142017 3.92611054

H -1.34683476 7.47829637 5.03104446

H 0.41780539 7.43494973 5.23209958

H -8.76816437 -1.21774067 -3.66774744

H -8.69898213 0.52062486 -4.05704972

H -7.96133391 -0.66076250 -5.15812310

H 1.33264804 -7.37877601 5.13774727

H -0.43414053 -7.34941173 5.31397993

H 0.31516772 -8.36450486 4.05472515

H 8.35173341 1.44889462 -4.39668182

H 8.94910965 -0.01953781 -3.59655128

H 7.99286993 -0.15261614 -5.09522502

C 0.00874927 -0.04080933 1.94766134

O -1.11276604 -0.21538597 2.44975067

O 1.13324516 0.11977304 2.44742674

[CoP(CO_2_)]^0^ M = 6

C 2.90834474 -0.49751271 -0.83053062

C 3.64953547 -1.60397154 -1.35569325

C 3.05323856 -2.75623689 -0.87570977

C 1.92622174 -2.34278704 -0.10538668

N 1.83589100 -0.98352341 -0.10008796

C 3.19310448 0.89481080 -1.02130803

C 0.91716643 -3.21844176 0.48688056

C -0.46544165 -2.99769901 0.13363066

C -1.49729000 -3.98619931 0.02508415

C -2.61926446 -3.34592134 -0.45661439

C -2.27041960 -1.96866055 -0.64338600

N -0.95524243 -1.78369911 -0.28477985

C -3.17142739 -0.92195489 -1.04794182

C -2.88110480 0.47018702 -0.86473627

C -3.62672742 1.56996101 -1.40025008

C -3.03857181 2.72772309 -0.92727826

C -1.91121961 2.32526347 -0.14954690

N -1.81077954 0.96648536 -0.13545040

C -0.91535047 3.21722371 0.43622019

C 0.47223055 2.99179768 0.10666072

C 1.50376382 3.97513442 -0.01567814

C 2.63199281 3.32234406 -0.47017524

C 2.28733987 1.94272025 -0.63152571

N 0.96475756 1.76787697 -0.27586470

C -1.33586969 4.28216816 1.27403315

C -4.43291164 -1.30315231 -1.64041392

C 1.32539962 -4.27598709 1.34610811

C 4.45859329 1.26884448 -1.61061350

C -4.54946380 -2.39235838 -2.56678255

C -5.74342242 -2.73611688 -3.13344429

N -6.90700811 -2.06636412 -2.82960024

C -6.85023387 -1.03424924 -1.92129370

C -5.67125623 -0.63980344 -1.35458202

C -0.43237801 5.08846895 2.05974095

C -0.86725521 6.10709823 2.85482378

N -2.19854474 6.41708888 2.97992795

C -3.10840357 5.61865154 2.33109519

C -2.72395016 4.58184270 1.53351915

C 5.69136124 0.59997602 -1.31100934

C 6.87421016 0.97200877 -1.88340945

N 6.94453460 2.00061700 -2.79603424

C 5.78386962 2.66344647 -3.12598803

C 4.58609704 2.34167196 -2.55393066

C 2.70842851 -4.59192971 1.60393447

C 3.07915401 -5.62891322 2.40947890

N 2.15955138 -6.40627572 3.06603224

C 0.83424220 -6.08008919 2.94193438

C 0.41174593 -5.06075121 2.13886524

C -2.63395315 7.58901481 3.74123761

C -8.19121687 -2.52005641 -3.36708271

C 2.58064664 -7.57974316 3.83685758

C 8.19884758 2.29884537 -3.48889060

Co 0.02509426 -0.01143631 0.26447174

H 4.49188128 -1.54860160 -2.03090976

H 3.32737464 -3.77931025 -1.09954361

H -1.39469734 -5.04630296 0.20857117

H -3.58466557 -3.79454801 -0.64419603

H -4.46519170 1.50333856 -2.07928522

H -3.31553599 3.74817553 -1.16002323

H 1.40024135 5.03951752 0.14159291

H 3.59909148 3.76683044 -0.65931313

H -3.66450010 -2.93644626 -2.87337996

H -5.83262856 -3.53210437 -3.86276755

H -7.79687275 -0.56859148 -1.67543447

H -5.69881153 0.16576311 -0.63138360

H 0.62417588 4.86366643 2.07736672

H -0.18513782 6.70647164 3.44642748

H -4.15160137 5.85390872 2.50650521

H -3.50515021 3.97173983 1.10173981

H 5.71015475 -0.19201647 -0.57269226

H 7.81515357 0.49670353 -1.63402896

H 5.87968308 3.44052940 -3.87457356

H 3.70689511 2.89021735 -2.86922680

H 3.49766278 -4.00046071 1.16126148

H 4.11887079 -5.88169047 2.58021691

H 0.14421740 -6.66804341 3.53549707

H -0.64302800 -4.82680485 2.15155934

H -2.70966830 8.46681980 3.09054727

H -3.60884996 7.38984184 4.19082061

H -1.91445201 7.79241806 4.53653594

H -8.63690641 -3.27940351 -2.71529164

H -8.87156365 -1.67034750 -3.44802489

H -8.03853234 -2.94513817 -4.36102098

H 3.55688165 -7.38838316 4.28622603

H 1.85667907 -7.76968605 4.63090386

H 2.64637978 -8.45972750 3.18901094

H 8.22364498 3.35671942 -3.75708638

H 9.03939091 2.08341088 -2.82615003

H 8.29296568 1.69357765 -4.39724604

C -0.07737754 0.39592258 2.28073016

O -0.08452025 1.47014562 2.86172830

O -0.08368612 -0.81440938 2.55445236

[CoP(CO_2_)]^–1^ M = 1

C 2.62708336 -1.15755213 -0.87452754

C 3.17209976 -2.44804011 -1.13878843

C 2.35905060 -3.37358433 -0.49868239

C 1.29948064 -2.64526568 0.10157629

N 1.44458245 -1.31672643 -0.15185638

C 3.18045063 0.11772051 -1.20599309

C 0.11705317 -3.19487451 0.77027050

C -1.15054568 -2.71031431 0.23837516

C -2.33010444 -3.45838473 -0.03336780

C -3.21072162 -2.60051994 -0.67396545

C -2.56149472 -1.33560796 -0.78435314

N -1.29987140 -1.43402701 -0.21504802

C -3.16554297 -0.11770767 -1.23077254

C -2.61432504 1.15949267 -0.90307087

C -3.15846222 2.44855417 -1.17646071

C -2.34968888 3.37741509 -0.53583400

C -1.29339520 2.65243987 0.07433168

N -1.43617425 1.32277413 -0.17470857

C -0.11759797 3.20494363 0.75212084

C 1.15503389 2.71742944 0.23560883

C 2.33693984 3.46406662 -0.02999024

C 3.22244940 2.60305841 -0.65951647

C 2.57390608 1.33769367 -0.76880642

N 1.30807562 1.43886996 -0.20962377

C -0.22304037 4.16886139 1.74794064

C -4.40173739 -0.21186271 -1.98080501

C 0.21349939 -4.15519528 1.77040129

C 4.42067863 0.20745077 -1.94964908

C -4.58680145 -1.20070794 -3.00284635

C -5.73266700 -1.27525428 -3.74138033

N -6.79032257 -0.41384326 -3.52481699

C -6.67890456 0.51516948 -2.51340561

C -5.53688197 0.63961397 -1.77410792

C 0.91431220 4.70201226 2.48525759

C 0.78078514 5.66925834 3.42823186

N -0.44555922 6.19729149 3.78072066

C -1.56718720 5.66568248 3.17400045

C -1.49338916 4.69453268 2.22840934

C 5.55344536 -0.64553735 -1.73374153

C 6.69880590 -0.52729155 -2.46829023

N 6.82567516 0.41236635 -3.46839453

C 5.76265267 1.26026571 -3.70812632

C 4.61268698 1.19115985 -2.97497532

C 1.47989019 -4.67818897 2.26418501

C 1.54591760 -5.64736600 3.21230336

N 0.41920536 -6.17902518 3.80953541

C -0.80432678 -5.65270656 3.44480200

C -0.93010573 -4.68753604 2.49862441

C -0.54072787 7.36506778 4.64919798

C -8.04121554 -0.57924822 -4.26403517

C 0.50740302 -7.34588756 4.67995068

C 8.00127891 0.42926980 -4.33785888

Co 0.00212488 0.00344759 0.03655562

H 4.04176801 -2.66831034 -1.74025316

H 2.46500026 -4.45112071 -0.49294088

H -2.47717865 -4.50990421 0.17544129

H -4.21220339 -2.83207278 -1.01021218

H -4.02481327 2.66558267 -1.78386874

H -2.45630249 4.45489664 -0.53550028

H 2.48241023 4.51659749 0.17486938

H 4.22655387 2.83281775 -0.98913697

H -3.78071557 -1.88522097 -3.23888419

H -5.86293386 -1.98932654 -4.54548425

H -7.55486069 1.12698396 -2.33443476

H -5.52945236 1.36936482 -0.97476410

H 1.90478683 4.30228467 2.30974008

H 1.63161208 6.06383963 3.97305863

H -2.51545180 6.06439504 3.51775943

H -2.42157569 4.30113757 1.83296555

H 5.54180230 -1.36856482 -0.92829829

H 7.57126071 -1.14352249 -2.28670321

H 5.89614508 1.96787626 -4.51728884

H 3.81073886 1.87879399 -3.21592647

H 2.41128525 -4.28449654 1.87665422

H 2.49135637 -6.04438404 3.56566923

H -1.65973462 -6.04689085 3.98271125

H -1.91929961 -4.28906896 2.31322035

H -0.48334795 8.29903205 4.07603830

H -1.48944298 7.34172276 5.19132821

H 0.27517700 7.34725892 5.37617396

H -8.66597396 -1.35457032 -3.80588620

H -8.58780174 0.36531155 -4.26874892

H -7.82055104 -0.86144631 -5.29615184

H 1.45145119 -7.32161023 5.23014027

H -0.31469167 -7.32777613 5.39991301

H 0.45536202 -8.28045049 4.10723982

H 8.19995964 1.45258078 -4.66330405

H 8.86775392 0.06194809 -3.78423727

H 7.84404966 -0.20369839 -5.21875824

C -0.01547815 0.01151525 2.00047147

O 1.09806223 0.19575113 2.52618611

O -1.13783664 -0.16789125 2.50877892

[CoP(CO_2_)]^–1^ M = 3

C 2.61723534 -1.19159536 -0.97729841

C 3.16540542 -2.47367514 -1.26454461

C 2.34852285 -3.41456408 -0.64462419

C 1.29074520 -2.69927212 -0.03165854

N 1.43423149 -1.36610078 -0.26258252

C 3.17492786 0.09562027 -1.26778169

C 0.11793853 -3.24928536 0.65722221

C -1.15714193 -2.76734078 0.13683143

C -2.33840400 -3.51059050 -0.12747586

C -3.23403138 -2.63860183 -0.73320724

C -2.58678677 -1.37352271 -0.83062730

N -1.31423241 -1.48265954 -0.28948719

C -3.19673352 -0.14114004 -1.23550989

C -2.63133166 1.12276268 -0.89860386

C -3.17273994 2.41986490 -1.15171414

C -2.35895939 3.33490206 -0.50564065

C -1.29737329 2.59714685 0.08794049

N -1.44393264 1.27247123 -0.17967341

C -0.11807369 3.14497460 0.75715968

C 1.14895335 2.65802392 0.23598952

C 2.33624050 3.40872808 -0.01079030

C 3.21757960 2.56435757 -0.65967760

C 2.56661341 1.30018881 -0.80265027

N 1.29927784 1.39028995 -0.24230931

C -0.21924931 4.12597792 1.74512361

C -4.45650933 -0.20877814 -1.94876481

C 0.22802413 -4.17173430 1.68605601

C 4.42996626 0.20682531 -1.98354351

C -4.68491017 -1.17063684 -2.98782000

C -5.85259807 -1.21128141 -3.69418783

N -6.89173349 -0.34087907 -3.42633247

C -6.73961466 0.55887928 -2.39267107

C -5.57546735 0.64827762 -1.68428263

C 0.91785797 4.66152032 2.47464161

C 0.78975834 5.65425713 3.39430079

N -0.43075212 6.19996929 3.72664481

C -1.55369601 5.65841294 3.13903662

C -1.48487341 4.66213731 2.21687464

C 5.56304985 -0.64365758 -1.75927244

C 6.73552635 -0.48136858 -2.44085370

N 6.88980733 0.49920180 -3.39743672

C 5.82176951 1.33629901 -3.65447573

C 4.64447739 1.22298552 -2.97255118

C 1.50360532 -4.66922160 2.18868447

C 1.58563697 -5.57430672 3.19543105

N 0.46775632 -6.07029346 3.84340462

C -0.76552277 -5.58200239 3.45053990

C -0.90764537 -4.68103522 2.44671191

C -0.52173496 7.38035777 4.58313109

C -8.16346836 -0.46704211 -4.13571134

C 0.57350808 -7.17596417 4.78590810

C 8.10861787 0.58429587 -4.19986251

Co -0.00216342 -0.05156300 -0.02209081

H 4.04164434 -2.67896989 -1.86229633

H 2.45959917 -4.49203165 -0.65132964

H -2.48132277 -4.56519893 0.07110968

H -4.24384819 -2.86146922 -1.05044105

H -4.04198062 2.64772492 -1.75097120

H -2.46004099 4.41246810 -0.49323343

H 2.48483420 4.45552742 0.21719226

H 4.22169860 2.80303073 -0.98180717

H -3.89491998 -1.85926147 -3.26330196

H -6.01614594 -1.90241575 -4.51236195

H -7.60165986 1.17664723 -2.17209421

H -5.53468329 1.35627668 -0.86653295

H 1.90598163 4.25033149 2.31548131

H 1.64268582 6.05610338 3.92980539

H -2.49989852 6.06997000 3.47223070

H -2.41665159 4.26376856 1.83618823

H 5.52946011 -1.39706673 -0.98276521

H 7.60989384 -1.09062302 -2.24521698

H 5.97637355 2.07441531 -4.43202592

H 3.84149702 1.90623736 -3.22259173

H 2.42738472 -4.29725064 1.76240256

H 2.53718956 -5.94297424 3.56344431

H -1.61307455 -5.94902025 4.01959220

H -1.90225929 -4.30593012 2.24080356

H -0.46254221 8.30306825 3.99408968

H -1.46971930 7.36572153 5.12587402

H 0.29571641 7.36899124 5.30769297

H -8.78374956 -1.25403732 -3.69100076

H -8.70115701 0.48147183 -4.08844924

H -7.97443511 -0.71014668 -5.18415140

H 1.52150958 -7.10760484 5.32600809

H -0.24200294 -7.11785772 5.51164134

H 0.52554958 -8.14915019 4.27938843

H 8.31291215 1.62887536 -4.44488935

H 8.94896811 0.18816704 -3.62634277

H 8.00657565 0.01124978 -5.12885254

C 0.02977673 -0.11268049 1.94337436

O 1.15916909 0.02532433 2.44728944

O -1.08574039 -0.28553844 2.46757001

[CoP(CO_2_)]^–1^ M = 5

C 2.62499076 -1.29326564 -0.77173456

C 3.13301963 -2.61400964 -0.98504301

C 2.25360277 -3.48959260 -0.38115483

C 1.17550688 -2.70699425 0.13135287

N 1.39012991 -1.38646802 -0.14049403

C 3.26766400 -0.05845896 -1.08975471

C -0.06700602 -3.22767749 0.66746659

C -1.26965694 -2.65029343 0.13761384

C -2.48322100 -3.32627304 -0.19737509

C -3.28780706 -2.41780127 -0.85576110

C -2.58005581 -1.16836956 -0.87979786

N -1.33606902 -1.34213591 -0.30688230

C -3.11937934 0.07109880 -1.31036160

C -2.49071903 1.30521489 -1.01220947

C -3.05954137 2.61010394 -1.19415938

C -2.22138789 3.50798647 -0.55758424

C -1.12014821 2.76359772 -0.04140009

N -1.27478948 1.43167329 -0.36391091

C 0.06533612 3.27658869 0.60188931

C 1.31524244 2.68261038 0.18564066

C 2.55955442 3.35269031 -0.02942499

C 3.41862569 2.43329289 -0.59423472

C 2.70119334 1.20013707 -0.70858894

N 1.41826355 1.38332940 -0.23417617

C 0.00961728 4.32546176 1.56124737

C -4.45261366 0.07050917 -1.96408571

C -0.06788786 -4.29686265 1.60795832

C 4.55267868 -0.04698802 -1.75800645

C -4.68989046 -0.59847945 -3.20259125

C -5.92706622 -0.64458678 -3.78324918

N -7.02861073 0.00821400 -3.20789557

C -6.83875044 0.61944050 -1.95902482

C -5.60753455 0.66542040 -1.36802077

C 1.16000154 4.80677347 2.29060632

C 1.08051916 5.83062895 3.18635476

N -0.11258749 6.45240430 3.48291090

C -1.25733338 5.95383419 2.90147984

C -1.22601013 4.93272938 2.00019401

C 5.62998182 -0.94455183 -1.45473192

C 6.82274485 -0.89413743 -2.11793695

N 7.05500415 0.01653537 -3.12642647

C 6.04792887 0.90401289 -3.45085487

C 4.85263374 0.90188526 -2.79120824

C 1.13585316 -4.90182755 2.12747054

C 1.10761725 -5.94599870 3.00253387

N -0.07591576 -6.46673738 3.48240735

C -1.24876705 -5.84782774 3.10773436

C -1.26839575 -4.80599112 2.23006964

C -0.15271562 7.64156631 4.33109242

C -8.37365881 -0.34402313 -3.63495599

C -0.08804338 -7.68062729 4.29503081

C 8.28261983 -0.03818540 -3.91821523

Co 0.02742456 0.02722272 -0.03905375

H 4.02329834 -2.87867522 -1.53557628

H 2.30879874 -4.57006527 -0.37324891

H -2.68036123 -4.37964971 -0.04895777

H -4.27044203 -2.58582490 -1.27491671

H -3.98279854 2.82375100 -1.71475784

H -2.32317099 4.58501118 -0.52379433

H 2.75704389 4.40127810 0.14494962

H 4.44781280 2.59896127 -0.88002511

H -3.86621527 -1.09614075 -3.70725353

H -6.12220543 -1.14764784 -4.72321934

H -7.72319304 1.06717219 -1.52041871

H -5.51321558 1.17655363 -0.41438242

H 2.12307455 4.33106304 2.16871325

H 1.94654644 6.19150958 3.72900216

H -2.18573094 6.41171681 3.22273136

H -2.17384579 4.56205833 1.63486126

H 5.53762674 -1.64625896 -0.63560829

H 7.65356692 -1.54304893 -1.86785084

H 6.26351120 1.58584280 -4.26443429

H 4.09888541 1.61758612 -3.09755926

H 2.10645731 -4.51713860 1.84499680

H 2.01220969 -6.40593725 3.38310093

H -2.15068778 -6.22824202 3.57334301

H -2.22281459 -4.33569076 2.03658554

H -0.03109674 8.55342793 3.73511139

H -1.10996549 7.68348986 4.85546247

H 0.64997753 7.58917856 5.06998290

H -8.72746106 -1.27383956 -3.16338869

H -9.06447551 0.46294760 -3.37433706

H -8.39124855 -0.47593596 -4.72057102

H 0.81586727 -7.71934962 4.90717616

H -0.95803250 -7.66865983 4.95518142

H -0.13063870 -8.57588899 3.66375727

H 8.54054522 0.96605377 -4.26112640

H 9.09812111 -0.41797698 -3.29925537

H 8.15785084 -0.69359971 -4.78801464

C -0.06672336 0.10855934 1.92431081

O 1.04404115 0.18906650 2.48121705

O -1.21605702 0.07541650 2.40204450

[CoP(CO_2_)]^–2^ M = 2

C -2.72626004 -1.17506895 -0.60168696

C -3.72014014 -0.76433631 -1.51389110

C -3.76732188 0.63559941 -1.46741323

C -2.79643042 1.04745284 -0.53426085

N -2.11595108 -0.05690670 -0.05237843

C -2.40136023 -2.55066594 -0.23042182

C -2.53760571 2.41934482 -0.10373080

C -1.17831714 2.81287980 0.20878556

C -0.75037181 3.95339498 0.93894884

C 0.63887624 3.99181667 0.85528039

C 1.03922048 2.86343369 0.09896519

N -0.07011496 2.11133767 -0.24152344

C 2.38420798 2.51353754 -0.28807387

C 2.73464944 1.14398782 -0.58715090

C 3.82288996 0.72428720 -1.40239521

C 3.87309253 -0.65989306 -1.32885439

C 2.81225464 -1.06784813 -0.47261209

N 2.08099923 0.03240302 -0.08388808

C 2.53640330 -2.42815244 -0.05153004

C 1.17742536 -2.81309814 0.28264204

C 0.76236675 -3.91062304 1.06868069

C -0.63449174 -3.95085854 1.00637461

C -1.04339016 -2.87369487 0.18815667

N 0.06395799 -2.13845235 -0.19965312

C 3.58912509 -3.37170114 -0.00194857

C 3.37800767 3.53029192 -0.37785011

C -3.60372040 3.33820176 -0.04516523

C -3.39090353 -3.54629119 -0.28925771

C 3.07735090 4.91595917 -0.67086035

C 4.04118696 5.87136123 -0.76823165

N 5.38175904 5.58699547 -0.57516302

C 5.72037953 4.28412556 -0.25556483

C 4.79180173 3.29197736 -0.18054125

C 3.40025400 -4.80693615 -0.12251013

C 4.43136261 -5.69115845 -0.08223562

N 5.74010757 -5.29455353 0.13764682

C 5.98096569 -3.93466339 0.25064978

C 4.98575529 -3.01231450 0.18038838

C -4.82485653 -3.27681778 -0.24804080

C -5.76257590 -4.25603648 -0.27337654

N -5.43503060 -5.60380192 -0.36013636

C -4.08648013 -5.91523482 -0.46733358

C -3.11169849 -4.97385377 -0.40360692

C -4.99711664 2.94070154 0.09437147

C -6.01416027 3.83571485 0.18218033

N -5.81233515 5.20419102 0.07623807

C -4.50718798 5.63984891 -0.08075953

C -3.45407973 4.78443828 -0.13468746

C 6.83978753 -6.24299156 0.02548528

C 6.37148102 6.65577580 -0.51149561

C -6.88861747 6.14326692 0.35750126

C -6.45495595 -6.59995126 -0.64944276

Co -0.04635191 -0.01306038 0.28411413

H -4.32776536 -1.41814877 -2.12749729

H -4.42288119 1.28918462 -2.03059811

H -1.38541401 4.65078533 1.46902875

H 1.29738076 4.72436686 1.30519334

H 4.47151394 1.37330799 -1.97684036

H 4.56895114 -1.31820231 -1.83325291

H 1.40649164 -4.58577132 1.61821800

H -1.28473093 -4.66056225 1.50232429

H 2.05463869 5.20783151 -0.87473103

H 3.81505025 6.89861593 -1.03130444

H 6.77202455 4.10879941 -0.05947997

H 5.13455811 2.29943671 0.08355288

H 2.40845824 -5.20017462 -0.30571883

H 4.28505499 -6.75568659 -0.22792045

H 7.01396936 -3.65864324 0.43220542

H 5.25404597 -1.97305094 0.32429595

H -5.16854524 -2.25631045 -0.13359345

H -6.82316667 -4.04249174 -0.19023502

H -3.86312282 -6.96743189 -0.60842243

H -2.08637158 -5.30880934 -0.49706146

H -5.24294097 1.88927195 0.17593518

H -7.04201488 3.52835209 0.34113044

H -4.38569557 6.71194917 -0.19419338

H -2.47589785 5.20931940 -0.31992294

H 7.18224342 -6.34744346 -1.01299807

H 7.67911958 -5.90462980 0.63887325

H 6.51864629 -7.22221072 0.38965286

H 6.44810682 7.07296529 0.50100384

H 7.34885348 6.26791786 -0.80847911

H 6.09093218 7.45548103 -1.20167863

H -6.97576267 6.35531706 1.43209133

H -6.70253719 7.08272380 -0.17011010

H -7.83731461 5.73092835 0.00378891

H -6.65724580 -6.68396678 -1.72702636

H -6.13105257 -7.57599266 -0.27797465

H -7.38496933 -6.32996443 -0.14109132

C 0.24308590 0.21890406 2.38949281

O 1.07891077 0.98917547 2.85302915

O -0.60456724 -0.56433464 2.84126057

[CoP(CO_2_)]^–2^_2 M = 2

C 2.85277547 -1.24152795 -0.36521191

C 3.53680294 -2.44524010 -0.12099501

C 2.60795630 -3.34211106 0.44947462

C 1.38344343 -2.65858207 0.54055659

N 1.54467009 -1.37944818 0.05073621

C 3.35828830 0.02192790 -0.92792981

C 0.06504067 -3.15520331 0.96049703

C -1.06229846 -2.67328870 0.15822130

C -2.18129552 -3.40428566 -0.33435533

C -2.93540774 -2.52629270 -1.09974774

C -2.26952219 -1.26416418 -1.07009576

N -1.12538754 -1.39229276 -0.29710334

C -2.77147665 -0.02154963 -1.57945747

C -2.24862638 1.23971344 -1.14952517

C -2.71494855 2.55073679 -1.47078407

C -1.98246155 3.44373993 -0.69960053

C -1.04149253 2.67820017 0.04183793

N -1.18531465 1.36179117 -0.25737358

C 0.03060188 3.17215480 0.91680858

C 1.36694757 2.66104411 0.59524366

C 2.60715690 3.32192644 0.60007182

C 3.54618193 2.43137967 0.03443784

C 2.85290482 1.25578884 -0.29958853

N 1.52981208 1.39791897 0.06378939

C -0.23071552 4.08382640 1.92863404

C -3.88201406 -0.07330121 -2.50904600

C -0.11279699 -4.03168199 2.02399379

C 4.24560766 0.05321408 -1.99404855

C -3.93698651 -1.04361922 -3.56542475

C -4.96256042 -1.08155770 -4.46496653

N -6.02173513 -0.19516513 -4.39452649

C -6.03866118 0.71879483 -3.36039934

C -5.01583199 0.80490290 -2.46002017

C 0.77752223 4.55898956 2.87270885

C 0.50282052 5.47267231 3.83581371

N -0.76114935 6.00968178 4.01604490

C -1.77430522 5.53697601 3.19673381

C -1.55798972 4.61708870 2.22447855

C 4.73930441 -1.13706598 -2.69117013

C 5.67297264 -1.07969855 -3.67055499

N 6.24948316 0.11388537 -4.08812746

C 5.68517172 1.28245360 -3.59017367

C 4.75104052 1.28277365 -2.60973773

C 0.97713258 -4.49801849 2.87624006

C 0.79181668 -5.39296819 3.87796605

N -0.45317395 -5.90974354 4.19442532

C -1.53926637 -5.42027704 3.48871388

C -1.41065855 -4.52096439 2.48151046

C -0.97533358 7.15662388 4.88707483

C -7.16553398 -0.33119637 -5.29281624

C -0.59180200 -7.04253416 5.09889927

C 7.11575176 0.14970915 -5.25654311

Co 0.16959003 -0.00340048 0.10213770

H 4.58498595 -2.63405832 -0.31955140

H 2.78296540 -4.37838237 0.71336643

H -2.37588583 -4.45555243 -0.16517155

H -3.86801313 -2.74237407 -1.60297069

H -3.48083183 2.80427516 -2.18937732

H -2.06652333 4.52347192 -0.68555917

H 2.78663161 4.34520401 0.90742786

H 4.60424403 2.61101877 -0.11552132

H -3.11998993 -1.74424326 -3.69068688

H -4.98949791 -1.78247659 -5.29058407

H -6.91789907 1.34915703 -3.30240673

H -5.10907442 1.52415488 -1.65645770

H 1.77715242 4.14497127 2.83389273

H 1.25571041 5.81520265 4.53822432

H -2.76176665 5.93546902 3.40507594

H -2.41521861 4.26415844 1.66407490

H 4.33634971 -2.10824646 -2.43393976

H 6.02415474 -1.96829358 -4.18513038

H 6.04699414 2.20032213 -4.04217877

H 4.35966358 2.23850982 -2.28548350

H 1.97261487 -4.09894937 2.72950445

H 1.60848238 -5.73411969 4.50577473

H -2.50763605 -5.78299445 3.81765393

H -2.31867710 -4.14270290 2.02969332

H -0.82352489 8.10736042 4.35814908

H -1.99581090 7.13424748 5.27965273

H -0.28060927 7.11108090 5.73016782

H -7.89765517 -1.04338182 -4.89353036

H -7.64619768 0.64086747 -5.42118312

H -6.82236765 -0.68314469 -6.26845243

H 0.18505714 -6.99519742 5.86680316

H -1.56739459 -7.00195948 5.59108503

H -0.50483367 -8.00112567 4.56987756

H 7.76359296 1.02927343 -5.20655241

H 7.75014458 -0.74078795 -5.27069021

H 6.54500199 0.18826446 -6.19590536

C -0.07772420 -0.02025285 2.05220194

O -1.27009886 -0.03485898 2.42227229

O 0.96746043 -0.01249817 2.73340700

[CoP(CO_2_)]^–2^ M = 4

C -2.74127333 -1.17267946 -0.59749901

C -3.76455050 -0.75650970 -1.47898842

C -3.81121368 0.63978609 -1.42388050

C -2.80785149 1.04851345 -0.52051487

N -2.11172405 -0.05819855 -0.06593765

C -2.41049870 -2.54812556 -0.23923625

C -2.53883227 2.41595704 -0.09840978

C -1.18583252 2.81299767 0.20565844

C -0.76024459 3.96290890 0.92879900

C 0.62505764 4.00319400 0.84586583

C 1.02926067 2.86852784 0.09192926

N -0.07655277 2.11148972 -0.24024871

C 2.37050802 2.51665205 -0.29703914

C 2.70634251 1.14312083 -0.61992961

C 3.75455215 0.72612137 -1.47767782

C 3.80624734 -0.66423326 -1.40963806

C 2.78821325 -1.07142907 -0.51150778

N 2.07563733 0.02926661 -0.08720111

C 2.53092406 -2.43500168 -0.06639686

C 1.16987342 -2.81844201 0.27496899

C 0.74923626 -3.90156259 1.07660209

C -0.64943043 -3.93757130 1.01282637

C -1.05265485 -2.87093215 0.18174148

N 0.05893450 -2.14591398 -0.21681736

C 3.58891073 -3.36144292 -0.00426876

C 3.37520620 3.52046990 -0.37262780

C -3.60750555 3.34292208 -0.03509265

C -3.39531934 -3.54999476 -0.30238682

C 3.09461602 4.91866377 -0.63035234

C 4.07253757 5.86078091 -0.71234104

N 5.40977406 5.55150120 -0.54080643

C 5.73157434 4.23385521 -0.26471541

C 4.78890078 3.25463322 -0.20622441

C 3.41631423 -4.80717111 -0.05604016

C 4.45657965 -5.67725140 0.00144764

N 5.76743028 -5.26137022 0.17976389

C 5.99530724 -3.89230646 0.21346070

C 4.99135315 -2.98403017 0.12039567

C -4.83059653 -3.29333444 -0.24604846

C -5.76032271 -4.28040073 -0.27931670

N -5.42161140 -5.62309226 -0.38699285

C -4.07075582 -5.92265037 -0.49982730

C -3.10394728 -4.97332442 -0.42945414

C -4.99122441 2.94984025 0.15706618

C -6.00645623 3.84967595 0.24197243

N -5.80254057 5.21144667 0.08984201

C -4.50413463 5.64123440 -0.11634928

C -3.45357981 4.78027510 -0.17308169

C 6.87325723 -6.19890859 0.05256688

C 6.41842661 6.60121231 -0.46223502

C -6.87908263 6.16248273 0.33450290

C -6.43459503 -6.62652178 -0.67569374

Co -0.03903116 -0.02409305 0.27587845

H -4.38834431 -1.40659252 -2.07963710

H -4.48198321 1.29614711 -1.96519647

H -1.39811529 4.66132968 1.45434229

H 1.28154763 4.73718039 1.29563002

H 4.38347747 1.37711049 -2.07227810

H 4.48519966 -1.32149569 -1.93861649

H 1.38865760 -4.57055128 1.63884707

H -1.30336594 -4.64017252 1.51462579

H 2.07621525 5.23224478 -0.82297500

H 3.85957205 6.89770529 -0.94730865

H 6.78312285 4.03645321 -0.09007792

H 5.11921906 2.24792946 0.01686794

H 2.42634303 -5.21976053 -0.20218059

H 4.31781159 -6.74877775 -0.09392237

H 7.02997381 -3.59794576 0.35383238

H 5.25427256 -1.93650355 0.20043854

H -5.18288330 -2.27832083 -0.11129759

H -6.82205519 -4.07715335 -0.18592901

H -3.83906694 -6.97162048 -0.65068386

H -2.07544127 -5.29798692 -0.52491016

H -5.23392180 1.90184702 0.28104629

H -7.02998216 3.55035059 0.43792949

H -4.38426812 6.70903448 -0.26367574

H -2.47861312 5.19526739 -0.39448382

H 7.18007526 -6.33293440 -0.99437575

H 7.73133882 -5.83317954 0.62338414

H 6.57919771 -7.17068737 0.45795080

H 6.52579404 6.98017949 0.56245842

H 7.38221686 6.20923891 -0.79630148

H 6.13542287 7.42987257 -1.11626739

H -6.95526730 6.42241134 1.39865614

H -6.69772715 7.07584694 -0.23798960

H -7.82941120 5.73243056 0.00830719

H -6.64189683 -6.70620241 -1.75254618

H -6.10014629 -7.60172440 -0.31144322

H -7.36397085 -6.36722208 -0.16074981

C 0.21626979 0.24210437 2.38060401

O 1.04649151 1.01610716 2.84797075

O -0.63548995 -0.53882883 2.82912859

[CoP(CO_2_)]^–2^_2 M = 4

C -2.68211115 1.13472538 -0.81516143

C -3.25970067 2.42358071 -1.01852896

C -2.43202169 3.34288357 -0.39372673

C -1.33143239 2.61538309 0.13640631

N -1.46712544 1.29130264 -0.14371656

C -3.23987681 -0.12927669 -1.16917470

C -0.12801663 3.18259539 0.74661816

C 1.11742374 2.72314974 0.14273210

C 2.24681049 3.49256661 -0.22580197

C 3.12811714 2.63733656 -0.89005172

C 2.52891484 1.34510750 -0.89537552

N 1.28069394 1.42427196 -0.28401938

C 3.13627919 0.14192334 -1.34456606

C 2.59955880 -1.14203093 -1.05984800

C 3.23718742 -2.39841265 -1.26753378

C 2.43247911 -3.36794412 -0.66155957

C 1.30900437 -2.70040321 -0.12339105

N 1.39365716 -1.35533941 -0.39699133

C 0.14231375 -3.28003035 0.54121390

C -1.13738159 -2.77588039 0.05297867

C -2.31301144 -3.52549755 -0.23743948

C -3.22858585 -2.64304455 -0.78619416

C -2.60303839 -1.36099619 -0.82715600

N -1.31918112 -1.47479420 -0.30815288

C 0.23800917 -4.25939743 1.52528507

C 4.45886761 0.23418056 -2.01807044

C -0.19948704 4.14361173 1.74889716

C -4.52426920 -0.19531325 -1.84504116

C 4.62661643 0.86651665 -3.28576094

C 5.84914965 0.99319077 -3.88670628

N 7.01070726 0.45623917 -3.30347551

C 6.88357948 -0.11743122 -2.02596990

C 5.66518676 -0.23963815 -1.41775286

C -0.90552690 -4.81805192 2.23945814

C -0.78019199 -5.80451054 3.16182260

N 0.44847641 -6.33186563 3.52650997

C 1.57619992 -5.76929394 2.95000365

C 1.50607025 -4.78265379 2.02198967

C -5.65829475 0.61042085 -1.49470908

C -6.85011794 0.51000704 -2.15353320

N -7.02737884 -0.36782498 -3.20529973

C -5.95974320 -1.16382710 -3.57927296

C -4.76308606 -1.10907550 -2.92417138

C -1.45163278 4.62274321 2.32574120

C -1.49584861 5.59987429 3.26471626

N -0.35116115 6.18832173 3.78298292

C 0.86894470 5.70443685 3.33629000

C 0.96871312 4.73401135 2.39432929

C 0.53712546 -7.54555400 4.32555768

C 8.31076858 0.94470928 -3.73400294

C -0.43071372 7.39966260 4.58611106

C -8.25885926 -0.35540104 -3.99088859

Co -0.00387727 -0.03858809 -0.04418343

H -4.16265437 2.64156641 -1.57029551

H -2.55322457 4.41810012 -0.35141095

H 2.35963689 4.55859204 -0.06797217

H 4.09394796 2.87979367 -1.31495872

H 4.18319429 -2.54584089 -1.77233401

H 2.59822714 -4.43868451 -0.63539998

H -2.43675759 -4.59067617 -0.09112148

H -4.23776935 -2.86799350 -1.10296716

H 3.75794719 1.27250312 -3.79738230

H 5.99103394 1.47166148 -4.84910561

H 7.80612685 -0.47937842 -1.58588975

H 5.61970567 -0.72614562 -0.44745393

H -1.89673856 -4.41992855 2.06265935

H -1.63551078 -6.21405723 3.68911301

H 2.52325281 -6.15538752 3.31265232

H 2.43463315 -4.36198596 1.65576031

H -5.60398586 1.28058326 -0.64589592

H -7.72225182 1.08689797 -1.86965447

H -6.13373340 -1.81951048 -4.42379820

H -3.96281962 -1.75568887 -3.26457036

H -2.38979001 4.17996137 2.01355253

H -2.43075206 5.95603787 3.68473790

H 1.74140819 6.13740868 3.81491723

H 1.95733991 4.37194661 2.13932732

H 0.49497175 -8.45005153 3.70351747

H 1.47696487 -7.54898038 4.88462159

H -0.28985632 -7.57465163 5.04031326

H 8.55820381 1.92061963 -3.28650260

H 9.08550896 0.22637668 -3.44963085

H 8.32137608 1.04994986 -4.82290382

H -1.33197261 7.37117346 5.20477934

H 0.43898508 7.45950038 5.24629821

H -0.46170047 8.30434915 3.96326000

H -8.44259146 -1.35222768 -4.39769965

H -9.09823506 -0.07947952 -3.34880308

H -8.19110850 0.36171789 -4.81780649

C 0.06432900 -0.15867761 1.90869024

O 1.19932512 -0.06920813 2.42401869

O -1.04662159 -0.31690836 2.45566480

[CoP(CO_2_)]^–2^ M = 6

C -2.62718732 1.10873343 -0.86138895

C -3.31099148 2.36337649 -0.96219860

C -2.50798308 3.31143957 -0.34717119

C -1.32078878 2.64834569 0.07502964

N -1.39082279 1.31233903 -0.27542714

C -3.17120139 -0.15877894 -1.19674332

C -0.13424197 3.25699873 0.62974683

C 1.13024616 2.75506011 0.14297207

C 2.28227600 3.51444490 -0.20598913

C 3.17409269 2.64710763 -0.82150122

C 2.57488605 1.34774062 -0.80009029

N 1.31377518 1.43866371 -0.24109436

C 3.20799129 0.14045372 -1.19911762

C 2.67196689 -1.13868400 -0.89266644

C 3.35249477 -2.38894885 -1.03738541

C 2.54955867 -3.35673986 -0.44781706

C 1.36836259 -2.70484873 0.00216036

N 1.44084305 -1.35880058 -0.30045111

C 0.17457486 -3.32691231 0.53236295

C -1.08236427 -2.80923666 0.05084383

C -2.24054608 -3.55691772 -0.30878987

C -3.13271067 -2.67420444 -0.89580614

C -2.52985968 -1.37560031 -0.84605646

N -1.26328222 -1.48299286 -0.30373700

C 0.24050793 -4.41418050 1.44778989

C 4.53842756 0.22825659 -1.85739978

C -0.21353542 4.32013720 1.57236983

C -4.52943699 -0.22845911 -1.79875754

C 4.69895255 0.76559349 -3.17016208

C 5.92217155 0.88344497 -3.77041233

N 7.09370109 0.42926356 -3.13923277

C 6.97277947 -0.04730323 -1.82179882

C 5.75300687 -0.15982443 -1.21400318

C -0.92530139 -5.02364123 2.05280109

C -0.84008743 -6.08316673 2.90306998

N 0.37224633 -6.62962013 3.27690043

C 1.51963040 -6.00818293 2.82323979

C 1.48024860 -4.94644602 1.97202776

C -5.70902700 0.15623380 -1.08839326

C -6.95735644 0.05882202 -1.63617761

N -7.15304811 -0.47948416 -2.91832251

C -6.00878218 -0.82136848 -3.65902920

C -4.75561769 -0.71877924 -3.11953115

C -1.46054579 4.81213751 2.11891391

C -1.51735425 5.85241689 2.99479714

N -0.38014848 6.48959861 3.45313378

C 0.84069286 5.97903969 3.05770167

C 0.94320296 4.94068753 2.18305510

C 0.43832692 -7.87284028 4.03686591

C 8.38279383 0.91655032 -3.60249015

C -0.46892791 7.71334927 4.24173657

C -8.41017944 -0.24537628 -3.61045068

Co 0.02241330 -0.02635234 -0.03364564

H -4.28155934 2.51163270 -1.41616647

H -2.69343406 4.37571774 -0.27826048

H 2.38559080 4.58691214 -0.10189986

H 4.14539097 2.87893709 -1.23847913

H 4.32036829 -2.52475771 -1.50146771

H 2.73138270 -4.42355822 -0.41626277

H -2.34750673 -4.63085913 -0.22700368

H -4.10780612 -2.89242451 -1.31039835

H 3.82286842 1.10146816 -3.71898856

H 6.05861714 1.29078328 -4.76578494

H 7.90191356 -0.34526631 -1.34899081

H 5.71472229 -0.57026092 -0.20857859

H -1.90915895 -4.61659325 1.86359511

H -1.71681004 -6.53611145 3.35226246

H 2.45139413 -6.40517009 3.21043221

H 2.42104062 -4.47922896 1.71440659

H -5.61905045 0.54623686 -0.07829624

H -7.86024469 0.34957970 -1.11129209

H -6.19687268 -1.19786731 -4.65798747

H -3.90929298 -1.03432169 -3.72451803

H -2.39282218 4.32908902 1.85984620

H -2.45468359 6.21763768 3.39931044

H 1.71045130 6.44238054 3.50998123

H 1.93427738 4.56030235 1.97645311

H 0.45880413 -8.74563323 3.37251883

H 1.34006475 -7.87690809 4.65426335

H -0.43312494 -7.94758570 4.69171967

H 8.60016004 1.93295941 -3.23710124

H 9.17494691 0.24639597 -3.25553367

H 8.39826257 0.93096361 -4.69636825

H -1.36502055 7.68206560 4.86643287

H 0.40621215 7.79397156 4.89103665

H -0.51651877 8.60019609 3.59767245

H -8.54527513 -0.99872658 -4.39211458

H -9.24004512 -0.33094718 -2.90302046

H -8.44937220 0.75188503 -4.07593729

C 0.00817088 -0.05828776 1.93084857

O 1.13524392 -0.06267266 2.47115220

O -1.12289906 -0.07052571 2.46098084
