# Supplementary material for: Self-Assembled Liposomes Enhance Electron Transfer for Efficient Photocatalytic CO2 Reduction
Source: J Am Chem Soc. 2022 May 20;144(21):9399–412. doi: 10.1021/jacs.2c01725 (PMC9164230; doi:10.1021/jacs.2c01725)
Supplement: Supplementary file 2 — ja2c01725_si_002.zip [file ja2c01725_si_002.zip › Coordinates/CoP_COOH/Coord_CoP_COOH.docx]

[CoP(COOH)]^+4^ M = 1

C -1.52722876 2.60574058 -0.05225585

C -1.33835100 4.02534108 0.10988937

C -0.00012129 4.23944410 0.17553114

C 0.63688286 2.95519702 0.02027005

N -0.30853238 1.95396143 -0.08471144

C -2.77116677 2.00465293 -0.23754507

C 2.01578099 2.77310734 -0.08034868

C 2.61718439 1.53000597 -0.27680995

C 4.03636215 1.34190966 -0.46629156

C 4.24612868 0.00436301 -0.54512437

C 2.95493383 -0.62749624 -0.40789371

N 1.96193775 0.31976696 -0.29338131

C 2.77585166 -2.00267426 -0.26680529

C 1.53626775 -2.60803294 -0.06452758

C 1.35049121 -4.02757680 0.10772329

C 0.01350238 -4.24222659 0.19254664

C -0.62667470 -2.95924130 0.03997089

N 0.31919852 -1.95861602 -0.08721470

C -2.00750782 -2.78207287 -0.03899336

C -2.61519157 -1.53839584 -0.21534083

C -4.02258768 -1.34803593 -0.44832656

C -4.23374190 -0.00814556 -0.53086263

C -2.95630117 0.62844754 -0.35287944

N -1.95910746 -0.32022153 -0.18496772

C -2.88911591 -3.98007249 0.03610634

C 3.98260031 -2.87557636 -0.31107415

C 2.89673994 3.97049882 0.00963755

C -3.97623905 2.88132421 -0.30307186

C 4.69313976 -3.06607218 -1.50650939

C 5.80491672 -3.88526966 -1.52397659

N 6.22386448 -4.51086818 -0.39779780

C 5.56196077 -4.34353105 0.76913975

C 4.44295984 -3.53276516 0.83776245

C -3.80744364 -4.13139700 1.08402040

C -4.63025732 -5.24319717 1.12875998

N -4.56350053 -6.19174579 0.16795235

C -3.68615794 -6.07326726 -0.85720300

C -2.84178764 -4.98394396 -0.94453062

C -4.46528977 3.52423712 0.84198473

C -5.57974482 4.33868788 0.75252274

N -6.20936531 4.52287810 -0.43033822

C -5.76490329 3.90746939 -1.55085755

C -4.65386582 3.08548373 -1.51341710

C 2.85476873 4.98275205 -0.96274287

C 3.68419599 6.08129321 -0.85146046

N 4.54293350 6.20045158 0.18944023

C 4.60844323 5.24093643 1.13970802

C 3.79997153 4.12038120 1.07109142

C -5.44747017 -7.38156721 0.21656312

C 7.41765971 -5.38619958 -0.47049790

C 5.41494754 7.39714508 0.26254398

C -7.42362185 5.37139169 -0.49954245

Co 0.01821875 -0.00144236 -0.04163669

H -2.12551973 4.76312008 0.15489617

H 0.50678744 5.18429016 0.30102734

H 4.77236716 2.12921011 -0.53399900

H 5.18868177 -0.50787307 -0.66846155

H 2.13703665 -4.76639388 0.14383902

H -0.49214981 -5.18606535 0.33033408

H -4.75588843 -2.13476819 -0.54461708

H -5.17345398 0.50062542 -0.68604233

H 4.37736968 -2.58541435 -2.42443831

H 6.38127968 -4.07217265 -2.42122929

H 5.95347878 -4.86734173 1.63095391

H 3.93724678 -3.41406588 1.78826480

H -3.88444377 -3.38964439 1.86959959

H -5.34845656 -5.40178123 1.92225967

H -3.69526670 -6.86720192 -1.59325177

H -2.15918965 -4.91310314 -1.78248553

H -3.98478120 3.39441492 1.80404300

H -5.99483116 4.85306434 1.60933259

H -6.31793231 4.09959968 -2.46128289

H -4.31545176 2.61550095 -2.42877387

H 2.18321399 4.91404280 -1.80976849

H 3.69297367 6.88390471 -1.57806682

H 5.31167277 5.40024858 1.94635116

H 3.87158558 3.37219950 1.85098555

H -6.03783691 -7.41746123 -0.69994703

H -4.82829620 -8.27556308 0.30356979

H -6.10729145 -7.30073578 1.07800306

H 8.26733587 -4.79456669 -0.81382503

H 7.62332182 -5.79127880 0.51840059

H 7.21487549 -6.19854644 -1.16981425

H 4.78902183 8.28998877 0.23980376

H 5.98355434 7.36783711 1.18990780

H 6.09434625 7.38915384 -0.59123979

H -7.40662392 6.08170124 0.32542749

H -7.42422966 5.90837355 -1.44737615

H -8.30394584 4.73017847 -0.42737049

C 0.12890876 0.00028323 1.85678640

O -1.05356769 0.00100442 2.50522704

O 1.16685869 0.00539198 2.46664515

H -1.76791700 -0.04054678 1.84140280

[CoP(COOH)]^+4^ M = 3

C -2.69183345 0.96477252 -0.98480375

C -3.33105245 2.21963783 -1.27842392

C -2.56961110 3.20243394 -0.70554792

C -1.44851789 2.55242151 -0.07938453

N -1.50102833 1.22388318 -0.28950859

C -3.17638322 -0.31837911 -1.19955580

C -0.31887710 3.19679078 0.59228871

C 0.96298113 2.77101614 0.16051875

C 2.14376427 3.57915368 -0.02639996

C 3.07507671 2.78538063 -0.63969439

C 2.48640988 1.48239419 -0.78218989

N 1.19767889 1.50450826 -0.31595757

C 3.16162899 0.30934970 -1.21658716

C 2.67386213 -0.97175422 -1.01034220

C 3.33142321 -2.22713552 -1.27808103

C 2.57541080 -3.20461262 -0.69592274

C 1.43719897 -2.54928952 -0.09442275

N 1.47243902 -1.23393620 -0.33901909

C 0.31640239 -3.19513079 0.59415463

C -0.97342386 -2.77670272 0.17498862

C -2.14560830 -3.59019437 -0.02366652

C -3.08053852 -2.79760586 -0.63603068

C -2.50232828 -1.49018558 -0.76529970

N -1.21558413 -1.50554752 -0.28481669

C 0.52396455 -4.21600728 1.59034038

C 4.49650320 0.47908263 -1.84913291

C -0.50983734 4.22863387 1.57942262

C -4.50304385 -0.48966868 -1.84607115

C 4.63475484 1.24236233 -3.01908726

C 5.87593956 1.37344752 -3.61134181

N 6.96758215 0.77942091 -3.07339366

C 6.86788622 0.05645615 -1.93602726

C 5.64717519 -0.10653554 -1.30441785

C -0.54673731 -4.71699194 2.38526114

C -0.33326509 -5.68197575 3.33758295

N 0.91047922 -6.17477012 3.58151927

C 1.97064670 -5.67406178 2.90021641

C 1.81180531 -4.71050455 1.93131608

C -5.65999482 0.09537042 -1.31422581

C -6.87715879 -0.08583093 -1.94771512

N -6.96703822 -0.82427820 -3.07578681

C -5.86647657 -1.40758202 -3.60849982

C -4.62929011 -1.26010574 -3.01326983

C -1.78600301 4.76360447 1.90527023

C -1.92470590 5.74029130 2.86315340

N -0.85681521 6.21553391 3.55171149

C 0.37367090 5.68225640 3.32484896

C 0.56708021 4.70399038 2.38226634

C 1.08546997 -7.22508867 4.60469631

C 8.28352143 0.95771780 -3.73447602

C -1.00836712 7.28494579 4.55805700

C -8.27207457 -1.01231303 -3.75396952

Co -0.00121103 -0.00223946 -0.14712528

H -4.24009199 2.34905206 -1.84784921

H -2.73252440 4.26911745 -0.75319273

H 2.22544693 4.63429590 0.18959315

H 4.06824853 3.06890990 -0.95632200

H 4.24847822 -2.35639725 -1.83412592

H 2.74899791 -4.27039614 -0.71946630

H -2.22038455 -4.64792249 0.18218325

H -4.06998569 -3.08661372 -0.95943794

H 3.77890255 1.72014381 -3.47987886

H 6.03251815 1.94134906 -4.51944919

H 7.78132821 -0.37746951 -1.55128979

H 5.60389637 -0.68175622 -0.38800150

H -1.54941722 -4.32542805 2.28896016

H -1.13477649 -6.08209736 3.94568546

H 2.94384791 -6.07015110 3.15875025

H 2.70758401 -4.33925601 1.45562762

H -5.62485206 0.67857076 -0.40248635

H -7.79556946 0.34380239 -1.57051616

H -6.01430302 -1.98241159 -4.51377305

H -3.76716688 -1.73447334 -3.46572263

H -2.68757556 4.41584189 1.42253956

H -2.88761012 6.16898393 3.10790725

H 1.18097789 6.06181229 3.93852265

H 1.55803662 4.28046734 2.29997969

H 0.52070688 -8.11029356 4.30649711

H 2.14248287 -7.47305430 4.68522457

H 0.72014222 -6.85414956 5.56404778

H 8.64427884 1.96756891 -3.53210891

H 8.98262809 0.22403714 -3.33763507

H 8.16033524 0.80733775 -4.80700148

H -2.06216741 7.54347252 4.64896209

H -0.63296496 6.92831615 5.51886066

H -0.44009435 8.16015666 4.23718233

H -8.48744746 -2.08010818 -3.80886236

H -9.04746557 -0.50609869 -3.18231400

H -8.21036656 -0.58669034 -4.75640538

C 0.05732164 -0.00627288 1.74614292

O 1.07671478 -0.10733919 2.37778244

O -1.13551796 0.10242976 2.37082407

H -1.83234841 0.23174229 1.70287657

[CoP(COOH)]^+3^ M = 2

C 2.62293888 -1.21039089 -0.88408923

C 3.23704931 -2.49937744 -1.00483477

C 2.40342257 -3.40104140 -0.39523720

C 1.24550493 -2.67503311 0.04764172

N 1.37688936 -1.35981713 -0.28218940

C 3.18997857 0.03137614 -1.20090429

C 0.03533878 -3.23463291 0.58839060

C -1.18858532 -2.69505023 0.06718485

C -2.37971351 -3.42650036 -0.27812775

C -3.23015546 -2.54481744 -0.89079227

C -2.58061438 -1.26478092 -0.88797637

N -1.31935534 -1.39957458 -0.33209517

C -3.17558384 -0.03420761 -1.20368319

C -2.60798876 1.20839346 -0.88838645

C -3.22998072 2.49651027 -0.99623001

C -2.39785404 3.39617165 -0.38329890

C -1.23264536 2.67014577 0.04591812

N -1.35919635 1.35957076 -0.30035636

C -0.02753361 3.23087364 0.59517642

C 1.20213645 2.69494093 0.08004810

C 2.37803670 3.42948867 -0.29351547

C 3.23074699 2.54609497 -0.90560815

C 2.59788645 1.26203989 -0.87863878

N 1.34306714 1.38901336 -0.29315089

C -0.05402360 4.29905663 1.55513390

C -4.52405290 -0.06728853 -1.82405588

C 0.05816029 -4.30153686 1.55051053

C 4.53531564 0.06290340 -1.82846741

C -4.71990890 -0.70665850 -3.06027093

C -5.97487879 -0.73571434 -3.63420066

N -7.03371130 -0.15574808 -3.01921461

C -6.88001281 0.45834383 -1.82389921

C -5.64267171 0.51495126 -1.20907135

C 1.13014835 4.83422684 2.15124901

C 1.07797343 5.85051196 3.06861683

N -0.10582567 6.37992726 3.48507543

C -1.26455108 5.84330645 3.01416615

C -1.26734361 4.82505512 2.09651588

C 5.65429461 -0.51519600 -1.21128171

C 6.89142460 -0.45959312 -1.82697166

N 7.04317555 0.14928994 -3.02462672

C 5.98269780 0.72219497 -3.64399133

C 4.72805450 0.69465168 -3.06911604

C 1.26997096 -4.84089768 2.08174474

C 1.26372092 -5.85789705 3.00088583

N 0.10327328 -6.37982358 3.48353739

C -1.07789150 -5.83645059 3.07790986

C -1.12648827 -4.82097711 2.15935504

C -0.12306071 7.52377924 4.41233551

C -8.36804012 -0.23417498 -3.65924769

C 0.11508973 -7.52251983 4.41235273

C 8.37087246 0.20517199 -3.68051543

Co 0.00061763 -0.00351592 -0.14073190

H 4.17530329 -2.71260635 -1.49608924

H 2.53353249 -4.47208328 -0.34135168

H -2.52666777 -4.48920360 -0.15366527

H -4.21194591 -2.75645886 -1.28953356

H -4.17131237 2.70979963 -1.48146627

H -2.53226744 4.46607129 -0.31851660

H 2.51699217 4.49512327 -0.18563169

H 4.20518963 2.76289444 -1.31935272

H -3.89168135 -1.17228251 -3.58010671

H -6.17063294 -1.20976847 -4.58751203

H -7.76800262 0.88810101 -1.37930498

H -5.55884568 1.00149087 -0.24544546

H 2.10498358 4.43012426 1.92078319

H 1.97208616 6.27027874 3.51264290

H -2.18175157 6.25693527 3.41399917

H -2.22782051 4.41482667 1.82121929

H 5.57154546 -0.99652898 -0.24494933

H 7.77999015 -0.88654475 -1.38120476

H 6.17786554 1.18798411 -4.60166015

H 3.89871244 1.15656310 -3.59050218

H 2.23298291 -4.44397099 1.79621281

H 2.17966071 -6.28254288 3.39191154

H -1.97268885 -6.24398253 3.53187568

H -2.09817128 -4.40393476 1.93914219

H 0.02236965 8.45172481 3.85323989

H -1.08284207 7.55212375 4.92779002

H 0.67611626 7.40532858 5.14514261

H -8.73654572 -1.25840223 -3.58038089

H -9.04732111 0.44730150 -3.15060389

H -8.27417422 0.05317496 -4.70677159

H 1.08250906 -7.56749972 4.91204653

H -0.66982816 -7.38878477 5.15787870

H -0.05606366 -8.44836439 3.85709734

H 8.64918302 1.25019756 -3.82323376

H 9.10288822 -0.28961648 -3.04516629

H 8.30982601 -0.30492792 -4.64278531

C -0.03571614 -0.01645296 1.74671501

O -1.02929493 -0.17907000 2.41091472

O 1.16250682 0.16356213 2.35573592

H 1.83539248 0.31392653 1.66821541

[CoP(COOH)]^+3^ M = 4

C 2.72527103 -1.16944052 -0.56406405

C 3.22289464 -2.47285300 -0.87333030

C 2.34023740 -3.39073513 -0.32730826

C 1.29650543 -2.65004707 0.28118688

N 1.50099043 -1.31162801 0.09851997

C 3.29005226 0.09989367 -0.82084255

C 0.09485806 -3.18957069 0.93902493

C -1.14759065 -2.69436195 0.47113368

C -2.39210989 -3.40077626 0.31383464

C -3.24392933 -2.56773208 -0.36652500

C -2.54455883 -1.33405230 -0.57452582

N -1.26879768 -1.43893576 -0.08563979

C -3.11966276 -0.11920442 -1.05384897

C -2.56400395 1.12577473 -0.79475398

C -3.11343368 2.43153415 -1.06385328

C -2.32365546 3.33554307 -0.41080308

C -1.25797502 2.58972300 0.22206179

N -1.38365068 1.28214492 -0.05919650

C -0.13922139 3.12939925 0.98841609

C 1.15200045 2.63374571 0.65119101

C 2.40174879 3.33657023 0.65842022

C 3.33629457 2.50679386 0.08036756

C 2.66302256 1.29546282 -0.26896665

N 1.35213235 1.38635715 0.07902332

C -0.34967431 4.12100348 2.00711645

C -4.40769720 -0.21738194 -1.77894220

C 0.19357592 -4.19447230 1.96252078

C 4.52232678 0.26923378 -1.55870693

C -4.51378739 -1.04995238 -2.90722390

C -5.70819662 -1.13505354 -3.59297689

N -6.79383096 -0.43194203 -3.18785124

C -6.72839877 0.36291791 -2.09601591

C -5.55318725 0.48604721 -1.37722792

C 0.68190365 4.49846497 2.92098653

C 0.47581804 5.44945409 3.88680429

N -0.73100286 6.05924354 4.04192163

C -1.76960864 5.67251170 3.25004688

C -1.61676821 4.72556757 2.27095834

C 5.63781753 -0.60744904 -1.45535504

C 6.77741158 -0.39663139 -2.19200004

N 6.88341438 0.64944898 -3.05597624

C 5.84074339 1.52473075 -3.17683519

C 4.69151141 1.37001746 -2.44706084

C 1.43008982 -4.80252702 2.32699720

C 1.49206760 -5.75951785 3.30895645

N 0.38298619 -6.15434611 3.99035867

C -0.80783952 -5.55129238 3.72616847

C -0.92338239 -4.58475004 2.75942880

C -0.91678141 7.13499382 5.03111612

C -8.06351031 -0.57621823 -3.93833977

C 0.46367341 -7.24679126 4.97770786

C 8.09089970 0.84360870 -3.87700367

Co 0.05981690 -0.01960912 -0.07518944

H 4.11362025 -2.70720765 -1.43479771

H 2.40857166 -4.46841203 -0.37706228

H -2.57526431 -4.42748357 0.59479283

H -4.25862756 -2.77956616 -0.67201989

H -3.98905646 2.64352374 -1.65992588

H -2.42656955 4.41076733 -0.40902974

H 2.55137652 4.35716912 0.97846894

H 4.38434004 2.71390489 -0.08117746

H -3.66179174 -1.61785168 -3.26047755

H -5.83397761 -1.75216909 -4.47339375

H -7.63536338 0.88262749 -1.81652373

H -5.54359019 1.11404570 -0.49544887

H 1.64609756 4.01137068 2.90367423

H 1.25622311 5.74585288 4.57595278

H -2.72136681 6.15165209 3.44193716

H -2.50279405 4.44619008 1.72047341

H 5.63966048 -1.42200537 -0.74410161

H 7.64471684 -1.03874928 -2.10625095

H 5.97481392 2.33073994 -3.88719753

H 3.89174349 2.08626795 -2.58965084

H 2.35554668 -4.52104114 1.84563107

H 2.42144602 -6.24137739 3.58470754

H -1.64931222 -5.86339862 4.33137829

H -1.88331700 -4.09963155 2.65483428

H -0.85972478 8.10497054 4.53065255

H -1.89173678 7.02166766 5.50718689

H -0.13495942 7.06503046 5.78699112

H -8.49042575 -1.55700752 -3.72144923

H -8.75178734 0.20783213 -3.62841156

H -7.85490018 -0.48280041 -5.00427838

H 1.44807206 -7.23534718 5.44549802

H -0.30187157 -7.09817283 5.73915662

H 0.30362404 -8.20287746 4.47313175

H 8.43889440 1.87290093 -3.77085321

H 8.86910951 0.16179555 -3.53624323

H 7.85679267 0.63850224 -4.92457906

C 0.15456262 0.02125520 -1.97539915

O 0.17615195 1.03793249 -2.62360500

O 0.19717970 -1.17716484 -2.60549254

H 0.14729408 -1.88925000 -1.94422345

[CoP(COOH)]^+2^ M = 1

C 2.62366665 -1.21365739 -0.91130804

C 3.22866018 -2.50266305 -1.03190006

C 2.38613229 -3.40341383 -0.42185416

C 1.24148235 -2.67357860 0.03167895

N 1.37887344 -1.36003210 -0.29195983

C 3.18494908 0.02873610 -1.23491992

C 0.03049267 -3.21852511 0.61339332

C -1.19674650 -2.69366517 0.04890979

C -2.37185065 -3.42857809 -0.31637779

C -3.22947060 -2.54468768 -0.92945705

C -2.58905569 -1.26587360 -0.92149492

N -1.33108421 -1.39890596 -0.33908608

C -3.17831644 -0.03292302 -1.23510349

C -2.61669470 1.21199354 -0.91595350

C -3.22287272 2.50040592 -1.03389322

C -2.37903730 3.40068176 -0.42254125

C -1.23527906 2.66899284 0.02890239

N -1.37223112 1.35839586 -0.30147050

C -0.02604273 3.21266266 0.62205469

C 1.20338069 2.69470556 0.05752632

C 2.36445423 3.43098750 -0.33448251

C 3.22280234 2.54550663 -0.94890276

C 2.59727303 1.26312002 -0.91843558

N 1.34689389 1.38891066 -0.30798527

C -0.05011585 4.21807781 1.60601986

C -4.51914637 -0.06467946 -1.86779147

C 0.05384955 -4.22548181 1.59787138

C 4.52431121 0.05853884 -1.87119769

C -4.71070821 -0.72014375 -3.09717467

C -5.95983484 -0.74777783 -3.68266378

N -7.02078891 -0.15164489 -3.08602590

C -6.87266907 0.47779716 -1.89747891

C -5.64083362 0.53441776 -1.27283419

C 1.14008018 4.75350793 2.22370591

C 1.08660345 5.74061456 3.16221909

N -0.10035941 6.26530649 3.60356099

C -1.26319688 5.72831179 3.11559112

C -1.26921973 4.74032551 2.17645279

C 5.64649499 -0.53087192 -1.26851845

C 6.87950873 -0.47489968 -1.89127266

N 7.02707591 0.14475977 -3.08443555

C 5.96438820 0.72859643 -3.69067345

C 4.71427079 0.70199231 -3.10714757

C 1.27258709 -4.75008273 2.16589573

C 1.26671663 -5.73893024 3.10456487

N 0.10405785 -6.27235756 3.59556877

C -1.08226662 -5.74315955 3.15915718

C -1.13548671 -4.75594486 2.22029671

C -0.12333193 7.40848589 4.52044306

C -8.34916587 -0.22970082 -3.73677959

C 0.12587456 -7.41458921 4.51400872

C 8.34906391 0.19672533 -3.75060852

Co -0.00505512 -0.00383794 -0.14592318

H 4.16742049 -2.72083204 -1.52111734

H 2.51303783 -4.47457463 -0.35220217

H -2.51526225 -4.49130687 -0.18248380

H -4.21121920 -2.75920105 -1.32831863

H -4.16257133 2.71962517 -1.52086985

H -2.50670005 4.47149280 -0.34795639

H 2.50147654 4.49622585 -0.21392694

H 4.19762729 2.76419251 -1.36221499

H -3.88094156 -1.20013645 -3.60101062

H -6.15062711 -1.23308574 -4.63134276

H -7.76190958 0.91911898 -1.46679901

H -5.56113757 1.03409440 -0.31576091

H 2.11519224 4.35901998 1.97215879

H 1.98141313 6.14943740 3.61661602

H -2.17958016 6.12847893 3.53300922

H -2.22939830 4.33743037 1.88396019

H 5.56718149 -1.02076561 -0.30634361

H 7.76944062 -0.90915058 -1.45526443

H 6.15536784 1.20192408 -4.64550531

H 3.88363042 1.17447220 -3.61667090

H 2.23353496 -4.34948817 1.87311441

H 2.18326552 -6.14173301 3.51894534

H -1.97692377 -6.14786574 3.61748026

H -2.10989790 -4.35694765 1.97410845

H -0.09172676 8.34988885 3.96238917

H -1.03556628 7.37469434 5.11856983

H 0.73861243 7.35605707 5.18817863

H -8.72587162 -1.25030482 -3.64914847

H -9.02882624 0.46228053 -3.24286800

H -8.24477065 0.04404985 -4.78703360

H 1.04390248 -7.38754119 5.10343282

H -0.72948206 -7.35410007 5.18946736

H 0.08134868 -8.35603243 3.95704873

H 8.62059830 1.24029186 -3.91543868

H 9.08908277 -0.28123542 -3.11150857

H 8.28517757 -0.33165477 -4.70294582

C -0.01932816 -0.01141920 1.73778049

O -0.99108998 -0.20225232 2.42906266

O 1.18751248 0.20672542 2.32390032

H 1.82710440 0.39745636 1.61363792

[CoP(COOH)]^+2^ M = 3

C -2.70241801 1.13085322 -0.85118296

C -3.25820765 2.41850165 -1.09446444

C -2.43627236 3.34884570 -0.48060132

C -1.34246617 2.63965389 0.07489042

N -1.48955321 1.29570093 -0.18299727

C -3.26383319 -0.14878902 -1.13938624

C -0.14679083 3.20745008 0.66293841

C 1.09732799 2.70398938 0.15979151

C 2.28056066 3.46430970 -0.14126727

C 3.13494906 2.62964747 -0.81384291

C 2.50092126 1.34460455 -0.88562228

N 1.24570599 1.42876370 -0.30521323

C 3.10449908 0.14779819 -1.30782587

C 2.56912561 -1.12275165 -1.04328685

C 3.20514496 -2.39460566 -1.23588407

C 2.43843430 -3.32648923 -0.58264534

C 1.29769614 -2.63575052 -0.04734726

N 1.36692532 -1.31922639 -0.38051326

C 0.16840797 -3.20931758 0.63585374

C -1.12699162 -2.71225634 0.24672024

C -2.33003664 -3.45528943 0.08911369

C -3.25313629 -2.61371874 -0.50108895

C -2.62161507 -1.35048086 -0.69136027

N -1.31999054 -1.43502503 -0.23460452

C 0.33643749 -4.22857148 1.62621571

C 4.42762167 0.24397543 -1.96974468

C -0.21147977 4.26166349 1.62444358

C -4.53579921 -0.26885932 -1.81499757

C 4.58357335 0.99848606 -3.14673056

C 5.81489166 1.07654117 -3.76480502

N 6.89303325 0.43603511 -3.25061126

C 6.78128249 -0.28387029 -2.11054503

C 5.56890363 -0.39329068 -1.45593827

C -0.75229839 -4.74126294 2.40494306

C -0.57089360 -5.71521784 3.34915501

N 0.66111193 -6.23266602 3.62234912

C 1.74415436 -5.71333898 2.97252462

C 1.61723187 -4.73528615 2.02454646

C -5.65498140 0.58582358 -1.57202302

C -6.83981705 0.42382828 -2.23874052

N -7.00611783 -0.55299460 -3.18216190

C -5.95965413 -1.40081495 -3.44666551

C -4.76714447 -1.29239444 -2.78640441

C -1.44847597 4.82470371 2.08010472

C -1.48325320 5.83457193 3.00217835

N -0.34564736 6.33764296 3.56446659

C 0.85203420 5.77529648 3.22851171

C 0.94233592 4.76459281 2.31080924

C 0.82609965 -7.34084523 4.57434844

C 8.20197610 0.56387988 -3.93259735

C -0.40893825 7.48278314 4.48427625

C -8.25561437 -0.67687679 -3.94206517

Co -0.03561134 -0.00941201 -0.11564358

H -4.14645825 2.63496856 -1.66776327

H -2.54760371 4.42427428 -0.49437806

H 2.41924130 4.51890169 0.04695251

H 4.11007370 2.87776560 -1.20827321

H 4.11298907 -2.57594974 -1.79284918

H 2.60090108 -4.39424607 -0.54824538

H -2.46274502 -4.50481869 0.30887772

H -4.27461289 -2.85530297 -0.75680399

H 3.73949473 1.51288949 -3.58933419

H 5.97701696 1.63545205 -4.67765913

H 7.68368691 -0.75396654 -1.74220171

H 5.52113249 -0.96403320 -0.53725107

H -1.74977001 -4.34288243 2.29150035

H -1.39418747 -6.10977380 3.93149319

H 2.70912381 -6.11061838 3.26260432

H 2.52980885 -4.33727375 1.60572845

H -5.61056695 1.34184177 -0.79948966

H -7.70513297 1.04256423 -2.03576384

H -6.13340608 -2.14245256 -4.21657385

H -3.97253979 -1.98000872 -3.04932310

H -2.39453695 4.44919035 1.71688195

H -2.41417247 6.27602470 3.33599804

H 1.72250298 6.16392936 3.74228917

H 1.91857275 4.32980818 2.15017431

H 0.88160026 -8.29018812 4.03435646

H 1.74236964 -7.19174465 5.14778555

H -0.02444445 -7.35821390 5.25617254

H 8.61118936 1.55527587 -3.72992918

H 8.87646445 -0.20269048 -3.55516112

H 8.05570379 0.42866091 -5.00439027

H -1.33380028 7.43182926 5.06070395

H 0.44064941 7.44350989 5.16673124

H -0.38017764 8.41802107 3.91805341

H -8.56472281 -1.72413877 -3.96977691

H -9.03255160 -0.08831843 -3.45394345

H -8.11217050 -0.31167504 -4.96338018

C 0.05024246 -0.05580708 1.77077976

O 1.02530448 -0.34560905 2.42186852

O -1.11441633 0.25181961 2.39608626

H -1.73745871 0.53762954 1.69741921

[CoP(COOH)]^+2^ M = 5

C -2.61577728 1.05114729 -0.98673869

C -3.09284448 2.34016357 -1.38372958

C -2.31191734 3.28680900 -0.74058482

C -1.34113167 2.58059175 0.01090753

N -1.49522125 1.23710381 -0.17039261

C -3.14296226 -0.23252831 -1.25488525

C -0.22638673 3.14058127 0.79209179

C 1.06468498 2.69822021 0.39190345

C 2.29788631 3.41642088 0.34475562

C 3.20583239 2.61355434 -0.31694499

C 2.53242815 1.39896961 -0.65269309

N 1.24203770 1.46475874 -0.22135920

C 3.14530594 0.22773667 -1.26830190

C 2.61911132 -1.05944525 -1.01089251

C 3.13908952 -2.34918413 -1.36776195

C 2.36683950 -3.29446218 -0.72160068

C 1.35439231 -2.58640849 -0.01394379

N 1.47647047 -1.25557775 -0.23725641

C 0.24213520 -3.15825106 0.76120591

C -1.05372390 -2.72003207 0.36796731

C -2.28169049 -3.44298336 0.30917672

C -3.19663045 -2.63120969 -0.33514126

C -2.53114148 -1.40967009 -0.64970782

N -1.23723918 -1.47640127 -0.22026764

C 0.47269539 -4.11913953 1.78758938

C 4.33567519 0.43574013 -2.05926928

C -0.45299268 4.10319122 1.81655389

C -4.32896356 -0.42633751 -2.05633173

C 4.46070068 1.57238813 -2.91448392

C 5.57066554 1.76371798 -3.69231514

N 6.62384414 0.88957366 -3.66167056

C 6.56659477 -0.18621042 -2.82473921

C 5.46587961 -0.43382311 -2.04587034

C -0.56353114 -4.57443701 2.66615861

C -0.31862594 -5.50216146 3.64115369

N 0.92911641 -6.02112027 3.84152808

C 1.96332791 -5.56645149 3.07374441

C 1.77283523 -4.64068389 2.08391380

C -5.44970569 0.45372601 -2.03876212

C -6.55206325 0.22041101 -2.82091926

N -6.62032748 -0.85409616 -3.65683001

C -5.57107884 -1.73139027 -3.69999542

C -4.45937326 -1.55421898 -2.92071533

C -1.74709810 4.65116408 2.09216553

C -1.93368874 5.58056898 3.07854750

N -0.90212725 6.01396259 3.86328015

C 0.33736505 5.47015494 3.68206137

C 0.57893775 4.53813551 2.71001150

C 1.14548813 -7.08157564 4.83732140

C 7.82680748 1.16545278 -4.46170896

C -1.12315433 7.07737954 4.85491052

C -7.78587619 -1.07118660 -4.52839060

Co 0.01029465 -0.00990630 -0.05690015

H -3.90589769 2.54109242 -2.06439037

H -2.39240988 4.36272862 -0.81442773

H 2.45271484 4.43268150 0.67794927

H 4.23539977 2.84758303 -0.54696524

H 3.97340770 -2.54685233 -2.02257275

H 2.47047332 -4.36991107 -0.76715409

H -2.43092792 -4.46646614 0.62220461

H -4.22553090 -2.86647797 -0.56706926

H 3.65078767 2.28710688 -2.99438990

H 5.66545683 2.59802354 -4.37602067

H 7.44455437 -0.81918047 -2.79788306

H 5.50802236 -1.27467860 -1.36733023

H -1.56044455 -4.16054688 2.60766301

H -1.09449998 -5.85489545 4.30907483

H 2.93972323 -5.97801322 3.29703211

H 2.64277873 -4.30754926 1.53474594

H -5.48422667 1.29061194 -1.35457724

H -7.42253818 0.86353228 -2.79500779

H -5.67116389 -2.55817445 -4.39192503

H -3.65578347 -2.27561212 -3.00294750

H -2.61368531 4.33830945 1.52558134

H -2.90385147 6.01476854 3.28587561

H 1.10996204 5.80617568 4.36208525

H 1.56828874 4.10441053 2.66548604

H 0.99033724 -8.06109794 4.37644660

H 2.16447531 -7.01373382 5.21970136

H 0.44386474 -6.94940547 5.66175212

H 8.44166289 1.91872764 -3.96120193

H 8.39962565 0.24574025 -4.58029907

H 7.52812200 1.53137788 -5.44556870

H -2.09703625 6.93538696 5.32605017

H -0.34499454 7.02528690 5.61658659

H -1.09070117 8.05435172 4.36467190

H -8.11999120 -2.10650099 -4.43614471

H -8.59152383 -0.40452101 -4.22216190

H -7.51350187 -0.86282591 -5.56640085

C 0.02262629 -0.03294348 1.83523987

O 0.98814010 -0.25439807 2.52566111

O -1.18227105 0.20576514 2.40994791

H -1.79341260 0.45871222 1.68802787

[CoP(COOH)]^+1^ M = 2

C 2.67831753 -1.14003295 -0.90484274

C 3.20303703 -2.42778326 -1.17148470

C 2.38435879 -3.35477400 -0.52803840

C 1.34064271 -2.63530605 0.08421544

N 1.49920964 -1.29319678 -0.16413675

C 3.22531054 0.14042373 -1.22742103

C 0.15980669 -3.17923844 0.75684749

C -1.09770750 -2.70936582 0.21817466

C -2.26709252 -3.47860195 -0.08243404

C -3.13374916 -2.65107329 -0.76158614

C -2.50984000 -1.36723632 -0.84753053

N -1.25534026 -1.43975796 -0.25013243

C -3.11566358 -0.17184296 -1.27415876

C -2.59303861 1.10519744 -1.00929418

C -3.22245399 2.37449520 -1.20853362

C -2.44289127 3.31363422 -0.56910879

C -1.30908125 2.62657818 -0.02977825

N -1.38944754 1.30693474 -0.33758415

C -0.16478893 3.20039939 0.65276905

C 1.12812521 2.71565577 0.19220601

C 2.30721796 3.46639857 -0.03875958

C 3.22150979 2.61517168 -0.64329109

C 2.60060886 1.34518928 -0.77781854

N 1.31356320 1.43102490 -0.25487564

C -0.30790516 4.18043917 1.64654870

C -4.41909978 -0.28095858 -1.96545146

C 0.25272295 -4.15373523 1.75620416

C 4.46620687 0.24854199 -1.96260183

C -4.54633526 -1.07093992 -3.12460171

C -5.75688541 -1.16122902 -3.77889046

N -6.84851294 -0.50074821 -3.31822540

C -6.76655518 0.25262005 -2.19623641

C -5.57516832 0.37657720 -1.50874224

C 0.80596816 4.72404214 2.39490311

C 0.64411951 5.69254300 3.33769376

N -0.59001629 6.19978967 3.66440267

C -1.69115458 5.65175869 3.05134866

C -1.58805348 4.67744342 2.10617108

C 5.59242823 -0.60963121 -1.75995604

C 6.74461842 -0.46651061 -2.48401843

N 6.87277695 0.49603933 -3.45135676

C 5.81708574 1.34519144 -3.68004234

C 4.65778610 1.25495421 -2.96070485

C 1.51580854 -4.66870300 2.24239121

C 1.58360735 -5.63229952 3.20068150

N 0.46043499 -6.15896202 3.79392432

C -0.76105075 -5.64760913 3.42542039

C -0.88899070 -4.68424394 2.47274119

C -0.72227979 7.33824446 4.57375965

C -8.13644283 -0.64626358 -4.03300651

C 0.55650596 -7.28954247 4.71734279

C 8.07598130 0.57357678 -4.28670627

Co 0.03017902 0.00575371 -0.08668775

H 4.07044370 -2.65500217 -1.77300646

H 2.48751784 -4.43197992 -0.52964821

H -2.39800964 -4.53089465 0.12726460

H -4.11139010 -2.90680849 -1.14656703

H -4.13358820 2.55621865 -1.76082201

H -2.60414663 4.38209110 -0.53020612

H 2.43532721 4.52125114 0.16097715

H 4.23081529 2.85987547 -0.94315323

H -3.69146019 -1.60208564 -3.52426744

H -5.89286143 -1.74486713 -4.68055898

H -7.67784319 0.73693113 -1.87058706

H -5.55312372 0.97294805 -0.60576767

H 1.80545726 4.34275830 2.23632888

H 1.48210577 6.10163240 3.89015413

H -2.65099634 6.03260187 3.38093015

H -2.50845092 4.26359225 1.71668401

H 5.57718694 -1.35501793 -0.97586585

H 7.61535924 -1.08794826 -2.31437253

H 5.95619622 2.07306877 -4.46978429

H 3.85512014 1.94348666 -3.19476033

H 2.44621023 -4.28114677 1.84775541

H 2.52952820 -6.02758507 3.55267804

H -1.61718857 -6.04759452 3.95636464

H -1.88122876 -4.29560144 2.28491316

H -0.69113672 8.28434862 4.02223170

H -1.67088075 7.26760254 5.11011303

H 0.09356129 7.32041062 5.29928149

H -8.56222616 -1.62489952 -3.80316725

H -8.81509124 0.14090889 -3.70879250

H -7.95837848 -0.55676384 -5.10482500

H 1.49682173 -7.22962715 5.26947866

H -0.27100858 -7.24942791 5.42874109

H 0.51808968 -8.24148491 4.17619300

H 8.31167313 1.62015156 -4.48904143

H 8.91279107 0.11724105 -3.75640389

H 7.91638368 0.04701808 -5.23300165

C -0.01278791 0.05667858 1.79743410

O -0.96870514 0.33492951 2.48523840

O 1.17561585 -0.23870503 2.38710111

H 1.76208072 -0.54013075 1.66006021

[CoP(COOH)]^+1^ M = 4

C 2.59957496 -1.14397312 -0.91199263

C 3.11835984 -2.43871831 -1.19293383

C 2.32844062 -3.35819914 -0.51779300

C 1.28878664 -2.63427689 0.11299534

N 1.43657028 -1.29404667 -0.15800178

C 3.15428898 0.13491531 -1.22528931

C 0.13048211 -3.17263609 0.80167313

C -1.14495782 -2.67200592 0.36128834

C -2.35707345 -3.39959212 0.18602237

C -3.23364934 -2.57099125 -0.48738725

C -2.56774846 -1.32672884 -0.70251649

N -1.29448031 -1.41436876 -0.18256167

C -3.16879404 -0.13199079 -1.22424870

C -2.61576149 1.14984550 -0.91776725

C -3.17383890 2.44267250 -1.16069942

C -2.38632238 3.36129993 -0.49139062

C -1.30762480 2.64115311 0.09228969

N -1.43249615 1.31270609 -0.21233974

C -0.15118547 3.19423285 0.76581216

C 1.12724076 2.69515394 0.32564481

C 2.33387602 3.42373372 0.13211005

C 3.21491543 2.58374269 -0.52377979

C 2.55501466 1.33479457 -0.71465816

N 1.27964458 1.42778470 -0.19139982

C -0.28181623 4.21408177 1.74599729

C -4.38722052 -0.26844403 -1.98788727

C 0.26319138 -4.19017703 1.78359150

C 4.37269480 0.25523722 -1.99138529

C -4.54838261 -1.32756137 -2.93671853

C -5.67768566 -1.44838788 -3.69770917

N -6.72821453 -0.57409298 -3.56764609

C -6.63915073 0.42542879 -2.63777845

C -5.51673418 0.60073970 -1.87356713

C 0.82947783 4.72308549 2.50701586

C 0.68020587 5.71805953 3.42912783

N -0.54177908 6.27269122 3.70752552

C -1.64607894 5.75606740 3.07969789

C -1.55107590 4.75818575 2.15364677

C 5.49061738 -0.62724975 -1.86610024

C 6.61834501 -0.47006855 -2.62585347

N 6.72785281 0.53062700 -3.55287905

C 5.68405202 1.40763783 -3.70268967

C 4.54850145 1.30428531 -2.94713688

C 1.53051546 -4.75653487 2.16390050

C 1.62706335 -5.75031164 3.09446534

N 0.52744134 -6.24066534 3.75054877

C -0.69017309 -5.66697936 3.49418052

C -0.84142156 -4.67523434 2.56875417

C -0.66100861 7.42098138 4.61083023

C -7.94558803 -0.77108138 -4.36407356

C 0.64637290 -7.38393776 4.66075280

C 7.90321749 0.61945871 -4.42837791

Co -0.01539547 0.01168062 -0.02232969

H 3.96195764 -2.66950754 -1.82519070

H 2.42844933 -4.43511134 -0.52978664

H -2.52124531 -4.43462225 0.45003072

H -4.24586923 -2.80572153 -0.78451519

H -4.03712779 2.66743584 -1.76739557

H -2.50426624 4.43629744 -0.48159708

H 2.49443691 4.46476611 0.37371229

H 4.22636207 2.81681269 -0.82486588

H -3.74190595 -2.03100279 -3.10405245

H -5.79255304 -2.21848130 -4.45054347

H -7.51282895 1.05645450 -2.53087165

H -5.53550248 1.38177091 -1.12584595

H 1.81632303 4.29804137 2.39040810

H 1.51702664 6.10656628 3.99690047

H -2.59898358 6.17929294 3.37414223

H -2.47286668 4.37066596 1.74272687

H 5.49495420 -1.40303951 -1.11237413

H 7.48340396 -1.11247999 -2.51592545

H 5.80841643 2.16991957 -4.46174130

H 3.75209831 2.01723620 -3.12169744

H 2.44929773 -4.39246780 1.72530390

H 2.57757662 -6.19203932 3.36868727

H -1.52149022 -6.03719223 4.08186263

H -1.82262129 -4.23288586 2.46902452

H -0.60473444 8.35782829 4.04736029

H -1.61658060 7.37186634 5.13613093

H 0.14797086 7.39090059 5.34259492

H -8.53093236 -1.60211483 -3.95909741

H -8.54384598 0.13980137 -4.34035717

H -7.67178829 -0.98968561 -5.39850897

H 1.60874137 -7.34084242 5.17399212

H -0.15327716 -7.34032460 5.40188319

H 0.57361264 -8.32376256 4.10431153

H 8.11181905 1.66745239 -4.64916884

H 8.76467553 0.18435885 -3.92000039

H 7.72047534 0.07896382 -5.36215113

C -0.00774414 0.03667516 1.86520732

O -0.94735233 0.29366254 2.58262249

O 1.19876868 -0.24982702 2.42569279

H 1.78223895 -0.52048286 1.68714524

[CoP(COOH)]^+1^ M = 6

C -2.88804191 0.59223810 -0.88204763

C -3.70521881 1.69948485 -1.29914350

C -3.08065291 2.84200266 -0.85551992

C -1.87162128 2.42572044 -0.19573149

N -1.76022182 1.07779491 -0.24346332

C -3.16292329 -0.78222795 -1.06350288

C -0.84072173 3.28406499 0.37459924

C 0.54198272 3.01796837 0.04181310

C 1.58706195 3.98331803 -0.12600214

C 2.70157001 3.29799906 -0.56133192

C 2.33564491 1.91583851 -0.66416886

N 1.01914152 1.77302992 -0.29234249

C 3.21838630 0.83348872 -1.02678440

C 2.89435760 -0.54879922 -0.83504084

C 3.64506794 -1.67472041 -1.31785305

C 3.01307078 -2.81084193 -0.85763789

C 1.85441230 -2.37192936 -0.14145386

N 1.78143922 -1.01261571 -0.15522762

C 0.81122416 -3.23328473 0.41246409

C -0.56059800 -2.96371108 0.03536347

C -1.58439870 -3.94158872 -0.25593769

C -2.66759495 -3.25303960 -0.73570504

C -2.31437460 -1.85515034 -0.72359575

N -1.01977792 -1.71947205 -0.24954916

C 1.15674732 -4.35324252 1.22634758

C 4.50450671 1.16975799 -1.59802233

C -1.21185184 4.36057300 1.23862334

C -4.48293618 -1.12794088 -1.64489163

C 4.66865940 2.21045809 -2.56904234

C 5.88667325 2.50231211 -3.11550350

N 7.02413052 1.82560326 -2.74535690

C 6.92064819 0.83786466 -1.79537563

C 5.71719928 0.49511533 -1.24490016

C 0.18578829 -5.15784949 1.91801041

C 0.54729498 -6.22234254 2.69421222

N 1.85627214 -6.56720064 2.88558238

C 2.82214610 -5.78131720 2.32293119

C 2.51507951 -4.70331986 1.54104951

C -5.68406555 -0.67090679 -1.07018173

C -6.89787661 -1.00521458 -1.63347147

N -6.96056834 -1.77061032 -2.75135254

C -5.82499047 -2.22369503 -3.33278341

C -4.58667358 -1.92080793 -2.80124533

C -2.57542010 4.70591213 1.53504464

C -2.89828458 5.74885774 2.35756045

N -1.94335103 6.49766287 2.98697178

C -0.63256216 6.14562098 2.82180498

C -0.25758362 5.11880005 2.00207101

C 2.20589326 -7.76996165 3.65200319

C 8.33385446 2.22075375 -3.27046663

C -2.30745103 7.66668309 3.79700597

C -8.28381594 -2.07804229 -3.33952546

Co -0.00869642 0.06515241 0.32263568

H -4.61511934 1.64367351 -1.88066742

H -3.38923368 3.86350516 -1.03229905

H 1.49740257 5.05433428 -0.01026149

H 3.67147894 3.72363994 -0.77486426

H 4.51408556 -1.63821606 -1.95928471

H 3.27831469 -3.83901824 -1.06682882

H -1.48104950 -5.01521004 -0.18675090

H -3.60783409 -3.67580879 -1.06107291

H 3.80518728 2.75523984 -2.93063155

H 6.01354898 3.25917183 -3.87985286

H 7.84900608 0.36314005 -1.50207495

H 5.70663269 -0.27650697 -0.48520473

H -0.86590708 -4.91333539 1.87532488

H -0.18451861 -6.83259738 3.20965680

H 3.84631374 -6.05745209 2.54226241

H 3.33671070 -4.10561556 1.17249015

H -5.66979868 -0.06564034 -0.17262457

H -7.84470415 -0.68660443 -1.21597717

H -5.94239538 -2.82060515 -4.22763097

H -3.69864707 -2.29332499 -3.29628622

H -3.39461945 4.13230418 1.12692375

H -3.92659313 6.02161470 2.56113270

H 0.08857928 6.71762544 3.39310714

H 0.79181950 4.86390625 1.97969108

H 2.14151019 -8.65575348 3.01323638

H 3.22262430 -7.67026916 4.03332123

H 1.51864209 -7.87630864 4.49331099

H 8.79591228 2.97362340 -2.62317896

H 8.98221028 1.34459724 -3.32616155

H 8.21150450 2.63400137 -4.27319064

H -3.32633677 7.54562252 4.16654528

H -1.62700013 7.74473853 4.64679861

H -2.24444824 8.57742494 3.19405266

H -8.16386246 -2.83616099 -4.11147562

H -8.94114220 -2.45433779 -2.55506943

H -8.69966090 -1.16687596 -3.77350679

C 0.02549168 -0.09272119 2.39346486

O 0.43621545 -1.29741341 2.98342182

O -0.27134926 0.78251529 3.20519459

H 0.64808950 -1.89768770 2.24547891

[CoP(COOH)]^0^ M = 1

C 2.59846037 -1.15263932 -0.96642194

C 3.09620413 -2.44513863 -1.26339875

C 2.29299135 -3.36708045 -0.59035028

C 1.28346615 -2.63887634 0.06708613

N 1.44870177 -1.29924351 -0.18172067

C 3.15369574 0.12953244 -1.27757362

C 0.12722934 -3.15505697 0.80308633

C -1.15523673 -2.68461452 0.29929024

C -2.34616176 -3.42199721 0.08766646

C -3.22911359 -2.58519744 -0.58461663

C -2.57629176 -1.33731048 -0.77144440

N -1.30520191 -1.42382450 -0.21879727

C -3.17039217 -0.13630924 -1.27824373

C -2.61784496 1.14422982 -0.96447068

C -3.15273746 2.43651881 -1.22153173

C -2.35214297 3.35561725 -0.55180428

C -1.30433829 2.62753868 0.05936884

N -1.44620959 1.30064134 -0.22303501

C -0.14676042 3.16125340 0.77884877

C 1.13565858 2.68466913 0.28864018

C 2.33168208 3.41747135 0.08087380

C 3.21549636 2.57565457 -0.58023117

C 2.56093089 1.32762714 -0.76619245

N 1.28520710 1.41945024 -0.21964010

C -0.27977382 4.10374497 1.79733261

C -4.38614164 -0.25910631 -2.05077663

C 0.26271146 -4.08305918 1.83102527

C 4.36884121 0.24603988 -2.04973741

C -4.55150138 -1.30450804 -3.01658619

C -5.67666722 -1.40925749 -3.78397609

N -6.72738299 -0.52896766 -3.64695675

C -6.63556885 0.45639618 -2.69545488

C -5.51481664 0.61452611 -1.92825472

C 0.83478993 4.61354767 2.57795022

C 0.67361520 5.55422617 3.54534015

N -0.56048269 6.07076945 3.87535033

C -1.66278589 5.56026281 3.22304492

C -1.56139636 4.61655352 2.25071574

C 5.48371774 -0.64604919 -1.93381392

C 6.60969516 -0.49452410 -2.69425717

N 6.72617026 0.51144026 -3.62094890

C 5.68238391 1.39626791 -3.76464201

C 4.55098906 1.29778198 -3.00462697

C 1.54594632 -4.59808191 2.27897820

C 1.65025326 -5.52505203 3.26641161

N 0.55054388 -6.01640884 3.93892252

C -0.68490801 -5.50335452 3.60607528

C -0.85004315 -4.57984557 2.62356005

C -0.68487250 7.19945762 4.79355156

C -7.94838113 -0.71618223 -4.43449819

C 0.67681904 -7.13118140 4.87345055

C 7.89133195 0.58733125 -4.50655855

Co -0.01949724 0.00173195 -0.04317242

H 3.93650312 -2.67931030 -1.89990704

H 2.38697382 -4.44543732 -0.59522990

H -2.50172719 -4.46014215 0.34869713

H -4.23739558 -2.82304389 -0.89476737

H -4.01241584 2.66685982 -1.83256589

H -2.46265052 4.43215138 -0.53382129

H 2.48884818 4.45709865 0.33401380

H 4.22577624 2.81008668 -0.88614535

H -3.74749863 -2.00993372 -3.18781048

H -5.79240050 -2.16762504 -4.54863509

H -7.50793914 1.08818036 -2.58081659

H -5.52793202 1.38645905 -1.17056154

H 1.83060689 4.22015687 2.42140968

H 1.50892793 5.93341825 4.12342390

H -2.62060163 5.95110620 3.54805962

H -2.47801888 4.23826981 1.81641516

H 5.48226582 -1.42594664 -1.18405094

H 7.47129104 -1.14255471 -2.58863811

H 5.80892756 2.15960878 -4.52246362

H 3.75823959 2.01667000 -3.17169343

H 2.45984990 -4.23619731 1.82451803

H 2.60825427 -5.91829137 3.58810581

H -1.51767164 -5.87173522 4.19481948

H -1.84607427 -4.18870592 2.46129646

H -0.62488322 8.15532706 4.25952477

H -1.64401914 7.14321905 5.31372727

H 0.11673768 7.15665924 5.53456545

H -8.55994358 -1.51902739 -4.00942174

H -8.52247390 0.21109089 -4.43809069

H -7.68364960 -0.97126427 -5.46328854

H 1.63879056 -7.06971050 5.38788444

H -0.12056313 -7.07472441 5.61815065

H 0.61138884 -8.09544428 4.35515058

H 8.10931328 1.63294134 -4.73184302

H 8.75485123 0.14561170 -4.00645120

H 7.69935535 0.04796179 -5.44002417

C -0.00216129 0.00413164 1.84087397

O -0.93847902 0.22295227 2.57748730

O 1.22081712 -0.25635595 2.38305376

H 1.78607464 -0.52471902 1.62724456

[CoP(COOH)]^0^ M = 3

C 2.59767579 -1.15197929 -0.96680233

C 3.09634406 -2.44430203 -1.26416998

C 2.29376589 -3.36670755 -0.59128001

C 1.28469076 -2.63902143 0.06776557

N 1.44886906 -1.29952552 -0.18108007

C 3.15092641 0.13043951 -1.27935697

C 0.12895293 -3.15519874 0.80506974

C -1.15210649 -2.68880099 0.29290401

C -2.33847393 -3.43023872 0.07037442

C -3.22204178 -2.59328718 -0.60081824

C -2.57329148 -1.34153771 -0.77767446

N -1.30437336 -1.42622059 -0.21956303

C -3.16912535 -0.13924602 -1.27873403

C -2.61871980 1.14095627 -0.96122814

C -3.15457833 2.43346563 -1.21702029

C -2.35430386 3.35222799 -0.54697682

C -1.30619870 2.62407668 0.06406790

N -1.44733691 1.29735406 -0.21958894

C -0.14877781 3.15765868 0.78380621

C 1.13203041 2.68425867 0.28604558

C 2.32407228 3.42111757 0.06854281

C 3.20781181 2.58032289 -0.59332579

C 2.55682444 1.32874786 -0.77129680

N 1.28346172 1.41803964 -0.21869310

C -0.27965536 4.09916124 1.80297672

C -4.38511807 -0.25998629 -2.05182842

C 0.26283979 -4.07985263 1.83498949

C 4.36656958 0.24634999 -2.05173589

C -4.54806443 -1.29923062 -3.02406476

C -5.67391074 -1.40178426 -3.79114216

N -6.72620360 -0.52541571 -3.64720450

C -6.63624075 0.45420421 -2.69021049

C -5.51519702 0.61005502 -1.92255518

C 0.83736782 4.61236660 2.57852674

C 0.67819083 5.55339676 3.54554453

N -0.55626241 6.06778120 3.88056949

C -1.66052479 5.55385933 3.23280881

C -1.56088101 4.60983878 2.26095749

C 5.48286536 -0.64233372 -1.93014407

C 6.60939179 -0.49155557 -2.69034259

N 6.72391842 0.51023893 -3.62120564

C 5.67864011 1.39146669 -3.77043034

C 4.54625817 1.29354371 -3.01136696

C 1.54619467 -4.59101067 2.28868537

C 1.64929206 -5.51579061 3.27784780

N 0.54791481 -6.00973568 3.94722350

C -0.68838981 -5.50133854 3.60739648

C -0.85226893 -4.58004146 2.62303861

C -0.67869012 7.20010961 4.79399326

C -7.94709626 -0.70956838 -4.43631623

C 0.67300155 -7.12621941 4.87929666

C 7.89101496 0.58602372 -4.50484006

Co -0.01991338 -0.00038546 -0.04093106

H 3.93713758 -2.67760414 -1.90038899

H 2.38868278 -4.44499951 -0.59534228

H -2.49096349 -4.47002603 0.32687332

H -4.22855912 -2.83298122 -0.91530533

H -4.01476063 2.66370295 -1.82742004

H -2.46567389 4.42864566 -0.52714856

H 2.47857208 4.46192533 0.31857162

H 4.21615640 2.81704316 -0.90382635

H -3.74239802 -2.00140531 -3.20055780

H -5.78842903 -2.15554268 -4.56050316

H -7.50990929 1.08334953 -2.57122591

H -5.52906074 1.37748958 -1.16031319

H 1.83345444 4.22105505 2.41766475

H 1.51489394 5.93490144 4.12015853

H -2.61786197 5.94248332 3.56197061

H -2.47829107 4.22897370 1.83029601

H 5.48192694 -1.41920544 -1.17723791

H 7.47236052 -1.13711999 -2.58117674

H 5.80414642 2.15161142 -4.53160970

H 3.75165648 2.00928805 -3.18291940

H 2.46063026 -4.22714001 1.83655950

H 2.60712755 -5.90546026 3.60448362

H -1.52242701 -5.87192596 4.19303667

H -1.84902493 -4.19296751 2.45464911

H -0.62034483 8.15425851 4.25644733

H -1.63657556 7.14585980 5.31683297

H 0.12464215 7.16073154 5.53344844

H -8.55622177 -1.51706067 -4.01684615

H -8.52321765 0.21637871 -4.43265948

H -7.68161707 -0.95626323 -5.46689421

H 1.63337408 -7.06455891 5.39683547

H -0.12654287 -7.07264368 5.62201443

H 0.61058185 -8.09000965 4.35942901

H 8.10554332 1.63128444 -4.73470273

H 8.75487311 0.14978443 -4.00063340

H 7.70239789 0.04128593 -5.43575752

C -0.00237864 -0.00025595 1.84313299

O -0.93955168 0.21302532 2.58032990

O 1.22209124 -0.25514568 2.38504827

H 1.78859470 -0.52030227 1.62921450

[CoP(COOH)]^0^ M = 5

C 2.67241739 -1.04397556 -0.90869553

C 3.26741006 -2.31046596 -1.20460164

C 2.49946584 -3.27697071 -0.58129434

C 1.40401449 -2.60520928 0.03241274

N 1.49771097 -1.26128399 -0.19837868

C 3.17664100 0.25935306 -1.21201387

C 0.25164222 -3.23224487 0.65933068

C -1.04688736 -2.78962062 0.21174211

C -2.21855520 -3.58353516 -0.00009271

C -3.15109425 -2.76692055 -0.60513242

C -2.55093063 -1.47242742 -0.74631523

N -1.27051140 -1.51783489 -0.24737791

C -3.22148597 -0.27883713 -1.17929692

C -2.70701549 1.02006365 -0.87114413

C -3.30454734 2.28896769 -1.16200502

C -2.53352306 3.25239770 -0.54315590

C -1.43287708 2.57571526 0.06265362

N -1.52698783 1.23507444 -0.17309668

C -0.27851592 3.20231110 0.68416664

C 1.01559418 2.75593302 0.23237026

C 2.18960880 3.54722826 0.02394928

C 3.11508768 2.73464591 -0.59539745

C 2.50853128 1.44544837 -0.75175342

N 1.23118241 1.48729230 -0.24341333

C -0.43891614 4.25245253 1.62748574

C -4.47382765 -0.42309373 -1.88648927

C 0.41899256 -4.26676305 1.61334324

C 4.41598228 0.41944927 -1.93748973

C -4.65950773 -1.43359858 -2.88663086

C -5.82286748 -1.54754452 -3.59292807

N -6.89318561 -0.71189983 -3.36157155

C -6.78042524 0.23685061 -2.37382226

C -5.62419580 0.40218278 -1.66390063

C 0.65832424 4.83887341 2.35592070

C 0.48015905 5.86964699 3.23147839

N -0.75999939 6.39140927 3.49807785

C -1.84954463 5.79862655 2.91165118

C -1.72490663 4.76159772 2.03390013

C 5.57518352 -0.40076675 -1.74472306

C 6.72385035 -0.20609618 -2.45991118

N 6.82497359 0.77774655 -3.41265147

C 5.73778514 1.59295562 -3.63187765

C 4.58060821 1.44956461 -2.92020599

C 1.71083152 -4.75484107 2.03390120

C 1.84517501 -5.76636418 2.93767864

N 0.76030607 -6.35796319 3.53835723

C -0.48682466 -5.85947187 3.25096373

C -0.67484555 -4.85453380 2.34967675

C -0.91025847 7.58296651 4.33750455

C -8.14995492 -0.90603048 -4.08799149

C 0.92285493 -7.52247582 4.40963322

C 8.02340970 0.90154436 -4.24617565

Co -0.02297858 -0.01432833 0.04709926

H 4.13808411 -2.48480578 -1.81887570

H 2.64209653 -4.34907996 -0.61730821

H -2.31808494 -4.64134178 0.20085673

H -4.15555329 -3.03801677 -0.89867751

H -4.17823396 2.46501145 -1.77132373

H -2.67362941 4.32455847 -0.57932663

H 2.29512789 4.60227204 0.23428795

H 4.11828055 3.00661763 -0.89201012

H -3.84256550 -2.09912609 -3.13871505

H -5.95485178 -2.27653435 -4.38320974

H -7.66542302 0.83194538 -2.18408648

H -5.61776992 1.14501479 -0.87694422

H 1.65918333 4.44525680 2.24938397

H 1.30664868 6.31577607 3.77167033

H -2.81538976 6.19686909 3.19880174

H -2.63516242 4.31820251 1.65480433

H 5.58542683 -1.15641979 -0.97013191

H 7.61627056 -0.79735819 -2.29401832

H 5.85384041 2.33898909 -4.40847078

H 3.75609815 2.11414773 -3.14841283

H 2.61631577 -4.30868165 1.64598050

H 2.81506912 -6.14345430 3.24001241

H -1.30959048 -6.30141906 3.80052606

H -1.68015619 -4.47630387 2.22855804

H -0.84647484 8.49104501 3.72922221

H -1.87874617 7.55308025 4.83996935

H -0.12046134 7.59791269 5.09093124

H -8.72837728 -1.72458626 -3.64628342

H -8.73682436 0.01248786 -4.04751828

H -7.93463746 -1.14021111 -5.13307226

H 1.88737991 -7.46438236 4.91784625

H 0.12912756 -7.52905893 5.15942175

H 0.87699619 -8.45019658 3.82899928

H 8.20929774 1.95520844 -4.46354872

H 8.88132724 0.49534535 -3.70792761

H 7.89564965 0.35511764 -5.18667073

C 0.07855989 -0.04286365 2.13332640

O -0.75107898 0.09174859 3.03276906

O 1.40162448 -0.24820824 2.62700063

H 1.91108126 -0.40419739 1.81151886

[CoP(COOH)]^–1^ M = 4

C 2.67117996 -0.98835161 -0.93515993

C 3.24863946 -2.25103713 -1.25421329

C 2.47627321 -3.22322436 -0.63236673

C 1.41140687 -2.55202111 0.02058552

N 1.51215808 -1.20827729 -0.18579725

C 3.16928620 0.31349454 -1.24917619

C 0.26802663 -3.17180290 0.69968479

C -1.03900642 -2.75896343 0.20187193

C -2.18217071 -3.57407283 -0.04884092

C -3.12901394 -2.76378516 -0.65180438

C -2.55632266 -1.45949748 -0.76542389

N -1.27676877 -1.48925490 -0.23145474

C -3.22997121 -0.27642867 -1.20022758

C -2.73189380 1.03287666 -0.90518313

C -3.32558623 2.28890772 -1.22342571

C -2.54322972 3.27168909 -0.62959723

C -1.46019045 2.60972863 0.00393437

N -1.55843071 1.26682332 -0.18759049

C -0.30425719 3.23738592 0.65865566

C 0.99410906 2.82059010 0.13900954

C 2.13720543 3.62786456 -0.12689770

C 3.07868973 2.80635188 -0.72718944

C 2.49920604 1.50547414 -0.82521477

N 1.22238926 1.54525397 -0.28470395

C -0.46059627 4.20266441 1.64531812

C -4.48029024 -0.43485356 -1.91701043

C 0.44345624 -4.15059990 1.67394120

C 4.41950380 0.45243344 -1.96875062

C -4.64754290 -1.43937691 -2.92732925

C -5.81202507 -1.58035900 -3.62551566

N -6.90547598 -0.77218090 -3.38011404

C -6.80596900 0.17523203 -2.38295719

C -5.64761888 0.36491187 -1.68417715

C 0.64497882 4.81845800 2.36940063

C 0.45536851 5.78110035 3.30656530

N -0.80127733 6.23017687 3.66742977

C -1.89061372 5.62291973 3.06905705

C -1.75984473 4.65271599 2.12988972

C 5.57553352 -0.36152115 -1.72752847

C 6.73191314 -0.20523560 -2.43694512

N 6.84847218 0.73922387 -3.43491218

C 5.76121974 1.54773926 -3.70069066

C 4.59832094 1.43978147 -2.99304330

C 1.74917044 -4.60912602 2.12515415

C 1.89503976 -5.60309551 3.03876102

N 0.81682576 -6.22140899 3.63990597

C -0.44316735 -5.75847602 3.32003579

C -0.64815953 -4.77190230 2.40970982

C -0.96619305 7.40129027 4.51869431

C -8.17581631 -1.01769644 -4.06085850

C 0.99466668 -7.40862862 4.46967484

C 8.03938427 0.79189883 -4.28156287

Co -0.03121998 0.03186410 0.05465030

H 4.11407439 -2.42299109 -1.87782559

H 2.61567403 -4.29645865 -0.66657900

H -2.26230911 -4.63446282 0.15091723

H -4.12568198 -3.05015346 -0.95873458

H -4.20796516 2.44881226 -1.82618674

H -2.69052188 4.34412324 -0.66684716

H 2.22296290 4.68982992 0.06351240

H 4.07542080 3.08411376 -1.04230947

H -3.81561555 -2.08357749 -3.18598153

H -5.93213165 -2.30861873 -4.41838764

H -7.70490765 0.74563740 -2.18225838

H -5.64728761 1.10756856 -0.89642309

H 1.65823902 4.48266083 2.18988456

H 1.28255175 6.23307374 3.84346825

H -2.86055440 5.96141020 3.41727828

H -2.66273328 4.19825921 1.74102306

H 5.56788765 -1.08827596 -0.92510567

H 7.62013045 -0.79262932 -2.23728722

H 5.88730416 2.25734438 -4.50936609

H 3.77749519 2.09689396 -3.25468616

H 2.64661330 -4.14906603 1.73076553

H 2.87040119 -5.95377206 3.35784922

H -1.26115008 -6.22146723 3.86114625

H -1.66356269 -4.42896111 2.25948584

H -0.97080956 8.33043171 3.93393344

H -1.90927451 7.32610102 5.06635950

H -0.14812225 7.44592034 5.24245936

H -8.75678425 -1.78794323 -3.54035702

H -8.75555775 -0.09330864 -4.09430979

H -7.98314544 -1.34726143 -5.08433978

H 1.95165136 -7.34838080 4.99376150

H 0.19411693 -7.45933229 5.21190162

H 0.97755127 -8.32496460 3.86689095

H 8.21175863 1.82035825 -4.60600552

H 8.90627264 0.45356659 -3.71036260

H 7.91997346 0.15224005 -5.16360907

C 0.10966022 0.03748733 2.14071209

O -0.67765079 0.23448206 3.06875276

O 1.44230958 -0.20608861 2.59327931

H 1.90170850 -0.42720827 1.76126720

[CoP(COOH)]^–1^ M = 6

C 2.65340272 -0.95420530 -0.91836463

C 3.33905050 -2.18795141 -1.16009192

C 2.59258578 -3.19122118 -0.56068287

C 1.43572029 -2.57992420 -0.00017282

N 1.47095030 -1.22120257 -0.24749252

C 3.13642821 0.35113016 -1.19476881

C 0.29934802 -3.23711489 0.60943149

C -1.01075620 -2.78522872 0.19297631

C -2.16174127 -3.59903439 -0.05560270

C -3.12054755 -2.78161556 -0.61492505

C -2.55814651 -1.46647367 -0.69753676

N -1.27221474 -1.49829910 -0.20063219

C -3.26263511 -0.28019718 -1.08883386

C -2.77386662 1.02637421 -0.77309829

C -3.41028769 2.28087034 -1.03946239

C -2.64199127 3.25987756 -0.44128610

C -1.50459217 2.60838364 0.12564383

N -1.57842874 1.26517526 -0.10863593

C -0.33964090 3.27513425 0.68281246

C 0.94451312 2.83891652 0.20627538

C 2.07617278 3.65572667 -0.10810677

C 3.00177657 2.84431499 -0.73545998

C 2.45089825 1.51805631 -0.76159054

N 1.18760154 1.54405134 -0.21146058

C -0.49108726 4.35820215 1.59522073

C -4.52408678 -0.44067287 -1.78062228

C 0.46607653 -4.31800412 1.51966678

C 4.44849315 0.51057285 -1.87238124

C -4.69862342 -1.42226714 -2.81273840

C -5.87534159 -1.56253798 -3.49024721

N -6.97378313 -0.77807535 -3.20274372

C -6.86746919 0.14573810 -2.18669559

C -5.69847889 0.33527017 -1.50542467

C 0.62448222 5.00462528 2.24986484

C 0.46369284 6.06063154 3.09415500

N -0.78174109 6.56617274 3.40585253

C -1.88571798 5.90925353 2.89943121

C -1.77076035 4.84495998 2.05693353

C 5.67427073 0.02852087 -1.31957760

C 6.87237621 0.17610130 -1.96140902

N 6.96827976 0.85776123 -3.18461005

C 5.77467883 1.31145129 -3.76891018

C 4.57147376 1.16245936 -3.13688598

C 1.75931373 -4.81079900 1.93454259

C 1.89927733 -5.87918941 2.76862359

N 0.81455858 -6.53481972 3.30578153

C -0.43721059 -6.02385067 3.04482354

C -0.62550727 -4.95894399 2.21548726

C -0.92328943 7.80579249 4.16243942

C -8.25453281 -1.02155336 -3.86629229

C 0.97702490 -7.77519983 4.06200470

C 8.14754276 0.67410314 -4.01643984

Co -0.04289106 0.01933459 0.07069748

H 4.27103514 -2.29247171 -1.69886892

H 2.79561218 -4.25427371 -0.58087843

H -2.23015103 -4.66782189 0.09172040

H -4.11801385 -3.06836858 -0.91654237

H -4.30737726 2.43678452 -1.62025019

H -2.80728509 4.32884145 -0.47220848

H 2.13855452 4.72762783 0.02631161

H 3.96518224 3.12673693 -1.13842699

H -3.86355411 -2.04635727 -3.10799912

H -6.00052757 -2.27183755 -4.29929339

H -7.77018433 0.69733233 -1.95315991

H -5.69645684 1.05559396 -0.69729000

H 1.62851378 4.62852822 2.10684068

H 1.30215554 6.54259820 3.58397761

H -2.84747086 6.28094359 3.23364037

H -2.68427062 4.35225534 1.75229138

H 5.66432455 -0.47917325 -0.35923250

H 7.80983981 -0.18822646 -1.55703056

H 5.88384298 1.80360087 -4.72850053

H 3.68365411 1.56098564 -3.62053248

H 2.66184860 -4.31876163 1.59873818

H 2.87115966 -6.25624259 3.06528316

H -1.25968288 -6.50839607 3.55810096

H -1.63325820 -4.57992067 2.11880489

H -0.91806131 8.67824776 3.49776418

H -1.86372859 7.78947084 4.71835870

H -0.09821471 7.89657386 4.87314881

H -8.82456991 -1.79689102 -3.34181635

H -8.83655140 -0.09821694 -3.88419370

H -8.07584130 -1.34353180 -4.89443959

H 1.95141118 -7.77589328 4.55497967

H 0.19713140 -7.84341612 4.82400361

H 0.90942558 -8.64716333 3.40168402

H 8.22985502 1.50358350 -4.72469065

H 9.04280126 0.66674260 -3.38797745

H 8.11085074 -0.26821222 -4.58434530

C 0.14412855 -0.03387528 2.17497233

O -0.59929852 0.19484579 3.13239482

O 1.47787822 -0.34678273 2.57658803

H 1.89843689 -0.56297321 1.72254530
