# Supplementary material for: Self-Assembled Liposomes Enhance Electron Transfer for Efficient Photocatalytic CO2 Reduction
Source: J Am Chem Soc. 2022 May 20;144(21):9399–412. doi: 10.1021/jacs.2c01725 (PMC9164230; doi:10.1021/jacs.2c01725)
Supplement: Supplementary file 2 — ja2c01725_si_002.zip [file ja2c01725_si_002.zip › Coordinates/CoP_H/Coord_CoP_H.docx]

[CoP(H)]^+4^  M = 1

C 2.5822738 -1.2666357 -0.8044474

C 3.1799028 -2.5733537 -0.9159074

C 2.3323878 -3.4508347 -0.3079244

C 1.1709728 -2.6973857 0.1117666

N 1.3273168 -1.3864737 -0.2412694

C 3.1911318 -0.0409207 -1.1061984

C -0.0435762 -3.2357237 0.6243916

C -1.2577052 -2.6527977 0.1698016

C -2.4842952 -3.3549157 -0.1424444

C -3.3134522 -2.4603007 -0.7503284

C -2.6267352 -1.1929177 -0.7641194

N -1.3624672 -1.3517727 -0.2410274

C -3.1978272 0.0450433 -1.0967694

C -2.5885942 1.2713073 -0.7980304

C -3.1865602 2.5779363 -0.9090374

C -2.3374332 3.4559923 -0.3040774

C -1.1744782 2.7030713 0.1123616

N -1.3319782 1.3917993 -0.2385834

C 0.0421648 3.2423263 0.6190736

C 1.2544808 2.6591503 0.1598886

C 2.4797328 3.3610073 -0.1583274

C 3.3072478 2.4652143 -0.7667074

C 2.6208238 1.1976663 -0.7753514

N 1.3580178 1.3573823 -0.2487384

C 0.0435498 4.3686853 1.5624906

C -4.5536922 0.0409793 -1.7047404

C -0.0411192 -4.3614267 1.5686526

C 4.5486188 -0.0399987 -1.7108044

C -4.7730372 -0.5952367 -2.9379634

C -6.0335772 -0.5947717 -3.5013714

N -7.0721692 0.0123503 -2.8788324

C -6.8946262 0.6245023 -1.6864304

C -5.6512852 0.6510173 -1.0806514

C 1.2335658 4.8416333 2.1734346

C 1.2132508 5.8790803 3.0774356

N 0.0511478 6.4663633 3.4521556

C -1.1175122 5.9905263 2.9643626

C -1.1481542 4.9531393 2.0578166

C 5.6426738 -0.6463067 -1.0774454

C 6.8890078 -0.6243297 -1.6773204

N 7.0722468 -0.0200927 -2.8725254

C 6.0368138 0.5827633 -3.5049844

C 4.7736668 0.5878983 -2.9474854

C 1.1525178 -4.9466917 2.0583406

C 1.1254828 -5.9841777 2.9648426

N -0.0411842 -6.4591567 3.4582346

C -1.2045892 -5.8706257 3.0895836

C -1.2284672 -4.8330607 2.1857416

C 0.0762468 7.6044683 4.3952016

C -8.4127782 -0.0311547 -3.5103814

C -0.0622632 -7.5985497 4.3998066

C 8.4095668 -0.0019757 -3.5118934

Co -0.0019852 0.0028153 -0.1381884

H 4.1152348 -2.8052217 -1.4032954

H 2.4416968 -4.5238527 -0.2641534

H -2.6590482 -4.4130247 -0.0210274

H -4.3028962 -2.6491927 -1.1404374

H -4.1232602 2.8094433 -1.3939554

H -2.4468642 4.5290193 -0.2604664

H 2.6543648 4.4194993 -0.0401004

H 4.2953348 2.6533673 -1.1606374

H -3.9609292 -1.0803637 -3.4655484

H -6.2483822 -1.0656427 -4.4521654

H -7.7675462 1.0783343 -1.2361464

H -5.5490812 1.1364193 -0.1181164

H 2.1917408 4.3832333 1.9843926

H 2.1168108 6.2604053 3.5359946

H -2.0228342 6.4567853 3.3307046

H -2.1232172 4.5895253 1.7745746

H 5.5355458 -1.1243847 -0.1117834

H 7.7594028 -1.0759737 -1.2202424

H 6.2571908 1.0446883 -4.4590464

H 3.9642998 1.0706273 -3.4814674

H 2.1264738 -4.5838137 1.7704586

H 2.0322088 -6.4513527 3.3265236

H -2.1061822 -6.2510587 3.5527306

H -2.1871022 -4.3736077 2.0015866

H 0.4528728 8.4875733 3.8751926

H -0.9338862 7.7889813 4.7567816

H 0.7274198 7.3569793 5.2342366

H -8.7994082 -1.0494347 -3.4437454

H -9.0752512 0.6549773 -2.9862114

H -8.3201462 0.2702543 -4.5540244

H 0.9483768 -7.7793367 4.7618816

H -0.7149972 -7.3548767 5.2386876

H -0.4348952 -8.4825247 3.8783666

H 8.7251898 1.0345963 -3.6370104

H 9.1157958 -0.5304217 -2.8745744

H 8.3420588 -0.4972237 -4.4813894

H 0.0006448 0.0040653 1.2883566

[CoP(H)]^+4^  M = 3

C 2.6695863 -1.0475110 -0.9250065

C 3.3035663 -2.3201280 -1.1621705

C 2.5117543 -3.2722370 -0.5858645

C 1.3752423 -2.5844610 -0.0172125

N 1.4487503 -1.2698510 -0.2723195

C 3.1842503 0.2181460 -1.1584095

C 0.2214093 -3.2016020 0.6387045

C -1.0466207 -2.7473530 0.1936535

C -2.2416147 -3.5309840 -0.0093755

C -3.1556437 -2.7126040 -0.6160355

C -2.5405057 -1.4201750 -0.7398125

N -1.2524927 -1.4723630 -0.2721085

C -3.1939867 -0.2242070 -1.1431235

C -2.6765447 1.0409000 -0.9101675

C -3.3080287 2.3155020 -1.1449345

C -2.5141137 3.2651430 -0.5676495

C -1.3778437 2.5744470 -0.0025615

N -1.4539057 1.2604090 -0.2598525

C -0.2210997 3.1897410 0.6497245

C 1.0442883 2.7372120 0.1957075

C 2.2371293 3.5222360 -0.0143945

C 3.1472183 2.7057960 -0.6295035

C 2.5321753 1.4130810 -0.7508225

N 1.2474403 1.4633880 -0.2740655

C -0.3708707 4.2189050 1.6469685

C -4.5365247 -0.3513330 -1.7679135

C 0.3761093 -4.2327890 1.6334035

C 4.5265073 0.3496150 -1.7836135

C -4.7012097 -1.0883420 -2.9518455

C -5.9496007 -1.1833020 -3.5349505

N -7.0249387 -0.5787610 -2.9753545

C -6.8999587 0.1209500 -1.8259725

C -5.6707747 0.2474620 -1.2029555

C 0.7337423 4.6922780 2.4115215

C 0.5771173 5.6701930 3.3629215

N -0.6422737 6.2011580 3.6403235

C -1.7371537 5.7251950 2.9913475

C -1.6349277 4.7529710 2.0266405

C 5.6659643 -0.2249130 -1.2050665

C 6.8987783 -0.0766180 -1.8167355

N 7.0204813 0.6181940 -2.9691435

C 5.9370243 1.1887550 -3.5485565

C 4.6856253 1.0739550 -2.9760215

C 1.6417903 -4.7684680 2.0053125

C 1.7486683 -5.7424920 2.9677115

N 0.6571023 -6.2188860 3.6218715

C -0.5633317 -5.6864380 3.3522125

C -0.7244507 -4.7066350 2.4034725

C -0.7791067 7.2993100 4.6167895

C -8.3476897 -0.7156510 -3.6317275

C 0.7988023 -7.3185860 4.5959135

C 8.3419493 0.7664310 -3.6254515

Co -0.0021987 -0.0048600 -0.1717275

H 4.2292493 -2.4766250 -1.6967865

H 2.6598553 -4.3420390 -0.5959655

H -2.3451457 -4.5867760 0.1937675

H -4.1544387 -2.9720670 -0.9356695

H -4.2332097 2.4755360 -1.6791415

H -2.6603867 4.3352290 -0.5752185

H 2.3411663 4.5777160 0.1901315

H 4.1432423 2.9666830 -0.9565135

H -3.8591747 -1.5739730 -3.4296475

H -6.1257107 -1.7292750 -4.4529365

H -7.8007277 0.5659640 -1.4245335

H -5.6081007 0.8040600 -0.2762645

H 1.7207293 4.2680630 2.2953115

H 1.4103563 6.0464400 3.9420655

H -2.6897017 6.1518980 3.2786855

H -2.5549197 4.4010100 1.5835395

H 5.6055123 -0.7741240 -0.2737795

H 7.8048103 -0.4986850 -1.4030295

H 6.1104673 1.7265020 -4.4719945

H 3.8382803 1.5389040 -3.4648625

H 2.5596993 -4.4164040 1.5581175

H 2.7025443 -6.1702990 3.2489615

H -1.3936767 -6.0629900 3.9352995

H -1.7116977 -4.2812950 2.2939275

H -0.7758347 8.2543680 4.0861315

H -1.7159867 7.1795030 5.1610255

H 0.0556053 7.2615690 5.3159475

H -8.6962937 -1.7423750 -3.5096985

H -9.0486337 -0.0256430 -3.1658225

H -8.2405827 -0.4751050 -4.6896495

H 1.7375703 -7.1986330 5.1368755

H -0.0333287 -7.2829700 5.2982415

H 0.7946383 -8.2727250 4.0636185

H 8.5555183 1.8288220 -3.7475765

H 9.1040333 0.3045230 -3.0007715

H 8.3073643 0.2728240 -4.5975255

H 0.0018443 -0.0074340 1.2539635

[CoP(H)]^+4^  M = 5

C 2.46839 -1.30561 -0.93077

C 3.04163 -2.62391 -1.05910

C 2.21728 -3.47931 -0.38394

C 1.11444 -2.68758 0.11387

N 1.25886 -1.39778 -0.26528

C 3.06702 -0.07997 -1.26893

C -0.05320 -3.14600 0.83141

C -1.31343 -2.62084 0.33180

C -2.53052 -3.33339 0.09519

C -3.40777 -2.43920 -0.47162

C -2.71346 -1.18211 -0.59237

N -1.43648 -1.33089 -0.11493

C -3.28172 0.06736 -1.01913

C -2.64939 1.30785 -0.66501

C -3.16088 2.64550 -0.82341

C -2.24701 3.49950 -0.25281

C -1.16892 2.69075 0.22029

N -1.40319 1.37527 -0.07837

C 0.08890 3.15365 0.79464

C 1.26429 2.61049 0.16601

C 2.46337 3.32144 -0.22979

C 3.24451 2.42755 -0.90131

C 2.54304 1.16384 -0.88497

N 1.32049 1.32767 -0.26005

C 0.14821 4.14952 1.82477

C -4.54860 0.03356 -1.72863

C 0.02738 -4.09243 1.90300

C 4.37982 -0.08929 -1.97041

C -4.77095 -0.91089 -2.76627

C -5.96142 -0.94381 -3.44970

N -6.97598 -0.08791 -3.14347

C -6.81559 0.80938 -2.13617

C -5.63671 0.89354 -1.43262

C 1.37683 4.55186 2.42457

C 1.40333 5.50915 3.40761

N 0.26016 6.08898 3.86348

C -0.93851 5.68901 3.36760

C -1.02341 4.73628 2.37979

C 5.51845 -0.65755 -1.38519

C 6.72391 -0.63404 -2.06503

N 6.81688 -0.07090 -3.28987

C 5.73260 0.48785 -3.87750

C 4.50673 0.49503 -3.23978

C 1.26713 -4.61744 2.37250

C 1.30885 -5.52378 3.40265

N 0.17630 -5.93228 4.03557

C -1.02236 -5.40438 3.66829

C -1.12292 -4.50084 2.64019

C 0.34395 7.14892 4.88853

C -8.25232 -0.18189 -3.87861

C 0.23747 -6.96098 5.09155

C 8.10859 -0.06515 -4.01961

Co -0.06422 -0.00438 -0.09148

H 3.95040 -2.87115 -1.58784

H 2.31445 -4.55097 -0.28824

H -2.69960 -4.38160 0.29452

H -4.43502 -2.62147 -0.75026

H -4.07425 2.92800 -1.32155

H -2.29196 4.57833 -0.21474

H 2.65333 4.37252 -0.07028

H 4.21279 2.60550 -1.34593

H -3.98656 -1.59535 -3.06537

H -6.14454 -1.63105 -4.26598

H -7.66682 1.43807 -1.90919

H -5.58890 1.59237 -0.60856

H 2.31704 4.09558 2.14811

H 2.32528 5.83818 3.86976

H -1.81771 6.15601 3.79187

H -2.01047 4.44163 2.05209

H 5.48216 -1.10198 -0.39839

H 7.63047 -1.05302 -1.64934

H 5.88253 0.91825 -4.85935

H 3.65730 0.94705 -3.73706

H 2.21115 -4.30041 1.95218

H 2.23954 -5.94281 3.76249

H -1.88530 -5.73105 4.23420

H -2.09995 -4.08991 2.42873

H 0.76609 8.04897 4.43642

H -0.65495 7.35910 5.26690

H 0.98185 6.80679 5.70472

H -8.80327 -1.06088 -3.53601

H -8.83860 0.71712 -3.69406

H -8.04335 -0.26655 -4.94597

H 1.22601 -6.94554 5.54938

H -0.51722 -6.74284 5.84732

H 0.04833 -7.94176 4.64849

H 8.34239 0.95893 -4.31188

H 8.88803 -0.45060 -3.36543

H 8.01375 -0.69904 -4.90246

H 0.03031 -0.02159 1.33089

[CoP(H)]^+3^  M = 2

C 2.6069918 -1.2327395 -0.8077344

C 3.2101148 -2.5281325 -0.9293304

C 2.3663608 -3.4203865 -0.3212794

C 1.2141098 -2.6806655 0.1199506

N 1.3567738 -1.3689235 -0.2161644

C 3.1888188 0.0056135 -1.1133294

C 0.0043628 -3.2290675 0.6730696

C -1.2224932 -2.6740905 0.1727776

C -2.4198712 -3.3973185 -0.1667114

C -3.2677412 -2.5104865 -0.7762604

C -2.6098732 -1.2359595 -0.7784914

N -1.3468472 -1.3753995 -0.2227504

C -3.1922332 -0.0026645 -1.1086854

C -2.6101092 1.2359725 -0.8047044

C -3.2131902 2.5314165 -0.9264004

C -2.3686692 3.4239175 -0.3197834

C -1.2158442 2.6843805 0.1202586

N -1.3591232 1.3724405 -0.2147924

C -0.0050482 3.2330945 0.6707566

C 1.2208378 2.6779075 0.1683556

C 2.4176058 3.4010135 -0.1736744

C 3.2646068 2.5137805 -0.7838174

C 2.6068328 1.2391925 -0.7839604

N 1.3445658 1.3789885 -0.2264934

C -0.0252732 4.3072495 1.6242846

C -4.5376032 -0.0262345 -1.7350994

C 0.0265968 -4.3028985 1.6269236

C 4.5352668 0.0278615 -1.7375904

C -4.7373372 -0.6825745 -2.9620484

C -5.9882462 -0.6976045 -3.5450724

N -7.0405842 -0.0876165 -2.9480864

C -6.8842582 0.5412955 -1.7608064

C -5.6507322 0.5848525 -1.1376024

C 1.1592118 4.8233345 2.2384116

C 1.1136158 5.8507885 3.1433886

N -0.0650542 6.4116345 3.5331936

C -1.2269292 5.8918585 3.0494596

C -1.2358402 4.8636575 2.1437896

C 5.6468188 -0.5800485 -1.1342724

C 6.8831328 -0.5345165 -1.7519044

N 7.0434108 0.0941365 -2.9384934

C 5.9916968 0.6977515 -3.5433164

C 4.7382158 0.6807395 -2.9660244

C 1.2383068 -4.8586005 2.1445606

C 1.2314398 -5.8868375 3.0501876

N 0.0706298 -6.4072885 3.5357636

C -1.1089542 -5.8469495 3.1480036

C -1.1565842 -4.8194745 2.2431416

C -0.0771952 7.5718505 4.4393266

C -8.3708552 -0.1480505 -3.5981254

C 0.0849768 -7.5677555 4.4415446

C 8.3652938 0.1173285 -3.6075094

Co -0.0010232 0.0018085 -0.1104584

H 4.1476598 -2.7509745 -1.4176074

H 2.4859258 -4.4925605 -0.2662064

H -2.5742762 -4.4588465 -0.0415124

H -4.2532202 -2.7166385 -1.1690084

H -4.1511732 2.7542445 -1.4138124

H -2.4881242 4.4961285 -0.2651124

H 2.5720608 4.4626585 -0.0495044

H 4.2494478 2.7196785 -1.1783064

H -3.9142802 -1.1714425 -3.4685624

H -6.1856592 -1.1827605 -4.4923984

H -7.7674432 0.9940355 -1.3295884

H -5.5654382 1.0848295 -0.1810534

H 2.1288238 4.3945055 2.0323116

H 2.0092348 6.2546485 3.5989116

H -2.1419162 6.3283315 3.4297746

H -2.2003952 4.4700535 1.8591506

H 5.5587888 -1.0763185 -0.1760464

H 7.7652548 -0.9854255 -1.3169784

H 6.1928548 1.1782505 -4.4923374

H 3.9164048 1.1679175 -3.4762004

H 2.2021858 -4.4644275 1.8584246

H 2.1472928 -6.3228255 3.4289736

H -2.0036082 -6.2512605 3.6050056

H -2.1267532 -4.3911075 2.0387076

H 0.0347678 8.4921705 3.8600456

H -1.0219832 7.5925885 4.9827016

H 0.7449968 7.4821645 5.1501646

H -8.7664372 -1.1603145 -3.4983724

H -9.0366672 0.5626495 -3.1121094

H -8.2608362 0.1117555 -4.6512424

H 1.0300298 -7.5871815 4.9845266

H -0.7370632 -7.4795825 5.1527376

H -0.0257792 -8.4880745 3.8620266

H 8.6091028 1.1466625 -3.8721534

H 9.1168938 -0.2760695 -2.9255844

H 8.3159818 -0.5016115 -4.5049054

H 0.0002618 0.0025665 1.3187646

[CoP(H)]^+3^  M = 4

C 2.5380463 -1.0836006 -0.9855003

C 3.1256623 -2.3756926 -1.2475053

C 2.3616043 -3.2991366 -0.5921913

C 1.2763923 -2.5800906 0.0377237

N 1.3600433 -1.2710466 -0.2549123

C 3.0706453 0.1724574 -1.2450153

C 0.1658133 -3.1489726 0.7948047

C -1.1276927 -2.6820046 0.4290887

C -2.3621247 -3.4074046 0.3933987

C -3.2953457 -2.5863796 -0.1990533

C -2.6366197 -1.3559906 -0.5134813

N -1.3290437 -1.4312496 -0.1388013

C -3.2777627 -0.1617386 -1.0485393

C -2.7273957 1.1126674 -0.7838103

C -3.2575897 2.4086374 -1.0928643

C -2.4212937 3.3426474 -0.5150993

C -1.3658437 2.6206414 0.1073047

N -1.5222147 1.2867964 -0.0985973

C -0.1751317 3.1976474 0.7495117

C 1.0693333 2.7253794 0.2653017

C 2.2834043 3.4703284 0.0446357

C 3.1394773 2.6434084 -0.6343283

C 2.4728143 1.3783814 -0.7776843

N 1.2121413 1.4586254 -0.2500493

C -0.2837247 4.2260724 1.7454687

C -4.5174897 -0.3348096 -1.7717563

C 0.3860253 -4.1400226 1.8077067

C 4.3671333 0.2835324 -1.9556173

C -4.6942867 -1.4263526 -2.6727643

C -5.8521127 -1.5815206 -3.3872823

N -6.8998517 -0.7137306 -3.2448613

C -6.7908757 0.3181824 -2.3613523

C -5.6425557 0.5291264 -1.6407083

C 0.8373753 4.6759104 2.5050457

C 0.7102753 5.6623034 3.4496017

N -0.4949707 6.2307054 3.7276407

C -1.6052527 5.7817114 3.0809147

C -1.5325037 4.8026534 2.1226157

C 5.5181583 -0.3888736 -1.5178093

C 6.7047003 -0.2589226 -2.2163523

N 6.7775153 0.5157094 -3.3220533

C 5.6864293 1.1880724 -3.7633763

C 4.4801313 1.0930984 -3.0998773

C 1.6719133 -4.6898616 2.1032357

C 1.8396083 -5.6380376 3.0779887

N 0.7966873 -6.0806396 3.8355747

C -0.4329397 -5.5261236 3.6479627

C -0.6546557 -4.5761596 2.6855977

C -0.5948147 7.3413814 4.6917927

C -8.1453857 -0.9473906 -3.9936153

C 1.0004433 -7.1573906 4.8193757

C 8.0444453 0.6263154 -4.0827893

Co -0.0695507 0.0153234 -0.0909563

H 4.0035073 -2.5642786 -1.8481293

H 2.4908673 -4.3716726 -0.5880783

H -2.5013767 -4.4364186 0.6909807

H -4.3329717 -2.8144016 -0.3949263

H -4.1371627 2.6189604 -1.6809593

H -2.5096447 4.4188604 -0.5648893

H 2.4371003 4.5131914 0.2808557

H 4.1328913 2.8802124 -0.9875363

H -3.8912197 -2.1336156 -2.8401853

H -5.9899867 -2.3809816 -4.1043983

H -7.6644397 0.9478184 -2.2486313

H -5.6402237 1.3351814 -0.9197273

H 1.8113613 4.2224544 2.3874347

H 1.5540363 6.0189594 4.0262697

H -2.5438997 6.2396174 3.3662977

H -2.4576967 4.4768024 1.6689807

H 5.5030713 -0.9976866 -0.6227563

H 7.6145813 -0.7579456 -1.9099543

H 5.8175243 1.7881104 -4.6547723

H 3.6264793 1.6401014 -3.4807503

H 2.5588793 -4.3632936 1.5801257

H 2.8067353 -6.0738616 3.2949447

H -1.2186497 -5.8658406 4.3106537

H -1.6401217 -4.1353726 2.6421007

H -0.5266087 8.2927124 4.1581337

H -1.5494557 7.2781444 5.2144547

H 0.2189223 7.2638404 5.4126077

H -8.6980687 -1.7755496 -3.5421503

H -8.7541337 -0.0441586 -3.9696303

H -7.9022807 -1.1893326 -5.0298473

H 1.9637003 -7.0176846 5.3119597

H 0.2054153 -7.1174586 5.5637597

H 0.9814353 -8.1255776 4.3123197

H 8.2610953 1.6807874 -4.2553573

H 8.8481883 0.1749484 -3.5042503

H 7.9293663 0.1035334 -5.0339093

H 0.0055953 0.0063254 1.3352727

[CoP(H)]^+3^  M = 6

C 2.4965806 -1.3976869 -0.8114638

C 2.9503966 -2.7542859 -0.9789848

C 2.0577816 -3.5658249 -0.3148658

C 1.0422076 -2.7143689 0.2136122

N 1.2960626 -1.4155079 -0.1364908

C 3.1404286 -0.1772689 -1.2107278

C -0.1731204 -3.1037029 0.9126692

C -1.3860124 -2.5486019 0.3472502

C -2.6272014 -3.2050619 0.0791532

C -3.4234314 -2.2929539 -0.5760548

C -2.6590344 -1.0801039 -0.7077528

N -1.4215584 -1.2725729 -0.1543098

C -3.1457124 0.1810171 -1.2048168

C -2.5026894 1.4040751 -0.8123058

C -2.9586024 2.7594411 -0.9830378

C -2.0640174 3.5744961 -0.3257838

C -1.0456824 2.7261011 0.2022062

N -1.2998544 1.4255971 -0.1414258

C 0.1721606 3.1190041 0.8948122

C 1.3833066 2.5615281 0.3275482

C 2.6230406 3.2172581 0.0513782

C 3.4180056 2.3016391 -0.6004158

C 2.6543286 1.0871941 -0.7218868

N 1.4181546 1.2824801 -0.1661078

C 0.1705286 4.0548121 1.9677622

C -4.3407874 0.1681331 -2.0245398

C -0.1675604 -4.0351059 1.9894522

C 4.3379416 -0.1724409 -2.0270068

C -4.5093604 -0.8168749 -3.0377738

C -5.6252414 -0.8326779 -3.8350668

N -6.6271434 0.0789411 -3.6699168

C -6.5226614 1.0157581 -2.6898608

C -5.4152014 1.0862121 -1.8791708

C 1.3596156 4.4110981 2.6782412

C 1.3289996 5.3194961 3.7029802

N 0.1646286 5.9087261 4.1005602

C -1.0031204 5.5545631 3.4926532

C -1.0312134 4.6489011 2.4647032

C 5.4106956 -1.0913419 -1.8677798

C 6.5228346 -1.0281689 -2.6710008

N 6.6380446 -0.0941909 -3.6541608

C 5.6369796 0.8135701 -3.8356478

C 4.5146086 0.8041651 -3.0458528

C 1.0358046 -4.6280979 2.4837132

C 1.0113286 -5.5300599 3.5150242

N -0.1541444 -5.8813249 4.1288922

C -1.3196914 -5.2924139 3.7341922

C -1.3538914 -4.3876659 2.7063762

C 0.1814236 6.9423341 5.1500362

C -7.8239654 0.0039221 -4.5277868

C -0.1678424 -6.9113019 5.1819822

C 7.8081936 -0.0949989 -4.5508168

Co -0.0015924 0.0053401 -0.0537648

H 3.8128016 -3.0797159 -1.5380098

H 2.0747316 -4.6443629 -0.2458288

H -2.8631884 -4.2331209 0.3133792

H -4.4421314 -2.4343389 -0.9063648

H -3.8238264 3.0819461 -1.5393088

H -2.0818434 4.6532891 -0.2608978

H 2.8589386 4.2469391 0.2784692

H 4.4355946 2.4419171 -0.9344878

H -3.7335224 -1.5488899 -3.2260178

H -5.7585114 -1.5501339 -4.6350128

H -7.3636794 1.6873091 -2.5736198

H -5.4194204 1.8187941 -1.0840578

H 2.3088176 3.9494441 2.4436582

H 2.2197026 5.6054301 4.2480862

H -1.9020694 6.0227751 3.8724142

H -1.9958534 4.3863061 2.0523292

H 5.4095126 -1.8155069 -1.0648858

H 7.3619816 -1.7008889 -2.5472408

H 5.7740196 1.5247431 -4.6401748

H 3.7427646 1.5380811 -3.2428218

H 1.9989326 -4.3677029 2.0664392

H 1.9115376 -5.9975359 3.8927032

H -2.2082784 -5.5756099 4.2841652

H -2.3036414 -3.9258959 2.4742522

H 0.4278896 7.9098191 4.7049022

H -0.8006274 6.9926621 5.6198832

H 0.9283916 6.6797771 5.8998962

H -8.4049424 -0.8834389 -4.2653138

H -8.4282344 0.8971381 -4.3764818

H -7.5135144 -0.0518899 -5.5726928

H 0.8177816 -6.9654729 5.6438022

H -0.9071174 -6.6421939 5.9371682

H -0.4232454 -7.8789059 4.7421862

H 8.0292256 0.9292401 -4.8514298

H 8.6652656 -0.5085579 -4.0194018

H 7.5915996 -0.7023979 -5.4332918

H 0.0004096 0.0097521 1.3749812

[CoP(H)]^+2^  M = 1

C 2.6221030 -1.2435929 -0.8030057

C 3.2087140 -2.5400059 -0.9251447

C 2.3459950 -3.4286889 -0.3232747

C 1.2105760 -2.6807149 0.1261253

N 1.3735390 -1.3682799 -0.1896757

C 3.2003380 -0.0036989 -1.1125477

C -0.0053680 -3.2123369 0.7166463

C -1.2360810 -2.6663869 0.1780113

C -2.4249230 -3.3900799 -0.1679297

C -3.2820260 -2.5011699 -0.7745607

C -2.6282520 -1.2302959 -0.7816097

N -1.3673100 -1.3672089 -0.2034337

C -3.2029520 0.0070331 -1.1085517

C -2.6245100 1.2473901 -0.8010607

C -3.2107970 2.5438481 -0.9246517

C -2.3473490 3.4331231 -0.3246947

C -1.2116990 2.6854881 0.1246213

N -1.3754010 1.3726311 -0.1889617

C 0.0051770 3.2176501 0.7126963

C 1.2349910 2.6712641 0.1724713

C 2.4234830 3.3945451 -0.1755737

C 3.2798510 2.5049421 -0.7822407

C 2.6259580 1.2341201 -0.7872317

N 1.3655670 1.3717531 -0.2080017

C -0.0136060 4.2296031 1.6913053

C -4.5430760 -0.0140889 -1.7423227

C 0.0149470 -4.2237999 1.6957513

C 4.5415480 0.0157961 -1.7442117

C -4.7435830 -0.6864429 -2.9614357

C -5.9912420 -0.7018429 -3.5502337

N -7.0434320 -0.0774939 -2.9667717

C -6.8875650 0.5675401 -1.7876177

C -5.6567630 0.6126281 -1.1602227

C 1.1764780 4.7476091 2.3269143

C 1.1284500 5.7456951 3.2537273

N -0.0540730 6.3007191 3.6702193

C -1.2193660 5.7777811 3.1719633

C -1.2301320 4.7794941 2.2442313

C 5.6539350 -0.6077319 -1.1563677

C 6.8871540 -0.5615289 -1.7788957

N 7.0466110 0.0825391 -2.9579397

C 5.9949590 0.7010361 -3.5487267

C 4.7449750 0.6844641 -2.9649117

C 1.2323000 -4.7733739 2.2472013

C 1.2229600 -5.7717759 3.1748163

N 0.0584320 -6.2950489 3.6744993

C -1.1247190 -5.7399719 3.2598673

C -1.1741510 -4.7417499 2.3332683

C -0.0684910 7.4570271 4.5699353

C -8.3705440 -0.1405859 -3.6213937

C 0.0742850 -7.4516639 4.5737913

C 8.3644380 0.1038811 -3.6335817

Co -0.0008540 0.0021861 -0.2138457

H 4.1513770 -2.7727599 -1.4000477

H 2.4593560 -4.5011079 -0.2494517

H -2.5800030 -4.4496579 -0.0230927

H -4.2743020 -2.7072139 -1.1516687

H -4.1536590 2.7764091 -1.3992267

H -2.4604010 4.5056701 -0.2521047

H 2.5787820 4.4542461 -0.0318567

H 4.2717240 2.7104441 -1.1607137

H -3.9210210 -1.1886709 -3.4552847

H -6.1878700 -1.1987539 -4.4916957

H -7.7701490 1.0311581 -1.3667307

H -5.5718080 1.1253081 -0.2105407

H 2.1475110 4.3291221 2.1007443

H 2.0243340 6.1392061 3.7195623

H -2.1344250 6.1975321 3.5728613

H -2.1942220 4.3896551 1.9477773

H 5.5666150 -1.1161169 -0.2045947

H 7.7689260 -1.0231919 -1.3544867

H 6.1945440 1.1932381 -4.4921067

H 3.9236650 1.1854891 -3.4620827

H 2.1959220 -4.3832419 1.9496393

H 2.1386370 -6.1913779 3.5744563

H -2.0199010 -6.1335869 3.7269583

H -2.1455350 -4.3232519 2.1086393

H -0.0115240 8.3899881 3.9996343

H -0.9894530 7.4489911 5.1556823

H 0.7829420 7.3985541 5.2506303

H -8.7659570 -1.1530419 -3.5223387

H -9.0396190 0.5694841 -3.1387847

H -8.2576300 0.1179641 -4.6746247

H 0.9958380 -7.4434309 5.1586153

H -0.7764940 -7.3938259 5.2553543

H 0.0171690 -8.3844449 4.0032073

H 8.6053470 1.1315601 -3.9072917

H 9.1206750 -0.2824479 -2.9525337

H 8.3133870 -0.5217269 -4.5264127

H -0.0022760 0.0015991 -1.6435257

[CoP(H)]^+2^  M = 3

C 2.5209080 -1.2618340 -0.8912417

C 3.0833870 -2.5683610 -1.0599007

C 2.2571000 -3.4463810 -0.4010007

C 1.1536590 -2.6856630 0.1140893

N 1.3080020 -1.3751580 -0.2240337

C 3.1120450 -0.0229200 -1.1950787

C -0.0221730 -3.1925340 0.7773063

C -1.2834050 -2.6287830 0.3700093

C -2.5264240 -3.3128700 0.2180573

C -3.4028720 -2.4348390 -0.3855887

C -2.7039220 -1.2068200 -0.5862577

N -1.4140380 -1.3491820 -0.1243907

C -3.2879690 0.0308520 -1.0198787

C -2.6703210 1.2763610 -0.6938427

C -3.1892630 2.5949060 -0.8626347

C -2.3063450 3.4648190 -0.2523077

C -1.2134720 2.6879830 0.2234873

N -1.4274890 1.3654460 -0.0766907

C 0.0223510 3.1992730 0.7762183

C 1.2352560 2.6362720 0.2540603

C 2.4518540 3.3396730 -0.0602927

C 3.2629900 2.4636540 -0.7339277

C 2.5671200 1.2103150 -0.7952937

N 1.3231060 1.3540120 -0.2026517

C 0.0284790 4.2504960 1.7451093

C -4.5562540 -0.0274070 -1.7096217

C 0.0714970 -4.2180480 1.7719483

C 4.4269400 0.0016910 -1.8738527

C -4.8132050 -1.0315330 -2.6957227

C -5.9934590 -1.0796350 -3.3842817

N -7.0032870 -0.1818900 -3.1409597

C -6.8205950 0.7662230 -2.1723167

C -5.6454580 0.8683000 -1.4768797

C 1.2215390 4.7197920 2.3903483

C 1.1966860 5.7329950 3.3086033

N 0.0300540 6.3315400 3.6911203

C -1.1424410 5.8557600 3.1792263

C -1.1721460 4.8450700 2.2580533

C 5.5429990 -0.6969540 -1.3823997

C 6.7514370 -0.6390090 -2.0497587

N 6.8871820 0.0896740 -3.1821077

C 5.8330550 0.7832570 -3.6791417

C 4.6071660 0.7571300 -3.0483917

C 1.3107340 -4.8143890 2.1809123

C 1.3621150 -5.8003800 3.1275513

N 0.2414870 -6.2438320 3.7700823

C -0.9489890 -5.6376470 3.4910493

C -1.0544800 -4.6523380 2.5479813

C 0.0445010 7.4813800 4.6060763

C -8.2734990 -0.2980280 -3.8676717

C 0.3155740 -7.3673950 4.7144253

C 8.1777500 0.1266700 -3.9071727

Co -0.0574810 0.0011060 -0.1781107

H 3.9852190 -2.8126120 -1.6026187

H 2.3613460 -4.5206360 -0.3499637

H -2.7157110 -4.3492200 0.4576293

H -4.4378020 -2.6228330 -0.6330147

H -4.0912370 2.8710840 -1.3859037

H -2.3779130 4.5430010 -0.2177407

H 2.6439360 4.3860900 0.1257633

H 4.2518080 2.6613330 -1.1232727

H -4.0436140 -1.7503850 -2.9495147

H -6.1836370 -1.8068960 -4.1640417

H -7.6643100 1.4160910 -1.9761457

H -5.5903030 1.6091120 -0.6909477

H 2.1783450 4.2573600 2.1958783

H 2.0976090 6.0968970 3.7873633

H -2.0476880 6.3178630 3.5532143

H -2.1431200 4.4913020 1.9431613

H 5.4807560 -1.2694770 -0.4657827

H 7.6335440 -1.1570960 -1.6974037

H 6.0107860 1.3419920 -4.5891837

H 3.7842890 1.3172850 -3.4749447

H 2.2524330 -4.4819840 1.7698493

H 2.2949180 -6.2650960 3.4223963

H -1.8015190 -5.9721080 4.0691953

H -2.0197610 -4.1817880 2.4332193

H 0.2131580 8.4043210 4.0438663

H -0.9130150 7.5404230 5.1245013

H 0.8410230 7.3500100 5.3400953

H -8.8418390 -1.1565510 -3.4977267

H -8.8555670 0.6120400 -3.7226407

H -8.0723920 -0.4269050 -4.9335947

H 1.2677170 -7.3304710 5.2458053

H -0.4994630 -7.2866390 5.4344963

H 0.2339190 -8.3151830 4.1746713

H 8.4493470 1.1662710 -4.0942557

H 8.9434160 -0.3491080 -3.2968067

H 8.0681770 -0.4098420 -4.8514877

H -0.1108390 0.0215330 -1.6064287

[CoP(H)]^+2^  M = 5

C 2.6211122 -1.1433950 -0.8405572

C 3.0954962 -2.4539990 -1.1749812

C 2.2703092 -3.3621400 -0.5379792

C 1.2751552 -2.6089550 0.1438338

N 1.4598262 -1.2811300 -0.0758692

C 3.1811942 0.1227960 -1.1266262

C 0.1275872 -3.1333800 0.9014898

C -1.1463498 -2.6536970 0.4885588

C -2.4030418 -3.3373090 0.4514068

C -3.2923558 -2.5102610 -0.2012802

C -2.5842098 -1.3163180 -0.5453022

N -1.2941148 -1.4176850 -0.1259442

C -3.1725138 -0.1196330 -1.1365562

C -2.6123678 1.1467600 -0.8511212

C -3.0844988 2.4570780 -1.1899172

C -2.2613608 3.3659000 -0.5512572

C -1.2695608 2.6133990 0.1360618

N -1.4540038 1.2852350 -0.0821922

C -0.1250788 3.1386030 0.8978698

C 1.1503962 2.6581450 0.4907308

C 2.4073972 3.3414080 0.4574748

C 3.2987082 2.5137620 -0.1917462

C 2.5913912 1.3198450 -0.5374872

N 1.3000702 1.4216200 -0.1222982

C -0.3103728 4.1063460 1.9281298

C -4.3703338 -0.2819070 -1.9246152

C 0.3088682 -4.0994400 1.9340828

C 4.3804092 0.2845420 -1.9126742

C -4.5358498 -1.4073090 -2.7884862

C -5.6510138 -1.5531260 -3.5684332

N -6.6728088 -0.6420630 -3.5323722

C -6.5793478 0.4226860 -2.6839142

C -5.4718568 0.6248150 -1.9023252

C 0.7456782 4.5178770 2.8065738

C 0.5462512 5.4609960 3.7768898

N -0.6750298 6.0398620 3.9768718

C -1.7314858 5.6240640 3.2156998

C -1.5859428 4.6851720 2.2317148

C 5.4833582 -0.6206670 -1.8859782

C 6.5933752 -0.4172420 -2.6635962

N 6.6924552 0.6525950 -3.5050802

C 5.6652992 1.5568760 -3.5521782

C 4.5471872 1.4093370 -2.7766252

C 1.5834732 -4.6769200 2.2441968

C 1.7253492 -5.6139800 3.2304538

N 0.6658572 -6.0291600 3.9877238

C -0.5548968 -5.4514480 3.7814578

C -0.7506998 -4.5101040 2.8086508

C -0.8459768 7.1198500 4.9594608

C -7.8839268 -0.8659180 -4.3358812

C 0.8335492 -7.1072620 4.9729378

C 7.8542162 0.8128900 -4.3921552

Co 0.0029662 0.0020060 -0.1231432

H 3.9342562 -2.6937290 -1.8099722

H 2.3357512 -4.4410810 -0.5700442

H -2.5860918 -4.3462730 0.7916738

H -4.3332678 -2.7089340 -0.4135132

H -3.9203188 2.6962360 -1.8289712

H -2.3260238 4.4448160 -0.5856052

H 2.5895352 4.3505540 0.7976798

H 4.3403492 2.7120570 -0.4007622

H -3.7516468 -2.1495210 -2.8735292

H -5.7742098 -2.3783980 -4.2585712

H -7.4351628 1.0852440 -2.6517912

H -5.4866098 1.4593880 -1.2150452

H 1.7219142 4.0571610 2.7521978

H 1.3393282 5.7790410 4.4420108

H -2.6879908 6.0792680 3.4406928

H -2.4733958 4.3869150 1.6908598

H 5.4985042 -1.4521980 -1.1949672

H 7.4488122 -1.0803260 -2.6310282

H 5.7877492 2.3806690 -4.2440202

H 3.7640002 2.1525410 -2.8621582

H 2.4730732 -4.3789540 1.7067338

H 2.6811572 -6.0681110 3.4605158

H -1.3506698 -5.7688110 4.4436838

H -1.7269648 -4.0500780 2.7492948

H -0.7471708 8.0910270 4.4662478

H -1.8337298 7.0400260 5.4159048

H -0.0843928 7.0250290 5.7340208

H -8.5385388 -1.5827070 -3.8319892

H -8.4095748 0.0799680 -4.4670932

H -7.5992278 -1.2560350 -5.3145132

H 1.8182162 -7.0241350 5.4354438

H 0.0668682 -7.0134120 5.7425508

H 0.7404452 -8.0793830 4.4804868

H 8.1232022 1.8689520 -4.4480542

H 8.6955722 0.2500550 -3.9875632

H 7.6126922 0.4416260 -5.3921692

H 0.0056212 0.0014990 -1.5523362

[CoP(H)]^+1^  M = 2

C 2.6932992 -1.2153081 -0.7454929

C 3.2181912 -2.5198241 -0.9368059

C 2.3381642 -3.4117531 -0.3336379

C 1.2583502 -2.6530341 0.1738441

N 1.4584752 -1.3265481 -0.0989409

C 3.2869552 0.0428859 -1.0687219

C 0.0334402 -3.1725921 0.7823631

C -1.1943558 -2.6544441 0.2215631

C -2.3876838 -3.3843661 -0.0961119

C -3.2229268 -2.5247411 -0.7720969

C -2.5563038 -1.2604001 -0.8405339

N -1.3090538 -1.3763871 -0.2348399

C -3.1281648 -0.0355271 -1.2306199

C -2.5673738 1.2178479 -0.9236289

C -3.1528798 2.5108969 -1.0841059

C -2.3328258 3.4063269 -0.4296759

C -1.2196788 2.6675439 0.0809791

N -1.3535648 1.3546889 -0.2507579

C -0.0498268 3.1886659 0.7646741

C 1.2282572 2.6695999 0.2941971

C 2.4331302 3.3919339 0.0916591

C 3.3351432 2.5238259 -0.5057619

C 2.6798622 1.2733139 -0.6636169

N 1.3884242 1.3870629 -0.1638299

C -0.1563318 4.1526999 1.7796411

C -4.4419268 -0.0875751 -1.9048179

C 0.0579372 -4.1554431 1.7829371

C 4.5659902 0.1088669 -1.7424119

C -4.6157868 -0.8553931 -3.0738559

C -5.8407508 -0.9026941 -3.7039749

N -6.9056128 -0.2210171 -3.2108639

C -6.7789008 0.5146059 -2.0804059

C -5.5707348 0.5971899 -1.4176669

C 0.9767302 4.6430489 2.5378341

C 0.8507732 5.5969299 3.5007211

N -0.3637548 6.1412819 3.8409921

C -1.4841198 5.6440439 3.2196991

C -1.4170858 4.6862799 2.2547071

C 5.6668302 -0.7624951 -1.4629409

C 6.8575432 -0.6541021 -2.1265519

N 7.0548152 0.2864459 -3.1067569

C 6.0255622 1.1492909 -3.4073159

C 4.8296222 1.0927459 -2.7480339

C 1.2830622 -4.7057991 2.3245781

C 1.2837572 -5.6760991 3.2791201

N 0.1233592 -6.1769161 3.8185361

C -1.0654568 -5.6260641 3.4052621

C -1.1253238 -4.6540411 2.4539581

C -0.4552598 7.2637009 4.7748951

C -8.2122998 -0.3277181 -3.8962519

C 0.1484562 -7.3130911 4.7401961

C 8.2984152 0.3222959 -3.8804779

Co 0.0518812 0.0083309 -0.2125969

H 4.1247972 -2.7792551 -1.4625449

H 2.4241762 -4.4896371 -0.2941499

H -2.5572418 -4.4322041 0.1074581

H -4.2107698 -2.7435101 -1.1538939

H -4.0659918 2.7396109 -1.6155259

H -2.4604448 4.4775899 -0.3573839

H 2.5900142 4.4375789 0.3176031

H 4.3613282 2.7397659 -0.7688589

H -3.7843568 -1.4046051 -3.4978589

H -6.0113078 -1.4691831 -4.6106799

H -7.6703088 1.0159559 -1.7264439

H -5.5146028 1.1783989 -0.5063029

H 1.9621562 4.2300519 2.3716831

H 1.7036272 5.9636119 4.0602641

H -2.4295688 6.0505039 3.5601001

H -2.3531138 4.3127389 1.8620591

H 5.5994972 -1.4872501 -0.6625009

H 7.7081282 -1.2836771 -1.8952919

H 6.2198692 1.8591079 -4.2019019

H 4.0532992 1.7920869 -3.0338469

H 2.2411562 -4.3377911 1.9824861

H 2.2031292 -6.0949561 3.6716071

H -1.9542148 -6.0004951 3.9001281

H -2.0975348 -4.2331691 2.2357011

H -0.3992308 8.2200539 4.2432741

H -1.4018578 7.2103889 5.3169281

H 0.3645742 7.2059299 5.4937411

H -8.6535098 -1.3009891 -3.6722899

H -8.8664628 0.4677949 -3.5431729

H -8.0583228 -0.2247321 -4.9707099

H 1.0749712 -7.2923171 5.3177081

H -0.6959028 -7.2420961 5.4290151

H 0.0871652 -8.2607261 4.1938151

H 8.5593032 1.3589879 -4.1029519

H 9.1015852 -0.1266251 -3.2942539

H 8.1828012 -0.2315641 -4.8181099

H 0.1047182 -0.0143551 -1.6431509

[CoP(H)]^+1^  M = 4

C 2.6017290 -1.2546154 -0.7927416

C 3.1064080 -2.5727444 -0.9966566

C 2.2567690 -3.4437964 -0.3362006

C 1.1980160 -2.6660554 0.2053704

N 1.3954090 -1.3459504 -0.1070546

C 3.2046460 -0.0019704 -1.1259826

C 0.0039020 -3.1575094 0.8648614

C -1.2478690 -2.6124294 0.4042934

C -2.4884350 -3.2971044 0.2371574

C -3.3405770 -2.4377214 -0.4275666

C -2.6299380 -1.2197004 -0.6481346

N -1.3566550 -1.3518954 -0.1378646

C -3.1966310 0.0069566 -1.1353596

C -2.5945860 1.2606056 -0.8044326

C -3.0982620 2.5781046 -1.0145656

C -2.2502770 3.4513356 -0.3548566

C -1.1935550 2.6752606 0.1929634

N -1.3903790 1.3540616 -0.1152426

C -0.0016970 3.1686496 0.8552334

C 1.2515830 2.6218476 0.4009784

C 2.4930030 3.3056876 0.2363544

C 3.3470010 2.4445006 -0.4235086

C 2.6367580 1.2260946 -0.6434316

N 1.3619540 1.3598306 -0.1374796

C -0.0799740 4.1751306 1.8574984

C -4.4351930 -0.0683484 -1.8723016

C 0.0787220 -4.1604694 1.8709534

C 4.4449820 0.0705046 -1.8600096

C -4.6645030 -1.1079184 -2.8303616

C -5.8166530 -1.1745374 -3.5619936

N -6.8297440 -0.2606934 -3.3935026

C -6.6756050 0.7225426 -2.4527686

C -5.5276490 0.8433566 -1.7179626

C 1.0555160 4.6135056 2.6274764

C 0.9567990 5.5926536 3.5734424

N -0.2356140 6.1987706 3.8694264

C -1.3641560 5.7506106 3.2335414

C -1.3196310 4.7714216 2.2837954

C 5.5367680 -0.8418224 -1.6994746

C 6.6865130 -0.7245384 -2.4310456

N 6.8495950 0.2641756 -3.3658236

C 5.8341670 1.1718176 -3.5466386

C 4.6789970 1.1077996 -2.8188006

C 1.3170020 -4.7546034 2.3041104

C 1.3583670 -5.7300664 3.2578344

N 0.2276620 -6.1762134 3.8912294

C -0.9639160 -5.5718984 3.5883474

C -1.0595090 -4.5964884 2.6381804

C -0.2992100 7.3268826 4.8038164

C -8.0723740 -0.3990244 -4.1604256

C 0.2883340 -7.3005744 4.8303524

C 8.0431980 0.3070476 -4.2166386

Co 0.0026260 0.0039876 -0.1619216

H 3.9778070 -2.8495134 -1.5695996

H 2.3317670 -4.5220024 -0.2986246

H -2.6908580 -4.3226954 0.5106694

H -4.3679830 -2.6304194 -0.7024976

H -3.9680580 2.8528956 -1.5908866

H -2.3251310 4.5296856 -0.3213446

H 2.6948800 4.3318936 0.5079424

H 4.3752820 2.6363406 -0.6957256

H -3.8918660 -1.8414534 -3.0264996

H -5.9821910 -1.9303354 -4.3200676

H -7.5214210 1.3844546 -2.3129016

H -5.4972940 1.6127626 -0.9587056

H 2.0202910 4.1434206 2.5014704

H 1.8128460 5.9254276 4.1479964

H -2.2950240 6.2113976 3.5415504

H -2.2612260 4.4393086 1.8699434

H 5.5044480 -1.6060774 -0.9350176

H 7.5307880 -1.3885424 -2.2905376

H 6.0006940 1.9247856 -4.3069796

H 3.9098450 1.8444946 -3.0166396

H 2.2600120 -4.4236924 1.8925164

H 2.2882570 -6.1892364 3.5711404

H -1.8219860 -5.9028534 4.1609414

H -2.0240470 -4.1274454 2.5065784

H -0.2047460 8.2747496 4.2645294

H -1.2532250 7.3056996 5.3337984

H 0.5115040 7.2417646 5.5291724

H -8.6585060 -1.2426134 -3.7829866

H -8.6563730 0.5167646 -4.0683026

H -7.8350830 -0.5650124 -5.2139726

H 1.2403830 -7.2768284 5.3637514

H -0.5250850 -7.2129724 5.5523644

H 0.1962920 -8.2506004 4.2944724

H 8.2919390 1.3458416 -4.4407306

H 8.8799170 -0.1520914 -3.6880646

H 7.8644640 -0.2338294 -5.1515156

H 0.0048540 0.0020726 -1.5937046

[CoP(H)]^0^  M = 1

C -2.2292576 1.2495686 -1.0749134

C -2.7495746 2.5624926 -1.2956704

C -2.0059316 3.4285006 -0.5164484

C -0.9889376 2.6577346 0.1265126

N -1.1149296 1.3525826 -0.2457454

C -2.7519926 0.0029196 -1.4992004

C 0.0935834 3.1317716 0.9738016

C 1.4227774 2.5881646 0.6933526

C 2.6705814 3.2372246 0.7369876

C 3.6152554 2.3598816 0.1716936

C 2.9316794 1.1908516 -0.1988454

N 1.6003954 1.3324566 0.1342736

C 3.4551014 -0.0534904 -0.7771414

C 2.9011404 -1.2900024 -0.2132074

C 3.5461624 -2.4883264 0.1321496

C 2.5806884 -3.3295824 0.7186396

C 1.3609384 -2.6300814 0.7136656

N 1.5733944 -1.3824604 0.1495846

C 0.0229454 -3.1063774 1.0745396

C -1.0732606 -2.6288094 0.2483776

C -2.2066126 -3.3547844 -0.2387644

C -2.9277116 -2.4993714 -1.0485734

C -2.2487046 -1.2410584 -1.0382874

N -1.1087546 -1.3631594 -0.2585504

C -0.2052986 -3.9769764 2.1534716

C 4.4196954 -0.0576364 -1.7781474

C -0.1504566 4.0736036 1.9874206

C -3.9285116 -0.0304214 -2.3732094

C 4.9749084 1.1501576 -2.3830164

C 5.9602324 1.1159296 -3.3142384

N 6.4916924 -0.0684384 -3.7944114

C 5.9313964 -1.2461584 -3.3314294

C 4.9454764 -1.2698234 -2.4004364

C -1.5167956 -4.4418494 2.5669816

C -1.6912276 -5.3193614 3.5919926

N -0.6398956 -5.7942584 4.3428026

C 0.6130714 -5.2955124 4.0698056

C 0.8437684 -4.4241974 3.0509516

C -5.0862226 0.7505896 -2.1502504

C -6.1608946 0.6834236 -3.0087204

N -6.1446736 -0.1310544 -4.0956544

C -5.0535316 -0.9095034 -4.3385794

C -3.9618516 -0.8827144 -3.5042214

C -1.4572476 4.6296706 2.2852246

C -1.6470846 5.5699796 3.2501186

N -0.6208436 6.0266746 4.0461936

C 0.6179064 5.4519156 3.8811176

C 0.8648004 4.5170266 2.9240936

C -0.8357106 -6.8514924 5.3322476

C 7.7038404 -0.0771494 -4.6020924

C -0.8254466 7.1381116 4.9728166

C -7.2835446 -0.1736484 -5.0327764

Co 0.2553184 -0.0143814 -0.1031704

H -3.5659776 2.8312816 -1.9508764

H -2.1199006 4.5019086 -0.4478914

H 2.8525504 4.2520946 1.0650216

H 4.6790784 2.5331136 0.0687266

H 4.6002454 -2.7017984 0.0078776

H 2.7301324 -4.3497714 1.0473356

H -2.4209316 -4.3962434 -0.0428754

H -3.8547226 -2.7195134 -1.5600814

H 4.5785054 2.1182186 -2.1051544

H 6.3685974 2.0197276 -3.7544854

H 6.3175344 -2.1531984 -3.7849034

H 4.5247124 -2.2317344 -2.1375934

H -2.4081126 -4.0706594 2.0806396

H -2.6737756 -5.6713434 3.8856346

H 1.4048354 -5.6287404 4.7313346

H 1.8475724 -4.0387074 2.9392726

H -5.1634106 1.3853526 -1.2774454

H -7.0645896 1.2577966 -2.8498154

H -5.0975256 -1.5307514 -5.2243024

H -3.1105376 -1.5097944 -3.7381174

H -2.3333196 4.2855436 1.7525686

H -2.6239436 5.9923786 3.4567246

H 1.3833194 5.7797786 4.5753846

H 1.8510344 4.0750776 2.8977886

H -0.7491956 -7.8429414 4.8727386

H -0.0844916 -6.7574694 6.1195076

H -1.8268306 -6.7524894 5.7808566

H 8.6085224 -0.0914874 -3.9797774

H 7.7081384 -0.9600684 -5.2468634

H 7.7284644 0.8136416 -5.2354964

H -1.8337776 7.0856296 5.3903146

H -0.1044636 7.0673856 5.7899966

H -0.6980206 8.1020476 4.4667446

H -7.5910416 -1.2106724 -5.1783254

H -8.1120436 0.3963616 -4.6150994

H -6.9818576 0.2623056 -5.9878094

H 0.4533804 -0.0114094 -1.5246664

[CoP(H)]^0^  M = 3

C -2.3185412 1.2638758 -1.0284981

C -2.7635862 2.5881068 -1.2877141

C -1.9633122 3.4483048 -0.5387781

C -1.0063352 2.6510738 0.1318439

N -1.2047002 1.3387908 -0.1897941

C -2.8781312 0.0144068 -1.4525341

C 0.1071308 3.0942498 0.9807269

C 1.4118018 2.5548858 0.6200689

C 2.6691068 3.2119218 0.5905769

C 3.5674238 2.3522308 -0.0367631

C 2.8602738 1.1649388 -0.3611851

N 1.5522938 1.3059598 0.0662139

C 3.3738288 -0.0742372 -0.9048721

C 2.8089308 -1.3078252 -0.3969991

C 3.4529488 -2.5395782 -0.1202821

C 2.5230038 -3.3570062 0.5243529

C 1.3151328 -2.6255182 0.6186069

N 1.5088318 -1.3819042 0.0693059

C 0.0047938 -3.0702942 1.0943419

C -1.1376572 -2.6101822 0.3023189

C -2.2952932 -3.3290852 -0.0978831

C -3.0648092 -2.4706602 -0.8745671

C -2.3732142 -1.2299062 -0.9469771

N -1.1933742 -1.3514322 -0.2283211

C -0.1477862 -3.8878172 2.2162479

C 4.3999738 -0.0773582 -1.8971021

C -0.0834862 4.0103008 2.0162759

C -4.0260482 -0.0289402 -2.3292071

C 4.9515108 1.1219918 -2.4763411

C 5.9538718 1.0912608 -3.4017051

N 6.4753378 -0.0875372 -3.8711231

C 5.9047158 -1.2586582 -3.4415181

C 4.9021588 -1.2786392 -2.5160561

C -1.4351462 -4.3255712 2.7334079

C -1.5478062 -5.1603792 3.7994199

N -0.4518442 -5.6250142 4.4949729

C 0.7876108 -5.1569912 4.1139839

C 0.9583788 -4.3230792 3.0550589

C -5.1279502 0.8843428 -2.2603901

C -6.1911542 0.7971868 -3.1148911

N -6.2455642 -0.1511952 -4.1076921

C -5.2202912 -1.0666322 -4.1974121

C -4.1533942 -1.0316372 -3.3450291

C -1.3744012 4.5890118 2.3508539

C -1.5258232 5.5067578 3.3412889

N -0.4730522 5.9291778 4.1258129

C 0.7564358 5.3448368 3.9115549

C 0.9662158 4.4261108 2.9325749

C -0.5885342 -6.6579972 5.5174219

C 7.6366248 -0.0970872 -4.7637081

C -0.6323372 7.0340668 5.0671669

C -7.4215522 -0.2684222 -4.9725141

Co 0.1708778 -0.0204762 -0.1184481

H -3.5656482 2.8829738 -1.9478881

H -2.0286222 4.5277838 -0.4934481

H 2.8709688 4.2162468 0.9368579

H 4.6258198 2.5240358 -0.1771241

H 4.4948768 -2.7711992 -0.2931061

H 2.6772038 -4.3748732 0.8562149

H -2.5135292 -4.3611192 0.1416299

H -4.0333682 -2.6836702 -1.3058951

H 4.5550558 2.0911158 -2.2118461

H 6.3736898 1.9958888 -3.8253751

H 6.2868408 -2.1652872 -3.8956621

H 4.4654998 -2.2384172 -2.2829391

H -2.3506802 -3.9654002 2.2835999

H -2.5115572 -5.4886202 4.1729649

H 1.6191258 -5.4813232 4.7299239

H 1.9587158 -3.9588182 2.8631149

H -5.1709402 1.6288758 -1.4768201

H -7.0473332 1.4568218 -3.0425041

H -5.3048202 -1.7943602 -4.9951671

H -3.3641362 -1.7607632 -3.4822751

H -2.2608532 4.2814068 1.8111939

H -2.4893552 5.9442328 3.5780179

H 1.5440828 5.6507628 4.5911209

H 1.9471178 3.9735068 2.8736459

H -0.5324712 -7.6620732 5.0798779

H 0.2090248 -6.5486162 6.2562249

H -1.5501822 -6.5455292 6.0237749

H 8.5652858 -0.1399222 -4.1851731

H 7.5820748 -0.9662172 -5.4219321

H 7.6334698 0.8086358 -5.3728081

H -1.6371582 7.0076938 5.4957819

H 0.0943338 6.9306778 5.8762269

H -0.4807942 8.0020938 4.5746339

H -7.8925302 0.7101258 -5.0779971

H -7.1129582 -0.6194452 -5.9594621

H -8.1438672 -0.9742232 -4.5481991

H 0.3135328 -0.0057312 -1.5452731

[CoP(H)]^0^  M = 5

C -2.4205702 1.2217041 -0.9538169

C -2.9019412 2.5421671 -1.2095439

C -2.0772202 3.4175021 -0.5198859

C -1.0696172 2.6297911 0.1082051

N -1.2594012 1.3166981 -0.2020499

C -2.9821152 -0.0345829 -1.3556519

C 0.0642958 3.1051031 0.9018841

C 1.3650958 2.5671651 0.5992771

C 2.6360318 3.2276901 0.6797661

C 3.5639558 2.3924491 0.0903141

C 2.8650788 1.2139961 -0.3255339

N 1.5412828 1.3427701 -0.0072179

C 3.4314298 -0.0023999 -0.8839959

C 2.8764148 -1.2583509 -0.4144409

C 3.5696278 -2.4720539 -0.1077199

C 2.6506488 -3.3263859 0.4725921

C 1.3940858 -2.6388709 0.4977331

N 1.5631058 -1.3911259 -0.0529149

C 0.1027358 -3.1445649 0.9058451

C -1.0726522 -2.6722489 0.1829301

C -2.2469412 -3.4089739 -0.1656349

C -3.0794132 -2.5447899 -0.8528899

C -2.4115312 -1.2828669 -0.9235939

N -1.1792352 -1.4039899 -0.3177499

C -0.0224202 -4.0497789 2.0007191

C 4.4905298 0.0397451 -1.8287239

C -0.1350112 4.0719571 1.9279441

C -4.1967182 -0.0805959 -2.1353699

C 5.0416718 1.2654751 -2.3557079

C 6.0790948 1.2772211 -3.2406279

N 6.6420048 0.1217431 -3.7203409

C 6.0798798 -1.0709689 -3.3417739

C 5.0432938 -1.1360859 -2.4581429

C -1.2853472 -4.5491999 2.4880781

C -1.3714412 -5.4433199 3.5149771

N -0.2624902 -5.8967019 4.1821791

C 0.9521728 -5.3580499 3.8437481

C 1.0888998 -4.4615839 2.8246071

C -5.3052282 0.8092591 -1.9491199

C -6.4355332 0.7238761 -2.7121099

N -6.5662812 -0.2122329 -3.7098639

C -5.5288502 -1.0921419 -3.9235129

C -4.3930872 -1.0576969 -3.1652469

C -1.4122882 4.6784451 2.2116541

C -1.5702192 5.6235701 3.1826261

N -0.5313572 6.0286071 3.9822331

C 0.6790688 5.4028451 3.8291021

C 0.8885178 4.4562091 2.8693681

C -0.3674712 -6.9540759 5.1903751

C 7.8351128 0.1591401 -4.5689829

C -0.7017242 7.1277671 4.9357371

C -7.7350192 -0.2111409 -4.5919969

Co 0.1803948 -0.0281329 -0.3295799

H -3.7393432 2.8171741 -1.8335909

H -2.1440962 4.4973771 -0.5044689

H 2.8184978 4.2252031 1.0526581

H 4.6281728 2.5655121 0.0098131

H 4.6263138 -2.6583659 -0.2392639

H 2.8300398 -4.3463879 0.7818011

H -2.4255092 -4.4575389 0.0276311

H -4.0688752 -2.7663069 -1.2278329

H 4.6174698 2.2205801 -2.0815579

H 6.4976678 2.2007181 -3.6230639

H 6.4981948 -1.9577069 -3.8033159

H 4.6188798 -2.1112599 -2.2678999

H -2.2147762 -4.1998849 2.0637021

H -2.3229002 -5.8257809 3.8652561

H 1.7940458 -5.6710729 4.4498581

H 2.0699768 -4.0349829 2.6779361

H -5.2941422 1.5311591 -1.1431199

H -7.2918172 1.3680981 -2.5528439

H -5.6657102 -1.7971779 -4.7342279

H -3.6066952 -1.7675629 -3.3913069

H -2.2943822 4.3821511 1.6622561

H -2.5275942 6.0913171 3.3795281

H 1.4525848 5.6907041 4.5310381

H 1.8495548 3.9627191 2.8672711

H -0.2806802 -7.9403039 4.7227581

H 0.4290228 -6.8337269 5.9270211

H -1.3318442 -6.8790399 5.6963601

H 8.7432728 0.1419441 -3.9574469

H 7.8335468 -0.7053789 -5.2353569

H 7.8240238 1.0693851 -5.1715279

H -1.7147732 7.1031931 5.3422551

H 0.0107258 7.0105861 5.7541351

H -0.5322602 8.0917801 4.4450061

H -7.9501292 -1.2325249 -4.9117679

H -8.5982732 0.1777991 -4.0489169

H -7.5525592 0.4129001 -5.4735629

H 0.3110628 0.0447191 -2.1533679

[CoP(H)]^–1^  M = 2

C 2.2653255 -1.2397560 -1.0554230

C 2.7304575 -2.5589090 -1.3119560

C 1.9697715 -3.4247160 -0.5294040

C 1.0100445 -2.6393790 0.1538890

N 1.1739865 -1.3278390 -0.1891220

C 2.7934285 0.0173390 -1.5003290

C -0.0739205 -3.0922010 1.0339180

C -1.4074175 -2.5795720 0.6997480

C -2.6512815 -3.2312600 0.7284050

C -3.5908085 -2.3592320 0.1358410

C -2.9020765 -1.1967760 -0.2392670

N -1.5758595 -1.3327610 0.1212700

C -3.4092905 0.0408800 -0.8550070

C -2.8790945 1.2847580 -0.2762630

C -3.5313455 2.4845930 0.0429990

C -2.5790485 3.3279950 0.6576900

C -1.3662645 2.6230590 0.6993380

N -1.5629795 1.3763070 0.1309940

C -0.0381675 3.0659950 1.1494280

C 1.0871755 2.6183740 0.3269150

C 2.2374345 3.3421950 -0.0891300

C 2.9867265 2.4959750 -0.8983200

C 2.2931345 1.2554400 -0.9697220

N 1.1314295 1.3668850 -0.2223190

C 0.1492835 3.8647140 2.2753660

C -4.3304605 0.0316640 -1.8903480

C 0.1711105 -3.9800610 2.0754460

C 3.9035525 0.0800430 -2.4212870

C -4.8655805 -1.1857640 -2.5008590

C -5.8104285 -1.1641580 -3.4714960

N -6.3210785 0.0151910 -3.9941340

C -5.7837965 1.2011020 -3.5155370

C -4.8386635 1.2375070 -2.5455150

C 1.4514865 4.2963510 2.7714190

C 1.5902515 5.1246510 3.8375300

N 0.5101595 5.5889660 4.5644810

C -0.7407125 5.1222780 4.2069290

C -0.9367985 4.2981100 3.1468990

C 5.0170415 -0.8256830 -2.4162480

C 6.0380345 -0.7216290 -3.3174590

N 6.0512825 0.2512310 -4.2902260

C 5.0063765 1.1514590 -4.3294600

C 3.9814705 1.0980600 -3.4291930

C 1.4870255 -4.5242910 2.3930130

C 1.6833105 -5.4250160 3.3884330

N 0.6588475 -5.8640570 4.2084190

C -0.5925675 -5.3104450 4.0114370

C -0.8476035 -4.4142400 3.0254460

C 0.6679185 6.6417460 5.5600810

C -7.5255525 0.0133230 -4.8107930

C 0.8556675 -6.9803270 5.1244500

C 7.0836005 0.2695390 -5.3264150

Co -0.2255475 0.0190760 -0.0935540

H 3.5188405 -2.8467660 -1.9914730

H 2.0599075 -4.5019990 -0.4705240

H -2.8345425 -4.2396230 1.0775440

H -4.6529335 -2.5343980 0.0139740

H -4.5808505 2.6996500 -0.1166610

H -2.7352355 4.3457850 0.9930440

H 2.4620805 4.3706300 0.1607270

H 3.9435245 2.7153560 -1.3526370

H -4.4815605 -2.1492940 -2.1900630

H -6.2006905 -2.0737800 -3.9169940

H -6.1531435 2.1020900 -3.9951500

H -4.4313215 2.2028980 -2.2725830

H 2.3564375 3.9318450 2.3033420

H 2.5633325 5.4452340 4.1943680

H -1.5565355 5.4413740 4.8469060

H -1.9406995 3.9359240 2.9685300

H 5.1057595 -1.5742370 -1.6403750

H 6.8994835 -1.3784760 -3.2950310

H 5.0473855 1.8876940 -5.1228620

H 3.1851695 1.8272590 -3.5166100

H 2.3547625 -4.1976550 1.8336640

H 2.6634755 -5.8330040 3.6112810

H -1.3554805 -5.6245190 4.7159180

H -1.8404465 -3.9864220 2.9751500

H 0.6053425 7.6397770 5.1074340

H -0.1149155 6.5496860 6.3173860

H 1.6391165 6.5388140 6.0511500

H -8.4388815 0.0325300 -4.1998080

H -7.5259445 0.8893780 -5.4654980

H -7.5434715 -0.8839350 -5.4359280

H 1.8715725 -6.9496540 5.5274370

H 0.1510195 -6.8991350 5.9561050

H 0.7030375 -7.9466400 4.6262140

H 7.3082845 1.3027450 -5.6003710

H 7.9918495 -0.1979100 -4.9417260

H 6.7495745 -0.2743330 -6.2172130

H -0.3922965 0.0063810 -1.5196680

[CoP(H)]^–1^  M = 4

C 2.7472942 -1.1287330 -0.8684330

C 3.2997272 -2.4140370 -1.1340510

C 2.4352732 -3.3507000 -0.5771280

C 1.3496622 -2.6279440 -0.0174510

N 1.5186622 -1.2954140 -0.2345720

C 3.3113622 0.1579190 -1.1340480

C 0.1655722 -3.1743390 0.6583530

C -1.1283338 -2.7017950 0.1725980

C -2.3272348 -3.4601670 0.0280420

C -3.2699588 -2.6220290 -0.5424100

C -2.6418978 -1.3579480 -0.7463470

N -1.3249378 -1.4402500 -0.3109150

C -3.3019338 -0.1510160 -1.1464910

C -2.7386508 1.1362280 -0.8817240

C -3.2891958 2.4210880 -1.1535830

C -2.4269098 3.3589040 -0.5954910

C -1.3442058 2.6372950 -0.0287040

N -1.5127928 1.3041810 -0.2427800

C -0.1630148 3.1855200 0.6505520

C 1.1328742 2.7115600 0.1718260

C 2.3326152 3.4693720 0.0309560

C 3.2774502 2.6300300 -0.5340470

C 2.6498972 1.3657130 -0.7384770

N 1.3313312 1.4490070 -0.3085890

C -0.2950488 4.1144950 1.6822920

C -4.6070318 -0.2634530 -1.7634940

C 0.2930312 -4.0992760 1.6941360

C 4.6195822 0.2691400 -1.7445460

C -4.9006238 -1.2778390 -2.7346550

C -6.1206688 -1.3721550 -3.3397730

N -7.1508698 -0.5042320 -3.0314510

C -6.9303168 0.4521940 -2.0631990

C -5.7147458 0.5952610 -1.4576650

C 0.8169182 4.6189870 2.4779560

C 0.6557422 5.5506890 3.4524200

N -0.5784338 6.0704420 3.7855630

C -1.6801818 5.5602320 3.1278020

C -1.5778808 4.6241660 2.1497220

C 5.7263822 -0.5885650 -1.4307870

C 6.9457442 -0.4449300 -2.0282560

N 7.1786012 0.5264920 -2.9785350

C 6.1418122 1.3762740 -3.3131240

C 4.9175752 1.2810080 -2.7163680

C 1.5738552 -4.6067940 2.1694690

C 1.6719362 -5.5382660 3.1523240

N 0.5673632 -6.0456270 3.8075790

C -0.6654928 -5.5280290 3.4661600

C -0.8225178 -4.6009190 2.4866710

C -0.7006258 7.2022470 4.6975730

C -8.4790108 -0.6945510 -3.6113370

C 0.6857242 -7.1730780 4.7254530

C 8.4460792 0.5821950 -3.7041010

Co 0.0034512 0.0042190 -0.4528640

H 4.2130322 -2.6260600 -1.6713360

H 2.5429232 -4.4282510 -0.5807860

H -2.4523368 -4.5034180 0.2851560

H -4.3032668 -2.8598840 -0.7553860

H -4.1998508 2.6321660 -1.6956890

H -2.5339848 4.4364840 -0.6028970

H 2.4569552 4.5130930 0.2864710

H 4.3116562 2.8673580 -0.7431630

H -4.1233608 -1.9684100 -3.0395100

H -6.3373818 -2.1080410 -4.1047710

H -7.7828708 1.0690740 -1.8058940

H -5.6200788 1.3474830 -0.6850340

H 1.8119902 4.2217900 2.3279580

H 1.4901512 5.9178820 4.0401320

H -2.6392148 5.9438200 3.4588150

H -2.4964008 4.2464270 1.7192320

H 5.6282342 -1.3370620 -0.6549120

H 7.7962462 -1.0637450 -1.7680110

H 6.3609742 2.1076800 -4.0815050

H 4.1423192 1.9718250 -3.0257400

H 2.4941952 -4.2308510 1.7412900

H 2.6295462 -5.9201350 3.4893880

H -1.5025008 -5.8929380 4.0516030

H -1.8171018 -4.2050420 2.3299940

H -0.6333308 8.1582800 4.1634210

H -1.6623448 7.1536740 5.2144220

H 0.0970262 7.1582520 5.4432950

H -9.0522488 -1.4365870 -3.0432740

H -9.0179478 0.2547230 -3.6055210

H -8.3804098 -1.0347540 -4.6447770

H 1.6454252 -7.1222020 5.2458220

H -0.1148078 -7.1253930 5.4678660

H 0.6203292 -8.1316670 4.1956570

H 8.6900772 1.6215990 -3.9352980

H 9.2397542 0.1660860 -3.0805230

H 8.3880082 0.0110660 -4.6380290

H 0.0075892 0.0029060 -2.3205210

[CoP(H)]^–1^  M = 6

C 2.6084240 -1.2726366 -0.7686997

C 3.1851620 -2.5768336 -0.8455957

C 2.2967380 -3.4504636 -0.2341567

C 1.1619480 -2.6955926 0.1660493

N 1.3499700 -1.3686006 -0.1893087

C 3.2432480 -0.0506116 -1.1021347

C -0.0736450 -3.2025026 0.7200863

C -1.2959950 -2.6239716 0.2161433

C -2.5070690 -3.3026416 -0.0941887

C -3.3490530 -2.3921976 -0.7138377

C -2.6641230 -1.1386216 -0.7399817

N -1.3980790 -1.3032896 -0.2002997

C -3.2195180 0.1034524 -1.1383347

C -2.5895220 1.3311054 -0.8171867

C -3.1668170 2.6333134 -0.9194997

C -2.2848580 3.5172314 -0.3133947

C -1.1530990 2.7697054 0.1087063

N -1.3369120 1.4372374 -0.2274377

C 0.0775700 3.2862874 0.6648603

C 1.3044140 2.7002584 0.1804223

C 2.5179150 3.3740254 -0.1307487

C 3.3667470 2.4526094 -0.7244477

C 2.6829820 1.1982114 -0.7335097

N 1.4112440 1.3722114 -0.2101617

C 0.0747010 4.3462784 1.6169363

C -4.5686410 0.1104414 -1.7601827

C -0.0793160 -4.2479626 1.6883733

C 4.6005880 -0.0672486 -1.7056867

C -4.8158770 -0.4763176 -3.0377887

C -6.0631460 -0.5121026 -3.5971787

N -7.1677490 0.0748684 -2.9581217

C -6.9663310 0.6009294 -1.6715317

C -5.7241690 0.6334524 -1.1020147

C 1.2707190 4.8629304 2.2459293

C 1.2446780 5.9015174 3.1266763

N 0.0678420 6.5103424 3.5082953

C -1.1112610 5.9754824 3.0330943

C -1.1322720 4.9377874 2.1512623

C 5.7448940 -0.5907456 -1.0285197

C 6.9921110 -0.5810406 -1.5877437

N 7.2247170 -0.0033456 -2.8459667

C 6.1123910 0.4853334 -3.5510497

C 4.8601970 0.4721204 -3.0016177

C 1.1226780 -4.8326346 2.2409653

C 1.0934650 -5.8584866 3.1363583

N -0.0900660 -6.3861436 3.6086963

C -1.2631240 -5.7807966 3.2101443

C -1.2808050 -4.7540396 2.3153953

C 0.0732680 7.7256684 4.3173853

C -8.5117760 -0.2874366 -3.3793307

C -0.1033440 -7.5918616 4.4319043

C 8.4464030 -0.3217486 -3.5672757

Co 0.0068650 0.0338444 -0.2664117

H 4.1501050 -2.8102326 -1.2744297

H 2.4019420 -4.5233726 -0.1418147

H -2.6912620 -4.3583896 0.0526843

H -4.3468230 -2.5645896 -1.0941727

H -4.1277960 2.8588904 -1.3612007

H -2.3917140 4.5913294 -0.2383247

H 2.6997890 4.4325164 -0.0017477

H 4.3685490 2.6180484 -1.0971917

H -3.9913660 -0.9175436 -3.5915747

H -6.2664740 -0.9547096 -4.5654807

H -7.8512770 0.9984104 -1.1877557

H -5.6210810 1.0813534 -0.1176417

H 2.2295760 4.4022834 2.0534063

H 2.1449730 6.2866234 3.5918963

H -2.0197120 6.4181244 3.4255173

H -2.0997510 4.5391474 1.8794583

H 5.6294500 -1.0172506 -0.0360977

H 7.8690630 -0.9767136 -1.0882447

H 6.3262160 0.8950944 -4.5315437

H 4.0419470 0.8968404 -3.5772957

H 2.0927370 -4.4382966 1.9720663

H 1.9981960 -6.2965006 3.5423203

H -2.1674920 -6.1582426 3.6737603

H -2.2374010 -4.2945036 2.1094313

H 0.1186160 8.6206544 3.6855533

H -0.8344900 7.7647864 4.9239693

H 0.9390960 7.7167594 4.9834183

H -8.8279230 -1.2587166 -2.9678787

H -9.2192830 0.4777374 -3.0469977

H -8.5518390 -0.3429926 -4.4710537

H 0.8000460 -7.6253706 5.0452953

H -0.9738470 -7.5738866 5.0916333

H -0.1457380 -8.4941086 3.8102693

H 8.6441850 0.4522304 -4.3146467

H 9.2879730 -0.3497176 -2.8690747

H 8.3847340 -1.2945116 -4.0795927

H 0.0127670 0.0220484 -1.7112537
