# Supplementary material for: Self-Assembled Liposomes Enhance Electron Transfer for Efficient Photocatalytic CO2 Reduction
Source: J Am Chem Soc. 2022 May 20;144(21):9399–412. doi: 10.1021/jacs.2c01725 (PMC9164230; doi:10.1021/jacs.2c01725)
Supplement: Supplementary file 2 — ja2c01725_si_002.zip [file ja2c01725_si_002.zip › Coordinates/CoP_H2O/Coord_CoP_H2O.docx]

[CoP(H_2_O)]^+5^  M = 1

C -1.23105763 -2.78296083 -0.14979825

C -0.87408487 -4.17661998 -0.11895599

C 0.47092597 -4.23660630 0.04979392

C 0.94946085 -2.88038057 0.07403970

N -0.10219103 -1.99006414 -0.05554138

C -2.54019768 -2.32099899 -0.24165301

C 2.29187930 -2.53093197 0.18027453

C 2.74987681 -1.21916654 0.09491434

C 4.14164702 -0.85925312 0.05991175

C 4.19661135 0.48684614 -0.10437606

C 2.83885627 0.95947245 -0.15240108

N 1.95240488 -0.09771139 -0.04800382

C 2.49308374 2.30501029 -0.22311534

C 1.18365533 2.76713504 -0.13693479

C 0.82485716 4.15932394 -0.10922219

C -0.52133227 4.21829702 0.05504593

C -0.99943893 2.86270955 0.07971718

N 0.05403573 1.97247815 -0.04179375

C -2.34264218 2.51166812 0.17618505

C -2.79824426 1.20049382 0.08193399

C -4.19098673 0.84091123 0.04225775

C -4.24529215 -0.50404642 -0.12670447

C -2.88588340 -0.97531843 -0.17325947

N -2.00111580 0.08092199 -0.06705245

Co -0.02580951 -0.01000352 0.07984395

H -1.56400053 -5.00228491 -0.20770788

H 1.08567756 -5.11995318 0.13505783

H 4.96836216 -1.54998343 0.13150511

H 5.07716400 1.10763250 -0.17380853

H 1.51416172 4.98575097 -0.19613759

H -1.13669706 5.10152506 0.13721822

H -5.01781981 1.53141734 0.11460066

H -5.12535739 -1.12509545 -0.19985035

H -5.52573389 4.94126632 2.59781946

H -3.84075811 3.11167051 2.39881182

C -4.97307168 4.76889082 1.68314520

C -4.03741229 3.76013918 1.55375220

H -7.24105698 6.18328927 0.95074050

H -6.01731544 7.23932138 1.72187904

C -6.26614622 6.65892411 0.83297795

N -5.24057100 5.60299773 0.65056772

C -3.35379665 3.59668735 0.34029984

H -6.26953433 7.30611795 -0.04183868

C -4.59872916 5.46628277 -0.53084102

C -3.65227303 4.47196937 -0.71088421

H -4.86621469 6.16163273 -1.31530563

H -3.15950050 4.38331360 -1.67136194

H -5.93528587 -4.51823865 -2.59363086

H -4.09843543 -2.83640141 -2.44013303

C -5.36393050 -4.37549992 -1.68532016

C -4.34464323 -3.44835575 -1.58096655

H -7.73983679 -5.57733118 -0.91055390

H -6.61578914 -6.74595721 -1.67085447

C -6.80741579 -6.13134420 -0.79086968

N -5.69492079 -5.16284953 -0.63454793

C -3.64116246 -3.31989601 -0.37464311

H -6.85770066 -6.76098943 0.09520123

C -5.03596483 -5.05778944 0.54077204

C -4.00631482 -4.14521296 0.69572696

H -5.35766904 -5.71117067 1.34093397

H -3.50105627 -4.08034547 1.65160643

H 5.29819261 5.69487825 1.38337812

H 3.44246644 4.05618841 1.68112139

C 4.97841149 5.04184990 0.58146331

C 3.95272089 4.12716192 0.72832029

H 6.42262174 7.08678069 -0.28685545

H 7.60734691 5.75312243 -0.13229315

C 6.75399259 6.13264895 -0.69679009

N 5.64286046 5.15568531 -0.59273223

C 3.59384927 3.30511727 -0.34944658

H 7.02262477 6.25414475 -1.74425842

C 5.32001677 4.37324374 -1.64680870

C 4.30119051 3.44104129 -1.55004928

H 5.89151158 4.51722626 -2.55427016

H 4.06056643 2.83347042 -2.41388021

H 5.46174022 -4.96220431 2.61689864

H 3.77866247 -3.13189762 2.40976073

C 4.91388909 -4.78938020 1.69942966

C 3.97941626 -3.78015429 1.56549228

H 7.18578762 -6.20425409 0.97277923

H 5.96022534 -7.25712744 1.74538331

C 6.21069311 -6.67955885 0.85510845

N 5.18614120 -5.62343931 0.66804793

C 3.30174965 -3.61633551 0.34875644

H 6.21483787 -7.32937438 -0.01776515

C 4.55024320 -5.48622321 -0.51651677

C 3.60502907 -4.49154523 -0.70108409

H 4.82133970 -6.18154133 -1.29976469

H 3.11696816 -4.40256105 -1.66393292

O -0.06177174 -0.02791142 1.98979012

H -0.03732773 0.88820468 2.33437104

H 0.73502500 -0.46784174 2.34905020

[CoP(H_2_O)]^+5^  M = 3

C -2.64654007 1.37275503 0.20700314

C -4.05950546 1.12821712 0.08855626

C -4.21005518 -0.16190244 -0.31790690

C -2.89475062 -0.73526116 -0.39478032

N -1.94597610 0.22204019 -0.08781562

C -2.08639536 2.63215323 0.42546146

C -2.64103987 -2.09334217 -0.58312260

C -1.38815125 -2.66015543 -0.35189520

C -1.12908423 -4.06994656 -0.26519076

C 0.16057431 -4.21543298 0.14393567

C 0.73335797 -2.89837946 0.21624505

N -0.23193909 -1.95187578 -0.07181772

C 2.08513825 -2.62883455 0.43153362

C 2.64537800 -1.36997296 0.21048354

C 4.05764581 -1.12537038 0.09023938

C 4.20749360 0.16417784 -0.31871904

C 2.89256643 0.73769901 -0.39551653

N 1.94332827 -0.21857034 -0.08315698

C 2.63861526 2.09497065 -0.58971774

C 1.38507542 2.66254426 -0.36363875

C 1.12795329 4.07301860 -0.27421386

C -0.16084555 4.21865368 0.13673553

C -0.73492659 2.90126480 0.20690562

N 0.22800710 1.95602329 -0.08710795

Co -0.00250283 0.00311913 0.01775279

H -4.83610082 1.85969656 0.25643443

H -5.13318079 -0.68671166 -0.51434452

H -1.84369595 -4.85434944 -0.46295535

H 0.67974162 -5.13957689 0.34823849

H 4.83433135 -1.85657570 0.25899049

H 5.13058928 0.68826186 -0.51719076

H 1.84355817 4.85693204 -0.47029482

H -0.67841698 5.14274905 0.34492244

H 5.79526050 5.45150651 0.26113229

H 3.87435151 4.02873369 0.95194565

C 5.36607529 4.71469183 -0.40484809

C 4.29714229 3.91716760 -0.03890893

H 7.04733181 6.37327009 -1.43200697

H 8.01425161 4.89331416 -1.70752707

C 7.10547966 5.43363131 -1.97944984

N 5.93238640 4.59662188 -1.62769021

C 3.78658535 2.97634973 -0.94390482

H 7.09027686 5.63071666 -3.05059038

C 5.46870361 3.68791074 -2.51654874

C 4.40093190 2.86831381 -2.19931611

H 5.97054196 3.64432732 -3.47436689

H 4.04717000 2.15682922 -2.93541152

H -4.36640700 6.66446052 -0.30033057

H -2.81983000 4.86489589 -1.04407616

C -4.13085274 5.82143620 0.33542631

C -3.26870502 4.81348013 -0.05982791

H -5.61515006 7.67098273 1.23190626

H -6.67608072 6.44922350 1.98746278

C -5.66583422 6.86012912 1.95586238

N -4.72815225 5.78585537 1.54732490

C -3.00286980 3.74347318 0.80474093

H -5.37434027 7.22509112 2.94111308

C -4.50005903 4.75756310 2.39846711

C -3.64637140 3.72806754 2.05174441

H -5.01493385 4.79418426 3.34996961

H -3.47775284 2.92329620 2.75678862

H 4.99285755 -4.80096975 3.36639146

H 3.45757350 -2.93304776 2.77169828

C 4.48860334 -4.76344504 2.40993917

C 3.63324225 -3.73221269 2.06203533

H 6.51611905 -6.84964383 1.26274629

H 5.96736738 -6.74613920 2.95863264

C 5.65073396 -6.88114742 1.92627572

N 4.72587848 -5.78254731 1.55455699

C 3.00106626 -3.74049568 0.81200167

H 5.12663826 -7.83163005 1.82045134

C 4.13634410 -5.81156060 0.33580053

C 3.27554245 -4.80581512 -0.05922596

H 4.38320346 -6.65216424 -0.29985835

H 2.83304254 -4.85088871 -1.04669223

H -5.79595362 -5.45499950 0.25601815

H -3.88082543 -4.02825676 0.95506546

C -5.36529364 -4.71734087 -0.40811496

C -4.29968066 -3.91752224 -0.03756020

H -7.03081825 -6.38520622 -1.45035314

H -8.00641366 -4.90883367 -1.71505725

C -7.09460503 -5.44197847 -1.99098814

N -5.92616735 -4.60085993 -1.63367845

C -3.78716699 -2.97566588 -0.94042109

H -7.07861538 -5.63133069 -3.06347539

C -5.46109808 -3.69067144 -2.52024563

C -4.39683748 -2.86840870 -2.19818205

H -5.95864241 -3.64828040 -3.48034810

H -4.04153609 -2.15607389 -2.93270630

O -0.02390845 0.06295791 2.13886940

H 0.86048952 0.18967376 2.52769208

H -0.39025401 -0.73987154 2.55154804

[CoP(H_2_O)]^+4^ M = 2

C -2.65101977 1.50388420 -0.03767131

C -4.08133263 1.29772230 -0.06267115

C -4.28280223 -0.04324648 -0.08238389

C -2.97605160 -0.66094611 -0.08830658

N -1.98713755 0.29745548 -0.05766523

C -2.04652144 2.76395981 -0.03758795

C -2.76761109 -2.04293549 -0.08000436

C -1.50796541 -2.64836065 -0.05722702

C -1.29890699 -4.07774043 -0.07176489

C 0.04269191 -4.27759026 -0.05464844

C 0.65943556 -2.97108950 -0.05509709

N -0.30113897 -1.98240509 -0.05148951

C 2.04089178 -2.76023742 -0.03177229

C 2.64616890 -1.50029717 -0.03286415

C 4.07575167 -1.29307431 -0.06003054

C 4.27698066 0.04827066 -0.08157348

C 2.97109722 0.66602603 -0.08589676

N 1.98152983 -0.29288733 -0.05267150

C 2.76097802 2.04770850 -0.07976645

C 1.50072182 2.65178702 -0.05940539

C 1.29252613 4.08202900 -0.07211598

C -0.04862065 4.28201094 -0.05662315

C -0.66508921 2.97457976 -0.06000350

N 0.29448294 1.98667698 -0.05746277

Co -0.00433624 0.00374424 0.00940472

H -4.83276943 2.07312656 -0.07232603

H -5.22919793 -0.56308141 -0.08710364

H -2.07246403 -4.83058653 -0.09716168

H 0.56287344 -5.22369736 -0.04060176

H 4.82766840 -2.06791257 -0.07013963

H 5.22346227 0.56795121 -0.08852038

H 2.06648791 4.83453242 -0.09526337

H -0.56906949 5.22790238 -0.04167374

H 5.65332174 5.23072118 1.76959590

H 3.64083177 3.77059375 1.87701037

C 5.37888617 4.58082406 0.94920929

C 4.26420788 3.76279861 0.99128714

H 7.42692984 6.05495164 0.73949592

H 8.27225542 4.84975537 -0.26892887

C 7.38058949 5.47164954 -0.17805169

N 6.18695678 4.59417379 -0.13497013

C 3.95779123 2.93374686 -0.09740417

H 7.29573310 6.13849323 -1.03713663

C 5.91954401 3.80291297 -1.20181220

C 4.81860991 2.96986024 -1.20697694

H 6.60622980 3.87146261 -2.03591729

H 4.62701861 2.35621575 -2.07871057

H -3.88806397 6.64792312 -1.89433150

H -2.38231044 4.66898351 -1.99614506

C -3.81577455 5.94646347 -1.07367174

C -2.98454315 4.84159206 -1.11259160

H -5.31169651 7.97770019 -0.84509138

H -6.52222963 7.02530542 0.05737745

C -5.48755872 7.37124182 0.04120236

N -4.58964506 6.19259182 0.00765530

C -2.93425170 3.95952469 -0.02350926

H -5.26982218 7.95407991 0.93694162

C -4.56616789 5.35774961 1.07448139

C -3.75246826 4.24235151 1.08272757

H -5.20805995 5.62043018 1.90574183

H -3.74972839 3.59926351 1.95428766

H 5.20986052 -5.60900096 1.90525823

H 3.74328194 -3.59923123 1.95877747

C 4.56790864 -5.35228726 1.07300090

C 3.74683170 -4.23915370 1.08492315

H 6.19459729 -7.26640419 -0.82648280

H 6.01045982 -7.42870385 0.94062520

C 5.48054965 -7.36930555 -0.00806679

N 4.59479099 -6.18104118 0.00490343

C 2.92878739 -3.95578488 -0.01817756

H 4.87040707 -8.26242462 -0.14870496

C 3.81557483 -5.93530970 -1.07602734

C 2.98017806 -4.83668784 -1.11131716

H 3.89523975 -6.63735882 -1.89625505

H 2.37630075 -4.66279129 -1.99350600

H -5.66211553 -5.22630115 1.76544687

H -3.64881036 -3.76750855 1.87484073

C -5.38712366 -4.57566795 0.94582534

C -4.27191054 -3.75844099 0.98894022

H -7.43616549 -6.04825414 0.73406185

H -8.28073192 -4.84059542 -0.27204696

C -7.38959993 -5.46345874 -0.18252122

N -6.19517937 -4.58713569 -0.13838551

C -3.96485655 -2.92819607 -0.09870980

H -7.30565391 -6.12891520 -1.04277435

C -5.92716339 -3.79475796 -1.20424998

C -4.82570371 -2.96243883 -1.20836123

H -6.61390865 -3.86178778 -2.03843005

H -4.63369592 -2.34786360 -2.07934682

O 0.03450022 -0.03032022 2.26976299

H 0.98360000 -0.05023423 2.48223977

H -0.28247959 -0.92016140 2.50185903

[CoP(H_2_O)]^+4^ M = 4

C 2.96755115 -0.44639419 0.35185799

C 4.21472971 0.26859712 0.42484497

C 3.94759613 1.55675534 0.07047575

C 2.52970861 1.62879022 -0.20365638

N 1.93957910 0.43365299 -0.01482537

C 2.82485643 -1.82007788 0.43958596

C 1.84233075 2.86110101 -0.53258570

C 0.46572266 3.00773109 -0.35335051

C -0.27690779 4.24883176 -0.44694278

C -1.54975850 3.97983749 -0.05063953

C -1.61806887 2.56118242 0.18957156

N -0.39198297 1.97711139 -0.00153990

C -2.80334777 1.85834501 0.51308263

C -2.94788628 0.48388749 0.43627117

C -4.19331314 -0.23015546 0.53927130

C -3.93567231 -1.51910274 0.18026631

C -2.52493063 -1.59394562 -0.12548262

N -1.92817710 -0.39839347 0.05025837

C -1.84732224 -2.82602958 -0.47182723

C -0.46582829 -2.97150484 -0.33395214

C 0.27258918 -4.21513652 -0.43912110

C 1.55523046 -3.94496814 -0.07842186

C 1.63104661 -2.52379900 0.15013104

N 0.40230100 -1.94040907 -0.01394341

Co 0.00787809 0.01681966 0.05624033

H 5.17331666 -0.15918478 0.68054690

H 4.64175756 2.38228150 0.01839345

H 0.11304601 5.20380228 -0.76210313

H -2.36652943 4.68085239 0.03637548

H -5.14523135 0.19903070 0.81662372

H -4.63220749 -2.34347339 0.14503319

H -0.12706893 -5.17179298 -0.73643594

H 2.37293279 -4.64654598 -0.00718058

H -3.43086716 -7.24015285 -0.37026831

H -1.98905732 -5.43902466 0.51036776

C -3.44326423 -6.24769516 -0.80169258

C -2.63394050 -5.23307094 -0.33405540

H -5.02745272 -8.01466909 -1.70918653

H -6.19603855 -6.82354600 -2.33452795

C -5.15139116 -7.13802626 -2.34252989

N -4.31164926 -6.03904867 -1.82063377

C -2.68853793 -3.94644057 -0.91087268

H -4.83847075 -7.37102858 -3.36239407

C -4.41007432 -4.80596978 -2.38652687

C -3.63165454 -3.76013705 -1.94854130

H -5.11917448 -4.70565933 -3.19836666

H -3.73750821 -2.79635517 -2.43190028

H 6.38513707 -4.73346111 -0.46000560

H 4.36498997 -3.46514771 -1.16621945

C 5.83455552 -4.11209559 0.23385165

C 4.70711396 -3.40270605 -0.14060796

H 7.78862254 -5.48623500 1.09385945

H 8.33719841 -4.07536773 2.04128726

C 7.52706170 -4.79027805 1.88854652

N 6.30337349 -4.04807955 1.50036420

C 4.03619726 -2.61031279 0.80001362

H 7.32820997 -5.33889023 2.80938891

C 5.67955900 -3.28403965 2.42853272

C 4.54945456 -2.55806503 2.10456543

H 6.11337252 -3.27952255 3.42024008

H 4.06999742 -1.95874651 2.86885527

H -6.01169603 3.35033141 3.56179205

H -3.98079939 2.03309501 2.97429712

C -5.61018016 3.34356795 2.55713274

C -4.48292778 2.61747069 2.21299728

H -8.32239351 4.47135795 1.43607206

H -7.65886590 4.73862764 3.07092273

C -7.47454915 4.85607924 2.00494378

N -6.26243411 4.08597326 1.63488761

C -4.00664520 2.65041067 0.89651497

H -7.30807046 5.90823706 1.77054641

C -5.82676040 4.13244939 0.35333335

C -4.70728971 3.42554112 -0.04011419

H -6.40497898 4.74006571 -0.33100397

H -4.39265428 3.47184696 -1.07537351

H 3.42533570 7.27595581 -0.48974934

H 2.00774417 5.47783997 0.43491590

C 3.42692431 6.28187591 -0.91759920

C 2.63040258 5.26885953 -0.42529344

H 4.98693938 8.04558662 -1.87210593

H 6.13886454 6.85233648 -2.52369338

C 5.09432544 7.16675547 -2.50544777

N 4.26867505 6.06960346 -1.95794785

C 2.67061970 3.97991758 -0.99837199

H 4.75482933 7.39624492 -3.51758560

C 4.35288810 4.83430872 -2.52149018

C 3.58677211 3.78984891 -2.05933970

H 5.04082219 4.73109847 -3.35097743

H 3.68085805 2.82413241 -2.54126354

O 0.08122117 0.04700299 2.30761322

H -0.72184715 -0.39436594 2.63291633

H -0.03545081 0.98182817 2.54955382

[CoP(H_2_O)]^+3^ M = 1

C -2.62011558 1.24184925 -0.77366170

C -3.23263513 2.53326639 -0.85860901

C -2.37651345 3.42921897 -0.26904552

C -1.20949471 2.69508328 0.12913877

N -1.35391929 1.38181689 -0.22012404

C -3.18483150 0.00399635 -1.11969419

C 0.00728164 3.22839995 0.68267762

C 1.23287367 2.68159183 0.17468721

C 2.44903600 3.38693818 -0.12119940

C 3.29811183 2.49012993 -0.71777241

C 2.62334676 1.22712736 -0.75831175

N 1.34664730 1.38147602 -0.24133140

C 3.18504323 -0.00801940 -1.12043968

C 2.58983000 -1.24350816 -0.83809672

C 3.16061848 -2.54782376 -0.98989065

C 2.32492517 -3.42750921 -0.34537157

C 1.20854355 -2.67224698 0.14888313

N 1.36921358 -1.36442104 -0.17358568

C -0.00584414 -3.20350335 0.73765620

C -1.23120346 -2.65997977 0.18835126

C -2.39647160 -3.39491855 -0.21964410

C -3.22899765 -2.51465605 -0.86605532

C -2.59451013 -1.23120470 -0.82335380

N -1.36221259 -1.36261890 -0.18790614

C 0.00938753 -4.23638768 1.69930235

C 4.52025852 0.01168407 -1.76852146

C -0.00864746 4.28394678 1.66052337

C -4.52050529 -0.01395912 -1.76635079

C 4.70990926 0.70041131 -2.97908267

C 5.95104396 0.71315866 -3.58303074

N 7.00367907 0.07176533 -3.02046359

C 6.85681770 -0.59047937 -1.85036356

C 5.63260594 -0.63508361 -1.20879378

C -1.18337996 -4.76184223 2.31346649

C -1.13862225 -5.77461316 3.22687016

N 0.04222287 -6.32926424 3.64084980

C 1.20902880 -5.80277670 3.15673942

C 1.22348433 -4.78969495 2.24267205

C -5.65069188 0.55658648 -1.16131976

C -6.87455592 0.52015270 -1.80388433

N -7.00483323 -0.06377832 -3.01661310

C -5.93479931 -0.63270129 -3.62314511

C -4.69266643 -0.62329413 -3.02165541

C -1.21702301 4.83351051 2.19351467

C -1.20469287 5.83542936 3.12726204

N -0.04102670 6.33733921 3.62699994

C 1.13445651 5.78337901 3.22167482

C 1.17613456 4.78055807 2.28834227

C 0.05339911 -7.49267875 4.53467178

C 8.32432732 0.13456119 -3.68984443

C -0.06572125 7.46751903 4.56937309

C -8.31507607 -0.08427244 -3.70849656

Co 0.00100356 0.01385155 -0.05132638

H -4.18044540 2.75745910 -1.32647563

H -2.50318997 4.49983005 -0.20390452

H 2.61826171 4.44445994 0.01650033

H 4.29203765 2.68789396 -1.09406955

H 4.08171634 -2.78403817 -1.50344481

H 2.42680366 -4.50192851 -0.28515914

H -2.53902496 -4.45932851 -0.09876680

H -4.19865782 -2.72831624 -1.29365222

H 3.88636937 1.21654203 -3.45694983

H 6.14079441 1.22201622 -4.51949975

H 7.73993356 -1.06906542 -1.44786018

H 5.55376402 -1.16336117 -0.26703819

H -2.15380267 -4.34470130 2.08207785

H -2.03626423 -6.18027436 3.67806324

H 2.12211673 -6.23126694 3.55212710

H 2.18820680 -4.39734785 1.95150351

H -5.58654485 1.01763639 -0.18383504

H -7.77008889 0.94397996 -1.36923769

H -6.11179553 -1.07823825 -4.59382082

H -3.85534001 -1.08036499 -3.53430644

H -2.18295849 4.45433243 1.89490061

H -2.11774626 6.26692249 3.51835511

H 2.03255826 6.17073314 3.68631163

H 2.14463335 4.35592642 2.06965992

H -0.00586419 -8.41880245 3.95466123

H 0.97450484 -7.49022084 5.11953527

H -0.79851300 -7.43579727 5.21437639

H 8.72617033 1.14343384 -3.58154779

H 8.99386607 -0.58612557 -3.22416039

H 8.19685473 -0.10900855 -4.74481576

H -0.88463763 7.32951242 5.27724566

H 0.87809738 7.50071808 5.11310539

H -0.20612445 8.40219112 4.02015951

H -9.06937460 0.35021527 -3.05528105

H -8.23713131 0.50022305 -4.62641762

H -8.57628083 -1.11755688 -3.94009020

O -0.00444187 -0.10893322 1.99229307

H -0.79199310 -0.62907765 2.24081568

H 0.75855237 -0.66432838 2.24119046

[CoP(H_2_O)]^+3^ M = 3

C 2.63036487 -1.22567535 -0.75269176

C 3.23963206 -2.51887338 -0.89635415

C 2.39531331 -3.42320163 -0.30857271

C 1.23976598 -2.69023496 0.14485928

N 1.38459628 -1.37385840 -0.15787880

C 3.20033776 0.01971066 -1.06115967

C 0.02602758 -3.25497731 0.68185890

C -1.21071290 -2.70307634 0.19242116

C -2.40054383 -3.43819493 -0.16162685

C -3.26129326 -2.55032607 -0.74979828

C -2.61600428 -1.26650597 -0.72718127

N -1.35522313 -1.40025813 -0.16890767

C -3.20752565 -0.03384866 -1.05191928

C -2.63938478 1.21615527 -0.75637860

C -3.25000568 2.50752193 -0.90389001

C -2.40333618 3.41651958 -0.32496659

C -1.24488381 2.68802913 0.12486999

N -1.39056241 1.36885368 -0.17051344

C -0.02842004 3.25196079 0.65956503

C 1.20782099 2.70028269 0.16542329

C 2.39747245 3.43165879 -0.19217601

C 3.25764086 2.53937530 -0.77649608

C 2.61180193 1.25696978 -0.74711149

N 1.35106429 1.39461420 -0.19030872

C -0.05362921 4.33751362 1.59572554

C -4.54776529 -0.06762925 -1.68978261

C 0.05456149 -4.34190663 1.61676499

C 4.54107361 0.04604874 -1.69824697

C -4.73261249 -0.72397712 -2.91923279

C -5.97661134 -0.74173526 -3.51686086

N -7.03722753 -0.13433429 -2.93198070

C -6.89602198 0.49345571 -1.74212488

C -5.66997410 0.53956321 -1.10481477

C 1.12900779 4.87446711 2.19956915

C 1.07833871 5.91798704 3.08439103

N -0.10357821 6.47877393 3.46791272

C -1.26358662 5.93868515 2.99796622

C -1.26754335 4.89421937 2.11288133

C 5.66166620 -0.55560665 -1.10510143

C 6.89055517 -0.51031069 -1.73732632

N 7.03559221 0.11173269 -2.92930090

C 5.97513185 0.70928748 -3.52511841

C 4.72881381 0.69266561 -2.93269488

C 1.27019652 -4.89783692 2.13057669

C 1.26961615 -5.94382714 3.01400787

N 0.11139117 -6.48582009 3.48589100

C -1.07195967 -5.92530289 3.10669027

C -1.12577956 -4.88033560 2.22363208

C -0.12514842 7.66046415 4.34380596

C -8.35965919 -0.19524582 -3.59778182

C 0.13643225 -7.66844277 4.36046609

C 8.35037739 0.13881688 -3.61205916

Co -0.00273942 -0.00338749 -0.04529811

H 4.17861264 -2.73209634 -1.38632455

H 2.51602310 -4.49631322 -0.27346290

H -2.54074567 -4.50426698 -0.05850005

H -4.24523754 -2.76161596 -1.14391704

H -4.19197608 2.71755099 -1.38948428

H -2.52554327 4.48960698 -0.29373607

H 2.53895643 4.49800391 -0.09332976

H 4.24201818 2.74769224 -1.17120828

H -3.90255501 -1.20949899 -3.41741233

H -6.16154652 -1.22600475 -4.46716727

H -7.78532346 0.94409100 -1.32137086

H -5.59629181 1.04072755 -0.14791490

H 2.10112241 4.44932064 1.99724775

H 1.97252685 6.33694579 3.52909904

H -2.18066136 6.37526373 3.37357044

H -2.22959878 4.48744837 1.83805824

H 5.58548714 -1.04866254 -0.14420079

H 7.77886988 -0.95680532 -1.31065575

H 6.16321863 1.18359238 -4.47996531

H 3.89974746 1.17394520 -3.43662754

H 2.23121862 -4.48909515 1.85522391

H 2.18808691 -6.37983569 3.38683810

H -1.96455391 -6.34531153 3.55356932

H -2.09868931 -4.45491662 2.02601063

H -0.08315570 8.57030660 3.73893059

H -1.04242371 7.65439541 4.93348953

H 0.73357444 7.62668673 5.01511804

H -8.76258748 -1.20391340 -3.49092838

H -9.02701910 0.52553921 -3.12898226

H -8.23523890 0.05117731 -4.65243449

H 1.05444472 -7.66121331 4.94900006

H -0.72153429 -7.63710771 5.03284325

H 0.09547779 -8.57762190 3.75453514

H 8.60631366 1.17300496 -3.84497300

H 9.10325929 -0.28835010 -2.95225102

H 8.28272415 -0.44887808 -4.52889106

O 0.02829458 0.13052887 2.21906891

H -0.60755563 0.84300208 2.40402862

H 0.89736264 0.53388966 2.38586422

[CoP(H_2_O)]^+3^ M = 5

C 2.58428018 -1.06115533 -0.91260319

C 3.18605908 -2.34354723 -1.19954254

C 2.41954699 -3.28645868 -0.57676430

C 1.32296490 -2.58594078 0.06193459

N 1.40321038 -1.27272691 -0.19416956

C 3.10233345 0.20608675 -1.15187296

C 0.20941850 -3.18238165 0.80003795

C -1.09371340 -2.72862570 0.44463180

C -2.31986490 -3.47304076 0.40806759

C -3.26932129 -2.65397737 -0.15830196

C -2.62604009 -1.40787055 -0.45825375

N -1.31852831 -1.47128750 -0.09338106

C -3.28643851 -0.21433608 -0.97862329

C -2.75165608 1.07181035 -0.72758075

C -3.28479710 2.35552446 -1.08325399

C -2.45265532 3.31014456 -0.53330002

C -1.40108704 2.60788655 0.12142010

N -1.55684946 1.26924276 -0.03246726

C -0.21676359 3.20930784 0.76062806

C 1.04440546 2.74963909 0.29853926

C 2.25156283 3.51243001 0.08663807

C 3.13225083 2.68793801 -0.56188125

C 2.48478542 1.40932629 -0.69710425

N 1.21778005 1.47648823 -0.18770924

C -0.34870490 4.24659327 1.74336271

C -4.52958744 -0.39776862 -1.69185962

C 0.44218825 -4.18119100 1.80091074

C 4.40667541 0.33343556 -1.84626360

C -4.70976599 -1.48913807 -2.59418414

C -5.87229037 -1.64944692 -3.29871616

N -6.92513689 -0.78773266 -3.14584242

C -6.81334444 0.24365803 -2.25983780

C -5.66000864 0.45994911 -1.55050992

C 0.75708467 4.71559867 2.51512768

C 0.60674964 5.70739157 3.45002798

N -0.60809461 6.26601456 3.70723980

C -1.70461381 5.80084025 3.04717356

C -1.60858497 4.81523386 2.09835826

C 5.55820797 -0.33050041 -1.39653914

C 6.75134755 -0.19138863 -2.08188089

N 6.83067676 0.58483579 -3.18612715

C 5.73978305 1.25077493 -3.63776384

C 4.52710935 1.14602707 -2.98771345

C 1.73655693 -4.71530365 2.09456632

C 1.91727909 -5.67206050 3.05782740

N 0.87987791 -6.14064257 3.80869110

C -0.35782653 -5.60109879 3.62551595

C -0.59240678 -4.64364644 2.67458434

C -0.73429660 7.38152268 4.66207152

C -8.17731497 -1.02890912 -3.87905282

C 1.09803399 -7.22627315 4.77859766

C 8.10352475 0.70224364 -3.93558750

Co -0.05842539 0.00271198 -0.00868435

H 4.07210774 -2.51313789 -1.79399568

H 2.55503371 -4.35830294 -0.59701871

H -2.44278087 -4.50891299 0.68895118

H -4.30659835 -2.89140761 -0.34529973

H -4.16008465 2.54453391 -1.68547228

H -2.54206138 4.38434140 -0.61843673

H 2.38469238 4.56117611 0.30960651

H 4.12917077 2.93414957 -0.89865954

H -3.90392525 -2.19093134 -2.77093364

H -6.01142443 -2.44780335 -4.01687659

H -7.68994176 0.86737666 -2.13752007

H -5.65366629 1.26654780 -0.82982597

H 1.73717938 4.27129078 2.41396465

H 1.43866848 6.07653876 4.03598650

H -2.65163157 6.25237884 3.31490311

H -2.52340779 4.47758396 1.63221659

H 5.53660141 -0.94232697 -0.50364110

H 7.66122664 -0.68491901 -1.76661389

H 5.87624048 1.85299168 -4.52689108

H 3.67364410 1.68722361 -3.37716699

H 2.61933832 -4.36907707 1.57692071

H 2.89078728 -6.09530606 3.27159021

H -1.13890892 -5.95966204 4.28383503

H -1.58392572 -4.21634642 2.63555543

H -0.69184157 8.33039225 4.12129946

H -1.68565605 7.29807180 5.18816810

H 0.08273727 7.33100064 5.38154625

H -8.73157876 -1.84607345 -3.40926468

H -8.78166075 -0.12225894 -3.86688369

H -7.94485494 -1.29038198 -4.91305011

H 2.05446572 -7.07439083 5.28102638

H 0.29684878 -7.21288558 5.51742227

H 1.10245279 -8.18822059 4.25922284

H 8.31476695 1.75766916 -4.10875496

H 8.90517031 0.25743416 -3.34912959

H 8.00052717 0.17680696 -4.88667520

O 0.00635545 0.02358010 2.24553587

H 0.67658359 0.68188730 2.49662499

H -0.84276566 0.44255284 2.46840391

[CoP(H_2_O)]^+2^ M = 2

C 2.64009112 -1.23179948 -0.81639090

C 3.22848470 -2.53010378 -0.95589756

C 2.36103055 -3.42610774 -0.37386627

C 1.22456351 -2.67835565 0.08145948

N 1.38979917 -1.36679281 -0.21563363

C 3.21497230 0.01400928 -1.11343302

C -0.00151941 -3.20547063 0.66776305

C -1.23312751 -2.67075178 0.10736713

C -2.41587119 -3.39750079 -0.26051085

C -3.27474238 -2.50029995 -0.85115873

C -2.62404052 -1.22374715 -0.82812712

N -1.36546738 -1.36992562 -0.25313339

C -3.20051268 0.02149814 -1.12581844

C -2.62581270 1.26448237 -0.80570625

C -3.21098484 2.56164518 -0.95072200

C -2.34531317 3.45881239 -0.36509378

C -1.21421684 2.71224634 0.10043418

N -1.38035318 1.39753327 -0.19026063

C 0.01022276 3.24977185 0.67902043

C 1.24424215 2.70559356 0.13296656

C 2.42438761 3.43212880 -0.24015028

C 3.28626070 2.53273855 -0.82584639

C 2.63807898 1.25691790 -0.79778908

N 1.37993861 1.40232665 -0.21770865

C -0.00596534 4.26037851 1.65271197

C -4.54237788 0.00620045 -1.75593099

C 0.01128049 -4.22103220 1.64472595

C 4.55942852 0.02957604 -1.73900615

C -4.74905544 -0.65991046 -2.97792543

C -5.99975908 -0.67540265 -3.55981072

N -7.04999154 -0.05523662 -2.96815096

C -6.88799665 0.58642052 -1.78773522

C -5.65403655 0.63093924 -1.16703154

C 1.18768694 4.78902326 2.27878962

C 1.14113522 5.78534311 3.20562790

N -0.04239137 6.33416256 3.63422149

C -1.20984709 5.80483024 3.14194273

C -1.22264804 4.80783709 2.21478035

C 5.66580840 -0.59717095 -1.14275033

C 6.90079034 -0.56758922 -1.76252510

N 7.06849246 0.06391165 -2.94729464

C 6.02344760 0.68669003 -3.54566641

C 4.77219818 0.68663290 -2.96458889

C 1.22485221 -4.77014173 2.20283688

C 1.20877010 -5.77340885 3.12555368

N 0.04102546 -6.30085413 3.61142640

C -1.13949115 -5.75008689 3.18585089

C -1.18305789 -4.74729327 2.26345975

C -0.05629370 7.49052937 4.53115298

C -8.38062219 -0.11910311 -3.61514254

C 0.05139739 -7.46235997 4.50586496

C 8.38669935 0.06282998 -3.62222417

Co 0.00822831 0.01592340 -0.09406865

H 4.17142353 -2.75792131 -1.43273242

H 2.47048468 -4.50019863 -0.31652786

H -2.56586316 -4.46086566 -0.13766592

H -4.26328892 -2.70491944 -1.23860921

H -4.14994551 2.78883479 -1.43583554

H -2.45336866 4.53341937 -0.31160263

H 2.57178772 4.49668875 -0.12299397

H 4.27414855 2.73616365 -1.21601265

H -3.92859992 -1.15922772 -3.47810811

H -6.20079791 -1.16960376 -4.50180226

H -7.76906830 1.04693766 -1.36034142

H -5.56411719 1.14071956 -0.21624556

H 2.15889083 4.37616696 2.04196001

H 2.03824932 6.18341096 3.66559276

H -2.12386349 6.21967513 3.55080569

H -2.18644768 4.41330775 1.92243577

H 5.57080001 -1.09863031 -0.18804667

H 7.77714209 -1.03447953 -1.33253531

H 6.22932003 1.16710903 -4.49375163

H 3.95604275 1.18908251 -3.46871983

H 2.18986566 -4.37882862 1.91087916

H 2.12147644 -6.19518554 3.52958015

H -2.03826780 -6.15225040 3.63841659

H -2.15361038 -4.33466126 2.02431813

H -0.01703771 8.42553461 3.96169698

H -0.96849143 7.47428706 5.13086516

H 0.80485403 7.44229596 5.20074215

H -8.77099064 -1.13412548 -3.52268408

H -9.05022461 0.58334799 -3.12212727

H -8.27553498 0.14903283 -4.66684397

H 0.96567069 -7.45245843 5.10175441

H -0.80806875 -7.41162851 5.17679384

H 0.00572879 -8.39171321 3.92913961

H 8.63570539 1.08340169 -3.91477844

H 9.13957960 -0.31659837 -2.93362356

H 8.33174271 -0.57880808 -4.50341600

O -0.09620638 -0.24036913 2.19648509

H -1.04656070 -0.07037478 2.30966314

H -0.02575382 -1.21281304 2.22041647

[CoP(H_2_O)]^+2^ M = 4

C -2.67786426 1.25101287 -0.57506694

C -3.21036029 2.56315686 -0.77600093

C -2.33383004 3.45379997 -0.18899624

C -1.23298158 2.69339266 0.30355439

N -1.43574626 1.36651084 0.03499575

C -3.28315646 -0.00877949 -0.88676449

C 0.00421734 3.22773399 0.83918928

C 1.22677235 2.67315773 0.32090170

C 2.43034813 3.39361942 -0.01598264

C 3.25625990 2.51443499 -0.66583919

C 2.57895667 1.24657392 -0.69416527

N 1.33476003 1.38556626 -0.10427004

C 3.13825358 0.01482507 -1.08101755

C 2.56357258 -1.23691603 -0.78992381

C 3.14558033 -2.53439078 -0.97941393

C 2.32309081 -3.43503168 -0.34735505

C 1.20814872 -2.69395630 0.17751591

N 1.34880241 -1.37837237 -0.13679543

C 0.03470427 -3.22498874 0.83368344

C -1.23919146 -2.67180448 0.44228489

C -2.47594366 -3.37037573 0.28687866

C -3.36935567 -2.48916664 -0.28557285

C -2.68452036 -1.24768217 -0.46621653

N -1.38796766 -1.38645129 -0.02351626

C 0.14343254 -4.26334090 1.81160900

C 4.46120991 0.05098789 -1.74546010

C 0.00142389 4.29420957 1.78849967

C -4.56149887 -0.07599080 -1.55824202

C 4.64452938 0.78314758 -2.93385940

C 5.87841660 0.81597123 -3.54906372

N 6.93705811 0.15231825 -3.02197996

C 6.79838458 -0.55322144 -1.87527749

C 5.58131864 -0.61939788 -1.22483343

C -0.97524059 -4.72353414 2.58550149

C -0.85662342 -5.72393221 3.51012449

N 0.34112083 -6.32403013 3.77455306

C 1.45520583 -5.85637028 3.13635392

C 1.39062776 -4.85421273 2.20836594

C -5.65657399 0.80514388 -1.29539272

C -6.84699517 0.69071527 -1.96113743

N -7.04503599 -0.26254340 -2.92341121

C -6.02316770 -1.13480209 -3.20771088

C -4.82684984 -1.07371045 -2.54861306

C -1.20505946 4.88233004 2.29886942

C -1.18439200 5.90884664 3.20161724

N -0.01542723 6.41038681 3.69935819

C 1.15760425 5.82115792 3.31957478

C 1.19172492 4.79294028 2.41923146

C 0.42973750 -7.46403565 4.69674352

C 8.25105613 0.24058856 -3.69848407

C -0.01369375 7.57644394 4.59269183

C -8.29572884 -0.32302190 -3.68828027

Co -0.03497731 0.00081919 -0.05606807

H -4.11528105 2.81776812 -1.30570430

H -2.41159931 4.53231825 -0.18211009

H 2.60274612 4.44847713 0.14057951

H 4.23925759 2.72066218 -1.06540994

H 4.05439079 -2.75667105 -1.52021386

H 2.43702504 -4.50935082 -0.32055604

H -2.65022906 -4.41482307 0.50235003

H -4.40379063 -2.68555491 -0.52867988

H 3.81743479 1.31772673 -3.38443651

H 6.05988842 1.35770581 -4.46870671

H 7.68503484 -1.04554733 -1.49772782

H 5.51279387 -1.17891683 -0.30058496

H -1.94544482 -4.26030442 2.48256069

H -1.70351978 -6.07659315 4.08581102

H 2.39389508 -6.31628586 3.42020001

H 2.32748764 -4.50417466 1.80085661

H -5.59059399 1.54148697 -0.50574104

H -7.69408959 1.32898804 -1.74199194

H -6.22000808 -1.85602462 -3.99126806

H -4.05363262 -1.78004580 -2.82565677

H -2.17254490 4.50940755 1.99505936

H -2.09393980 6.36455137 3.57329502

H 2.05599837 6.20564744 3.78698413

H 2.15390327 4.34077166 2.22655334

H 0.36052852 -8.40306900 4.13992848

H 1.38116129 -7.42506477 5.22949844

H -0.38628532 -7.40782481 5.41814290

H 8.65778778 1.24287754 -3.55214869

H 8.92338305 -0.50036389 -3.26962574

H 8.11529880 0.04171826 -4.76186912

H -0.95824930 7.61485493 5.13660412

H 0.80592204 7.48401056 5.30692939

H 0.11024526 8.49461255 4.01108443

H -8.59296330 -1.36566831 -3.81813469

H -9.07784854 0.20446162 -3.14144902

H -8.16216009 0.14297666 -4.66948621

O -0.20786038 -0.02615866 -2.40236475

H -0.28064964 -0.99082536 -2.49685624

H -1.12533215 0.28708748 -2.48701502

[CoP(H_2_O)]^+2^ M = 6

C 2.63693297 -1.10365582 -0.88350815

C 3.12636862 -2.40214178 -1.25287789

C 2.31590175 -3.33406706 -0.63503709

C 1.31561821 -2.60559402 0.07290591

N 1.48379290 -1.27411422 -0.11600244

C 3.17946515 0.17499828 -1.15817176

C 0.17337849 -3.16299977 0.82015666

C -1.11233949 -2.70450455 0.41600834

C -2.35716985 -3.41341878 0.37132228

C -3.26434457 -2.58965997 -0.25874534

C -2.57695711 -1.37343484 -0.58146325

N -1.28474536 -1.46114649 -0.17171555

C -3.18913754 -0.17732002 -1.15553658

C -2.64528556 1.10243316 -0.88667429

C -3.13815540 2.40073516 -1.25424774

C -2.32792070 3.33326394 -0.63768071

C -1.32458616 2.60486910 0.06725522

N -1.49023827 1.27476086 -0.12374439

C -0.18263624 3.16263567 0.81592022

C 1.10450329 2.70624717 0.41019559

C 2.35154188 3.41148372 0.37012073

C 3.25750679 2.58619503 -0.25911932

C 2.56782988 1.37197006 -0.58542082

N 1.27484820 1.46179671 -0.18027193

C -0.38499479 4.14104131 1.83364167

C -4.39719397 -0.35211816 -1.92496766

C 0.37542997 -4.14000285 1.83731313

C 4.38528789 0.34886876 -1.93124511

C -4.56400737 -1.47264665 -2.79664668

C -5.69193209 -1.62930450 -3.55464433

N -6.72852686 -0.73486213 -3.48962416

C -6.63251680 0.32577142 -2.63457492

C -5.51206775 0.53875541 -1.87549298

C 0.66396719 4.58900377 2.70327823

C 0.44512807 5.53831122 3.66304716

N -0.78889746 6.09061262 3.86076006

C -1.83739741 5.64151655 3.10704667

C -1.67279522 4.69465855 2.13396751

C 5.50065785 -0.54120462 -1.88382722

C 6.61986115 -0.32682168 -2.64480578

N 6.71723477 0.73902985 -3.49234410

C 5.67599427 1.62735718 -3.56260312

C 4.54889054 1.46891924 -2.80366808

C 1.66259735 -4.69590595 2.13825829

C 1.82587324 -5.64157781 3.11243510

N 0.77678017 -6.08757019 3.86772969

C -0.45639313 -5.53252973 3.66990309

C -0.67375914 -4.58432258 2.70895836

C -0.98269710 7.17718987 4.83160798

C -7.94987194 -0.97415699 -4.27128247

C 0.96883782 -7.17323361 4.83957173

C 7.89535264 0.92803796 -4.35068363

Co -0.00775160 0.00001450 -0.04095024

H 3.96113068 -2.61507532 -1.90289450

H 2.38872476 -4.41139094 -0.69780408

H -2.51973526 -4.43338631 0.68913406

H -4.30167661 -2.80533402 -0.47206393

H -3.97504865 2.61289271 -1.90170227

H -2.40258309 4.41055223 -0.69896410

H 2.51648819 4.43032245 0.69010387

H 4.29584315 2.79933319 -0.47018402

H -3.77024604 -2.20156555 -2.90506510

H -5.81658965 -2.45006472 -4.25000216

H -7.49744290 0.97484563 -2.57884336

H -5.52305084 1.36939947 -1.18306043

H 1.65094465 4.15144488 2.65054087

H 1.23257546 5.88277420 4.32165531

H -2.80355949 6.07758335 3.32891191

H -2.55382160 4.37045051 1.59732163

H 5.51468305 -1.37025130 -1.18949873

H 7.48387400 -0.97742597 -2.59393895

H 5.79713022 2.44642514 -4.26044041

H 3.75505365 2.19817070 -2.90929855

H 2.54424114 -4.37426336 1.60097594

H 2.79127642 -6.07924849 3.33454859

H -1.24392324 -5.87397650 4.33002756

H -1.65957272 -4.14413143 2.65599952

H -0.91826557 8.14484310 4.32588208

H -1.96292574 7.07342884 5.29946522

H -0.21094666 7.11515747 5.59939739

H -8.55801755 -1.74455263 -3.78850213

H -8.52136246 -0.04857094 -4.34003159

H -7.67688527 -1.30107112 -5.27646135

H 1.94948651 -7.07101540 5.30700107

H 0.19763767 -7.10879931 5.60776258

H 0.90229143 -8.14164659 4.33545615

H 8.22440135 1.96765046 -4.29316735

H 8.69927781 0.27827819 -4.00529685

H 7.64492068 0.67910064 -5.38574395

O 0.14704762 0.01016834 2.24562791

H 1.01884758 -0.41758599 2.30640786

H 0.32325069 0.94551432 2.44716452

[CoP(H_2_O)]^+1^ M = 1

C 2.59011861 -1.24954767 -0.89594688

C 3.15808986 -2.55416072 -1.06385238

C 2.30207443 -3.44366169 -0.45120002

C 1.18908054 -2.68677873 0.04110569

N 1.35705697 -1.37672919 -0.26287577

C 3.17644319 -0.00011734 -1.17147529

C -0.01838740 -3.18577249 0.68741044

C -1.27511575 -2.65732009 0.15580797

C -2.47873334 -3.36179792 -0.11308864

C -3.35309730 -2.45933072 -0.70050950

C -2.67680070 -1.20930528 -0.78811514

N -1.40185553 -1.36176856 -0.26169757

C -3.25404225 0.04862316 -1.15434323

C -2.64046613 1.29164256 -0.79737519

C -3.13303832 2.60856084 -1.01129465

C -2.24910562 3.48471825 -0.39335544

C -1.20102317 2.70161339 0.14929473

N -1.42397133 1.38115989 -0.11699651

C 0.02984275 3.20054535 0.77184419

C 1.26091700 2.66626638 0.22017311

C 2.46276137 3.38182682 -0.10306218

C 3.29933499 2.49834012 -0.74713777

C 2.61884671 1.23831483 -0.79511482

N 1.36613704 1.38153029 -0.20682072

C 0.00960913 4.17865686 1.77153379

C -4.52836119 0.03058061 -1.84083407

C 0.03597015 -4.15621353 1.70181947

C 4.50500658 0.02562337 -1.82120263

C -4.80544177 -0.91294255 -2.88263393

C -5.99896813 -0.92675963 -3.54721057

N -7.01031264 -0.04610496 -3.23102922

C -6.80511238 0.84677859 -2.20665896

C -5.61497120 0.91226341 -1.53710772

C 1.19480674 4.66815323 2.45086099

C 1.13737834 5.63517563 3.40571618

N -0.05042099 6.19428712 3.81752337

C -1.21308649 5.70605330 3.26621014

C -1.21524458 4.74119593 2.30791428

C 5.60902650 -0.67974413 -1.30908888

C 6.82847111 -0.63564963 -1.95585016

N 6.99042936 0.08692446 -3.08951297

C 5.95026949 0.78971061 -3.60507661

C 4.71482126 0.77760877 -2.99415661

C 1.27232461 -4.70104638 2.22381515

C 1.29197592 -5.66404781 3.18593249

N 0.14356822 -6.15617674 3.75630736

C -1.05270718 -5.60834238 3.36311530

C -1.13288683 -4.64897967 2.40044206

C -0.07065907 7.32838463 4.73937256

C -8.31050411 -0.14644689 -3.89760310

C 0.18874218 -7.28339878 4.68930880

C 8.29016264 0.10237322 -3.79694203

Co -0.02424810 0.00516291 -0.09627018

H 4.08020082 -2.79204882 -1.57536302

H 2.40496381 -4.51874621 -0.39389836

H -2.65294821 -4.41367452 0.06853799

H -4.37217140 -2.65425810 -1.00442978

H -4.01792632 2.88183828 -1.56688691

H -2.30674680 4.56530203 -0.36770884

H 2.63885779 4.43298952 0.07888352

H 4.29200278 2.70044179 -1.12591471

H -4.03823276 -1.61192756 -3.19284244

H -6.20370876 -1.60856617 -4.36369233

H -7.64701007 1.47939113 -1.95351240

H -5.53409503 1.61389432 -0.71719849

H 2.16461459 4.24077657 2.23319283

H 2.02621597 5.99986435 3.90802966

H -2.13101060 6.13101909 3.65606884

H -2.17377345 4.38342502 1.95525510

H 5.52444415 -1.25033209 -0.39321360

H 7.69973455 -1.16115428 -1.58757032

H 6.14783376 1.34173445 -4.51519243

H 3.90376061 1.34358411 -3.43518721

H 2.22556705 -4.33443116 1.86709852

H 2.21933100 -6.08069398 3.56169867

H -1.93149925 -5.97937704 3.87773732

H -2.10932655 -4.23769684 2.18342121

H -0.00076931 8.27849588 4.19723249

H -0.99908030 7.31385019 5.31453633

H 0.77044678 7.25076062 5.43185601

H -8.91469178 -0.94078558 -3.44632083

H -8.83851619 0.80384515 -3.80615865

H -8.16069139 -0.36635739 -4.95698113

H 1.11688580 -7.24338315 5.26314201

H -0.65436890 -7.21766207 5.37987545

H 0.13918719 -8.23635965 4.15154220

H 8.57215122 1.13630947 -3.99969762

H 9.04456560 -0.36709101 -3.16778459

H 8.19126368 -0.45071870 -4.73299540

O -0.12384359 -0.33212058 2.22361638

H -1.07825244 -0.15015310 2.25284246

H -0.07344590 -1.30567284 2.16531179

[CoP(H_2_O)]^+1^ M = 3

C 2.59051328 -1.24961223 -0.89545671

C 3.15850885 -2.55421216 -1.06319503

C 2.30252195 -3.44369303 -0.45047809

C 1.18934241 -2.68690465 0.04143532

N 1.35725438 -1.37679787 -0.26275825

C 3.17699047 -0.00019904 -1.17102728

C -0.01826926 -3.18616109 0.68705169

C -1.27500751 -2.65753624 0.15584899

C -2.47900589 -3.36170685 -0.11207952

C -3.35357184 -2.45910024 -0.69902604

C -2.67706162 -1.20931749 -0.78714471

N -1.40170737 -1.36199532 -0.26173306

C -3.25423160 0.04859641 -1.15352530

C -2.64100736 1.29165234 -0.79642655

C -3.13348902 2.60848706 -1.01049174

C -2.24905508 3.48470129 -0.39329666

C -1.20091847 2.70167785 0.14924861

N -1.42409428 1.38114465 -0.11636338

C 0.02994664 3.20071249 0.77158735

C 1.26108083 2.66628562 0.22037144

C 2.46303941 3.38168243 -0.10244794

C 3.29973338 2.49817956 -0.74641072

C 2.61922956 1.23824091 -0.79470325

N 1.36632738 1.38148295 -0.20673781

C 0.00965554 4.17917792 1.77105850

C -4.52859174 0.03042189 -1.84003407

C 0.03604419 -4.15691132 1.70136551

C 4.50541987 0.02561085 -1.82077395

C -4.80552558 -0.91311574 -2.88177361

C -5.99895718 -0.92686431 -3.54656910

N -7.01026084 -0.04616295 -3.23052420

C -6.80520967 0.84670695 -2.20624133

C -5.61514702 0.91216646 -1.53647891

C 1.19476093 4.66875705 2.45041403

C 1.13727782 5.63605432 3.40501309

N -0.05051752 6.19535815 3.81647062

C -1.21312242 5.70702504 3.26519444

C -1.21520573 4.74185456 2.30718541

C 5.60936811 -0.68062017 -1.30952004

C 6.82868485 -0.63646215 -1.95644622

N 6.99077509 0.08699661 -3.08956755

C 5.95074875 0.79068807 -3.60428538

C 4.71546055 0.77859749 -2.99312394

C 1.27235904 -4.70167869 2.22339419

C 1.29203312 -5.66489655 3.18533254

N 0.14364695 -6.15723320 3.75547592

C -1.05260178 -5.60937648 3.36238154

C -1.13276745 -4.64980192 2.39987996

C -0.07081085 7.32963090 4.73812966

C -8.31012365 -0.14619456 -3.89786335

C 0.18885276 -7.28451951 4.68843200

C 8.29031687 0.10248267 -3.79726743

Co -0.02393102 0.00519784 -0.09632254

H 4.08064557 -2.79216444 -1.57460891

H 2.40542338 -4.51878008 -0.39324664

H -2.65337849 -4.41353500 0.06965004

H -4.37289074 -2.65381714 -1.00226072

H -4.01830161 2.88180022 -1.56619459

H -2.30675046 4.56528559 -0.36768562

H 2.63919826 4.43282953 0.07952381

H 4.29252190 2.70027503 -1.12486239

H -4.03833239 -1.61222132 -3.19174315

H -6.20363546 -1.60866578 -4.36306631

H -7.64708738 1.47941361 -1.95326819

H -5.53432176 1.61386409 -0.71663047

H 2.16456513 4.24124203 2.23304198

H 2.02607507 6.00080418 3.90734875

H -2.13107623 6.13213886 3.65481563

H -2.17371273 4.38397385 1.95460187

H 5.52482822 -1.25184999 -0.39404451

H 7.69981502 -1.16258206 -1.58872376

H 6.14833852 1.34337435 -4.51399349

H 3.90456368 1.34527979 -3.43354692

H 2.22560928 -4.33487557 1.86692678

H 2.21940151 -6.08151635 3.56108612

H -1.93140498 -5.98050654 3.87690986

H -2.10920318 -4.23844620 2.18305176

H -0.00077818 8.27961190 4.19579216

H -0.99931679 7.31525854 5.31315382

H 0.77019692 7.25207045 5.43073430

H -8.91365430 -0.94214529 -3.44858231

H -8.83905587 0.80337108 -3.80439725

H -8.15970246 -0.36354328 -4.95769179

H 1.11693696 -7.24443226 5.26234875

H -0.65434246 -7.21891282 5.37890104

H 0.13945367 -8.23741671 4.15055359

H 8.57291955 1.13647928 -3.99889477

H 9.04459269 -0.36817523 -3.16884470

H 8.19090676 -0.44944962 -4.73395804

O -0.12670267 -0.33226731 2.22341359

H -1.08114985 -0.15018665 2.25094682

H -0.07627303 -1.30583708 2.16566514

[CoP(H_2_O)]^+1^ M = 5

C 2.62908253 -1.20162723 -0.81945987

C 3.15214909 -2.51276354 -1.04532539

C 2.31502135 -3.40376761 -0.39916096

C 1.24733684 -2.64277086 0.15634986

N 1.42677180 -1.31977458 -0.13761131

C 3.22024743 0.06141906 -1.14492994

C 0.05276998 -3.15780555 0.80403139

C -1.20872075 -2.63965635 0.33113096

C -2.43661797 -3.34638452 0.14958705

C -3.30222701 -2.48983877 -0.49935617

C -2.61063047 -1.25382307 -0.69695371

N -1.33546934 -1.37425366 -0.19289362

C -3.20036072 -0.02850885 -1.16696119

C -2.61994319 1.23852888 -0.84118514

C -3.14591442 2.54655182 -1.07370463

C -2.31096652 3.44394082 -0.43083952

C -1.24129829 2.69062364 0.12867195

N -1.41892015 1.36378221 -0.15500160

C -0.04526430 3.21514468 0.76632272

C 1.21764454 2.69143497 0.30111152

C 2.44049331 3.40232138 0.10298697

C 3.31191210 2.53822542 -0.52989511

C 2.62786706 1.29380783 -0.70121974

N 1.35205283 1.41884222 -0.19554746

C -0.13198979 4.22500713 1.75894716

C -4.44125788 -0.12062941 -1.89856397

C 0.13722828 -4.17363714 1.79995643

C 4.47918992 0.13004674 -1.84883188

C -4.65687484 -1.15529550 -2.86596243

C -5.81628535 -1.24509587 -3.58288731

N -6.85047897 -0.35860701 -3.39292583

C -6.70717566 0.62138091 -2.44576993

C -5.55280281 0.76521772 -1.72574293

C 1.00541590 4.70207404 2.50766639

C 0.89508965 5.67759505 3.45393848

N -0.31063233 6.25009555 3.77355173

C -1.43782828 5.76751870 3.15558145

C -1.38181611 4.78973243 2.20700889

C 5.57376723 -0.76743098 -1.62919654

C 6.74028952 -0.66887547 -2.33493091

N 6.92089902 0.28894961 -3.30129081

C 5.90363100 1.18263516 -3.53775561

C 4.73150444 1.13657306 -2.83625770

C 1.38151231 -4.75362383 2.22947538

C 1.43209081 -5.74574448 3.16688023

N 0.30535573 -6.21877726 3.78427191

C -0.89323957 -5.63074662 3.48396860

C -0.99869428 -4.64019114 2.54930375

C -0.38890428 7.37640267 4.70553352

C -8.10850698 -0.53351517 -4.12578083

C 0.37833776 -7.35857597 4.70641884

C 8.12801959 0.29483149 -4.13248723

Co 0.01081562 0.02233074 -0.06644484

H 4.02389586 -2.76888287 -1.62777959

H 2.39827869 -4.48198959 -0.38371991

H -2.62060148 -4.38113682 0.40113196

H -4.32406748 -2.69885541 -0.78275969

H -4.01860874 2.79614315 -1.65773478

H -2.39859168 4.52221540 -0.41832976

H 2.61755529 4.44321056 0.33532684

H 4.33272192 2.74650138 -0.81787055

H -3.86902401 -1.86754744 -3.07908176

H -5.97245898 -1.99928401 -4.34448988

H -7.56786116 1.26003067 -2.28878723

H -5.52951860 1.53066987 -0.96186450

H 1.98090609 4.25971863 2.36294189

H 1.75081463 6.03420460 4.01487266

H -2.37635016 6.20005689 3.48154433

H -2.31997440 4.42858929 1.80903015

H 5.52274464 -1.50825381 -0.84255633

H 7.58468633 -1.32204313 -2.15095228

H 6.08333283 1.90813445 -4.32151719

H 3.96184738 1.85891098 -3.08024430

H 2.32097467 -4.40403339 1.82522865

H 2.36653900 -6.19884211 3.47512861

H -1.75007764 -5.98830019 4.04196098

H -1.97083636 -4.18791527 2.41488499

H -0.32102497 8.32820604 4.16780458

H -1.33633964 7.33556552 5.24669526

H 0.43048198 7.31035798 5.42366159

H -8.69242479 -1.35048958 -3.69027028

H -8.68609875 0.38999410 -4.07690074

H -7.89211873 -0.76180891 -5.17186429

H 1.32459445 -7.32538251 5.24906916

H -0.44415971 -7.29831623 5.42054791

H 0.30958969 -8.29911593 4.15156399

H 8.35200199 1.31797326 -4.43932516

H 8.96773429 -0.09371958 -3.55370805

H 7.98351141 -0.32753206 -5.02171484

O -0.25217404 -0.02023053 2.27001704

H -1.11964871 0.41024952 2.17596257

H -0.46503436 -0.96652690 2.35118075

[CoP(H_2_O)]^0^ M = 2

C 2.67253822 -1.16414200 -0.83337796

C 3.21695284 -2.45745659 -1.07169463

C 2.38617011 -3.37633604 -0.43600774

C 1.32104603 -2.64160741 0.13751922

N 1.47966296 -1.31541375 -0.13020458

C 3.22263676 0.12077494 -1.14986718

C 0.13175434 -3.15935290 0.83136749

C -1.14320170 -2.71593022 0.26743641

C -2.32138480 -3.46825411 0.02109411

C -3.21890591 -2.61734635 -0.61136317

C -2.58069775 -1.35134476 -0.74359159

N -1.30738914 -1.44263553 -0.20168407

C -3.19688811 -0.12445594 -1.15940760

C -2.64166921 1.14655536 -0.80978040

C -3.17864744 2.44394730 -1.04804849

C -2.35453201 3.35560624 -0.40046995

C -1.29420509 2.61614418 0.18195935

N -1.45370893 1.28929811 -0.08929683

C -0.11037247 3.15194800 0.86158692

C 1.16688079 2.68502276 0.33514021

C 2.35030151 3.43876997 0.09735426

C 3.24377654 2.59792528 -0.54756978

C 2.60481457 1.33218301 -0.69733006

N 1.33225321 1.41735155 -0.14677190

C -0.21786781 4.09321072 1.88457597

C -4.44942918 -0.20967941 -1.87792919

C 0.23083750 -4.06471646 1.88712305

C 4.47294303 0.22840946 -1.86973360

C -4.66661442 -1.20000687 -2.89161530

C -5.83507672 -1.27280567 -3.59461628

N -6.88198525 -0.40976808 -3.34707266

C -6.73765901 0.52292301 -2.34648017

C -5.57391914 0.64680493 -1.64094411

C 0.91679812 4.61352959 2.63133776

C 0.77904527 5.55088266 3.60506474

N -0.44826402 6.05786970 3.97600054

C -1.56653014 5.53945754 3.35668241

C -1.48862415 4.59810512 2.38038929

C 5.60040338 -0.63141959 -1.65663249

C 6.76199482 -0.48793632 -2.36130648

N 6.90873758 0.48154335 -3.32672137

C 5.85169928 1.33411239 -3.56502697

C 4.68463064 1.24125156 -2.86158467

C 1.49957674 -4.54617302 2.41015599

C 1.57168232 -5.44625402 3.42467612

N 0.45002452 -5.94487869 4.05349631

C -0.77719505 -5.47068736 3.64170290

C -0.91132604 -4.57492493 2.62936077

C -0.55220580 7.18399441 4.89931837

C -8.15259761 -0.57095607 -4.05526949

C 0.55121633 -7.03128307 5.02413660

C 8.11218861 0.54047554 -4.15690445

Co 0.01661516 -0.01297080 -0.03142454

H 4.10202204 -2.68971269 -1.64545280

H 2.49828871 -4.45284357 -0.40520830

H -2.46475666 -4.51547658 0.25264299

H -4.22928154 -2.85414597 -0.91589269

H -4.05362822 2.68055414 -1.63540164

H -2.45905283 4.43278363 -0.37480078

H 2.49480876 4.48416404 0.33509410

H 4.25226872 2.84024712 -0.85354595

H -3.87030000 -1.88782179 -3.14949087

H -5.99234201 -1.98958799 -4.39153053

H -7.60564824 1.13832618 -2.14350774

H -5.54297839 1.37703601 -0.84299172

H 1.91054344 4.22876115 2.44306601

H 1.62923318 5.93474745 4.15803037

H -2.51670570 5.92149657 3.71321847

H -2.41647024 4.21339440 1.97642661

H 5.57449132 -1.37709770 -0.87297725

H 7.63124354 -1.10825087 -2.17856761

H 6.00259877 2.06652433 -4.34857591

H 3.88701398 1.93424335 -3.10101333

H 2.42779689 -4.17690985 1.99236361

H 2.51991074 -5.81087086 3.80379452

H -1.63192610 -5.84727151 4.19263180

H -1.90708867 -4.21734921 2.40112649

H -0.51157045 8.14189646 4.36673623

H -1.49624031 7.12234759 5.44602975

H 0.26955379 7.14369767 5.61819670

H -8.74554221 -1.37658223 -3.60843315

H -8.71665576 0.36145665 -4.00377112

H -7.95838924 -0.80739203 -5.10410438

H 1.49157829 -6.94342568 5.57334849

H -0.27489433 -6.96318778 5.73590862

H 0.51672468 -8.01064601 4.53184201

H 8.32026719 1.57860634 -4.42403178

H 8.96003419 0.14473370 -3.59469337

H 7.98156232 -0.04795577 -5.07195346

O -0.17458247 -0.37330729 2.34020844

H -1.13481448 -0.25864665 2.24399092

H -0.04388696 -1.33525544 2.22135718

[CoP(H_2_O)]^0^ M = 4

C 2.66741730 -1.20119869 -0.87369762

C 3.19540214 -2.50192345 -1.11003977

C 2.34758414 -3.40952300 -0.48125257

C 1.28804200 -2.66042055 0.08534806

N 1.46742749 -1.33672842 -0.17902051

C 3.23776374 0.07631884 -1.18283762

C 0.08540809 -3.16207476 0.76729921

C -1.17831710 -2.69776577 0.19435870

C -2.36539679 -3.43140657 -0.06144780

C -3.24791030 -2.56419167 -0.69349851

C -2.59096193 -1.30734798 -0.81696807

N -1.32112407 -1.41979348 -0.27040992

C -3.18675830 -0.06906549 -1.22988748

C -2.61182848 1.19189173 -0.87437130

C -3.12379251 2.49815110 -1.11546076

C -2.28659395 3.39533636 -0.46327340

C -1.24473067 2.63732660 0.12642525

N -1.42575170 1.31302658 -0.14638803

C -0.05826657 3.15200343 0.81852057

C 1.21534535 2.66640422 0.30218000

C 2.41473536 3.40059359 0.07896365

C 3.29771431 2.54834970 -0.56467681

C 2.63750001 1.29471018 -0.72686459

N 1.36267981 1.39902697 -0.18551004

C -0.16102259 4.09176483 1.84375902

C -4.44138308 -0.13056064 -1.94712390

C 0.16115457 -4.07109959 1.82176042

C 4.49003051 0.16918035 -1.90175427

C -4.67812140 -1.11362868 -2.96275159

C -5.85144377 -1.16558462 -3.66035428

N -6.88244499 -0.28809718 -3.40471028

C -6.71870832 0.64027059 -2.40485779

C -5.54967165 0.74403150 -1.70388923

C 0.97325972 4.59478250 2.60244983

C 0.83829448 5.53061655 3.57836319

N -0.38543311 6.05158238 3.94006558

C -1.50466918 5.54993352 3.30866607

C -1.42953735 4.61157098 2.32934463

C 5.60560324 -0.70842768 -1.69474960

C 6.76661538 -0.58121194 -2.40262747

N 6.92609094 0.38986066 -3.36620222

C 5.88049570 1.26060743 -3.59743954

C 4.71516891 1.18324247 -2.89002422

C 1.41686188 -4.57608865 2.35419153

C 1.46436624 -5.48017968 3.36671111

N 0.32911504 -5.96035808 3.98508408

C -0.88635637 -5.46306916 3.56460621

C -0.99594452 -4.56284508 2.55353044

C -0.48448079 7.17414712 4.86845759

C -8.15854093 -0.42533990 -4.11044247

C 0.40219416 -7.05147117 4.95281040

C 8.12177136 0.42573421 -4.20695689

Co 0.02279522 -0.01166942 -0.08547406

H 4.08056167 -2.74603834 -1.67872460

H 2.44353967 -4.48763601 -0.45171507

H -2.52575684 -4.47731316 0.16480911

H -4.26054596 -2.78491984 -1.00228258

H -3.99121942 2.75031683 -1.70753942

H -2.37326922 4.47408598 -0.43682708

H 2.57528824 4.44196228 0.32393167

H 4.31217258 2.77464214 -0.86336636

H -3.89434832 -1.81349110 -3.22636099

H -6.02413888 -1.87777646 -4.45816347

H -7.57544457 1.26951944 -2.19694196

H -5.50351483 1.47227361 -0.90494186

H 1.96372346 4.19773562 2.42249494

H 1.68817467 5.90174896 4.14044874

H -2.45318000 5.94273840 3.65774673

H -2.35830405 4.24088865 1.91468785

H 5.57025962 -1.45609340 -0.91327042

H 7.62672470 -1.21572349 -2.22503038

H 6.03993379 1.99339234 -4.37898682

H 3.92758917 1.88964214 -3.12366604

H 2.35503147 -4.22192618 1.94553182

H 2.40276301 -5.86271316 3.75268526

H -1.75218425 -5.82544970 4.10763550

H -1.98322394 -4.18720269 2.31778453

H -0.43247078 8.13405630 4.34059204

H -1.43204460 7.11836397 5.40954761

H 0.33286706 7.12281626 5.59162391

H -8.75854913 -1.22816685 -3.66886640

H -8.71023642 0.51343785 -4.04616298

H -7.97079885 -0.65295364 -5.16226852

H 1.34082895 -6.98447951 5.50799647

H -0.42665038 -6.96870502 5.65982433

H 0.35045444 -8.02854147 4.45742178

H 8.34598840 1.45950397 -4.47857447

H 8.96808824 0.01640432 -3.65180551

H 7.97400550 -0.16189219 -5.12028044

O -0.19359948 -0.37368951 2.28453133

H -1.14798065 -0.22374407 2.17814287

H -0.09579445 -1.33915542 2.16344361

[CoP(H_2_O)]^0^_2 M = 4

C 2.64311127 -1.20036908 -0.88937079

C 3.16421481 -2.50112608 -1.13623588

C 2.31641562 -3.40966727 -0.50749941

C 1.26540573 -2.66028612 0.07128125

N 1.44708903 -1.33466158 -0.18765062

C 3.21621736 0.07605551 -1.19944635

C 0.06968457 -3.16170009 0.76635431

C -1.19900450 -2.69494448 0.20811497

C -2.39058294 -3.42661974 -0.03886155

C -3.27376360 -2.55924017 -0.66866634

C -2.61418183 -1.30375943 -0.79767030

N -1.34241568 -1.41777903 -0.25628239

C -3.20975332 -0.06671754 -1.21176158

C -2.63624070 1.19440791 -0.85840645

C -3.15570432 2.50044524 -1.09029328

C -2.31802100 3.39738291 -0.44059616

C -1.26598830 2.64046555 0.13612221

N -1.44467301 1.31753153 -0.13972617

C -0.07263607 3.15681307 0.81304472

C 1.19640299 2.67110465 0.28299708

C 2.39013825 3.40708963 0.04659708

C 3.27295890 2.55254324 -0.59577844

C 2.61605473 1.29683695 -0.74694646

N 1.34332037 1.40067017 -0.19944612

C -0.16222174 4.09742566 1.83852915

C -4.45994813 -0.13149186 -1.93714216

C 0.15770410 -4.07370547 1.81764054

C 4.47490760 0.16312740 -1.90744762

C -4.68437716 -1.11443563 -2.95692446

C -5.84724965 -1.16627000 -3.67028187

N -6.88363514 -0.28694305 -3.42821399

C -6.73355751 0.63790505 -2.41962335

C -5.57419534 0.74021810 -1.70425154

C 0.98344434 4.59530995 2.58383859

C 0.86550614 5.53295819 3.55966253

N -0.35162139 6.06212989 3.93415334

C -1.48044794 5.56542117 3.31660999

C -1.42238127 4.62414252 2.33869308

C 5.58446746 -0.71549872 -1.68193349

C 6.75847733 -0.58758770 -2.36975034

N 6.93325955 0.38159201 -3.32899066

C 5.89408143 1.24991717 -3.58072469

C 4.71512248 1.17356650 -2.89449029

C 1.42097599 -4.57523905 2.33469209

C 1.48365220 -5.48068614 3.34496606

N 0.35675317 -5.96685307 3.97437997

C -0.86459983 -5.47409555 3.56820077

C -0.98934990 -4.57150457 2.56045888

C -0.43178495 7.18924389 4.85855602

C -8.15026564 -0.42670487 -4.14589686

C 0.44647014 -7.05837111 4.94056796

C 8.15508391 0.43214479 -4.13430131

Co 0.00589986 -0.00905783 -0.08528625

H 4.04570905 -2.74487745 -1.71074587

H 2.41026850 -4.48807646 -0.48298017

H -2.55088056 -4.47204188 0.18973996

H -4.28791575 -2.77799544 -0.97438424

H -4.02760403 2.75176901 -1.67612414

H -2.40652689 4.47588394 -0.41091498

H 2.55001255 4.45023978 0.28426886

H 4.28565078 2.78023096 -0.89878251

H -3.89627505 -1.81310734 -3.21100834

H -6.00836747 -1.87651779 -4.47228719

H -7.59395778 1.26518085 -2.22041258

H -5.53836723 1.46577618 -0.90216065

H 1.96945142 4.19289024 2.39212843

H 1.72397469 5.89981471 4.11132883

H -2.42254814 5.96427986 3.67622862

H -2.35825386 4.25666338 1.93710744

H 5.53590620 -1.46339848 -0.90154177

H 7.61526038 -1.22177512 -2.17647572

H 6.06778068 1.98165095 -4.36014081

H 3.93184687 1.87923172 -3.14386450

H 2.35296108 -4.21717006 1.91568260

H 2.42786312 -5.85989045 3.71967917

H -1.72288591 -5.84159955 4.11977201

H -1.98072328 -4.19884609 2.33711677

H -0.36999464 8.14664554 4.32708524

H -1.37727579 7.14732547 5.40471507

H 0.38850481 7.13060718 5.57783709

H -8.75996987 -1.22480851 -3.70739055

H -8.70079937 0.51401578 -4.09581285

H -7.95212753 -0.66244206 -5.19431825

H 1.38757850 -6.98268499 5.49020456

H -0.37902451 -6.98458490 5.65248698

H 0.40206778 -8.03511591 4.44395635

H 8.38409303 1.47010783 -4.38383042

H 8.98469108 0.01633080 -3.55992246

H 8.03271458 -0.14324794 -5.05839398

O -0.19125561 -0.38973642 2.29324671

H -1.15232049 -0.28944979 2.19085204

H -0.04575195 -1.34817964 2.16314553

[CoP(H_2_O)]^0^ M = 8

C 2.78863197 -0.95121493 -0.62516495

C 3.35721152 -2.17744616 -1.08432651

C 2.55689203 -3.20487284 -0.60846146

C 1.47808741 -2.59764136 0.09290542

N 1.61590746 -1.23652870 0.06117126

C 3.30372588 0.37393948 -0.83527569

C 0.31461370 -3.28799417 0.65487645

C -1.00234712 -2.84491303 0.26528290

C -2.15360639 -3.65199509 0.01382192

C -3.13491811 -2.82519546 -0.50470406

C -2.59643312 -1.49553467 -0.54344497

N -1.29051193 -1.53464322 -0.09100476

C -3.30405107 -0.32307012 -0.94244963

C -2.81318503 1.00215885 -0.76321608

C -3.48411976 2.21798978 -1.11700090

C -2.70025075 3.26796684 -0.65455023

C -1.54440111 2.69835919 -0.05002580

N -1.61772321 1.32528047 -0.14465920

C -0.39887546 3.38036220 0.53629531

C 0.93943206 2.91549381 0.19855743

C 2.11155579 3.71929369 0.04173754

C 3.12663771 2.88518638 -0.38543619

C 2.57782652 1.57025473 -0.49766620

N 1.24344192 1.61881216 -0.14069022

C -0.59323322 4.48702652 1.41254391

C -4.66327166 -0.49483253 -1.52064247

C 0.51167233 -4.41374456 1.50822782

C 4.63090894 0.51798376 -1.39100482

C -4.89092200 -1.21071963 -2.73451107

C -6.14298390 -1.38862211 -3.25460398

N -7.27467799 -0.82953536 -2.64011144

C -7.08748519 -0.16646210 -1.41738041

C -5.84014132 0.00978078 -0.88685237

C 0.46737344 5.12052037 2.16213310

C 0.25287012 6.20115057 2.96509420

N -0.99821302 6.74197069 3.14364028

C -2.06105192 6.10300067 2.55073002

C -1.89635620 5.01753549 1.74313852

C 5.73086749 -0.32663533 -1.02692290

C 6.97755541 -0.16236358 -1.56092900

N 7.24176969 0.81316765 -2.49482155

C 6.21155052 1.63643285 -2.89078653

C 4.95833389 1.52357009 -2.35977260

C 1.81131056 -4.94648662 1.83941862

C 1.96760455 -6.04827617 2.62712318

N 0.89545027 -6.69802161 3.19697197

C -0.35668840 -6.16031777 3.00693596

C -0.56211683 -5.06737413 2.21899696

C -1.18303931 7.98729520 3.88805258

C -8.59829907 -1.31223952 -3.00035087

C 1.07553378 -7.95874243 3.91526046

C 8.55328191 0.90513336 -3.13861352

Co -0.06089001 0.06665770 0.19084841

H 4.22837615 -2.28351688 -1.71559613

H 2.67558164 -4.26479221 -0.79340511

H -2.20945994 -4.72696603 0.12250286

H -4.12431166 -3.11011906 -0.83545083

H -4.42564803 2.28504259 -1.64501012

H -2.88868790 4.32573823 -0.78809802

H 2.17347243 4.79123529 0.16560360

H 4.14983511 3.17199592 -0.58198766

H -4.04697243 -1.63854083 -3.26879814

H -6.33175452 -1.93058263 -4.17405216

H -7.98903460 0.21416416 -0.95124672

H -5.75049168 0.56054746 0.04521336

H 1.47024315 4.71915226 2.13814329

H 1.05423769 6.67240728 3.52214775

H -3.04061461 6.50600493 2.78036632

H -2.79036420 4.54298623 1.36411501

H 5.59991556 -1.08744301 -0.26751006

H 7.82226631 -0.77014816 -1.25990949

H 6.45467588 2.36316903 -3.65640389

H 4.18821288 2.19016247 -2.72818607

H 2.70876868 -4.46797723 1.47181434

H 2.94360153 -6.45848321 2.85853581

H -1.16479235 -6.64723555 3.54031283

H -1.56675650 -4.67035935 2.17157089

H -1.08383247 8.85461088 3.22603618

H -2.17591598 7.99541270 4.34230044

H -0.43326422 8.05362433 4.67938030

H -8.84369497 -2.26182977 -2.50008040

H -9.34941794 -0.56787319 -2.72079462

H -8.65098182 -1.46562797 -4.08210789

H 2.04559985 -7.95719572 4.41695189

H 0.29084123 -8.06476519 4.66721317

H 1.02926081 -8.80987613 3.22655124

H 8.79467300 1.95295725 -3.32949792

H 9.31120996 0.48463988 -2.47560223

H 8.55841181 0.35480891 -4.08585161

O 0.22485813 -0.40756331 2.38445118

H 1.06108836 -0.84953603 2.13272510

H -0.40214808 -1.14313845 2.50356966

[CoP(H_2_O)]^–1^ M = 1

C 2.89289372 -1.32418401 -0.18097539

C 3.51082966 -2.52489713 0.20311632

C 2.51231090 -3.34376432 0.77748951

C 1.30900435 -2.62189949 0.72959199

N 1.55472886 -1.39470269 0.14578010

C 3.46566758 -0.09888875 -0.76693478

C -0.05665344 -3.02720949 1.10714476

C -1.12105472 -2.60215419 0.19197886

C -2.25787142 -3.31853307 -0.27223248

C -2.95963349 -2.47039111 -1.11931300

C -2.25028420 -1.23464921 -1.16559680

N -1.12037580 -1.35721637 -0.37273239

C -2.71888337 0.01082659 -1.70632046

C -2.18846307 1.26748052 -1.25057150

C -2.59593033 2.58790611 -1.57793512

C -1.85154124 3.46227365 -0.78456705

C -0.96099115 2.67758817 -0.01940500

N -1.14975909 1.35575479 -0.31961541

C 0.09697578 3.12655623 0.89896099

C 1.43960826 2.59976648 0.61734000

C 2.69072619 3.23592570 0.68262386

C 3.64245119 2.33162343 0.16010136

C 2.95113465 1.16804275 -0.21150958

N 1.61147393 1.33870650 0.07733364

C -0.18372011 4.01725061 1.92579329

C -3.80931764 -0.03536127 -2.65111666

C -0.33476782 -3.77313765 2.25326608

C 4.43160463 -0.13306680 -1.75982850

C -3.88460922 -1.05255545 -3.65985175

C -4.90555072 -1.10155729 -4.56539594

N -5.93403521 -0.18486160 -4.54430751

C -5.92756769 0.78176211 -3.56661831

C -4.91026089 0.88320172 -2.66003831

C 0.79734867 4.45088905 2.91601307

C 0.50014366 5.33825186 3.89758768

N -0.76050931 5.88720732 4.04968170

C -1.74886517 5.45946591 3.17891643

C -1.51071936 4.56764591 2.18561338

C 4.94865636 -1.36560855 -2.35697210

C 5.94795530 -1.37287464 -3.27098512

N 6.57237869 -0.21147331 -3.70759938

C 6.01213506 0.99059531 -3.29546276

C 5.01364788 1.05696112 -2.38244827

C 0.68422339 -4.18864445 3.20790934

C 0.40071115 -4.95627129 4.29136985

N -0.87976180 -5.37676873 4.58928756

C -1.89936535 -4.93387676 3.77023984

C -1.67414410 -4.16248204 2.67600860

C -0.99563623 6.99535238 4.96532322

C -7.07330284 -0.32336781 -5.45376822

C -1.12705372 -6.36550462 5.63330980

C 7.51472262 -0.24876616 -4.81607872

Co 0.23537378 -0.01227926 -0.09987901

H 4.56386563 -2.75920061 0.10383207

H 2.63423022 -4.35586037 1.14361036

H -2.50655086 -4.34184578 -0.02387666

H -3.89577928 -2.68501828 -1.61626318

H -3.33476579 2.87111776 -2.31336310

H -1.91098178 4.54345430 -0.76977714

H 2.87422401 4.25061363 1.01430702

H 4.71139637 2.48853907 0.07735567

H -3.09006889 -1.78403020 -3.74399945

H -4.94954551 -1.84278739 -5.35407132

H -6.78584749 1.44246076 -3.54978794

H -4.99203500 1.64230199 -1.89356612

H 1.79269112 4.02566793 2.89994398

H 1.23274069 5.64810704 4.63556349

H -2.73668911 5.86914149 3.36132563

H -2.35141508 4.25087509 1.58061726

H 4.51231869 -2.31580032 -2.07665708

H 6.31774431 -2.29198756 -3.71350648

H 6.43184222 1.87885515 -3.75631723

H 4.63055649 2.03513039 -2.12025671

H 1.70699192 -3.86260442 3.07189356

H 1.16737237 -5.26632864 4.99336097

H -2.89967510 -5.22447694 4.07253694

H -2.53841514 -3.81270193 2.12698828

H -0.81403746 7.96672510 4.48611102

H -2.03014958 6.96680833 5.31801519

H -0.33363246 6.90205339 5.83033293

H -7.82987313 -0.99094139 -5.02707786

H -7.51753676 0.65761363 -5.63157195

H -6.72982938 -0.73276710 -6.40591134

H -0.39607986 -6.24008763 6.43595086

H -2.12716829 -6.21685508 6.04804128

H -1.05389594 -7.38847564 5.24365887

H 8.18191934 0.61586440 -4.76035466

H 8.12290019 -1.15494244 -4.74989106

H 7.00708317 -0.23794442 -5.79132014

O -1.15159754 -0.02174966 2.45884068

H -1.37076856 0.44608560 1.62859859

H -1.03614249 -0.94251390 2.16457184

[CoP(H_2_O)]^–1^ M = 3

C 2.89286999 -1.32411127 -0.18104801

C 3.51091127 -2.52480374 0.20296354

C 2.51243697 -3.34383291 0.77723308

C 1.30909933 -2.62207058 0.72931696

N 1.55469395 -1.39478553 0.14568190

C 3.46555886 -0.09885504 -0.76719897

C -0.05655188 -3.02705954 1.10717121

C -1.12106975 -2.60222299 0.19181444

C -2.25792522 -3.31856431 -0.27220154

C -2.96004593 -2.47027430 -1.11901697

C -2.25078049 -1.23465497 -1.16514334

N -1.12057205 -1.35733540 -0.37262401

C -2.71894593 0.01090797 -1.70610786

C -2.18920567 1.26764575 -1.25010294

C -2.59668113 2.58799439 -1.57734279

C -1.85163809 3.46228457 -0.78439173

C -0.96106286 2.67762951 -0.01935489

N -1.15020906 1.35580681 -0.31910736

C 0.09699219 3.12644040 0.89911768

C 1.43960538 2.60001623 0.61686791

C 2.69069147 3.23621109 0.68191452

C 3.64243506 2.33181409 0.15950638

C 2.95113889 1.16816939 -0.21184847

N 1.61148086 1.33882706 0.07716866

C -0.18362018 4.01658436 1.92634515

C -3.80904410 -0.03543645 -2.65144484

C -0.33468163 -3.77231348 2.25364293

C 4.43128595 -0.13316634 -1.76029908

C -3.88385741 -1.05273168 -3.65991893

C -4.90441670 -1.10176247 -4.56597560

N -5.93274783 -0.18502770 -4.54538888

C -5.92674556 0.78163417 -3.56793389

C -4.90980166 0.88316017 -2.66086364

C 0.79763158 4.45033816 2.91634796

C 0.50034285 5.33718606 3.89836491

N -0.76050744 5.88549572 4.05109302

C -1.74890354 5.45793556 3.18028398

C -1.51070377 4.56662585 2.18653243

C 4.94821901 -1.36576397 -2.35741056

C 5.94734766 -1.37312746 -3.27160830

N 6.57169643 -0.21179257 -3.70847276

C 6.01150736 0.99034041 -3.29638599

C 5.01321375 1.05680104 -2.38316698

C 0.68433189 -4.18758731 3.20842802

C 0.40079733 -4.95478185 4.29215238

N -0.87972377 -5.37512639 4.59028956

C -1.89933069 -4.93243649 3.77107425

C -1.67409767 -4.16148025 2.67657254

C -0.99587966 6.99306896 4.96739809

C -7.07150485 -0.32332905 -5.45562442

C -1.12701758 -6.36353635 5.63456865

C 7.51378547 -0.24918528 -4.81716421

Co 0.23542029 -0.01214153 -0.10020385

H 4.56397478 -2.75897024 0.10365341

H 2.63443762 -4.35585979 1.14353750

H -2.50660889 -4.34183570 -0.02363284

H -3.89645943 -2.68468384 -1.61557887

H -3.33557533 2.87143278 -2.31262907

H -1.91133429 4.54346386 -0.76931282

H 2.87415845 4.25099455 1.01334603

H 4.71134415 2.48883178 0.07649535

H -3.08946882 -1.78444709 -3.74331227

H -4.94816447 -1.84311585 -5.35453947

H -6.78496423 1.44241751 -3.55160130

H -4.99179293 1.64239651 -1.89455011

H 1.79319029 4.02563031 2.89975599

H 1.23308807 5.64714172 4.63615315

H -2.73678884 5.86734906 3.36295614

H -2.35138147 4.25001517 1.58141182

H 4.51193884 -2.31592360 -2.07689608

H 6.31707376 -2.29229161 -3.71407763

H 6.43107912 1.87854663 -3.75746564

H 4.63016155 2.03499772 -2.12101598

H 1.70713729 -3.86174582 3.07212722

H 1.16742631 -5.26462679 4.99427614

H -2.89963730 -5.22287378 4.07354778

H -2.53831458 -3.81189837 2.12733295

H -0.81532850 7.96480878 4.48853485

H -2.03013812 6.96355356 5.32075754

H -0.33323480 6.89982183 5.83191415

H -7.82850351 -0.99050956 -5.02913571

H -7.51524610 0.65776931 -5.63395487

H -6.72753174 -0.73311957 -6.40740138

H -0.39606624 -6.23792203 6.43721013

H -2.12714197 -6.21480751 6.04926438

H -1.05385329 -7.38664021 5.24524269

H 8.18108783 0.61537760 -4.76159791

H 8.12188764 -1.15542085 -4.75110605

H 7.00593133 -0.23830808 -5.79229461

O -1.15195273 -0.02139782 2.45808176

H -1.37212139 0.44688196 1.62831832

H -1.03705575 -0.94207095 2.16329473
