# Supplementary material for: Self-Assembled Liposomes Enhance Electron Transfer for Efficient Photocatalytic CO2 Reduction
Source: J Am Chem Soc. 2022 May 20;144(21):9399–412. doi: 10.1021/jacs.2c01725 (PMC9164230; doi:10.1021/jacs.2c01725)
Supplement: Supplementary file 2 — ja2c01725_si_002.zip [file ja2c01725_si_002.zip › Coordinates/Others/Coord_Others.docx]

CO2

O 0.00000 0.00000 1.16973

O 0.00000 0.00000 -1.16973

C 0.00000 0.00000 0.00000

CO

O 0.00000 0.00000 0.56862

C 0.00000 0.00000 -0.56862

H2O

O 0.00000 0.11854 0.0000

H 0.76860 -0.47414 0.0000

H -0.76860 -0.47414 0.0000

[Ru(bpy)_3_]^1+^

C -3.11710 -2.99220 -2.31114

C -3.47755 -2.26842 -1.17849

C -2.53267 -1.44918 -0.54664

N -1.26084 -1.34154 -1.03509

C -0.91644 -2.05385 -2.12728

C -1.80798 -2.88695 -2.79261

C -2.80415 -0.66056 0.66528

C 2.54630 -1.32059 -0.67397

N 1.70326 -0.34594 -1.13102

C 2.04778 0.35420 -2.23161

C 3.22975 0.12670 -2.92592

C 4.10053 -0.86799 -2.46715

C 3.75211 -1.59535 -1.33374

C -4.03409 -0.65129 1.33668

C -4.18718 0.11657 2.48690

C -3.09994 0.86641 2.94852

C -1.90392 0.81643 2.24257

N -1.74757 0.07298 1.12795

Ru -0.01018 -0.03435 -0.00238

C 2.24632 3.59313 2.35101

C 2.45062 2.26404 2.80497

C 1.81118 1.23793 2.12799

N 1.00733 1.43460 1.06254

C 0.80423 2.73000 0.59069

C 1.43476 3.81682 1.25894

C 2.07544 -2.04506 0.51538

C -0.05480 2.85699 -0.54525

N -0.60418 1.67261 -1.03299

C -1.43963 1.71851 -2.09128

C -1.77961 2.89009 -2.74856

C -1.21660 4.10619 -2.28101

C -0.36769 4.08392 -1.19471

C 2.78285 -3.08647 1.13124

C 2.23963 -3.73027 2.23861

C 0.98748 -3.32237 2.71121

C 0.33685 -2.27851 2.06462

N 0.86182 -1.64129 0.99809

H -3.84335 -3.62782 -2.80773

H -4.48630 -2.34182 -0.79014

H -1.47911 -3.43505 -3.66905

H 1.34225 1.11324 -2.54686

H -4.86499 -1.23935 0.96572

H -5.13624 0.12942 3.01344

H -3.17218 1.48312 3.83804

H -1.03338 1.37834 2.55898

H 2.72198 4.42804 2.85812

H 3.08381 2.04087 3.65690

H 1.27100 4.82977 0.90702

H -1.83738 0.76300 -2.41736

H -2.45608 2.86085 -3.59594

H -1.44938 5.04657 -2.77289

H 0.06921 5.00830 -0.83232

H 0.52057 -3.79816 3.56699

H -0.62933 -1.92216 2.40237

H 1.92895 0.20628 2.44267

H 0.10485 -1.93296 -2.46899

H 2.78126 -4.53683 2.72254

H 3.45926 0.72181 -3.80343

H 5.03268 -1.07339 -2.98391

H 4.41293 -2.37124 -0.96594

H 3.74915 -3.39261 0.74863

[Ru(bpy)_3_]^2+^

C 3.90731 1.79889 2.29234

C 3.32642 2.43560 1.19835

C 2.19220 1.88047 0.59837

N 1.64729 0.72035 1.06625

C 2.21473 0.10739 2.12437

C 3.34083 0.61402 2.76546

C 1.50078 2.47553 -0.56005

C 0.54891 -2.82839 0.57070

N -0.18948 -1.78557 1.04923

C -0.96932 -1.97200 2.13117

C -1.06752 -3.19845 2.77988

C -0.32483 -4.27559 2.29314

C 0.49384 -4.08530 1.18174

C 1.87731 3.68129 -1.16107

C 1.16201 4.15364 -2.25951

C 0.08371 3.40680 -2.73793

C -0.24296 2.21635 -2.09670

N 0.44128 1.75665 -1.03214

Ru -0.00377 0.00814 0.00457

C -4.14663 -1.07525 -2.32336

C -2.94745 -1.59187 -2.81699

C -1.76172 -1.27150 -2.16409

N -1.72333 -0.47951 -1.07441

C -2.88940 0.03665 -0.58928

C -4.11376 -0.25421 -1.19828

C 1.39255 -2.51394 -0.59839

C -2.74871 0.91089 0.58938

N -1.47494 1.05179 1.05878

C -1.25337 1.85165 2.12110

C -2.27743 2.52647 2.77724

C -3.58471 2.38129 2.30984

C -3.81892 1.57049 1.20188

C 2.25183 -3.43111 -1.21328

C 3.00142 -3.03921 -2.32025

C 2.87578 -1.73183 -2.79268

C 2.01024 -0.86440 -2.13480

N 1.28710 -1.23539 -1.06140

H 4.78862 2.22155 2.76373

H 3.75766 3.35331 0.81740

H 3.76012 0.08368 3.61352

H -1.52361 -1.10608 2.47307

H 2.71692 4.24986 -0.77870

H 1.44440 5.08732 -2.73480

H -0.50529 3.73701 -3.58669

H -1.07690 1.60691 -2.42458

H -5.09239 -1.30362 -2.80389

H -2.92151 -2.23512 -3.68986

H -5.03420 0.15702 -0.80201

H -0.22645 1.93339 2.45197

H -2.04616 3.14924 3.63460

H -4.40924 2.89445 2.79402

H -4.82593 1.45905 0.81835

H 3.43795 -1.38058 -3.65101

H 1.87680 0.15769 -2.46571

H -0.80989 -1.65472 -2.51241

H 1.74308 -0.80981 2.45541

H 3.66932 -3.74435 -2.80420

H -1.71478 -3.29688 3.64438

H -0.37606 -5.24800 2.77201

H 1.07702 -4.91233 0.79590

H 2.34197 -4.44172 -0.83546
